# Association between pathologic response and survival after neoadjuvant therapy in lung cancer

**DOI:** 10.1038/s41591-023-02660-6

**Published:** 2023-10-30

**Authors:** Julie Stein Deutsch, Ashley Cimino-Mathews, Elizabeth Thompson, Mariano Provencio, Patrick M. Forde, Jonathan Spicer, Nicolas Girard, Daphne Wang, Robert A. Anders, Edward Gabrielson, Peter Illei, Jaroslaw Jedrych, Ludmila Danilova, Joel Sunshine, Keith M. Kerr, Mia Tran, Judith Bushong, Junliang Cai, Vipul Devas, Jaclyn Neely, David Balli, Tricia R. Cottrell, Alex S. Baras, Janis M. Taube

**Affiliations:** 1 Bloomberg–Kimmel Institute for Cancer Immunotherapy, Johns Hopkins University School of Medicine, Baltimore, MD USA; 2 Hospital Universitario Puerta de Hierro, Madrid, Spain; 3 McGill University Health Center, Montreal, Québec Canada; 4 Institut du Thorax Curie-Montsouris, Institut Curie, Paris, France; 5 Aberdeen Royal Infirmary, Aberdeen, UK; 6 Bristol Myers Squibb, Princeton, NJ USA; 7 Queen’s University, Kingston, Ontario Canada; 8 The Mark Foundation Center for Advanced Genomics and Imaging, Johns Hopkins University School of Medicine, Baltimore, MD USA

**Keywords:** Non-small-cell lung cancer, Cancer immunotherapy

## Abstract

Neoadjuvant immunotherapy plus chemotherapy improves event-free survival (EFS) and pathologic complete response (0% residual viable tumor (RVT) in primary tumor (PT) and lymph nodes (LNs)), and is approved for treatment of resectable lung cancer. Pathologic response assessment after neoadjuvant therapy is the potential analog to radiographic response for advanced disease. However, %RVT thresholds beyond pathologic complete response and major pathologic response (≤10% RVT) have not been explored. Pathologic response was prospectively assessed in the randomized, phase 3 CheckMate 816 trial (NCT02998528), which evaluated neoadjuvant nivolumab (anti-programmed death protein 1) plus chemotherapy in patients with resectable lung cancer. RVT, regression and necrosis were quantified (0–100%) in PT and LNs using a pan-tumor scoring system and tested for association with EFS in a prespecified exploratory analysis. Regardless of LN involvement, EFS improved with 0% versus >0% RVT-PT (hazard ratio = 0.18). RVT-PT predicted EFS for nivolumab plus chemotherapy (area under the curve = 0.74); 2-year EFS rates were 90%, 60%, 57% and 39% for patients with 0–5%, >5–30%, >30–80% and >80% RVT, respectively. Each 1% RVT associated with a 0.017 hazard ratio increase for EFS. Combining pathologic response from PT and LNs helped differentiate outcomes. When compared with radiographic response and circulating tumor DNA clearance, %RVT best approximated EFS. These findings support pathologic response as an emerging survival surrogate. Further assessment of the full spectrum of %RVT in lung cancer and other tumor types is warranted. ClinicalTrials.gov registration: NCT02998528.

## Abstract (web summary)

Analysis of the phase 3 CheckMate 816 trial shows that the depth of pathologic response as assessed by percent residual viable tumor is correlated with event-free survival following neoadjuvant immunotherapy plus chemotherapy, supporting pathologic response as a biomarker of survival.

## Main

With the successes of immune checkpoint inhibitors for advanced cancers, treatments targeting programmed death protein 1 (PD-1) and programmed death ligand 1 (PD-L1) are now being evaluated in earlier stages of cancer, and a standardized system for assessing therapeutic benefit is an unmet need. The gold standard for benefit of cancer therapies is improved overall survival. However, collection of survival data can take as long as 10 years, particularly for earlier-stage disease^1^. Event-free survival (EFS) is currently an accepted surrogate end point for the approval of new neoadjuvant therapies; however, this end point relies heavily on radiographic assessment of tumor recurrence (for example, Response Evaluation Criteria in Solid Tumors (RECIST)), and RECIST has recognized limitations in the context of earlier-stage disease^2,3^. Robust metrics that can be measured early in therapy and are associated with survival outcomes are invaluable for informing treatment decisions; these can also facilitate and expedite clinical trial reporting. Pathologic response provides a rigorous and objective assessment of therapeutic efficacy, and a growing body of data support its role as a surrogate end point for new therapy approval.

Neoadjuvant systemic immunotherapy offers the advantage of enhanced priming of the immune system while higher levels of tumor antigens are present, leading to improved immune surveillance of micrometastatic disease^4^. Tumors resected after neoadjuvant therapy also provide a unique opportunity for pathologic assessment of treatment efficacy, and trials in numerous tumor types have incorporated pathologic response as an independent or coprimary end point. Pathologic complete response (pCR; 0% residual viable tumor (RVT)) has been used as a surrogate end point in neoadjuvant chemotherapy studies^5,6^; additionally, ‘near pCR’ or major pathologic response (MPR; ≤10% RVT) has been suggested empirically as an alternative to pCR due to a larger proportion of patients who experience MPR versus pCR^1^. However, the most clinically meaningful and practical RVT thresholds for immunotherapy-treated tumors are yet to be established.

Neoadjuvant immunotherapy results in recognizable histologic features, and a standardized, pan-tumor scoring system (immune-related pathologic response criteria (irPRC))^7,8^ was developed to capture features of pathologic response, inclusive of immune-mediated tumor regression. This quantitative system is independent of disease location, for example, primary tumor (PT), lymph node (LN) or distant metastasis^7,8^, and scores RVT from 0 to 100%. irPRC for the assessment of %RVT has been used in multiple phase 1 and 2 trials evaluating neoadjuvant immunotherapy across diverse tumor types^9–18^.

In the randomized, phase 3 CheckMate 816 study (NCT02998528), neoadjuvant nivolumab plus chemotherapy significantly improved EFS and pCR (primary end points) versus chemotherapy in patients with resectable non-small-cell lung carcinoma^19^, leading to the first regulatory approval of neoadjuvant chemoimmunotherapy for lung cancer. Since the first report, periadjuvant chemotherapy plus immunotherapy regimens have shown efficacy in this patient population^20,21^. Here, in a prespecified exploratory analysis from CheckMate 816, we report the first in-depth assessment of the full spectrum of %RVT (beyond pCR alone) in both the PT and LNs and its association with EFS. To our knowledge, this represents the first use of this pan-tumor pathologic scoring system in a phase 3 registrational trial for any tumor type and provides evidence to support pathologic assessment of %RVT in both the PT and LNs for patients receiving neoadjuvant regimens that include immunotherapy.

## Results

### Patient population

**Fig. 1:**
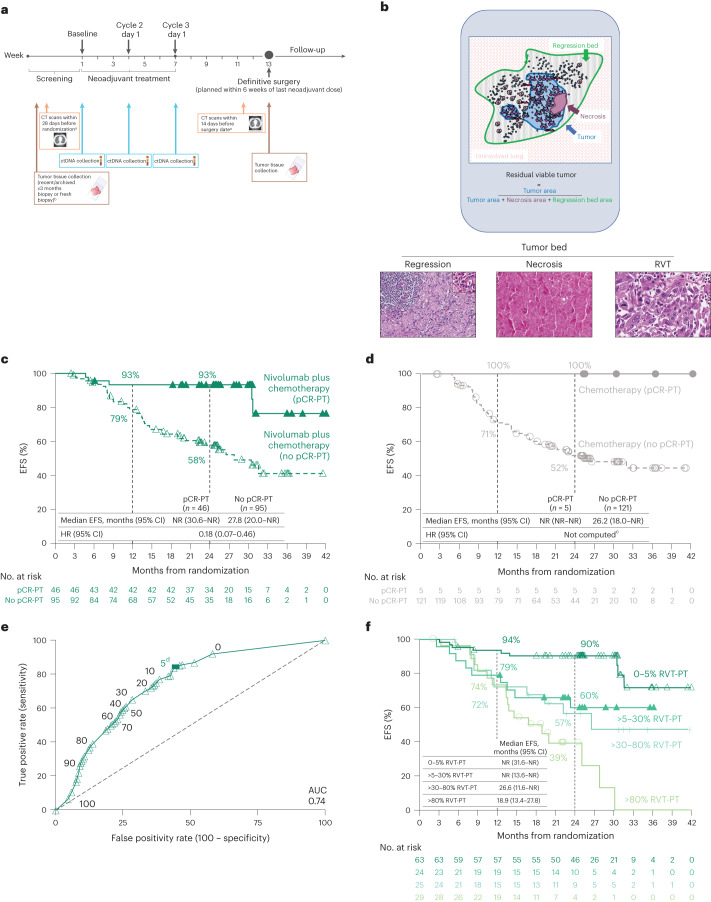
EFS by pCR and RVT. **a**, CheckMate 816 timeline of sample collection for biomarker studies (radiographic imaging (orange), ctDNA (blue) and tumor tissue (brown)). **b**, Schematic of irPRC scoring. Representative photomicrographs show histologic components of the scoring system. The photomicrograph of regression shows a background zone of fibrosis with neovascularization with numerous TIL. A TLS is present in the upper left corner. The inset highlights a collection of plasma cells, which are commonly seen in areas of regression (hematoxylin and eosin staining). **c**,**d**, Kaplan–Meier curves of EFS by pCR status (PT) in the path-evaluable patient population in the nivolumab plus chemotherapy arm (**c**) and in the chemotherapy arm (**d**). **e**, ROC curve analysis of 2-year EFS rate by %RVT (PT) in the path-evaluable patient population for patients treated with nivolumab plus chemotherapy. **f**, Kaplan–Meier curves of EFS by %RVT categories (PT) in the path-evaluable patient population for patients treated with nivolumab plus chemotherapy. Database lock: 20 October 2021; minimum follow-up: 21 months for nivolumab plus chemotherapy and chemotherapy arms; median follow-up: 29.5 months. CT, computed tomography; EBUS, endobronchial ultrasound; NR, not reached; TIL, tumor infiltrating lymphocyte; TLS, tertiary lymphoid structure. ^a^Using RECIST1.1. ^b^Mediastinal lymph node sampling. All suspicious mediastinal lymph nodes require sampling for pathologic confirmation if accessible by EBUS, mediastinoscopy or thoracoscopy. ^c^HR was not computed for the chemotherapy arm owing to only five patients having a pCR-PT. ^d^The solid square is the optimal cutoff, which is the difference between the true positive rate and false positive rate over all possible cutoff values.

**Extended Data Fig. 1:**
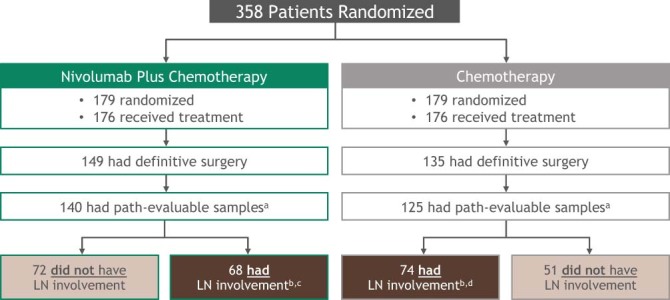
CheckMate 816 analysis population. Database lock: October 20, 2021; minimum follow-up: 21 months; median follow-up, 29.5 months. LN denotes lymph node; RVT, residual viable tumor. ^a^Represents patients with path-evaluable samples from both the primary tumor and LN; 141 and 126 patients in the nivolumab plus chemotherapy arm and the chemotherapy arm, respectively, had path-evaluable samples from the primary tumor only. ^b^Pathologic evidence of ≥0% RVT in LNs. ^c^94 patients and ^d^71 patients had LN involvement based on baseline radiographic imaging in the path-evaluable population; LN involvement refers to pathologic evidence of LN disease at resection that had or had not fully regressed after neoadjuvant treatment (0% or >0% RVT in the resected LN).

Clinical and biomarker assessments were performed during the course of the trial (Fig. 1a,b). Of 358 patients concurrently randomized to the nivolumab plus chemotherapy or chemotherapy arms, pathologically evaluable samples from the PT were available in 141 and 126 patients, respectively (path-evaluable patient population; Extended Data Fig. 1). Baseline characteristics in the path-evaluable patient population were consistent with those of the overall population^19^ and well-balanced across treatment arms (Table 1). Baseline characteristics were generally similar in patients with and without pathologic evidence of LN involvement. Approximately one third of patients received optional adjuvant therapy, which is summarized by subpopulation in Supplementary Table 1. Forty-seven patients had matched pretreatment and on-treatment specimens available.

**Table 1:** Baseline characteristics: all path-evaluable patients and patients with or without pathologic evidence of LN involvement

	Path-evaluable population^a^					
	All		With LN involvement^b^		Without LN involvement^b^	
	Nivolumab plus chemotherapy (*n* = 141)	Chemotherapy (*n* = 126)	Nivolumab plus chemotherapy (*n* = 68)	Chemotherapy (*n* = 74)	Nivolumab plus chemotherapy (*n* = 72)	Chemotherapy (*n* = 51)
**Age, median (range), years**	64 (46–82)	65 (34–83)	64 (47–76)	65 (34–84)	67 (49–80)	65 (46–82)
**Male**	69	71	72	72	65	69
**Region**^**c**^						
North America	26	29	26	27	25	33
Europe	20	10	15	12	25	6
Asia	49	55	50	54	47	57
**ECOG PS**						
0	75	68	75	73	75	59
1	25	32	25	27	25	41
**Stage**^**d,e**^						
IB–II	37	39	26	32	46	47
IIIA	63	61	74	68	54	53
**Histology**						
Squamous	47	52	46	51	47	51
Nonsquamous	53	48	54	49	53	49
**Smoking status**^**f**^						
Current/former	89	87	88	85	90	92
Never	11	12	12	15	10	6
**Tumor PD-L1 expression**^**g**^						
Not evaluable	8	8	10	12	6	2
<1%	44	46	48	34	40	63
≥1%	48	46	41	54	54	35
1–49%	29	29	16	35	42	20
≥50%	19	18	25	19	12	16
**TMB**^**h**^						
Not evaluable/not reported^i^	50	48	56	51	46	43
<12.3 mut/Mb	29	29	28	30	29	28
≥12.3 mut/Mb	21	23	16	19	25	29

Data reported as % unless otherwise noted.

ECOG PS, Eastern Cooperative Oncology Group performance status.

^a^Path-evaluable: patients who underwent surgery and had pathologically evaluable samples.

^b^Among 179 patients randomized to both the nivolumab plus chemotherapy and chemotherapy groups, 149 and 135 received treatment and had definitive surgery, respectively, and 140 and 125 had path-evaluable samples from both PT and LN; LN involvement refers to pathologic evidence of LN disease at resection that had or had not fully regressed after neoadjuvant treatment (0% or >0% RVT in the resected LN).

^c^Rest of the world: 6% of patients in the nivolumab plus chemotherapy and chemotherapy arms (path-evaluable patient population), 9% and 7% of patients in the nivolumab plus chemotherapy and chemotherapy arms (with LN involvement), 3% and 4% of patients in the nivolumab plus chemotherapy and chemotherapy arms (without LN involvement).

^d^Disease stage by case report form, per American Joint Committee on Cancer 7th edition.

^e^Stage IB, IIA, IIB disease: 6%, 16% and 15% of patients in the nivolumab plus chemotherapy arm and 3%, 21% and 14% in the chemotherapy arm, respectively (path-evaluable patient population); 3%, 16% and 7% of patients in the nivolumab plus chemotherapy arm and 3%, 24% and 5% in the chemotherapy arm, respectively (with LN involvement); 8%, 17% and 21% of patients in the nivolumab plus chemotherapy arm and 4%, 18% and 26% in the chemotherapy arm, respectively (without LN involvement).

^f^Smoking status unknown: one patient in the chemotherapy arm (path-evaluable patient population); one patient in the chemotherapy arm (without LN involvement).

^g^Level of PD-L1 expression was determined using the PD-L1 IHC 28-8 pharmDx assay (Dako); patients with tumor tissue that could not be assessed for PD-L1 (≤10% of concurrently randomized patients) were stratified to the PD-L1 expression <1% subgroup at randomization.

^h^TMB was evaluated using the Illumina TSO500 assay. A 12.3-mut/Mb cutoff per TSO500 corresponds to 10 mut/Mb per the FoundationOne assay.

^i^TMB was not analyzed for patients in China; these patients are included in the ‘not reported’ category.

### RVT and EFS

**Extended Data Fig. 2:**
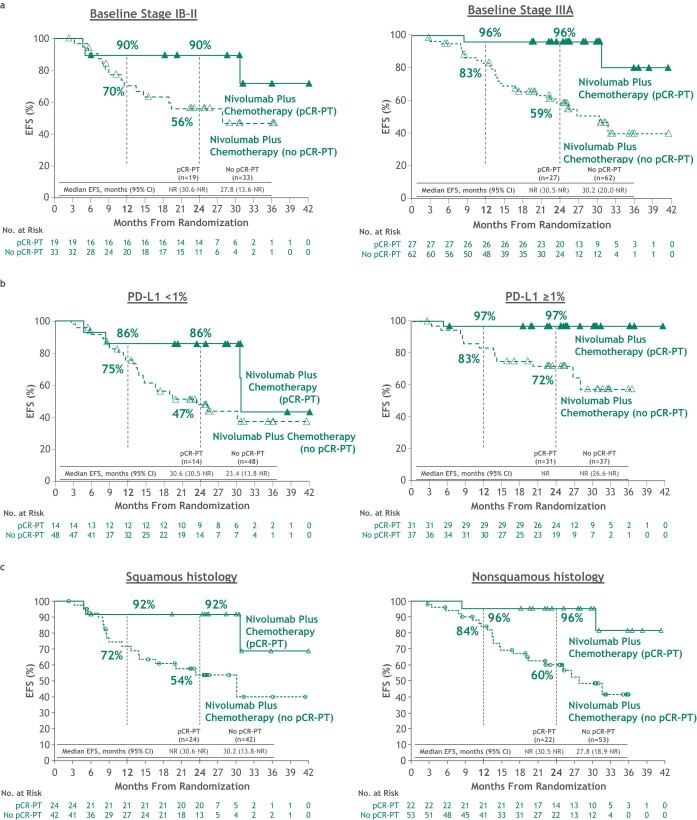
Kaplan–Meier curves of EFS by pCR status (PT) and disease characteristics in the path-evaluable patient population from the nivolumab plus chemotherapy arm. **a**, Kaplan–Meier curves of EFS by pCR status (PT) and baseline disease stage. **b**, Kaplan–Meier curves of EFS by pCR status (PT) and baseline PD-L1 expression. **c**, Kaplan–Meier curves of EFS by pCR status (PT) and histology. Subgroup analyses were not performed for the chemotherapy arm because of small sample sizes. HRs were not computed because of low number of events for the pCR-PT subgroups. CI denotes confidence interval; EFS, event-free survival; HR, hazard ratio; NR, not reached; pCR, pathologic complete response; PD-L1, programmed death ligand 1; PT, primary tumor.

**Extended Data Fig. 3:**
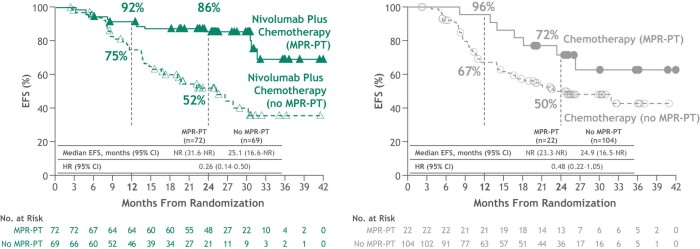
Kaplan–Meier curves of EFS by MPR status (PT) in the path-evaluable patient population. CI denotes confidence interval; EFS, event-free survival; HR, hazard ratio; MPR, major pathologic response; NR, not reached; PT, primary tumor.

The prognostic relevance of RVT in surgical resection specimens was investigated through the association of RVT and EFS in all path-evaluable patients. Patients with no RVT in the PT (pCR-PT) had improved EFS versus those with RVT in both treatment arms (Fig. 1c,d; hazard ratio (HR) = 0.18 for the nivolumab plus chemotherapy arm); however, only 5 of 126 patients (4.0%) in the chemotherapy arm had a pCR-PT, as compared with 46 of 141 in the nivolumab plus chemotherapy arm (32.6%). The association of pCR-PT with EFS was observed regardless of baseline disease stage, PD-L1 expression, and squamous or nonsquamous histology (Extended Data Fig. 2). A threshold of ≤10% RVT (that is, MPR-PT) was also able to identify patients with EFS benefit in both the nivolumab plus chemotherapy and chemotherapy arms (HR = 0.26 and 0.48, respectively; Extended Data Fig. 3), consistent with previous reports for chemotherapy^1,22^. The HR for progression associated with 1% increase of RVT was 1.017 (95% confidence interval (CI): 1.010–1.025), suggesting that each 1% increase in residual tumor is associated with a 0.017 increase in HR for EFS.

**Extended Data Fig. 4:**
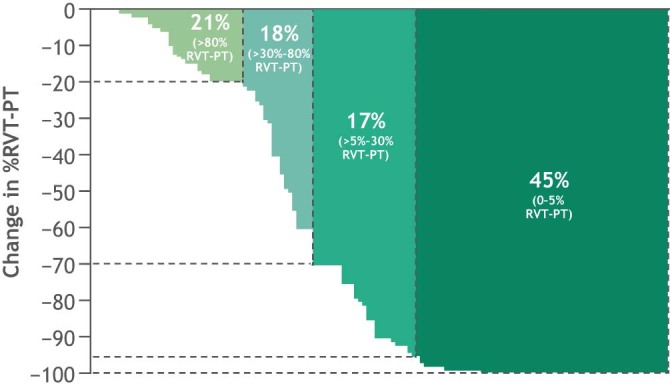
Depth of pathologic response (%RVT) in the PT of path-evaluable patients receiving nivolumab plus chemotherapy. The optimal value for %RVT (5% RVT) was calculated from the receiver operating characteristic curve analysis using the Youden’s index (that maximizes the true positive rate and minimizes the false positive rate). PT denotes primary tumor; RVT, residual viable tumor.

Receiver operating characteristic (ROC) curve analysis of 2-year EFS rate by %RVT-PT (as a continuous variable) showed that %RVT-PT was predictive of EFS at 2 years for nivolumab plus chemotherapy (area under the curve (AUC) = 0.74; Fig. 1e). The distribution of the depth of pathologic response (Extended Data Fig. 4) was used to choose thresholds for the EFS subgroup analysis. Patients with RVT-PT 0–5%, >5–30%, >30–80% and >80% had 2-year EFS rates of 90%, 60%, 57% and 39%, respectively (Fig. 1f), suggesting that patients with deeper pathologic response have better EFS outcomes at 2 years. The ROC analysis for the chemotherapy arm was limited by a narrow range of pathologic response for %RVT-PT and fewer surviving patients at 24 months compared with the nivolumab plus chemotherapy arm (AUC = 0.54).

### Regression, necrosis, PD-L1, tumor mutational burden and treatment-related adverse events

**Fig. 2:**
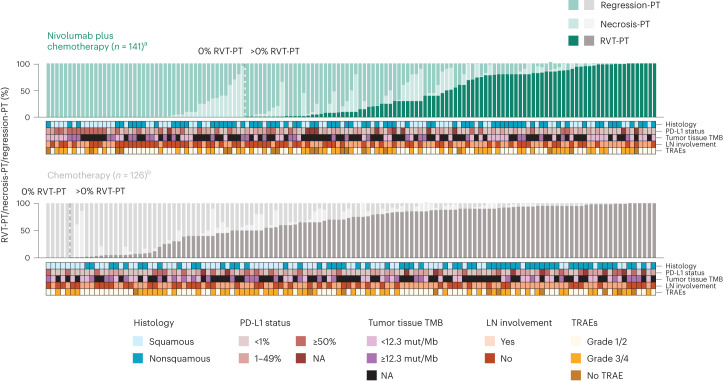
PT pathologic features in patients with path-evaluable samples. Pathologic features (histology, tumor PD-L1 status, tumor tissue TMB, LN involvement and TRAEs) for the path-evaluable population in the nivolumab plus chemotherapy (*n* = 141) and chemotherapy (*n* = 126) arms. LN involvement: yes indicates patients with pathologic evidence of LN disease at resection that had fully regressed (0% RVT) or had not regressed (>0% RVT) after neoadjuvant treatment. NA, not available. ^a^Median %RVT for nivolumab plus chemotherapy was 10.0% (squamous), 10.0% (nonsquamous), 35.0% (PD-L1 < 1%), 8.0% (PD-L1 1–49%), 0 (PD-L1 ≥ 50%), 30.0% (PD-L1 NA), 40.0% (TMB < 12.3 mut/Mb), 1.0% (TMB ≥ 12.3 mut/Mb), 10.0% (TMB NA), 35.0% (LN involvement), 1.0% (no LN involvement), 10.0% (TRAEs tissue grade 1/2), 23.0% (TRAEs grade 3/4) and 5.0% (No TRAE). ^b^Median %RVT for chemotherapy was 56.0% (squamous), 88.0% (nonsquamous), 69.0% (PD-L1 < 1%), 81.0% (PD-L1 1–49%), 60.0% (PD-L1 ≥ 50%), 94.0% (PD-L1 NA), 76.0% (TMB < 12.3 mut/Mb), 56.0% (TMB ≥ 12.3 mut/Mb), 77.0% (TMB NA), 82.5% (LN involvement), 62.5% (no LN involvement), 75.0% (TRAEs grade 1/2), 65.0% (TRAEs grade 3/4) and 90.5% (No TRAE).

**Extended Data Fig. 5:**
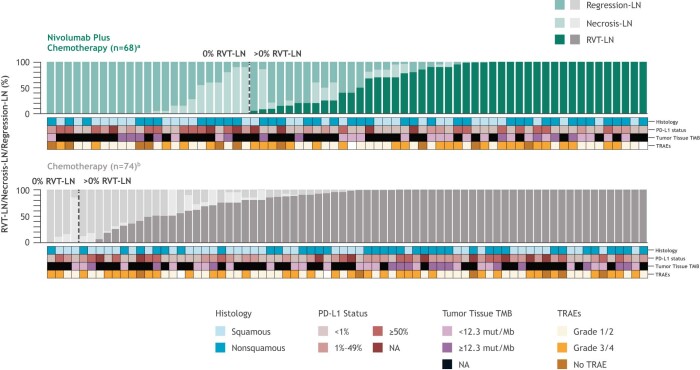
Pathologic features in patients with LN involvement. Pathologic features (histology, tumor PD-L1 status, tumor tissue TMB, and TRAEs) for the path-evaluable population with LN involvement in the nivolumab plus chemotherapy (n = 68) and chemotherapy (n = 74) arms. LN denotes lymph node; mut/Mb, mutations/megabase; NA, not available; PD-L1, programmed death ligand 1; RVT, residual viable tumor; TMB, tumor mutational burden; TRAE, treatment-related adverse event. ^a^Median %RVT for nivolumab plus chemotherapy was 25.0% (squamous), 50.0% (nonsquamous), 70.0% (PD-L1 < 1%), 25.0% (PD-L1 1–49%), 0 (PD-L1 ≥ 50%), 5.0% (PD-L1 NA), 90.0% (TMB < 12.3 mut/Mb), 8.0% (TMB ≥ 12.3 mut/Mb), 17.5% (TMB NA), 32.5% (TRAEs grade 1/2), 84.0% (TRAEs grade 3/4), and 0 (No TRAE). ^b^Median %RVT for chemotherapy was 83.5% (squamous), 99.5% (nonsquamous), 85.0% (PD-L1 < 1%), 99.5% (PD-L1 1–49%), 97.5% (PD-L1 ≥ 50%), 100% (PD-L1 NA), 96.0% (TMB < 12.3 mut/Mb), 100% (TMB ≥ 12.3 mut/Mb), 88.5% (TMB NA), 93.0% (TRAEs grade 1/2), 95.0% (TRAEs grade 3/4), and 100% (No TRAE).

In addition to RVT, the proportions of necrosis and regression in the PT bed and LN were scored as part of response assessment (Fig. 2 and Extended Data Fig. 5) and provide insight into treatment effects. The relationships of these components to each other and to patient characteristics were also determined. There was an inverse relationship between %RVT and %regression in both arms. Patients in the nivolumab plus chemotherapy arm showed lower %RVT and higher %regression in both the PT and LN than those in the chemotherapy arm (Fig. 2). In contrast, median necrosis was similar between the two treatment arms, and no relationships between necrosis and RVT or regression were observed in either treatment arm. The pathologic and baseline clinical characteristics (histologic subtype, PD-L1 or tumor mutational burden (TMB) status; occurrence of treatment-related adverse events (TRAEs); pathologic evidence of LN involvement) are shown in Fig. 2 and Supplementary Table 2, and no clear patterns of association were observed between depth of pathologic response and these features, though some subgroups were small. Specifically, occurrence of TRAEs was similar in patients with or without pCR-PT/MPR-PT (Supplementary Table 3).

**Fig. 3:**
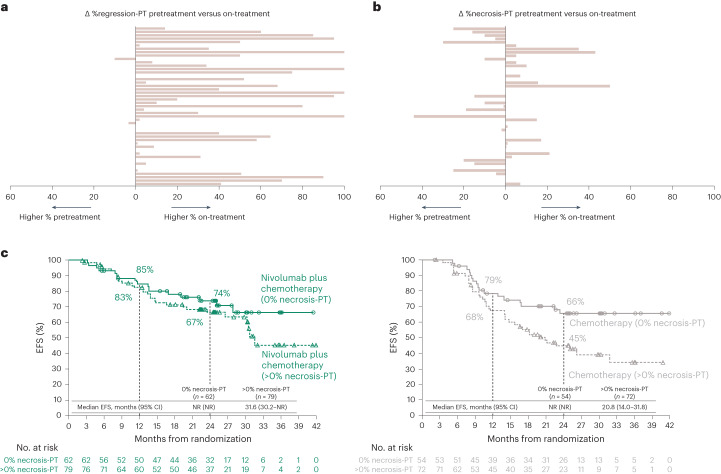
PT regression and necrosis and EFS in the path-evaluable patient population. **a**,**b**, Difference in %regression-PT (**a**) and %necrosis-PT (**b**) between paired pretreatment and on-treatment tumor tissue specimens from individual patients (*n* = 47). **c**, Kaplan–Meier curves showing EFS by %necrosis-PT (0% versus >0%) in on-treatment specimens for all path-evaluable patients treated with nivolumab plus chemotherapy and chemotherapy.

**Extended Data Fig. 6:**
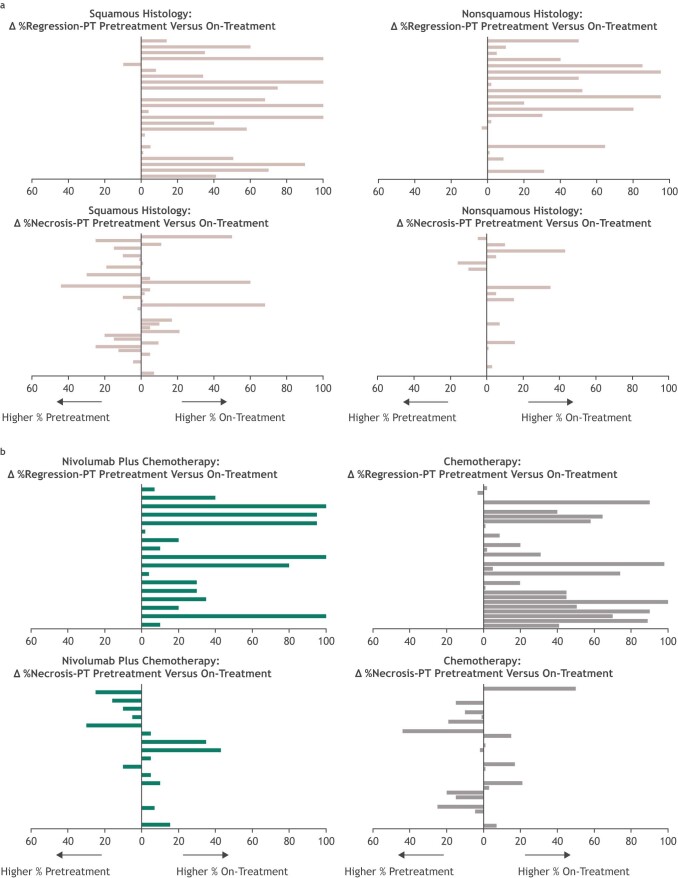
PT regression and necrosis in the path-evaluable patient population. Paired pretreatment and on-treatment %regression-PT and %necrosis-PT in tissue specimens from individual patients by **(a)** histology (n = 24, squamous; n = 23, nonsquamous) and **(b)** treatment arm (n = 17, nivolumab plus chemotherapy; n = 30, chemotherapy). PT denotes primary tumor.

**Extended Data Fig. 7:**
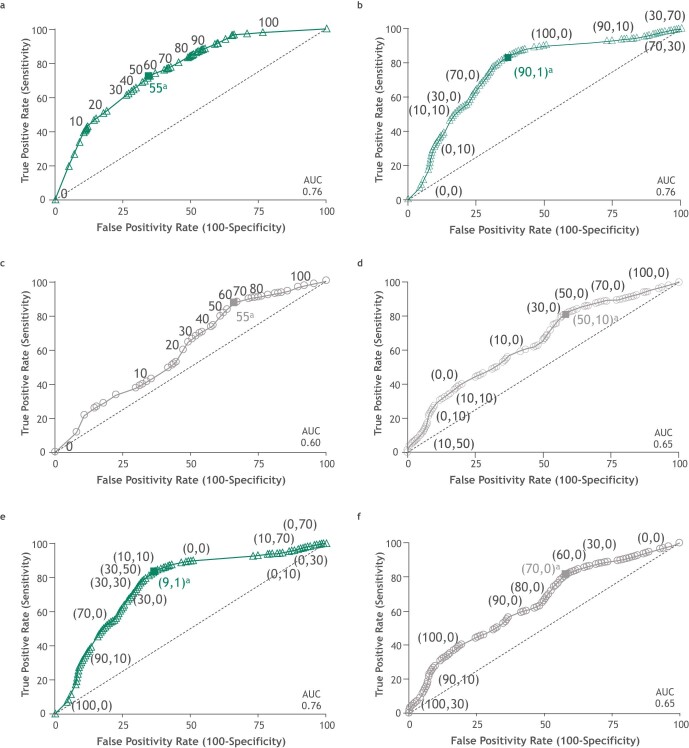
ROC curve analyses for regression and necrosis (PT) and combined %RVT plus necrosis (PT) in the path-evaluable patient population. **a**, ROC curve analysis of 2-year EFS rate by %regression-PT for patients treated with nivolumab plus chemotherapy. AUC = 0.76, similar to that for %RVT-PT. **b**, ROC curve analysis of 2-year EFS rate by %regression + necrosis-PT for patients treated with nivolumab plus chemotherapy; points are labeled as ‘(%regression-PT, %necrosis-PT)’. Addition of %necrosis-PT to %regression-PT did not increase AUC. **c**, ROC curve analysis of 2-year EFS rate by %regression-PT for patients treated with chemotherapy. **d**, ROC curve analysis of 2-year EFS rate by %regression + necrosis-PT for patients treated with chemotherapy; points are labeled as ‘(%regression-PT, %necrosis-PT)’. Considering necrosis may be a tumor-intrinsic parameter, it was combined with %RVT-PT in patients receiving nivolumab plus chemotherapy (**e**), and in patients receiving chemotherapy (**f**). However, %RVT + necrosis-PT did not greatly alter the AUC achieved with %RVT-PT alone. Points are labeled as ‘(%RVT-PT, %necrosis-PT)’. Nivolumab plus chemotherapy arm, n = 141; chemotherapy arm, n = 126. EFS denotes event-free survival; PT, primary tumor; ROC, receiver operating characteristic; RVT, residual viable tumor. ^a^The solid square is the optimal cutoff, which is the difference between the true positive rate and false positive rate over all possible cutoff values.

To further delineate treatment effect, paired pretreatment and on-treatment pathologic specimens were compared for %regression and %necrosis. Although most pretreatment specimens showed no evidence of tumor regression, a subset showed features consistent with a degree of spontaneous immune-mediated regression (Fig. 3a and Extended Data Fig. 6a), which has been reported in lung cancer^23^. Features of immune-mediated regression generally increased on therapy. In contrast, a systematic increase in %necrosis-PT after therapy was not observed (Fig. 3b and Extended Data Fig. 6b). The relationship of these features with therapeutic effect or a lack thereof was affirmed with 2-year EFS analyses, namely %regression reflects overall treatment effect on the clinical outcome, whereas %necrosis does not (Extended Data Fig. 7). In fact, on-treatment necrosis was associated with lower EFS rates, particularly in the chemotherapy arm (Fig. 3c; 2-year EFS rates for absence versus presence of necrosis: 74% versus 67% for nivolumab plus chemotherapy, and 66% versus 45% for chemotherapy).

### LN involvement

**Fig. 4:**
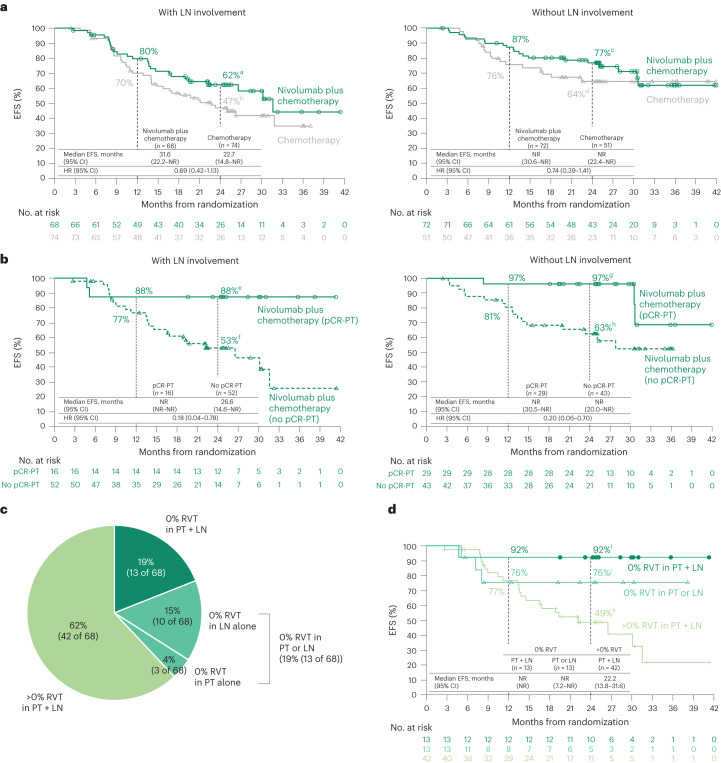
Treatment efficacy in patients with or without pathologic evidence of LN involvement. **a**, Kaplan–Meier curves showing EFS by LN involvement. Among 358 patients concurrently randomized to the nivolumab plus chemotherapy and chemotherapy arms, 149 and 135 received treatment and had definitive surgery, respectively, and 140 and 125 had path-evaluable samples from both PT and LNs. **b**, Kaplan–Meier curves showing EFS by pCR status (PT) in patients with or without pathologic evidence of LN involvement who received nivolumab plus chemotherapy. **c**, Percent RVT in PT and LNs in patients receiving nivolumab plus chemotherapy. For comparison, in the chemotherapy arm: 0% RVT in both PT + LNs, 1% (1 of 74); in PT alone, 1% (1 of 74); in LNs alone, 4% (3 of 74); in either PT or LNs, 5% (4 of 74); and >0% RVT in PT + LNs, 93% (69 of 74). **d**, Kaplan–Meier curves showing EFS by %RVT in PT and LNs in patients receiving nivolumab plus chemotherapy. HRs were not computed because of the low number of events in the 0% RVT subgroups. LN involvement refers to pathologic evidence of LN disease at resection that had or had not fully regressed after neoadjuvant treatment (0% or >0% RVT in the resected LNs). 95% CIs: ^a^49–73, ^b^34–58, ^c^65–85, ^d^49–76, ^e^59–97, ^f^37–66, ^g^78–100, ^h^46–76, ^i^57–99, ^j^42–91, ^k^32–64.

**Extended Data Fig. 8:**
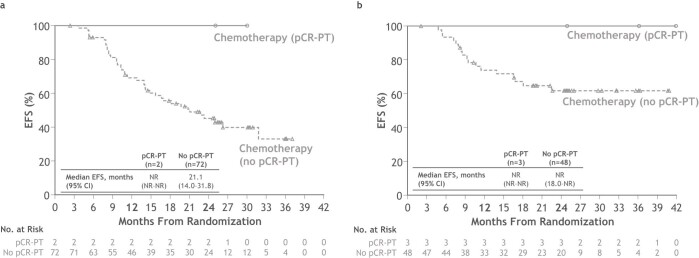
Kaplan–Meier curves of EFS by pCR status (PT) in patients with or without pathologic evidence of LN involvement. **a**, Patients with LN involvement who received chemotherapy. **b**, Patients without LN involvement who received chemotherapy. CI denotes confidence interval; EFS, event-free survival; LN, lymph node; pCR, pathologic complete response; PT, primary tumor.

**Extended Data Fig. 9:**
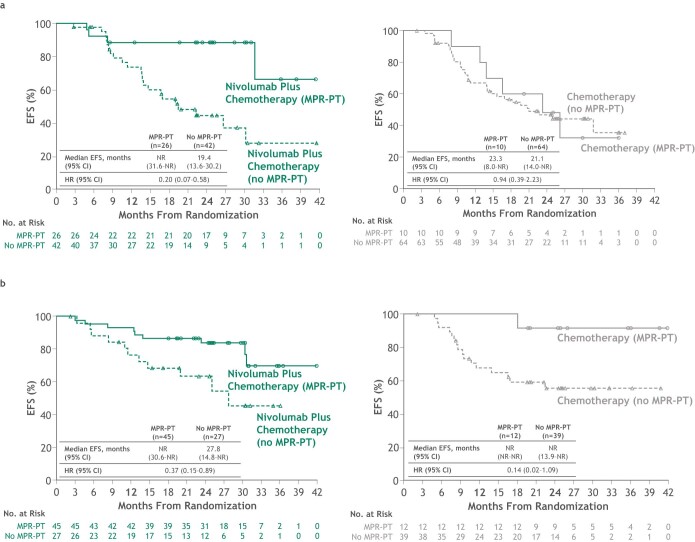
Kaplan–Meier curves of EFS by MPR status (PT) in patients with or without pathologic evidence of LN involvement. **a**, Patients with LN involvement who received nivolumab plus chemotherapy and chemotherapy. **b**, Patients without LN involvement who received nivolumab plus chemotherapy and chemotherapy. CI denotes confidence interval; EFS, event-free survival; LN, lymph node; MPR, major pathologic response; PT, primary tumor.

EFS favored nivolumab plus chemotherapy versus chemotherapy in patients with or without pathologic evidence of LN involvement (HR = 0.69 and 0.74, respectively, Fig. 4a). The relationships between pCR-PT or MPR-PT and EFS in both arms were maintained regardless of LN involvement (Fig. 4b and Extended Data Figs. 8 and 9). Notably in the nivolumab plus chemotherapy arm, patients who had pathologic evidence of LN involvement and then had a complete response in the LN (0% RVT-LN) had comparable survival to those who had no pathologic evidence of ever having LN involvement (86% versus 77% EFS at 2 years by Kaplan–Meier analysis; number at risk: 13 and 43, respectively).

The prognostic value of %RVT was further demonstrated by combining assessment of the PT and LNs (Fig. 4c). A graduated improvement in EFS was observed in patients with LN involvement who had 0% RVT in both the PT and LNs versus 0% RVT in either specimen versus those with >0% RVT in both specimens (2-year EFS rates: 92%, 76% and 49%, respectively; Fig. 4d), indicating that pathologic assessment of LNs provides additional prognostic information beyond the PT alone.

### Pathology versus radiology and circulating tumor DNA

**Fig. 5:**
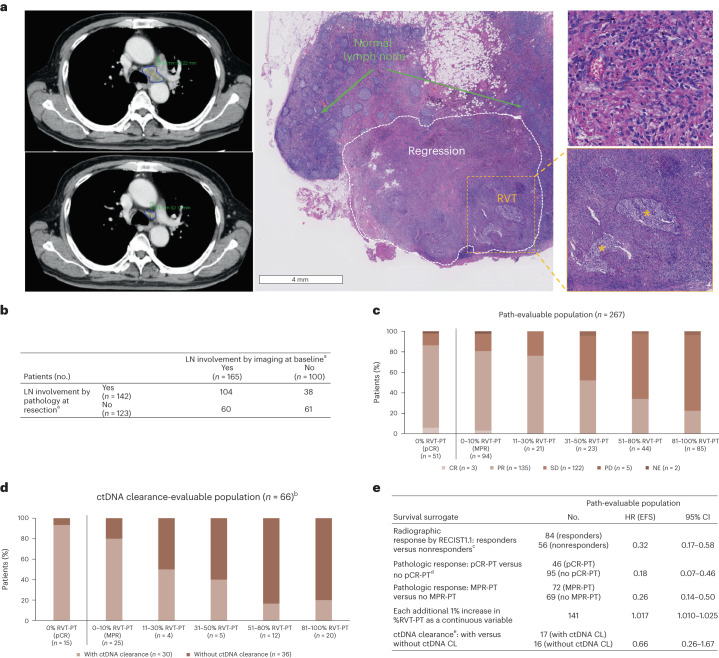
Relationship between pathology and other clinical/biomarker correlates. **a**, Representative case showing the potential disconnect between assessment of LN involvement on radiology and pathology. Top left, the pretreatment CT scan showed an enlarged LN. Bottom left, the CT scan just before surgical resection demonstrated an approximately 50% reduction in the size of the LN. Right, photomicrograph of the same LN in the definitive resection specimen showed an MPR (90% reduction in tumor), highlighting the underestimation of pathologic response by imaging. The regression bed is surrounded by a white dotted line and is shown on higher power in top right. RVT is shown in the lower right and is marked by yellow asterisks. **b**, Patients with LN involvement by imaging at baseline and by pathology at resection, across treatment arms. **c**, Radiographic (BOR per RECIST1.1) and pathologic response (%RVT) in PT, across treatment arms. **d**, ctDNA clearance and pathologic response (%RVT) in PT, across treatment arms. **e**, Association of EFS and survival surrogates for patients receiving nivolumab plus chemotherapy. BOR, best overall response; CL, clearance; CR, complete response; NE, not evaluable; PD, progressive disease; PR, partial response; RECIST1.1, Response Evaluation Criteria in Solid Tumors version 1.1; SD, stable disease. ^a^Data were not reported for two patients. ^b^ctDNA data were not available/not evaluable for 201 patients. ^c^Responders = CR + PR; nonresponders = SD + PD. ^d^In the concurrently randomized population, 43 patients had 0% RVT in PT + LN versus 136 without 0% RVT in PT + LN (HR, 0.13; 95% CI, 0.05–0.37)^19^. ^e^In the concurrently randomized population, 24 patients had ctDNA CL versus 19 without ctDNA CL (HR, 0.60; 95% CI, 0.20–1.82)^19^.

**Extended Data Fig. 10:**
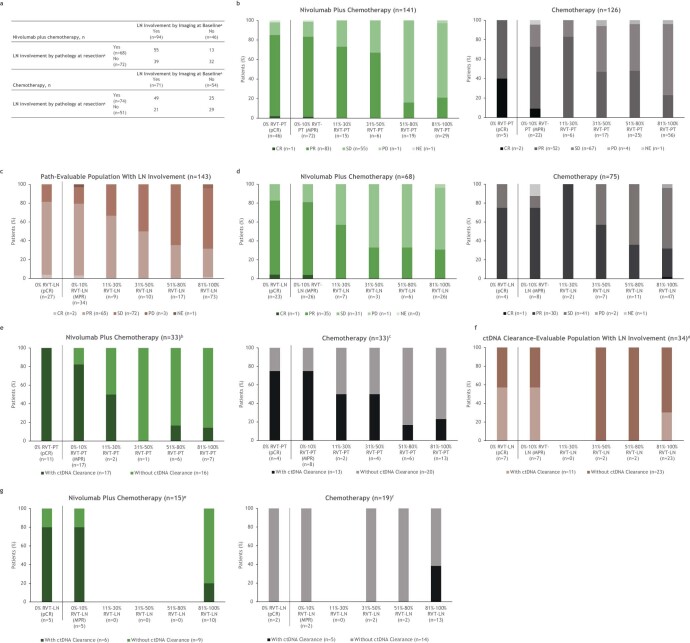
Relationship between radiology and pathology and other clinical/biomarker correlates. **a**, Patients with LN involvement by imaging at baseline and by pathology at resection by treatment arm. **b**, Radiographic (BOR per RECIST1.1) and pathologic response (%RVT) in PT. **c**, Radiographic (BOR per RECIST1.1) and pathologic response (%RVT) in LN, across treatment arms. **d**, Radiographic (BOR per RECIST1.1) and pathologic response (%RVT) in LN by treatment arm. **e**, ctDNA clearance and pathologic response (%RVT) in PT. **f**, ctDNA clearance and pathologic response (%RVT) in LN, across treatment arms. **g**, ctDNA clearance and pathologic response (%RVT) in LN by treatment arm. BOR denotes best overall response; CR complete response; LN, lymph node; MPR, major pathologic response; NE, not evaluable; pCR, pathologic complete response; PD, progressive disease; PR, partial response; PT, primary tumor; RECIST1.1, Response Evaluation Criteria in Solid Tumors version 1.1; RVT, residual viable tumor; SD, stable disease. ^a^Data were not reported for 1 patient. ^b^ctDNA data were not available/not evaluable for 108 patients. ^c^ctDNA data were not available/not evaluable for 93 patients. ^d^ctDNA data were not available/not evaluable for 109 patients. ^e^ctDNA data were not available/not evaluable for 53 patients. ^f^ctDNA data were not available/not evaluable for 56 patients.

The relationship between %RVT and other proposed clinical and translational correlates was also assessed. Radiology did not fully manifest underlying %RVT-PT or %RVT-LN (Fig. 5a–c and Extended Data Fig. 10). Although patients with complete response or partial response by imaging tended to have lower %RVT-PT than those with stable disease or progressive disease, only 6% of patients (3 of 51) with pCR-PT demonstrated complete response by RECIST1.1. Furthermore, 19% (18 of 94) who had MPR-PT did not show a radiographic response. When pathology and radiology assessment of LN involvement were compared, radiographic studies suggested LN involvement in 36% of cases, but no evidence of involvement was evident on pathology (Fig. 5a,b). Conversely, 27% of patients who had LN involvement on pathology were not suspected to have nodal disease on radiology.

The association of circulating tumor DNA (ctDNA) clearance with pathologic response in the PT was also explored. A higher rate of ctDNA clearance was seen in patients with deeper pathologic responses (0–10% RVT-PT) in the nivolumab plus chemotherapy arm, although small numbers of patients in several %RVT categories limited subset analysis (Fig. 5d). Notably, 9% (6 of 66) had ctDNA clearance but demonstrated >50% RVT-PT. Furthermore, whereas 267 patients were path-evaluable, only 66 (25%) of these were evaluable for ctDNA clearance, highlighting the current technical and material challenges with this approach. When ctDNA clearance, radiographic response and pathologic response were investigated as surrogates for EFS in the path-evaluable patient population, pathologic response most closely estimated outcome (Fig. 5e).

## Discussion

In CheckMate 816, neoadjuvant nivolumab plus chemotherapy significantly improved both primary end points of EFS and pCR versus chemotherapy in patients with resectable lung cancer^19^. Here, we showed improved EFS in both arms for patients with pCR or MPR in the PT only, irrespective of LN involvement. Furthermore, the depth of pathologic response (%RVT-PT) was associated with improved EFS outcomes. We also demonstrated the prognostic value of other pathologic features and of combining %RVT in the PT and LNs.

The United States Food and Drug Administration requires that a surrogate end point supporting accelerated approval be at least “reasonably likely to predict clinical benefit”^24^. In contrast to correlates, surrogates not only associate with outcome, but also manifest overall treatment effect on the clinical outcome^24,25^. The substantiation of a surrogate requires large cohorts, as are found in phase 3 clinical trials such as CheckMate 816 or robust meta-analyses. Results of the current study provide prospective evidence of pathologic response assessment that includes immune-mediated regression as an emerging surrogate for outcome in the neoadjuvant immuno-oncology setting.

Radiographic response by RECIST has been used as a surrogate for long-term clinical benefit and to support accelerated approvals in oncology for patients with advanced, unresectable disease^26^. It also serves as a guide for the treating oncologist^27^. However, it has limitations when applied to neoadjuvant therapy, as further highlighted by the radiographic–pathologic disconnect in both the PT and LNs. In the neoadjuvant setting, pathologic response is poised to parallel the role of RECIST in advanced disease with regard to surrogacy for regulatory approvals, given that it is available within 2 to 3 months after treatment initiation. It also has the potential to inform adjuvant therapy decisions. Here, we used thresholds of 5% and 80% RVT to separate patients receiving nivolumab plus chemotherapy into three distinct prognostic groups. Although the final determination of clinically meaningful thresholds will require a larger number of patients, %RVT thresholds could be used to determine whether to de-escalate therapy (for example, ≤5–10% RVT at resection), continue therapy in the adjuvant setting or consider an alternative adjuvant regimen (for example, >80% RVT). It is worth noting that potential clinically relevant cut points, for example, the 80% RVT identified here, require the assessment of the full spectrum of %RVT to be captured. To date, reports on immunotherapy-containing regimens in lung have only reported pCR and MPR, with a focus on PT. In this study, approximately one third of patients experienced pCR or MPR in the PT. However, capturing the full spectrum of %RVT as performed herein allows for the refined prognostication and potential adaptive management for the remaining two thirds of patients.

The percent RVT in this study was determined using the pan-tumor irPRC. This scoring system was developed using a data-driven approach that compared pre- versus on-treatment specimens and responders versus nonresponders, as well as studying more than ten different solid tumor types treated in both the neoadjuvant and advanced disease settings. The assessment of treatment effect has historically varied by tumor type, including different approaches to the calculation of %RVT (surface area of RVT divided by the surface area of where tumor used to be). The variability exists largely in guidance for determining histologically where tumor used to be, that is, the denominator of the calculation. The goal of the pan-tumor effort was to identify histologic features of treatment effect (including immune-mediated regression) that are common across tumor types, disease stage and anatomic location^7,8^, analogous to a RECIST radiographic assessment. Importantly, this quantitative system also accounts for histopathologic features observed in patients treated with chemotherapy^8^. This has the advantage of allowing pathologists to use one scoring system, rather than asking them to change between systems based on drug indication, treatment arm and/or tumor type. Capturing and reporting %RVT using such a pan-tumor scoring system will facilitate important meta-analyses across trials and tumor types and better determination of objective and generalizable cut points.

Owing to the lack of a preexisting, established pan-tumor precedent for scoring pathologic response, some individual disease groups developed recommendations for pathologic response scoring, including the International Association for the Study of Lung Cancer (IASLC). The development of their recommendations postdated the start of this study; notwithstanding, irPRC is generally inclusive of these recommendations, albeit with some minor differences, for example, irPRC and IASLC both score histologic components that include fibrosis, inflammation, necrosis and residual tumor. The notable difference between the two systems is that irPRC distinguishes noninflamed tumor stroma from the fibroinflammatory wound-healing response seen in tumor regression, whereas IASLC does not. The finding that %regression is as predictive as %RVT in this study underscores the notion that regression represents an important tissue class with biologic importance. Both systems have been shown to be reproducible for the scoring of %RVT in the PT, but IASLC has not yet reported on the reproducibility of LN scoring^28^. A Society for Immunotherapy of Cancer-led multi-institutional reproducibility study for pan-tumor irPRC that includes 14 international pathologists and numerous tumor types beyond lung cancer is currently underway, with an interim analysis showing an intraclass correlation coefficient of 0.88 for assessing %RVT at 10% intervals. In subset analysis, similar reproducibility was seen for both the PTs and LNs (intraclass correlation coefficient = 0.88, for each)^29^.

Immune-mediated regression is characterized by (1) immune activation—tumor-infiltrating lymphocytes (TILs) with macrophages and variable tertiary lymphoid structures; (2) tumor cell clearance—foamy macrophages and often associated cholesterol clefts; and (3) tissue repair—neovascularization and proliferative fibrosis. irPRC recognizes these histologic features of the regression bed and scores them collectively as a constellation of colocalized features. This is readily and efficiently achieved for practicing pathologists and facilitates accurate determination of where the tumor used to be when performing %RVT calculations. If immune-mediated regression is not part of the calculation for %RVT, the potential exists to overestimate the %RVT and underestimate the therapeutic effect of a given regimen. It is possible that some individual histologic features may ultimately prove to be more predictive than the collective, and/or that tumor type, histologic grade or anatomic location (PT- or LN)-specific nuances will emerge once survival data mature and retrospective studies on individual histologic features are completed. The theoretical additive value of more detailed, elaborate or disease-specific approaches to scoring will have to be clearly superior with regard to predicting survival outcomes to outweigh the benefits of an efficient, robust and effective pan-tumor system for RVT assessment.

In addition to identifying survival surrogates in %RVT and %regression, we demonstrated that %necrosis may be negatively associated with EFS. Some reports have suggested that necrosis may represent treatment effect, although it has also been noted in untreated tumors^22,30–33^. In the current analysis, necrosis did not increase with neoadjuvant therapy or improve AUC for predicting EFS when added to %regression, in contrast to what would be expected if necrosis reflected therapeutic response. Preclinical studies have reported the adverse effect of necrosis on T cell effector function^34^, and a recent study in renal cell carcinoma showed that necrosis attenuated the survival benefit of TILs in patients receiving anti-PD-1 (ref. ^35^). Future studies should be designed to determine how to best incorporate necrosis into prognosis across therapeutic agents and contexts.

Previous scoring systems for pathologic response to chemotherapy as well as American Joint Committee on Cancer pathologic downstaging require pathologic confirmation of pretreatment nodal disease^36^, which is subject to sampling error. However, the irPRC scoring system allowed for pathologic analysis of the PT and all LNs recovered from definitive surgery, allowing us to ask outstanding questions regarding the benefit of neoadjuvant therapy in patients with and without LN involvement. Although the study was not powered to address this question, we found similar EFS improvements in patients with or without LN involvement. Importantly, when pathologic response in the PT was taken into account, patients who showed a response demonstrated a clear survival benefit, irrespective of LN involvement. Furthermore, LN assessment provided additional prognostic information beyond the PT alone, which may potentially be used to refine adjuvant therapy decisions.

Other potential predictors and surrogates of response and survival that have been considered in this setting are PD-L1 expression level, TMB and ctDNA clearance^37^. There was no clear association between the different %RVT categories evaluated and TMB or PD-L1 status. These results are largely consistent with previously reported results in the intention-to-treat population for pCR/MPR in the PD-L1 subgroups^19^. We found some association between ctDNA clearance and pCR/MPR; however, the number of patients evaluable for ctDNA was small, with only a quarter of patients evaluated. The sensitivity and specificity of ctDNA clearance for predicting pCR to neoadjuvant immunotherapy requires further development before its use as a solitary biomarker for presurgical clinical decision-making. Furthermore, the personalized ctDNA detection method used here requires whole-exome sequencing of pretreatment tumor, a relatively expensive assay requiring specialized equipment. Pathologic assessment of slides is very feasible and affordable, and can be generated using routine workflows in hospital and community laboratories around the world, without any additional equipment or individualization. In this study, every patient who went to surgery had slides made for pathologic evaluation, as is typical for hospital workflows. Neoadjuvant treatment response in breast, colon and pancreatic carcinomas, as well as many sarcomas, is assessed by pathologists routinely as a part of the College of American Pathologists/American Joint Committee on Cancer and European Society of Pathology /European Organisation for Research and Treatment of Cancer staging guidelines. Additionally, the presence of immune-mediated regression is assessed as part of melanoma staging, further indicating the ability and expectation of pathologists in performing such analyses as a part of routine care. Future studies will be required to understand the best way to leverage the potential complementarity of pathologic response, ctDNA clearance and radiologic assessment.

TRAEs, specifically immune-related adverse events, have also been associated with survival outcomes in some studies. However, survival-time bias often exists, with surviving patients receiving more immunotherapy. CheckMate 816 included optional adjuvant chemotherapy with or without radiotherapy, but not immunotherapy. As such, this study provided a unique opportunity to assess for a potential relationship between tumor regression and TRAEs in an unbiased manner, and no apparent association was observed.

Potential limitations of the study include the fact that details regarding postoperative nodal staging (information regarding anatomic levels of LNs, that is, N1 versus N2) were not available, precluding a more detailed analysis on the impact of %RVT by nodal level. Additionally, given the fact that adjuvant therapy was not part of the trial schema, it was not possible to determine whether there was a specific %RVT cutoff for which patients would benefit from the addition of an adjuvant regimen. It will also be important to assess the full spectrum of %RVT by irPRC in the lung cancer resection specimens from other registrational trials of neoadjuvant chemotherapy plus immunotherapy to validate the emerging clinically important %RVT thresholds identified herein.

The need for a standardized and shared language to report radiographic response to cancer therapy led to the development of the RECIST criteria 50 years ago. Here, we present the first prospective evidence, to our knowledge, that pathologic response can be used similarly in the neoadjuvant setting, with a standardized scoring system to assess %RVT that was specifically designed to be pan-tumor. Although this study was performed on resection specimens from patients with lung cancer, these findings have implications for response assessment and trial design in multiple tumor types. In summary, given the prognostic value of %RVT, its assessment using routine surgical pathology workflows and a scoring system generalizable to any solid tumor type, it is anticipated to become a biomarker for guiding subsequent adjuvant therapy in perisurgical clinical trials, substantiate more refined pathologic response end points and ultimately lead to a new standard of care in clinical diagnostics.

## Methods

### Patients and tissue samples

CheckMate 816 (ClinicalTrials.gov identifier: NCT02998528) is an ongoing, global, open-label, randomized phase 3 study in treatment-naive adults with resectable stage IB (≥4 cm) to IIIA non-small-cell lung cancer (per American Joint Committee on Cancer 7th edition staging criteria), Eastern Cooperative Oncology Group performance status 0 to 1 and no known sensitizing *EGFR* mutations or *ALK* alterations. From March 2017 through November 2019, a total of 773 patients were enrolled at study sites in 14 different countries. Patients were randomized 1:1 to receive nivolumab 360 mg plus platinum-doublet chemotherapy or platinum-doublet chemotherapy every 3 weeks for three cycles before undergoing definitive surgery within 6 weeks of completing neoadjuvant treatment. Randomization was stratified per interactive response technology by PD-L1 (<1%/not evaluable versus ≥1%), disease stage (IB–II versus IIIA) and sex (male versus female). The sex of participants was determined based on self-reporting to the site. Additional information on the study design and eligibility criteria of CheckMate 816 have been previously described and can be found in Supplementary Information Protocol^19^.

The path-evaluable patient population includes patients who underwent definitive surgery after neoadjuvant treatment and had pathologically evaluable samples. Within this population, analyses were conducted in patients with and without pathologic evidence of LN involvement. LN involvement refers to pathologic evidence of LN disease at resection that had fully regressed (0% RVT) or had not regressed (>0% RVT) after neoadjuvant treatment. When available, pretreatment biopsy samples were also evaluated.

#### Definitive resection specimen

PT and LN <1 cm in greatest dimension were submitted in their entirety. Tumors >1 cm and <3 cm had two small 3-mm^2^ pieces taken for additional biomarker studies, and the remainder of the specimen was submitted for histologic processing. For larger tumors (>3 cm) and LN, a full-thickness cross-section was taken from the largest dimension of the tumor mass and submitted for evaluation in addition to standard sections taken for routine staging purposes, including background normal lung. Hematoxylin-and-eosin-stained slides from all tissue blocks generated from the definitive surgical specimen (lung with or without LNs) from each case were submitted for central pathology review, along with a de-identified pathology report. For cases from China, digitized slides were submitted for review.

Pathologic assessment using irPRC was then performed by a central pathology committee consisting of a team of academic pathologists from Johns Hopkins University. Each case was read by two pathologists. If the estimate of residual tumor differed by >10% between the two pathologists, a third pathologist served as an adjudicator to finalize the score. The pathologists were blinded to treatment arm, clinical outcome and patient identifiers.

#### Pretreatment samples

When available, the original diagnostic biopsy sample was also assessed by irPRC for evidence of pre-existing immune control or clearance of tumor and/or necrosis. This approach has previously been applied to incisional pretreatment and on-treatment biopsy samples. The latter was associated with long-term patient outcomes after anti-PD-1-based therapy in the neoadjuvant and advanced disease settings^18,33^.

### Assessments

There were two primary end points, EFS according to blinded independent central review and pCR (0% RVT in the PT and sampled LN) according to blinded independent pathologic review. EFS was defined as time from randomization to progressive disease (according to RECIST 1.1) that precluded surgery, progressive disease after surgery, progressive disease in the absence of surgery or death due to any cause per blinded independent central review.

Secondary end points included MPR (≤10% RVT in the PT and sampled LNs), time to death or distant metastasis, and overall survival. Adverse events were assessed in all the treated patients. Adverse events reported here were assessed at baseline, continuously while on treatment, and within 100 days after the last dose of neoadjuvant therapy or 90 days after surgery or up to 30 days after the last dose of adjuvant therapy (whichever was longest). Grade 5 adverse events were events leading to death within 24 hours; events leading to death >24 hours after onset were reported with the worst grade before death. Clinical and biomarker assessments were performed during the course of the trial.

The timeline for sample collection for biomarker studies is shown in Fig. 1a. Each specimen was scored for pathologic response per blinded independent pathologic review using pan-tumor irPRC^7,8^. Specifically, features of %RVT, necrosis and tumor regression were determined for the tumor bed (where tumor used to be) of the PT and any involved LNs (Fig. 1b). pCR was defined as 0% and MPR as ≤10% RVT in both the PT and LNs (pCR-PT, MPR-PT, pCR-LN and MPR-LN, respectively). The association of different pathologic response categories and associated histologic features with EFS were assessed in the overall path-evaluable population and subpopulations by LN involvement. We also evaluated relationships between %RVT and radiographic response, tumor PD-L1 expression, TMB and ctDNA clearance before definitive surgery. Level of PD-L1 expression was determined using the PD-L1 IHC 28-8 pharmDx assay (Dako); patients with tumor tissue that could not be assessed for PD-L1 (≤10% of concurrently randomized patients) were stratified to the PD-L1 expression <1% subgroup at randomization. TMB was evaluated using the Illumina TSO500 assay. A 12.3-mutations per megabase (mut/Mb) cutoff per TSO500 corresponds to 10 mut/Mb per the FoundationOne assay. Analyses of ctDNA were performed with the use of a tumor-guided personalized ctDNA panel for whole-exome sequencing (ArcherDX Personalized Cancer Monitoring). Clearance of ctDNA was defined as presurgery change from detectable levels of ctDNA before cycle 1 to undetectable ctDNA before cycle 3 (ref. ^19^). ctDNA analyses were performed on plasma samples collected on day 1 before each of the three treatment cycles. TMB and ctDNA analyses were not conducted on patients from China because of local regulations. Comparisons of histologic features were made between paired pretreatment and on-treatment pathology specimens, where available.

### Statistical analysis

Approximately 350 patients were planned for concurrent randomization to nivolumab plus chemotherapy and chemotherapy. This sample size was based on the primary end points of pCR and EFS with 0.01 and 0.04 type I error allocation (two-sided), respectively. If the pCR comparison was significant, the 0.01 alpha was planned to be reallocated to the EFS comparison, which would be based on a two-sided type I error of 0.05. Additionally, overall survival was planned to be tested hierarchically after EFS significance.

AUC provides an overall diagnostic accuracy of the pathologic response parameters above; a value of 0.5 indicates random chance, 0.7 to 0.8 indicates fair predictive value, and 1 indicates perfect accuracy. The optimal cutoff value for any of the pathologic response parameters (single or combination) was calculated using Youden’s index to maximize the true positive rate and minimize the false positive rate.

Analyses are exploratory, and descriptive statistics were used to report associations. pCR, MPR and %RVT data are from the final analysis of pCR (16 September 2020), whereas all other efficacy and safety results are from the prespecified interim analysis 1 of EFS (20 October 2021; final analysis for EFS). A time-dependent ROC curve^38^ was constructed using an appropriate modeling approach to assess the predictive ability of single or combined pathologic response parameters (%RVT, and so on) for EFS at 2 years. EFS was estimated using the Kaplan–Meier method, with HRs and associated two-sided 95% confidence intervals calculated using an unstratified Cox proportional hazards model.

Bristol Myers Squibb’s Trial Access online eWR number 8091 dated 4 February 2022 along with eDM/Oracle Clinical Release number 5.4.012r7 dated 10 April 2023 was used for data collection. SAS Studio v.9.04.01M7P08062020 (AWS) was used for data analysis.

### Trial oversight

The sponsor (Bristol Myers Squibb) analyzed the data with participation from all authors. CheckMate 816 was performed in accordance with the Declaration of Helsinki and the International Conference on Harmonisation Good Clinical Practice guidelines. The extended pathologic analysis of resection specimens reported herein was conducted at Johns Hopkins University and was approved by the Johns Hopkins University institutional review board (IRB00122321). The study protocol for the parent trial and all amendments were approved by an institutional review board or independent ethics committee at each study site, and an independent data and safety monitoring committee reviewed/monitored the efficacy and safety of all evaluated treatments. A list of investigators and study sites was previously published^19^. All patients provided written informed consent before initiating study procedures. No compensation was provided to the participants except for a few study sites who provided travel costs, as necessary.

### Reporting summary

Further information on research design is available in the Nature Portfolio Reporting Summary linked to this article.

## Online content

Any methods, additional references, Nature Portfolio reporting summaries, source data, extended data, supplementary information, acknowledgements, peer review information; details of author contributions and competing interests; and statements of data and code availability are available at 10.1038/s41591-023-02660-6.

### Supplementary information

Supplementary Information Supplementary Tables 1–3 and Clinical Trial Protocol.

Reporting Summary

## Data Availability

De-identified and anonymized data will be made available within a secured portal to qualified researchers who submit an in-scope proposal approved by the Independent Review Committee. Proposals will be reviewed to ensure that there is adequate scientific rationale and methodology, a robust statistical analysis plan and a publication plan. Researchers should have relevant experience and demonstrate a plan to address any conflicts of interest, if applicable. Requests will be reviewed and processed by an independent committee; consequently, Bristol Myers Squibb cannot provide an estimated response time. For more information and to submit a data-sharing request, please visit https://www.bms.com/researchers-and-partners/independent-research/data-sharing-request-process.html.
