# Supplementary material for: Association between pathologic response and survival after neoadjuvant therapy in lung cancer
Source: Nat Med. 2023 Oct 30;30(1):218–28. doi: 10.1038/s41591-023-02660-6 (PMC10803255; doi:10.1038/s41591-023-02660-6)
Supplement: Supplementary file 1 — Supplementary Tables 1–3 and Clinical Trial Protocol. [file 41591_2023_2660_MOESM1_ESM.pdf]

nature medicine

Article https://doi.org/10.1038/s41591-023-02660-6

Association between pathologic response and survival after neoadjuvant therapy in lung cancer

In the format provided by the authors and unedited

Supplementary Table 1 │ Adjuvant therapy in path-evaluable patients and patients with or without pathologic evidence of LN involvement

	Path-evaluable population					
	All		With LN involvement		Without LN involvement	
	Nivolumab plus chemotherapy (*n* = 141)	Chemotherapy (*n* = 126)	Nivolumab plus chemotherapy (*n* = 68)	Chemotherapy (*n* = 74)	Nivolumab plus chemotherapy (*n* = 72)	Chemotherapy (*n* = 51)
**No adjuvant treatment**	75.9	57.1	67.6	50.0	83.3	66.7
%RVT-PT						
Mean (SD)	24.79 (34.01)	53.28 (36.64)	31.35 (35.07)	55.47 (37.01)	20.17 (32.78)	51.29 (37.13)
Median (IQR)	2.00 (40.00)	60.50 (81.50)	17.00 (70.00)	65.00 (71.00)	1.00 (30.00)	58.00 (83.00)
**All adjuvant treatment**	24.1	42.9	32.4	50.0	16.7	33.3
Chemotherapy	14.2	30.2	16.2	32.4	12.5	27.5
Radiotherapy	6.4	9.5	11.8	13.5	1.4	3.9
Chemotherapy + radiotherapy	3.5	3.2	4.4	4.1	2.8	2.0
%RVT-PT						
Mean (SD)	64.52 (39.07)	74.49 (26.31)	70.75 (36.45)	78.66 (21.31)	53.11 (42.70)	65.41 (33.80)
Median (IQR)	82.50 (78.00)	85.00 (35.00)	85.25 (44.00)	85.00 (30.00)	65.50 (84.15)	73.00 (42.00)

Data reported as % unless otherwise noted.
Per investigator discretion after surgery, all patients could receive up to 4 cycles of adjuvant chemotherapy and/or radiotherapy.
IQR denotes interquartile range; LN; lymph node; PT, primary tumor; RVT, residual viable tumor; SD, standard deviation.

Supplementary Table 2 │ PD-L1 and TMB by %RVT in PT

	PD-L1				TMB		
**Patients, *n* (%)**	**<1%**	**1–49%**	**≥50%**	**NE**	**<12.3 mut/Mb**	**≥12.3 mut/Mb**	**NR/NE**
**Nivolumab plus chemotherapy (*n* = 141)**	71 (50.4)	32 (22.7)	27 (19.1)	11 (7.8)	41 (29.1)	29 (20.6)	71 (50.4)
0% RVT-PT (pCR; *n* = 46)	15 (10.6)	12 (8.5)	18 (12.8)	1 (0.7)	12 (8.5)	13 (9.2)	21 (14.9)
0%–10% RVT-PT (MPR; *n* = 72)	28 (19.9)	19 (13.5)	21 (14.9)	4 (2.8)	16 (11.3)	19 (13.5)	37 (26.2)
11%–30% RVT-PT (*n* = 15)	9 (6.4)	2 (1.4)	1 (0.7)	3 (2.1)	4 (2.8)	3 (2.1)	8 (5.7)
31%–50% RVT-PT (*n* = 6)	4 (2.8)	1 (0.7)	0	1 (0.7)	1 (0.7)	1 (0.7)	4 (2.8)
51%–80% RVT-PT (*n* = 19)	11 (7.8)	5 (3.5)	2 (1.4)	1 (0.7)	8 (5.7)	3 (2.1)	8 (5.7)
81%–100% RVT-PT (*n* = 29)	19 (13.5)	5 (3.5)	3 (2.1)	2 (1.4)	12 (8.5)	3 (2.1)	14 (9.9)
**Chemotherapy (*n* = 126)**	61 (48.4)	33 (26.2)	22 (17.5)	10 (7.9)	37 (29.4)	29 (23.0)	60 (47.6)
0% RVT-PT (pCR; *n* = 5)	3 (2.4)	0	2 (1.6)	0	2 (1.6)	1 (0.8)	2 (1.6)
0%–10% RVT-PT (MPR; *n* = 22)	17 (13.5)	2 (1.6)	3 (2.4)	0	9 (7.1)	5 (4.0)	8 (6.3)
11%–30% RVT-PT (*n* = 6)	2 (1.6)	1 (0.8)	3 (2.4)	0	2 (1.6)	2 (1.6)	2 (1.6)
31%–50% RVT-PT (*n* = 17)	5 (4.0)	6 (4.8)	4 (3.2)	2 (1.6)	2 (1.6)	7 (5.6)	8 (6.3)
51%–80% RVT-PT (*n* = 25)	14 (11.1)	8 (6.3)	3 (2.4)	0	7 (5.6)	4 (3.2)	14 (11.1)
81%–100% RVT-PT (*n* = 56)	23 (18.3)	16 (12.7)	9 (7.1)	8 (6.3)	17 (13.5)	11 (8.7)	28 (22.2)

Percents are calculated out of the total population for each treatment arm.
MPR denotes major pathologic response; mut/Mb, mutations/megabase; NE, not evaluable; NR, not reported; pCR, pathologic complete response; PD-L1, programmed death ligand 1; RVT, residual viable tumor; TMB, tumor mutational burden.

Supplementary Table 3 │ Safety in the path-evaluable patient population

	All treated		pCR-PT[b]		No pCR-PT[b]		MPR-PT[b]		No MPR-PT[b]	
**TRAEs**	**Nivolumab plus chemo (*n* = 176)**	**Chemo (*n* = 176)**	**Nivolumab plus chemo (*n* = 46)**	**Chemo (*n* = 5)**	**Nivolumab plus chemo (*n* = 95)**	**Chemo (*n* = 121)**	**Nivolumab plus chemo (*n* = 72)**	**Chemo (*n* = 22)**	**Nivolumab plus chemo (n = 69)**	**Chemo (*n* = 104)**
**Any grade[a]**	82	89	85	100	84	88	79	96	90	86
**Grade 3–4[a]**	34	37	30	40	37	35	31	32	39	36

Values shown are percent of patients.

Chemo denotes chemotherapy; MPR, major pathologic response; pCR, pathologic complete response; PT, primary tumor; TRAE, treatment-related adverse event.

[a]Includes events reported between the first neoadjuvant dose and 30 days after the last neoadjuvant dose as per Common Terminology Criteria for Adverse Events Version 4.0; Medical Dictionary for Regulatory Activities Version 24.0.

[b]pCR and MPR were assessed in the path-evaluable patient population.

Protocol

This document contains the following items related to CheckMate 816:

1. Original protocol (page 2), final protocol (page 129), summary of changes (document history, page 130).
2. Original statistical analysis plan (page 300), final statistical analysis plan (page 382), and summary of changes (document history, page 457).

Clinical Protocol CA209816

Randomized, Open-Label, Phase 3 Trial of Nivolumab and Ipilimumab versus Platinum-Doublet Chemotherapy in Early Stage NSCLC

(CheckMate 816: CHECKpoint pathway and nivoluMAb clinical Trial Evaluation 816)

[redacted]

24-hr Emergency Telephone Number

[redacted]

Bristol-Myers Squibb Research and Development
Route 206 & Province Line Road
Lawrenceville, NJ 08543

This document is the confidential and proprietary information of Bristol-Myers Squibb Company and its global affiliates (BMS). By reviewing this document, you agree to keep it confidential and to use and disclose it solely for the purpose of assessing whether your organization will participate in and/or the performance of the proposed BMS sponsored study. Any permitted disclosures will be made only on a confidential "need to know" basis within your organization or to your independent ethics committee(s). Any other use, copying, disclosure or dissemination of this information is strictly prohibited unless expressly authorized in writing by BMS. Any supplemental information (e.g., amendments) that may be added to this document is also confidential and proprietary to BMS and must be kept in confidence in the same manner as the contents of this document. Any person who receives this document without due authorization from BMS is requested to return it to BMS or promptly destroy it. All other rights reserved. References to BMS in this

protocol may apply to partners to which BMS has transferred obligations, e.g., a Contract Research Organization (CRO).

DOCUMENT HISTORY

Document	Date of Issue	Summary of Change
Original Protocol	30-Sep-2016	Not Applicable

Approved v1.0 [redacted]

TABLE OF CONTENTS

TITLE PAGE 1
DOCUMENT HISTORY 3
TABLE OF CONTENTS 4
1. SYNOPSIS 7
2. SCHEDULE OF ACTIVITIES 13
3. INTRODUCTION 25
3.1 Study Rationale 25
3.1.1 Research Hypothesis 27
3.2 Background 27
3.2.1 Indication Background 27
3.2.2 Nivolumab Mechanism of Action 28
3.2.3 Ipilimumab Mechanism of Action 29
3.2.4 Nivolumab Combined with Ipilimumab 29
3.3 Benefit/Risk Assessment 30
4. OBJECTIVES AND ENDPOINTS 31
5. STUDY DESIGN 32
5.1 Overall Design 32
5.1.1 Data Monitoring Committee and Other External Committees 36
5.2 Number of Participants 37
5.3 End of Study Definition 37
5.4 Scientific Rationale for Study Design 37
5.4.1 Rationale for Open-Label Design 37
5.4.2 Rationale for Preoperative Systemic Therapy in NSCLC 38
5.4.3 Rationale for Nivolumab and Ipilimumab for Neoadjuvant NSCLC 38
5.4.4 Rationale for Combination of Nivolumab and Ipilimumab (Arm A): 39
5.4.5 Rationale for Major Pathological Response Endpoint 42
5.4.6 Rationale for Main Inclusion and Exclusion Criteria: 42
5.4.7 Rationale for Choices of Platinum-based Chemotherapy Doublet 43
5.5 Justification for Dose 43
5.5.1 Rationale for Shorter Infusion Times for Nivolumab 43
6. STUDY POPULATION 43
6.1 Inclusion Criteria 43
6.2 Exclusion Criteria 45
6.3 Lifestyle Restrictions 47
6.4 Screen Failures 47
6.4.1 Retesting During Screening or Lead-In Period 47
7. TREATMENT 47
7.1 Treatments Administered 50
7.2 Method of Treatment Assignment 54
7.3 Blinding 55
7.4 Dosage Modification 55
7.4.1 Dose Reductions for Platinum Doublet Chemotherapy 55
7.4.1.1 Platinum Doublet Chemotherapy - Dose Reductions for Hematologic Toxicity 55

Approved v1.0 [redacted]

7.4.1.2 Platinum Doublet Chemotherapy - Dose Reductions for Non-Hematologic Toxicities 58
7.4.2 Dose Delay 59
7.4.2.1 Nivolumab Dose Delay Criteria 59
7.4.2.2 Dose Delay Criteria for Platinum Doublet Chemotherapy 60
7.4.2.3 Docetaxel Dose Delay Criteria 61
7.4.3 Treatment of Nivolumab- and Ipilimumab-related Infusion Reactions ... 61
7.5 Preparation/Handling/Storage/Accountability 62
7.5.1 Retained Samples for Bioavailability / Bioequivalence 63
7.6 Treatment Compliance 63
7.7 Concomitant Therapy 63
7.7.1 Prohibited and/or Restricted Treatments 63
7.7.2 Other Restrictions and Precautions 63
7.7.3 Permitted Therapy 64
7.8 Treatment After the End of the Study 64
8. DISCONTINUATION CRITERIA 64
8.1 Discontinuation from Study Treatment 64
8.1.1 Study Treatment Discontinuation Criteria 65
8.1.1.1 Nivolumab Discontinuation Criteria 65
8.1.1.2 Platinum Doublet Chemotherapy Dose Discontinuation 66
8.1.1.3 Docetaxel Dose Discontinuation 67
8.1.2 Criteria to Resume Treatment 68
8.1.2.1 Criteria to Resume Nivolumab Treatment 68
8.1.2.2 Criteria to Resume Platinum Doublet Chemotherapy Dosing 69
8.1.2.3 Criteria to Resume Treatment with Docetaxel 69
8.1.3 Post Study Treatment Study Follow-up 69
8.2 Discontinuation from the Study 70
8.3 Lost to Follow-Up 70
9. STUDY ASSESSMENTS AND PROCEDURES 70
9.1 Efficacy Assessments 71
9.1.1 Imaging Assessment for the Study 72
9.1.2 Patient-reported Outcomes 72
9.2 Adverse Events 73
9.2.1 Time Period and Frequency for Collecting AE and SAE Information 73
9.2.2 Method of Detecting AEs and SAEs 74
9.2.3 Follow-up of AEs and SAEs 74
9.2.4 Regulatory Reporting Requirements for SAEs 74
9.2.5 Pregnancy 75
9.2.6 Laboratory Test Result Abnormalities 75
9.2.7 Potential Drug Induced Liver Injury (DILI) 76
9.2.8 Other Safety Considerations 76
9.2.9 Management Algorithms 77
9.3 Overdose 78
9.4 Safety 78
9.4.1 Clinical Safety Laboratory Assessments 79
9.4.2 Imaging Safety Assessment 79

Approved v1.0 [redacted]

9.5 Pharmacokinetics 79
9.6 Pharmacodynamics 80
9.7 Pharmacogenomics 80
9.8 Biomarkers 80
9.8.1 Additional Research Collection 80
9.8.1.1 Tumor Tissue Specimens 82
[redacted]
9.8.2 Safety Analyses 85
9.9 Health Economics OR Medical Resource Utilization and Health Economics . 85
9.10 Other Assessments 85
9.10.1 Diagnostic/Pre-surgical Evaluation and Surgical Guidelines: 85
9.10.2 Post-surgical Evaluation: 86
10. STATISTICAL CONSIDERATIONS 86
10.1 Sample Size Determination 86
10.2 Populations for Analyses 87
10.3 Statistical Analyses 87
10.3.1 Efficacy Analyses 87
10.3.2 Other Analyses 88
10.3.3 Interim Analyses 88
11. REFERENCES 90
12. APPENDICES 94
APPENDIX 1 TNM STAGING SYSTEM FOR LUNG CANCER (7TH EDITION) 94
APPENDIX 2 ABBREVIATIONS AND TRADEMARKS 95
APPENDIX 3 ECOG PERFORMANCE STATUS 98
APPENDIX 4 MANAGEMENT ALGORITHMS 99
APPENDIX 5 RESPONSE CRITERIA (RECIST 1.1) 107
APPENDIX 6 WOMEN OF CHILDBEARING POTENTIAL DEFINITIONS AND METHODS OF CONTRACEPTION 115
APPENDIX 7 STUDY GOVERANCE CONSIDERATIONS 118
APPENDIX 8 ADVERSE EVENTS AND SERIOUS ADVERSE EVENTS: DEFINITIONS AND PROCEDURES FOR RECORDING, EVALUATING, FOLLOW UP AND REPORTING 125

Approved v1.0 [redacted]

1. SYNOPSIS

Protocol Title: Randomized, Open-Label Phase 3 Trial of Nivolumab and Ipilimumab versus Platinum-Doublet Chemotherapy in Early Stage NSCLC

Study Phase: 3

Rationale:

Approximately 80% of lung cancer cases are non-small cell lung cancer (NSCLC), with most patients presenting with late-stage disease. At initial diagnosis, 20% of patients present with stage I or II disease, whereas 30% present with stage III disease and 50% with stage IV disease. With enhanced lung cancer screening techniques, the percentage of patients diagnosed during the early stages may increase over the duration of the trial. A standard TNM staging system is used to determine the staging for NSCLC (Appendix 1). Patients with pathologic stage I NSCLC have a 5-year survival of approximately 60%. Stage II to III NSCLC patients have a 5-year survival of approximately 25% to 40%.[1] Surgical resection remains the mainstay of treatment for stage I and II patients; however, despite potentially curative surgery, approximately 50% of stage IB and 60-75% of stage II NSCLC patients will relapse and eventually die of their disease.[2,3] A rational approach to improve survival in these patients is to eradicate micrometastatic disease and to minimize the risk of relapse after adjuvant or neoadjuvant chemotherapy.

This Phase 3 study, CA209816, will evaluate the clinical efficacy of nivolumab and ipilimumab in operable lung cancer. Specifically, this study will compare the major pathological response (MPR) rate among participants with Stage IB-IIIa NSCLC treated with nivolumab and ipilimumab to the MPR rate in participants treated with platinum doublet chemotherapy.

Study Population:

Key Inclusion Criteria

a) Eastern Cooperative Group (ECOG) Performance Status 0-1
b) Participants with histologically confirmed Stage IB (≥ 4 cm), II, IIIA (N2) NSCLC (per the 8th American Joint Committee on Cancer - the AJCC) who are considered resectable[4]
c) Measurable disease according to Response Evaluation Criteria in Solid Tumors version 1.1 (RECIST 1.1)
d) Participants must have tumor tissue available for PD-L1 immunohistochemical (IHC) testing performed by a third-party analyzing lab during the screening period:
    i) Either a formalin-fixed, paraffin-embedded (FFPE) tissue block or unstained tumor tissue sections, with an associated pathology report, must be submitted for biomarker evaluation prior to randomization. The tumor tissue sample may be fresh or archival if obtained within 6 months prior to enrollment.
    ii) Tissue must be a core needle biopsy, excisional or incisional biopsy. Fine needle biopsies obtained by EBUS is not considered adequate for biomarker review and randomization. Core needle biopsies obtained by EBUS are acceptable for randomization.

Approved v1.0 [redacted]

Key Exclusion Criteria:

a) Presence of locally advanced, unresectable or metastatic disease . Mediastinal lymph node samples at levels 4 (bilaterally) and 7 are required for clinical staging to assess nodal involvement in participants with mediastinal adenopathy on PET/CT.
b) Participants with known EGFR mutations or ALK translocation. If testing is done, testing will be done using an FDA-approved assay and will be performed locally.

Objectives and Endpoints:

Objective	Endpoint
**Primary**	
• To compare the MPR rate by blinded independent pathology review (BIPR) of participants receiving nivolumab and ipilimumab to that of participants receiving platinum doublet chemotherapy in operable stage IB (≥ 4 cm), II, or resectable IIIA (N2) PD-L1+ (≥ 1%) NSCLC	• MPR rate, defined as number of randomized participants with <10% residual tumor in lung and lymph nodes as evaluated by BIPR, divided by the number of randomized participants for each treatment group.
**Secondary**	
• To compare the event-free survival (EFS) by blinded independent central review (BICR) in participants receiving nivolumab and ipilimumab to participants receiving platinum doublet chemotherapy in operable stage IB (≥ 4 cm), II, or resectable IIIA (N2) PD-L1+ (≥ 1%) NSCLC<br>• To assess the OS of participants receiving nivolumab and ipilimumab to that of participants receiving platinum doublet chemotherapy in operable stage IB (≥ 4 cm), II, or resectable IIIA (N2) PD-L1+ (≥ 1%) NSCLC<br>• To assess complete pathological response (pCR) by BIPR of participants receiving nivolumab and ipilimumab compared to participants receiving platinum doublet chemotherapy in operable stage IB (≥ 4 cm), II, or resectable IIIA (N2) PD-L1+ (≥ 1%) NSCLC	• EFS defined as the length of time from randomization to any of the following events: progression of disease, recurrence disease, or death due to any cause. Progression/recurrence will be assessed by BICR per RECIST 1.1.<br>• OS is defined as the time between the date of randomization and the date of death. OS will be censored on the last date a participants was known to be alive.<br>• pCR is defined as the absence of residual tumor in lung and lymph nodes as evaluated by BIPR

Overall Design:

This is an open-label, randomized clinical trial of up to 3 cycles of neoadjuvant nivolumab (3 mg/kg every 2 weeks) and one 1 mg/kg dose of ipilimumab vs neoadjuvant platinum doublet chemotherapy (up to 3 cycles) in participants with early stage (Stage IB [≥ 4 cm], II, and resectable IIIA [N2]) NSCLC.

Eligible participants will be randomized between 2 arms in a 1:1 ratio. Participants will be stratified by:

- PD-L1 expression (≥1% or <1%/not evaluable/indeterminate)
- Disease stage (IB/II vs IIIA)
- Gender

PD-L1 status will be determined by immunohistochemical (IHC) staining of PD-L1 protein in the submitted tumor sample and categorized as follows:

- PD-L1 positive - defined as ≥ 1% tumor cell membrane staining positive in a minimum of 100 evaluable tumor cells
- PD-L1 negative – defined as < 1% tumor cell membrane staining positive in a minimum of 100 evaluable tumor cells
- PD-L1 not evaluable/indeterminate - defined as participants with insufficient sample quantity or quality to stain for PD-L1 status or those in whom PD-L1 status could not be determined despite appropriate amounts of tissue sample. For this category, key efficacy and safety parameters will be summarized and grouped with PD-L1 negative participants. No more than 10% of participants will be randomized into this category.

Tumor tissue (archival [blocks/slides ≤ 6 months old] or recent tumor biopsy) must be submitted to a third-party vendor for determination of PD-L1 status testing prior to randomization.

PET/CT including IV contrast (CT of diagnostic quality) will be performed at baseline and within 7 days prior to planned surgery. Subsequent assessments (CT or MRI) will be performed in accordance with the Schedule of Activities. Tumor assessments must continue per protocol until disease recurrence/progression is confirmed by BICR per RECIST 1.1. Details will be outlined in the radiology manual. OS will be followed continuously every 3 months via in-person or phone contact after Post-neoadjuvant Follow-up Visit 2.

Following the completion of treatment in Arm A or Arm B, all participants who remain operative candidates will undergo definitive surgery for NSCLC. Surgery should be performed within 6 weeks after completing nivolumab or chemotherapy treatment.

Following definitive surgery, participants in each arm may receive up to 4 cycles of adjuvant chemotherapy with or without radiation per institutional standard at the discretion of the investigator. Investigators may choose from the same chemotherapy regimens used in the neoadjuvant phase of the study.

Number of Participants:

Approximately 326 participants will be randomized to 2 arms in a 1:1 ratio; therefore, approximately 163 participants will be included in each arm.

Approved v1.0 [redacted]

Treatment Arms and Duration:

Participants randomized into Arm A will receive nivolumab 3 mg/kg IV over 30 minutes every 2 weeks for up to 3 doses (ie, 6 weeks of treatment; each cycle is 14 days). In Cycle 1 Day 1 only, nivolumab will be followed by a single dose of ipilimumab 1 mg/kg over 30 minutes.

Participants randomized to Arm B will receive 1 of the following investigator-choice platinum doublet chemotherapy regimens in 3-week cycles up to a maximum of 3 cycles of IV chemotherapy (ie, 9 weeks of treatment; each cycle is 21 days):

- Regimen 1:
  - Vinorelbine 30 mg/m^2 IV push over 10 minutes, Days 1 and 8
  - Cisplatin 75 mg/m^2 IV over 120 minutes Day 1, immediately following vinorelbine
- Regimen 2:
  - Docetaxel 75 mg/m^2 IV over 60 minutes on Day 1
  - Cisplatin 75 mg/m^2 IV over 120 minutes on Day 1, immediately following docetaxel
- Regimen 3 (squamous histology):
  - Gemcitabine 1250 mg/m^2 IV over 30 minutes on Days 1 and 8
  - Cisplatin 75 mg/m^2 IV over 120 minutes on Day 1, immediately following gemcitabine
- Regimen 4 (non-squamous histology only):
  - Pemetrexed 500 mg/m^2 IV are over 10 minutes on Day 1
  - Cisplatin 75 mg/m^2 IV over 120 minutes on Day 1, immediately following pemetrexed

Study treatment:

Study Drug for CA209816		
**Medication**	**Potency**	**IP/Non-IP**
BMS-936558-01 Nivolumab	10 mg/mL	IP
Ipilimumab Solution for Injection	5 mg/mL	IP
Vinorelbine	10 mg/mL	IP
Gemcitabine	38 mg/mL	IP
Docetaxel	10 mg/mL	IP
Pemetrexed	500 mg/vial	IP
Cisplatin	1 mg/mL	IP
Carboplatin	10 mg/mL	IP

Approved v1.0 [redacted]

Independent Data Monitoring Committee

An independent Data Monitoring Committee (DMC) will be established to provide oversight of safety and efficacy considerations, study conduct, and risk-benefit ratio in protocol CA209816. An interim DMC review will occur after 15 participants are enrolled in each arm and have completed surgery. Following review, the DMC will recommend continuation, modification, or discontinuation of this study based on reported safety data. Details of DMC responsibilities and procedures will be specified in the DMC charter. Representatives of the Sponsor will serve only as coordinators of the committee, without having full member responsibilities or privileges. In addition, the Sponsor will independently review safety data in a blinded manner during the conduct of this trial to ensure that any safety issues are identified and addressed.

In addition, efficacy will also be reviewed by the DMC for the formal interim analysis of event-free survival (EFS).

Independent Radiology/Pathology Review:

Independent pathology and radiology review will be established for central review and confirmation of responses. Images and tumor/lymph node samples will be submitted to these third-party vendors for central review. Sites will be trained prior to enrolling the first study participant. Images and pathology samples acquisition guidelines and submission process will be outlined in the study Imaging/Pathology Manuals to be provided by the vendors. For histologic assessment, all tumor and associated lymph node tissue should be sectioned at 1 cm intervals. For assessments of pathological response, the percentage of viable tumor cells in at least 1 section per centimeter of the tumor and lymph node tissue resected should be evaluated.

Radiologic tumor assessments should be submitted to the third-party radiology vendor as they are performed on an ongoing basis.

Tumor assessments will be sent to and reviewed by a Blinded Independent Central Review (BICR) from a third-party radiology vendor on an on-going basis. At the time of investigator-assessed radiographic progression per RECIST 1.1, the site must request a BICR for confirmation of progression or disease recurrence. Details of the BICR responsibilities and procedures will be specified in the BICR charter.

Participants whose disease progression or disease recurrence is not confirmed by central review will be required to continue tumor assessments (if clinically feasible) according to the protocol-specified schedule. Subsequent tumor assessments must be submitted to the third party radiology vendor for subsequent review and may be discontinued when the investigator and independent radiologists both assess the participant to have met RECIST 1.1 criteria for progression or disease recurrence.

Abbreviations are listed in Appendix 2.

Approved v1.0 [redacted]

REFERENCES

1 Mountain, CA. Regional lymph node classification for lung cancer staging. Chest. 1997: 1718-23.

2 Goldstraw P, Crowley J, Chansky K, et al for the International Association for the Study of Lung Cancer Internatonal Staging Committee. The IASLC Lung Cancer Staging Project: proposals for the revision of the TNM stage groupings in the forthcoming (seventh) edition of the TNM Classification of malignant tumours. J Thorac Onco. 2007; 2(8):706-14.

3 Detterbeck FC, Boffa DJ, Tanoue LT. The new lung cancer staging system. Chest. 2009; 136: 260-71.

4 Rami-Porta, R. Bolejack, V, Crowley, J. et al. The ISLAC Lung Cancer Staging Project: Proposals for the Revisions of the Descriptors in the Forthcoming Eith Edition of the TNM Classification for Lung Cancer. J Thorac Onco. 2015;Jul; 10(7):990-1003.

2. SCHEDULE OF ACTIVITIES

Table 2.-1: Screening Procedural Outline (CA209816[a])

Procedure	Screening Visit	Notes
**Eligibility Assessments**		
Informed Consent	X	Informed Consent Form prior to screening for study participation. If a participant is re-enrolled, the participant must be re-consented, and eligibility should be re-confirmed.
Inclusion/Exclusion Criteria	X	All inclusion/exclusion criteria should be assessed at screening and confirmed prior to first dose. Participants must also meet operative criteria (Section 9.10.1)
Medical History	X	
Tumor Tissue Samples	X	Tumor biopsy prior to randomization is mandatory. If a recent/archived (within 6 months) biopsy sample is not available at screening, a fresh biopsy will be taken at any point prior to randomization.<br>Sufficient tumor tissue obtained prior to randomization (block or minimum of 10 slides, obtained from core biopsy, punch biopsy, excisional biopsy or surgical specimen). If less than 10 slides are available, then the Medical Monitor should be contracted to advise on eligibility.<br>For participants for whom a biopsy is not feasible, archival tumor material must be made available.<br>Fine needle aspirate of draining lymph node is not acceptable. Core needle biopsies obtained by EBUS are acceptable.<br>PD-L1 status must be determined prior to randomization.
Mediastinal lymph node sampling	X	Mediastinal lymph node samples at levels 4 (bilaterally) and 7 are required for clinical staging prior to study therapy to assess nodal involvement in participants with mediastinal adenopathy on PET/CT. Mediastinoscopy, thoracotomy, or EBUS are all acceptable for such assessment.
Screening/Baseline Tumor Assessments	X	PET/CT including IV contrast (CT of diagnostic quality) from the base of the skull to the upper thighs, within 28 days prior to randomization.<br>Participants with stage II or higher disease and those with a suspicion of brain metastasis should have a MRI or CT of the brain with contrast.<br>Tumor assessments should be performed following RECIST 1.1 criteria.
Prior Medication	X	
ECOG Performance Status	X	Within 14 days prior to randomization (Appendix 3)

Table 2.-1: Screening Procedural Outline (CA209816[a])

Procedure	Screening Visit	Notes
**Safety Assessments**		
History and Physical Examination	X	Includes assessment of symptoms, review of system (ROS), height, weight, BSA (for platinum dosing), and full physical exam within 14 days prior to randomization.
Vital Signs and Oxygen Saturation	X	Includes body temperature, respiratory rate, and seated blood pressure, heart rate, and oxygen saturation by pulse oximetry (at rest). Blood pressure and heart rate should be measured after the participant has been resting quietly for at least 5 minutes. To be assessed at the start of the Screening process. Consider alternate position(s) for vital sign collection.
Pulmonary Function Test	X	Within 28 days prior to randomization.
Concomitant Medication Collection	X	Within 14 days of randomization.
Serious Adverse Events Assessment	X	
Adverse Events Assessment	X	
Laboratory Tests	X	CBC w/differential, chemistry panel including: Albumin, LDH, AST, ALT, ALP, T. Bili, BUN or serum urea level, creatinine, Ca, Mg, Na, K, Cl, glucose, amylase, lipase, TSH, Free T4, Free T3 within 14 days prior to randomization.<br>Hepatitis B surface antigen (HBV s Ag), and hepatitis C antibody (HCV Ab) or Hepatitis C RNA (HCV RNA) within 28 days prior to randomization.
ECG	X	ECG should be done after the participants meets all eligibility criteria and should be recorded after the participant has been supine for at least 5 minutes.
Pregnancy Test	X	Urine or serum test within 14 days prior to randomization.
**Study Treatment**		
Randomize	X	

a Screening assessments should occur within 28 days of randomization unless otherwise noted.

ALT, alanine aminotransferase; AST, aspartate aminotransferase; BUN, blood urea nitrogen; Ca, calcium; CBC complete blood count; Cl, chloride; EBUS, endobronchial ultrasound; ECG, electrocardiogram; HBV s Ag, Hepatitis B surface antigen; HCV Ab, Hepatitis C antibody; ICF, informed consent form;

Approved v1.0 [redacted]

LDH, lactate dehydrogenase, K, potassium; Mg, magnesium; NA, sodium; RECIST, Response Evaluation Criteria in Solid Tumors; SOC, standard of care; T.Bili, total bilirubin; TSH, thyroid stimulating hormone.

Table 2.-2: Neoadjuvant Period Procedural Outline (CA209816)

Procedure	During Treatment Visits[a] for Arms A and B (± 3 days)	Notes
**Safety Assessments**		
Physical Examination	X	Includes assessment of signs and symptoms and ROS.
Vital Signs and Oxygen Saturation	X	Including BP, HR, temperature, respiratory rate, and oxygen saturation by pulse oximetry.
Physical Measurements	X	Weight, Body Surface Area (for cytotoxic dosing), and ECOG status. The dosing calculations for treatment arms should be based on the body weight. If the participant's weight on the day of dosing differs by > 10% from the weight used to calculate the dose, the dose must be recalculated for the dose of the day by using the participant'ss weight of the day, and this updated value becomes the reference for subsequent dosing calculations. All doses should be rounded to the nearest milligram.
Serious Adverse Event Assessment	X	
Adverse Events Assessment	X	
Review of Concomitant Medications	X	
Pulmonary Function Test	X	PFTs should be re-evaluated prior to surgery.
Laboratory test	X	On-study local laboratory assessments should be done within 72 hours prior to each dose, starting with Cycle 1 Day 1 (C1D1).<br>CBC w/differential, LFTs (ALT, AST, total bilirubin, alkaline phosphatase), BUN or serum urea level, creatinine, Ca, Mg, Na, K, Cl, LDH, glucose, amylase, lipase.<br>TSH with reflexive Free T4, Free T3 for nivolumab arm only. Repeat every 6 weeks while receiving nivolumab.
Pregnancy Test	X	Serum or urine within 24 hours prior to first dose and then every 4 weeks (± 1 week) while receiving nivolumab and per standard prescribing guidelines for platinum doublet chemotherapy.

Table 2.-2: Neoadjuvant Period Procedural Outline (CA209816)

Procedure	During Treatment Visits[a] for Arms A and B (± 3 days)	Notes
**Efficacy Assessments**		
Tumor Assessments	X	PET/CT including IV contrast (CT of diagnostic quality) of the base of the skull through the upper thighs, within 7 days prior to surgery per RECIST 1.1.
**Pharmacokinetic Assessments**		
PK samples[b]	X	Refer to Table 9.5-1
**Biomarker Assessments**		
Exploratory Biomarker	X	[redacted]
**Patient-reported Outcomes Assessment**		
EQ-5D-3L	X	For C1D1: performed after randomization PRIOR to first dose.<br>On the day of each study drug administration, the EQ-5D-3L will be administered PRIOR to treatment.
**Study Treatment**		
IRT Drug Vial Assignments	X	

Table 2.-2: Neoadjuvant Period Procedural Outline (CA209816)

Procedure	During Treatment Visits[a] for Arms A and B (± 3 days)	Notes
Dispense Study treatment	X	Within 7 calendar days after randomization, the participants must receive the first dose of study medication. Participants may be dosed no less than 12 days between doses on the nivolumab arm.<br>If a dose is delayed for any reason, participants should be dosed no later than 7 days following a planned dose on any arm.<br>If more than 7 days delay is needed for any reason, the intended dose should be skipped. If a participant receiving chemotherapy on a Day 1 and Day 8 schedule (ie, cisplatin/gemcitabine) is unable to receive Day 1 of chemotherapy but recovers in time to receive the Day 8 dose, the Day 8 dose of chemotherapy may be administered.
Surgery per Standard of Care	See notes	Surgery should be performed within 6 weeks after completing up to 3 cycles of nivolumab or chemotherapy as indicated by the institutional standard of care (SOC).<br>Doses skipped during pre-operative treatment should not be replaced.

[a] Each cycle in Arm A is 14 days; each cycle in Arm B is 21 days.

[b] Arm A only

ALT, alanine aminotransferase; AST, aspartate aminotransferase; BP, blood pressure; BUN, blood urea nitrogen; Ca, calcium; CBC complete blood count; Cl, chloride; HR, heart rate; ECOG, Eastern Cooperative Oncology Group; IRT, interactive response technology; LDH, lactate dehydrogenase, K, potassium; Mg, magnesium; NA, sodium; PFT, pulmonary function test; ROS, review of systems; T.Bili, total bilirubin; TSH, thyroid stimulating hormone.

.

Table 2.-3: Post-Neoadjuvant Period for Those Participants Not Receiving Adjuvant Therapy (CA209816)

Procedure	Post-Neoadjuvant Therapy Visits 1 and 2[a]	Survival Follow-Up Visits [b,c]	Notes
**Safety Assessments**			
Physical Examination	X		Includes assessment of signs and symptoms and ROS.
Vital Signs	X		
Assessment of Signs and Symptoms	X		
Serious Adverse Events Assessment	X		All AE/SAEs to be collected for up to 100 days after the last dose of study drug or 90 days after surgery, whichever is longer.
Adverse Events Assessment	X		All AE/SAEs to be collected for up to 100 days after the last dose of study drug or 90 days after surgery, whichever is longer.
Review of Concomitant Medications	X	X	Document subsequent cancer therapy
Laboratory Tests	X		On-study local laboratory assessments should be done within 72 hours prior to each dose, starting with C1D1.<br>CBC w/ differential, LFTs (ALTs, AST, total bilirubin, alkaline phosphatase), BUN or serum urea level, creatinine, Ca, Mg, Na, K, Cl, LDH, glucose, amylase, lipase.<br>TSH with reflex Free T4 and Free T3 for nivolumab/ipilimumab arm only.<br>Laboratory tests should be done at Post-neoadjuvant Visit 1 and repeated at Post-neoadjuvant Visit 2, if study-related toxicity persists.
Pregnancy Test	X		Serum or urine

Table 2.-3: Post-Neoadjuvant Period for Those Participants Not Receiving Adjuvant Therapy (CA209816)

Procedure	Post-Neoadjuvant Therapy Visits 1 and 2[a]	Survival Follow-Up Visits [b,c]	Notes
**Efficacy Assessments**			
Tumor Assessments	----See Notes---		Tumor assessments with CT including IV contrast of the chest, including the liver and adrenal glands, and CT or MRI of suspected/known sites of disease should occur every 12 weeks (± 7 days) per RECIST 1.1 for 2 years (104 weeks), then every 6 months (24 weeks ±7 days) for 3 years, and every year (52 weeks ± 7 days) for 5 years or until disease recurrence or progression is confirmed by BICR.<br>Use same imaging method and a machine of the same quality as was used at screening/baseline
**Biomarker Assessments**			
Tumor biopsy for biomarker assessments	--See notes--		Tumor biopsy collection from definitive surgical resection is mandatory on the day of surgery. Tumor biopsy collection is optional but highly recommended from participants at disease progression.<br>All tumor biopsy collection details will be provided in the lab manual.<br>[redacted]
**Patient-reported Outcomes Assessment**			
EQ-5D	X	X	Every 3 months after Post-neoadjuvant Visit 2 for 1 year and then once every 6 months thereafter. May be completed as a phone call if a clinic visit is not otherwise required.
**Participant Survival Status**			
Survival Status	X	X	Every 3 months after Post-neoadjuvant Follow-Up Visit 2. May be accomplished by visit, phone contact, or email to assess subsequent anti-cancer therapy

[a] Post-neoadjuvant Visit 1 = 30 days from the last dose of neoadjuvant therapy ± 7 days. If Post-neoadjuvant Visit 1 falls within 7 days of the scheduled surgery date, then the Post-neoadjuvant Visit 1 visit may coincide with the surgery admission. All Neoadjuvant Visit 1 assessment procedures should be completed prior to surgery. Post-adjuvant Visit 2 = 70 days (± 7 days) from Post-neoadjuvant Visit 1. Post-neoadjuvant visits may coincide with the date of post-surgical follow-up. Timing of Post-neoadjuvant Visits 1 and 2 are based on the pre-surgical nivolumab and chemotherapy timelines.

[b] Survival Follow-up Visits begin 3 months after Post-neoadjuvant Visit 2. Visits should occur every 3 months (± 7 days).

[c] For those participants who have progression confirmed by BICR, participants will continued to be followed for survival status every 3 months by visit, phone contact, or email.

ALT, alanine aminotransferase; AST, aspartate aminotransferase; BUN, blood urea nitrogen; Ca, calcium; CBC complete blood count; Cl, chloride; LDH, lactate dehydrogenase, K, potassium; Mg, magnesium; NA, sodium; T. Bili, total bilirubin; TSH, thyroid stimulating hormone.

Approved v1.0 [redacted]

Table 2.-4: Post-Operative Period for Participants Receiving Adjuvant Chemotherapy (CA209816)

Procedure	During Adjuvant Treatment Visit for Arms A and B[a]	Post Neoadjuvant Therapy Visits 1 and 2[b]	Survival Follow-Up Visits[c,d]	Notes
**Safety Assessments**				
Physical Measurements	X			Weight, BSA (for cytotoxic dosing), and ECOG status. The dosing calculations for the platinum doublet chemotherapy arm should be based on the body weight. If the participant's weight on the day of dosing differs by > 10% from the weight used to calculate the dose. The dose must be recalculated for the dose of the day using the participant's weight of the day, and this updated value becomes the reference for subsequent dosing calculations. All doses should be rounded to the nearest milligram.
Physical Examination	X	X		Includes assessment of signs and symptoms and ROS.
Vital Signs and Oxygen Saturation	X			Include BP, HR, temperature, and oxygen saturation by pulse oximetry
Assessment of Signs and Symptoms	X	X		
Serious Adverse Events Assessment	X	X		All AEs/SAEs to be collected for up to 30 days after the last dose of adjuvant therapy.
Adverse Event Assessments	X	X		All AEs/SAEs to be collected for up to 30 days after the last dose of adjuvant therapy
Review Concomitant Medication	X	X	X	Document subsequent cancer therapy.

Table 2.-4: Post-Operative Period for Participants Receiving Adjuvant Chemotherapy (CA209816)

Procedure	During Adjuvant Treatment Visit for Arms A and B[a]	Post Neoadjuvant Therapy Visits 1 and 2[b]	Survival Follow-Up Visits[c,d]	Notes
Laboratory test	X	X		On-study local laboratory assessments should be done within 72 hours prior to each dose and at Post-neoadjuvant Visit 1. To be repeated at Post-neoadjuvant Visit 2 if study-related toxicity persists.<br>CBC w/ differential, albumin, LFTs (ALT, AST, total bilirubin, alkaline, phosphatase), BUN or serum urea level, creatinine, Ca, Mg, Na, K, Cl, LDH, glucose, amylase, lipase.<br>TSH with reflex Free T4 and Free T3 for nivolumab/ipilimumab arm only.
Pregnancy test	X	X		Serum or urine per standard prescribing information.
**Efficacy Assessments**				
Tumor Assessments	---See Notes---			Tumor assessments using CT with contrast of the chest, including liver and adrenal glands, and CT or MRI of suspected/known sites of disease should occur every 12 weeks (± 7 days) per RECIST 1.1 for 2 years (104 weeks), then every 6 months (24 weeks ± 7 days) for 3 years, and every year (52 weeks ± 7 days) for 5 years or until disease recurrence or progression is confirmed by BICR. Use same imaging methods and machine of the same quality as was used at screening/baseline.
**Biomarker Assessment**				
Exploratory Biomarker	---See Notes---			Tumor biopsy collection from definitive surgical resection is mandatory on the day of the surgery. Tumor biopsy collection is optional but highly recommended from participants at disease progression.<br>All tumor biopsy collection details will be provided in the lab manual.<br>[redacted]

Table 2.-4: Post-Operative Period for Participants Receiving Adjuvant Chemotherapy (CA209816)

Procedure	During Adjuvant Treatment Visit for Arms A and B[a]	Post Neoadjuvant Therapy Visits 1 and 2[b]	Survival Follow-Up Visits[c,d]	Notes
**Patient-reported Outcomes Assessment**				
EQ-5D-3L	X	X	X	Every 3 months after Post-neoadjuvant Visit 2 for 1 year and then once every 6 months thereafter. May be completed as a phone call if a clinical visit is not otherwise specified.
**Participant Survival Status**				
Survival Status	X	X	X	Every 3 months after Post-neoadjuvant Follow-Up Visit 2. May be accomplished by visit, phone contact, or email to assess subsequent anti-cancer therapy
**Study Treatment**				
Administer Adjuvant Chemotherapy ±PORT	X			Post-operative radiation therapy (PORT) should be administered according to standard of care.

[a] Each cycle is 21 days.

[b] Post-neoadjuvant Visit 1 = 30 days from the last dose of neoadjuvant therapy ± 7 days. If Post-neoadjuvant Visit 1 falls within 7 days of the scheduled surgery date, then the Post-neoadjuvant Visit may coincide with the surgery admission. All Post-neoadjuvant Visit 1 assessment procedures should be completed prior to surgery. Post-neoadjuvant Visit 2 = 70 days (± 7 days) from Post-neoadjuvant Visit 1. Post-neoadjuvant Visit 2 may coincide with the date of post-surgical follow-up. Timing of Post-neoadjuvant Visits 1 and 2 are based on the pre-surgical nivolumab and chemotherapy timelines.

[c] Survival Follow-up Visits begin 3 months after Post-neoadjuvant Visit 2. Visits should occur every 3 months (± 7 days).

[d] For those participants who have progression confirmed, participants will continued to be followed for survival status every 3 months by visit, phone contact, or email.

ALT, alanine aminotransferase; AST, aspartate aminotransferase; BP, blood pressure; BUN, blood urea nitrogen; Ca, calcium; CBC complete blood count; Cl, chloride; HR, heart rate; ECOG, Eastern Cooperative Oncology Group; LDH, lactate dehydrogenase, K, potassium; Mg, magnesium; NA, sodium; ROS, review of systems; T.Bili, total bilirubin; PORT post-operative radiation therapy.

3. INTRODUCTION

Nivolumab (BMS-936558) is a fully human, IgG4 (kappa) isotype mAb that binds PD-1 on activated immune cells and disrupts engagement of the receptor with its ligands PD-L1 (B7 H1/CD274) and PD-L2 (B7-DC/CD273), thereby abrogating inhibitory signals and augmenting the host antitumor response. In early clinical trials, nivolumab has demonstrated activity in several tumor types, including melanoma, renal cell carcinoma (RCC), and nonsmall cell lung cancer (NSCLC).

CTLA-4, an activation-induced T-cell surface molecule, is a member of the CD28:B7 immunoglobulin superfamily that competes with CD28 for B7. The proposed mechanism of action for ipilimumab is interference of the interaction of CTLA-4 with B7 molecules on APCs, with subsequent blockade of the inhibitory modulation of T-cell activation promoted by the CTLA 4/B7 interaction.

Nivolumab is in clinical development for the treatment of patients with NSCLC, RCC, melanoma, squamous cell carcinoma of the head and neck (SCCHN) and other tumors (eg, glioblastoma multiforme, mesothelioma, small cell lung cancer, gastric). The combination of nivolumab and ipilimumab is in clinical development for the treatment of NSCLC, RCC, and other tumors. Opdivo® is approved in the United States (US), European Union, and other countries for the treatment of patients with unresectable or metastatic melanoma, advanced NSCLC with progression on or after platinum-based chemotherapy, advanced RCC whose disease progressed on an antiangiogenic therapy (US only), and classical Hodgkin lymphoma that has relapsed or progressed after autologous hematopoietic stem cell transplantation and post-transplantation brentuximab vedotin treatment (US only). Opdivo is also approved in combination with Yervoy® in unresectable and metastatic melanoma.

3.1 Study Rationale

Approximately 80% of lung cancer cases are NSCLC, with most patients presenting with late-stage disease. Of patients with NSCLC, 20% present with stage I or II disease, whereas 30% present with stage III disease and 50% with stage IV disease. With enhanced lung cancer screening techniques, the percentage of those diagnosed during the early stages may increase over the duration of the trial. A standard TNM staging system is used to determine the staging for NSCLC (Appendix 1). Patients with pathologic stage I NSCLC have a 5-year survival of approximately 60%. Stage II to III NSCLC patients have a 5-year survival of approximately 25% to 40%.[1] Surgical resection remains the mainstay of treatment for stage I and II patients; however, despite potential curative surgery, approximately 50% of stage IB and 60-75% of stage I-II NSCLC patients will relapse and eventually die of their disease.[2,3] A rational approach to improve survival in these patients is to eradicate micrometastatic disease and to minimize the risk of relapse with adjuvant or neoadjuvant chemotherapy. Many adjuvant studies have been performed, and these trials are summarized in Table 3.1-1. Although there are some conflicting results, the overall evidence from these studies suggests that adjuvant platinum doublet chemotherapy is beneficial for good Performance Status patients with stage ≥ I disease. The benefit to stage IB patients is less clear and may depend on the size of the primary tumor and other risk factors. The LACE

Approved v1.0 [redacted]

meta-analysis of modern adjuvant and neoadjuvant trials, all of which used cisplatin-based chemotherapy, suggested a 5% absolute survival advantage at 5 years from adjuvant chemotherapy with the benefit being greatest for stage II and IIIA patients. A 2010 meta-analysis including both older and more recent trials confirmed the survival benefit shown in the LACE meta-analysis and also suggested a benefit of adjuvant chemotherapy for stage IB disease.[4]

Table 3.1-1: Select Adjuvant NSCLC Studies

Trial	Stage	Treatment	# of Pts	5 Yr OS	HR	P Value
ECOG 1505	I-III	Cis-doublet<br>Cis-double+Bev	749<br>752	72 mo mOS in both arms	0.99	0.9
ALPI	I - III	Surg<br>MVP	603<br>601	45%<br>50%	0.96	0.59
IALT	I – III	Surg<br>Cis-based	935<br>932	40%<br>44.5%	0.86	<0.03
ANITA	IB - IIIA	Surg<br>Cis-Vin	433<br>407	43%<br>51%	0.80	0.017
BLT	I - IIIA	Surg<br>Cis-based	189<br>192	58%<br>60%	1.02	0.90
NCIC/JBR10	IB – II	Surg<br>Cis-Vin	240<br>242	54%<br>69%	0.69	0.03
CALGB	IB	Surg<br>Carb-pac	171<br>173	57%<br>59%	0.80	0.10
ALPI	I - III	Surg<br>MVP	603<br>601	45%<br>50%	0.96	0.59

ALPI, Adjuvant Lung Cancer Project Italy; OS, overall survival; HR, hazard ratio; ECOG, Eastern Cooperative Oncology Group, IALT, International Adjuvant Lung Cancer Trial; ANITA, Adjuvant Navelbine International Triallist Association; BLT, Big Lung Trial; NCI-C, National Cancer Institute-Canada; CALGB, Cancer and Leukemia Group B; MVP, mitomycin, vindesine, cisplatin; Cis-Vin, cisplatin-vinorelbine; Carb-pac, carboplatin-paclitaxel.

The early-stage (IB-III) NSCLC represents a population of high unmet need with a 5-year survival rate of 25-50%.[2,3,5] The current available standard of care (SOC) only provides a 5% absolute improvement in 5-year overall survival (OS).[6,7,8,9] The SOC comprises adjuvant or neoadjuvant platinum doublet chemotherapy for patients with operable stage IB-IIIA NSCLC or chemoradiation for patients with unresectable stage IIIA/B NSCLC. Follow-up of adjuvant trials are long and may require decades before a new treatment is introduced into clinical practice. Preoperative or neoadjuvant chemotherapy has been assessed in a number of trials for patients with operable NSCLC. A meta-analysis based on 7 trials involving 988 patients suggested that neoadjuvant chemotherapy improved OS when given preoperatively in a similar magnitude to those observed with adjuvant chemotherapy.[10] Neoadjuvant therapy offers the possibility for the

identification of surrogate clinical and biological markers that may correlate with response to therapy and a potential long-term outcome. Studies that address the role of preoperative chemotherapy have found chemotherapy compliance to be favorable in the preoperative setting.[6,7,8] Two Phase 3 registrational trials are currently on-going to explore the potential benefit of nivolumab in the adjuvant and locally advanced unresectable NSCLC setting. In addition, the safety and efficacy profile of neoadjuvant nivolumab monotherapy or in combination (nivolumab plus ipilimumab or nivolumab plus chemotherapy) are being evaluated in ongoing trials.

This Phase 3 study, CA209816, will evaluate the clinical efficacy and will establish the safety of nivolumab and ipilimumab in operable lung cancer. Specifically, this study will compare the major pathological response (MPR) rate among participants with Stage IB-IIIa NSCLC treated with neoadjuvant nivolumab and ipilimumab to the MPR rate in participants treated with platinum doublet chemotherapy.

3.1.1 Research Hypothesis

In participants with stage IB (≥ 4 cm), II or IIIA (N2), PD-L1+ (≥1%) NSCLC considered resectable by the local multidisciplinary team, administration of neoadjuvant nivolumab and ipilimumab (up to 3 cycles of nivolumab with a single dose of ipilimumab) has superior efficacy to neoadjuvant platinum doublet chemotherapy (up to 3 cycles).

3.2 Background

3.2.1 Indication Background

Approval of nivolumab in advanced NSCLC was based on 2 Phase 3 trials (CheckMate 017 and CheckMate 057) which demonstrated survival benefit over docetaxel across histologies. The approval in squamous NSCLC was based on the results of CA209017, a randomized trial of nivolumab versus docetaxel. The median OS for patients in the nivolumab arm was 9.2 months versus 6 months for those in the docetaxel arm (HR = 0.59). Improvement in survival was observed for nivolumab regardless of PD-L1 expression, though there was a trend toward better efficacy for those with PD-L1 expressing tumors. A single-arm trial (CA209063) of 117 patients with metastatic squamous NSCLC with progression after platinum-based chemotherapy and at least 1 additional systemic regimen showed a 15% objective response rate (ORR); 59% of participants with an ORR had response durations of 6 months or longer.[11]

The approval of nivolumab for the treatment of non-squamous NSCLC is based on a second Phase 3 study, CA209057, which met its primary endpoint of superior OS of nivolumab versus docetaxel in patients with previously treated non-squamous NSCLC at a preplanned interim analysis. Patients in the nivolumab arm had a 27% reduction in risk of death (HR = 0.73; P = 0.0015). Interaction P values, reported for PD-L1 expression subgroups by each of the predefined expression levels, suggested a clinically important signal of a predictive association. Nivolumab also significantly improved ORR vs docetaxel (P=0.0246), with ORR as high as 36% in patients with PD-L1 expressing tumors. OS approximately doubled with nivolumab vs docetaxel at 1%, 5% and 10% PD-L1 expression level. In contrast, no statistically significant difference in OS was seen between nivolumab and docetaxel when PD-L1 was not expressed in the tumor, although

Approved v1.0 [redacted]

these patients also experienced durable responses, and the safety profile was more favorable for nivolumab vs docetaxel.[12]

Although the first-line CA209026 trial did not demonstrate that single agent nivolumab provided superior efficacy over platinum doublet chemotherapy, the efficacy of nivolumab was similar to that of chemotherapy in this first line patient population. Nivolumab (3 mg/kg q 2 weeks) plus ipilimumab (1 mg/kg q 6 weeks) demonstrated an ORR of 39% among 38 first-line NSCLC patients. This regimen also demonstrated an acceptable safety profile, a median duration of response that had not been reached at 11.8 months, and a one-year overall survival of 69%.[23] This compares to an ORR of 26-36% for platinum doublet chemotherapy in Western and international populations, and a median overall survival of 7.5-14.9 months. These data suggest that nivolumab plus ipilimumab has the potential to provide superior MPR and event-free survival compared to platinum doublet chemotherapy in early-stage NSCLC, and, specifically as neoadjuvant treatment.[13]

In general, nivolumab is well tolerated, with a favorable safety profile relative to anticipated toxicities based on an immunostimulatory mechanism of action. Nivolumab is currently in Phase 3 development in the first-line metastatic and the early stage NSCLC settings.

A detailed description of the chemistry, pharmacology, efficacy, and safety of nivolumab is provided in the Investigator's Brochure and local package insert.

3.2.2 Nivolumab Mechanism of Action

Cancer immunotherapy rests on the premise that tumors can be recognized as foreign rather than as self and can be effectively attacked by an activated immune system. An effective immune response in this setting is thought to rely on immune surveillance of tumor antigens expressed on cancer cells that ultimately results in an adaptive immune response and cancer cell death. Meanwhile, tumor progression may depend upon acquisition of traits that allow cancer cells to evade immunosurveillance and escape effective innate and adaptive immune responses.[14,15,16] Current immunotherapy efforts attempt to break the apparent tolerance of the immune system to tumor cells and antigens by either introducing cancer antigens by therapeutic vaccination or by modulating regulatory checkpoints of the immune system. T-cell stimulation is a complex process involving the integration of numerous positive as well as negative co-stimulatory signals in addition to antigen recognition by the T-cell receptor (TCR).[17] Collectively, these signals govern the balance between T-cell activation and tolerance.

PD-1 is a member of the CD28 family of T-cell co-stimulatory receptors that also includes CD28, CTLA 4, ICOS, and BTLA.[18] PD-1 signaling has been shown to inhibit CD-28-mediated upregulation of IL-2, IL-10, IL-13, interferon-γ (IFN-γ) and Bcl-xL. PD-1 expression has also been noted to inhibit T cell activation, and expansion of previously activated cells. Evidence for a negative regulatory role of PD-1 comes from studies of PD-1 deficient mice, which develop a variety of autoimmune phenotypes.[19] These results suggest that PD-1 blockade has the potential to activate anti-self T-cell responses, but these responses are variable and dependent upon various

Approved v1.0 [redacted]

host genetic factors. Thus, PD-1 deficiency or inhibition is not accompanied by a universal loss of tolerance to self-antigens.

In vitro, nivolumab (BMS-936558) binds to PD-1 with high affinity (EC50 0.39-2.62 nM), and inhibits the binding of PD-1 to its ligands PD-L1 and PD-L2 (IC50 ± 1 nM). Nivolumab binds specifically to PD-1 and not to related members of the CD28 family such as CD28, ICOS, CTLA-4 and BTLA. Blockade of the PD-1 pathway by nivolumab results in a reproducible enhancement of both proliferation and IFN-γ release in the mixed lymphocyte reaction (MLR). Using a CMV restimulation assay with human PBMC, the effect of nivolumab on antigen specific recall response indicates that nivolumab augmented IFN-γ secretion from CMV specific memory T cells in a dose-dependent manner versus isotype-matched control. In vivo blockade of PD-1 by a murine analog of nivolumab enhances the anti-tumor immune response and result in tumor rejection in several immunocompetent mouse tumor models (MC38, SA1/N, and PAN02).[20]

3.2.3 Ipilimumab Mechanism of Action

CTLA-4, an activation-induced T-cell surface molecule, is a member of the CD28:B7 immunoglobulin superfamily that competes with CD28 for B7. CTLA-4 mediated signals are inhibitory and turn off T cell-dependent immune responses.[21] Ipilimumab is a fully human monoclonal IgG1κ that binds to the CTLA-4 antigen expressed on a subset of T cells from human and nonhuman primates. The proposed mechanism of action for ipilimumab is interference of the interaction of CTLA-4 with B7 molecules on APCs, with subsequent blockade of the inhibitory modulation of T-cell activation promoted by the CTLA 4/B7 interaction.

3.2.4 Nivolumab Combined with Ipilimumab

CA209012 is a multi-arm Phase 1b trial evaluating the safety and tolerability of nivolumab in patients with chemotherapy-naïve advanced NSCLC, as either a monotherapy or in combination with other agents including ipilimumab, at different doses and schedules. The primary endpoint of the study was safety with secondary endpoints of objective response rates (ORR) and 24-week PFS. Exploratory endpoints included OS and efficacy by PD-L1 expression. In the study, patients were tested for PD-L1 expression and 68% of participants in the Q12W cohort and 77% of patients in the Q6W cohort expressed PD-L1. Participants were randomized to nivolumab 3 mg/kg Q2W + ipilimumab 1 mg/kg Q12W (n=38), nivolumab 3 mg/kg Q2W + ipilimumab 1 mg/kg Q6W (n=39) and nivolumab 3 mg/kg Q2W (n=52). The confirmed ORR was 47% (N3 Q2W + I1 Q12W), 39% (N3 Q2W + I1 Q6W) and 23% (N3 Q2W). The median duration of response (DOR) was not reached.[22] Improved ORR were also consistently seen at all levels of PD-L1 expression. In the N3 Q2W + I1 Q12 arm, ORRs were 30%, 57%, and 100% in participants with <1%, ≥1%, and ≥50% of PD-L1 expression levels, respectively. In the N3 Q2W and I1 Q6W arm, ORRs were 0%, 57%, and 86% in <1%, ≥1%, and ≥50% PD-L1 expression levels, respectively.[23]

The rate of treatment-related adverse events in the Q12W (82%) and Q6W (72%) arms were comparable to monotherapy (72%). In the study, Grades 3 to 4 AEs were 37%, 33%, and 19% for the Q12W, Q6W and nivolumab monotherapy arms, respectively. Treatment-related Grade 3-4 AEs lead to discontinuation in 5% and 8% of patients in the Q12W and Q6W cohorts, respectively,

Approved v1.0 [redacted]

and were similar to nivolumab monotherapy. There were no treatment-related deaths. The treatment-related select AEs in patients administered the optimized dosing scheduled (3 mg/kg of nivolumab every 2 weeks plus 1 mg/kg of ipilimumab every 6 weeks) were skin-related (36%), gastrointestinal (23%), endocrine (20%), and pulmonary (5%), and there were ≤ 5% treatment related grade 3/4 AEs per category.

3.3 Benefit/Risk Assessment

The early-stage (IB-III) NSCLC represents a population of high unmet need with a 5-year survival rate of 25-50%. The current available SOC, including adjuvant or neoadjuvant platinum doublet chemotherapy, only provides a 5% absolute improvement in 5-year OS.

Follow-up of adjuvant trials are long and may require decades before a new treatment is introduced into clinical practice. Preoperative or neoadjuvant chemotherapy has been assessed in a number of trials for participants with operable NSCLC. A meta-analysis based on 7 trials involving 988 participants suggested that neoadjuvant chemotherapy improved OS when given preoperatively in a similar magnitude to those observed with adjuvant chemotherapy. Several studies have also shown preoperative cytotoxic chemotherapy to be safe prior to surgical resection of NSCLC with no difference in extent of surgical procedures performed, operative morbidity and mortality.

The clinical activity of nivolumab observed to date in NSCLC, including 2 positive Phase 3 studies demonstrating prolonged survival with nivolumab monotherapy compared to docetaxel in squamous and non-squamous NSCLC after platinum failure, suggests the potential for improved clinical outcomes. CA209057 (non-squamous NSCLC) study demonstrated OS was superior for participants receiving nivolumab compared to those receiving docetaxel. In this study, interaction P values reported for PD-L1 expression subgroups by each of the pre-defined expression levels suggested a clinically important signal of a predictive association. Higher confirmed ORRs in PD-L1 expressors were seen in the combination arm compare to the nivolumab monotherapy arm in CA209012. Based on these data, CA209816 will stratify participants based on PD-L1 status, disease stage at randomization, and gender.

In general, nivolumab is well tolerated, with a favorable safety profile relative to anticipated toxicities based on an immunostimulatory mechanism of action. Overall, the safety profile of nivolumab monotherapy as well as combination therapy is manageable and generally consistent across completed and ongoing clinical trials with no maximum tolerated dose (MTD) reached at any dose tested up to 10 mg/kg. Most AEs were low-grade (Grade 1 to 2) with relatively few related high-grade (Grade 3 to 4) AEs. There was no pattern in the incidence, severity, or causality of AEs with respect to nivolumab dose level.

Nivolumab plus ipilimumab provides a higher ORR (39%) than nivolumab alone.[22] In first line metastatic NSCLC, this regimen also provided impressive overall survival, with an acceptable safety profile. These data support our belief that nivolumab plus ipilimumab will demonstrate superior benefit over platinum chemotherapy doublets.in the neoadjuvant setting with an acceptable safety profile. Extensive details on the safety profile of nivolumab and ipilimumab are available in the respective Investigator Brochures and will not be repeated herein.

Approved v1.0 [redacted]

A pattern of immune-related AEs has been defined, for which management algorithms have been developed; these are provided in Appendix 4. Most high-grade events were manageable with the use of corticosteroids or hormone replacement therapy (endocrinopathies) as instructed in these algorithms.

The potential benefit of nivolumab plus ipilimumab as neoadjuvant therapy in Stage I-III NSCLC is not yet known. The platinum doublet chemotherapy regimens have well described safety profiles, characterized by myelosuppression and other regimen-specific non-hematologic toxicities, such as peripheral neuropathy, nausea/vomiting, and renal impairment.

In order to assess the potential benefit of nivolumab plus ipilimumab over platinum doublet chemotherapy as neoadjuvant therapy in Stage I-III resectable NSCLC, this trial will randomize participants to 1 of 2 arms: nivolumab plus ipilimumab or physician's choice of platinum doublet. To assure an ongoing favorable risk/benefit assessment for participants enrolled onto CA209816, an independent Data Monitoring Committee (DMC) will be utilized to monitor the safety and activity of the treatments throughout the conduct of the trial.

4. OBJECTIVES AND ENDPOINTS

Table 4.-1: Objectives and Endpoints

Objectives	Endpoints
**Primary**	
• To compare the MPR rate by blinded independent pathology review (BIPR) of participants receiving nivolumab and ipilimumab to that of participants receiving platinum doublet chemotherapy in operable stage IB (≥ 4 cm), II, or resectable IIIA (N2) PD-L1+ (≥ 1%) NSCLC	• MPR rate, defined as number of randomized participants with <10% residual tumor in lung and lymph nodes as evaluated by BIPR, divided by the number of randomized participants for each treatment group.
**Secondary**	
• To compare the event-free survival (EFS) by blinded independent central radiology review (BICR) in participants receiving nivolumab and ipilimumab to participants receiving platinum doublet chemotherapy in operable stage IB (≥ 4 cm), II, or resectable IIIA (N2) PD-L1+ (≥ 1%) NSCLC<br>• To assess the OS of participants receiving nivolumab and ipilimumab to that of participants receiving platinum doublet chemotherapy in operable stage IB (≥ 4 cm), II, or resectable IIIA (N2) PD-L1+ (≥ 1%) NSCLC<br>• To assess complete pathological response (pCR) by BIPR of participants receiving nivolumab and ipilimumab compared to participants receiving platinum doublet chemotherapy in operable stage IB (≥ 4 cm), II, or resectable IIIA (N2) PD-L1+ (≥1%) NSCLC	• EFS defined as the length of time from randomization to any of the following events: progression of disease, recurrence disease, or death due to any cause. Progression/recurrence will be assessed by BICR per Response Evaluation Criteria in Solid Tumors (RECIST) 1.1.<br>• OS is defined as the time between the date of randomization and the date of death. OS will be censored on the last date a participant was known to be alive.<br>• pCR is defined as the absence of residual tumor in lung and lymph nodes as evaluated by BIPR

Approved v1.0 [redacted]

Table 4.-1: Objectives and Endpoints

Objectives	Endpoints
**Tertiary/Exploratory**<br>• To assess clinical response rate (cRR) by BICR of participants receiving nivolumab and ipilimumab compared to participants receiving platinum doublet chemotherapy in operable stage IB (≥ 4 cm), II, or resectable IIIA (N2) PD-L1+ (≥ 1%) NSCLC<br>• To assess the MPR rate , pCR, cRR, EFS, and OS in all randomized (PD-L1 ≥ 1% and PD-L1< 1%/not evaluable/indeterminate) early-stage NSCLC participants treated with nivolumab and ipilimumab compared to those treated with platinum doublet chemotherapy<br>• To assess the feasibility of surgery and rate of peri- and post-operative complications (within 90 days of surgery) in participants receiving nivolumab and ipilimumab compared to participants receiving platinum doublet<br>• To assess the safety and tolerability of nivolumab and ipilimumab compared to platinum doublet chemotherapy in early stage NSCLC<br>• To assess pharmacokinetics of the nivolumab and ipilimumab in participants with early stage NSCLC<br>• To investigate the potential association of pre-treatment and on-treatment candidate biomarkers in peripheral blood and in tumor tissue with safety and clinical efficacy measurements as well as to explore the pharmacodynamic properties of nivolumab and ipilimumab in early-stage NSCLC<br>• To assess the participant's overall health status and health utility using the 3-level version of the EQ-5D-3L visual analog scale (VAS) and utility index, respectively	• cRR is defined as proportion of all randomized participants whose best overall radiological response (BOR) prior to definitive surgery is either a complete response or partial response per RECIST 1.1 criteria by BICR<br>• MPR rate, pCR, cRR, EFS, and OS as described above<br>• Proportion of delayed or canceled surgery, duration of surgery, length of hospital stay, surgical approach, incidence of AE/SAE associated with surgery, including pneumonitis, ARDS, re-admission to the Intensive Care Unit, atrial fibrillation or other supraventricular tachycardia (SVT) to 90 days post-surgery<br>• Safety and tolerability will be measured by incidence of AE, SAE, immune-related AEs, deaths, and laboratory abnormalities<br>• Pharmacokinetic endpoints - See Table 9.5-1<br>• Biomarker endpoints [redacted]<br>• Change in EQ-5D-3L scores

5. STUDY DESIGN

5.1 Overall Design

This is an open-label, randomized clinical trial of up to 3 cycles of neoadjuvant nivolumab (3 mg/kg every 2 weeks) and a single dose of 1 mg/kg dose of ipilimumab vs platinum doublet chemotherapy (up to 3 cycles) as neoadjuvant treatment in participants with early stage (Stage IB [≥ 4 cm], II, and resectable IIIA [N2]) NSCLC.

Participants will be randomized between 2 arms in a 1:1 ratio. Eligible participants will be stratified by:

- PD-L1 expression (≥1% or <1%/not evaluable/indeterminate)
- Disease stage (IB/II vs IIIA)
- Gender

PD-L1 status will be determined by immunohistochemical (IHC) staining of PD-L1 protein in the submitted tumor sample and categorized as follows:

- PD-L1 positive - defined as ≥ 1% tumor cell membrane staining positive in a minimum of 100 evaluable tumor cells
- PD-L1 negative – defined as < 1% tumor cell membrane staining positive in a minimum of 100 evaluable tumor cells
- PD-L1 not evaluable/indeterminate - defined as participants with insufficient sample quantity or quality to stain for PD-L1 status or those participants in whose samples PD-L1 status could not be determined despite appropriate amounts of tissue sample. For this category, key efficacy and safety parameters will be summarized and grouped with PD-L1 negative participants. No more than 10% of participants enrolled in this trial will be PD-L1 not evaluable/indeterminate category.

Screening begins by establishing the participant's initial eligibility and signing of the informed consent (ICF). Tumor tissue (archival [slides/block ≤ 6 month] or recent tumor biopsy) must be submitted to a third-party vendor for determination of PD-L1 status prior to randomization.

All screening assessments and procedures must be performed in accordance with Table 2.-1.

The Treatment Phase begins when the randomization call is made into the Interactive Response Technology (IRT). The participant will be randomly assigned to 1 of the 2 treatment arms: Arm A or Arm B. The first dose of study treatment must begin within 7 days of randomization.

Participants randomized into Arm A will receive nivolumab 3 mg/kg IV over 30 minutes every 2 weeks for up to 3 doses (ie, 6 weeks of treatment; each cycle is 14 days). With Cycle 1 only, nivolumab will be followed by a single dose ipilimumab 1 mg/kg IV over 30 minutes.

Participants randomized into Arm B will receive 1 of the following investigator-choice platinum doublet chemotherapy in 3-week cycles up to a maximum of 3 cycles of IV chemotherapy (ie, 9 weeks of treatment; each cycle is 21 days):

- Regimen 1:
  - Vinorelbine 30 mg/m^2 IV push over 10 minutes, Days 1 and 8
  - Cisplatin 75 mg/m^2 IV over 120 minutes Day 1, immediately following vinorelbine
- Regimen 2:
  - Docetaxel 75 mg/m^2 IV over 60 minutes on Day 1
  - Cisplatin 75 mg/m^2 IV over 120 minutes on Day 1, immediately following docetaxel
- Regimen 3 (squamous histology):
  - Gemcitabine 1250 mg/m^2 IV over 30 minutes on Days 1 and 8
  - Cisplatin 75 mg/m^2 IV over 120 minutes on Day 1, immediately following gemcitabine
- Regimen 4 (non-squamous histology only):
  - Pemetrexed 500 mg/m^2 IV over 10 minutes on Day 1
  - Cisplatin 75 mg/m^2 IV over 120 minutes on Day 1, immediately following pemetrexed

Within 7 days of randomization, the participants must receive the first dose of study medication. Participants may be dosed no less than 12 days between doses on the nivolumab arm. If a dose is delayed for any reason, participants should be dosed no later than 7 days following a planned dose on any arm. If more than 7 days delay is needed for any reason, the intended dose should be skipped. If a participant receiving chemotherapy on a Day 1 and Day 8 schedule (ie, cisplatin/gemcitabine) is unable to receive Day 1 of chemotherapy but recovers in time to receive the Day 8 dose, the Day 8 dose of chemotherapy may be administered. Doses that are skipped or missed will not be replaced. Surgery should be performed within 6 weeks after completing up to 3 cycles (last dose) of nivolumab or chemotherapy as indicated by the institutional SOC.

For participants who are unable to tolerate cisplatin, the reasons for intolerability should be documented. If the investigator would like to use a non-cisplatin-containing regimen, the investigator should discuss this with and obtain approval from the Medical Monitor prior to utilization.

<u>All Arms</u>

PET/CT including IV contrast (CT of diagnostic quality) will be performed at baseline and within 7 days prior to planned surgery. Subsequent assessments (CT or MRI) will be performed in accordance with Table 2.-3 and Table 2.-4. Tumor assessments must continue per protocol until disease recurrence/progression is confirmed by BICR per RECIST 1.1 (Appendix 5). Pharmacokinetics assessments are described in Section 9.5, and biomarker assessments are described in Section 9.8. OS will be followed continuously every 3 months via in-person or phone contact after Post-neoadjuvant Follow-up Visit 2.

Following the completion of treatment in Arm A or B, all participants who remain operative candidates will undergo definitive surgery for their NSCLC. Surgery should be performed within 6 weeks after completing nivolumab or chemotherapy treatment.

Prior to surgery, any treatment-related toxicity should have resolved to ≤ Grade 1 or returned to baseline (except for alopecia, fatigue, and neuropathy). Investigators should discuss residual endocrine toxicities or mild renal impairment with the Medical Monitor.

All AEs, serious adverse event (SAE), and drug-related AEs resulting in surgical delays and post-surgical complications will be collected. Peri-operative complications, including a delay in planned surgery, pneumonitis, ARDS, re-admission to the Intensive Care Unit, atrial fibrillation or other SVTs, potential immune-related toxicities, and post-operative complications will be collected. Study drug dose omission or delays due to AEs will not be replaced for either arm, and participants should proceed to surgery within the predefined timeframe after standard preoperative evaluation. Surgical complications occurring within 90 days of surgery will be documented and followed until resolution.

Approved v1.0 [redacted]

All AEs will be documented for a minimum of 100 days after the last dose of study drugs or 90 days post-surgery, whichever is longer, and for 30 days after the last dose of adjuvant therapy in participants who receive adjuvant therapy.

Following definitive surgery, participants in each arm may receive up to 4 cycles of adjuvant chemotherapy per institutional standard at the discretion of the investigator. Investigators may choose from the following post-operative regimens:

- Regimen 1:
  - Vinorelbine 30 mg/m^2 IV push over 10 minutes, Days 1 and 8
  - Cisplatin 75 mg/m^2 IV over 120 minutes Day 1, immediately following vinorelbine
- Regimen 2:
  - Docetaxel 75 mg/m^2 IV over 60 minutes on Day 1
  - Cisplatin 75 mg/m^2 IV over 120 minutes on Day 1, immediately following docetaxel
- Regimen 3 (squamous histology):
  - Gemcitabine 1250 mg/m^2 IV over 30 minutes on Days 1 and 8
  - Cisplatin 75 mg/m^2 IV over 120 minutes on Day 1, immediately following gemcitabine
- Regimen 4 (non-squamous histology only):
  - Pemetrexed 500 mg/m^2 IV over 10 minutes on Day 1
  - Cisplatin 75 mg/m^2 IV over 120 minutes on Day 1, immediately following pemetrexed

For participants who are unable to tolerate cisplatin, the reasons for intolerability should be documented. If the investigator would like to use a non-cisplatin-containing regimen, the investigator should discuss this with and obtain approval from the Medical Monitor prior to utilization of a non-protocol regimen.

Postoperative chemotherapy should not commence until pre-operative, treatment-related toxicity has returned to baseline or resolved to ≤ Grade 1 (exceptions for fatigue, alopecia, and neuropathy). Investigators should discuss residual endocrine toxicities or mild renal impairment with the Medical Monitor.

Assessments during adjuvant therapy are outlined in Table 2.-4.

Post-operative radiation should be administered per institutional standard of care.

The Survival Follow-Up Phase begins 3 months after Post-neoadjuvant Follow-up Visit 2. Participants will be followed every 3 months for survival. Survival follow-up visits may be performed by phone contact or office visit.

The study design schematic is presented in Figure 5.1-1.

Figure 5.1-1: Study Design Schematic

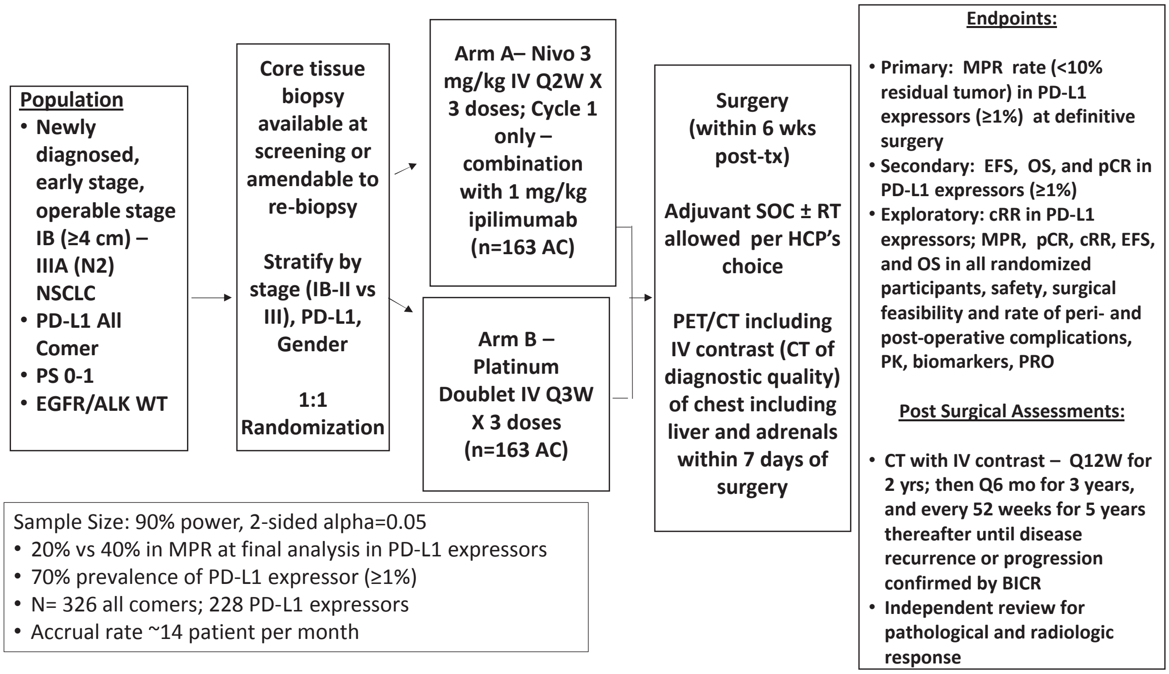

5.1.1 Data Monitoring Committee and Other External Committees

When required, adjudicated events will be submitted to the DMC and Health Authorities for review on a specified timeframe in accordance with the adjudication documentation.

An independent Data Monitoring Committee (DMC) will be established to provide oversight of safety and efficacy considerations, study conduct, and risk-benefit ratio in protocol CA209816. An interim DMC review will occur after 15 participants have enrolled in each arm and have completed surgery. Following review, the DMC will recommend continuation, modification, or discontinuation of this study based on reported safety data. Details of DMC responsibilities and procedures will be specified in the DMC charter. Representatives of the Sponsor will serve only as coordinators of the committee, without having full member responsibilities or privileges. In addition, the Sponsor will independently review safety data in a blinded manner during the conduct of this trial to ensure that any safety issues are identified and addressed.

In addition, efficacy will also be reviewed by the DMC for the formal interim analysis of EFS.

Independent Radiology/Pathology Review:

Independent pathology and radiology review will be established for central review and confirmation of endpoints. Images and tumor/lymph node samples will be submitted to these third-party vendors for central review. Sites will be trained prior to enrolling the first study participant. Images and pathology samples acquisition guidelines and submission process will be outlined in the study Imaging/Pathology Manuals to be provided by the vendors. For histologic assessment, all tumor and lymph node tissue should be sectioned at 1 centimeter intervals. For assessments of

pathological response, the percentage of viable tumor cells in at least 1 section per centimeter of the tumor should be evaluated for all tumor and lymph node samples.

Radiologic tumor assessments should be submitted to the third-party radiology vendor as they are performed on an ongoing basis.

Tumor assessments will be sent to and reviewed by a Blinded Independent Central Review (BICR) from a third-party radiology vendor on an on-going basis. At the time of investigator-assessed radiographic progression per RECIST 1.1, the site must request a BICR review confirmation of progression or recurrence. Details of the Blinded Independent Central Review responsibilities and procedures will be specified in the Blinded Independent Central Review charter.

Participants whose disease progression or recurrence is not confirmed by central review will be required to continue tumor assessments (if clinically feasible) according to the protocol-specified schedule. Subsequent tumor assessments must be submitted to the third party radiology vendor for subsequent review and may be discontinued when the investigator and independent radiologists both assess the participant to have met RECIST 1.1 criteria for progression or recurrence.

5.2 Number of Participants

Approximately 587 to 665 participants will be screened, and approximately 326 participants will be randomized to the 2 arms in a 1:1 ratio. Therefore, approximately 163 participants will be included in each arm.

5.3 End of Study Definition

The start of the trial is defined as the first participant's first visit. The primary analysis of MPR rate will take place at the time of surgery. After this analysis, participants will still be followed for EFS and OS. One formal interim analysis for EFS is planned after 140 events have been observed, which is projected to occur approximately 76 months after study initiation. This formal comparison of EFS will allow for early stopping for superiority. The final analysis of EFS secondary endpoint will be conducted after at least 186 participants have experienced an event (approximately 10 years from the start of randomization). [redacted]

5.4 Scientific Rationale for Study Design

5.4.1 Rationale for Open-Label Design

This study will use an open-label design. Due to the obvious difference in chemotherapy- and immunotherapy-related toxicities, histology-dependent chemotherapy options, different dose modification rules for safety management, including different dose delay rules per arm, and different premedication requirements according to chemotherapy, an open-label design is appropriate. An open-label design will also help ensure that immune-related toxicities in participant receiving immunotherapy are promptly identified and managed.

Approved v1.0 [redacted]

Because this study will be open-label, independent pathology and radiology review will be used for central review and confirmation of pathological and clinical responses in all randomized participants to determine all response-related endpoints.

5.4.2 Rationale for Preoperative Systemic Therapy in NSCLC

Randomization and follow-up of adjuvant trials may require decades until a new treatment can be introduced into the early treatment setting. Preoperative chemotherapy has been assessed in a number of trials for patients with resectable NSCLC, though most trials were closed early when the adjuvant chemotherapy data revealed a survival advantage. A meta-analysis based upon 7 trials involving 988 participants suggested that neoadjuvant chemotherapy improved OS when given preoperatively (5-year survival 20% vs 14% without neoadjuvant chemotherapy). This improvement in survival is similar to that noted in the meta-analyses of predominantly adjuvant chemotherapy.[24,25]

Several studies have shown preoperative cytotoxic chemotherapy to be safe prior to surgical resection of NSCLC with no difference in extent of surgical procedures performed, operative morbidity, and mortality.[6,26,27] Immune checkpoint inhibition has the potential to provide benefits in early-stage disease. Among these benefits are the opportunity to evaluate EFS in a moderate-sized population of early-stage NSCLC patients and the potential to demonstrate long-term, disease-free status in these patients.[7,28,29,30]

5.4.3 Rationale for Nivolumab and Ipilimumab for Neoadjuvant NSCLC

In contrast to the adjuvant setting in which only micrometastatic disease is present, one may hypothesize that the higher tumor burden present at the time of induction treatment may be necessary for abundant antigen release and presentation to the immune system, and consequently, development of a robust immune response to immune checkpoint inhibitors.

In an ongoing feasibility trial with nivolumab monotherapy, a major pathological response (MPR) rate was observed in 39% (7/18) of stage IB-IIIA NSCLC participants who were evaluable post-surgery after 2 cycles of nivolumab. One patient achieved a complete pathological response. Major pathological response was defined as < 10% residual viable tumor at resection.[31] Responses were observed across histology and regardless of PD-L1 expression. Nivolumab was well tolerated with a safety profile comparable to that observed in the Phase 3 program. Administration of nivolumab in the neoadjuvant setting was deemed to be feasible with no surgical delays or post-surgical complications (within 30 days post-surgery). This trial has enrolled 18 participants to date and was recently expanded to include an additional 30 participants, who will be treated with nivolumab monotherapy (n=15) and nivolumab plus ipilimumab (n=15). In addition, nivolumab monotherapy will be extended to 3 cycles. It is hypothesized that the addition of a single dose of ipilimumab to 3 doses of nivolumab will result in the achievement of higher rate and deeper pathological responses while maintaining an acceptable safety profile.

The 39% MPR rate from nivolumab in neoadjuvant NSCLC compares with a rate of approximately 20% from platinum doublet chemotherapy in this context. These findings, coupled with the data from Checkmate 012, indicating a high ORR (38%) and 1-year OS (69%) from nivolumab plus

ipilimumab in first-line advanced NSCLC suggest that nivolumab plus ipilimumab may improve both MPR and EFS relative to platinum doublet chemotherapy, while providing an acceptable safety profile in the neoadjuvant treatment of NSCLC.[22]

5.4.4 Rationale for Combination of Nivolumab and Ipilimumab (Arm A):

Combining immunotherapeutic agents with different mechanisms of action offers the possibility of synergistic response. PD-1 and CTLA-4 are both co-inhibitory molecules, but evidence suggests that they use distinct mechanisms to limit T-cell activation. Preliminary indirect data from peripheral T-cell assessments suggests that a given T-cell checkpoint inhibitor may modulate host immune cell phenotype rendering them more susceptible to alternate checkpoint inhibitors and thereby enhancing anti-tumor activity.

Preclinical data indicate that the combination of PD-1 and CTLA-4 receptor blockade may improve antitumor activity. In vitro combinations of nivolumab plus ipilimumab increase IFN-γ production 2- to 7-fold over either agent alone in a mixed lymphocyte reaction. Increased antitumor activity of the combination was also observed in 3 of 5 syngeneic murine cancer models. In a murine melanoma vaccine model, blockade with either CTLA-4 or PD-1 antibodies increased the proportion of CTLA-4 and PD-1-expressing CD4/CD8 tumor infiltrating T effector cells, and dual blockade increased tumor infiltration of T effector cells and decreased intratumoral T regulatory cells, as compared to either agent alone.[32]

The combination of nivolumab and ipilimumab was evaluated in CA209004 (MDX1106-04), a Phase 1b, multiple ascending dose study in participants with treatment-naive and previously-treated advanced melanoma. Results showed promising activity with higher but tolerable toxicity than ipilimumab alone. Based on these data, CA209069, a Phase 2 study, compared the combination to ipilimumab alone in treatment-naïve participants with advanced melanoma: nivolumab 1 mg/kg and ipilimumab 3 mg/kg every 3 weeks for 4 doses followed by nivolumab 3 mg/kg every 2 weeks versus ipilimumab 3 mg/kg every 3 weeks for 4 doses.[33] In participants with BRAF wild type tumors, the ORR was 61% (44/72), including 22% (16/72) CRs in the group treated with the combination, compared to 11% (4/37) with 0 CRs in those treated with ipilimumab alone. The median PFS was not reached in the combination versus 4.4 months for ipilimumab alone (HR = 0.4). Recently, a Phase 3 study (CA209067, n = 945) reported significantly improved PFS and ORR with the combination of nivolumab and ipilimumab versus ipilimumab alone in previously untreated melanoma. The median PFS was 6.9 months (95% confidence interval [CI], 4.3 to 9.5) in the nivolumab group, 11.5 months (95% CI, 8.9 to 16.7) in the nivolumab plus ipilimumab group, and 2.9 months (95% CI, 2.8 to 3.4) in the ipilimumab group. Significantly longer PFS was observed in the nivolumab plus ipilimumab group than in the ipilimumab group (hazard ratio for death or disease progression, 0.42; 99.5% CI, 0.31 to 0.57; $P < 0.001$) and in the nivolumab group than in the ipilimumab group (hazard ratio, 0.57; 99.5% CI, 0.43 to 0.76; $P < 0.001$). The hazard ratio for the comparison between the nivolumab plus ipilimumab group and the nivolumab group was 0.74 (95% CI, 0.60 to 0.92).[34]

Approved v1.0 [redacted]

In addition, deep and durable responses were observed in previously treated, extensive stage small cell lung cancer (SCLC), with a response rate of 31.1% with the combination of nivolumab and ipilimumab.[35]

Based on the initial data in melanoma and the activity observed with nivolumab and ipilimumab in lung cancer, the nivolumab plus ipilimumab combination has been also evaluated as first-line therapy in participants with advanced NSCLC. In CA209012, early combination cohorts evaluated 2 dosing schedules that were studied in the CA209004 study in melanoma[36]:

- Nivolumab 1 mg/kg + ipilimumab 3 mg/kg, every 3 weeks for 4 doses, followed by nivolumab 3 mg/kg q 2 weeks (Arms G and H, n=24);
- Nivolumab 3 mg/kg + ipilimumab 1 mg/kg, every 3 weeks for 4 doses, followed by nivolumab 3 mg/kg q 2 weeks (Arms I and J, n=25)

These regimens resulted in significant toxicity, with 39% of participants discontinuing treatment due to a treatment-related adverse event. Thus, additional combination cohorts were initiated (Arms N, O, P, Q), using lower doses of both nivolumab and ipilimumab, or the approved dose of nivolumab with less frequent dosing of ipilimumab. These new regimens were much better tolerated, and the safety data are not dissimilar to what has been observed in the nivolumab monotherapy cohort (Arm F in CA209012) (Table 5.4.4-1).

Table 5.4.4-1: Treatment-related Adverse Events from Selected Cohorts in CA209012

Arm[a]	No. Participants/ arm	Follow-up time (median, wks)	No. Participants still on treatment	No. Participants with drug-related AEs	No. Participants with grade 3-4 drug-related AEs	No. participants d/c due to drug-related AEs (all grades)
N[b]	31	72	6 (19%)	24 (77%)	9 (29%)	4 (13%)
O[b]	40	27	14 (35%)	29 (73%)	14 (35%)	3 (8%)
P[b]	38	37	20 (53%)	28 (74%)	11 (29%)	2 (5%)
Q[b]	39	34	15 (39%)	27 (69%)	11 (28%)	4 (10%)
F[c]	52	62	5 (10%)	37 (71%)	10 (19%)	5 (10%)

[a] N:nivolumab 1 mg/kg plus ipilimumab 1 mg/kg every 3 weeks x 4, followed by nivolumab 3 mg/kg every 2 weeks; O: nivolumab 1 mg/kg every 2 weeks plus ipilimumab 1 mg/kg every 6 weeks; P: nivolumab 3 mg/kg every 2 weeks plus ipilimumab every 12 weeks; Q: nivolumab 3 mg/kg every 2 weeks plus ipilimumab 1 mg/kg every 6 weeks; F: nivolumab 3 mg/kg every 2 weeks

[b] based on August 2015 database lock

[c] based on March 2015 database lock

Approved v1.0 [redacted]

Activity was observed in all cohorts, with response rates greater than 39% in the 2 cohorts in which nivolumab was dosed at 3 mg/kg (N3). PFS and OS is also encouraging in the nivolumab 3 mg/kg cohorts (Table 5.4.4-2).

Clinical activity was observed in participants with and without PD-L1 expressing tumors, though there was a greater magnitude of efficacy in participants with PD-L1 expressing tumors. In participants with PD-L1 expressing tumors (≥ 1 % level), the response rate was 57% with nivolumab 3 mg/kg and ipilimumab 1 mg/kg every 6 or 12 weeks. For participants with PD-L1 non-expressing tumors, the response rates were lower, but the participant numbers are small. For participants with PD-L1 expressing and non-expressing tumors, the nivolumab 3 mg/kg every 2 weeks plus ipilimumab 1 mg/kg every 6 weeks is being further explored in a Phase 3 study in first-line NSCLC (CA209227).

Table 5.4.4-2: Efficacy of First-Line Treatment of Nivolumab/Ipilimumab Combination in CA209012[36]

	Nivo 3 mg/kg Q2W + Ipi 1 mg/kg Q12W (n=38)	Nivo 3 mg/kg Q2W+ Ipi 1 mg/kg Q6W (n=39)	Nivo 3 Q2W (n=52)
**Confirmed ORR, % (95% CI)**	47 (31, 64)3	39 (23, 55)	23 (13, 37)
**Duration of response, months (95% CI)**	NR (11.3, NR)	NR (8.4, NR)	NR (5.7, NR)
**mPFS, mos (95% CI)**	8.1 (5.6, 13.6)	3.9 (2.6, 13.2)	3.6 (2.3, 6.6)
**Median length of follow-up, mos (range)**	12.9 (0.9, 18.0)	11.8 (1.1, 18.2)	14.3 (0.2, 30.1)

CI, confidence interval; mPFS, median progression-free survival; PFS, progression-free survival; ORR, objective response rate

Table 5.4.4-3: Efficacy by PD-L1 Expression[37]

	Nivo 3 mg/kg Q2W + Ipi 1 mg/kg Q12W	Nivo 3 mg/kg Q2W + Ipi 1 mg/kg Q6W	Nivolumab 3 mg/kg Q2W
**ORR, % (n/N)**			
< 1% PD-L1	30 (3/10)	0 (0/7)	14 (2/14)
≥ 1% PD-L1	57 (12/21)	57 (13/23)	28 (9/32)
≥ 50% PD-L1	100 (6/6)	86 (6/7)	50 (6/12)
**Median PFS (95% CI), mo**			
< 1% PD-L1	4.7 (0.9, NR)	2.4 (1.7, 2.9)	6.6 (2.0, 11.2)
≥ 1% PD-L1	8.1 (5.6, NR)	10.6 (3.6, NR)	3.5 (2.2, 6.6)
≥ 50% PD-L1	13.6 (6.4, NR)	NR (7.8, NR)	8.4 (2.2 (NR)

Approved v1.0 [redacted]

Table 5.4.4-3: Efficacy by PD-L1 Expression[37]

	Nivo 3 mg/kg Q2W + Ipi 1 mg/kg Q12W	Nivo 3 mg/kg Q2W + Ipi 1 mg/kg Q6W	Nivolumab 3 mg/kg Q2W
**1-year OS rate (95% CI), %**			
< 1% PD-L1	NC	NC	79 (47, 93)
≥ 1% PD-L1	90 (66, 97)	83 (60, 93)	69 (50, 82)
≥ 50% PD-L1	NC	100 (100, 100)	83 (48, 96)

NC, not calculated (when > 25% of patients are censored); NR, not reached due to high percentage of ongoing response.

Combination data based on a February 2016 database lock; monotherapy data based on a March 2015 database lock except for IS data, which are based on an August 2015 database lock.

5.4.5 Rationale for Major Pathological Response Endpoint

Pathological complete response (pCR) has been long known to correlate with survival after tumor resection. Recently, the FDA approved pertuzumab based on pathological response rate for the treatment of locally advanced breast cancer. However, only 4% of NSCLC patients treated with neoadjuvant chemotherapy achieve pCR, limiting utility of this outcome. In a recent publication by Hellmann and colleagues, major pathological response, defined as ≤ 10% viable tumor, was proposed as a better surrogate for survival and is observed in approximately 22% of NSCLC patients treated with neoadjuvant chemotherapy.[38] This position is supported by an analysis showing that NSCLC patients with > 60% pathological response had a median OS (mOS) of 61 months compared to 22 months in patients with < 60% pathological response (P=0.03).[39] Pataer and colleagues did a comprehensive analysis of 192 patients with resected stage I–IV NSCLC and treated with neoadjuvant chemotherapy and 166 patients treated with surgery alone.[40] Using a score system that quantifies the percentage of viable tumor cells in at least 1 section per centimeter of the tumor of greatest diameter (5 to 30 slides examined for each case), the authors demonstrated that there is a statistically significant correlation between higher percentage of viable cells and shorter disease-free and OS in patients who received induction treatment. This correlation was not evident in patients treated with upfront surgery alone. In this cohort, 89% of the neoadjuvant-treated patients received platinum and a taxane-based regimen, and a pathological response (defined as ≤ 10% viable tumor cells) occurred in 19% of the patients. The 5-year recurrence-free survival for patients with and without a pathological response were 78% and 35%, respectively (P < 0.001). The 5-year overall survival for patients with and without a pathological response were 85% and 40%, respectively (P < 0.0001). These results support the use of major pathologic response as a surrogate endpoint for recurrence-free and overall survival in patients with NSCLC treated with neoadjuvant chemotherapy.

5.4.6 Rationale for Main Inclusion and Exclusion Criteria:

The OS benefit of several adjuvant therapies in this population is presented in Table 3.1-1. The choice of stage II and resectable IIIA (N2) NSCLC was made because these participants have a high risk of tumor relapse and death with current standard therapy, including surgery with

preoperative or postoperative chemotherapy. There is an urgent need for improved, novel therapies for this group of participants. Participants with stage IB NSCLC with primary tumors of ≥ 4 cm diameter have been included because these participants are also at a high risk of tumor relapse and may be considered candidates for standard adjuvant chemotherapy.[2,41] It is anticipated that participants enrolled on this study may require adjuvant chemotherapy with or without radiation, and the administration of adjuvant chemotherapy will commence if considered clinically indicated in the postoperative period.

5.4.7 Rationale for Choices of Platinum-based Chemotherapy Doublet

The regimens selected for use in this study are commonly used adjuvant and neoadjuvant therapies in the clinical setting. A significant difference in outcome was not seen between participants using the regimens proposed in this trial.[42] Cisplatin-based therapy has been shown to significantly improve survival.[43] Several clinical trials have found a significant improvement in OS when using platinum-based chemotherapy regimens.[44,45,46,47,48]

5.5 Justification for Dose

5.5.1 Rationale for Shorter Infusion Times for Nivolumab

Nivolumab has been administered safely over 60 minutes at doses ranging up to 10 mg/kg safely over long treatment duration. In Study CA209010 (a Phase 2, randomized, double-blinded, dose-ranging study of nivolumab in participants with advanced/metastatic clear cell RCC), a dose association was observed for infusion site reactions and hypersensitivity reactions (1.7% at 0.3 mg/kg, 3.7% at 2 mg/kg and 18.5% at 10 mg/kg). All the events were Grade 1-2 and were manageable. An infusion duration of 30 minutes for 3 mg/kg of nivolumab (30% of the dose provided at 10 mg/kg) is not expected to present any safety concerns compared to the prior experience at 10 mg/kg nivolumab dose infused over a 60-minute duration.

Of note, CA209153, a Phase 3b/4 safety study of nivolumab in participants with metastatic NSCLC who have progressed during or after at least 1 prior systemic regimen, used a 30-minute infusion in a cohort of participants with no safety issues.

Overall, infusion reactions including high-grade hypersensitivity reactions have been uncommon across nivolumab. Overall, a change in safety profile is not anticipated with 30-minute infusion of nivolumab.

6. STUDY POPULATION

For entry into the study, the following criteria MUST be met.

6.1 Inclusion Criteria

1) **Signed Written Informed Consent**
   a) Participants must have signed and dated an IRB/IEC approved written informed consent form in accordance with regulatory and institutional guidelines. This must be obtained

Approved v1.0 [redacted]

before the performance of any protocol related procedures that are not part of normal participant care.

b) Participants must be willing and able to comply with scheduled visits, treatment schedule, and laboratory testing.

2) **Type of Participant and Target Disease Characteristics**

a) Eastern Cooperative Group (ECOG) Performance Status 0-1 (Appendix 3)

b) P Participants with histologically confirmed Stage IB (≥ 4 cm), II, IIIA (N2) NSCLC (per the 8th American Joint Committee on Cancer (AJCC) (Rami-Porta, 2015) with disease that is considered resectable.[49]

c) Measurable disease according to RECIST version 1.1

d) Participants must have a tumor tissue sample available for PD-L1 IHC testing performed by a third-party analyzing lab during the screening period:

i) Either a formalin-fixed, paraffin-embedded (FFPE) tissue block or unstained tumor tissue sections, with an associated pathology report, must be submitted for biomarker evaluation prior to randomization. The tumor tissue sample may be fresh or archival if obtained within 6 months prior to enrollment.

ii) Tissue must be a core needle biopsy, excisional or incisional biopsy. Fine needle biopsies obtained by EBUS is not considered adequate for biomarker review and randomization. Core needle biopsies obtained by EBUS are acceptable for randomization.

e) Absence of major associated pathologies that increase the surgery risk to an unacceptable level

f) Mediastinal lymph node samples at levels 4 (bilaterally) and 7 are required for clinical staging to assess nodal involvement in participants with mediastinal adenopathy on PET/CT. Mediastinoscopy, thoracostomy, or EBUS are all acceptable for such assessment.

g) Pulmonary function capacity capable of tolerating the proposed lung resection according to the surgeon.

3) **Age and Reproductive Status**

a) Males and Females, ages ≥18 or age of majority

b) Women of childbearing potential (WOCBP) must have a negative serum or urine pregnancy test (minimum sensitivity 25 IU/L or equivalent units of HCG) within 24 hours prior to the start of study treatment.

c) Women must not be breastfeeding

d) Women of childbearing potential (WOCBP) must agree to follow instructions for method(s) of contraception (Appendix 6) for the duration of treatment with nivolumab and 5 months after the last dose of study treatment (ie, 30 days [duration of ovulatory cycle]) [plus the time required for the investigational drug to undergo approximately 5 half-lives (for participants treated in Arm A).

e) WOCBP must also agree to follow instructions for method(s) of contraception from the time of enrollment for the duration of treatment with chemotherapy plus 5 half-lives of chemotherapy plus 30 days (duration of ovulatory cycle) for a total of 30 days

Approved v1.0 [redacted]

post-treatment completion or a duration specified by the local labels of the chemotherapy drugs received, whichever is longer (for participants treated in Arm B).

f) Males who are sexually active with WOCBP must agree to follow instructions for method(s) of contraception (Appendix 6) for the duration of treatment with nivolumab and 7 months after the last dose of study treatment (ie, 90 days [duration of sperm turnover] plus the time required for the investigational drug to undergo approximately 5 half-lives) (for participants treated in Arm A). In addition, male participants must be willing to refrain from sperm donation during this time.

g) Males who are sexually active with WOCBP must agree to follow instructions for method(s) of contraception for the duration of treatment with study treatment(s) with chemotherapy plus 5 half-lives of the study treatment plus 90 days (duration of sperm turnover) for a total of 90 days post-treatment completion or a duration specified by the local labels of the chemotherapy drugs received, whichever is longer (participants in Arm B). In addition, male participants must be willing to refrain from sperm donation during this time.

h) Azoospermic males are exempt from contraceptive requirements. WOCBP who are continuously not heterosexually active are also exempt from contraceptive requirements, and still must undergo pregnancy testing as described in this section.

Investigators shall counsel WOCBP, and male participants who are sexually active with WOCBP, on the importance of pregnancy prevention and the implications of an unexpected pregnancy. Investigators shall advise on the use of highly effective methods of contraception, (Appendix 6) which have a failure rate of < 1% when used consistently and correctly.

6.2 Exclusion Criteria

1) Medical Conditions

a) Presence of locally advanced unresectable regardless of stage or metastatic disease (stage IV). Staging assessment should include sample of lymph nodes at levels 4, bilaterally, and level 7 to rule out stage IIIB disease.

b) Participants with known EGFR mutations or ALK translocation. If testing is done, an FDA-approved assay should be used, and testing will be performed locally.

c) Participants with brain metastases are excluded from this study and all participants with stage II disease or higher should have brain imaging (either MRI brain or CT brain with contrast) 28 days prior to randomization.

d) Participants with ≥ Grade 2 peripheral neuropathy

e) Participants with an active, known or suspected autoimmune disease. Participants with type I diabetes mellitus, hypothyroidism only requiring hormone replacement, skin disorders (such as vitiligo, psoriasis, or alopecia) not requiring systemic treatment, or conditions not expected to recur in the absence of an external trigger are permitted to enroll.

f) Participants with a condition requiring systemic treatment with either corticosteroids (> 10 mg daily prednisone equivalent) or other immunosuppressive medications within 14 days of randomization. Inhaled or topical steroids, and adrenal replacement steroid

Approved v1.0 [redacted]

   doses > 10 mg daily prednisone equivalent, are permitted in the absence of active autoimmune disease

   g) Known history of positive test for human immunodeficiency virus (HIV) or known acquired immunodeficiency syndrome (AIDS). NOTE: Testing for HIV must be performed at sites where mandated locally.

   h) Participants with serious or uncontrolled medical disorders

2) Prior/Concomitant Therapy

   a) Administration of chemotherapy or any other cancer therapy in the pre-operative period.

   b) Prior therapy with an anti-PD-1, anti-PD-L1, anti-PDL-2, or anti-CTLA-4 antibody or any other antibody targeting T cell co-regulatory pathways.

3) Physical and Laboratory Test Findings

   a) Screening laboratory values must meet the following criteria (using CTCAE v4):

      i) WBC < 2000/μL

      ii) Neutrophils < 1500/μL

      iii) Platelets < $100x10^3$/μL

      iv) Hemoglobin < 9.0 g/dL

      v) Serum creatinine > 1.5 x ULN or calculated creatinine clearance (CrCl) < 50 mL/min (using the Cockcroft-Gault formula)

      Female CrCl = (140- age in years) x weight in kg x 0.85
      72 x serum creatinine in mg/ dL
      Male CrCl = (140- age in years) x weight in kg x 1.00
      72 x serum creatinine in mg/ dL

      vi) AST > 3.0 x ULN

      vii) ALT > 3.0 x ULN

      viii) Total Bilirubin> 1.5 x ULN (except participants with Gilbert Syndrome who must have a total bilirubin level of < 3.0 x ULN).

   b) Any positive test for hepatitis B virus or hepatitis C virus indicating presence of a virus, eg, Hepatitis B surface antigen (HBsAg, Australia antigen) positive or Hepatitis C antibody (anti-HCV) positive (except if HCV-RNA negative)

   c) Prior malignancy active within the previous 3 years except for locally curable cancers that have been apparently cured, such as basal or squamous cell skin cancer, superficial bladder cancer, or carcinoma in situ of the prostate, cervix, or breast.

   d) Excluding participants with serious or uncontrolled medical disorders

4) Allergies and Adverse Drug Reaction

   a) History of allergy or hypersensitivity to study drug components

5) Other Exclusion Criteria

   a) Prisoners or participants who are involuntarily incarcerated. (Note: under certain specific circumstances a person who has been imprisoned may be included or permitted to continue as a participant. Strict conditions apply and Bristol-Myers Squibb approval is required.

   b) Participants who are compulsorily detained for treatment of either a psychiatric or physical (eg, infectious disease) illness

Approved v1.0 [redacted]

Eligibility criteria for this study have been carefully considered to ensure the safety of the study participants and that the results of the study can be used. It is imperative that participants fully meet all eligibility criteria.

6.3 Lifestyle Restrictions

Not applicable. No restrictions are required

6.4 Screen Failures

Screen failures are defined as participants who consent to participate in the clinical study but are not subsequently randomized. A minimal set of screen failure information is required to ensure transparent reporting of screen failure participants, to meet the Consolidated Standards of Reporting Trials (CONSORT) publishing requirements, and to respond to queries from regulatory authorities. Minimal information includes date of consent, demography, screen failure details, eligibility criteria, and any serious AEs.

6.4.1 Retesting During Screening or Lead-In Period

Participant Re-enrollment: This study permits the re-enrollment of a participant who has discontinued the study as a pre-treatment failure (ie, participant has not been randomized / has not been treated). If re-enrolled, the participant must be re-consented. Participants are allowed to re-enroll twice. Eligibility will need to be confirmed if a participant is re-enrolled.

Retesting of laboratory parameters and/or other assessments within any single Screening or Lead-in period will be permitted up to 3 times (in addition to any parameters that require a confirmatory value).

The most current result prior to randomization is the value by which study inclusion will be assessed, as it represents the participant's most current, clinical state.

7. TREATMENT

Study treatment is defined as any investigational treatment(s), marketed product(s), placebo or medical device intended to be administered to a study participant according to the study randomization or treatment allocation

Study treatment includes both Investigational [Medicinal] Product (IP/IMP) and Non-investigational [Medicinal] Product (Non-IP/Non-IMP) and can consist of the following:

- All products, active or placebo, being tested or used as a comparator in a clinical trial.

An investigational product, also known as investigational medicinal product in some regions, is defined a pharmaceutical form of an active substance or placebo being tested or used as a reference in a clinical study, including products already with a marketing authorization but used or assembled (formulated or packaged) differently than the authorized form, or used for an unauthorized indication, or when used to gain further information about the authorized form.

Other medications used as support or escape medication for preventative, diagnostic, or therapeutic reasons, as components of the standard of care for a given diagnosis, may be considered as non-investigational products.

Approved v1.0 [redacted]

Table 7.-1: Study treatments for CA209816

Product Description / Class and Dosage Form	Potency	IP/ Non-IMP	Blinded or Open Label	Packaging / Appearance	Storage Conditions (per label)
BMS-936558-01 Solution for Injection/Nivolumab[a]	10 mg/mL	IP	Open-label	5 or 10 (100 mg) vials per carton; Clear to opalescent, colorless to pale yellow liquid. Few particulates may be present.	2 to 8° C. Protect from light and freezing
Ipilimumab Solution for Injection	5 mg/mL	IP	Open-label	4 vials/carton. Clear to opalescent, colorless to pale yellow liquid. May contain particles	2 to 8° C. Protect from light and freezing
Vinorelbine NC Concentrate for Solution for Infusion[b]	10 mg/mL	IP	Open-label	1 vial/carton. Clear, colorless to pale yellow solution.	Product should be stored as per market product conditions.
Gemcitabine Concentrate for Solution for Infusion[b]	1000 mg/vial	IP	Open-label	1 vial/carton. Clear, colorless or light straw-colored solution	Product should be stored as per market product conditions.
Docetaxel Concentrate for Solution for Infusion[b]	10 mg/mL	IP	Open-label	1 vial/carton. Pale yellow to brownish yellow solution	Product should be stored as per market product conditions.
Pemetrexed Powder for Concentrate for Solution for Infusion[b]	500 mg/vial	IP	Open-label	1 vial/carton. White to either light yellow or green-yellow lyophilised powder	Product should be stored as per market product conditions.
Cisplatin Concentrate for Solution for Infusion[b]	100 mg/vial (1 mg/mL)	IP	Open-label	4 vials per carton. Clear, colorless solution	Product should be stored as per market product conditions.
Carboplatin Solution for Injection[b]	450 mg/vial (10 mg/vial)	IP	Open-label	4 vials/carton. Clear, colorless or slightly yellow solution.	Product should be stored as per market product conditions.

[a] May be labeled as either “BMS-936558-01” or “nivolumab”

[b] These products may be obtained as local commercial product in certain countries if allowed by local regulations. In these cases, products may be a different pack size/potency than listed in the table. These products should be prepared, stored, stored and administered with the Package Insert or Summary of Product Characteristics.

7.1 Treatments Administered

The selection and timing of dose for each participant is presented in Table 7.1-1.

Table 7.1-1: Selection and Timing of Dose

Study Treatment	Unit dose strength(s)/ Dosage level(s)	Dosage formulation Frequency of Administration	Route of Administration
BMS-936558-01 Nivolumab	10 mg	3 mg/kg every 2 weeks for up to 3 cycles	IV
Ipilimumab	5 mg/mL	1 mg/kg at Cycle 1 only	IV
Vinorelbine	10 mg/mL	30 mg/m^2 on Days 1 and 8 of a 3-week cycle for up to 3 cycles[a]	IV
Gemcitabine	38 mg/mL	1250 mg/m^2 on Days 1 and 8 of a 3-week cycle for up to 3 cycles[a]	IV
Docetaxel	10 mg/mL	75 mg/m^2 on Day 1 of a 3-week cycle for up to 3 cycles[a]	IV
Pemetrexed	500 mg/vial	500 mg/m^2 on Day 1 of a 3-week cycle for up to 3 cycles[a]	IV
Cisplatin	1 mg/mL	75 mg/m^2 on Day 1 of a 3-week cycle for up to 3 cycles[a]	IV
Carboplatin	10 mg/mL	AUC 6 on Day 1 of a 3-week cycle for up to 3 cycles[a]	IV.

[a] Following definitive surgery, participants may receive up to 4 cycles of adjuvant chemotherapy with or without radiation at the discretion of the investigator.

<u>Nivolumab and Ipilimumab</u>

Participants are to receive nivolumab at a dose of 3 mg/kg as a 30-minute infusion on Day 1 of each treatment cycle every 2 weeks for 3 doses. In Cycle 1, participants will also receive a single dose of ipilimumab 1 mg/kg as a 30-minute infusion on Day 1. Participants are to begin study treatment within 7 calendar days of randomization.

Dosing calculations should be based on the body weight assessed at baseline. It is not necessary to re-calculate subsequent doses if the participant weight is within 10% of the weight used to calculate the previous dose. All doses should be rounded up or to the nearest milligram per institutional standard.

When study drugs (nivolumab and ipilimumab) are to be administered on the same day, nivolumab is to be administered first. Nivolumab infusion must be promptly followed by a saline flush to clear the line of nivolumab before starting the ipilimumab infusion. The second infusion will always be the ipilimumab study drug and will start after the infusion line has been flushed, filters

changed and participant has been observed to ensure no infusion reaction has occurred. The time in between infusions is expected to be approximately 30 minutes but may be more or less depending on the situation.

There will be no dose escalations or reductions of nivolumab or ipilimumab allowed. Participants may be dosed no less than 12 days from the previous dose. Premedications are not recommended for the first dose of nivolumab and ipilimumab.

Participants should be carefully monitored for infusion reactions during nivolumab and ipilimumab administration. If an acute infusion reaction is noted, participants should be managed according to Section 7.4.3.

Doses of nivolumab and ipilimumab may be interrupted, delayed, or discontinued depending on how well the participants tolerates the treatment. If a participant requires a dose delay of > 7 days, the dose should be skipped.

Dose delay criteria can be found in Section 7.4.2.1, and discontinuation criteria can be found in Section 8.1.1.1. Criteria to resume treatment can be found in Section 8.1.2.1

Nivolumab Injection, 100 mg/10 mL (10 mg/mL) is to be administered as an IV infusion through a 0.2-micron to 1.2-micron pore size, low-protein binding in-line filter at the protocol-specified doses. It is not to be administered as an IV push or bolus injection. Nivolumab injection can be infused undiluted (10 mg/mL) or diluted with 0.9% Sodium Chloride Injection, USP or 5% Dextrose Injection, USP to protein concentrations as low as 1 mg/mL. Instructions for dilution and infusion of nivolumab injection may be provided in the pharmacy binder or pharmacy reference sheet. Care must be taken to assure sterility of the prepared solution as the product does not contain any antimicrobial preservative or bacteriostatic agent.

Nivolumab infusions are compatible with polyvinyl chloride (PVC) or polyolefin containers and infusion sets, and glass bottles.

Ipilimumab injection can be used for IV administration without dilution after transferring to a PVC, non-PVC/non-DEHP or glass container and is stable for 24 hours at 2-8°C or room temperature/room light (RT/RL). For ipilimumab storage instructions, refer to ipilimumab Investigator's Brochure and/or pharmacy reference sheets.

Separate infusion bags and filters should be used when administering nivolumab and ipilimumab on the same day.

Ipilimumab is to be administered as a 30-minute IV infusion, using a volumetric pump with a 0.2 to 1.2 micron in-line filter at the protocol-specified dose. The drug can be diluted with 0.9% normal saline or 5% Dextrose Injection to concentrations between 1 mg/mL and 4 mg/mL. Ipilumumab is not to be administered as an IV push or bolus injections. Care must be taken to assure sterility of the prepared solutions, since the drug product does not contain any antimicrobial preservatives or bacteriostatic agents. At the end of the infusion, flush the line with a sufficient quantity of normal saline or 5% dextrose solution.

Approved v1.0 [redacted]

Vinorelbine/Cisplatin

Vinorelbine will be administered at a dose of 30 mg/m^2 as an IV push over 10 minutes on Days 1 and 8 followed by cisplatin at a dose of 75 mg/m^2 as a 120-minute IV infusion on Day 1 only, of a 3-week treatment cycle for up to 3 cycles.

Dosing calculations for vinorelbine should be based on the body surface area calculation assessed as per standard of care. The dose should remain the same if the participant's weight is within 10% of the baseline weight or prior dose weight. Doses of vinorelbine and/or cisplatin may be interrupted, delayed, reduced, or discontinued depending on how well the participant tolerates the treatment. Dose modifications for toxicity will be performed according to Section 7.4.1. Dose delay criteria can be found in Section 7.4.2.2, and discontinuation criteria can be found in Section 8.1.1.2. Criteria to resume treatment can be found in Section 8.1.2.2.

See below for the details regarding administration of cisplatin.

Docetaxel/Cisplatin

Docetaxel will be administered at a dose of 75 mg/m^2 as a 60-minute IV infusion on Day 1 followed by cisplatin at a dose of 75 mg/m^2 as a 120-minute IV infusion on Day 1 of a 3-week treatment cycle for up to 3 cycles.

Dosing calculations for docetaxel should be based on the body surface area calculation assessed as per standard of care. The dose should remain the same if the participant's weight is within 10% of the baseline weight or prior dose weight. Dose modifications for toxicity will be performed according to Section 7.4.1. Dose delay criteria can be found in Section 7.4.2.3, and dose discontinuation criteria can be found in Section 8.1.1.3. Criteria to resume treatment can be found in Section 8.1.2.3.

See below for the details regarding administration of cisplatin.

Premedications for use with docetaxel: Premedication with corticosteroids will be given to participants receiving docetaxel. The recommended premedication per the USPI and SmPC is dexamethasone 8 mg PO twice daily given one day before, on the day of, and one day after administration of chemotherapy. For institutions that have established an equivalent premedication regimen consistent with local docetaxel labeling, such premedication regimens will be permitted.

Gemcitabine/Cisplatin (Squamous histology only)

Participants will receive gemcitabine at a dose of 1250 mg/m^2 as a 30-minute IV infusion on Days 1 and 8 followed by cisplatin at a dose of 75 mg/m^2 as a 120-minute IV infusion on Day 1 of a 3-week treatment cycle for up to 3 cycles.

Dosing calculations for gemcitabine should be based on the body surface area calculation and may be capped per local standards. The dose may remain the same if the participant's weight is within 10% of the baseline weight or prior dose weight.

Doses of gemcitabine and/or cisplatin may be modified, delayed, or discontinued depending on how well the participant tolerates the treatment. Dose modifications for toxicity will be performed

according to Section 7.4.1. Dose delay criteria can be found in Section 7.4.2.2, and discontinuation criteria can be found in Section 8.1.1.2. Criteria to resume treatment can be found in Section 8.1.2.2.

See below for the details regarding administration of cisplatin.

Pemetrexed/Cisplatin (non-Squamous histology only)

Pemetrexed will be administered at a dose of 500 mg/m^2 as a 10-minute IV infusion with cisplatin at a dose of 75 mg/m^2 as a 120-minute IV infusion on Day 1 of a 3-week treatment cycle for up to 3 cycles.

Dosing calculation for pemetrexed should be based on the body surface area calculation and may be capped per local standards. The dose may remain the same if the participant's weight is within 10% of the baseline weight or prior dose weight. All doses should be rounded up or to the nearest milligram per institutional standard.

Doses of pemetrexed and/or cisplatin may be interrupted, delayed, or discontinued depending on how well the participant tolerates the treatment. Dose modifications for toxicity will be performed according to Section 7.4.1. Dose delay criteria can be found in Section 7.4.2.2, and discontinuation criteria can be found in Section 8.1.1.2.

Premedications for use with pemetrexed: Oral corticosteroid should be given according to local standards at a dose equivalent to dexamethasone 4 mg twice daily on the day prior to, the day of, and the day after the administration of pemetrexed. Oral folic acid 350 to 1,000 mcg daily should be given starting 1 week prior to the first dose of pemetrexed, with at least 5 doses of folic acid administered in the 7 days prior to the first dose. Oral folic acid should be continued daily throughout the treatment with pemetrexed and for 21 days after the last dose of pemetrexed. Intramuscular (IM) injection of vitamin B12 1000 mcg should be given approximately 1 week prior to the first dose of pemetrexed.

See below for the details regarding administration of cisplatin.

Cisplatin

Cisplatin 75 mg/m^2 will be administered to participants as the second infusion. Dosing calculations for cisplatin should be based on the body surface area calculation and may be capped per local standards. The dose may remain the same if the participant's weight is within 10% of the baseline weight or prior dose weight.

Pretreatment hydration for cisplatin can follow local standard of care or use 1 to 2 liters of fluid infused IV for 8 to 12 hours prior to cisplatin infusion is recommended. Adequate hydration and urinary output must be maintained for at least 24 hours following cisplatin administration. Administration and monitoring should be performed according to local standards. Use of mannitol following the cisplatin infusion should also follow local standards of care.

All participants who will be receiving cisplatin should have audiometric testing performed prior to initiation of therapy and prior to subsequent doses of cisplatin, as per local or institutional standards of care.

Approved v1.0 [redacted]

For participants who are unable to tolerate cisplatin, the reasons for intolerability should be documented. If the investigator would like to use a non-cisplatin-containing regimen, the investigator should discuss this with and obtain approval from the Medical Monitor prior to use of a non-protocol regimen.

For All Arms of Chemotherapy:

Participants should begin study treatment within 7 calendar days of randomization. Doses of chemotherapy may be interrupted, delayed, or discontinued depending on how well the participants tolerates the treatment. If a dose is delayed for any reason, participants should be dosed no later than 7 days following a planned dose on any arm.

If more than 7 days delay is needed for any reason, the intended dose should be skipped. If a participant receiving chemotherapy on a Day 1 and Day 8 schedule (ie, cisplatin/gemcitabine) is unable to receive Day 1 of chemotherapy but recovers in time to receive the Day 8 dose, the Day 8 dose of chemotherapy may be administered.

Premedications: Antiemetic premedication will be administered according to local standards. Recommended antiemetic treatments are dexamethasone (dosing according to local standards; an equivalent dose of another corticosteroid may be substituted) and a 5-HT3 receptor antagonist (type per investigator discretion and local standards-of-care). Additional use of antiemetic premedications may be employed at the discretion of the Investigator.

7.2 Method of Treatment Assignment

CA209816 is an open-label, randomized trial. Participants with Stage IB (≥ 4 cm), II and IIIA (N2) considered resectable will be eligible to participate. After the participant's initial eligibility is established and informed consent has been obtained, the participant must be enrolled into the study by calling the IRT to obtain a participant number. Every participant that signs the informed consent form must be assigned a participant number in IRT. Specific instructions for using IRT will be provided to the investigational site in a separate document. The investigator or designee will register the participant for enrollment by following the enrollment procedures established by BMS.

The following information is required for enrollment:

- Date that informed consent was obtained
- Date of birth, where applicable by local regulations
- Gender at birth

Once enrolled in IRT, enrolled participants who have met all eligibility criteria will be ready to be randomized through IRT. PD-L1 expression data will be transferred directly from analyzing lab to IRT. The following information is required for participant randomization:

- Participant number
- Date of birth, where applicable per local regulations
- Stage of disease (IB, IIA, IIB or IIIA)
- PD-L1 status
- Gender

Participants meeting all eligibility criteria will be stratified according to PD-L1 status into 2 categories (≥1% and < 1% or not evaluable/indeterminate). Enrollment of participants with not evaluable or indeterminate PD-L1 status will be capped at 10%. Participants will also be stratified based disease stage (IB/II vs IIIA) and gender.

The exact procedures for using the IRT will be detailed in the IRT manual.

7.3 Blinding

This is an open-label study; blinding procedures between participants and investigators are not applicable. The BIPR and BICR will be blinded.

7.4 Dosage Modification

7.4.1 Dose Reductions for Platinum Doublet Chemotherapy

Dose reductions of platinum doublet chemotherapy may be required and will be performed according to Table 7.4.1-1. Chemotherapy dose reductions are permanent; once the dose of any chemotherapy agent is reduced, it may not be re-escalated in subsequent cycles. The dose reductions for each agent in the platinum doublet chemotherapy regimen are not linked and may be adjusted independently as summarized below.

Table 7.4.1-1: Dose Modifications of Chemotherapeutic Agents

Dose Level	Vinorelbine	Docetaxel	Gemcitabine	Pemetrexed	Cisplatin	Carboplatin
Starting dose	30 mg/m^2	75 mg/m^2	1250 mg/m^2 (with cisplatin) or 1000 mg/m^2 (with carboplatin)	500 mg/m^2	75 mg/m^2	AUC 6
First dose reduction	22.5 mg/m^2	55 mg/m^2	900 mg/m^2 (with cisplatin) or 750 mg/m^2 (with carboplatin)	375 mg/m^2	56 mg/m^2	AUC 5
Second dose reduction	15 mg/m^2	37.5 mg/m^2	600 mg/m^2 (with cisplatin) or 500 mg/m^2 (with carboplatin)	250 mg/m^2	38 mg/m^2	AUC 4
Third dose reduction	Discontinue	Discontinue	Discontinue	Discontinue	Discontinue	Discontinue

Any participants with 2 prior dose reductions for 1 agent who experiences a toxicity that would cause a third dose reduction must be discontinued from that agent.

7.4.1.1 Platinum Doublet Chemotherapy - Dose Reductions for Hematologic Toxicity

Dose modifications for hematologic toxicities (according to CTCAE version 4) are summarized in Table 7.4.1.1-1. Dose adjustments are based on nadir blood counts (assessed as per local standards)

since the preceding drug administration. Dose level adjustments for platinum doublet chemotherapy are relative to that of the preceding administration. Generally, both chemotherapy agents in the platinum doublet chemotherapy regimen should be dose reduced together for hematologic toxicity. After the first cycle, growth factors may be used to assist hematologic recovery. Use local standards of care in the use of these supportive measures. Additionally, prophylactic antibiotics may be used according to local standards of care. Please report any antibiotic or growth factor use on the eCRF.

Approved v1.0 [redacted]

Table 7.4.1.1-1: Dose Modifications for Hematologic Toxicity (Based on Nadir Counts)

Toxicity	Vinorelbine	Gemcitabine	Pemetrexed	Cisplatin	Carboplatin
**Neutrophil Count Decreased**					
Grade 4 (< 500/mm^3 or < 0.5 x 10^9/L)	Reduce one dose level and consider prophylactic G-CSF in subsequent cycles	Reduce one dose level and consider prophylactic G-CSF in subsequent cycles	Reduce one dose level and consider prophylactic G-CSF in subsequent cycles	Reduce one dose level and consider prophylactic G-CSF in subsequent cycles	Reduce one dose level and consider prophylactic G-CSF in subsequent cycles
**Platelet Count Decreased**					
Grade 3 (25,000 to < 50,000/mm^3; 25.0 to < 50.0 x 10^9/L)	Reduce one dose level	Reduce one dose level	Reduce one dose level	Reduce one dose level	Reduce one dose level
Grade 4 (< 25,000/mm^3; < 25.0 x 10^9/L)	Reduce one dose level	Reduce one dose level	Reduce one dose level	Reduce one dose level	Reduce one dose level
**Anemia**					
Grade 2 (< 10.0 to 8.0 g/dL; < 6.2 to 4.9 mmol/L; < 100 - 80 g/L)	Reduce one dose level	Reduce one dose level	Reduce one dose level	Reduce one dose level	Reduce one dose level
Grade 3 (< 8.0 g/dL; < 4.9 mmol/L, < 80 g/L)	Reduce one dose level	Reduce one dose level	Reduce one dose level	Reduce one dose level	Reduce one dose level
Grade 4 (Life threatening consequences)	Hold drug	Hold drug	Hold drug	Hold Drug	Hold Drug

Doses of docetaxel will be modified for participants who experience docetaxel-related events of febrile neutropenia, neutrophils < 500 cell/mm^3 for > 7 days, severe or cumulative cutaneous reactions, or other Grade 3/4 non-hematological toxicities during docetaxel treatment. Participants should have treatment delayed according to Section 7.4.2.3 and then resumed at 1 dose level reduction (55 mg/m^2). Should these AEs occur after the first dose reduction, then a second dose reduction to 37.5 mg/m^2 is permitted. If a third dose reduction is required, then the participants should discontinue docetaxel treatment.

7.4.1.2 Platinum Doublet Chemotherapy - Dose Reductions for Non-Hematologic Toxicities

Dose adjustments for platinum doublet chemotherapy for non-hematologic toxicities during treatment are described in Table 7.4.1.2-1. All dose reductions should be made based on the worst grade toxicity. Participants experiencing any of the toxicities detailed in Table 7.4.1.2-1 during the previous cycle should have their chemotherapy delayed until retreatment criteria are met (per Section 8.1.2.2) and then reduced for all subsequent cycles by 1 dose level or discontinued as appropriate. Dose levels for the 2 drugs in the platinum-doublet chemotherapy regimen are not linked and may be reduced independently, as summarized in Table 7.4.1.2-1.

Approved v1.0 [redacted]

Table 7.4.1.2-1: Dose Modifications for Non-hematologic Toxicity

Toxicity	Vinorelbine	Gemcitabine	Pemetrexed	Cisplatin	Carboplatin
**Febrile Neutropenia** Grade ≥ 3	Reduce one dose level	Reduce one dose level	Reduce one dose level	Reduce one dose level	Reduce one dose level
**Diarrhea** Grade ≥ 3	Reduce one dose level	Reduce one dose level	Reduce one dose level	No change	No change
**Allergic reaction**[a] Grade ≥ 3	Reduce one dose level	Discontinue	Discontinue	Discontinue	Discontinue
**Neuropathy** Grade 2	No Change	No change	No change	Reduce one dose level	No change
**Neuropathy** Grade ≥ 3	Discontinue	Discontinue	Discontinue	Discontinue	Discontinue
**Calculated creatinine clearance < 50 mL/min**	No Change	No change	No change	Discontinue	Discontinue if creatinine clearance < 20 mL/min
**Other Grade ≥ 3** toxicity (except for fatigue and transient arthralgia and myalgia)	Adjust as medically indicated	Adjust as medically indicated	Adjust as medically indicated	Adjust as medically indicated	Adjust as medically indicated

[a] Only the drug(s) causing the hypersensitivity reaction or acute infusion reaction (≥ Grade 3) require(s) discontinuation. All other drugs may be continued.

Participants receiving docetaxel who develop Grade ≥ 3 peripheral neuropathy, or who otherwise meet criteria specified in Section 8.1.1.3, should discontinue docetaxel treatment.

7.4.2 Dose Delay

If any agent is delayed > 7 days, the dose should be skipped, and the participant should resume treatment at the next scheduled dose if criteria to resume treatment (Section 8.1.2) for the appropriate arm are met. If the last dose of study drug is delayed >7 but < 11 days, while it is recommended that this dose be skipped, if the investigator feels that it is appropriate to administer the last dose, discuss with the Medical Monitor before administering the last dose of study drug.

7.4.2.1 Nivolumab Dose Delay Criteria

Nivolumab administration should be delayed for the following:

- Grade 2 non-skin, drug-related adverse event, with the exception of fatigue
- Grade 2 drug-related creatinine, AST, ALT and/or Total Bilirubin abnormalities
- Grade 3 skin, drug-related adverse event

- Grade 3 drug-related laboratory abnormality, with the following exceptions:
  - Grade 3 lymphopenia or asymptomatic amylase or lipase does not require dose delay
  - Grade ≥ 3 AST, ALT, Total Bilirubin will require dose discontinuation (see Section 8.1.1.1)
- Any adverse event, laboratory abnormality, or intercurrent illness which, in the judgment of the investigator, warrants delaying the dose of study medication.

Participants who require delay of nivolumab should be re-evaluated weekly or more frequently if clinically indicated and resume nivolumab dosing when re-treatment criteria are met.

7.4.2.2 Dose Delay Criteria for Platinum Doublet Chemotherapy

Dosing of both drugs in the platinum doublet chemotherapy regimen selected should be delayed for any of the following on the Day 1 of each cycle:

- Absolute neutrophil count (ANC) < 1500/µL
- Platelets < 100,000/mm^3
- Any Grade ≥ 2 non-skin, non-hematologic, drug-related adverse event (excluding Grade 2 alopecia, Grade 2 fatigue, and Grade 2 laboratory abnormalities)
- Any Grade ≥ 3 skin, drug-related adverse event
- Any Grade ≥ 3 drug-related laboratory abnormality, with the following exceptions for lymphopenia, AST, ALT, or total bilirubin:
  - Grade 3 lymphopenia does not require dose delay.
  - If a participant has a baseline AST, ALT or total bilirubin that is within normal limits, delay dosing for drug-related Grade ≥ 2 toxicity.
  - If a participant has baseline AST, ALT, or total bilirubin within the Grade 1 toxicity range, delay dosing for drug-related Grade ≥ 3 toxicity.
- Any adverse event, laboratory abnormality, or intercurrent illness which, in the judgment of the investigator, warrants delaying the dose of study medication. Investigators should consult local labeling for the chemotherapy drugs being administered to any given participant for additional guidance on dose delays.

In addition, participants receiving cisplatin with pemetrexed must discontinue cisplatin if the calculated creatinine clearance decreases to < 50 mL/min (based on the Cockroft Gault formula).

If any non-hematologic adverse event meeting the dose delay criteria above is felt to be related to only 1 particular agent in the platinum doublet chemotherapy regimen, then that agent alone may be omitted for that cycle while the other agent is given. In order to maintain synchronized dosing of the regimen, the omitted agent should be resumed with the next scheduled cycle once the AE has improved and retreatment criteria are met. Please refer to Section 7.4.1 to determine if dose reduction of the resumed agent is required.

Approved v1.0 [redacted]

7.4.2.3 Docetaxel Dose Delay Criteria

Docetaxel administration should be delayed for the following:

- Any Grade ≥ 2 non-skin, drug-related adverse event, with the following exceptions:
  - Grade 2 drug-related fatigue or laboratory abnormalities do not require a treatment delay
- Any Grade 3 skin, drug-related adverse event
- Any Grade 3 drug-related laboratory abnormality, with the following exceptions for lymphopenia, neutrophil count, AST, ALT, or total bilirubin:
  - Grade 3 lymphopenia does not require dose delay
  - Should not be given if neutrophil counts are < 1500 cells/mm^3
  - Should not be given if total bilirubin > upper limit of normal (ULN), or if AST and/or ALT > 1.5 x ULN concomitant with alkaline phosphatase > 2.5 x ULN
- Any AE, laboratory abnormality or inter-current illness which, in the judgment of the investigator, warrants delaying the dose of study medication

Subsequent dose reductions may be required as per Section 7.4.1.

Participants receiving docetaxel may receive growth factors (including G-CSF and erythropoietin) at the discretion of the investigator.

7.4.3 Treatment of Nivolumab- and Ipilimumab-related Infusion Reactions

Since nivolumab and ipilimumab contain only human immunoglobulin protein sequences, they are unlikely to be immunogenic and induce infusion or hypersensitivity reactions. However, if such a reaction were to occur, it might manifest with fever, chills, rigors, headache, rash, pruritus, arthralgias, hypotension, hypertension, bronchospasm, or other allergic-like reactions. All Grade 3 or 4 infusion reactions should be reported within 24 hours to the study Medical Monitor and reported as an SAE if it meets the criteria. Infusion reactions should be graded according to NCI CTCAE (Version 4) guidelines.

Treatment recommendations are provided below and may be modified based on local treatment standards and guidelines, as appropriate:

For Grade 1 symptoms: (mild reaction; infusion interruption not indicated; intervention not indicated):

- Remain at bedside and monitor participant until recovery from symptoms. The following prophylactic premedications are recommended for future infusions: diphenhydramine 50 mg (or equivalent) and/or acetaminophen/paracetamol 325 to 1000 mg at least 30 minutes before additional nivolumab administrations.

For Grade 2 symptoms: (moderate reaction required therapy or infusion interruption but responds promptly to symptomatic treatment (eg, antihistamines, non-steroidal anti-inflammatory drugs,

Approved v1.0 [redacted]

narcotics, corticosteroids, bronchodilators, IV fluids); prophylactic medications indicated for ≤ 24 hours):

- Stop the nivolumab/ipilimumab infusion, begin an IV infusion of normal saline, and treat the participant with diphenhydramine 50 mg IV (or equivalent) and/or acetaminophen/paracetamol 325 to 1000 mg; remain at bedside and monitor participant until resolution of symptoms. Corticosteroid and/or bronchodilator therapy may also be administered as appropriate. If the infusion is interrupted, then restart the infusion at 50% of the original infusion rate when symptoms resolve; if no further complications ensue after 30 minutes, the rate may be increased to 100% of the original infusion rate. Monitor participant closely. If symptoms recur, then no further nivolumab will be administered at that visit.
- For future infusions, the following prophylactic premedications are recommended: diphenhydramine 50 mg (or equivalent) and/or acetaminophen/paracetamol 325 to 1000 mg should be administered at least 30 minutes before nivolumab infusions. If necessary, corticosteroids (up to 25 mg of hydrocortisone or equivalent) may be used.

For Grade 3 or 4 symptoms: (severe reaction, Grade 3: prolonged [ie, not rapidly responsive to symptomatic medication and/or brief interruption of infusion]; recurrence of symptoms following initial improvement; hospitalization indicated for other clinical sequelae (eg, renal impairment, pulmonary infiltrates). Grade 4: Life-threatening; pressor or ventilatory support indicated):

- Immediately discontinue infusion of nivolumab/ipilimumab. Begin an IV infusion of normal saline and treat the participant as follows: Recommend bronchodilators, epinephrine 0.2 to 1 mg of a 1:1000 solution for subcutaneous administration or 0.1 to 0.25 mg of a 1:10,000 solution injected slowly for IV administration, and/or diphenhydramine 50 mg IV with methylprednisolone 100 mg IV (or equivalent), as needed. Participant should be monitored until the Investigator is comfortable that the symptoms will not recur. Nivolumab will be permanently discontinued. Investigators should follow their institutional guidelines for the treatment of anaphylaxis. Remain at bedside and monitor participant until recovery of the symptoms.

In case of late-occurring hypersensitivity symptoms (eg, appearance of a localized or generalized pruritus within 1 week after treatment), symptomatic treatment may be given (eg, oral antihistamine or corticosteroids).

7.5 Preparation/Handling/Storage/Accountability

The investigational product should be stored in a secure area according to local regulations. It is the responsibility of the investigator to ensure that investigational product is only dispensed to study participants. The investigational product must be dispensed only from official study sites by authorized personnel according to local regulations.

The product storage manager should ensure that the study treatment is stored in accordance with the environmental conditions (temperature, light, and humidity) as determined by BMS. If concerns regarding the quality or appearance of the study treatment arise, the study treatment should not be dispensed and contact BMS immediately.

Approved v1.0 [redacted]

Study treatment not supplied by BMS will be stored in accordance with the package insert.

Investigational product documentation (whether supplied by BMS or not) must be maintained that includes all processes required to ensure drug is accurately administered. This includes documentation of drug storage, administration and, as applicable, storage temperatures, reconstitution, and use of required processes (e.g., required diluents, administration sets).

Handling of chemotherapy should be according to local prescribing information.

Further guidance and information for final disposition of unused study treatment are provided in Appendix 7 and the Pharmacy Manual.

7.5.1 *Retained Samples for Bioavailability / Bioequivalence*

At the time of receipt of the investigational product by the investigator or designee's, BMS will specify the appropriate number of containers or units to select for retention, the conditions of sample storage, required duration of sample retention, and provisions for returning or disposing of the investigational product. When samples are selected, containers or units should be placed in packaging with a tamper evident seal provided by BMS. Package labeling should clearly identify the contents as bioavailability/bioequivalence (BA/BE) samples and state that the investigational product should be stored in the restricted area with limited access.

7.6 Treatment Compliance

Treatment compliance will be monitored by drug accountability as well as the participant's medical record and eCRF.

7.7 Concomitant Therapy

7.7.1 *Prohibited and/or Restricted Treatments*

The following medications are prohibited during the study (unless utilized to treat a drug related adverse event):

- Immunosuppressive agents
- Immunosuppressive doses of systemic corticosteroids (except as stated in Section 7.7.3)
- Any concurrent anti-neoplastic therapy (ie, chemotherapy, hormonal therapy, immunotherapy, extensive, non-palliative radiation therapy, or standard or investigational agents for treatment of NSCLC)
- Investigators should refer to the local product labeling for the chemotherapy drugs selected for use in Arms B and for additional prohibited and restricted concomitant medications.
- Caution should be used regarding the use of herbal medication as there may be as yet unknown interactions with nivolumab and ipilimumab. Discontinuation of the use of herbal medications prior to the study enrollment is encouraged.

7.7.2 *Other Restrictions and Precautions*

Participants with a condition requiring systemic treatment with either corticosteroids (> 10 mg daily prednisone equivalent) or other immunosuppressive medications within 14 days of randomization are excluded. Inhaled or topical steroids, and adrenal replacement steroid doses > 10 mg daily prednisone equivalent, are permitted in the absence of active autoimmune disease.

Approved v1.0 [redacted]

It is the local imaging facility's responsibility to determine, based on participants attributes (eg, allergy history, diabetic history and renal status), the appropriate imaging modality and contrast regimen for each participant. Imaging contraindications and contrast risks should be considered in this assessment. Participants with renal insufficiency should be assessed as to whether or not they should receive contrast and if so, what type and dose of contrast is appropriate. Specific to MRI, participants with severe renal insufficiency (ie, estimated glomerular filtration rate [eGFR] < 30 mL/min/1.73 m^2) are at increased risk of nephrogenic systemic fibrosis. MRI contrast should not be given to this population. In addition, participants are excluded from MRI if they have tattoos, metallic implants, pacemakers, etc. The ultimate decision to perform MRI in an individual participant in this study rests with the site radiologist, the investigator, and the standard set by the local Ethics Committee.

7.7.3 Permitted Therapy

Participants are permitted the use of topical, ocular, intra-articular, intranasal, and inhalational corticosteroids (with minimal systemic absorption). Adrenal replacement steroid doses > 10 mg daily prednisone are permitted. A brief (less than 3 weeks) course of corticosteroids for prophylaxis (eg, contrast dye allergy) or for treatment of non-autoimmune conditions (eg, delayed-type hypersensitivity reaction caused by a contact allergen) is permitted.

7.8 Treatment After the End of the Study

At the end of the study/Period, BMS will not continue to provide BMS supplied study treatment to participants/investigators unless BMS chooses to extend the study. The investigator should ensure that the participant receives appropriate standard of care lung cancer treatment.

8. DISCONTINUATION CRITERIA

8.1 Discontinuation from Study Treatment

Participants MUST discontinue investigational product (and non-investigational product at the discretion of the investigator) for any of the following reasons:

- Participant's request to stop study treatment. Participants who request to discontinue study treatment will remain in the study and must continue to be followed for protocol specified follow-up procedures. The only exception to this is when a participant specifically withdraws consent for any further contact with him/her or persons previously authorized by participant to provide this information. Participant should notify the investigator of the decision to withdraw from future follow-up in writing, whenever possible, and this should be documented in the medical records.
- Any clinical adverse event (AE), laboratory abnormality or intercurrent illness which, in the opinion of the investigator, indicates that continued participation in the study is not in the best interest of the participant
- Termination of the study by Bristol-Myers Squibb (BMS)
- Loss of ability to freely provide consent through imprisonment or involuntarily incarceration for treatment of either a psychiatric or physical (e.g., infectious disease) illness

Approved v1.0 [redacted]

Refer to the Schedule of Activities for data to be collected at the time of treatment discontinuation and follow-up and for any further evaluations that can be completed

In the case of pregnancy, the investigator must immediately notify the BMS Medical Monitor/designee of this event. In the event a normal healthy female participant becomes pregnant during a clinical trial, the study treatment must be discontinued immediately. In most cases, the study treatment will be permanently discontinued in an appropriate manner (e.g., dose tapering if necessary for participant safety). Please call the BMS Medical Monitor within 24 hours of awareness of the pregnancy. If the investigator determines a possible favorable benefit/risk ratio that warrants continuation of study treatment, a discussion between the investigator and the BMS Medical Monitor/designee must occur.

All participants who discontinue study treatment should comply with protocol specified follow-up procedures as outlined in Section 2. The only exception to this requirement is when a participant withdraws consent for all study procedures including post-treatment study follow-up or loses the ability to consent freely (ie, is imprisoned or involuntarily incarcerated for the treatment of either a psychiatric or physical illness).

If study treatment is discontinued prior to the participant's completion of the study, the reason for the discontinuation must be documented in the participant's medical records and entered on the appropriate case report form (CRF) page.

8.1.1 Study Treatment Discontinuation Criteria

Doses that are missed or delayed by > 7 days will not be replaced, and participants should proceed to surgery within the indicated timelines.

8.1.1.1 Nivolumab Discontinuation Criteria

Nivolumab treatment should be permanently discontinued for the following:

- Any Grade 2 drug-related uveitis, eye pain or blurred vision that does not respond to topical therapy and does not improve to Grade 1 severity within the re-treatment period OR requires systemic treatment
- Any Grade 3 non-skin, drug-related adverse event lasting > 7 days, or recurs with the following exceptions for laboratory abnormalities, diarrhea, colitis, neurologic toxicity, drug-related uveitis, pneumonitis, bronchospasm, hypersensitivity reactions, infusion reactions, and endocrinopathies:
  - Grade 3 drug-related diarrhea, colitis, neurologic toxicity, uveitis, pneumonitis, bronchospasm, hypersensitivity reaction, or infusion reaction of any duration requires discontinuation
  - Grade 3 drug-related endocrinopathies, adequately controlled with only physiologic hormone replacement do not require discontinuation. Adrenal insufficiency requires discontinuation regardless of control with hormone replacement.

- Grade 3 drug-related laboratory abnormalities do not require treatment discontinuation except:
  - ♦ Grade 3 drug-related thrombocytopenia > 7 days or associated with bleeding requires discontinuation
  - ♦ Any drug-related liver function test (LFT) abnormality that meets the following criteria require discontinuation:
    - o Grade ≥ 3 drug-related AST, ALT or Total Bilirubin requires discontinuation*

      * In most cases of Grade 3 AST or ALT elevation, study drug(s) will be permanently discontinued. If the investigator determines a possible favorable benefit/risk ratio that warrants continuation of study drug(s), a discussion between the investigator and the BMS Medical Monitor/designee must occur.
    - o Concurrent AST or ALT > 3 x ULN and total bilirubin > 2x ULN

- Any Grade 4 drug-related adverse event or laboratory abnormality (including but not limited to creatinine, AST, ALT, or Total Bilirubin), except for the following events which do not require discontinuation:
  - Grade 4 neutropenia ≤ 7 days
  - Grade 4 lymphopenia or leukopenia or asymptomatic amylase or lipase
  - Isolated Grade 4 electrolyte imbalances/abnormalities that are not associated with clinical sequelae and are corrected with supplementation/appropriate management within 72 hours of their onset
  - Grade 4 drug-related endocrinopathy adverse events, such as, hyper- or hypothyroidism, or glucose intolerance, which resolve or are adequately controlled with physiologic hormone replacement (corticosteroids, thyroid hormones) or glucose-controlling agents, respectively, may not require discontinuation after discussion with and approval from the BMS Medical Monitor.
- Any event that leads to delay in dosing of any study drug for > 6 weeks from the previous dose requires discontinuation of that drug(s) with the following exception:
  - Dosing delays lasting > 6 weeks from the previous dose that occur for non-drug-related reasons may be allowed if approved by the BMS medical monitor. Prior to re-initiating treatment in a participant with a dosing delay lasting > 6 weeks, the BMS Medical Monitor must be consulted. Periodic study visits to assess safety and laboratory studies should also continue every 6 weeks or more frequently if clinically indicated during such dosing delays.
- Any adverse event, laboratory abnormality, or intercurrent illness which, in the judgment of the Investigator, presents a substantial clinical risk to the participant with continued nivolumab dosing.

8.1.1.2 Platinum Doublet Chemotherapy Dose Discontinuation

Except where specified below, both chemotherapy drugs in the platinum doublet chemotherapy regimen should be discontinued for any of the following:

- Any Grade ≥ 3 peripheral neuropathy
- Grade ≥ 3 drug-related thrombocytopenia associated with clinically significant bleeding

Approved v1.0 [redacted]

- Any drug-related liver function test (LFT) abnormality that meets the following criteria requires discontinuation:
  - AST or ALT > 5-10x ULN for > 2 weeks
  - AST or ALT > 10x ULN
  - Total bilirubin > 5 x ULN
  - Concurrent AST or ALT > 3 x ULN and total bilirubin > 2 x ULN
- Any cisplatin-related decrease in creatinine clearance to < 50 mL/min (using the Cockroft Gault formula) requires discontinuation of cisplatin.
- Any drug-related adverse event which recurs after 2 prior dose reductions for the same drug-related adverse event (as specified in Sections 7.4.1.1 and 7.4.1.2) requires discontinuation of the drug(s) which was/were previously dose reduced.
- Any Grade ≥ 3 drug-related hypersensitivity reaction or infusion reaction requires discontinuation of the drug(s) felt to be causing the reaction. The drug not felt to be related to the hypersensitivity reaction or infusion reaction may be continued.
- Any Grade 4 drug-related adverse event which the investigator deems is inappropriate to be managed by dose reduction(s) requires discontinuation of the drug(s) felt to be causing the event. The drug not felt to be related to the event may be continued.
- Any event that leads to delay in dosing of any study drug(s) for > 6 weeks from the previous dose requires discontinuation of that drug(s) with the following exception:
  - Dosing delays lasting > 6 weeks from the previous dose that occur for non-drug-related reasons may be allowed if approved by the BMS medical monitor. Prior to re-initiating treatment in a participant with a dosing delay lasting > 6 weeks, the BMS Medical Monitor must be consulted. Periodic study visits to assess safety and laboratory studies should also continue every 6 weeks or more frequently if clinically indicated during such dosing delays.
- Any adverse event, laboratory abnormality, or intercurrent illness which, in the judgment of the Investigator, presents a substantial clinical risk to the participant with continued platinum doublet chemotherapy dosing. Investigators should consult local labeling for the chemotherapy drugs being administered to any given participant for additional guidance on dose discontinuation.

8.1.1.3 Docetaxel Dose Discontinuation

Docetaxel treatment should be permanently discontinued for the following:

- Any ≥ Grade 3 peripheral neuropathy
- Any Grade 3 non-skin, drug-related adverse event lasting > 7 days, with the following exceptions for laboratory abnormalities:
  - Grade 3 drug-related laboratory abnormalities do not require treatment discontinuation except
    - ♦ Grade 3 drug-related thrombocytopenia associated with bleeding requires discontinuation.

Approved v1.0 [redacted]

- ♦ Any drug-related liver function test (LFT) abnormalities that meets the following criteria require discontinuation:
  - AST or ALT > 5 - 10x ULN for > 2 weeks
  - AST or ALT > 10x ULN
  - Total bilirubin > 5x ULN
  - Concurrent AST or ALT > 3x ULN and total bilirubin > 2x ULN
- Any Grade 4 drug-related adverse event including laboratory abnormalities except for the following events which do not require discontinuation:
  - Grade 4 neutropenia >7 days despite 2 prior docetaxel reductions requires discontinuation
  - Grade 4 lymphopenia or leukopenia does not require discontinuation
  - Isolated Grade 4 electrolyte imbalances/abnormalities that are not associated with clinical sequelae and are corrected with supplementation/appropriate management within 72 hours of their onset does not require discontinuation.
- Any dosing interruption lasting > 6 weeks with the following exceptions:
  - Dosing interruptions > 6 weeks that occur for non-drug-related reasons may be allowed if approved by the BMS Medical Monitor/Study Director. Prior to re-initiating treatment in a participant with a dosing interruption lasting > 6 weeks, the BMS Medical Monitor/Study Director must be consulted. Tumor assessments should continue as per protocol even if dosing is interrupted.

8.1.2 Criteria to Resume Treatment

8.1.2.1 Criteria to Resume Nivolumab Treatment

Participants may resume treatment with study drug when the drug-related AE(s) resolve to Grade ≤ 1 or baseline value, with the following exceptions:

- Participants may resume treatment in the presence of Grade 2 fatigue
- Participants who have not experienced a Grade 3 drug-related skin AE may resume treatment in the presence of Grade 2 skin toxicity
- For participants with Grade 2 AST, ALT and/or Total Bilirubin Abnormalities, dosing may resume when laboratory values return to baseline and management with corticosteroids, if needed, is complete.
- Participants with combined Grade 2 AST/ALT AND total bilirubin values meeting discontinuation parameters (Section 8.1.1.1) should have treatment permanently discontinued.
- Drug-related pulmonary toxicity, diarrhea or colitis must have resolved to baseline before treatment is resumed. Participants with persistent Grade 1 pneumonitis after completion of a steroid taper over at least 1 month may be eligible for retreatment if discussed with and approved by BMS Medical Monitor.

Participants with drug-related endocrinopathies adequately controlled with only physiologic hormone replacement may resume treatment after consultation with the BMS Medical Monitor.

Approved v1.0 [redacted]

Grade 4 adrenal insufficiency requires discontinuation regardless of control with hormone replacement.

8.1.2.2 Criteria to Resume Platinum Doublet Chemotherapy Dosing

- Participants may resume treatment with platinum doublet chemotherapy when the ANC returns to ≥ 1500/µL, the platelet count returns to ≥ 100,000/mm^3, and all other drug-related toxicities have returned to baseline or Grade ≤ 1 (or Grade ≤ 2 for alopecia and fatigue)
- If a participant fails to meet criteria for reinitiating treatment, then treatment should be delayed, and the participant should be re-evaluated weekly or more frequently as clinically indicated
- When resuming platinum doublet chemotherapy treatment, please follow the dose reduction recommendations in Section 7.4.1

8.1.2.3 Criteria to Resume Treatment with Docetaxel

Participants may resume treatment with docetaxel when the drug-related AE(s) resolve(s) to Grade ≤ 1 or baseline, with the following exceptions:

- Participants may resume treatment in the presence of Grade 2 fatigue
- Participants who have not experienced a Grade 3 drug-related skin AE may resume treatment in the presence of Grade 2 skin toxicity
- Participants with decreased neutrophil counts, or with elevations in total bilirubin, AST or ALT must meet criteria for resuming treatment according to the boxed warning contained within the docetaxel Prescribing Information
- Participants with combined Grade 2 AST/ALT AND total bilirubin values meeting discontinuation parameters (Section 8.1.1.3) should have treatment permanently discontinued

When resuming docetaxel treatment, please follow the dose reduction recommendations noted in Section 7.4.1.

8.1.3 Post Study Treatment Study Follow-up

In this study, MPR is a key endpoint of the study, and EFS is a key secondary endpoint. Post study follow-up is of critical importance and is essential to preserving participant safety and the integrity of the study. Participants who discontinue study treatment must continue to be followed for collection of outcome and/or survival follow-up data as required and in line with Section 2 until death or the conclusion of the study.

BMS may request that survival data be collected on all treated/randomized participants outside of the protocol defined window (Table 2.-3 and Table 2.-4). At the time of this request, each participant will be contacted to determine their survival status unless the participant has withdrawn consent for all contacts or is lost to follow-up.

Approved v1.0 [redacted]

8.2 Discontinuation from the Study

Participants who request to discontinue study participation will remain in the study and must continue to be followed for protocol specified follow-up procedures. The only exception to this is when a participant specifically withdraws consent for any further contact with him/her or persons previously authorized by participant to provide this information.

- Participants should notify the investigator of the decision to withdraw consent from future follow-up **in writing**, whenever possible.
- The withdrawal of consent should be explained in detail in the medical records by the investigator, as to whether the withdrawal is from further treatment with study treatment only or also from study procedures and/or post treatment study follow-up, and entered on the appropriate CRF page.
- In the event that vital status (whether the participant is alive or dead) is being measured, publicly available information should be used to determine vital status only as appropriately directed in accordance with local law.
- If the participant withdraws consent for disclosure of future information, the sponsor may retain and continue to use any data collected before such a withdrawal of consent.

8.3 Lost to Follow-Up

- All reasonable efforts must be made to locate participants to determine and report their ongoing status. This includes follow-up with persons authorized by the participant.
- Lost to follow-up is defined by the inability to reach the participant after a minimum of **three** documented phone calls, faxes, or emails as well as lack of response by participant to one registered mail letter. All attempts should be documented in the participant's medical records.
- If it is determined that the participant has died, the site will use permissible local methods to obtain date and cause of death.
- If investigator's use of third-party representative to assist in the follow-up portion of the study has been included in the participant's informed consent, then the investigator may use a Sponsor retained third-party representative to assist site staff with obtaining participant's contact information or other public vital status data necessary to complete the follow-up portion of the study.
- The site staff and representative will consult publicly available sources, such as public health registries and databases, in order to obtain updated contact information.
- If after all attempts, the participant remains lost to follow-up, then the last known alive date as determined by the investigator should be reported and documented in the participant's medical records.

9. STUDY ASSESSMENTS AND PROCEDURES

- Study procedures and timing are summarized in the Schedule of Activities.
- Protocol waivers or exemptions are not allowed.
- All immediate safety concerns must be discussed with the Sponsor immediately upon occurrence or awareness to determine if the participant should continue or discontinue treatment.

Approved v1.0 [redacted]

- Adherence to the study design requirements, including those specified in the Schedule of Activities, is essential and required for study conduct.
- All screening evaluations must be completed and reviewed to confirm that potential participants meet all eligibility criteria before randomization. The investigator will maintain a screening log to record details of all participants screened and to confirm eligibility or record reasons for screening failure, as applicable.
- Procedures conducted as part of the participant's routine clinical management (eg, blood count) and obtained before signing of informed consent may be utilized for screening or baseline purposes provided the procedure meets the protocol-defined criteria and has been performed within the timeframe defined in the Schedule of Activities.

Additional measures, including non-study required laboratory tests, should be performed as clinically indicated or to comply with local regulations. Laboratory toxicities (eg, suspected drug induced liver enzyme evaluations) will be monitored during the follow-up phase via on site/local labs until all study drug related toxicities resolve, return to baseline, or are deemed irreversible.

If a participant shows pulmonary-related signs (hypoxia, fever) or symptoms (eg. dyspnea, cough, fever) consistent with possible pulmonary adverse events, the participant should be immediately evaluated to rule out pulmonary toxicity, according to the suspected pulmonary toxicity management algorithm in the BMS-936558 (nivolumab) Investigator Brochure.

Some of the assessments referred to in this section may not be captured as data in the eCRF. They are intended to be used as safety monitoring by the treating physician. Additional testing or assessments may be performed as clinically necessary or where required by institutional or local regulations.

9.1 Efficacy Assessments

The primary efficacy assessment will be MPR rate (defined as number of randomized participants with <10% residual tumor in lung and lymph nodes as evaluated by BIPR, divided by the number of randomized participants for each treatment group), which will be assessed by BIPR at the time of definitive surgery. Surgery should be performed within 6 weeks after completing up to 3 cycles (last dose) of nivolumab or chemotherapy. The following lymph node levels should be samples at the time of definitive surgery: level 4, 7, and 9 for right sided tumors or levels 9, 7, and 5 and/or 6 for left sided tumors, and hilar nodes 10 and 11. For histologic assessment, all tumor and lymph node tissue should be sectioned at 1 centimeter intervals. For assessments of pathological response, the percentage of viable tumor cells in at least 1 section per centimeter of the tumor should be evaluated for all tumor and lymph node samples. Tumor sample acquisition guidelines and submission process will be outlined in the study Pathology Manual to be provided by the third-party pathology vendor.

Radiographic assessments will be performed with the Schedule Activities in Table 2.-2.

Disease recurrence, change in tumor measurements, and tumor response will be assessed by the BICR using the RECIST 1.1 criteria.

Approved v1.0 [redacted]

9.1.1 Imaging Assessment for the Study

Study evaluations will take place in accordance with the Schedule of Activities.

Screening (baseline) assessments are to be performed within 28 days prior to the first dose. PET/CT with IV contrast (CT of diagnostic quality) should be assessed at baseline. Participants with suspected brain metastases and all those with stage II disease or higher should be evaluated with MRI/CT of the brain without contrast. Tumor assessments should be performed following RECIST 1.1 criteria.

A preoperative PET/CT with IV contrast (CT of diagnostic quality) should be acquired within 7 days of surgery.

Postoperative assessments with CT with IV contrast of the chest, including the liver and adrenal glands, and CT or MRI of suspected/known sites of disease should occur every 12 weeks for 2 years (104 weeks), then every 6 months (24 weeks ± 7 days) for 3 years, and then every year (52 weeks ± 7 days) for 5 years or until disease recurrence or progression confirmed by BICR. The same imaging method should be used as the screening/baseline.

Images will be submitted to the third-party radiology vendor for central review as they are performed on an ongoing basis. Sites will be trained prior to scanning the first study participant. Image acquisition guidelines and submission process will be outlined in the study Imaging Manual to be provided by the radiology vendor. Any incidental findings of potential clinical relevance that are not directly associated with the objectives of the protocol should be evaluated and handled by the Study Investigator as per standard medical/clinical judgement.

9.1.2 Patient-reported Outcomes

The evaluation of health-related quality of life (QoL) is an increasingly important aspect of a clinical efficacy in oncology trials. Such data provides an understanding of the impact of treatment from the participants' perspective and offers insights into the patient experience that may not be captured through physician reporting. Generic health-related QoL scales additionally provide data necessary in calculating utility values for health economic models.

Participants will be asked to complete the 3-level version of the EQ-5D before any clinical activities are performed during on-treatment clinic visits, at Post-neoadjuvant Visits 1 and 2, and at designated visits or during phone calls during the Survival Follow-up Phase. The questionnaire will be provided in the participants preferred language and may be administered by telephone during the Survival Follow-up Phase. A standardized script will be used to facilitate telephone administration of the EQ-5D-3L. Table 2.-2 and Table 2.-3 provide information regarding the timing of participant-reported outcomes assessments.

The EQ-5D-3L is a standardized instrument used to measure self-reports of health status and functioning. The instrument's descriptive system consists of 5 dimensions: mobility, self-care, usual activities, pain/discomfort, and anxiety/depression. Each dimension has 3 levels, reflecting "no health problems," "moderate health problems," and "extreme health problems." A dimension for which there are no problems is said to be at level 1, while a dimension for which there are

extreme problems is said to be at level 3. Thus, the vectors 11111 and 33333 represent the best health state and the worst health state, respectively, described by the EQ-5D-3L. Altogether, the instrument describes $3^5 = 243$ health states. Empirically derived weights can be applied to an individual's responses to the EQ-5D-3L descriptive system to generate an index measuring the value to society of his or her current health. Such preference-weighting systems have been developed for Japan, UK, US, Spain, Germany, and numerous other populations. In addition, the EQ-5D-3L includes a visual analog scale that allows respondents to rate their own current health on a 101-point scale ranging from "best imaginable" to "worst imaginable" health. The EQ-5D is available for use in over 150 languages.

9.2 Adverse Events

The definitions of an AE or serious adverse event (SAE) can be found in Appendix 8.

AEs will be reported by the participant (or, when appropriate, by a caregiver, surrogate, or the participant's legally authorized representative).

The investigator and any designees are responsible for detecting, documenting, and reporting events that meet the definition of an AE or SAE and remain responsible for following up AEs that are serious, considered related to the study treatment or the study, or that caused the participant to discontinue before completing the study.

Contacts for SAE reporting specified in Appendix 8.

Immune-mediated adverse events (IMAEs) are AEs consistent with an immune-mediated mechanism or immune-mediated component for which non-inflammatory etiologies (eg, infection or tumor progression) have been ruled out. IMAEs can include events with an alternate etiology which were exacerbated by the induction of autoimmunity. Information supporting the assessment will be collected on the participant's case report form.

9.2.1 Time Period and Frequency for Collecting AE and SAE Information

The collection of nonserious AE information should begin at initiation of study treatment until 100 days after the last dose of neoadjuvant therapy or 90 days after surgery, whichever is longer, and 30 days after the last dose of adjuvant therapy, at the time points specified in the Schedule of Activities (Section 2). Nonserious AE information should also be collected from the start of a placebo lead-in period or other observational period intended to establish a baseline status for the participants.

Sections 5.6.1 and 5.6.2 in the Investigator Brochure (IB) represent the Reference Safety Information to determine expectedness of serious adverse events for expedited reporting. Following the participant's written consent to participate in the study, all SAEs, whether related or not related to study drug, must be collected, including those thought to be associated with protocol-specified procedures.

All SAEs must be collected that occur during the screening period and 100 days of discontinuation of dosing. If applicable, SAEs must be collected that relate to any later protocol-specified procedure (eg, a follow-up skin biopsy).

The investigator must report any SAE that occurs after these time periods and that is believed to be related to study drug or protocol-specified procedure.

- Medical occurrences that begin before the start of study treatment but after obtaining informed consent will be recorded on the appropriate section of the eCRF section.
- All SAEs will be recorded and reported to Sponsor or designee within 24 hours, as indicated in Appendix 8.
- The investigator will submit any updated SAE data to the sponsor within 24 hours of this being available.

Investigators are not obligated to actively seek AEs or SAEs in former study participants. However, if the investigator learns of any SAE, including a death, at any time after a participant has been discharged from the study, and he/she considers the event reasonably related to the study treatment or study participation, the investigator must promptly notify the sponsor.

The method of evaluating, and assessing causality of AEs and SAEs and the procedures for completing and reporting/transmitting SAE reports are provided in Appendix 8.

9.2.2 Method of Detecting AEs and SAEs

Adverse events can be spontaneously reported or elicited during open-ended questioning, examination, or evaluation of a participant. (In order to prevent reporting bias, participants should not be questioned regarding the specific occurrence of one or more AEs.)

9.2.3 Follow-up of AEs and SAEs

- Nonserious AEs should be followed to resolution or stabilization, or reported as SAEs if they become serious (see Appendix 8)
- Follow-up is also required for nonserious AEs that cause interruption or discontinuation of study treatment and for those present at the end of study treatment as appropriate
- All identified nonserious AEs must be recorded and described on the nonserious AE page of the CRF (paper or electronic). Completion of supplemental CRFs may be requested for AEs and/or laboratory abnormalities that are reported/identified during the course of the study

After the initial AE/SAE report, the investigator is required to proactively follow each participant at subsequent visits/contacts. All SAEs, and non-serious AEs of special interest (as defined in Section 9.2 will be followed until resolution, until the condition stabilizes, until the event is otherwise explained, or until the participant is lost to follow-up (as defined in Section 8.3).

Further information on follow-up procedures is given in Appendix 8.

9.2.4 Regulatory Reporting Requirements for SAEs

- Prompt notification by the investigator to the Sponsor of SAEs is essential so that legal obligations and ethical responsibilities towards the safety of participants and the safety of a product under clinical investigation are met.
- An investigator who receives an investigator safety report describing SAEs or other specific safety information (e.g., summary or listing of SAEs) from the Sponsor will file it along with

the Investigator's Brochure and will notify the IRB/IEC, if appropriate according to local requirements.

Sponsor or designee will be reporting adverse events to regulatory authorities and ethics committees according to local applicable laws including European Directive 2001/20/EC and FDA Code of Federal Regulations 21 CFR Parts 312 and 320. A SUSAR (Suspected, Unexpected Serious Adverse Reaction) is a subset of SAEs and will be reported to the appropriate regulatory authorities and investigators following local and global guidelines and requirements.

All SAEs must be collected that occur during the screening period and within 100 days of the last dose of study treatment. For participants randomized/assigned to treatment and never treated with study drug, SAEs should be collected for 30 days from the date of randomization.

9.2.5 Pregnancy

If, following initiation of the study treatment, it is subsequently discovered that a participant is pregnant or may have been pregnant at the time of study exposure, including during at least 5 half-lives after product administration, the investigator must immediately notify the BMS Medical Monitor/designee of this event and complete and forward a Pregnancy Surveillance Form to BMS Designee within 24 hours of awareness of the event and in accordance with SAE reporting procedures described in Appendix 8.

In most cases, the study treatment will be permanently discontinued in an appropriate manner (eg, dose tapering if necessary for participant safety). Please call the BMS Medical Monitor within 24 hours of awareness of the pregnancy.

Protocol-required procedures for study discontinuation and follow-up must be performed on the participant.

Follow-up information regarding the course of the pregnancy, including perinatal and neonatal outcome and, where applicable, offspring information must be reported on the Pregnancy Surveillance Form.

Any pregnancy that occurs in a female partner of a male study participant should be reported to Sponsor or designee. In order for Sponsor or designee to collect any pregnancy surveillance information from the female partner, the female partner must sign an informed consent form for disclosure of this information. Information on this pregnancy will be collected on the Pregnancy Surveillance Form.

9.2.6 Laboratory Test Result Abnormalities

The following laboratory test result abnormalities should be captured on the nonserious AE CRF page or SAE Report Form electronic, as appropriate. Paper forms are only intended as a back-up option when the electronic system is not functioning.

- Any laboratory test result that is clinically significant or meets the definition of an SAE

Approved v1.0 [redacted]

- Any laboratory test result abnormality that required the participant to have study treatment discontinued or interrupted
- Any laboratory test result abnormality that required the participant to receive specific corrective therapy

It is expected that wherever possible, the clinical rather than laboratory term would be used by the reporting investigator (e.g., anemia versus low hemoglobin value).

9.2.7 Potential Drug Induced Liver Injury (DILI)

Wherever possible, timely confirmation of initial liver-related laboratory abnormalities should occur prior to the reporting of a potential DILI event. All occurrences of potential DILIs, meeting the defined criteria, must be reported as SAEs (see Section 9.2 and Appendix 8 for reporting details).

Potential drug induced liver injury is defined as:

1) AT (ALT or AST) elevation > 3 times upper limit of normal (ULN)

AND

2) Total bilirubin > 2 times ULN, without initial findings of cholestasis (elevated serum alkaline phosphatase),

AND

3) No other immediately apparent possible causes of AT elevation and hyperbilirubinemia, including, but not limited to, viral hepatitis, pre-existing chronic or acute liver disease, or the administration of other drug(s) known to be hepatotoxic.

9.2.8 Other Safety Considerations

Any significant worsening noted during interim or final physical examinations, electrocardiogram, x-ray filming, any other potential safety assessment required or not required by protocol should also be recorded as a nonserious or serious AE, as appropriate, and reported accordingly.

<u>Definition of immune-mediated adverse events (IMAEs)</u>

Immune-mediated AEs are specific events (that include pneumonitis, diarrhea/colitis, hepatitis, nephritis/renal dysfunction, rash, and endocrine [adrenal insufficiency, hypothyroidism/thyroiditis, hyperthyroidism, diabetes mellitus, and hypophysitis]) for which participants received immunosuppressive medication for treatment of the event, with the exception of endocrine events (hypothyroidism/thyroiditis, hyperthyroidism, hypophysitis, diabetes mellitus, adrenal insufficiency), which are included regardless of treatment since these events are often managed without immunosuppression.

Immune-mediated AEs are specific events (or groups of PTs describing specific events) that include pneumonitis, diarrhea/colitis, hepatitis, nephritis/renal dysfunction, rash, endocrine (adrenal insufficiency, hypothyroidism/thyroiditis, hyperthyroidism, diabetes mellitus, and

Approved v1.0 [redacted]

hypophysitis), and other specific events, considered as potential immune-mediated events by investigator, that meet the definition summarized below:

- those occurring within 100 days of the last dose
- regardless of causality
- with no clear alternate etiology based on investigator assessment, or with an immune-mediated component
- treated with immune-modulating medication (Of note, adrenal insufficiency, hypothyroidism/thyroiditis, hyperthyroidism, diabetes mellitus, and hypophysitis are considered IMAEs regardless of immune-modulating medication use, since endocrine drug reactions are often managed without immune-modulating medication).

Table 9.2.8-1 below provides a summary of the IMAEs category and their respective preferred terms.

Table 9.2.8-1: Preferred Terms Included in Analysis of IMAEs to Support Warnings and Precautions

IMAE Category	PTs included under IMAE Category
Pneumonitis	Pneumonitis, interstitial lung disease
Diarrhea/Colitis	Diarrhea, colitis, enterocolitis
Hepatitis	Hepatotoxicity, hepatitis, hepatitis acute, autoimmune hepatitis, AST increased, ALT increased, bilirubin increased, ALP increased
Adrenal insufficiency	Adrenal insufficiency
Hypothyroidism/Thyroiditis	Hypothyroidism, thyroiditis<br>Thyroiditis acute (collapsed with thyroiditis for frequency), Autoimmune thyroiditis (collapsed with thyroiditis for frequency)
Hyperthyroidism	Hyperthyroidism
Hypophysitis	Hypophysitis
Diabetes mellitus	Diabetes mellitus, diabetic ketoacidosis
Nephritis and renal dysfunction	Nephritis, nephritis allergic, tubulointerstitial nephritis, acute renal failure, renal failure, increased creatinine
Rash	Rash, rash maculopapular

9.2.9 Management Algorithms

Immuno-oncology (I-O) agents are associated with AEs that can differ in severity and duration than AEs caused by other therapeutic classes. Nivolumab and ipilimumab are considered an immuno-oncology agent in this protocol. Early recognition and management of AEs associated with immuno-oncology agents may mitigate severe toxicity. Management Algorithms have been developed to assist investigators in assessing and managing the following groups of AEs:

- Gastrointestinal
- Renal

- Pulmonary
- Hepatic
- Endocrinopathy
- Skin
- Neurological

The above algorithms are found in the nivolumab Investigator Brochure and Appendix 4.

9.3 Overdose

All occurrences of overdose must be reported as SAEs (see Section 9.2).

An overdose is defined as the accidental or intentional administration of any dose of a product that is considered both excessive and medically important. All occurrences of overdose must be reported as an SAE (see Section 9.2).

9.4 Safety

Planned time points for all safety assessments are listed in the Schedule of Activities.

At screening, a medical history will be obtained to capture relevant underlying conditions. The screening examinations should include weight, height, ECOG Performance Status, blood pressure (BP), heart rate (HR), and temperature. Screening assessments and screening laboratory assessments should be performed in accordance with Table 2.-1.

Screening local laboratory assessments should be done within 14 days prior to randomization unless otherwise noted in Table 2.-1 and are to include: CBC with differential, chemistry panel including LFTs (ALT, AST, total bilirubin, alkaline phosphatase), BUN or serum urea level, creatinine, Ca, Na, K, Cl, phosphate, LDH, glucose, lipase, amylase, and thyroid panel including TSH, free T3, and free T4.

Screening pregnancy tests for WOCBP must be performed within 24 hours prior to the initial administration of study drug.

Participants will be evaluated for safety if they have received any study drug. Toxicity assessments will be continuous during the treatment phase as well as during the first 2 safety (neo-adjuvant) follow-up visits, for 90 days after surgery, and for 30 days after adjuvant therapy. Once participants reach the Survival Follow-up Phase, either in-person visits or documented telephone calls/email correspondence to assess the participant's status are acceptable.

Adverse events and laboratory values will be graded according to the NCI-CTCAE version 4.

The start and stop time of the study therapy infusions and any interruptions or infusion rate reductions should be documented.

Physical examinations are to be performed as clinically indicated. If there are any new or worsening clinically significant changes since the last exam, report changes on the appropriate non-serious or serious adverse event page.

On treatment local laboratory assessments are to be completed within 3 calendar days prior to dosing: CBC with differential, albumin, LFTs (ALT, AST, total bilirubin, alkaline phosphatase), BUN or serum urea level, creatinine, Ca, Mg, Na, K, Cl, LDH, glucose, amylase, and lipase.

On treatment pregnancy tests should be performed as per the schedule in the Schedule of Activity Table.

Additional measures, including non-study required laboratory tests, should be performed as clinically indicated or to comply with local regulations. Laboratory toxicities (eg, suspected drug induced liver enzyme evaluations) will be monitored during the Neoadjuvant and Adjuvant Follow-up Phases via on site/local labs until all study drug-related toxicities resolve, return to baseline, or are deemed irreversible.

If a participant shows pulmonary-related signs (hypoxia, fever) or symptoms (eg. dyspnea, cough, fever) consistent with possible pulmonary adverse events, the participant should be immediately evaluated to rule out pulmonary toxicity, according to the suspected pulmonary toxicity management algorithm in the nivolumab Investigator Brochure.

Some of the assessments referred to in this section may not be captured as data in the eCRF. They are intended to be used as safety monitoring by the treating physician. Additional testing or assessments may be performed as clinically necessary or where required by institutional or local regulations.

9.4.1 Clinical Safety Laboratory Assessments

Investigators must document their review of each laboratory safety report.

Please refer to the Schedule of Activities in Section 2 for details related to the required laboratory assessment.

9.4.2 Imaging Safety Assessment

Any incidental findings of potential clinical relevance that are not directly associated with the objectives of the protocol should be evaluated and handled by the Study Investigator as per standard medical/clinical judgment.

9.5 Pharmacokinetics

Samples for PK assessments will be collected for all participants receiving nivolumab and ipilimumab as described in the Table 9.5-1. All time points are relative to the start of study drug administration. All on-treatment time points are intended to align with days on which study drug is administered, if dosing occurs on a different day, the PK sampling should be adjusted accordingly. Further details of sample collection, processing, and shipment will be provided in the laboratory procedures manual. PK samples will be analyzed for nivolumab and ipilimumab by a validated ligand binding assay.

Table 9.5-1: Pharmacokinetic (PK) Sample Collections for Nivolumab and Ipilimumab - Arm A

Study Day<br>1 Cycle = 2 weeks)	Event<br>(Relative to Dosing)<br>Hour	Time<br>(Relative to Dosing)<br>Hour: Min	Pharmacokinetic Blood Sample for Nivolumab	Pharmacokinetic Blood Sample for Ipilimumab
C1D1	0.5 (EOI)[a]	00:30	X	X
C2D1	Predose[b]	00:00	X	X
C3D1	Predose[b]	00:00	X	X

[a] EOI: End of Infusion. This sample should be taken immediately prior to stopping the second drug infusion. If the end of infusion is delayed to beyond the nominal infusion duration, the collection of this sample should also be delayed accordingly. EOI samples may not be collected from the same IV access as drug was administered.

[b] Predose sample should be collected just before the administration of the first drug (preferably within 30 minutes). If it is known that a dose is going to be delayed, then the predose sample should be collected just prior to the delayed dose. However, if a predose sample is collected, but the dose is subsequently delayed, an additional predose sample should not be collected.

9.6 Pharmacodynamics

Pharmacodynamic parameters are not evaluated in this study.

9.7 Pharmacogenomics

Not applicable.

9.8 Biomarkers

9.8.1 Additional Research Collection

Additional research retention are mandatory for all study participants, except where prohibited by local laws or regulations, ethics committees, institutional requirements, or where a waiver is provided by BMS or their designee. Where one or more of these exceptions occurs, participation in the additional research should be encouraged, but will not be a condition of overall study participation. Study participants may opt out of the additional research.

This protocol will include residual sample storage for additional research (AR).

This retention for additional research is intended to expand the translational R&D capability at Bristol-Myers Squibb and will support as yet undefined research aims that will advance our understanding of disease and options for treatment. It may also be used to support health authority requests for analysis, and advancement of pharmacodiagnostic development to better target drugs to the right participants. This may also include genetic/genomic exploration aimed at exploring disease pathways, progression and response to treatment etc.

All requests for access to samples or data for additional research will be vetted through a diverse committee of the study sponsor's senior leaders in Research and Development to ensure the research supports appropriate and well-defined scientific research activities.

Approved v1.0 [redacted]

[redacted]

Samples kept for future research will be stored at the BMS Biorepository [redacted] or an independent, BMS-approved storage vendor. The manager of these samples will ensure they are properly used throughout their usable life and will destroy the samples at the end of the scheduled storage period, no longer than fifteen (15) years after the end of the study or the maximum allowed by applicable law. Transfers of samples by research sponsor to third parties will be participant to the recipient's agreement to establish similar storage procedures.

Samples will be stored in a coded fashion; no researcher will have access to the key, which is securely held at the clinical site, so that there is no direct ability for a researcher to connect a sample to a specific individual.

Further details of sample collection and processing will be provided to the site in the procedure manual.

[redacted]

9.8.1.1 Tumor Tissue Specimens

Archival (slides [blocks/slides] ≤ 6 months) FFPE tumor tissue collected at initial diagnosis is required for study entry. Samples will be sent to a third party to stain and score for PD-L1 expression using the PD-L1 IHC 28-8 pharmDx kit (Dako). Stained tissue samples will be assessed by a pathologist at a central lab identified by the Sponsor and scored as PD-L1 expressing if membrane staining is observed in ≥ 1% tumor cells among a minimum of 100 evaluable tumor cells. If an archived specimen is not available, a fresh tumor biopsy will be collected. Additional tumor will be collected during surgical resection, which will be after 3 cycles of treatment in each arm. A third (optional) biopsy will be collected at the time of disease progression.

Collected tumors may be further analyzed by additional modalities [redacted]

Approved v1.0 [redacted]

[redacted]

[redacted]

9.8.2 Safety Analyses

Endpoint	Statistical Analysis Methods
Exploratory	Proportion of delayed or canceled surgery, duration of surgery, length of hospital stay, surgical approach, incidence of AE/SAE associated with surgery, including pneumonitis, ARDS, re-admission to the Intensive Care Unity, atrial fibrillation or other supraventricular tachycardia up to 90 days after surgery will be summarized by treatment group using descriptive statistics.<br>The safety and tolerability objective will be measured by the incidence of adverse events, serious adverse events, deaths, and laboratory abnormalities.<br>The safety analysis will be performed in all treated participants. The rate of treatment-related selected AEs and SAEs in each treatment arm will be summarized. Descriptive statistics of safety will be presented using National Cancer Institute (NCI) Common Terminology Criteria for Adverse Events (CTCAE) version 4.0 by treatment arm. All treatment-emergent AEs, drug-related AEs, SAEs and drug-related SAEs will be tabulated using worst grade per NCI CTCAE v 4.0 criteria by system organ class and preferred term. On-study lab parameters including hematology, chemistry, liver function, thyroid function and renal function will be summarized using worst grade per NCI CTCAE v 4.0 criteria.

9.9 Health Economics OR Medical Resource Utilization and Health Economics

Health Economics/Medical Resource Utilization and Health Economics parameters will not be evaluated in this study.

9.10 Other Assessments

9.10.1 Diagnostic/Pre-surgical Evaluation and Surgical Guidelines:

A participant is considered functionally operable if the following criteria are met:

- ECOG Performance Status ≤ 1
- Absence of major associated pathologies that increase the surgery risk to an unacceptable level
- Pulmonary function capacity capable of tolerating the proposed lung resection according to the surgeon
- Absence of locally advanced, unresectable or metastatic disease (stage IV) in the pre-treatment assessment

All participants enrolled on this study must be surgical candidates with clinical stage IB (≥ 4 cm), II or resectable IIIA (N2) NSCLC. Participants will have undergone radiographic evaluation (PET/CT) indicating no evidence of distant disease and no evidence of unresectable loco-regional tumor extension (EBUS or thoracostomy) prior to randomization. Any further preoperative testing that is recommended by the surgeon or anesthesiologist will be performed as part of standard of care. Surgery for participants enrolled on this protocol will be according to generally accepted standards of care. Operative approach (VATS vs open) will be determined by the surgeon.

Accepted types of resection (with negative margins) will consist of lobectomy, sleeve lobectomy, bi-lobectomy, or pneumonectomy. Resections by segmentectomy or wedge resection will not be

Approved v1.0 [redacted]

accepted, unless there is more than 1 ipsilateral lesion, and a lobectomy is performed on the most extensively-involved lobe. If mediastinoscopy was not performed preoperatively, it is expected that, at a minimum, mediastinal lymph node systematic sampling will have occurred, though complete mediastinal lymph node dissection (MLND) is preferred. It is recommended that participants have at least 3 mediastinal and hilar lymph node stations sampled during surgery. Systematic sampling is defined as removal of at least 1 representative lymph node at specified levels. MLND entails resection of all lymph nodes at those same levels. For a right thoracotomy, sampling or MLND is required at levels 4, 7, and 9. For a left thoracotomy, levels 5 and/or 6, 7, and 9. Hilar nodes 10 and 11 should also be required. If there is clear documentation in the operative report or in a separately submitted addendum by the surgeon of exploration of the required lymph node areas, the participant will be considered eligible if no lymph nodes are found in those areas.

Participants should have a PET-CT scan with IV contrast of chest performed within the 7 days prior to planned surgery to assess for clinical response. Surgery should be performed within 6 weeks of completing up to 3 cycles (last dose) of nivolumab or chemotherapy.

9.10.2 Post-surgical Evaluation:

Following surgery, radiographic assessment will be performed every 12 weeks (± 7 days) for 2 years (104 weeks) following surgery, every 6 months (24 weeks ± 7 days) for 3 years, and then every year (52 weeks ±7 days) for 5 years or until recurrence or progression of disease is documented.

10. STATISTICAL CONSIDERATIONS

10.1 Sample Size Determination

MPR rate is the primary endpoints for this study. The primary analysis population is PD-L1+ (PD-L1 tumor expression ≥ 1%) participants.

Approximately 228 PD-L1+ participants will be randomized to the 2 arms in a 1:1 ratio. With an estimated MPR rate of 20% on chemotherapy and 40% on nivolumab and ipilimumab, respectively, the 228 PDL1 + participants will provide about 90% power to detect a response rate difference of 20% with a 2-sided type I error of 5%.

Assuming 70% of randomized participants are PD-L1+ participants, it is estimated that approximately 326 participants (all comers) will be randomized. Assuming an accrual rate of 14 participants (all comers) a month, it is anticipated that the 228 PDL1+ participants will be randomized in approximately 24 months. The primary endpoint will be assessed at the time of surgery of the last randomized participants. The primary endpoint is expected to be analyzed after about 27 months.

The secondary endpoint of EFS as assessed by BICR in the population of PD-L1+ participants will be tested hierarchically (Section 10.3.1).

For EFS, 186 events ensure that a 2-sided 5% significance level sequential test procedure with 1 interim analysis after 140 events (75% of events required for final analysis) will have 80% power

Approved v1.0 [redacted]

assuming an exponential distribution with the median EFS time in the control is 40 months and of 60 months in the nivolumab and ipilimumab arm (corresponding to an hazard ratio of 0.66). It is anticipated it will take about 130 months to observe 186 EFS events.

10.2 Populations for Analyses

Population	Description
All enrolled participants	All participants who signed an informed consent form and were registered into the IRT
All randomized participants	All participants who were randomized to any treatment arm in the study. This population is considered as the secondary efficacy analysis population. Analysis of demography, protocol deviations, baseline characteristics, and secondary efficacy analysis will be performed for this population.
PD-L1+ participants	All randomized participants with baseline PD-L1 expression in ≥ 1% of tumor cells. This is the primary efficacy analysis population. Analysis of demography, protocol deviations, baseline characteristics, and primary efficacy analysis will be performed for this population.
All treated participants	All participants who received any dose of study medication (nivolumab and ipilimumab or investigator choice of platinum doublet chemotherapy in neoadjuvant setting). This is the primary dataset for drug exposure and safety analysis.
PK participants	All participants with available serum time-concentration data from randomized participants dosed with nivolumab and ipilimumab

10.3 Statistical Analyses

10.3.1 Efficacy Analyses

Endpoint	Statistical Analysis Methods
Primary	Pathological response will be summarized by category for each treatment group. MPR rate will be computed in each treatment group along with the exact 95% CI using Clopper-Pearson method.<br>The stratified by disease stage (IB/II vs IIIA) and gender odds ratios (Mantel-Haenszel estimator) between the treatments will be provided along with the 95% CI. The difference will be tested via the Cochran Mantel-Haenszel (CMH) test using a 2-sided, 5% α level.<br>In addition, an estimate of the difference in MPR rates and corresponding 95% CI will be calculated using CMH methodology and adjusted by stratification factors.
Secondary	EFS is defined as time from randomization to any of the following events: documented progression, recurrence disease, or death due to any cause. Progression/recurrence will be assessed by BICR per RECIST 1.1 Participants who die without a reported progression/disease recurrence will be considered to have experienced an event on the date of their death. Participants who did not report progression/recurrence of disease or die will be censored on the date of their last evaluable tumor assessment. Participants who did not have any on-study tumor assessments and did not die will be censored on the date they were randomized. Participants who started any subsequent anti-cancer therapy outside of the protocol-specified adjuvant therapy without a prior reported progression/recurrence will be censored at the last evaluable tumor assessment prior to initiation of the subsequent anti-cancer therapy.<br>If the comparison of primary endpoint is statistically significant, EFS among PD-L1+ participants will be tested. The distribution of EFS among PD-L1+ participants will be compared between 2 randomized arms via a 2-sided, log rank test stratified by the same stratification factor

Approved v1.0 [redacted]

Endpoint	Statistical Analysis Methods
	as the primary endpoint (ie, disease stage [IB/II vs IIIA] and gender).The hazard ratio and the corresponding (1-adjustedα) confidence interval will be estimated in a stratified Cox proportional hazards model using the randomized arm as a single covariate. The EFS curves for each randomized arm will be estimated using the Kaplan-Meier (KM) product-limit method using a log-log transformation. To allow for sufficient power, this analysis will take place when about 186 EFS events have been observed. In addition, EFS rates at 1, 2, 3, and 4 years will be estimated using KM estimates on the EFS curve for each randomized arm provided a minimum follow-up is longer than the time point to generate the rate. Associated 2-sided 95% CIs will be calculated using the Greenwood formula (using log-log transformation).<br>Sensitivity analysis on EFS will also be performed, and details will be included in statistical analysis plan.<br>At the time of EFS analysis, OS curves, OS medians with 95% CIs, and OS rates with rates at 1, 2, 3, and 4 years with 95% CIs will be estimated using Kaplan-Meier methodology. HRs and corresponding 2-sided 95% CI will be estimated using a Cox proportional hazards model with treatment group as a single covariate, stratified by the above factors. There will be no formal comparison of OS.<br>Complete pathological response among PD-L1+ participants will be summarized by treatment arm. Response rates and their corresponding 95% exact CI will be calculated by Clopper-Pearson method presented for each randomized arm.
Exploratory	Will be described in the statistical analysis plan finalized before database lock

10.3.2 Other Analyses

Biomarker exploratory analyses will be described in the statistical analysis plan finalized before database lock. The population pharmacokinetics analysis will be presented separately from the main clinical study report.

The EQ-5D-3L questionnaire completion rate, defined as the proportion of questionnaires actually received out of the expected number, will be calculated and summarized at each assessment point. Summary statistics (ie, N, mean with SD and 95% CI, median, first and third quartiles, minimum, maximum) for the EQ-5D-3L utility index and VAS scores, as well as their corresponding changes from baseline, will be tabulated by treatment and planned time point. In the base case, index scores will be derived using the UK weighting algorithm. In addition, the proportion of participants reporting no, moderate, or severe problems in each of the 5 EQ-5D-3L dimensions will be summarized by treatment group at each time point. Proportions will be based on the number of participants assessed at assessment time point. A by-participant listing of the level of problems in each dimension, corresponding EQ-5D-3L health state (ie, 5-digit vector), EQ-5D-3L index score, and EQ-5D VAS score will be provided.

10.3.3 Interim Analyses

One formal interim analysis for EFS is planned after 140 events have been observed. This is projected to occur approximately 76 months after study initiation. This formal comparison of EFS will allow for early stopping for superiority, and the boundaries for declaring superiority will be derived based on the actual number of events using Lan-DeMets α spending function with O'Brien

and Fleming type of boundary in EAST v6.3.1. If the analysis were performed exactly at 140 events, the boundary for declaring superiority would be 0.02.

An independent statistician external to BMS will perform the analysis. If the study continues beyond the interim analysis the nominal significance level for the final look after 186 EFS events would be 0.046. All events in the database at the time of the lock will be used. If number of final events exceeds the number specified per protocol (186 events), final boundary will not be recalculated using updated information fraction at interim.

Approved v1.0 [redacted]

11. REFERENCES

1 Mountain, CA. Regional lymph node classification for lung cancer staging. Chest. 1997: 1718-23.

2 Goldstraw P, Crowley J, Chansky K, et al for the International Association for the Study of Lung Cancer International Staging Committee. The IASLC Lung Cancer Staging Project: proposals for the revision of the TNM stage groupings in the forthcoming (seventh) edition of the TNM Classification of malignant tumours. J Thorac Onco. 2007; 2(8):706-14.

3 Detterbeck FC, Boffa DJ, Tanoue LT. The new lung cancer staging system. Chest. 2009; 136: 260-71.

4 NSCLC Meta-analyses Collaborative Group. Adjuvant chemotherapy, with or without postoperative radiotherapy, in operable non-small-cell lung cancer: two meta-analyses of individual patient data. Lancet. 2010 Apr 10;375(9722):1267-77.

5 Siegel RL, Miller KD, Jemal A. Cancer statistics, 2015. A Cancer J Clin. 2015; 65(1):5-29.

6 Arriagada R, Bergman B, Dunant A, et al. Cisplatin-based adjuvant chemotherapy in patients with completely resected non-small-cell lung cancer. N Engl J Med, 2004, 351-60.

7 Douillard JY, R. R. Adjuvant vinorelbine plus cisplatin versus observation in patients with completely resected stage IB-IIIA non-small-cell lung cancer (Adjuvant Navelbine International Trialist Association [ANITA]): a randomised controlled trial. Lancet Oncology. 2006;7: 719-27.

8 Strauss GM, Herndon JE 2nd, Maddaus MA, et al. Adjuvant paclitaxel plus carboplatin compared with observation in stage IB non-small cell lung cancer: CALGB 9633 with the Cancer and Leukemia Group B, Radiation Therapy Oncology Group and Northern Central Cancer Treatment Study Groups. Journal of Clinical Oncology. 2008;26: 5043-51.

9 Winton T, L. R. Vinorelbine plus cisplatin vs. observation in resected non-small-cell lung cancer. N Engl J Med. 2005;352: 2589-97.

10 Burdett S, Stewart LA, Rydzewska L. A systematic review and meta-analysis of the literature: chemotherapy and surgery versus surgery alone in non-small cell lung cancer. J Thorac Oncol. 2006 Sep;1(7):611-21.

11 Brahmer J, Reckamp KL, Baas P, et al. Nivolumab versus Docetaxel in Advanced Squamous Cell Non-small Cell Lung Cancer. N Engl J Med 2015;373:123-35.

12 Borghaei H, Paz-Ares L, Horn L, et al. Nivolumab versus Docetaxel in Advanced Nonsquamous Non-small Cell Lung Cancer. N Engl J Med 2015;373:1627-39.

13 Sorenson S, Glimelius B, Nygren P. A Systematic Overview of Chemotherapy effects in non-small cell lung cancer. Act Oncologica. 2001; 40 (2-3): 327-339.

Approved v1.0 [redacted]

14 Pardoll D. Does the immune system see tumors as foreign or self? Annu Rev Immunol. 2003;21:807-39.

15 Zitvogel L, Tesniere A, Kroemer G. Cancer despite immunosurveillance: immunoselection and immunosubversion. Nat Rev Immunol. 2006;6:715-27.

16 Dunn GP, Bruce AT, Ikeda H, et al. Cancer immunoediting: from immunosurveillance to tumor escape. Nat Immunol. 2002;3:991-8.

17 Greenwald RJ, Freeman GH, Sharpe AH. The B7 family revisited. Annu Rev Immunol. 2004;23:515-48.

18 Freeman FJ, Long AJ, Iwai Y, et al. Engagement of the PD-1 immunohibitory receptor by a novel B7 family member leads to negative regulation of lymphocyte activation. J Exp Med 2000; 192(7): 1027-34.

19 Sharpe AH, Wherry EJ, Ahmed R, et al. The function of programmed cell death 1 and its ligands in regulating autoimmunity and infection. Nature Immunol. 2007;8:237-45.

20 Wolchok JD, Hoos A, O'Day S, et al. Guidelines for the evaluation of immune therapy activity in solid tumors: immune-related response criteria. Clin Cancer Res. 2009;15:7412-20.

21 Postow MA, Harding J, Wolchok JD. Targeting immune checkpoints: Releasing the restraints on anti-tumor immunity for patients with melanoma. Cancer J 2012; 18:153-159.

22 Hellmann, MD, Gettinger, SN, Goldman, JW, et al. J Clin Oncol 34, 2016 (suppl; abstr 3001).

23 Hellmann MD, Gettinger SN Goldman J, et al. CheckMate 012: Safety and Efficacy of First‐line Nivolumab and Ipilimumab in Advanced NSCLC. Presented at the 2016 American Society of Clinical Oncologist Annual Meeting, June 3-7, 2016, Chicago, Il.

24 Xu XL, D. L. Neoadjuvant chemoradiotherapy or chemotherapy followed by surgery is superior to that followed by definitive chemoradiation or radiotherapy in stage IIIA (N2) nonsmall-cell lung cancer: a meta-analysis and system review. Onco Targets Ther.2016:9: 845-53.

25 Gilligan D, Nicolson M, Smith I, et al. Preoperative chemotherapy in patients with resectable non-small cell lung cancer: results of the results of the MRC 22 ? NVALT 2 / EORTC 08012 multicentre randomised trial and update of systematic review. Lancet. 2007;369:1929.

26 Depierre A, Milleron B, Moro-Sibilot D, et al. Preoperative chemotherapy followed by surgery compared with primary surgery in resectable stage I (except T1N0), II, and IIIa non-small-cell lung cancer. J Clin Oncol. 2002;20:247-253.

27 Scagliotti GV, Pastorino U, Vansteenkiste JF, et al. Randomized phase III study of surgery alone or surgery plus preoperative cisplatin and gemcitabine in stages IB to IIIA non-small-cell lung cancer. J Clin Oncol. 2012:30(2),172-8.

Approved v1.0 [redacted]

28 Pisters K. S9900: A phase III trial of surgery alone or surgery plus preoperative (preop) paclitaxel/carboplatin (PC) chemotherapy in early stage non-small cell lung cancer (NSCLC): Preliminary results. Proc ASCO 2005;(Abstract 7012), 23.

29 S. G. (2009). A phase III randomized study of surgery alone or surgery plus preoperative gemcitabine-cisplatin in early-stage non-small cell lung cancer (NSCLC): Follow-up data of C.h.E.S.T. . Proc ASCO 2009, abstract 7508.

30 F. E. (2009). Surgery alone, pre-operative paclitaxel/carboplatin chemotherapy followed by surgery, or surgery followed by adjuvant PC chemotherapy in early stage non-small cell lung cancer. Results of the NATCH multicenter randomized phase III trial. Proc ASCO 2009 (Abstract 7500).

31 Forde PM, Smith KN, Chaft JE, et al. Neoadjuvant anti-PD1, Nivolumab, in early stage resectable NSCLC. Presented at the 2016 European Society of Medical Oncologist, October 7-11, 2016, Copenhagen, Denmark.

32 Curran MA, Montalvo W, Yagita H, et al. PD-1 and CTLA-4 combination blockade expands infiltrating T cells and reduces regulatory T and myeloid cells within B16 melanoma tumors. PNAS 2010; 107: 4275-80.

33 Postow MA, Chesney J, Pavlick AC, et al. Nivolumab and ipilimumab versus ipilimumab in untreated melanoma. N Engl J Med 2015; 372:2006-2017.

34 Larkin J, Chiarion-Sileni V, Gonzalez R, et al. Combined Nivolumab and Ipilimumab or Monotherapy in Untreated Melanoma. New Eng J Med. 2015;373(1):23-34.

35 Calvo, et al., presented at the 18th ECCO—40th ESMO European Cancer Congress; September 25-29, 2015; Vienna, Austria.

36 Rizvi NA, Gettinger S, Goldman J et al. Safety and Efficacy of First-line Nivolumab (Anti-programmed Death-1 [PD-1]) and Ipilimumab in Non-small Cell Lung Cancer (NSCLC). Presented at WCLC 2015 Annual Meeting in Denver, CO

37 Hellmann MD, Gettinger SN, Goldman J, et al. CheckMate 012: Safety and Efficacy of First-line Nivolumab and Ipilimumab in Advanced NSCLC. Presented at the American Society of Clinical Oncologists (ASCO), June 3-7, 2016, Chicago, Illinois.

38 Hellmann MD, Chaft JE, William Jr WN, for The University of Texas MD Anderson Lung Cancer Collaborative Group. Pathological response after neoadjuvant chemotherapy in resectable non-small-cell lung cancers: proposal for the use of major pathological response as a surrogate endpoint. Lancet Oncol 2014; 15: e42–50.

39 Betticher DC, Hsu Schmitz S-F, Totsch M. Prognostic factors affecting long-term outcomes in patients with resected stage IIIA pN2 non-small-cell lung cancer: 5-year follow-up of a phase II study. British Journal of Cancer. 2006;94:1099 – 1106.

40 Pataer A, Kalhor N, Correa AM, et al for the University of Texas M. D. Anderson Lung Cancer Collaborative Research Group. Histopathologic response criteria predict survival of patients with resected lung cancer after neoadjuvant chemotherapy. J Thorac Oncol. 2012; 7(5):825-32.

Approved v1.0 [redacted]

41 NSCLC Meta-analyses Collaborative Group. Adjuvant chemotherapy, with or without postoperative radiotherapy, in operable non-small-cell lung cancer: two meta-analyses of individual patient data. Lancet. 2010;375:267-77.

42 Wakelee H, Chhatwani L. Adjuvant Chemotherapy for Resected Non-Small Cell Lung Cancer. Seminars in Thoracic and Cardiovascular Surgery. 2008 20:198-203.

43 Felip E, Rosell R, Maestre JA, et al. Preoperative chemotherapy plus surgery versus surgery plus adjuvant chemotherapy versus surgery alone in early-stage non-small-cell lung cancer. J Clin Oncol 2010; 28: 3138–45.

44 Arriagada R, Bergman B, Dunant A, et al: Cisplatin-based adjuvant chemotherapy in patients with completely resected non-small-cell lung cancer: The International Adjuvant Lung Cancer Trial Collaborative Group. N Engl J Med. 2004; 350:351-360.

45 Arriagada R, Dunant A, Pignon JP, et al: Long-term results of the International Adjuvant Lung Cancer Trial evaluating adjuvant cisplatin-based chemotherapy in resected lung cancer. J Clin Oncol 2010; 28:35-42.

46 Winton T, Livingston R, Johnson D, et al: Vinorelbine plus cisplatin vs. observation in resected non-small-cell lung cancer. N Engl J Med. 2005; 352:2589-2597.

47 Butts CA, Ding K, Seymour L, et al: Randomized, phase III trial of vinorelbine plus cisplatin compared with observation in completely resected stage IB and II non-small-cell lung cancer: Updated survival analysis of JBR-10. J Clin Oncol. 2010; 28:29-34.

48 Douillard JY, Rosell R, De Lena M, et al: Adjuvant vinorelbine plus cisplatin versus observation in patients with completely resected stage IB-IIIA non-small-cell lung cancer (Adjuvant Navelbine International Trialist Association [ANITA]): A randomised controlled trial. Lancet Oncol. 2006; 7:719-727.

49 Rami-Porta, R. Bolejack, V. Crowley, J. et al. The IASLC Cancer Staging Project: Proposals for the Revisions of the T Descriptors in the Forthcoming Eigth Edition of the TNM Classification for Lung Cancer. J Thorac Oncol. 2015 Jul;10(7):990-1003.

[redacted]

Approved v1.0 [redacted]

12. APPENDICES

APPENDIX 1 TNM STAGING SYSTEM FOR LUNG CANCER (7TH EDITION)

TNM staging system for lung cancer (7th edition)

**Primary tumor (T)**			
T1	Tumor ≤3 cm diameter, surrounded by lung or visceral pleura, without invasion more proximal than lobar bronchus		
T1a	Tumor ≤2 cm in diameter		
T1b	Tumor >2 cm but ≤3 cm in diameter		
T2	Tumor >3 cm but ≤7 cm, or tumor with any of the following features:		
	Involves main bronchus, ≥2 cm distal to carina		
	Invades visceral pleura		
	Associated with atelectasis or obstructive pneumonitis that extends to the hilar region but does not involve the entire lung		
T2a	Tumor >3 cm but ≤5 cm		
T2b	Tumor >5 cm but ≤7 cm		
T3	Tumor >7 cm or any of the following:		
	Directly invades any of the following: chest wall, diaphragm, phrenic nerve, mediastinal pleura, parietal pericardium, main bronchus <2 cm from carina (without involvement of carina)		
	Atelectasis or obstructive pneumonitis of the entire lung		
	Separate tumor nodules in the same lobe		
T4	Tumor of any size that invades the mediastinum, heart, great vessels, trachea, recurrent laryngeal nerve, esophagus, vertebral body, carina, or with separate tumor nodules in a different ipsilateral lobe		
**Regional lymph nodes (N)**			
N0	No regional lymph node metastases		
N1	Metastasis in ipsilateral peribronchial and/or ipsilateral hilar lymph nodes and intrapulmonary nodes, including involvement by direct extension		
N2	Metastasis in ipsilateral mediastinal and/or subcarinal lymph node(s)		
N3	Metastasis in contralateral mediastinal, contralateral hilar, ipsilateral or contralateral scalene, or supraclavicular lymph node(s)		
**Distant metastasis (M)**			
M0	No distant metastasis		
M1	Distant metastasis		
M1a	Separate tumor nodule(s) in a contralateral lobe; tumor with pleural nodules or malignant pleural or pericardial effusion		
M1b	Distant metastasis (in extrathoracic organs)		
**Stage groupings**			
Stage IA	T1a-T1b	N0	M0
Stage IB	T2a	N0	M0
Stage IIA	T1a,T1b,T2a	N1	M0
	T2b	N0	M0
Stage IIB	T2b	N1	M0
	T3	N0	M0
Stage IIIA	T1a,T1b,T2a,T2b	N2	M0
	T3	N1,N2	M0
	T4	N0,N1	M0
Stage IIIB	T4	N2	M0
	Any T	N3	M0
Stage IV	Any T	Any N	M1a or M1b

Adapted from: Goldstraw P, Crowley J, Chansky K, et al. The IASLC Lung Cancer Staging Project: Proposals for the revision of the TNM stage groups in the forthcoming (seventh) edition of the TNM classification of malignant tumours. J Thorac Oncol 2007; 2:706.

UpToDate

Approved v1.0 [redacted]

APPENDIX 2 ABBREVIATIONS AND TRADEMARKS

Term	Definition
AE	adverse event
ALT	alanine aminotransferase
ANC	absolute neutrophil count
AR	Additional Research
AST	aspartate aminotransferase
BICR	Blinded independent central review
BID, bid	bis in die, twice daily
BIPR	Blinded independent pathological review
BMI	body mass index
BMS	Bristol-Myers Squibb
BP	blood pressure
BUN	blood urea nitrogen
C	Celsius
CBC	complete blood count
CFR	Code of Federal Regulations
CI	confidence interval
$C1^{-}$	chloride
cm	centimeter
CONSORT	Consolidated Standards of Reporting Trials
CRF	Case Report Form, paper or electronic
cRR	clinical response rate
[redacted]	
dL	deciliter
DMC	Data Monitoring Committee
EBUS	endobronchial ultrasound
ECG	electrocardiogram
ECOG	Eastern Cooperative Oncology Group
eCRF	Electronic Case Report Form
EFS	event-free survival

Term	Definition
eg	exempli gratia (for example)
FDA	Food and Drug Administration
FFPE	formalin-fixed paraffin-embedded
FSH	follicle stimulating hormone
g	gram
GCP	Good Clinical Practice
GFR	glomerular filtration rate
[redacted]	
HBsAg	hepatitis B surface antigen
HBV	hepatitis B virus
HCV	hepatitis C virus
HIV	Human Immunodeficiency Virus
HR	heart rate
HRT	hormone replacement therapy
ICH	International Conference on Harmonisation
ie	id est (that is)
IEC	Independent Ethics Committee
IHC	Immunohistochemical
IMP	investigational medicinal products
IRB	Institutional Review Board
IRT	Interactive Response Technology
IU	International Unit
IV	intravenous
kg	kilogram
L	liter
LC	liquid chromatography
LDH	lactate dehydrogenase
mg	milligram
min	minute
mL	milliliter

Approved v1.0 [redacted]

Term	Definition
mmHg	millimeters of mercury
MPR	major pathological response
MLND	mediastinal lymph node dissection
µg	microgram
N	number of subjects or observations
Na^+	sodium
N/A	not applicable
NCI CTCAE	National Cancer Institute Common Terminology Criteria for Adverse Events
NSCLC	non-small cell lung carcinoma
NIMP	non-investigational medicinal products
OS	overall survival
PBMC	peripheral blood mononuclear cells
pCR	complete pathological response
PK	pharmacokinetics
PPK	Population pharmacokinetic
PO	per os (by mouth route of administration)
R&D	Research and Development
RCC	renal cell carcinoma
RECIST	Response Evaluation Criteria in Solid Tumors
SAE	serious adverse event
SCCHN	squamous cell carcinoma of the head and neck
SD	standard deviation
SOC	Standard of care
SOP	Standard Operating Procedures
t	temperature
[redacted]	
WHO	World Health Organization
WOCBP	women of childbearing potential

Approved v1.0 [redacted]

APPENDIX 3 ECOG PERFORMANCE STATUS

ECOG PERFORMANCE STATUS [a]	
0	Fully active, able to carry on all pre-disease performance without restriction
1	Restricted in physically strenuous activity but ambulatory and able to carry out work of a light or sedentary nature, eg, light house work, office work
2	Ambulatory and capable of all selfcare but unable to carry out any work activities. Up and about more than 50% of waking hours
3	Capable of only limited selfcare, confined to bed or chair more than 50% of waking hours
4	Completely disabled. Cannot carry on any selfcare. Totally confined to bed or chair
5	Dead

[a] Oken MM, Creech RH, Tormey DC, Horton J, Davis TE, McFadden ET, and Carbone PP. Toxicity and Response Criteria of the Eastern Cooperative Oncology Group. Am J Clin Oncol 1982; 5: 649-655.

Approved v1.0 [redacted]

APPENDIX 4 MANAGEMENT ALGORITHMS

These general guidelines constitute guidance to the Investigator and may be supplemented by discussions with the Medical Monitor representing the Sponsor. The guidance applies to all immuno-oncology agents and regimens.

A general principle is that differential diagnoses should be diligently evaluated according to standard medical practice. Non-inflammatory etiologies should be considered and appropriately treated.

Corticosteroids are a primary therapy for immuno-oncology drug-related adverse events. The oral equivalent of the recommended IV doses may be considered for ambulatory patients with low-grade toxicity. The lower bioavailability of oral corticosteroids should be taken into account when switching to the equivalent dose of oral corticosteroids.

Consultation with a medical or surgical specialist, especially prior to an invasive diagnostic or therapeutic procedure, is recommended.

The frequency and severity of the related adverse events covered by these algorithms will depend on the immuno-oncology agent or regimen being used.

Approved v1.0 [redacted]

GI Adverse Event Management Algorithm

Rule out non-inflammatory causes. If non-inflammatory cause is identified, treat accordingly and continue I-O therapy. Opiates/narcotics may mask symptoms of perforation. Infliximab should not be used in cases of perforation or sepsis.

Grade of Diarrhea/ Colitis (NCI CTCAE v4)	Management	Follow-up
**Grade 1**<br>Diarrhea: < 4 stools/day over baseline;<br>Colitis: asymptomatic	• Continue I-O therapy per protocol<br>• Symptomatic treatment	• Close monitoring for worsening symptoms.<br>• Educate patient to report worsening immediately<br>If worsens:<br>• Treat as Grade 2 or 3/4
**Grade 2**<br>Diarrhea: 4-6 stools per day over baseline; IV fluids indicated <24 hrs; not interfering with ADL<br>Colitis: abdominal pain; blood in stool	• Delay I-O therapy per protocol<br>• Symptomatic treatment	If improves to grade 1:<br>• Resume I-O therapy per protocol<br>If persists > 5-7 days or recur:<br>• 0.5-1.0 mg/kg/day methylprednisolone or oral equivalent<br>• When symptoms improve to grade 1, taper steroids over at least 1 month, consider prophylactic antibiotics for opportunistic infections, and resume I-O therapy per protocol.<br>If worsens or persists > 3-5 days with oral steroids:<br>• Treat as grade 3/4
**Grade 3-4**<br>Diarrhea (G3): ≥7 stools per day over baseline; incontinence; IV fluids ≥24 hrs; interfering with ADL<br>Colitis (G3): severe abdominal pain, medical intervention indicated, peritoneal signs<br>G4: life-threatening, perforation	• Discontinue I-O therapy per protocol<br>• 1.0 to 2.0 mg/kg/day methylprednisolone IV or IV equivalent<br>• Add prophylactic antibiotics for opportunistic infections<br>• Consider lower endoscopy	If improves:<br>• Continue steroids until grade 1, then taper over at least 1 month<br>If persists > 3-5 days, or recurs after improvement:<br>• Add infliximab 5 mg/kg (if no contraindication). Note: Infliximab should not be used in cases of perforation or sepsis

Patients on IV steroids may be switched to an equivalent dose of oral corticosteroids (e.g. prednisone) at start of tapering or earlier, once sustained clinical improvement is observed. Lower bioavailability of oral corticosteroids should be taken into account when switching to the equivalent dose of oral corticosteroids.

Updated 05-Jul-2016

Renal Adverse Event Management Algorithm

Rule out non-inflammatory causes. If non-inflammatory cause, treat accordingly and continue I-O therapy

Grade of Creatinine Elevation (NCI CTCAE v4)	Management	Follow-up
**Grade 1**<br>Creatinine > ULN and > than baseline but ≤ 1.5x baseline	• Continue I-O therapy per protocol<br>• Monitor creatinine weekly	<u>If returns to baseline</u>:<br>•Resume routine creatinine monitoring per protocol<br><u>If worsens:</u><br>•Treat as Grade 2 or 3/4
**Grade 2-3**<br>Creatinine > 1.5x baseline to ≤ 6x ULN	• Delay I-O therapy per protocol<br>• Monitor creatinine every 2-3 days<br>• 0.5 to 1.0 mg/kg/day methylprednisolone IV or oral equivalent<br>• Consider renal biopsy with nephrology consult	<u>If returns to Grade 1:</u><br>•Taper steroids over at least 1 month, consider prophylactic antibiotics for opportunistic infections, and resume I-O therapy and routine creatinine monitoring per protocol<br><br><u>If elevations persist > 7 days or worsen:</u><br>•Treat as Grade 4
**Grade 4**<br>Creatinine > 6x ULN	• Discontinue I-O therapy per protocol<br>• Monitor creatinine daily<br>• 1.0-2.0 mg/kg/day methylprednisolone IV or IV equivalent<br>• Consult nephrologist<br>• Consider renal biopsy	<u>If returns to Grade 1 :</u> Taper steroids over at least 1 month and add prophylactic antibiotics for opportunistic infections

Patients on IV steroids may be switched to an equivalent dose of oral corticosteroids (e.g. prednisone) at start of tapering or earlier, once sustained clinical improvement is observed. Lower bioavailability of oral corticosteroids should be taken into account when switching to the equivalent dose of oral corticosteroids.

Updated 05-Jul-2016

Pulmonary Adverse Event Management Algorithm

Rule out non-inflammatory causes. If non-inflammatory cause, treat accordingly and continue I-O therapy. Evaluate with imaging and pulmonary consultation.

Grade of Pneumonitis (NCI CTCAE v4)	Management	Follow-up
**Grade 1**<br>Radiographic changes only	• Consider delay of I-O therapy<br>• Monitor for symptoms every 2-3 days<br>• Consider Pulmonary and ID consults	• Re-image at least every 3 weeks<br>If worsens:<br>• Treat as Grade 2 or 3-4
**Grade 2**<br>Mild to moderate new symptoms	• Delay I-O therapy per protocol<br>• Pulmonary and ID consults<br>• Monitor symptoms daily, consider hospitalization<br>• 1.0 mg/kg/day methyl-prednisolone IV or oral equivalent<br>• Consider bronchoscopy , lung biopsy	• Re-image every 1-3 days<br>If improves:<br>• When symptoms return to near baseline, taper steroids over at least 1 month and then resume I-O therapy per protocol and consider prophylactic antibiotics<br>If not improving after 2 weeks or worsening:<br>• Treat as Grade 3-4
**Grade 3-4**<br>Severe new symptoms;<br>New/worsening hypoxia;<br>Life-threatening	• Discontinue I-O therapy per protocol<br>• Hospitalize<br>• Pulmonary and ID consults<br>• 2-4 mg/kg/day methylprednisolone IV or IV equivalent<br>• Add prophylactic antibiotics for opportunistic infections<br>• Consider bronchoscopy, lung biopsy	If improves to baseline:<br>• Taper steroids over at least 6 weeks<br>If not improving after 48 hours or worsening:<br>• Add additional immunosuppression

Patients on IV steroids may be switched to an equivalent dose of oral corticosteroids (e.g. prednisone) at start of tapering or earlier, once sustained clinical improvement is observed. Lower bioavailability of oral corticosteroids should be taken into account when switching to the equivalent dose of oral corticosteroids.

Updated 05-Jul-2016

Hepatic Adverse Event Management Algorithm

Rule out non-inflammatory causes. If non-inflammatory cause, treat accordingly and continue I-O therapy. Consider imaging for obstruction.

Grade of Liver Test Elevation (NCI CTCAE v4)	Management	Follow-up
**Grade 1**<br>AST or ALT > ULN to 3.0 x ULN <u>and/or</u> T. bili > ULN to 1.5 x ULN	• Continue I-O therapy per protocol	• Continue LFT monitoring per protocol<br><u>If worsens:</u><br>• Treat as Grade 2 or 3-4
**Grade 2**<br>AST or ALT > 3.0 to ≤ 5 x ULN <u>and/or</u> T. bili > 1.5 to ≤ 3 x ULN	• Delay I-O therapy per protocol<br>• Increase frequency of monitoring to every 3 days	<u>If returns to baseline</u>:<br>•Resume routine monitoring, resume I-O therapy per protocol<br><br><u>If elevations persist > 5-7 days or worsen :</u><br>• 0.5-1 mg/kg/day methylprednisolone or oral equivalent and when LFT returns to grade 1 or baseline, taper steroids over at least 1 month, consider prophylactic antibiotics for opportunistic infections, and resume I-O therapy per protocol
**Grade 3-4**<br>AST or ALT > 5 x ULN <u>or</u> T.bili >3 x ULN	• Discontinue I-O therapy*<br>• Increase frequency of monitoring to every 1-2 days<br>• 1.0 to 2.0 mg/kg/day methylprednisolone IV or IV equivalent**<br>• Add prophylactic antibiotics for opportunistic infections<br>• Consult gastroenterologist	<u>If returns to grade 2</u>:<br>• Taper steroids over at least 1 month<br><br><u>If does not improve in >3-5 days, worsens or rebounds:</u><br>• Add mycophenolate mofetil 1 g BID<br>• If no response within an additional 3-5 days, consider other immunosuppressants per local guidelines

Patients on IV steroids may be switched to an equivalent dose of oral corticosteroids (e.g. prednisone) at start of tapering or earlier, once sustained clinical improvement is observed. Lower bioavailability of oral corticosteroids should be taken into account when switching to the equivalent dose of oral corticosteroids.
*I-O therapy may be delayed rather than discontinued if AST/ALT ≤ 8 x ULN or T.bili ≤ 5 x ULN.
**The recommended starting dose for grade 4 hepatitis is 2 mg/kg/day methylprednisolone IV.

Updated 05-Jul-2016

Endocrinopathy Management Algorithm

Rule out non-inflammatory causes. If non-inflammatory cause, treat accordingly and continue I-O therapy. Consider visual field testing, endocrinology consultation, and imaging.

Asymptomatic TSH elevation →

- Continue I-O therapy per protocol
- If TSH < 0.5 x LLN, or TSH > 2 x ULN, or consistently out of range in 2 subsequent measurements: include fT4 at subsequent cycles as clinically indicated; consider endocrinology consult

Symptomatic endocrinopathy →

- Evaluate endocrine function
- Consider pituitary scan

Symptomatic with abnormal lab/pituitary scan:

- Delay I-O therapy per protocol
- 1-2 mg/kg/day methylprednisolone IV or PO equivalent
- Initiate appropriate hormone therapy

No abnormal lab/pituitary MRI scan but symptoms persist:

- Repeat labs in 1-3 weeks / MRI in 1 month

→ If improves (with or without hormone replacement):

- Taper steroids over at least 1 month and consider prophylactic antibiotics for opportunistic infections
- Resume I-O therapy per protocol
- Patients with adrenal insufficiency may need to continue steroids with mineralocorticoid component

Suspicion of adrenal crisis (e.g. severe dehydration, hypotension, shock out of proportion to current illness →

- Delay or discontinue I-O therapy per protocol
- Rule out sepsis
- Stress dose of IV steroids with mineralocorticoid activity
- IV fluids
- Consult endocrinologist
- If adrenal crisis ruled out, then treat as above for symptomatic endocrinopathy

Patients on IV steroids may be switched to an equivalent dose of oral corticosteroids (e.g. prednisone) at start of tapering or earlier, once sustained clinical improvement is observed. Lower bioavailability of oral corticosteroids should be taken into account when switching to the equivalent dose of oral corticosteroids.

Updated 05-Jul-2016

Approved v1.0 [redacted]

Skin Adverse Event Management Algorithm

Rule out non-inflammatory causes. If non-inflammatory cause, treat accordingly and continue I-O therapy.

Grade of Rash (NCI CTCAE v4)	Management	Follow-up
**Grade 1-2**<br>Covering ≤ 30% BSA*	• Symptomatic therapy (e.g. antihistamines, topical steroids)<br>• Continue I-O therapy per protocol	<u>If persists > 1-2 weeks or recurs:</u><br>• Consider skin biopsy<br>• Delay I-O therapy per protocol<br>• Consider 0.5-1.0 mg/kg/day methylprednisolone IV or oral equivalent. Once improving , taper steroids over at least 1 month, consider prophylactic antibiotics for opportunistic infections, and resume I-O therapy per protocol<br><u>If worsens:</u><br>• Treat as Grade 3-4
**Grade 3-4**<br>Covering >30% BSA; Life threatening consequences*^	• Delay or discontinue I-O therapy per protocol<br>• Consider skin biopsy<br>• Dermatology consult<br>• 1.0-2.0 mg/kg/day IV methylprednisolone IV or IV equivalent	<u>If improves to Grade 1:</u><br>• Taper steroids over at least 1 month and add prophylactic antibiotics for opportunistic infections<br>• Resume I-O therapy per protocol

Patients on IV steroids may be switched to an equivalent dose of oral corticosteroids (e.g. prednisone) at start of tapering or earlier, once sustained clinical improvement is observed. Lower bioavailability of oral corticosteroids should be taken into account when switching to the equivalent dose of oral corticosteroids.
*Refer to NCI CTCAE v4 for term-specific grading criteria.
^If SJS/TEN is suspected, withhold I-O therapy and refer patient for specialized care for assessment and treatment. If SJS or TEN is diagnosed, permanently discontinue I-O therapy.

Updated 05-Jul-2016

Neurological Adverse Event Management Algorithm

Rule out non-inflammatory causes. If non-inflammatory cause, treat accordingly and continue I-O therapy.

Grade of Neurological Toxicity (NCI CTCAE v4)	Management	Follow-up
**Grade 1**<br>Asymptomatic or mild symptoms; Intervention not indicated	• Continue I-O therapy per protocol	Continue to monitor the patient.<br>If worsens:<br>• Treat as Grade 2 or 3-4
**Grade 2**<br>Moderate symptoms; Limiting instrumental ADL	• Delay I-O therapy per protocol<br>• Treat symptoms per local guidelines<br>• Consider 0.5 to 1.0 mg/kg/day methylprednisolone IV or PO equivalent	If improves to baseline:<br>•Resume I-O therapy per protocol when improved to baseline<br>If worsens:<br>• Treat as Grade 3-4
**Grade 3-4**<br>Severe symptoms; Limiting self-care ADL; Life-threatening	• Discontinue I-O therapy per protocol<br>• Obtain neurology consult<br>• Treat symptoms per local guidelines<br>• 1.0-2.0 mg/kg/day IV methylprednisolone IV or IV equivalent<br>• Add prophylactic antibiotics for opportunistic infections	If improves to Grade 2:<br>• Taper steroids over at least 1 month<br>If worsens or atypical presentation:<br>• Consider IVIG or other immunosuppressive therapies per local guidelines

Patients on IV steroids may be switched to an equivalent dose of oral corticosteroids (e.g. prednisone) at start of tapering or earlier, once sustained clinical improvement is observed. Lower bioavailability of oral corticosteroids should be taken into account when switching to the equivalent dose of oral corticosteroids.

Updated 05-Jul-2016

APPENDIX 5 RESPONSE CRITERIA (RECIST 1.1)

1 EVALUATION OF LESIONS

At baseline, tumor lesions/lymph nodes will be categorized measurable or non-measurable as follows:

1.1 Measurable

Tumor lesions: Must be accurately measured in at least one dimension (longest diameter in the plane of measurement is to be recorded) with a minimum size of:

1) 10 mm by CT scan (CT scan slice thickness no greater than 5 mm)
2) 10 mm caliper measurement by clinical exam (lesions which cannot be accurately measured with calipers should be recorded as non-measurable)
3) 20 mm by chest x-ray

Malignant lymph nodes: To be considered pathologically enlarged and measurable, a lymph node must be ≥ 15 mm in short axis when assessed by CT scan (CT scan slice thickness recommended to be no greater than 5 mm).

Lymph nodes merit special mention since they are normal anatomical structures which may be visible by imaging even if not involved by tumor. Pathological nodes which are defined as measurable and may be identified as target lesions must meet the criterion of a short axis of ≥ 15 mm by CT scan. Only the short axis of these nodes will contribute to the baseline sum. The short axis of the node is the diameter normally used by radiologists to judge if a node is involved by solid tumor. Nodal size is normally reported as two dimensions in the plane in which the image is obtained (for CT scan this is almost always the axial plane; for MRI the plane of acquisition may be axial, saggital or coronal). The smaller of these measures is the short axis. For example, an abdominal node which is reported as being 20 mm x 30 mm has a short axis of 20 mm and qualifies as a malignant, measurable node. In this example, 20 mm should be recorded as the node measurement. All other pathological nodes (those with short axis ≥ 10 mm but < 15 mm) should be considered non-target lesions. Nodes that have a short axis < 10 mm are considered non-pathological and should not be recorded or followed.

1.2 Non-Measurable

All other lesions are considered non-measurable, including small lesions (longest diameter < 10mm or pathological lymph nodes with ≥ 10 to < 15 mm short axis) as well as truly non-measurable lesions. Lesions considered truly non-measurable include: leptomeningeal disease, ascites, pleural or pericardial effusion, inflammatory breast disease, lymphangitic involvement of skin or lung, abdominal masses/abdominal organomegaly identified by physical exam that is not measurable by reproducible imaging techniques.

Approved v1.0 [redacted]

2 BASELINE DOCUMENTATION OF 'TARGET' AND 'NON-TARGET' LESIONS

When more than one measurable lesion is present at baseline all lesions up to a maximum of five lesions total (and a maximum of two lesions per organ) representative of all involved organs should be identified as target lesions and will be recorded and measured at baseline (this means in instances where patients have only one or two organ sites involved a maximum of two and four lesions respectively will be recorded).

Target lesions should be selected on the basis of their size (lesions with the longest diameter), be representative of all involved organs, but in addition should be those that lend themselves to reproducible repeated measurements. It may be the case that, on occasion, the largest lesion does not lend itself to reproducible measurement in which circumstance the next largest lesion which can be measured reproducibly should be selected.

A sum of the diameters (longest for non-nodal lesions, short axis for nodal lesions) for all target lesions will be calculated and reported as the baseline sum diameters. If lymph nodes are to be included in the sum, then as noted above, only the short axis is added into the sum. The baseline sum diameters will be used as reference to further characterize any objective tumor regression in the measurable dimension of the disease.

All other lesions (or sites of disease) including pathological lymph nodes should be identified as non-target lesions and should also be recorded at baseline. Measurements are not required and these lesions should be followed as 'present', 'absent', or in rare cases 'unequivocal progression' (more details to follow). In addition, it is possible to record multiple nontarget lesions involving the same organ as a single item on the case record form (eg, 'multiple enlarged pelvic lymph nodes' or 'multiple liver metastases').

3 RESPONSE CRITERIA

3.1 Evaluation of Target Lesions

Complete Response (CR): Disappearance of all target lesions. Any pathological lymph nodes (whether target or non-target) must have reduction in short axis to < 10 mm.

Partial Response (PR): At least a 30% decrease in the sum of diameters of target lesions, taking as reference the baseline sum diameters.

Progressive Disease (PD): At least a 20% increase in the sum of diameters of target lesions, taking as reference the smallest sum on study (this includes the baseline sum if that is the smallest on study). In addition to the relative increase of 20%, the sum must also demonstrate an absolute increase of at least 5 mm. (Note: the appearance of one or more new lesions is also considered progression).

Stable Disease (SD): Neither sufficient shrinkage to qualify for PR nor sufficient increase to qualify for PD, taking as reference the smallest sum diameters while on study.

3.1.1 Special Notes on the Assessment of Target Lesions

3.1.1.1 Lymph nodes

Lymph nodes identified as target lesions should always have the actual short axis measurement recorded (measured in the same anatomical plane as the baseline examination), even if the nodes regress to below 10 mm on study. This means that when lymph nodes are included as target lesions, the ‘sum’ of lesions may not be zero even if complete response criteria are met, since a normal lymph node is defined as having a short axis of < 10 mm. Case report forms or other data collection methods may therefore be designed to have target nodal lesions recorded in a separate section where, in order to qualify for CR, each node must achieve a short axis < 10 mm. For PR, SD and PD, the actual short axis measurement of the nodes is to be included in the sum of target lesions.

3.1.1.2 Target lesions that become ‘too small to measure’

While on study, all lesions (nodal and non-nodal) recorded at baseline should have their actual measurements recorded at each subsequent evaluation, even when very small (eg, 2 mm). However, sometimes lesions or lymph nodes which are recorded as target lesions at baseline become so faint on CT scan that the radiologist may not feel comfortable assigning an exact measure and may report them as being ‘too small to measure’. When this occurs it is important that a value be recorded on the case report form. If it is the opinion of the radiologist that the lesion has likely disappeared, the measurement should be recorded as 0 mm. If the lesion is believed to be present and is faintly seen but too small to measure, a default value of 5 mm should be assigned (Note: It is less likely that this rule will be used for lymph nodes since they usually have a definable size when normal and are frequently surrounded by fat such as in the retroperitoneum; however, if a lymph node is believed to be present and is faintly seen but too small to measure, a default value of 5 mm should be assigned in this circumstance as well). This default value is derived from the 5 mm CT slice thickness (but should not be changed with varying CT slice thickness). The measurement of these lesions is potentially non-reproducible, therefore providing this default value will prevent false responses or progressions based upon measurement error. To reiterate, however, if the radiologist is able to provide an actual measure, that should be recorded, even if it is below 5 mm.

3.1.1.3 Lesions that split or coalesce on treatment

When non-nodal lesions ‘fragment’, the longest diameters of the fragmented portions should be added together to calculate the target lesion sum. Similarly, as lesions coalesce, a plane between them may be maintained that would aid in obtaining maximal diameter measurements of each individual lesion. If the lesions have truly coalesced such that they are no longer separable, the vector of the longest diameter in this instance should be the maximal longest diameter for the ‘coalesced lesion’.

3.2 Evaluation of Non-Target Lesions

This section provides the definitions of the criteria used to determine the tumor response for the group of non-target lesions. While some non-target lesions may actually be measurable, they

Approved v1.0 [redacted]

need not be measured and instead should be assessed only qualitatively at the time points specified in the protocol.

Complete Response (CR): Disappearance of all non-target lesions and normalization of tumor marker level. All lymph nodes must be non-pathological in size (< 10mm short axis).

Non-CR/Non-PD: Persistence of one or more non-target lesion(s) and/or maintenance of tumor marker level above the normal limits.

Progressive Disease (PD): Unequivocal progression (see comments below) of existing non-target lesions. (Note: the appearance of one or more new lesions is also considered progression).

3.2.1 Special Notes on Assessment of Progression of Non-Target Disease

The concept of progression of non-target disease requires additional explanation as follows:

3.2.1.1 When the patient also has measurable disease

In this setting, to achieve ‘unequivocal progression’ on the basis of the non-target disease, there must be an overall level of substantial worsening in non-target disease such that, even in presence of SD or PR in target disease, the overall tumor burden has increased sufficiently to merit discontinuation of therapy (see examples in Appendix 2 and further details below). A modest ‘increase’ in the size of one or more non-target lesions is usually not sufficient to qualify for unequivocal progression status. The designation of overall progression solely on the basis of change in non-target disease in the face of SD or PR of target disease will therefore be extremely rare.

3.2.1.2 When the patient has only non-measurable disease

This circumstance arises in some trials when it is not a criterion of study entry to have measurable disease. The same general concepts apply here as noted above, however, in this instance there is no measurable disease assessment to factor into the interpretation of an increase in non-measurable disease burden. Because worsening in non-target disease cannot be easily quantified (by definition: if all lesions are truly non-measurable) a useful test that can be applied when assessing patients for unequivocal progression is to consider if the increase in overall disease burden based on the change in non-measurable disease is comparable in magnitude to the increase that would be required to declare PD for measurable disease: ie, an increase in tumor burden representing an additional 73% increase in ‘volume’ (which is equivalent to a 20% increase diameter in a measurable lesion). Examples include an increase in a pleural effusion from ‘trace’ to ‘large’, an increase in lymphangitic disease from localized to widespread, or may be described in protocols as ‘sufficient to require a change in therapy’. If ‘unequivocal progression’ is seen, the patient should be considered to have had overall PD at that point. While it would be ideal to have objective criteria to apply to non-measurable disease, the very nature of that disease makes it impossible to do so; therefore the increase must be substantial.

3.2.2 New Lesions

The appearance of new malignant lesions denotes disease progression; therefore, some comments on detection of new lesions are important. There are no specific criteria for the

identification of new radiographic lesions; however, the finding of a new lesion should be unequivocal: ie, not attributable to differences in scanning technique, change in imaging modality or findings thought to represent something other than tumor (for example, some 'new' bone lesions may be simply healing or flare of pre-existing lesions). This is particularly important when the patient's baseline lesions show partial or complete response. For example, necrosis of a liver lesion may be reported on a CT scan report as a 'new' cystic lesion, which it is not.

A lesion identified on a follow-up study in an anatomical location that was not scanned at baseline is considered a new lesion and will indicate disease progression. An example of this is the patient who has visceral disease at baseline and while on study has a CT or MRI brain ordered which reveals metastases. The patient's brain metastases are considered to be evidence of PD even if he/she did not have brain imaging at baseline.

If a new lesion is equivocal, for example because of its small size, continued therapy and follow-up evaluation will clarify if it represents truly new disease. If repeat scans confirm there is definitely a new lesion, then progression should be declared using the date of the initial scan. While FDG-PET response assessments need additional study, it is sometimes reasonable to incorporate the use of FDG-PET scanning to complement CT scanning in assessment of progression (particularly possible 'new' disease). New lesions on the basis of FDG-PET imaging can be identified according to the following algorithm:

1) Negative FDG-PET at baseline, with a positive FDG-PET at follow-up is a sign of PD based on a new lesion.
2) No FDG-PET at baseline and a positive FDG-PET at follow-up: If the positive FDG-PET at follow-up corresponds to a new site of disease confirmed by CT, this is PD. If the positive FDG-PET at follow-up is not confirmed as a new site of disease on CT, additional follow-up CT scans are needed to determine if there is truly progression occurring at that site (if so, the date of PD will be the date of the initial abnormal FDG-PET scan). If the positive FDG-PET at follow-up corresponds to a pre-existing site of disease on CT that is not progressing on the basis of the anatomic images, this is not PD.

3.3 Response Assessment

3.3.1 Evaluation of Best Overall Response

The best overall response is the best response recorded from the start of the study treatment until the end of treatment taking into account any requirement for confirmation. The patient's best overall response assignment will depend on the findings of both target and non-target disease and will also take into consideration the appearance of new lesions. Furthermore, depending on the nature of the study and the protocol requirements, it may also require confirmatory measurement.

3.3.2 Time Point Response

It is assumed that at each protocol specified time point, a response assessment occurs. Table 3.3.2-1 provides a summary of the overall response status calculation at each time point for

Approved v1.0 [redacted]

patients who have measurable disease at baseline. When patients have non-measurable (therefore non-target) disease only, Table 3.3.2-2 is to be used.

Table 3.3.2-1: Time Point Response - Patients With Target (+/- Non-Target) Disease

Target Lesions	Non-Target Lesions	New Lesions	Overall Response
CR	CR	No	CR
CR	Non-CR/non-PD	No	PR
CR	Not evaluated	No	PR
PR	Non-PD or not all evaluated	No	PR
SD	Non-PD or not all evaluated	No	SD
Not all evaluated	Non-PD	No	NE
PD	Any	Yes or No	PD
Any	PD	Yes or No	PD
Any	Any	Yes	PD

CR = complete response, PR = partial response, SD = stable disease, PD = progressive disease and NE = inevaluable

Table 3.3.2-2: Time Point Response - Patients with Non-target Disease Only

Non-Target Lesions	New Lesions	Overall Response
CR	No	CR
Non-CR/non-PD	No	Non-CR/non-PD[a]
Not all evaluated	No	NE
Unequivocal PD	Yes or No	PD
Any	Yes	PD
CR = complete response, PD = progressive disease and NE = inevaluable		

[a] Non-CR/non-PD is preferred over SD for non-target disease since SD is increasingly used as endpoint for assessment of efficacy in some trials so to assign this category when no lesions can be measured is not advised.

3.3.3 Best Overall Response

Best response determination of complete or partial response requires confirmation: Complete or partial responses may be claimed only if the criteria for each are met at a subsequent time point of ≥ 4 weeks later. In this circumstance, the best overall response can be interpreted as in Table 3.3.3-1.

Special note on response assessment: When nodal disease is included in the sum of target lesions and the nodes decrease to 'normal' size (< 10 mm), they may still have a measurement reported on scans. This measurement should be recorded even though the nodes are normal in order not to overstate progression should it be based on increase in size of the nodes. As noted earlier, this means that patients with CR may not have a total sum of 'zero' on the case report form (CRF).

Table 3.3.3-1: Best Overall Response (Confirmation of CR&PR Required)

Overall Response First Time Point	Overall Response Subsequent Time Point	BEST Overall Response
CR	CR	CR
CR	PR	SD, PD OR PR[a]
CR	SD	SD provided minimum criteria for SD duration[b] met, otherwise, PD
CR	PD	SD provided minimum criteria for SD duration[b] met, otherwise, PD
CR	NE	SD provided minimum criteria for SD duration[b] met, otherwise, NE
PR	CR	PR
PR	PR	PR
PR	SD	SD
PR	PD	SD provided minimum criteria for SD duration[b] met, otherwise, PD
PR	NE	SD provided minimum criteria for SD duration [b]met, otherwise, NE
NE	NE	NE
CR = complete response, PR = partial response, SD = stable disease, PD = progressive disease, and NE = inevaluable		

[a] If a CR is truly met at first time point, then any disease seen at a subsequent time point, even disease meeting PR criteria relative to baseline, makes the disease PD at that point (since disease must have reappeared after CR). Best response would depend on whether minimum duration for SD was met. However, sometimes 'CR' may be claimed when subsequent scans suggest small lesions were likely still present and in fact the patient had PR, not CR at the first time point. Under these circumstances, the original CR should be changed to PR and the best response is PR.

[b] Minimum criteria for SD duration is 6 weeks.

3.3.4 Confirmation Scans

<u>Verification of Response:</u> To be assigned a status of CR or PR, changes in tumor measurements must be confirmed by consecutive repeat assessments that should be performed no less than

Approved v1.0 [redacted]

28 days after the criteria for response are first met. For this study, the next scheduled tumor assessment can meet this requirement.

<u>Verification of Progression:</u> Progression of disease should be verified in cases where progression is equivocal. If repeat scans confirm PD, then progression should be declared using the date of the initial scan. If repeat scans do not confirm PD, then the subject is considered to not have progressive disease.

Approved v1.0 [redacted]

APPENDIX 6 WOMEN OF CHILDBEARING POTENTIAL DEFINITIONS AND METHODS OF CONTRACEPTION

DEFINITIONS

Woman of Childbearing Potential (WOCBP)

A woman is considered fertile following menarche and until becoming post-menopausal unless permanently sterile. Permanent sterilization methods include hysterectomy, bilateral salpingectomy, and bilateral oophorectomy.

Women in the following categories are not considered WOCBP

- Premenarchal
- Premenopausal female with 1 of the following:
  - Documented hysterectomy
  - Documented bilateral salpingectomy
  - Documented bilateral oophorectomy

Note: Documentation can come from the site personnel's review of the participant's medical records, medical examination, or medical history interview.

- Postmenopausal female
  - A postmenopausal state is defined as 12 months of amenorrhea in a woman over age 45 years in the absence of other biological or physiological causes. In addition, females under the age of 55 years must have a serum follicle stimulating hormone, (FSH) level > 40 mIU/mL to confirm menopause.

CONTRACEPTION GUIDANCE FOR FEMALE PARTICIPANTS OF CHILD BEARING POTENTIAL

One of the highly effective methods of contraception listed below is required during study duration and until the end of relevant systemic exposure, defined as 4 months after the end of study treatment, plus 1 month.

Local laws and regulations may require use of alternative and/or additional contraception methods.

**Highly Effective Contraceptive Methods That Are User Dependent**<br>*Failure rate of <1% per year when used consistently and correctly.*[a]
• Combined (estrogen- and progestogen-containing) hormonal contraception associated with inhibition of ovulation[b]<br>– oral<br>– intravaginal
**Highly Effective Methods That Are User Independent**

• Implantable progestogen-only hormonal contraception associated with inhibition of ovulation [b]<br>• Intrauterine device (IUD)[c]<br>• Intrauterine hormone-releasing system (IUS)[c]<br>• Bilateral tubal occlusion
• Vasectomized partner<br>*A vasectomized partner is a highly effective contraception method provided that the partner is the sole male sexual partner of the WOCBP and the absence of sperm has been confirmed. If not, an additional highly effective method of contraception should be used.*
• Sexual abstinence<br>*Sexual abstinence is considered a highly effective method only if defined as refraining from heterosexual intercourse during the entire period of risk associated with the study drug. The reliability of sexual abstinence needs to be evaluated in relation to the duration of the study and the preferred and usual lifestyle of the participant.*<br>• It is not necessary to use any other method of contraception when complete abstinence is elected.<br>• WOCBP participants who choose complete abstinence must continue to have pregnancy tests, as specified in Section 2.<br>• Acceptable alternate methods of highly effective contraception must be discussed in the event that the WOCBP participants chooses to forego complete abstinence
NOTES:<br>[a] Typical use failure rates may differ from those when used consistently and correctly. Use should be consistent with local regulations regarding the use of contraceptive methods for participants participating in clinical studies.<br>[b] Hormonal contraception may be susceptible to interaction with the study drug, which may reduce the efficacy of the contraceptive method. Hormonal contraception is permissible only when there is sufficient evidence that the IMP and other study medications will not alter hormonal exposures such that contraception would be ineffective or result in increased exposures that could be potentially hazardous. In this case, alternative methods of contraception should be utilized.<br>[c] Intrauterine devices and intrauterine hormone releasing systems are acceptable methods of contraception in the absence of definitive drug interaction studies when hormone exposures from intrauterine devices do not alter contraception effectiveness

**Unacceptable Methods of Contraception**
• Periodic abstinence (calendar, symptothermal, post-ovulation methods)<br>• Withdrawal(coitus interruptus).<br>• Spermicide only<br>• Lactation amenorrhea method (LAM)

Approved v1.0 [redacted]

CONTRACEPTION GUIDANCE FOR MALE PARTICIPANTS WITH PARTNER(S) OF CHILD BEARING POTENTIAL.

Male participants with female partners of childbearing potential are eligible to participate if they agree to the following during the treatment and until the end of relevant systemic exposure.

- Inform any and all partner(s) of their participation in a clinical drug study and the need to comply with contraception instructions as directed by the investigator.
- Male participants are required to use a condom for study duration and until the end of relevant systemic exposure defined as 4 months after the end of treatment in the male participant, plus 3 months.
- Female partners of males participating in the study to consider use of effective methods of contraception until the end of relevant systemic exposure, defined as 4 months after the end of treatment in the male participant, plus 1 month after the end of treatment in the female participant.
- Male participants with a pregnant or breastfeeding partner must agree to remain abstinent from penile vaginal intercourse or use a male condom during each episode of penile penetration during the treatment and until 4 months after the end of treatment plus 3 months.
- Refrain from donating sperm for the duration of the study treatment and until 4 months after the end of treatment plus 3 months.

COLLECTION OF PREGNANCY INFORMATION

Guidance for collection of Pregnancy Information and outcome of pregnancy on the Pregnancy Surveillance Form is provided in Section 9.2.5 and the Appendix for Adverse Events and Serious Adverse Events Definitions and procedures for Evaluating, Follow-up and Reporting

APPENDIX 7 STUDY GOVERANCE CONSIDERATIONS

The term 'Participant' is used in the protocol to refer to a person who has consented to participate in the clinical research study. The term 'Subject' used in the eCRF is intended to refer to a person (Participant) who has consented to participate in the clinical research study.

REGULATORY AND ETHICAL CONSIDERATIONS

GOOD CLINICAL PRACTICE

This study will be conducted in accordance with:

- Good Clinical Practice (GCP),
- as defined by the International Council on Harmonisation (ICH)
- in accordance with the ethical principles underlying European Union Directive 2001/20/EC
- United States Code of Federal Regulations, Title 21, Part 50 (21CFR50)
- applicable local requirements.

The study will be conducted in compliance with the protocol. The protocol and any amendments and the participant informed consent will receive approval/favorable opinion by Institutional Review Board/Independent Ethics Committee (IRB/IEC), and regulatory authorities according to applicable local regulations prior to initiation of the study.

All potential serious breaches must be reported to Sponsor or designee immediately. A serious breach is a breach of the conditions and principles of GCP in connection with the study or the protocol, which is likely to affect, to a significant degree, the safety or physical or mental integrity of the subjects of the study or the scientific value of the study.

Personnel involved in conducting this study will be qualified by education, training, and experience to perform their respective tasks.

This study will not use the services of study personnel where sanctions have been invoked or where there has been scientific misconduct or fraud (e.g., loss of medical licensure, debarment).

INSTITUTIONAL REVIEW BOARD/INDEPENDENT ETHICS COMMITTEE

Before study initiation, the investigator must have written and dated approval/favorable opinion from the IRB/IEC for the protocol, consent form, participant recruitment materials (e.g., advertisements), and any other written information to be provided to subjects. The investigator or BMS should also provide the IRB/IEC with a copy of the Investigator Brochure or product labeling information to be provided to subjects and any updates.

The investigator, Sponsor or designee should provide the IRB/IEC with reports, updates and other information (e.g., expedited safety reports, amendments, and administrative letters) according to regulatory requirements or institution procedures.

Approved v1.0 [redacted]

COMPLIANCE WITH THE PROTOCOL AND PROTOCOL REVISIONS

The investigator should not implement any deviation or change to the protocol without prior review and documented approval/favorable opinion of an amendment from the IRB/IEC (and if applicable, also by local health authority) except where necessary to eliminate an immediate hazard(s) to study subjects.

If a deviation or change to a protocol is implemented to eliminate an immediate hazard(s) prior to obtaining relevant approval/favorable opinion(s) the deviation or change will be submitted, as soon as possible to:

- IRB/IEC for
- Regulatory Authority(ies), if applicable by local regulations (per national requirements)

Documentation of approval/favorable opinion signed by the chairperson or designee of the IRB(s)/IEC(s) and if applicable, also by local health authority must be sent to BMS.

If an amendment substantially alters the study design or increases the potential risk to the participant: (1) the consent form must be revised and submitted to the IRB(s)/IEC(s) for review and approval/favorable opinion; (2) the revised form must be used to obtain consent from subjects currently enrolled in the study if they are affected by the amendment; and (3) the new form must be used to obtain consent from new subjects prior to enrollment.

If the revision is done via an administrative letter, investigators must inform their IRB(s)/IEC(s).

FINANCIAL DISCLOSURE

Investigators and sub-Investigators will provide the Sponsor with sufficient, accurate financial information in accordance with local regulations to allow the Sponsor to submit complete and accurate financial certification or disclosure statements to the appropriate health authorities. Investigators are responsible for providing information on financial interests during the course of the study and for 1 year after completion of the study.

INFORMED CONSENT PROCESS

Investigators must ensure that subjects are clearly and fully informed about the purpose, potential risks, and other critical issues regarding clinical studies in which they volunteer to participate.

In situations where consent cannot be given to subjects, their legally acceptable representatives (as per country guidelines) are clearly and fully informed about the purpose, potential risks, and other critical issues regarding clinical studies in which the participant volunteers to participate.

Sponsor or designee will provide the investigator with an appropriate (i.e., Global or Local) sample informed consent form which will include all elements required by ICH, GCP and applicable regulatory requirements. The sample informed consent form will adhere to the ethical principles that have their origin in the Declaration of Helsinki.

Investigators must:

- Provide a copy of the consent form and written information about the study in the language in which the participant is most proficient prior to clinical study participation. The language must be non-technical and easily understood.
- Allow time necessary for participant or participant's legally acceptable representative to inquire about the details of the study.
- Obtain an informed consent signed and personally dated by the participant or the participant's legally acceptable representative and by the person who conducted the informed consent discussion.
- Obtain the IRB/IEC's written approval/favorable opinion of the written informed consent form and any other information to be provided to the subjects, prior to the beginning of the study, and after any revisions are completed for new information.

If informed consent is initially given by a participant's legally acceptable representative or legal guardian, and the participant subsequently becomes capable of making and communicating his or her informed consent during the study, consent must additionally be obtained from the participant.

Revise the informed consent whenever important new information becomes available that is relevant to the participant's consent. The investigator, or a person designated by the investigator, should fully inform the participant or the participant's legally acceptable representative or legal guardian, of all pertinent aspects of the study and of any new information relevant to the participant's willingness to continue participation in the study. This communication should be documented.

The confidentiality of records that could identify subjects must be protected, respecting the privacy and confidentiality rules applicable to regulatory requirements, the subjects' signed ICF and, in the US, the subjects' signed HIPAA Authorization.

The consent form must also include a statement that BMS and regulatory authorities have direct access to participant records.

Subjects unable to give their written consent (e.g., stroke or subjects with or severe dementia) may only be enrolled in the study with the consent of a legally acceptable representative. The participant must also be informed about the nature of the study to the extent compatible with his or her understanding, and should this participant become capable, he or she should personally sign and date the consent form as soon as possible. The explicit wish of a participant who is unable to give his or her written consent, but who is capable of forming an opinion and assessing information to refuse participation in, or to be withdrawn from, the clinical study at any time should be considered by the investigator.

The rights, safety, and well-being of the study subjects are the most important considerations and should prevail over interests of science and society.

Approved v1.0 [redacted]

SOURCE DOCUMENTS

The Investigator is responsible for ensuring that the source data are accurate, legible, contemporaneous, original and attributable, whether the data are hand-written on paper or entered electronically. If source data are created (first entered), modified, maintained, archived, retrieved, or transmitted electronically via computerized systems (and/or any other kind of electronic devices) as part of regulated clinical trial activities, such systems must be compliant with all applicable laws and regulations governing use of electronic records and/or electronic signatures. Such systems may include, but are not limited to, electronic medical/health records (EMRs/EHRs), adverse event tracking/reporting, protocol required assessments, and/or drug accountability records).

When paper records from such systems are used in place of electronic format to perform regulated activities, such paper records should be certified copies. A certified copy consists of a copy of original information that has been verified, as indicated by a dated signature, as an exact copy having all of the same attributes and information as the original.

STUDY TREATMENT RECORDS

Records for study treatments (whether supplied by BMS, its vendors, or the site) must substantiate study treatment integrity and traceability from receipt, preparation, administration, and through destruction or return. Records must be made available for review at the request of BMS/designee or a Health Authority.

If	Then
Supplied by BMS (or its vendors):	Records or logs must comply with applicable regulations and guidelines and should include:<br>• amount received and placed in storage area<br>• amount currently in storage area<br>• label identification number or batch number<br>• amount dispensed to and returned by each participant, including unique participant identifiers<br>• amount transferred to another area/site for dispensing or storage<br>• nonstudy disposition (e.g., lost, wasted)<br>• amount destroyed at study site, if applicable<br>• amount returned to BMS<br>• retain samples for bioavailability/bioequivalence, if applicable<br>• dates and initials of person responsible for Investigational Product dispensing/accountability, as per the Delegation of Authority Form.
Sourced by site, and not supplied by BMS or its vendors (examples include IP sourced from the sites stock or commercial supply, or a specialty pharmacy)	The investigator or designee accepts responsibility for documenting traceability and study drug integrity in accordance with requirements applicable under law and the SOPs/standards of the sourcing pharmacy.<br>These records should include:

Approved v1.0 [redacted]

If	Then
	• label identification number or batch number<br>• amount dispensed to and returned by each participant, including unique participant identifiers<br>• dates and initials of person responsible for Investigational Product dispensing/accountability, as per the Delegation of Authority Form.

BMS or designee will provide forms to facilitate inventory control if the investigational site does not have an established system that meets these requirements.

CASE REPORT FORMS

An investigator is required to prepare and maintain adequate and accurate case histories designed to record all observations and other data pertinent to the investigation on each individual treated or entered as a control in the investigation. Data that are derived from source documents and reported on the CRF must be consistent with the source documents or the discrepancies must be explained. Additional clinical information may be collected and analyzed in an effort to enhance understanding of product safety. CRFs may be requested for AEs and/or laboratory abnormalities that are reported or identified during the course of the study.

For sites using the Sponsor or designee electronic data capture tool, electronic CRFs will be prepared for all data collection fields except for fields specific to SAEs and pregnancy, which will be reported on the electronic SAE form and Pregnancy Surveillance form, respectively. If electronic SAE form is not available, a paper SAE form can be used. Spaces may be left blank only in those circumstances permitted by study-specific CRF completion guidelines provided by Sponsor or designee.

The confidentiality of records that could identify subjects must be protected, respecting the privacy and confidentiality rules in accordance with the applicable regulatory requirement(s).

The investigator will maintain a signature sheet to document signatures and initials of all persons authorized to make entries and/or corrections on CRFs.

The completed CRF, SAE/pregnancy CRFs, must be promptly reviewed, signed, and dated by the investigator or qualified physician who is a subinvestigator and who is delegated this task on the Delegation of Authority Form. Subinvestigators in Japan may not be delegated the CRF approval task For electronic CRFs, review and approval/signature is completed electronically through the BMS electronic data capture tool. The investigator must retain a copy of the CRFs including records of the changes and corrections.

Each individual electronically signing electronic CRFs must meet Sponsor or designee training requirements and must only access the BMS electronic data capture tool using the unique user account provided by Sponsor or designee. User accounts are not to be shared or reassigned to other individuals

MONITORING

Sponsor or designee representatives will review data centrally to identify potential issues to determine a schedule of on-site visits for targeted review of study records.

Representatives of BMS must be allowed to visit all study site locations periodically to assess the data quality and study integrity. On site they will review study records and directly compare them with source documents, discuss the conduct of the study with the investigator, and verify that the facilities remain acceptable.

In addition, the study may be evaluated by Sponsor or designee internal auditors and government inspectors who must be allowed access to CRFs, source documents, other study files, and study facilities. BMS audit reports will be kept confidential.

The investigator must notify BMS promptly of any inspections scheduled by regulatory authorities, and promptly forward copies of inspection reports to Sponsor or designee.

RECORDS RETENTION

The investigator (or head of the study site in Japan) must retain all study records and source documents for the maximum period required by applicable regulations and guidelines, or institution procedures, or for the period specified by BMS or designee, whichever is longer. The investigator (or head of the study site in Japan) must contact BMS prior to destroying any records associated with the study.

BMS or designee will notify the investigator (or head of the study site in Japan) when the study records are no longer needed.

If the investigator withdraws from the study (e.g., relocation, retirement), the records shall be transferred to a mutually agreed upon designee (e.g., another investigator, study site, IRB). Notice of such transfer will be given in writing to BMS or designee.

RETURN OF STUDY TREATMENT

For this study, study treatments (those supplied by BMS, a vendor or sourced by the investigator) such as partially used study treatment containers, vials and syringes may be destroyed on site.

If..	Then
Study treatments supplied by BMS (including its vendors	Any unused study treatments supplied by BMS can only be destroyed after being inspected and reconciled by the responsible Study Monitor unless study treatments containers must be immediately destroyed as required for safety, or to meet local regulations (e.g., cytotoxics or biologics).<br>If study treatments will be returned, the return will be arranged by the responsible Study Monitor.
Study treatments sourced by site, not supplied by BMS (or its vendors) (examples include study treatments sourced from the sites stock or commercial supply, or a specialty pharmacy)	It is the investigator's or designee's responsibility to dispose of all containers according to the institutional guidelines and procedures.

It is the investigator's or designee's responsibility to arrange for disposal, provided that procedures for proper disposal have been established according to applicable federal, state, local,

Approved v1.0 [redacted]

and institutional guidelines and procedures, and provided that appropriate records of disposal are kept. The following minimal standards must be met:

- On-site disposal practices must not expose humans to risks from the drug.
- On-site disposal practices and procedures are in agreement with applicable laws and regulations, including any special requirements for controlled or hazardous substances.
- Written procedures for on-site disposal are available and followed. The procedures must be filed with the site's SOPs and a copy provided to BMS upon request.
- Records are maintained that allow for traceability of each container, including the date disposed of, quantity disposed, and identification of the person disposing the containers. The method of disposal, i.e., incinerator, licensed sanitary landfill, or licensed waste disposal vendor must be documented.
- Accountability and disposal records are complete, up-to-date, and available for the Monitor to review throughout the clinical trial period.

It is the investigator's or designee's responsibility to arrange for disposal of all empty containers.

If conditions for destruction cannot be met the responsible Study Monitor will make arrangements for return of study treatments provided by BMS (or its vendors). Destruction of non- study treatments sourced by the site, not supplied by BMS, is solely the responsibility of the investigator or designee.

CLINICAL STUDY REPORT AND PUBLICATIONS

A Signatory Investigator must be selected to sign the clinical study report.

For this protocol, the Signatory Investigator will be selected as appropriate based on the following criteria:

- External Principal Investigator designated at protocol development
- National Coordinating Investigator
- Study Steering Committee chair or their designee
- Participant recruitment (e.g., among the top quartile of enrollers)
- Involvement in trial design
- Regional representation (e.g., among top quartile of enrollers from a specified region or country)
- Other criteria (as determined by the study team)

The data collected during this study are confidential and proprietary to Sponsor or designee. Any publications or abstracts arising from this study must adhere to the publication requirements set forth in the clinical trial agreement (CTA) governing [Study site or Investigator] participation in the study. These requirements include, but are not limited to, submitting proposed publications to Sponsor or designee at the earliest practicable time prior to submission or presentation and otherwise within the time period set forth in the CTA.

Approved v1.0 [redacted]

APPENDIX 8 ADVERSE EVENTS AND SERIOUS ADVERSE EVENTS: DEFINITIONS AND PROCEDURES FOR RECORDING, EVALUATING, FOLLOW UP AND REPORTING

ADVERSE EVENTS

**Adverse Event Definition:**
An Adverse Event (AE) is defined as any new untoward medical occurrence or worsening of a preexisting medical condition in a clinical investigation participant administered study drug and that does not necessarily have a causal relationship with this treatment.
An AE can therefore be any unfavorable and unintended sign (such as an abnormal laboratory finding), symptom, or disease temporally associated with the use of study drug, whether or not considered related to the study drug.
All nonserious adverse events (not only those deemed to be treatment-related) should be collected continuously during the treatment period and for a minimum of 100 days following discontinuation of nedoadjuvant therapy, 90 days after surgery, and 30 days after adjuvant therapy<br>Every adverse event must be assessed by the investigator with regard to whether it is considered immune-mediated. For events which are potentially immune-mediated, additional information will be collected on the participant's case report form.

SERIOUS ADVERSE EVENTS

**Serious Adverse Event (SAE) is defined as any untoward medical occurrence that, at any dose:**
Results in death
Is life-threatening (defined as an event in which the participant was at risk of death at the time of the event; it does not refer to an event which hypothetically might have caused death if it were more severe)
Requires inpatient hospitalization or causes prolongation of existing hospitalization (see NOTE below)<br>NOTE:<br>The following hospitalizations are not considered SAEs in BMS clinical studies:<br>• a visit to the emergency room or other hospital department < 24 hours, that does not result in admission (unless considered an important medical or life-threatening event)<br>• elective surgery, planned prior to signing consent<br>• admissions as per protocol for a planned medical/surgical procedure<br>• routine health assessment requiring admission for baseline/trending of health status (e.g., routine colonoscopy)<br>• medical/surgical admission other than to remedy ill health and planned prior to entry into the study. Appropriate documentation is required in these cases<br>• admission encountered for another life circumstance that carries no bearing on health status and requires no medical/surgical intervention (e.g., lack of housing, economic inadequacy, caregiver respite, family circumstances, administrative reason)<br>• admission for administration of anticancer therapy in the absence of any other SAEs (applies to oncology protocols)
Results in persistent or significant disability/incapacity
Is a congenital anomaly/birth defect

Serious Adverse Event (SAE) is defined as any untoward medical occurrence that, at any dose:
Is an important medical event (defined as a medical event(s) that may not be immediately life-threatening or result in death or hospitalization but, based upon appropriate medical and scientific judgment, may jeopardize the participant or may require intervention [e.g., medical, surgical] to prevent one of the other serious outcomes listed in the definition above.) Examples of such events include, but are not limited to, intensive treatment in an emergency room or at home for allergic bronchospasm; blood dyscrasias or convulsions that do not result in hospitalization.) Potential drug induced liver injury (DILI) is also considered an important medical event. (See Section 8.1.1 for the definition of potential DILI.)

Suspected transmission of an infectious agent (e.g., pathogenic or nonpathogenic) via the study treatment is an SAE.

Although pregnancy, overdose, cancer, and potential drug induced liver injury (DILI) are not always serious by regulatory definition, these events must be handled as SAEs. (See Section 9.2.5 for reporting pregnancies).

Any component of a study endpoint that is considered related to study therapy should be reported as SAE (eg, death is an endpoint, if death occurred due to anaphylaxis, anaphylaxis must be reported).

All SAEs must be collected that occur during the screening period and within 100 days of the last dose of neoadjuvant therapy, 90 days of surgery, and 100 days of adjuvant therapy. For participants randomized/assigned to treatment and never treated with study drug, SAEs should be collected for 30 days from the date of randomization.

EVALUATING AES AND SAES

Assessment of Causality
The causal relationship to study drug is determined by a physician and should be used to assess all adverse events (AE). The causal relationship can be one of the following:<br>Related: There is a reasonable causal relationship between study drug administration and the AE.<br>Not related: There is not a reasonable causal relationship between study drug administration and the AE.<br>The term "reasonable causal relationship" means there is evidence to suggest a causal relationship.

Follow-up of AEs and SAEs
If only limited information is initially available, follow-up reports are required. (Note: Follow-up SAE reports must include the same investigator term(s) initially reported.)<br>If an ongoing SAE changes in its intensity or relationship to study drug or if new information becomes available, the SAE report must be updated and submitted within 24 hours to BMS (or designee) using the same procedure used for transmitting the initial SAE report.<br>All SAEs must be followed to resolution or stabilization.

REPORTING OF SAES TO SPONSOR OR DESIGNEE

• SAEs, whether related or not related to study drug, and pregnancies must be reported to BMS (or designee) within 24 hours of awareness of the event.<br>• SAEs must be recorded on the SAE Report Form; pregnancies on a Pregnancy Surveillance Form

(electronic or paper forms).

- The preferred method for SAE data reporting collection is through the eCRF.
- The paper SAE/pregnancy surveillance forms are only intended as a back-up option when the eCRF system is not functioning.
- In this case, the paper forms are to be transmitted via email or confirmed facsimile (fax) transmission to:

SAE Email Address: Refer to Contact Information list.

SAE Facsimile Number: Refer to Contact Information list.

For studies capturing SAEs through electronic data capture (EDC), electronic submission is the required method for reporting. In the event the electronic system is unavailable for transmission, paper forms must be used and submitted immediately. When paper forms are used, the original paper forms are to remain on site.

SAE Telephone Contact (required for SAE and pregnancy reporting): Refer to Contact Information list

Protocol Number: CA209816
IND Number: 125,872
EUDRACT Number: 2016-003536-21
Date: 30-Sep-2016
Revised Date: 18-Aug-2021

CLINICAL PROTOCOL CA209816

Randomized, Open-Label, Phase 3 Trial of Nivolumab plus Ipilimumab or Nivolumab plus Platinum-Doublet Chemotherapy versus Platinum-Doublet Chemotherapy in Early Stage NSCLC
(CheckMate 816: CHECKpoint pathway and nivoluMAb clinical Trial Evaluation 816)

Protocol Amendment 07

[redacted]

24-hr Emergency Telephone Number

[redacted]

Bristol-Myers Squibb Company
3401 Princeton Pike
Lawrenceville, NJ 08648

This document is the confidential and proprietary information of Bristol-Myers Squibb Company and its global affiliates (BMS). By reviewing this document, you agree to keep it confidential and to use and disclose it solely for the purpose of assessing whether your organization will participate in and/or the performance of the proposed BMS sponsored study. Any permitted disclosures will be made only on a confidential "need to know" basis within your organization or to your independent ethics committee(s). Any other use, copying, disclosure or dissemination of this information is strictly prohibited unless expressly authorized in writing by BMS. Any supplemental information (e.g., amendments) that may be added to this document is also confidential and proprietary to BMS and must be kept in confidence in the same manner as the contents of this document. Any person who receives this document without due authorization from BMS is requested to return it to BMS or promptly destroy it. All other rights reserved. References to BMS in this protocol may apply to partners to which BMS has transferred obligations, e.g., a Contract Research Organization (CRO).

DOCUMENT HISTORY

Document	Date of Issue	Summary of Changes
Protocol Amendment 07	18-Aug-2021	• To account for a potential slowdown in EFS events accrual in long-term follow-up, the protocol was amended to:<br>– Revise EFS modeling assumptions to a piecewise exponential distribution with lower events rate in the longer term.<br>– Include one additional EFS interim analysis at 90% information fraction [redacted]<br>[redacted]
Revised Protocol 06	14-Jul-2020	• Clarified EFS definition<br>• Removed the first of 2 interim analyses of EFS and updated alpha spending on the remaining interim and final analyses of EFS<br>• Clarified that the actual timing of analyses may differ from projected timing<br>• Removed text about descriptive EFS analysis<br>[redacted]
Revised Protocol 05	18-Sep-2019	• Modified pCR analysis population and projected timelines<br>• Updated surgical approach endpoint<br>• Updated the censoring rule of TTDM<br>• No optional biopsy at disease progression collected in China.<br>• Updated Management Algorithms to include myocarditis<br>[redacted]
Revised Protocol 04	25-Jun-2019	• Added the concomitant administration of substances that are also tubularly secreted (eg, probenecid) could potentially result in delayed clearance of pemetrexed.<br>• Added hypothesis testing for overall survival<br>• Clarified the pCR analysis population<br>• Added exploratory endpoint of Event Free Survival on next line of therapy<br>• Added instructions for BICR<br>• Updated Appendix 8 Adverse Events and Serious Adverse Events: Definitions and Procedures for Recording, Evaluating, Follow Up and Reporting Adverse Events
Revised Protocol 03	21-Sep-2018	• Enrollment in Arm A (nivolumab plus ipilimumab) was stopped<br>• Randomization of participants (1:1 ratio) into Arm C and B will continue to for a total of 350 participants<br>• Definition of event free survival is clarified.<br>• Participants with large-cell neuroendocrine carcinoma tumor histology are excluded<br>• Additional platinum doublet chemotherapy regimen (paclitaxel/carboplatin) was added

Approved v9.0 [redacted]

Document	Date of Issue	Summary of Changes
		• Dose modification for docetaxel was updated<br>• Time to death or distant metastases (TTDM) was added to secondary endpoints.<br>• Tumor assessments for participants who do not proceed to definitive surgery was clarified<br>• Statistical analysis plan was updated<br>• Rationale, background information, and trial schematic were updated<br>• Pulmonary function parameters were clarified<br>• Time relationship between adjuvant radiotherapy and tumor imaging assessments was clarified<br>• Time window of Cycle 1 Day 1 end-of-infusion PK sampling for Arm A and C was clarified
Administrative Letter 03	31-Jan-2018	• 1. Page 19, Table 2-1 Screening Procedural Outline (CA209816), Pulmonary Function Test notes<br>• Previously written:<br>• Should be performed within 6 weeks prior to randomization.<br>• Changed to:<br>• Should be performed within 6 weeks prior to randomization. PFTs should be evaluated at screening with the following parameters: FVC, FEV1, TLC, FRC, and DLco.<br>•<br>• 2. Page 22, Table 2-2 Neoadjuvant Period Procedural Outline (CA209816), Pulmonary Function Test notes<br>• Previously written:<br>• PFTs should be re-evaluated prior to surgery only.<br>• Changed to:<br>• PFTs should be re-evaluated prior to surgery with the following parameters: FVC, FEV1, TLC, FRC, and DLco.<br>• 3. Page 28, Section Table 2-4 Post-Neoadjuvant Period for Participants Receiving Adjuvant Chemotherapy (CA209816), Tumor Assessments<br>• Previously written:<br>• Tumor assessments using CT, with contrast, of the chest including adrenal glands and CT or MRI of other additional suspected/known sites of disease. The first tumor assessment should occur 12 weeks (± 7 days) after definitive surgery per RECIST 1.1 and then should occur every 12 weeks (± 7 days) per RECIST 1.1 for 2 years (104 weeks), then every 6 months (24 weeks ± 7 days) for 3 years, and every year (52 weeks ± 7 days) for 5 years or until disease recurrence or progression is confirmed by BICR. Use same imaging methods and machine of the same quality as was used at screening/baseline.<br>• Changed to:<br>• Tumor assessments using CT, with contrast, of the chest including adrenal glands and CT or MRI of other additional suspected/known sites of disease. The first tumor assessment should occur 12 weeks (± 7 days) after definitive surgery per RECIST 1.1 and then should occur every 12

Approved v9.0 [redacted]

Document	Date of Issue	Summary of Changes
		weeks (± 7 days) per RECIST 1.1 for 2 years (104 weeks), then every 6 months (24 weeks ± 7 days) for 3 years, and every year (52 weeks ± 7 days) for 5 years or until disease recurrence or progression is confirmed by BICR. Use same imaging methods and machine of the same quality as was used at screening/baseline.<br>• Adjuvant radiotherapy is allowed on study. As radiotherapy may interfere with imaging interpretation, efforts should be made to schedule radiotherapy so tumor assessments as scheduled per protocol do not cross over into the radiation period.<br>• 4. Page 92, Table 9.5-1 Pharmacokinetic (PK) Sample Collections for Nivolumab and Ipilimumab - Arm A and Arm C Footnote d<br>• Previously written:<br>• No text<br>• Added footnote:<br>• dAs per revised protocol 02, the Cycle 1 Day 1 end-of-infusion PK sampling of nivolumab/ipilimumab in Arm A and of nivolumab in Arm C should be taken immediately, ie within 5 minutes prior to stopping the infusion of nivolumab and ipilimumab, respectively. PK sampling greater than 5 minutes before the end of infusion will be reported as a deviation.
Revised Protocol 02	06-Jul-2017	• Nivolumab plus platinum-doublet chemotherapy arm (Arm C) was added<br>• The sample size was increased to 642 participants with the addition of the new treatment arm.<br>• The primary objective was changed to multiple primary objectives of event free survival and pathological complete response; the secondary objective was changed to major pathology response.<br>• Additional rationale and background information was provided.<br>• Pre-screening tissue requirement was increased from minimum of 10 slides to 15 slides.<br>• Contrast requirements for brain MRI scans were updated<br>• Time window for pulmonary function test window was expanded from with 28 days of randomization to within 6 weeks of randomization<br>• Synopsis was updated with exploratory objectives, endpoints, and schema.<br>• Language in treatments administered was deleted and reference to Investigator Brochure and Pharmacy Manual was included.<br>[redacted]
Revised Protocol 01	03-Mar-2017	Incorporates Amendment 02 and Administrative Letters 01 and 02
Amendment 02	03-Mar-2017	• To adjust the dosing details of the chemotherapy regimens to include the dose approved by the local prescribing information and the standard of care infusion time for each country included in this study.<br>• To expanded and to split the broad biomarker objective into 3 more detailed objectives<br>• Clarify lymph node samples at screening and at definitive surgery

Approved v 9.0 [redacted]

Document	Date of Issue	Summary of Changes
		• Clarify requirements for PET/CT scans and broadening the window of scans prior to surgery<br>• Clarify tissue sample process for calculation of the primary endpoint<br>• Adjust Hepatitis B Virus criteria<br>• Added live vaccines and strong CYP3A4 inhibitors to the Prohibited Treatments<br>• Added caution for concomitant administration of NSAIDs with pemetrexed.<br>• Added unacceptable methods of contraception to Appendix 6
Administrative Letter 02	30-Nov-2017	• Clarify the correct version of the TNM Staging System.<br>• Clarify that a minimum of 228 PD-L1+ participants will be randomized.<br>• Clarify that physical exams, vital signs, and physical measurements should be collected prior to each dose of neoadjuvant and adjuvant therapy.<br>• Clarify that the first post-operative tumor assessment should be performed 12 weeks (± 7 days) after definitive surgery.<br>• Remove the phrase "non-protocol regimen" in regards to a non-cisplatin regimen as the protocol includes a non-cisplatin regimen option.<br>• Clarify that weight-based dosing should be rounded up to the nearest milligram or per institutional standards.<br>• Clarify that the EQ-5D-3L should be collected prior to Day 1 only in cycles that have multiple dosing days in each cycle.
Administrative Letter 01	31-Oct-2017	• To correct the IND number
Original Protocol	30-Sep-2016	Not Applicable

Approved v9.0 [redacted]

OVERALL RATIONALE FOR PROTOCOL AMENDMENT 07:

Based on historical and emerging external data, event accrual for event-free survival (EFS) analysis is expected to slow down in long-term follow up after definitive surgery for resectable non-small-cell lung cancer (NSCLC). Recurrence dynamics after complete resection for stage I/II NSCLC show a constant recurrence rate of 6-7 events per 100 person-years of follow-up during the first 4 years, decreasing to 2 events per 100 person-year thereafter.[1] A similar slowdown was seen with stage III NSCLC. In a randomized, Phase III trial comparing neoadjuvant chemoradiation and chemotherapy both followed by surgery in stage III NSCLC, no difference was seen in progression-free survival (PFS, primary endpoint), with the PFS curves dropping sharply during the first year indicating initial high hazard rates, then flattening thereafter suggesting a markedly decreasing and stabilizing hazard rate.[2] Such pattern of recurrence dynamics was repeatedly seen in other neoadjuvant trials conducted in Stage I-III NSCLC.[3,4]

Furthermore, preliminary data from recently published Phase II trials incorporating nivolumab into neoadjuvant regimens suggest potentially further decreased hazard rates for recurrence after surgery by the flattened shape of Kaplan-Meier curves of EFS/PFS. For example, in the single-arm NADIM trial of neoadjuvant nivolumab + chemotherapy followed by surgery and adjuvant nivolumab for Stage IIIA NSCLC, the 24-month PFS rate was 77.1% (95% CI 59.9-87.7), greatly exceeding historic standards.[5,6]

To account for the aforementioned event dynamics which may preclude the necessary EFS events from accruing in a reasonable timeframe or potentially never accruing, the statistical analyses plan has been updated to model the assumptions based on a piecewise exponential distribution and incorporate a calendar-based rule in addition to the existing event-driven approach to trigger analysis timing. Moreover, one additional EFS interim analysis (IA) has been added [redacted] at 90% of EFS events information fraction [redacted]. No changes have been made to the target number of events for the existing interim and final analyses, and the overall type 1 error has been maintained. At the time of this amendment, EFS and overall survival (OS) remain blinded and have not been analyzed by the Sponsor or investigators.

Approved v[illegible].0 [redacted]

SUMMARY OF KEY CHANGES OF PROTOCOL AMENDMENT 07		
**Section Number and Name**	**Description of Change**	**Brief Rationale**
Title Page	Updated title to "Clinical Trial Physician" and added Clinical Scientist contact information.	Updated to align with study contacts and the role name for Medical Monitor at BMS.
Section 5.3, End of Study Definition	Included additional interim analysis and calendar-based rule or the EFS final analysis.	To account for a potential slowdown in EFS event accrual in long-term follow-up in this setting.
Section 9.2.1, Time Period and Frequency for Collecting AE and SAE Information	Removed obsolete cross-referenced sections to the Investigator Brochure (IB).	To align with the updated IB.
Section 10.1.2, Event Free Survival (EFS)	• Updated power calculation.<br>• Revised EFS assumptions for the control arm with piecewise exponential model.<br>• Added an additional interim analysis at 90% information fraction [redacted]<br>[redacted]<br>• Updated the timing of analyses based on a piecewise exponential model.	To account for a potential slowdown in EFS event accrual in long-term follow-up in this setting.
[redacted]		
Section 10.1.4, Analyses Timing Projections<br><br>Section 10.3.3, Interim Analyses	• Revised timing projections based on updated EFS assumptions modeling and additional interim analysis included.<br>• Removed Table 10.1.4-1, Scheduled Analyses, Criteria, and Projected Timelines.	To align with Sections 10.1.2 and 10.1.3 updates.
Appendix 2, Abbreviations and Trademarks	Updated the following abbreviations:<br>• final analysis<br>• interim analysis	Abbreviations were updated for clarity and completeness.

References

1 Luo F, Huang J, Sima CS et al. Patterns of recurrence and second primary lung cancer in early-stage lung cancer survivors followed with routine computed tomography surveillance. J Thorac Cardiovasc Surg. 2013; 145:75-81)

2 Thomas M, Rube C, Hoffknecht P et al. Effect of preoperative chemoradiation in addition to preoperative chemotherapy: a randomised trial in stage III non-small-cell lung cancer. Lancet Oncol 2008; 9:636-48.)

3 Pless M, Stupp R, Ris HB, et al. Induction chemoradiation in stage IIIA/N2 non-small-cell lung cancer: a phase 3 randomised trial. Lancet 2015; 386:1049-56.

4 Felip E, Rosell R, Maestre JA, et al. Preoperative chemotherapy plus surgery versus surgery plus adjuvant chemotherapy versus surgery alone in early-stage non-small-cell lung cancer. Clin Oncol 2010 Jul 1; 28(19): 3138-45

5 Provencio M, Insa A, Casal-Rubio J et al. Neoadjuvant chemotherapy and nivolumab in resectable non-small-cell lung cancer (NADIM): an open-label, multicentre, single-arm, phase 2 trial. Lancet Oncol 2020; 21:1413-22.

6 Cascone T, William WN, Weissferdt A, et al. Neoadjuvant nivolumab or nivolumab plus ipilimumab in operable non-small cell lung cancer: the phase II randomized NEOSTAR trial. Nat Med 2021.27;504-14.)

Approved v 1.0 [redacted]

TABLE OF CONTENTS

TITLE PAGE 1
DOCUMENT HISTORY 2
OVERALL RATIONALE FOR PROTOCOL AMENDMENT 07: 6
SUMMARY OF KEY CHANGES OF PROTOCOL AMENDMENT 07 7
TABLE OF CONTENTS 9
1 SYNOPSIS 13
2 SCHEDULE OF ACTIVITIES 23
3 INTRODUCTION 34
3.1 Study Rationale 34
3.1.1 Research Hypothesis 36
3.2 Background 36
3.2.1 Indication Background 36
3.2.2 Nivolumab Mechanism of Action 39
3.2.3 Ipilimumab Mechanism of Action 40
3.2.4 Nivolumab Combined with Ipilimumab Clinical Activity 40
3.2.5 Immuno-oncology Treatment Combined with Chemotherapy Clinical Activity 41
3.3 Benefit/Risk Assessment 43
4 OBJECTIVES AND ENDPOINTS 45
5 STUDY DESIGN 47
5.1 Overall Design 47
5.1.1 Data Monitoring Committee and Other External Committees 55
5.2 Number of Participants 56
5.3 End of Study Definition 56
5.4 Scientific Rationale for Study Design 56
5.4.1 Rationale for Open-Label Design 56
5.4.2 Rationale for Preoperative Systemic Therapy in NSCLC 57
5.4.3 Rationale for Immuno-oncology Treatment in Neoadjuvant NSCLC 57
5.4.3.1 Rationale for Combination of Nivolumab and Ipilimumab (Arm A): 58
5.4.3.2 Rationale for PD-1 Inhibitor with Chemotherapy (Arm C) 61
5.4.4 Rationale for Pathologic Response 63
5.4.5 Rationale for Patient Population 64
5.4.6 Rationale for Choices of Platinum-based Chemotherapy Doublet 64
5.5 Justification for Dose 65
5.5.1 Rationale for Shorter Infusion Times for Nivolumab 65
5.5.2 Rationale for Nivolumab 360 mg Flat Dose 65
6 STUDY POPULATION 66
6.1 Inclusion Criteria 66
6.2 Exclusion Criteria 68
6.3 Lifestyle Restrictions 69
6.4 Screen Failures 69
6.4.1 Retesting During Screening or Lead-In Period 70
7 TREATMENT 70

Approved v 9.0 [redacted]

7.1 Treatments Administered 73
7.2 Method of Treatment Assignment 80
7.3 Blinding 81
7.4 Dosage Modification 81
7.4.1 Dose Reductions for Platinum Doublet Chemotherapy 81
7.4.1.1 Platinum Doublet Chemotherapy - Dose Reductions for Hematologic Toxicity 83
7.4.1.2 Platinum Doublet Chemotherapy - Dose Reductions for Non-Hematologic Toxicities 85
7.4.2 Dose Delay 87
7.4.2.1 Nivolumab Dose Delay Criteria 87
7.4.2.2 Dose Delay Criteria for Platinum Doublet Chemotherapy 87
7.4.2.3 Docetaxel Dose Delay Criteria 88
7.4.3 Treatment of Nivolumab- and Ipilimumab-related Infusion Reactions 89
7.5 Preparation/Handling/Storage/Accountability 90
7.5.1 Retained Samples for Bioavailability / Bioequivalence 90
7.6 Treatment Compliance 91
7.7 Concomitant Therapy 91
7.7.1 Prohibited and/or Restricted Treatments 91
7.7.2 Other Restrictions and Precautions 91
7.7.3 Permitted Therapy 92
7.8 Treatment After the End of the Study 92
8 DISCONTINUATION CRITERIA 92
8.1 Discontinuation from Study Treatment 92
8.1.1 Study Treatment Discontinuation Criteria 93
8.1.1.1 Nivolumab Discontinuation Criteria 93
8.1.1.2 Platinum Doublet Chemotherapy Dose Discontinuation 94
8.1.1.3 Docetaxel Dose Discontinuation 96
8.1.2 Criteria to Resume Treatment 96
8.1.2.1 Criteria to Resume Nivolumab Treatment 96
8.1.2.2 Criteria to Resume Platinum Doublet Chemotherapy Dosing 97
8.1.2.3 Criteria to Resume Treatment with Docetaxel 97
8.1.3 Post Study Follow-up 97
8.2 Discontinuation from the Study 98
8.3 Lost to Follow-Up 98
9 STUDY ASSESSMENTS AND PROCEDURES 99
9.1 Efficacy Assessments 99
9.1.1 Imaging Assessment for the Study 100
9.1.2 Patient-reported Outcomes 101
9.2 Adverse Events 102
9.2.1 Time Period and Frequency for Collecting AE and SAE Information 102
9.2.2 Method of Detecting AEs and SAEs 103
9.2.3 Follow-up of AEs and SAEs 103
9.2.4 Regulatory Reporting Requirements for SAEs 103
9.2.5 Pregnancy 104
9.2.6 Laboratory Test Result Abnormalities 104

Approved v9.0 [redacted]

9.2.7 Potential Drug Induced Liver Injury (DILI) 105
9.2.8 Other Safety Considerations 105
9.2.9 Management Algorithms 106
9.3 Overdose 107
9.4 Safety 107
9.4.1 Clinical Safety Laboratory Assessments 108
9.4.2 Imaging Safety Assessment 108
9.5 Pharmacokinetics 108
9.6 Pharmacodynamics 109
9.7 Pharmacogenomics 109
9.8 Biomarkers 109
9.8.1 Additional Research Collection 109
9.8.1.1 Tumor Tissue Specimens 111
[redacted]
9.8.2 Safety Analyses 113
9.9 Health Economics OR Medical Resource Utilization and Health Economics . 114
9.10 Other Assessments 114
9.10.1 Diagnostic/Pre-surgical Evaluation and Surgical Guidelines: 114
9.10.2 Post-surgical Evaluation: 115
10 STATISTICAL CONSIDERATIONS 115
10.1 Sample Size Determination 115
10.1.1 Pathologic Complete Response (pCR) 116
10.1.2 Event Free Survival (EFS) 116
10.1.3 Power Consideration for Overall Survival (OS) 117
10.1.4 Analyses Timing Projections 119
10.2 Populations for Analyses 119
10.3 Statistical Analyses 120
10.3.1 Efficacy Analyses 120
10.3.2 Other Analyses 121
10.3.3 Interim Analyses 121
11 REFERENCES 123
12 APPENDICES 128
APPENDIX 1 TNM STAGING SYSTEM FOR LUNG CANCER (7TH EDITION) 129
APPENDIX 2 ABBREVIATIONS AND TRADEMARKS 131
APPENDIX 3 ECOG PERFORMANCE STATUS 135
APPENDIX 4 NIVOLUMAB MANAGEMENT ALGORITHMS 136
APPENDIX 5 RESPONSE CRITERIA (RECIST 1.1) 145
APPENDIX 6 WOMEN OF CHILDBEARING POTENTIAL DEFINITIONS AND METHODS OF CONTRACEPTION 152
APPENDIX 7 STUDY GOVERNANCE CONSIDERATIONS 155

Approved v 9.0 [redacted]

APPENDIX 8 ADVERSE EVENTS AND SERIOUS ADVERSE EVENTS: DEFINITIONS AND PROCEDURES FOR RECORDING, EVALUATING, FOLLOW UP AND REPORTING ADVERSE EVENTS 162
APPENDIX 9 REVISED PROTOCOL SUMMARY OF CHANGE HISTORY 166

1 SYNOPSIS

Protocol Title: Randomized, Open-Label, Phase 3 Trial of Nivolumab plus Ipilimumab or Nivolumab plus Platinum-Doublet Chemotherapy versus Platinum-Doublet Chemotherapy in Early Stage NSCLC

Study Phase: 3

Rationale:

Approximately 80% of lung cancer cases are non-small cell lung cancer (NSCLC), with most patients presenting with late-stage disease. At initial diagnosis, 20% of patients present with stage I or II disease, whereas 30% present with stage III disease and 50% with stage IV disease. With enhanced lung cancer screening techniques, the percentage of patients diagnosed during the early stages may increase over the duration of the trial. A standard TNM staging system is used to determine the staging for NSCLC (Appendix 1). Patients with pathologic stage I NSCLC have a 5-year survival of approximately 60%. Stage II to III NSCLC patients have a 5-year survival of approximately 25% to 40%.[1] Surgical resection remains the mainstay of treatment for stage I and II patients; however, despite potentially curative surgery, approximately 50% of stage IB and 60-75% of stage II NSCLC patients will relapse and eventually die of their disease.[2,3] A rational approach to improve survival in these patients is to eradicate micrometastatic disease and to minimize the risk of relapse after adjuvant or neoadjuvant chemotherapy.

This phase 3 study, CA209816, will evaluate the clinical efficacy of nivolumab plus ipilimumab or nivolumab plus platinum doublet chemotherapy in operable lung cancer. Specifically, this study will compare event free survival (EFS) and compare the pathologic complete response (pCR) rate among participants with Stage IB-IIIa NSCLC treated with nivolumab plus platinum doublet chemotherapy to the EFS and pCR rates in participants treated with platinum-doublet chemotherapy.

Study Population:

1) Key Inclusion Criteria
   a) Eastern Cooperative Group (ECOG) Performance Status 0-1
   b) Participants with histologically confirmed Stage IB (≥ 4 cm), II, IIIA (N2) NSCLC (per the 7th International Association for the Study of Lung Cancer) who are considered resectable
   c) Measurable disease according to Response Evaluation Criteria in Solid Tumors version 1.1 (RECIST 1.1)
   d) Participants must have tumor tissue sample available for PD-L1 immunohistochemical (IHC) testing performed by a third-party analyzing lab during the screening period:
      i) Either a formalin-fixed, paraffin-embedded (FFPE) tissue block or unstained tumor tissue sections, with an associated pathology report, must be submitted for biomarker

evaluation prior to randomization. The tumor tissue sample may be fresh or archival if obtained within 3 months prior to enrollment.

ii) Tissue must be a core needle biopsy, excisional or incisional biopsy. Fine needle biopsies obtained by EBUS is not considered adequate for biomarker review and randomization. Core needle biopsies obtained by EBUS are acceptable for randomization.

e) All suspicious mediastinal lymph nodes including those that are pathologically enlarged or FDG avid on PET/CT require further sampling for pathological confirmation if accessible by EBUS, thoracoscopy, or mediastinoscopy.

2) Key Exclusion Criteria:
   a) Presence of locally advanced, unresectable or metastatic disease.
   b) Participants with known EGFR mutations or ALK translocation. If testing is done, testing will be done using an FDA-approved assay and will be performed locally.

Objectives and Endpoints:

Objective	Endpoint
**Primary**	
• To compare the event-free survival (EFS) by blinded independent central review (BICR) in participants receiving nivolumab plus platinum doublet chemotherapy vs participants receiving platinum doublet chemotherapy in operable stage IB (≥ 4 cm), II, or resectable IIIA (N2) NSCLC.	• EFS defined as the length of time from randomization to any of the following events: any progression of disease precluding surgery, progression or recurrence disease (based on BICR assessment per RECIST 1.1) after surgery, or death due to any cause. Participants who don’t undergo surgery for reason other than progression will be considered to have an event at RECIST 1.1 progression (based on BICR) or death.
• To compare the pathologic complete response (pCR) rate in participants receiving nivolumab plus platinum doublet chemotherapy vs participants receiving platinum doublet chemotherapy in operable stage IB (≥ 4 cm), II, or resectable IIIA (N2) NSCLC	• pCR rate is defined as number of randomized participants with absence of residual tumor in lung and lymph nodes as evaluated by blinded independent pathology review (BIPR), divided by the number of randomized participants for each treatment group.
**Secondary**	
• To assess the major pathologic response (MPR) rate by BIPR of participants receiving nivolumab plus platinum doublet chemotherapy vs participants receiving platinum doublet chemotherapy in operable stage IB (≥ 4 cm), II, or resectable IIIA (N2) NSCLC	• MPR rate, defined as number of randomized participants with ≤ 10% residual tumor in lung and lymph nodes as evaluated by BIPR, divided by the number of randomized participants for each treatment group. Viable tumors in situ carcinoma should not be included in MPR calculation.

Approved v9.0 [redacted]

Objective	Endpoint
• To compare the OS of participants receiving nivolumab plus platinum doublet chemotherapy vs participants receiving platinum doublet chemotherapy in operable stage IB (≥ 4 cm), II, or resectable IIIA (N2) NSCLC	• OS is defined as the time between the date of randomization and the date of death. OS will be censored on the last date a participant was known to be alive.
• To assess the time to death or distant metastases (TTDM) of participants receiving nivolumab plus platinum doublet chemotherapy vs participants receiving platinum doublet chemotherapy in operable stage IB (≥ 4 cm), II, or resectable IIIA (N2) NSCLC	• TTDM is defined as the time between the date of randomization and the first date of distant metastasis or the date of death in the absence of distant metastasis. Distant metastasis is defined as any new lesion that is outside of the thorax using BICR according to RECIST 1.1. Patients who have not developed distant metastasis or died at the time of analysis will be censored on the date of their last evaluable tumor assessment.
• **Exploratory**	
• To assess clinical response rate (cRR) by BICR of participants receiving nivolumab plus platinum doublet chemotherapy vs participants receiving platinum doublet chemotherapy in operable stage IB (≥ 4 cm), II, or resectable IIIA (N2) NSCLC	• cRR is defined as proportion of all randomized participants whose overall radiological response prior to definitive surgery is either a complete response or partial response per RECIST 1.1 criteria by BICR
• To assess the pCR rate, MPR rate , cRR, EFS, TTDM, and OS in early-stage NSCLC participants treated with nivolumab plus platinum doublet chemotherapy compared to those treated with platinum doublet chemotherapy by PDL1 status (PD-L1 ≥ 1% , PD-L1< 1%/not evaluable/indeterminate)	• pCR rate, MPR rate, cRR, EFS, TTDM, and OS as described above
• To assess the feasibility of surgery and rate of peri- and post-operative complications (within 90 days of surgery) in participants receiving nivolumab plus platinum doublet chemotherapy compared to participants receiving platinum doublet	• Proportion of delayed or canceled surgery, duration of surgery, length of hospital stay, surgical approach, including completeness of the resection, incidence of AE/SAE associated with surgery, including pneumonitis, ARDS, re-admission to the Intensive Care Unit, atrial fibrillation or other supraventricular tachycardia (SVT) to 90 days post-surgery
• To assess the safety and tolerability of nivolumab plus platinum doublet chemotherapy compared to platinum doublet chemotherapy in early stage NSCLC	• Safety and tolerability will be measured by incidence of AE, SAE, immune-related AEs, deaths, and laboratory abnormalities
• To describe the pCR rate, MPR rate , cRR, EFS, TTDM, OS, feasibility of surgery, rate of peri- and post-operative complications (within 90 days of surgery), safety and tolerability in early-stage NSCLC participants treated with nivolumab plus ipilimumab and by PDL1 status (PD-L1 ≥ 1% , PD-L1< 1%/not evaluable/indeterminate)	• pCR rate, MPR rate, cRR, EFS, OS, TTDM, feasibility of surgery, rate of peri- and post-operative complications, safety and tolerability

Approved v 9.0 [redacted]

Objective	Endpoint
• To assess pharmacokinetics of the nivolumab plus ipilimumab or nivolumab plus platinum doublet chemotherapy in participants with early stage NSCLC	• Pharmacokinetic endpoints - See Table 9.5-1
• To assess the participant's overall health status and health utility using the 3-level version of the EQ-5D-3L visual analog scale (VAS) and utility index, respectively	• Change in EQ-5D-3L scores
• To assess Event Free survival on next line of therapy (EFS2) in early-stage NSCLC participants treated with nivolumab plus platinum doublet chemotherapy compared to those treated with platinum doublet	• Event Free survival on next line of therapy (EFS2)
• To evaluate tumor mutation burden as a potential predictive biomarker of efficacy (such as EFS and OS) of nivolumab plus platinum doublet chemotherapy and of platinum-doublet chemotherapy, using data generated from tumor and blood (germ-line control) specimens.	• Biomarker endpoints - See Section 9.8.
[redacted]	
• To explore potential predictive biomarkers of nivolumab plus platinum doublet chemotherapy efficacy (such as EFS and OS) in peripheral blood and tumor specimens [redacted]	
[redacted]	

Approved v[illegible].0 [redacted]

Overall Design:

This is an open-label, randomized clinical trial of up to 3 cycles of neoadjuvant nivolumab (3 mg/kg every 2 weeks) and a single dose of 1 mg/kg dose of ipilimumab, up to 3 cycles of nivolumab (360 mg) plus platinum doublet chemotherapy every 3 weeks, or neoadjuvant platinum doublet chemotherapy (up to 3 cycles) in participants with early stage (Stage IB [≥ 4 cm], II, and resectable IIIA [N2]) NSCLC.

Revised protocol 03 withholds randomization into Arm A of neoadjuvant nivolumab plus ipilimumab but continues randomizing eligible participants into either neoadjuvant nivolumab plus platinum doublet chemotherapy arm or platinum doublet chemotherapy arm. Participants already randomized in the original 2-arm part (neoadjuvant nivolumab plus ipilimumab vs neoajduvant chemotherapy) and in the arm of neoadjuvant nivolumab plus ipilimumab in 3-arm part defined by revised protocol 02 will remain in trial and continue scheduled trial procedures.

Study Schema

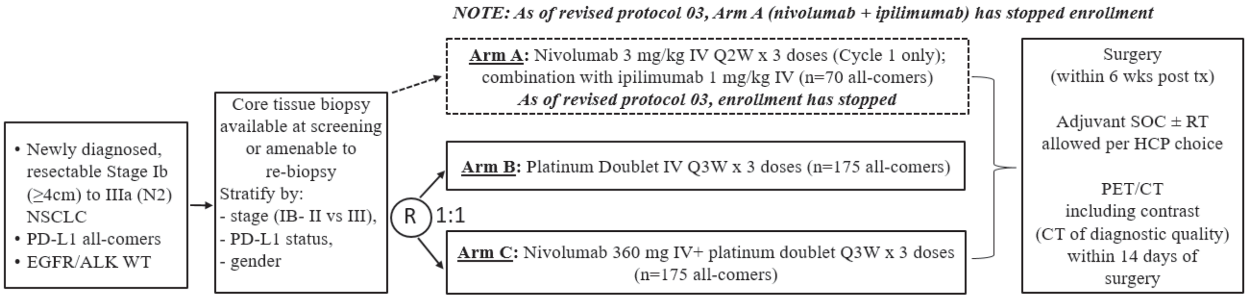

Endpoints
Primary: EFS and pCR rate in PD-L1 all-comers
Secondary: MPR, OS, and TTDM in PD-L1 all-comers
Exploratory: cRR in PD-L1 all-comers; pCR rate, EFS, MPR rate, OS, TTDM, and cRR by PD-L1 status. Safety, surgical feasibility, and rate of peri- and post-operative complications; PK, biomarkers, PROs
Post Surgical Assessments: CT /MRI Q12W for 2 yrs; then Q6 mos for 3 years, and every 52 weeks for 5 years thereafter until disease recurrence or PD. Independent review for pathological and radiologic response

Eligible participants will be randomized between 2 arms in a 1:1 ratio. Participants will be stratified by:

- PD-L1 expression (≥1% or <1%/not evaluable/indeterminate)
- Disease stage (IB/II vs IIIA)
- Gender

PD-L1 status will be determined by immunohistochemical (IHC) staining of PD-L1 protein in the submitted tumor sample and categorized as follows:

- PD-L1 positive - defined as ≥ 1% tumor cell membrane staining positive in a minimum of 100 evaluable tumor cells
- PD-L1 negative – defined as < 1% tumor cell membrane staining positive in a minimum of 100 evaluable tumor cells
- PD-L1 not evaluable/indeterminate - defined as participants with insufficient sample quantity or quality to stain for PD-L1 status or those in whom PD-L1 status could not be determined

Approved v9.0 [redacted]

despite appropriate amounts of tissue sample. For the purpose of stratification, this category will be grouped with PD-L1 negative participants. No more than 10% of participants will be randomized into this category.

Tumor tissue (archival [blocks/slides ≤ 3 months old] or recent tumor biopsy) must be submitted to a third-party vendor for determination of PD-L1 status testing prior to randomization.

PET/CT including contrast from the base of the skull to upper thighs will be performed at baseline (within 28 days prior to randomization) and within 14 days prior to planned definitive surgery. A separate CT, with contrast, of the chest, abdomen, and all other suspected sites of disease (as well as the PET/CT) is required if the CT component of a PET/CT is not of sufficient diagnostic quality for RECIST 1.1 measurements. Subsequent assessments (CT, including contrast, of the chest including adrenal glands and CT or MRI of other additional suspected/known sites of disease.) will be performed in accordance with the Schedule of Activities. Tumor assessments must continue per protocol until disease recurrence/progression is confirmed by BICR per RECIST 1.1. Details will be outlined in the radiology manual. OS will be followed continuously every 3 months via in-person or phone contact after Post-neoadjuvant Follow-up Visit 2 or after completion of adjuvant therapy, when applicable.

Following the completion of neoadjuvant treatment, all participants who remain operative candidates will undergo definitive surgery for NSCLC. Surgery should be performed within 6 weeks after completing neoadjuvant treatment for each treatment arm.

Following definitive surgery, participants in each arm may receive adjuvant chemotherapy with or without radiation per institutional standard at the discretion of the investigator.

Number of Participants:

Approximately 350 participants will be randomized in a 1:1 ratio (175 participants in each arm) to the 2 arms (neoadjuvant nivolumab plus platinum doublet chemotherapy and platinum doublet chemotherapy) from 1:1:1 randomization in revised protocol 02 and 1:1 randomization in revised protocol 03.

In addition, it is expected to have around 70 participants randomized in the original 2-arm study (Arm A and Arm B) and approximately another 75 participants randomized in the arm of neoadjuvant nivolumab plus ipilimumab (Arm A) when the study had 3 arms.

Treatment Arms and Duration:

Arm A: Participants randomized into Arm A received nivolumab 3 mg/kg IV over 30 minutes every 2 weeks for up to 3 doses (ie, 6 weeks of treatment; each cycle is 14 days). In Cycle 1 Day 1 only, nivolumab will be followed by a single dose of ipilimumab 1 mg/kg IV over 30 minutes.

Arm B: Participants randomized to Arm B will receive investigator-choice of platinum doublet chemotherapy regimens in 3-week cycles up to a maximum of 3 cycles of IV chemotherapy (ie, 9 weeks of treatment; each cycle is 21 days).

Approved v 9.0 [redacted]

- Regimen 1:
  - Vinorelbine 25 mg/m^2 or 30 mg/m^2 IV (per local prescribing information) push over 10 minutes or per institutional standard, Days 1 and 8
  - Cisplatin 75 mg/m^2 IV over 120 minutes or per institutional standard Day 1, immediately following vinorelbine
- Regimen 2:
  - Docetaxel 60 mg/m^2 or 75 mg/m^2 IV (per local prescribing information) over 60 minutes or per institutional standard on Day 1
  - Cisplatin 75 mg/m^2 IV over 120 minutes or per institutional standard on Day 1, immediately following docetaxel
- Regimen 3: (squamous histology):
  - Gemcitabine 1000 mg/m^2 or 1250 mg/m^2 IV (per local prescribing information) over 30 minutes or per institutional standard on Days 1 and 8
  - Cisplatin 75 mg/m^2 IV over 120 minutes or per institutional standard on Day 1, immediately following gemcitabine
- Regimen 4: (non-squamous histology only):
  - Pemetrexed 500 mg/m^2 IV over 10 minutes or per institutional standard on Day 1
  - Cisplatin 75 mg/m^2 IV over 120 minutes or per institutional standard on Day 1, immediately following pemetrexed
- Regimen 5:
  - Paclitaxel 175 or 200 mg/m^2 IV over 180 minutes or per institutional standard on Day 1
  - Carboplatin AUC 5 or 6 IV over 30 minutes or per institutional standard on Day 1, immediately following paclitaxel

Arm C: Participants randomized into Arm C will receive nivolumab 360 mg IV + platinum doublet chemotherapy in 3-week cycles up to a maximum of 3 cycles of IV chemotherapy (ie, 9 weeks of treatment; each cycle is 21 days)

- Non-squamous NSCLC: nivolumab at a flat dose of 360 mg as 30-minute IV infusion on Day 1, followed by pemetrexed at a dose of 500 mg/m2 IV over 10 minutes or per institutional standard with cisplatin at a dose of 75 mg/m^2 IV over 120 minutes or per institutional standard, of a 3-week treatment cycle, for up to 3 cycles.
- Squamous NSCLC: nivolumab at a flat dose of 360 mg as 30 minute IV infusion on day 1, followed by gemcitabine at a dose of 1000 mg/m^2 or 1250 mg/m^2 (per local prescribing information) IV over 30 minutes or per institutional standard with cisplatin at a dose of 75 mg/m^2 IV over 120 minutes or per institutional standard, of a 3-week treatment cycle for up to 3 cycles. Gemcitabine will also be administered at a dose of 1000 mg/m^2 or 1250 mg/m^2

Approved v9.0 [redacted]

as a 30 minute IV infusion or per institutional standard on day 8 of each 3-week treatment cycle.

- Any histology: nivolumab at a flat dose of 360 mg as 30-minute IV infusion on Day 1, followed by paclitaxel 175 or 200 mg/m^2 IV over 180 minutes or per institutional standard and carboplatin AUC 5 or 6 IV over 30 minutes or per institutional standard of a 3-week treatment cycle, for up to 3 cycles

Study treatment:

Study Drug for CA209816		
**Medication**	**Potency**	**IP/Non-IP**
BMS-936558-01 Nivolumab	10 mg/mL	IP
Ipilimumab Solution for Injection	5 mg/mL	IP
Vinorelbine	10 mg/mL	IP
Gemcitabine	38 mg/mL	IP
Docetaxel	10 mg/mL	IP
Pemetrexed	500 mg/vial	IP
Cisplatin	1 mg/mL	IP
Carboplatin	10 mg/mL	IP
Paclitaxel	6 mg/mL	IP

Independent Data Monitoring Committee

An independent Data Monitoring Committee (DMC) will be established to provide oversight of safety and efficacy considerations, study conduct, and risk-benefit ratio in protocol CA209816. An interim DMC review will occur after 15 participants are enrolled in Arm A and after 15 participants are enrolled in Arm C and have completed surgery. Following review, the DMC will recommend continuation, modification, or discontinuation of this study based on reported safety data. Representatives of the Sponsor will serve only as coordinators of the committee, without having full member responsibilities or privileges. In addition, the Sponsor will independently review safety data in a blinded manner during the conduct of this trial to ensure that any safety issues are identified and addressed. Available efficacy data will also be reviewed by the DMC during the conduct of the study. Details of the DMC responsibilities and procedures will be specified in the DMC charter.

Approved v9.0 [redacted]

<u>Independent Radiology/Pathology Review:</u>

Independent pathology and radiology review will be established for central review and confirmation of endpoints. Images and tumor/lymph node samples will be submitted to these third-party vendors for central review. Sites will be trained prior to enrolling the first study participant. Images and pathology samples acquisition guidelines and submission process will be outlined in the study Imaging/Laboratory Manuals to be provided by the vendors.

Tumor and lymph node collection from definitive surgical resection and sampling of fresh tumor sample in RNAlater for biomarker studies (as applicable dependent on the size of the residual tumor) is mandatory on the day of surgery. RNAlater sample will not be collected in China. Processing the remainder of the specimen for histopathologic analysis should be performed within 72 hours of the procedure. Sections will be used for central pathology review assessing pathologic complete response (pCR) and major pathologic response (MPR). Any tumor, tumor bed or lymph node specimens that are reviewed locally must be submitted for central pathology review. Gross examination on the entire specimen should be performed which includes all tumor, and associated lymph node tissue and uninvolved parenchyma. The specimen should be sectioned at 0.5 cm intervals, and blocks should be submitted for the full cross section for every other 0.5 cm interval. For very large tumors with no gross evidence of response, a minimum of 1 slide/cm is required for assessment of pathologic response. When estimating viable tumor, in situ carcinoma should not be included. Pathology specimen collection and processing guidelines are outlined in the CA209816 Laboratory Manual. A blinded pathology review process will be utilized to assess for confirmation of endpoints.

Radiologic tumor assessments should be submitted to the third-party radiology vendor as they are performed on an ongoing basis.

Tumor assessments will be sent to and reviewed by a Blinded Independent Central Review (BICR) from a third-party radiology vendor on an on-going basis. At the time of investigator assessed radiographic progression per RECIST 1.1, the site must request a BICR- for confirmation of progression or disease recurrence. However, BICR confirmation of progression should not be requested if investigator judges the progression will not preclude surgery. Participants should proceed to definitive surgery and tumor assessments post surgery should be continue per schedule of activities. Details of the BICR responsibilities and procedures will be specified in the BICR charter.

Participants whose disease progression or disease recurrence is not confirmed by central review will be required to continue tumor assessments (if clinically feasible) according to the protocol-specified schedule, even after the initiation of subsequent anti-cancer therapies. Subsequent tumor assessments must be submitted to the third party radiology vendor for subsequent review and may be discontinued when the investigator and independent radiologists both assess the participant to have met RECIST 1.1 criteria for progression or disease recurrence.

Abbreviations are listed in Appendix 2.

Approved v 9.0 [redacted]

REFERENCES

[1] Mountain, CA. Regional lymph node classification for lung cancer staging. Chest. 1997: 1718-23.

[2] Goldstraw P, Crowley J, Chansky K, et al for the International Association for the Study of Lung Cancer Internatonal Staging Committee. The IASLC Lung Cancer Staging Project: proposals for the revision of the TNM stage groupings in the forthcoming (seventh) edition of the TNM Classification of malignant tumours. J Thorac Onco. 2007; 2(8):706-14.

[3] Detterbeck FC, Boffa DJ, Tanoue LT. The new lung cancer staging system. Chest. 2009; 136: 260-71.

Approved v1.0 [redacted]

2 SCHEDULE OF ACTIVITIES

Table 2-1: Screening Procedural Outline (CA209816) [a]

Procedure	Screening Visit	Notes
**Eligibility Assessments**		
Informed Consent	X	Informed Consent Form prior to screening for study participation. If a participant is re-enrolled, the participant must be re-consented, and eligibility should be re-confirmed. Register in Interactive Response Technology to obtain participant number.
Inclusion/Exclusion Criteria	X	All inclusion/exclusion criteria should be assessed at screening and confirmed prior to first dose. Participants must also meet operative criteria (Section 9.10.1).
Medical History	X	
Tumor Tissue Samples	X	Tumor tissue submission prior to randomization is mandatory. If a recent/archived (within 3 months) biopsy sample is not available at screening, a fresh biopsy will be taken.<br>Sufficient tumor tissue obtained prior to randomization (block or minimum of 15 slides, obtained from core biopsy, punch biopsy, excisional biopsy or surgical specimen). If less than 15 slides are available, then the Medical Monitor should be contacted to advise on eligibility.<br>For participants for whom a biopsy is not feasible, archival tumor material must be made available. Fine needle aspirate of draining lymph node is not acceptable. Core needle biopsies obtained by EBUS are acceptable. PD-L1 status must be determined prior to randomization.
Mediastinal lymph node sampling	X	All suspicious mediastinal lymph nodes including those that are pathologically enlarged or FDG avid on PET/CT require further sampling for pathological confirmation if accessible by EBUS, mediastinoscopy, or thoracoscopy.
Screening/Baseline Tumor Assessments	X	PET/CT including contrast from the base of the skull to the upper thighs, within 28 days prior to randomization. A separate CT with contrast of the chest, abdomen, and other suspected areas of disease (as well as the PET/CT) is required if the CT component of the PET/CT is not of sufficient diagnostic quality for RECIST 1.1 measurements.<br>Participants with stage II or higher disease and those with a suspicion of brain metastasis should have a MRI or CT of the brain pre- and post-contrast within 28 days of randomization.<br>Tumor assessments should be performed following RECIST 1.1 criteria.
Prior Medication	X	
ECOG Performance Status	X	Within 14 days prior to randomization (Appendix 3)

Approved v9.0 [redacted]

Table 2-1: Screening Procedural Outline (CA209816) [a]

Procedure	Screening Visit	Notes
**Safety Assessments**		
History and Physical Examination	X	Includes assessment of symptoms, review of system (ROS), height, weight, BSA (for platinum dosing), and full physical exam within 14 days prior to randomization.
Vital Signs and Oxygen Saturation	X	Includes body temperature, respiratory rate, and seated blood pressure, heart rate, and oxygen saturation by pulse oximetry (at rest). Obtained at the screening visit and within 72 hours prior to first dose.
Pulmonary Function Test	X	Should be performed within 6 weeks prior to randomization. Includes FVC, FEV1, TLC, FRC, and DLco.
Concomitant Medication Collection	X	Within 14 days of randomization.
Serious Adverse Events Assessment	X	Serious Adverse Events from the time of consent
Adverse Events Assessment	X	
Laboratory Tests	X	CBC w/differential, chemistry panel including: Albumin, LDH, AST, ALT, ALP, T. Bili, BUN or serum urea level, creatinine, phosphate, Ca, Mg, Na, K, Cl, glucose, amylase, lipase, TSH, T4 (free), T3 (free or total) within 14 days prior to randomization.<br>Hepatitis B surface antigen (HBsAg), Hepatitis B core antibody (HBcAb), reflex HBV DNA, hepatitis C antibody (HCV Ab), and reflex hepatitis C RNA (HCV RNA). Within 28 days prior to randomization.
ECG	X	ECG to be performed prior to randomization and recorded after the participant has been supine for at least 5 minutes.
Pregnancy Test	X	Urine or serum test (minimum sensitivity 25 IU/L or equivalent units of HCG) within 14 days prior to randomization.
**Study Treatment**		
Randomize	X	

[a] Screening assessments should occur within 28 days of randomization unless otherwise noted.

ALT, alanine aminotransferase; AST, aspartate aminotransferase; BUN, blood urea nitrogen; Ca, calcium; CBC complete blood count; Cl, chloride; DLco, diffusing capacity; EBUS, endobronchial ultrasound; ECG, electrocardiogram; FEV1, forced expiratory volume in 1 second; FVC, forced vital capacity; FRC, functional residual capacity; FVC, forced vital capacity; HBcAb, Hepatitis B core antibody; HBsAg, Hepatitis B surface antigen; HCV Ab, Hepatitis C antibody; ICF, informed consent form; LDH, lactate dehydrogenase, K, potassium; Mg, magnesium; NA, sodium; RECIST, Response Evaluation Criteria in Solid Tumors; SOC, standard of care; T.Bili, total bilirubin; TLC, total lung capacity; TSH, thyroid stimulating hormone.

Table 2-2: Neoadjuvant Period Procedural Outline (CA209816)

Procedure	During Treatment Visits[a,b]	Notes
**Safety Assessments**		
Physical Examination	X	Includes assessment of signs, symptoms, and ROS prior to each dose.
Vital Signs and Oxygen Saturation	X	Including BP, HR, temperature, respiratory rate, and oxygen saturation by pulse oximetry (at rest) prior to each dose.
Physical Measurements	X	Weight, Body Surface Area (for cytotoxic dosing), and ECOG status. The dosing calculations for treatment arms should be based on the body weight, except for nivolumab on Arm C, which will be administered using a flat dose of 360 mg. If the participant's weight on the day of dosing differs by > 10% from the weight used to calculate the previous dose, the dose must be recalculated for the dose of the day by using the participant's weight of the day, and this updated value becomes the reference for subsequent dosing calculations. All doses should be rounded to the nearest milligram or as per institutional standard.
Serious Adverse Event Assessment	X	
Adverse Events Assessment	X	
Review of Concomitant Medications	X	
Pulmonary Function Test	See notes	PFTs should be re-evaluated prior to surgery only. Includes FVC, FEV1, TLC, FRC, and DLco.
Laboratory test	X	On-study local laboratory assessments should be done within 72 hours prior to each dose, starting with Cycle 1 Day 1 (C1D1). C1D1 labs do not need to be repeated if they were performed within 14 days of dosing.<br>CBC w/differential, LFTs (ALT, AST, total bilirubin, alkaline phosphatase), BUN or serum urea level, creatinine, Ca, Mg, Na, K, Cl, LDH, glucose, amylase, lipase, albumin, and phosphate.<br>TSH with reflexive T4 (free), T3 (free or total) for Arm A and Arm C only. Repeat every 6 weeks while receiving nivolumab.
Pregnancy Test	X	Serum or urine (minimum sensitivity 25 IU/L or equivalent units of HCG) within 24 hours prior to first dose and then every 4 weeks for Arm A, every 3 weeks for Arm C, and Arm B while receiving study treatment. An extension of up to 72 hours prior to the start of study drug may be permissible in situations where results cannot be obtained within the standard 24 hours window, subject to Medical Monitor approval.

Table 2-2: Neoadjuvant Period Procedural Outline (CA209816)

Procedure	During Treatment Visits[a,b]	Notes
**Efficacy Assessments**		
Tumor Assessments	X	PET/CT including contrast of the base of the skull through the upper thighs, within 14 days prior to surgery per RECIST 1.1. A separate CT with contrast of the chest, abdomen, and all other suspected areas of disease (as well as the PET/CT) is required if the CT component of a PET/CT is not of sufficient diagnostic quality for RECIST measurements.
**Pharmacokinetic Assessments**		
PK samples[c]	X	Arm C only. Refer to Table 9.5-1
**Biomarker Assessments**		
Exploratory Biomarker	X	[redacted]
**Patient-reported Outcomes Assessment**		
EQ-5D-3L	X	For C1D1: performed after randomization PRIOR to first dose. On Day 1 of each cycle of study drug administration, the EQ-5D-3L will be administered PRIOR to treatment.
**Study Treatment**		
IRT Drug Vial Assignments	X	
Dispense Study treatment	X	Within 7 calendar days after randomization, the participants must receive the first dose of study medication. Participants no less than 18 days between doses for nivolumab on Arm C.<br>If a dose is delayed for any reason, participants should be dosed no later than 7 days following a planned dose on any arm.<br>If more than 7 days delay is needed for any reason, the intended dose should be skipped. If a participant receiving chemotherapy on a Day 1 and Day 8 schedule (ie, cisplatin/gemcitabine) is unable to receive Day 1 of chemotherapy but recovers in time to receive the Day 8 dose, the Day 8 dose of chemotherapy may be administered.
Surgery per Standard of Care	See notes	Surgery should be performed within 6 weeks after completing up to 3 cycles of neoadjuvant therapy as indicated by the institutional standard of care (SOC).<br>Doses skipped during pre-operative treatment should not be replaced.

[a] Each cycle in Arm B and Arm C is 21 days.

[b] Neoadjuvant period assessments should be performed within 72 hours prior to dosing, unless otherwise noted.

[c] Arm C only

ALT, alanine aminotransferase; AST, aspartate aminotransferase; BP, blood pressure; BUN, blood urea nitrogen; Ca, calcium; CBC complete blood count; Cl, chloride; DLco, diffusing capacity; EBUS, endobronchial ultrasound; ECG, electrocardiogram; ECOG, Eastern Cooperative Oncology Group; FEV1, forced expiratory volume in 1 second; FVC, forced vital capacity; FRC, functional residual capacity; FVC, forced vital capacity; HR, heart rate; HBcAb, Hepatitis B core antibody; HBsAg, Hepatitis B surface antigen; HCV Ab, Hepatitis C antibody; ICF, informed consent form; IRT, interactive response technology; LDH, lactate dehydrogenase, K, potassium; Mg, magnesium; NA, sodium; PFT, pulmonary function test; RECIST, Response Evaluation Criteria in Solid Tumors; SOC, standard of care; T.Bili, total bilirubin; TLC, total lung capacity; TSH, thyroid stimulating hormone.

Table 2-3: Post-Neoadjuvant Period for Those Participants Not Receiving Adjuvant Therapy (CA209816)

Procedure	Post-Neoadjuvant Therapy Visits 1 and 2[a]	Survival Follow-Up Visits [b,c]	Notes
**Safety Assessments**			
Physical Examination	X		Includes assessment of signs and symptoms and ROS.
Vital Signs and Oxygen Saturation	X		Include BP, HR, temperature, and oxygen saturation by pulse oximetry (at rest).
Serious Adverse Events Assessment	X		All AE/SAEs to be collected for up to 100 days after the last dose of neoadjuvant therapy or 90 days after surgery, whichever is longer.
Adverse Events Assessment	X		All AE/SAEs to be collected for up to 100 days after the last dose of neoadjuvant therapy or 90 days after surgery, whichever is longer.
Review of Concomitant Medications	X	X	Document subsequent cancer therapy
Laboratory Tests	X		CBC w/ differential, LFTs (ALTs, AST, total bilirubin, alkaline phosphatase), BUN or serum urea level, creatinine, Ca, Mg, Na, K, Cl, LDH, glucose, amylase, lipase, albumin, and phosphate.<br>TSH with reflex T4 (free) and T3 (free or total) for Arm A and Arm C only.<br>Laboratory tests should be done at Post-neoadjuvant Visit 1 and repeated at Post-neoadjuvant Visit 2 if study-related toxicity persists.
Pregnancy Test	X		Serum or urine (minimum sensitivity 25 IU/L or equivalent units of HCG)
**Efficacy Assessments**			
Tumor Assessments	----See Notes---		Tumor assessments with CT, including contrast, of the chest including adrenal glands and CT or MRI of other additional suspected/known sites of disease. The first tumor assessment should occur 12 weeks (±7 days) after definitive surgery per RECIST 1.1 and then should occur every 12 weeks (± 7 days) per RECIST 1.1 for 2 years (104 weeks), then every 6 months (24 weeks ±7 days) for 3 years, and every year (52 weeks ± 7 days) for 5 years or until disease recurrence or progression is confirmed by blinded independent central review (BICR).<br>For participants who did not receive definitive surgery AND without tumor progression confirmed by BICR, the first tumor assessments should be done 12 weeks (± 7 days) following the tumor restaging, subsequent tumor assessments will be performed with the same frequency and methods as described for those

Approved v1.0 [redacted]

Table 2-3: Post-Neoadjuvant Period for Those Participants Not Receiving Adjuvant Therapy (CA209816)

Procedure	Post-Neoadjuvant Therapy Visits 1 and 2[a]	Survival Follow-Up Visits [b,c]	Notes
			having received definitive surgery; in case the planned initiation of subsequent anti-cancer therapy is within 12 weeks of restaging, every effort should be made to repeat tumor assessment prior to subsequent anti-cancer therapy. Use same imaging method and a machine of the same quality as was used at screening/baseline
**Pathology and Biomarker Assessments**			
Tissue and biomarker assessments	--See notes--		Tissue collection from definitive surgical resection is mandatory for pathologic response assessments and highly recommended for biomarker assessments on the day of surgery. Tumor biopsy collection is optional but highly recommended from participants at disease progression. All tumor biopsy collection details will be provided in the lab manual. [redacted]
**Patient-reported Outcomes Assessment**			
EQ-5D-3L	X	X	Every 3 months after Post-neoadjuvant Visit 2 for 1 year and then once every 6 months thereafter. May be completed as a phone call if a clinic visit is not otherwise required.
**Participant Survival Status**			
Survival Status	X	X	Every 3 months after Post-neoadjuvant Follow-Up Visit 2. May be accomplished by visit, phone contact, or email to assess subsequent anti-cancer therapy

[a] Post-neoadjuvant Visit 1within 30 days from the last dose of neoadjuvant therapy ± 7 days and prior to surgery (this may lead to shortened interval between last dose of neoadjuvant treatment and post-neoadjuvant visit 1). If Post-neoadjuvant Visit 1 falls within 7 days of the scheduled surgery date, then the Post-neoadjuvant Visit 1 visit may coincide with the surgery admission. All Neoadjuvant Visit 1 assessment procedures should be completed prior to surgery. Post-adjuvant Visit 2 = 70 days (± 7 days) from Post-neoadjuvant Visit 1. Post-neoadjuvant visits may coincide with the date of post-surgical follow-up. Timing of Post-neoadjuvant Visits 1 and 2 are based on the pre-surgical neoadjuvant treatment timelines.

[b] Survival Follow-up Visits begin 3 months after Post-neoadjuvant Visit 2. Visits should occur every 3 months (± 7 days).

[c] For those participants who have progression confirmed by BICR, participants will continue to be followed for survival status every 3 months by visit, phone contact, or email.

ALT, alanine aminotransferase; AST, aspartate aminotransferase; BUN, blood urea nitrogen; Ca, calcium; CBC complete blood count; Cl, chloride; DLco, diffusing capacity; EBUS, endobronchial ultrasound; ECG, electrocardiogram; FEV1, forced expiratory volume in 1 second; FVC, forced vital capacity; FRC, functional residual capacity; FVC, forced vital capacity; HBcAb, Hepatitis B core antibody; HBsAg, Hepatitis B surface antigen; HCV Ab, Hepatitis C antibody; ICF, informed consent form; LDH, lactate dehydrogenase, K, potassium; Mg, magnesium; NA, sodium; RECIST, Response Evaluation Criteria in Solid Tumors; SOC, standard of care; T.Bili, total bilirubin; TLC, total lung capacity; TSH, thyroid stimulating hormone.

Approved v9.0 [redacted]

Table 2-4: Post-Neoadjuvant Period for Participants Receiving Adjuvant Chemotherapy (CA209816)

Procedure	During Adjuvant Treatment Visit[a]	Post Neoadjuvant Therapy Visits 1 and 2[b]	Survival Follow-Up Visits[c,d]	Notes
**Safety Assessments**				
Physical Measurements	X			Weight, BSA (for cytotoxic dosing), and ECOG status prior to each dose. The dosing calculations for the platinum doublet chemotherapy arm should be based on the body weight. If the participant’s weight on the day of dosing differs by > 10% from the weight used to calculate the previous dose, the dose must be recalculated for the dose of the day using the participant’s weight of the day, and this updated value becomes the reference for subsequent dosing calculations. All doses should be rounded to the nearest milligram or per institutional standard.
Physical Examination	X	X		Includes assessment of signs, symptoms, and ROS prior to each dose.
Vital Signs and Oxygen Saturation	X	X		Include BP, HR, temperature, and oxygen saturation by pulse oximetry (at rest) prior to each dose
Serious Adverse Events Assessment	X	X		All AEs/SAEs to be collected for up to 30 days after the last dose of adjuvant therapy.
Adverse Event Assessments	X	X		All AEs/SAEs to be collected for up to 30 days after the last dose of adjuvant therapy
Review Concomitant Medication	X	X	X	Document subsequent cancer therapy.
Laboratory test	X	X		On-study local laboratory assessments should be done within 72 hours prior to each dose and at Post-neoadjuvant Visit 1. To be repeated at Post-neoadjuvant Visit 2 if study-related toxicity persists.<br>CBC w/ differential, albumin, LFTs (ALT, AST, total bilirubin, alkaline, phosphatase), BUN or serum urea level, creatinine, Ca, Mg, Na, K, Cl, LDH, glucose, amylase, lipase, phosphate.<br>TSH with reflex T4 (free) and T3 (free or total) for Arm A and Arm C only. To be conducted only at Post-neoadjuvant Therapy

Approved v1.0 [redacted]

Table 2-4: Post-Neoadjuvant Period for Participants Receiving Adjuvant Chemotherapy (CA209816)

Procedure	During Adjuvant Treatment Visit[a]	Post Neoadjuvant Therapy Visits 1 and 2[b]	Survival Follow-Up Visits[c,d]	Notes
				Visit 1 and repeated at Post-Neoadjuvant Therapy Visit 2 if study-related toxicity persists.
Pregnancy test	X	X		Serum or urine (minimum sensitivity 25 IU/L or equivalent units of HCG) within 24 hours prior to first dose and then every 4 weeks while receiving study treatment.<br>An extension of up to 72 hours prior to the start of study drug may be permissible in situations where results cannot be obtained within the standard 24 hours window, subject to Medical Monitor approval.
**Efficacy Assessments**				
Tumor Assessments	---See Notes---			Tumor assessments using CT, with contrast, of the chest including adrenal glands and CT or MRI of other additional suspected/known sites of disease.<br>The first tumor assessment should occur 12 weeks (± 7 days) after definitive surgery per RECIST 1.1 and then should occur every 12 weeks (± 7 days) per RECIST 1.1 for 2 years (104 weeks), then every 6 months (24 weeks ± 7 days) for 3 years, and every year (52 weeks ± 7 days) for 5 years or until disease recurrence or progression is confirmed by BICR.<br>Use same imaging methods and machine of the same quality as was used at screening/baseline.
**Biomarker Assessment**				
Exploratory Biomarker	---See Notes---			Tumor biopsy collection is optional but highly recommended from participants at disease progression.<br>All tumor biopsy collection details will be provided in the lab manual.<br>[redacted]

Table 2-4: Post-Neoadjuvant Period for Participants Receiving Adjuvant Chemotherapy (CA209816)

Procedure	During Adjuvant Treatment Visit[a]	Post Neoadjuvant Therapy Visits 1 and 2[b]	Survival Follow-Up Visits[c,d]	Notes
**Patient-reported Outcomes Assessment**				
EQ-5D-3L	X	X	X	Every 3 months after Post-neoadjuvant Visit 2 for 1 year and then once every 6 months thereafter. May be completed as a phone call if a clinical visit is not otherwise specified.
**Participant Survival Status**				
Survival Status	X	X	X	Every 3 months after Post-neoadjuvant Follow-Up Visit 2. May be accomplished by visit, phone contact, or email to assess subsequent anti-cancer therapy
**Study Treatment**				
Administer Adjuvant Chemotherapy ±PORT	X			Post-operative radiation therapy (PORT) should be administered according to standard of care.

[a] Each cycle is 21 days. Assessments should be performed within 72 hours prior to dosing of adjuvant therapy.

[b] Post-neoadjuvant Visit 1 within 30 days from the last dose of neoadjuvant therapy ± 7 days and prior to surgery (this may lead to shortened interval between last dose of neoadjuvant treatment and post-neoadjuvant visit 1). If Post-neoadjuvant Visit 1 falls within 7 days of the scheduled surgery date, then the Post-neoadjuvant Visit may coincide with the surgery admission. All Post-neoadjuvant Visit 1 assessment procedures should be completed prior to surgery. Post-neoadjuvant Visit 2 = 70 days (± 7 days) from Post-neoadjuvant Visit 1. Post-neoadjuvant Visit 2 may coincide with the date of post-surgical follow-up. Timing of Post-neoadjuvant Visits 1 and 2 are based on the pre-surgical neoadjuvant therapy timelines.

[c] Survival Follow-up Visits begin 3 months after Post-neoadjuvant Visit 2 or after completion of adjuvant therapy, whichever occurs later. Visits should occur every 3 months (± 7 days).

[d] For those participants who have progression confirmed, participants will continued to be followed for survival status every 3 months by visit, phone contact, or email.

ALT, alanine aminotransferase; AST, aspartate aminotransferase; BP, blood pressure; BUN, blood urea nitrogen; Ca, calcium; CBC complete blood count; Cl, chloride; HR, heart rate; ECOG, Eastern Cooperative Oncology Group; DLco, diffusing capacity; EBUS, endobronchial ultrasound; ECG, electrocardiogram; FEV1, forced expiratory volume in 1 second; FVC, forced vital capacity; FRC, functional residual capacity; FVC, forced vital capacity; HBcAb, Hepatitis B core antibody; HBsAg, Hepatitis B surface antigen; HCV Ab, Hepatitis C antibody; ICF, informed consent form; LDH, lactate dehydrogenase, K, potassium; Mg, magnesium; NA, sodium; RECIST, Response Evaluation Criteria in Solid Tumors; SOC, standard of care; T.Bili, total bilirubin; PORT post-operative radiation therapyTLC, total lung capacity; TSH, thyroid stimulating hormone

3 INTRODUCTION

Nivolumab (BMS-936558) is a fully human, IgG4 (kappa) isotype mAb that binds PD-1 on activated immune cells and disrupts engagement of the receptor with its ligands PD-L1 (B7 H1/CD274) and PD-L2 (B7-DC/CD273), thereby abrogating inhibitory signals and augmenting the host antitumor response. In early clinical trials, nivolumab has demonstrated activity in several tumor types, including melanoma, renal cell carcinoma (RCC), and non-small cell lung cancer (NSCLC).

CTLA-4, an activation-induced T-cell surface molecule, is a member of the CD28:B7 immunoglobulin superfamily that competes with CD28 for B7. The proposed mechanism of action for ipilimumab is interference of the interaction of CTLA-4 with B7 molecules on APCs, with subsequent blockade of the inhibitory modulation of T-cell activation promoted by the CTLA 4/B7 interaction.

Nivolumab is in clinical development for the treatment of patients with NSCLC, RCC, melanoma, squamous cell carcinoma of the head and neck (SCCHN) and other tumors (eg, glioblastoma multiforme, mesothelioma, small cell lung cancer, gastric). The combination of nivolumab and ipilimumab is in clinical development for the treatment of NSCLC, RCC, and other tumors. Opdivo® is approved in the United States (US), European Union, and other countries for the treatment of patients with unresectable or metastatic melanoma, advanced NSCLC with progression on or after platinum-based chemotherapy, advanced RCC whose disease progressed on an antiangiogenic therapy (US only), classical Hodgkin lymphoma that has relapsed or progressed after autologous hematopoietic stem cell transplantation and post-transplantation brentuximab vedotin treatment (US only), head and neck carcinoma (US only), and bladder cancer (US only). Opdivo is also approved in combination with Yervoy® in unresectable and metastatic melanoma.

3.1 Study Rationale

Approximately 80% of lung cancer cases are NSCLC, with most patients presenting with late-stage disease. Of patients with NSCLC, 20% present with stage I or II disease, whereas 30% present with stage III disease and 50% with stage IV disease. With enhanced lung cancer screening techniques, the percentage of those diagnosed during the early stages may increase over the duration of the trial. A standard TNM staging system is used to determine the staging for NSCLC (Appendix 1). Patients with pathologic stage I NSCLC have a 5-year survival of approximately 60%. Stage II to III NSCLC patients have a 5-year survival of approximately 25% to 40%.[1] Surgical resection remains the mainstay of treatment for stage I and II patients; however, despite potential curative surgery, approximately 50% of stage IB and 60-75% of stage I-II NSCLC patients will relapse and eventually die of their disease.[2,3] A rational approach to improve survival in these patients is to eradicate micrometastatic disease and to minimize the risk of relapse with adjuvant or neoadjuvant chemotherapy. Many adjuvant studies have been performed, and these trials are summarized in Table 3.1-1. Although there are some conflicting results, the overall evidence from these studies suggests that adjuvant platinum doublet chemotherapy is beneficial for good Performance Status patients with stage ≥ I disease. The benefit to stage IB patients is less

Approved v[illegible].0 [redacted]

clear and may depend on the size of the primary tumor and other risk factors. The LACE meta-analysis of modern adjuvant and neoadjuvant trials, all of which used cisplatin-based chemotherapy, suggested a 5% absolute survival advantage at 5 years from adjuvant chemotherapy with the benefit being greatest for stage II and IIIA patients. A 2010 meta-analysis including both older and more recent trials confirmed the survival benefit shown in the LACE meta-analysis and also suggested a benefit of adjuvant chemotherapy for stage IB disease.[4]

Table 3.1-1: Select Adjuvant NSCLC Studies

Trial	Stage	Treatment	# of Pts	5 Yr OS	HR	P Value
ECOG 1505	I-III	Cis-doublet<br>Cis-double+Bev	749<br>752	72 mo mOS in both arms	0.99	0.9
ALPI	I - III	Surg<br>MVP	603<br>601	45%<br>50%	0.96	0.59
IALT	I – III	Surg<br>Cis-based	935<br>932	40%<br>44.5%	0.86	<0.03
ANITA	IB - IIIA	Surg<br>Cis-Vin	433<br>407	43%<br>51%	0.80	0.017
BLT	I - IIIA	Surg<br>Cis-based	189<br>192	58%<br>60%	1.02	0.90
NCIC/JBR10	IB – II	Surg<br>Cis-Vin	240<br>242	54%<br>69%	0.69	0.03
CALGB	IB	Surg<br>Carb-pac	171<br>173	57%<br>59%	0.80	0.10
ALPI	I - III	Surg<br>MVP	603<br>601	45%<br>50%	0.96	0.59

ALPI, Adjuvant Lung Cancer Project Italy; OS, overall survival; HR, hazard ratio; ECOG, Eastern Cooperative Oncology Group, IALT, International Adjuvant Lung Cancer Trial; ANITA, Adjuvant Navelbine International Trialist Association; BLT, Big Lung Trial; NCI-C, National Cancer Institute-Canada; CALGB, Cancer and Leukemia Group B; MVP, mitomycin, vindesine, cisplatin; Cis-Vin, cisplatin-vinorelbine; Carb-pac, carboplatin-paclitaxel.

The early-stage (IB-III) NSCLC represents a population of high unmet need with a 5-year survival rate of 25-50%.[2,3,5] The current available standard of care (SOC) only provides a 5% absolute improvement in 5-year overall survival (OS).[6,7,8,9] The SOC comprises adjuvant or neoadjuvant platinum doublet chemotherapy for patients with operable stage IB-IIIA NSCLC or chemoradiation for patients with unresectable stage IIIA/B NSCLC. Follow-up of adjuvant trials are long and may require decades before a new treatment is introduced into clinical practice. Preoperative or neoadjuvant chemotherapy has been assessed in a number of trials for patients with operable NSCLC. A meta-analysis based on 7 trials involving 988 patients suggested that neoadjuvant chemotherapy improved OS when given preoperatively in a similar magnitude to

Approved v9.0 [redacted]

those observed with adjuvant chemotherapy.[10] Neoadjuvant therapy offers the possibility for the identification of surrogate clinical and biological markers that may correlate with response to therapy and a potential long-term outcome. Studies that address the role of preoperative chemotherapy have found chemotherapy compliance to be favorable in the preoperative setting.[6,7,8] Randomized trials are currently on-going to explore the potential benefit of nivolumab in the adjuvant and locally advanced unresectable NSCLC setting. In addition, the safety and efficacy profile of neoadjuvant nivolumab monotherapy or in combination (nivolumab plus ipilimumab or nivolumab plus chemotherapy) are being evaluated in ongoing trials.

The phase 3 study, CA209816, will evaluate the clinical efficacy and will establish the safety of nivolumab plus ipilimumab, especially nivolumab plus platinum doublet chemotherapy, in resectable lung cancer. Specifically, this study will compare EFS and pCR rate among participants treated with neoadjuvant nivolumab plus platinum doublet chemotherapy vs participants treated with platinum doublet chemotherapy, and will describe pCR rate and EFS for those treated with neoadjuvant nivolumab plus ipilimumab in Stage Ib-IIIa NSCLC.

3.1.1 Research Hypothesis

In participants with stage IB (≥ 4 cm), II or IIIA (N2) NSCLC considered resectable by the local multidisciplinary team, administration of neoadjuvant nivolumab plus platinum doublet chemotherapy (up to 3 cycles) has superior efficacy to neoadjuvant platinum doublet chemotherapy (up to 3 cycles).

3.2 Background

3.2.1 Indication Background

Approval of nivolumab in advanced NSCLC was based on 2 phase 3 trials (CheckMate 017 and CheckMate 057) which demonstrated survival benefit over docetaxel across histologies. The approval in squamous NSCLC was based on the results of CA209017, a randomized trial of nivolumab versus docetaxel. The median OS for patients in the nivolumab arm was 9.2 months versus 6 months for those in the docetaxel arm (HR = 0.59). Improvement in survival was observed for nivolumab regardless of PD-L1 expression, though there was a trend toward better efficacy for those with PD-L1 expressing tumors. A single-arm trial (CA209063) of 117 patients with metastatic squamous NSCLC with progression after platinum-based chemotherapy and at least 1 additional systemic regimen showed a 15% objective response rate (ORR); 59% of participants with an ORR had response durations of 6 months or longer.[11]

The approval of nivolumab for the treatment of non-squamous NSCLC is based on a second phase 3 study, CA209057, which met its primary endpoint of superior OS of nivolumab versus docetaxel in patients with previously treated non-squamous NSCLC at a preplanned interim analysis (IA). Patients in the nivolumab arm had a 27% reduction in risk of death (HR = 0.73; P = 0.0015). Interaction P values, reported for PD-L1 expression subgroups by each of the predefined expression levels, suggested a clinically important signal of a predictive association. Nivolumab also significantly improved ORR vs docetaxel (P=0.0246), with ORR as high as 36% in patients with PD-L1 expressing tumors. OS approximately doubled with nivolumab vs docetaxel at 1%,

Approved v 9.0 [redacted]

5% and 10% PD-L1 expression level. In contrast, no statistically significant difference in OS was seen between nivolumab and docetaxel when PD-L1 was not expressed in the tumor, although these patients also experienced durable responses, and the safety profile was more favorable for nivolumab vs docetaxel.[12]

Although the first-line CA209026 trial did not demonstrate that single agent nivolumab provided superior efficacy over platinum doublet chemotherapy, the efficacy of nivolumab was similar to that of chemotherapy in this first line patient population. In the ongoing study CA209012, 77 patients were treated with nivolumab 3 mg/kg Q2W + ipilimumab 1 mg/kg Q12W or Q6W. Overall response rates were observed in 43% of all treated patients and in 57% and 92% of patients with ≥ 1% and ≥ 50% tumor PD-L1 expression, respectively. Six investigator-assessed CRs (8%) and 3 pathologic responses were observed. The median duration of response (DOR) was not reached in the overall population or subpopulations. Duration of response ranged from 1.4+ to 27.9+ months.[13] Recent 2-year OS data in CA209012 showed survival data in all treated patients and by PD-L1 expression. All treated patients (n = 77) showed a 1 years OS of 76% and 2 year OS of 49%. When analyzed by PD-L1 expression, patients with ≥ 1% PD-L1 (n = 47) had a 1 years OS of 87% and 2 year OS of 58%, which increased to 100% and 62%, respectively for ≥ 50% PD-L1 patients. Nivolumab plus ipilimumab remained tolerable, as most treatment-related AEs were manageable and no new safety concerns were identified in follow-up.[14]

The ongoing phase 3 CheckMate 227 study enrolled patients with stage IV or recurrent NSCLC that was not previously treated. Those with a level of tumor PD-L1 expression of at least 1% were randomly assigned, in a 1:1:1 ratio, to receive nivolumab plus ipilimumab, nivolumab monotherapy, or chemotherapy; those with a tumor PD-L1 expression level of less than 1% were randomly assigned, in a 1:1:1 ratio, to receive nivolumab plus ipilimumab, nivolumab plus chemotherapy, or chemotherapy. Tumor mutational burden was determined by the FoundationOne CDx assay. Recently, in the pre-specified analysis of patients with high tumor mutational burden at a prospective cutoff of ≥ 10 mutations/MB, progression-free survival was significantly longer in the group treated by nivolumab plus ipilimumab compared to chemotherapy, demonstrating a median PFS of 7.2 months vs 5.5 months, respectively (HR 0.58; 97.5% CI 0.41, 0.81) (Nivolumab plus ipilimumab in lung cancer with a high tumor mutational burden.[15] The treatment effect was consistent in a pre-specified multi-variate analysis when adjusted for PD-L1 expression, histology, and other clinical factors. The ORR was also significantly greater for the nivolumab plus ipilimumab group (45.3% vs 26.9%; treatment difference 18.4%; 95% CI (7.6, 28.8), and the responses appeared durable with 68% of responses ongoing after 1 year and the DOR not reached (95% CI 12.2, NR) compared to 5.4 months (95% CI 4.2,6.9) for chemotherapy. In patients with TMB <10 mut/Mb, ORR was 24.6% in nivolumab plus ipilimumab arm and 25.9% in chemotherapy arm, HR for PFS being 1.07 (95% CI: 0.84, 1.35). In all randomized patients (irrespective of TMB or PD-L1 status), 1-year PFS was also higher with nivolumab plus ipilimumab than with chemotherapy [30.9% vs 17%; HR 0.83 (95% CI, 0.72 to 0.96)]. The rate of grade 3 or 4 treatment-related adverse events in all treated population (regardless of TMB or PD-L1 status) was 31.2% with nivolumab plus ipilimumab and 36.1% with chemotherapy, it was

Approved v 9.0 [redacted]

slightly higher with nivolumab plus ipilimumab in patients with high TMB tumor (37%), but the safety profile was overall consistent with previously reported data with no new safety signals.

Nivolumab added to chemotherapy has been evaluated in several cohorts of chemotherapy-naïve subjects with advanced NSCLC in CA209012.[16] Nivolumab 10 mg/kg was combined with gemcitabine and cisplatin or pemetrexed and cisplatin. Nivolumab 10 mg/kg and 5 mg/kg was combined with paclitaxel and carboplatin. The safety profile of nivolumab plus platinum-doublet chemotherapy reflects additive toxicities of the individual agents, which were manageable using established safety guidelines. No dose-limiting toxicities were observed during first 6 weeks of treatment. The overall response rate across all the nivolumab and chemotherapy cohorts range from 33-47% and median duration of response was 27.3 weeks. The 1-year survival rate for all cohorts combined is 71%.

In the ongoing CheckMate 227 trial, data in patients with tumor of <1% PD-L1 expression became also available recently.[17] In the setting of first line NSCLC with <1% tumor PD-L1 expression, PFS was improved with nivolumab plus chemotherapy vs chemotherapy (mPFS: 5.6m vs 4.7m; HR=0.74 [95% CI: 0.58 to 0.94]), mPFS of nivolumab plus ipilimumab was 4.4 months (95%CI: 3.1 to 6.0). ORR was 36.7% in nivolumab plus chemotherapy arm, 23.1% in chemotherapy arm, and 25.1% in nivolumab plus ipilimumab arm. mDOR was 7.2 months in nivolumab plus chemotherapy arm, 4.7 months in chemotherapy arm, and 18.0 months in nivolumab plus ipilimumab arm. The rate of grade 3 or 4 treatment-related adverse events was 52% with nivolumab plus chemotherapy, 35% with chemotherapy, and 25% with nivolumab plus ipilimumab, overall, for nivolumab plus chemotherapy, the safety profile and efficacy are consistent with previously reported data as well as data from other PD-(L)1 blockades in combination with chemotherapy.

Both nivolumab plus ipilimumab and nivolumab plus chemotherapy compare favorably in terms of the expected clinical benefit including response rate and overall survival observed with platinum doublet chemotherapy in first line NSCLC. These data suggest that nivolumab plus ipilimumab or nivolumab plus platinum doublet chemotherapy have the potential to provide superior pathologic response and event-free survival compared to platinum doublet chemotherapy as neoadjuvant therapy in early-stage NSCLC.[18]

In general, nivolumab plus ipilimumab or nivolumab plus platinum doublet chemotherapy is well tolerated, with a favorable safety profile relative to anticipated toxicities based on an immunostimulatory mechanism of action. Nivolumab plus ipilimumab and nivolumab plus platinum doublet chemotherapy are currently in phase 3 development in the first-line metastatic and as monotherapy in the early stage NSCLC settings.

As neoadjuvant therapy, in Stage I-III NSCLC, in ongoing phase 2 trial (Section 5.4.3) nivolumab as monotherapy showed promising anti-tumor activity in terms of depth of pathological response while the potential benefit of neoadjuvant nivolumab plus ipilimumab are still under study. On the other hand, preliminary data from ongoing phase 2 trial (NADIM, Section 5.4.3) of neoadjuvant nivolumab plus platinum doublet chemotherapy showed striking pCR rate. Preliminary experience gained from these ongoing phase 2 trials with neoadjuvant nivolumab and nivolumab-based

Approved v 9.0 [redacted]

combinations suggests these study treatments were well tolerated with little adverse impact to the feasibility of surgery. The platinum doublet chemotherapy regimens have well described safety profiles, characterized by myelosuppression and other regimen-specific non-hematologic toxicities, such as peripheral neuropathy, nausea/vomiting, and renal impairment.

A detailed description of the chemistry, pharmacology, efficacy, and safety of nivolumab is provided in the Investigator's Brochure and local package insert.

3.2.2 Nivolumab Mechanism of Action

Cancer immunotherapy rests on the premise that tumors can be recognized as foreign rather than as self and can be effectively attacked by an activated immune system. An effective immune response in this setting is thought to rely on immune surveillance of tumor antigens expressed on cancer cells that ultimately results in an adaptive immune response and cancer cell death. Meanwhile, tumor progression may depend upon acquisition of traits that allow cancer cells to evade immunosurveillance and escape effective innate and adaptive immune responses.[19,20,21] Current immunotherapy efforts attempt to break the apparent tolerance of the immune system to tumor cells and antigens by either introducing cancer antigens by therapeutic vaccination or by modulating regulatory checkpoints of the immune system. T-cell stimulation is a complex process involving the integration of numerous positive as well as negative co-stimulatory signals in addition to antigen recognition by the T-cell receptor.[22] Collectively, these signals govern the balance between T-cell activation and tolerance.

PD-1 is a member of the CD28 family of T-cell co-stimulatory receptors that also includes CD28, CTLA 4, ICOS, and BTLA.[23] PD-1 signaling has been shown to inhibit CD-28-mediated upregulation of IL-2, IL-10, IL-13, interferon-γ (IFN-γ) and Bcl-xL. PD-1 expression has also been noted to inhibit T cell activation, and expansion of previously activated cells. Evidence for a negative regulatory role of PD-1 comes from studies of PD-1 deficient mice, which develop a variety of autoimmune phenotypes.[24] These results suggest that PD-1 blockade has the potential to activate anti-self T-cell responses, but these responses are variable and dependent upon various host genetic factors. Thus, PD-1 deficiency or inhibition is not accompanied by a universal loss of tolerance to self-antigens.

In vitro, nivolumab (BMS-936558) binds to PD-1 with high affinity (EC50 0.39-2.62 nM), and inhibits the binding of PD-1 to its ligands PD-L1 and PD-L2 (IC50 ± 1 nM). Nivolumab binds specifically to PD-1 and not to related members of the CD28 family such as CD28, ICOS, CTLA-4 and BTLA. Blockade of the PD-1 pathway by nivolumab results in a reproducible enhancement of both proliferation and IFN-γ release in the mixed lymphocyte reaction (MLR). Using a CMV restimulation assay with human PBMC, the effect of nivolumab on antigen specific recall response indicates that nivolumab augmented IFN-γ secretion from CMV specific memory T cells in a dose-dependent manner versus isotype-matched control. In vivo blockade of PD-1 by a murine analog of nivolumab enhances the anti-tumor immune response and result in tumor rejection in several immunocompetent mouse tumor models (MC38, SA1/N, and PAN02).[25]

Approved v9.0 [redacted]

3.2.3 Ipilimumab Mechanism of Action

CTLA-4, an activation-induced T-cell surface molecule, is a member of the CD28:B7 immunoglobulin superfamily that competes with CD28 for B7. CTLA-4 mediated signals are inhibitory and turn off T cell-dependent immune responses.[26] Ipilimumab is a fully human monoclonal IgG1κ that binds to the CTLA-4 antigen expressed on a subset of T cells from human and nonhuman primates. The proposed mechanism of action for ipilimumab is interference of the interaction of CTLA-4 with B7 molecules on APCs, with subsequent blockade of the inhibitory modulation of T-cell activation promoted by the CTLA 4/B7 interaction.

3.2.4 Nivolumab Combined with Ipilimumab Clinical Activity

CA209012 (Checkmate 012) is a multi-arm phase 1b trial evaluating the safety and tolerability of nivolumab in patients with chemotherapy-naïve advanced NSCLC, as either a monotherapy or in combination with other agents including ipilimumab, at different doses and schedules. The primary endpoint of the study was safety with secondary endpoints of objective response rates (ORR) and 24-week PFS. Exploratory endpoints included OS and efficacy by PD-L1 expression. In the study, patients were tested for PD-L1 and 69% had evaluable PD-L1 expression. Participants were enrolled to receive nivolumab 3 mg/kg Q2W + ipilimumab 1 mg/kg Q12W or Q6W (n = 77), and nivolumab 3 mg/kg Q2W (n = 52). ORR was 43% in all treated patients and 57% and 92% in patients with ≥ 1% and ≥ 50% tumor PD-L1 expression, respectively, 6 investigator-assessed CRs (8%); including 3 pathologic responses were achieved in the nivolumab 3 mg/kg Q2W + ipilimumab 1 mg/kg Q12W/Q6W cohorts. The median duration of response (DOR) was not reached in the overall population or subpopulations. Duration of response ranged from 1.4+ to 27.9+ months.

The rate of treatment-related adverse events in the Q12W/Q6W (79%) arms were comparable to monotherapy (73%). In the study, Grades 3 to 4 AEs were 36% and 19% for the Q12W/Q6W and nivolumab monotherapy arms, respectively. Treatment-related Grade 3-4 AEs led to discontinuation in 8% and 12% of patients in the Q12W/Q6W cohorts and nivolumab monotherapy arms, respectively. There were no treatment-related deaths. The treatment-related select AEs in patients across all treatment arms were skin-related (31%), gastrointestinal (19%), endocrine (18%), and pulmonary (6%).

As described in Section 3.2.1, ongoing phase 3 CheckMate 227 study randomized patients with stage IV or recurrent NSCLC that was not previously treated in various study treatment arms by tumor PD-L1 status. Recently, in the pre-specified analysis of patients with high tumor mutational burden at a prospective cutoff of ≥ 10 mutations/MB, progression-free survival was significantly longer in the group treated by nivolumab plus ipilimumab compared to chemotherapy, HR of PFS being 0.58 (97.5% CI 0.41, 0.81). The treatment effect was consistent and independent of PD-L1 expression, or histology. The ORR was also significantly greater for the nivolumab plus ipilimumab group (45.3% vs 26.9%) and the responses appeared more durable with median DoR not reached (95% CI 12.2, NR) compared to 5.4 months (95% CI 4.2, 6.9) for chemotherapy. In patients with TMB <10 mut/Mb, similar efficacy was seen in terms of PFS and ORR between nivolumab plus ipilimumab and chemotherapy. The safety profile was overall consistent with

previously reported data with no new safety signals. In another readout focusing on patients with tumor of <1% PD-L1 expression, nivolumab plus ipilimumab showed similar efficacy to chemotherapy in terms of PFS and ORR but with much longer DoR, with a consistent safety profile of previously reported data.

As of revised protocol 03, the nivolumab plus ipilimumab has stopped enrollment.

3.2.5 Immuno-oncology Treatment Combined with Chemotherapy Clinical Activity

The combination of immuno-oncology agents and traditional chemotherapy has been explored. In addition to the additive effects of combining drugs with different mechanisms of action, emerging evidence indicates that one of the mechanisms of actions of chemotherapy is via activation of the immune system through multiple pathways.[13] The subsequent cytotoxic cell death from chemotherapies and subsequent antigen release may provide immune stimulation. Certain chemotherapy regimens have a broad effect on cell death and physiological response. As an example, cyclophosphamide reduces the number of circulating Treg cells, which are a key component in immunosuppression and gemcitabine have also been shown to reduce MDSCs and hence interferon-gamma, which have inhibitory roles in the immune response.[27,28] The dual role of cytotoxicity and immune activation from chemotherapy has provided the biological rationale for the development of combinations with immunotherapy.[29]

Nivolumab added to chemotherapy has been evaluated in several cohorts of chemotherapy-naive patients with advanced NSCLC in study Checkmate 012. Nivolumab 10 mg/kg was combined with gemcitabine and cisplatin or pemetrexed + cisplatin. Nivolumab 10 mg/kg and 5 mg/kg was combined with paclitaxel and carboplatin.

The safety profile of nivolumab plus platinum-doublet chemotherapy reflects additive toxicities of the individual agents, which were manageable using established safety guidelines. No dose-limiting toxicities were observed during first 6 weeks of treatment. The frequency of most immune-related select AEs was higher for the combination than what has been observed for nivolumab monotherapy. However, these treatment-related AEs, including pneumonitis, were effectively managed and did not lead to any deaths. Pneumonitis of any grade was reported in 7 subjects (13%): Grade 3-4 in 4 subjects (7%). Twelve (21%) subjects discontinued due to treatment-related AEs (Table 3.2.5-1).

Table 3.2.5-1: Safety in CA209012

	Total (N=56)		
	**All Grades**	**Grade 3**	**Grade 4**
Subjects with any treatment-related AE, % (n)	95 (53)	41 (23)	4 (2)[a]
Treatment-related AE in >15% of Patients, % (n)			
Fatigue	71(40)	5 (3)	0

Approved v 1.0 [redacted]

Table 3.2.5-1: Safety in CA209012

	Total (N=56)		
	All Grades	Grade 3	Grade 4
Nausea	46 (26)	2 (1)	0
Decrease Appetite	36 (20)	2 (1)	0
Alopecia	30 (17)	0	0
Anemia	27 (15)	4 (2)	0
Rash	27 (15)	2 (1)	0
Arthralgia	21 (12)	0	0
Diarrhea	21 (12)	2 (1)	0
Constipation	20 (11)	0	0
Peripheral Neuropathy	20 (11)	0	0

a Grade 4 events: neutrophil count decreased (n = 1), pneumonitis and neutropenia (n = 1 each; occurred in the same patient).

The overall response rate across all the nivolumab and chemotherapy cohorts ranged from 33-47% and median duration of response was 27.3 weeks (see Table 3.2.5-2). Activity was evaluated by PD-L1 expression and was observed in subjects with both PD-L1 expressing and non-expressing tumors. Overall, 79% (44/56) of subjects had evaluable tumor samples. At the ≥ 1% expression level, the response rate was 48% and 43% for expressers and non-expressers, respectively. The 1-year OS was 70% and 76% for expressers and non-expressers, respectively.

Table 3.2.5-2: Efficacy of First-Line Treatment of Nivolumab/Chemotherapy Combination in CA209012

Efficacy of First-Line Treatment of Nivolumab/Chemotherapy Combination in CA209012				
	Nivolumab 10 mg/kg			Nivolumab 5 mg/kg
	Gem/Cis (n=12)	Pem/Cis (n=15)	Pac/Carb (n=15)	Pac/Carb (n=14)
ORR, %	33	47	47	43
SD, %	58	47	27	43
Median Duration of Response, Weeks	45	24.4	27.3	27.3
12-mo OS rate, %	50	87	72	86
18-mo OS Rate, %	33	60	40	62
Median OS, Weeks	51	83	65	Not Reached

Approved v 1.0 [redacted]

As described in Section 3.2.1 in the ongoing CheckMate 227 trial, in the setting of first line NSCLC with <1% tumor PD-L1 expression, PFS was improved with nivolumab plus chemotherapy vs chemotherapy (mPFS: 5.6m vs 4.7m; HR=0.74 [95% CI: 0.58 to 0.94]), mPFS of nivolumab plus ipilimumab was 4.4 months (95%CI: 3.1 to 6.0). ORR was 36.7% in nivolumab plus chemotherapy arm, 23.1% in chemotherapy arm, and 25.1% in nivolumab plus ipilimumab arm. mDOR was 7.2 months in nivolumab plus chemotherapy arm, 4.7 months in chemotherapy arm, and 18.0 months in nivolumab plus ipilimumab arm. The rate of grade 3 or 4 treatment-related adverse events was 52% with nivolumab plus chemotherapy, 35% with chemotherapy, and 25% with nivolumab plus ipilimumab. Overall, the safety profile and efficacy are consistent with previously reported data for nivolumab plus chemotherapy, as well as data from other PD-(L)1 blockades in combination with chemotherapy.

3.3 Benefit/Risk Assessment

The early-stage (IB-III) NSCLC represents a population of high unmet need with a 5-year survival rate of 25-50%. The current available SOC, including adjuvant or neoadjuvant platinum doublet chemotherapy, only provides a 5% absolute improvement in 5-year OS.

Follow-up of adjuvant trials are long and may require decades before a new treatment is introduced into clinical practice. Preoperative or neoadjuvant chemotherapy has been assessed in a number of trials for participants with operable NSCLC. A meta-analysis based on 7 trials involving 988 participants suggested that neoadjuvant chemotherapy improved OS when given preoperatively in a similar magnitude to those observed with adjuvant chemotherapy. Several studies have also shown preoperative cytotoxic chemotherapy to be safe prior to surgical resection of NSCLC with no difference in extent of surgical procedures performed, operative morbidity and mortality.

The clinical activity of nivolumab observed to date in NSCLC, including 2 positive phase 3 studies demonstrating prolonged survival with nivolumab monotherapy compared to docetaxel in squamous and non-squamous NSCLC after platinum failure, suggests the potential for improved clinical outcomes. CA209057 (non-squamous NSCLC) study demonstrated OS was superior for participants receiving nivolumab compared to those receiving docetaxel. In this study, interaction P values reported for PD-L1 expression subgroups by each of the pre-defined expression levels suggested a clinically important signal of a predictive association. Higher confirmed ORRs in PD-L1 expressors were seen in the combination arm compare to the nivolumab monotherapy arm in CA209012. Based on these data, CA209816 will stratify participants based on PD-L1 status, disease stage at randomization, and gender.

In CA209012, nivolumab plus ipilimumab provides a higher ORR (43%) than nivolumab alone.[27,30] In first-line metastatic NSCLC, this regimen also provided encouraging overall survival, with an acceptable safety profile. Combination of anti-PD1 plus chemotherapy has also shown encouraging ORR and PFS with an acceptable safety profile compared to platinum doublet chemotherapy in first-line metastatic NSCLC. These data support our belief that combination of nivolumab with ipilimumab or platinum doublet chemotherapy will demonstrate superior benefit over platinum chemotherapy doublets in the neoadjuvant setting with an acceptable safety profile.

Approved v 9.0 [redacted]

In general, nivolumab is well tolerated, with a favorable safety profile relative to anticipated toxicities based on an immunostimulatory mechanism of action. Overall, the safety profile of nivolumab monotherapy as well as combination therapy is manageable and generally consistent across completed and ongoing clinical trials with no maximum tolerated dose (MTD) reached at any dose tested up to 10 mg/kg. Most AEs were low-grade (Grade 1 to 2) with relatively few related high-grade (Grade 3 to 4) AEs. There was no pattern in the incidence, severity, or causality of AEs with respect to nivolumab dose level.

The ongoing phase 3 CheckMate 227 study randomized patients with stage IV or recurrent NSCLC not previously treated in various study treatment arms by tumor PD-L1 status. Recently, in the pre-specified analysis of patients with high tumor mutational burden at a prospective cutoff of ≥ 10 mutations/MB, PFS was significantly longer in the group treated by nivolumab plus ipilimumab compared to chemotherapy, HR of PFS being 0.58 (97.5% CI 0.41, 0.81). The treatment effect was consistent and independent of PD-L1 expression, or histology. The ORR was also significantly greater for the nivolumab plus ipilimumab group (45.3% vs 26.9%) and the responses appeared more durable with median DoR not reached (95% CI 12.2, NR) compared to 5.4 months (95% CI 4.2, 6.9) for chemotherapy. In patients with TMB <10 mut/Mb, similar efficacy was seen in terms of PFS and ORR between nivolumab plus ipilimumab and chemotherapy. The safety profile was overall consistent with previously reported data with no new safety signals. In another readout focusing on patients with tumor of <1% PD-L1 expression, nivolumab plus ipilimumab showed similar efficacy to chemotherapy in terms of PFS and ORR but with much longer DoR, the safety profile was consistent with previously reported data.

In the setting of first line NSCLC with <1% tumor PD-L1 expression in the ongoing CheckMate 227 trial, PFS was improved with nivolumab plus chemotherapy vs chemotherapy (mPFS: 5.6m vs 4.7m; HR=0.74 [95% CI: 0.58 to 0.94]), mPFS of nivolumab plus ipilimumab was 4.4 months (95%CI: 3.1 to 6.0). ORR was 36.7% in nivolumab plus chemotherapy arm, 23.1% in chemotherapy arm, and 25.1% in nivolumab plus ipilimumab arm. mDOR was 7.2 months in nivolumab plus chemotherapy arm, 4.7 months in chemotherapy arm, and 18.0 months in nivolumab plus ipilimumab arm. The rate of grade 3 or 4 treatment-related adverse events was 52% with nivolumab plus chemotherapy, 35% with chemotherapy, and 25% with nivolumab plus ipilimumab, overall, for nivolumab plus chemotherapy, the safety profile and efficacy are consistent with previously reported data as well as data from other PD-(L)1 blockades in combination with chemotherapy.

As described in Section 3.2.1, two ongoing phase 2 trials with nivolumab as monotherapy and nivolumab in combination with chemotherapy showed promising anti-tumor activity in terms of depth of pathological response without adverse impact on surgical results. Nivolumab and ipilimumab as neoadjuvant therapy remain under study, with data pending.

A pattern of immune-related AEs has been defined, for which management algorithms have been developed; these are provided in Appendix 4. Most high-grade events were manageable with the use of corticosteroids or hormone replacement therapy (endocrinopathies) as instructed in these algorithms. Extensive details on the safety profile of nivolumab and ipilimumab are available in the respective Investigator Brochures and will not be repeated herein.

Approved v9.0 [redacted]

The potential benefit of nivolumab plus ipilimumab or nivolumab plus platinum doublet chemotherapy as neoadjuvant therapy in Stage I-III NSCLC are not yet known. The platinum doublet chemotherapy regimens have well described safety profiles, characterized by myelosuppression and other regimen-specific non-hematologic toxicities, such as peripheral neuropathy, nausea/vomiting, and renal impairment.

In order to assess the potential benefit of nivolumab plus platinum doublet chemotherapy over platinum doublet chemotherapy as neoadjuvant therapy in Stage I-III resectable NSCLC, this trial will randomize participants to 2 arms: nivolumab plus platinum doublet chemotherapy, or platinum doublet chemotherapy alone. Under revised protocol 02, participants already enrolled in the original 2-arm part and in the arm of nivolumab plus ipilimumab in the 3-arm part per revised protocol 02 will continue scheduled trial procedures. To assure an ongoing favorable risk/benefit assessment for participants enrolled onto CA209816, an independent Data Monitoring Committee (DMC) will be utilized to monitor the safety and activity of the treatments throughout the conduct of the trial.

4 OBJECTIVES AND ENDPOINTS

Table 4-1: Objectives and Endpoints

Objectives	Endpoints
**Primary**	
• To compare the event-free survival (EFS) by blinded independent central review (BICR) in participants receiving nivolumab plus platinum doublet chemotherapy vs participants receiving platinum doublet chemotherapy in operable stage IB (≥ 4 cm), II, or resectable IIIA (N2) NSCLC	• EFS defined as the length of time from randomization to any of the following events: any progression of disease precluding surgery, progression or recurrence disease (based on BICR assessment per RECIST 1.1) after surgery, or death due to any cause. Participants who don't undergo surgery for reason other than progression will be considered to have an event at RECIST 1.1 (based on BICR) progression or death.
• To compare the pathologic complete response (pCR) rate in participants receiving nivolumab plus platinum doublet chemotherapy vs participants receiving platinum doublet chemotherapy in operable stage IB (≥ 4 cm), II, or resectable IIIA (N2) NSCLC	• pCR rate is defined as number of randomized participants with absence of residual tumor in lung and lymph nodes as evaluated by blinded independent pathological review (BIPR), divided by the number of randomized participants for each treatment group.
**Secondary**	
• To assess the major pathologic response (MPR) rate by BIPR of participants receiving nivolumab plus platinum doublet chemotherapy vs participants receiving platinum doublet chemotherapy in operable stage IB (≥ 4 cm), II, or resectable IIIA (N2) NSCLC	• MPR rate, defined as number of randomized participants with ≤ 10% residual tumor in lung and lymph nodes as evaluated by BIPR, divided by the number of randomized participants for each treatment group. Viable tumors in situ carcinoma should not be included in MPR calculation.

Approved v1.0 [redacted]

Table 4-1: Objectives and Endpoints

Objectives	Endpoints
• To compare the OS of participants receiving nivolumab plus platinum doublet chemotherapy vs participants receiving platinum doublet chemotherapy in operable stage IB (≥ 4 cm), II, or resectable IIIA (N2) NSCLC	• OS is defined as the time between the date of randomization and the date of death. OS will be censored on the last date a participant was known to be alive.
• To assess the time to death or distant metastases (TTDM) of participants receiving nivolumab plus platinum doublet chemotherapy vs participants receiving platinum doublet chemotherapy in operable stage IB (≥ 4 cm), II, or resectable IIIA (N2) NSCLC	• TTDM is defined as the time between the date of randomization and the first date of distant metastasis or the date of death in the absence of distant metastasis. Distant metastasis is defined as any new lesion that is outside of the thorax using BICR according to RECIST 1.1. Patients who have not developed distant metastasis or died at the time of analysis will be censored on the date of their last evaluable tumor assessment.
**Exploratory**	
• To assess clinical response rate (cRR) by BICR of participants receiving nivolumab plus platinum doublet chemotherapy vs participants receiving platinum doublet chemotherapy in operable stage IB (≥ 4 cm), II, or resectable IIIA (N2) NSCLC	• cRR is defined as proportion of all randomized participants whose overall radiological response prior to definitive surgery is either a complete response or partial response per RECIST 1.1 criteria by BICR
• To assess the pCR rate, MPR rate , cRR, EFS, TTDM and OS in early-stage NSCLC participants treated with nivolumab plus platinum doublet chemotherapy compared to those treated with platinum doublet chemotherapy by PDL1 status (PD-L1 ≥ 1% , PD-L1< 1% /not evaluable/ indeterminate)	• pCR rate, MPR rate, cRR, EFS, TTDM, and OS as described above
• To assess the feasibility of surgery and rate of peri- and post-operative complications (within 90 days of surgery) in participants receiving nivolumab plus platinum doublet chemotherapy compared to participants receiving platinum doublet	• Proportion of delayed or canceled surgery, duration of surgery, length of hospital stay, surgical approach including completeness of surgery, incidence of AE/SAE associated with surgery, including pneumonitis, ARDS, re-admission to the Intensive Care Unit, atrial fibrillation or other supraventricular tachycardia (SVT) to 90 days post-surgery
• To assess the safety and tolerability of nivolumab plus platinum doublet chemotherapy compared to platinum doublet chemotherapy in early stage NSCLC	• Safety and tolerability will be measured by incidence of AE, SAE, immune-related AEs, deaths, and laboratory abnormalities
• To describe the pCR rate, MPR rate , cRR, EFS, OS, TTDM, feasibility of surgery, rate of peri- and post-operative complications (within 90 days of surgery), safety and tolerability in early-stage NSCLC participants treated with nivolumab plus ipilimumab and by PDL1 status (PD-L1 ≥ 1% , PD-L1< 1%/not evaluable/indeterminate)	• pCR rate, MPR rate, cRR, EFS, OS, TTDM, feasibility of surgery, rate of peri- and post-operative complications, safety and tolerability as described above

Approved v9.0 [redacted]

Table 4-1: Objectives and Endpoints

Objectives	Endpoints
• To assess pharmacokinetics of the nivolumab plus ipilimumab or nivolumab plus platinum doublet chemotherapy in participants with early stage NSCLC	• Pharmacokinetic endpoints (See Section 9.5)
• To assess the participant's overall health status and health utility using the 3-level version of the EQ-5D-3L visual analog scale (VAS) and utility index, respectively	• Change in EQ-5D-3L scores
• To assess Event Free survival on next line of therapy (EFS2) in early-stage NSCLC participants treated with nivolumab plus platinum doublet chemotherapy compared to those treated with platinum doublet	• Event Free survival on next line of therapy (EFS2)
• To evaluate tumor mutation burden as a potential predictive biomarker of efficacy (such as EFS and OS) of nivolumab plus platinum doublet chemotherapy and of platinum-doublet chemotherapy, using data generated from tumor and blood (germ-line control) specimens.	• Biomarker endpoints (See Section 9.8)
[redacted]	
• To explore potential predictive biomarkers of nivolumab plus platinum doublet chemotherapy efficacy (such as EFS and OS) in peripheral blood and tumor specimens [redacted]	
[redacted]	

5 STUDY DESIGN

5.1 Overall Design

This is an open-label, randomized clinical trial of up to 3 cycles of neoadjuvant nivolumab (3 mg/kg every 2 weeks) and a single dose of 1 mg/kg dose of ipilimumab, nivolumab 360mg flat dose plus platinum doublet chemotherapy (up to 3 cycles), or platinum doublet chemotherapy (up

Approved v9.0 [redacted]

to 3 cycles) as neoadjuvant treatment in participants with early stage (Stage IB [≥ 4 cm], II, and resectable IIIA [N2]) NSCLC.

The original study design (before revised protocol 02) had two arms. After signing the informed consent form and upon confirmation of the participant's eligibility, participants were randomized in an open-label fashion (1:1 ratio) to either neoadjuvant nivolumab plus ipilimumab or platinum doublet chemotherapy.

Revised protocol 02 added a new, neoadjuvant nivolumab plus platinum doublet chemotherapy arm. When the third arm had opened and as each site had received IRB/EC approval of revised protocol 02, the IRT switched to a 1:1:1 randomization at the respective site. Starting from that point on, the sites were only enrolling under revised protocol 02.

Revised protocol 03 withholds randomization into the arm of neoadjuvant nivolumab plus ipilimumab but continues randomizing eligible participants into either neoadjuvant nivolumab plus platinum doublet chemotherapy arm or platinum doublet chemotherapy arm. Participants already randomized in the original 2-arm part (neoadjuvant nivolumab plus ipilimumab vs neoadjuvant chemotherapy) and in the arm of neoadjuvant nivolumab plus ipilimumab in 3-arm part defined by revised protocol 02 will remain in trial and continue scheduled trial procedures.

Participants will be randomized between 2 arms in a 1:1 ratio to neoadjuvant nivolumab plus platinum doublet chemotherapy or platinum doublet chemotherapy. Eligible participants will be stratified by:

- PD-L1 expression (≥1% or <1%/not evaluable/indeterminate)
- Disease stage (IB/II vs IIIA)
- Gender

PD-L1 status will be determined by immunohistochemical (IHC) staining of PD-L1 protein in the submitted tumor sample and categorized as follows:

- PD-L1 positive - defined as ≥ 1% tumor cell membrane staining positive in a minimum of 100 evaluable tumor cells
- PD-L1 negative – defined as < 1% tumor cell membrane staining positive in a minimum of 100 evaluable tumor cells
- PD-L1 not evaluable/indeterminate - defined as participants with insufficient sample quantity or quality to stain for PD-L1 status or those participants in whose samples PD-L1 status could not be determined despite appropriate amounts of tissue sample. For the purpose of stratification, this category will be grouped with PD-L1 negative participants. No more than 10% of participants enrolled in this trial will be PD-L1 not evaluable/indeterminate category.

Screening begins by establishing the participant's initial eligibility and signing of the informed consent (ICF). Tumor tissue (archival [slides/block ≤ 3 month] or recent tumor biopsy) must be submitted to a third-party vendor for determination of PD-L1 status prior to randomization.

All screening assessments and procedures must be performed in accordance with Table 2-1.

Approved v 1.0 [redacted]

The Treatment Phase begins when the randomization call is made into the Interactive Response Technology (IRT). The participant will be randomly assigned to 1 of the 2 treatment arms: Arm B or Arm C. The first dose of study treatment must begin within 7 days of randomization.

- **Arm A treatment:** Participants randomized into Arm A received nivolumab 3 mg/kg IV over 30 minutes every 2 weeks for up to 3 doses (ie, 6 weeks of treatment; each cycle is 14 days). With Cycle 1 only, nivolumab was followed by a single dose ipilimumab 1 mg/kg IV over 30 minutes.

- **Arm B treatment:** Participants randomized into Arm B will receive investigator-choice platinum doublet chemotherapy in 3-week cycles up to a maximum of 3 cycles (ie, 9 weeks of treatment; each cycle is 21 days):

- Regimen 1:
  - Vinorelbine 25 mg/m^2 or 30 mg/m^2 IV (per local prescribing information) push over 10 minutes or per institutional standard on Days 1 and 8
  - Cisplatin 75 mg/m^2 IV over 120 minutes or per institutional standard on Day 1, immediately following vinorelbine
- Regimen 2:
  - Docetaxel 60 mg/m^2 or 75 mg/m^2 IV (per local prescribing information) over 60 minutes or per institutional standard on Day 1
  - Cisplatin 75 mg/m^2 IV over 120 minutes or per institutional standard on Day 1, immediately following docetaxel
- Regimen 3 (squamous histology):
  - Gemcitabine 1000 mg/m^2 or 1250 mg/m^2 (per local prescribing information) IV over 30 minutes or per institutional standard on Days 1 and 8
  - Cisplatin 75 mg/m^2 IV over 120 minutes or per institutional standard on Day 1, immediately following gemcitabine
- Regimen 4 (non-squamous histology only):
  - Pemetrexed 500 mg/m^2 IV over 10 minutes or per institutional standard on Day 1
  - Cisplatin 75 mg/m^2 IV over 120 minutes or per institutional standard on Day 1, immediately following pemetrexed
- Regimen 5:
  - Paclitaxel 175 or 200 mg/m^2 IV over 180 minutes or per institutional standard on Day 1
  - Carboplatin AUC 5 or 6 IV over 30 minutes or per institutional standard on Day 1, immediately following paclitaxel

Arm C treatment: Participants randomized into Arm C will receive nivolumab 360 mg IV plus platinum doublet chemotherapy in 3-week cycles up to a maximum of 3 cycles of chemotherapy (ie, 9 weeks of treatment; each cycle is 21 days)

Approved v9.0 [redacted]

- Non-squamous NSCLC: nivolumab at a flat dose of 360 mg as 30-minute IV infusion on Day 1, followed by pemetrexed at a dose of 500 mg/m^2 IV over 10 minutes or per institutional standard and cisplatin at a dose of 75 mg/m^2 IV over 120 minutes or per institutional standard of a 3-week treatment cycle, for up to 3 cycles.
- Squamous NSCLC: nivolumab at a dose of flat dose of 360 mg as 30 minute IV infusion on Day 1, followed by gemcitabine at a dose of 1000 mg/m^2 or 1250 mg/m^2 (per local prescribing information) for a 30 minute IV infusion or per institutional standard and cisplatin at a dose of 75 mg/m^2 as a 120-minute IV infusion or per institutional standard, of a 3-week treatment cycle for up to 3 cycles. Gemcitabine will also be administered at a dose of 1000 mg/m^2 or 1250 mg/m^2 as a 30 minute IV infusion or per institutional standard on day 8 of each 3-week treatment cycle.
- Any histology: nivolumab at a flat dose of 360 mg as 30-minute IV infusion on Day 1, followed by paclitaxel 175 or 200 mg/m^2 IV over 180 minutes or per institutional standard and carboplatin AUC 5 or 6 IV over 30 minutes or per institutional standard of a 3-week treatment cycle, for up to 3 cycles.

Within 7 days of randomization, the participants must receive the first dose of study medication. Participants may be dosed no less than 12 days between nivolumab doses in Arm A and no less than 18 days between nivolumab doses in Arm C. If a dose is delayed for any reason, participants should be dosed no later than 7 days following a planned dose on any arm. If more than 7 days delay is needed for any reason, the intended dose should be skipped. If a participant receiving chemotherapy on a Day 1 and Day 8 schedule (ie, cisplatin/gemcitabine) is unable to receive Day 1 of chemotherapy but recovers in time to receive the Day 8 dose, the Day 8 dose of chemotherapy may be administered. Doses that are skipped or missed will not be replaced. Surgery should be performed within 6 weeks after completing up to 3 cycles (last dose) of neodjuvant treatment as indicated by the institutional SOC.

- For participants who are unable to tolerate cisplatin, the reasons for intolerability should be documented. If the investigator would like to use a carboplatin-containing regimen, the investigator should discuss this with and obtain approval from the Medical Monitor prior to utilization, except for opting for carboplatin plus paclitaxel.
- Carboplatin will be administered at a dose of AUC 5 or 6 as a 30-minute IV infusion or per institutional standard, on Day 1 of each 3-week cycle.
- Carboplatin should be given following gemcitabine, docetaxel, vinorelbine, pemetrexed, or paclitaxel on Day 1 of each cycle, and the carboplatin dose will be calculated using the Calvert formula as follows:
- Carboplatin dose (mg) = Target AUC x [(CrCl (mL/min) + 25]
- Creatinine clearance (CrCl) calculation is based on the Cockroft-Gault formula) and should include the most recent serum creatinine and most recent weight. NOTE: If calculation of the

Approved v 9.0 [redacted]

CrCl by the Cockroft-Gault formula yields a result of > 125 mL/min, then a CrCl should be calculated by an alternative formula per institutional standards or capped at 125 mL/min.

- The dose of carboplatin may be capped per local standards.

All Arms

PET/CT including contrast from the base of the skull to upper thighs will be performed at baseline (within 28 days prior to randomization) and within 14 days prior to planned definitive surgery. A separate CT, with contrast, of the chest, abdomen, and all other suspected sites of disease (as well as the PET/CT) is required if the CT component of a PET/CT is not of sufficient diagnostic quality for RECIST 1.1 measurements. Subsequent radiologic assessments CT, with contrast, of the chest including adrenal glands and CT or MRI of other additional suspected/known sites of disease will be performed in accordance with Table 2-3 and Table 2-4. Tumor assessments must continue per protocol until disease recurrence/progression is confirmed by BICR per RECIST 1.1 (Appendix 5), even after the initiation of subsequent anti-cancer therapies. Exceptions are cases where disease progression does not preclude surgery, regardless of whether it has been confirmed by BICR, participants should proceed to definitive surgery and continue to get tumor assessments per Table 2-3 and Table 2-4. Tumor assessments should be done as per Table 2-3 for those who did not get definitive surgery. Pharmacokinetics assessments are described in Section 9.5, and biomarker assessments are described in Section 9.8. OS will be followed continuously every 3 months via in-person or phone contact after Post-neoadjuvant Follow-up Visit 2 or after completion of adjuvant therapy, when applicable.

Following the completion of neoadjuvant treatment, all participants who remain operative candidates will undergo definitive surgery for their NSCLC within 6 weeks after completing neoadjuvant treatment.

Prior to surgery, any treatment-related toxicity should have resolved to ≤ Grade 1 or returned to baseline (except for alopecia, fatigue, and neuropathy). Investigators should discuss residual endocrine toxicities or mild renal impairment with the Medical Monitor.

All AEs, serious adverse event (SAE), and drug-related AEs resulting in surgical delays and post-surgical complications will be collected. Peri-operative complications, including a delay in planned surgery, pneumonitis, ARDS, re-admission to the Intensive Care Unit, atrial fibrillation or other SVTs, potential immune-related toxicities, and post-operative complications will be collected. Study drug dose omission or delays due to AEs will not be replaced for either arm, and participants should proceed to surgery within the predefined timeframe after standard preoperative evaluation. Surgical complications occurring within 90 days of surgery will be documented and followed until resolution.

All AEs and SAEs will be documented for a minimum of 100 days after the last dose of neoadjuvant therapy or 90 days post-surgery, whichever is longer, and for 30 days after the last dose of adjuvant therapy in participants who receive adjuvant therapy.

Approved v9.0 [redacted]

Following definitive surgery, participants in each arm may receive up to 4 cycles of adjuvant chemotherapy with or without radiation per institutional standard at the discretion of the investigator. Investigators may choose from the following post-operative regimens:

- Regimen 1:
  - Vinorelbine 25 mg/m^2 or 30 mg/m^2 IV (per local prescribing information) push over 10 minutes or per institutional standard on Days 1 and 8
  - Cisplatin 75 mg/m^2 IV over 120 minutes or per institutional standard on Day 1, immediately following vinorelbine
- Regimen 2:
  - Docetaxel 60 mg/m^2 or 75 mg/m^2 IV (per local prescribing information) over 60 minutes or per institutional standard on Day 1
  - Cisplatin 75 mg/m^2 IV over 120 minutes or per institutional standard on Day 1, immediately following docetaxel
- Regimen 3 (squamous histology):
  - Gemcitabine 1000 mg/m^2 or 1250 mg/m^2 IV (per local prescribing information) over 30 minutes or per institutional standard on Days 1 and 8
  - Cisplatin 75 mg/m^2 IV over 120 minutes or per institutional standard on Day 1, immediately following gemcitabine
- Regimen 4 (non-squamous histology only):
  - Pemetrexed 500 mg/m^2 IV over 10 minutes or per institutional standard on Day 1
  - Cisplatin 75 mg/m^2 IV over 120 minutes or per institutional standard on Day 1, immediately following pemetrexed
- Regimen 5:
  - Paclitaxel 175 or 200 mg/m^2 IV over 180 minutes or per institutional standard on Day 1
  - Carboplatin AUC5 or 6 IV over 30 minutes or per institutional standard on Day 1, immediately following paclitaxel

For participants who are unable to tolerate cisplatin, the reasons for intolerability should be documented. If the investigator would like to use a carboplatin regimen, the investigator should discuss this with and obtain approval from the Medical Monitor prior to utilization, except for opting for carboplatin plus paclitaxel.

Postoperative chemotherapy should not commence until pre-operative, treatment-related toxicity has returned to baseline or resolved to ≤ Grade 1 (exceptions for fatigue, alopecia, and neuropathy). Investigators should discuss residual endocrine toxicities or mild renal impairment with the Medical Monitor.

Assessments during adjuvant therapy are outlined in Table 2-4.

Approved v[illegible].0 [redacted]

Post-operative radiation should be administered per institutional standard of care. As radiation may interfere with imaging interpretation, efforts should be made to schedule radiotherapy so that scheduled tumor assessments do not cross over into the radiation period.

The Survival Follow-Up Phase begins 3 months after post-neoadjuvant Follow-up Visit 2 or after completion of adjuvant therapy, when applicable. Participants will be followed every 3 months for survival. Survival follow-up visits may be performed by phone contact or office visit.

The study design schematic is presented in Figure 5.1-1.

Approved v 9.0 [redacted]

Figure 5.1-1: Study Design Schematic

NOTE: As of revised protocol 03, Arm A (nivolumab + ipilimumab) has stopped enrollment

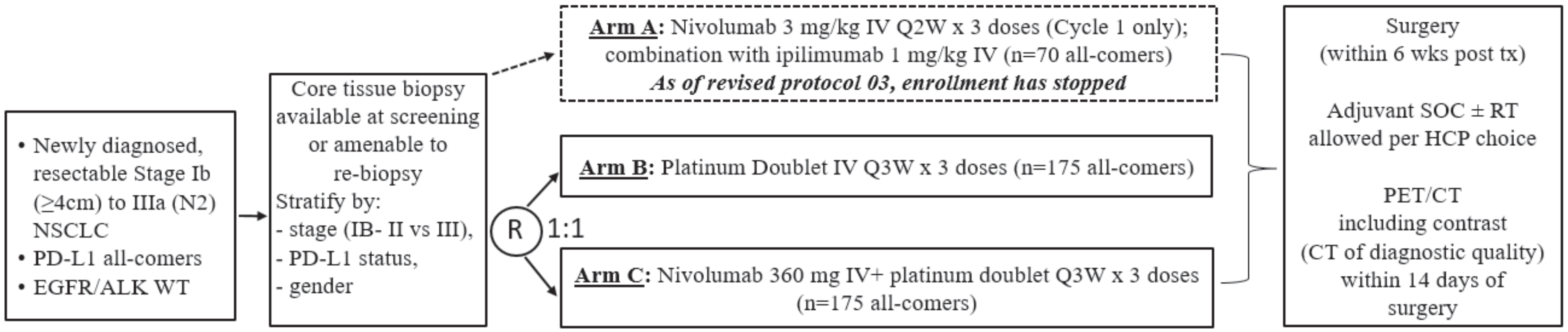

Endpoints

Primary: EFS and pCR rate in PD-L1 all-comers

Secondary: MPR, OS, and TTDM in PD-L1 all-comers

Exploratory: cRR in PD-L1 all-comers; pCR rate, EFS, MPR rate, OS, TTDM, and cRR by PD-L1 status. Safety, surgical feasibility, and rate of peri- and post-operative complications; PK, biomarkers, PROs

Post Surgical Assessments: CT /MRI Q12W for 2 yrs; then Q6 mos for 3 years, and every 52 weeks for 5 years thereafter until disease recurrence or PD. Independent review for pathological and radiologic response

Approved v9.0 [redacted]

5.1.1 Data Monitoring Committee and Other External Committees

When required, adjudicated events will be submitted to the DMC and Health Authorities for review on a specified timeframe in accordance with the adjudication documentation.

An independent Data Monitoring Committee (DMC) will be established to provide oversight of safety and efficacy considerations, study conduct, and risk-benefit ratio in protocol CA209816. An interim DMC review will occur after 15 participants have enrolled into Arm A and after 15 participants have enrolled into Arm C and have completed surgery. Following review, the DMC will recommend continuation, modification, or discontinuation of this study based on reported safety data. Representatives of the Sponsor will serve only as coordinators of the committee, without having full member responsibilities or privileges. In addition, the Sponsor will independently review safety data in a blinded manner during the conduct of this trial to ensure that any safety issues are identified and addressed. Available efficacy data will also be reviewed by the DMC during the conduct of the study. Details of the DMC responsibilities and procedures will be specified in the DMC charter.

Independent Radiology/Pathology Review

Independent pathology and radiology review will be established for central review and confirmation of endpoints. Images and tumor/lymph node samples will be submitted to these third-party vendors for central review. Sites will be trained prior to enrolling the first study participant. Images and pathology samples acquisition guidelines and submission process will be outlined in the study Imaging/Laboratory Manuals to be provided by the vendors.

Independent Pathology Review: Tumor and lymph node collection from definitive surgical resection and sampling of fresh tumor sample in RNAlater for biomarker studies (as applicable dependent on size of residual tumor) is mandatory on the day of surgery. RNAlater sample will not be collected in China. Processing of the remainder of the specimens for histopathologic analysis should be performed within 72 hours of the procedure. Sections will be used for central pathology review assessing pathologic complete response (pCR) and major pathological response (MPR). Any tumor, tumor bed, or lymph node specimens that are reviewed locally must be submitted for central pathology review. Gross examination on the entire specimen should be performed which includes all tumor, associated lymph node tissue, and uninvolved parenchyma. The specimen should be sectioned at 0.5 cm intervals, and blocks should be submitted for the full cross section for every other 0.5 cm interval. For very large tumors with no gross evidence of response, a minimum of 1 slide/cm is required for assessment of pathologic response. When estimating viable tumor, in situ carcinoma should not be included. Pathology specimen collection and processing guidelines are outlined in the CA209816 Laboratory Manual. A blinded pathology review process will be utilized to assess for confirmation of endpoints.

Independent Radiology Review: Radiologic tumor assessments will be sent to and reviewed by a Blinded Independent Central Review (BICR) from a third-party radiology vendor on an on-going basis. At the time of investigator assessed radiographic progression per RECIST 1.1, the site must request a BICR- review confirmation of progression or recurrence. However, BICR confirmation of progression should not be requested if investigator judges the progression does not preclude

surgery. Participants should proceed to definitive surgery and tumor assessments post surgery should be continued per schedule of activities. Details of the Blinded Independent Central Review responsibilities and procedures will be specified in the Blinded Independent Central Review charter.

Participants whose disease progression or recurrence is not confirmed by central review will be required to continue tumor assessments (if clinically feasible) according to the protocol-specified schedule, even after the initiation of subsequent anti-cancer therapies. Subsequent tumor assessments must be submitted to the third party radiology vendor for subsequent review and may be discontinued when the investigator and independent radiologists both assess the participant to have met RECIST 1.1 criteria for progression or recurrence.

5.2 Number of Participants

Approximately 350 participants will be randomized in a 1:1 ratio (175 participants in each arm) to the 2 arms (neoadjuvant nivolumab plus platinum doublet chemotherapy and platinum doublet chemotherapy), from 1:1:1 randomization in revised protocol 02 and 1:1 randomization in revised protocol 03.

In addition, it is expected to have around 70 participants randomized in the original 2-arm study (Arm A and Arm B) and approximately another 75 participants randomized in the arm of neoadjuvant nivolumab plus ipilimumab (Arm A) when the study had 3 arms.

5.3 End of Study Definition

The start of the trial is defined as the first participant's first visit. The analysis of pCR rate will take place after 350 participants on Arms B and C from 1:1:1 randomization defined by revised protocol 02, and 1:1 randomization by revised protocol 03 have the opportunity for surgery. After this analysis, participants will be followed for EFS and OS. Two formal interim analyses for EFS are planned after 148 events and 167 events have been observed in the participants on Arms B and C, which is projected to occur approximately 48 and 58 months after 1:1:1 randomization (the second interim analysis will take place when 167 events are observed [redacted]). These formal comparisons of EFS will allow for determination of superiority. The final analysis (FA) of EFS will be conducted after approximately 185 participants in Arms B and C have experienced an event (approximately 73 months from 1:1:1 randomization), or 4 years after the last participant's randomization. [redacted]

5.4 Scientific Rationale for Study Design

5.4.1 Rationale for Open-Label Design

This study will use an open-label design. Due to the obvious difference in chemotherapy- and immunotherapy-related toxicities, histology-dependent chemotherapy options, different dose modification rules for safety management, including different dose delay rules per arm, and different premedication requirements according to chemotherapy, an open-label design is

Approved v9.0 [redacted]

appropriate. An open-label design will also help ensure that immune-related toxicities in participant receiving immunotherapy are promptly identified and managed.

Because this study will be open-label, independent pathology and radiology review will be used for central review and confirmation of pathologic and clinical responses in all randomized participants to determine all response-related endpoints.

5.4.2 Rationale for Preoperative Systemic Therapy in NSCLC

Follow-up of adjuvant trials may require decades until a new treatment can be introduced into the early treatment setting. Preoperative chemotherapy has been assessed in a number of trials for patients with resectable NSCLC, though most trials were closed early when the adjuvant chemotherapy data revealed a survival advantage. A meta-analysis based upon 7 trials involving 988 participants suggested that neoadjuvant chemotherapy improved OS when given preoperatively (5-year survival 20% vs 14% without neoadjuvant chemotherapy). This improvement in survival is similar to that noted in the meta-analyses of predominantly adjuvant chemotherapy.[31,32]

Several studies have shown preoperative cytotoxic chemotherapy to be safe prior to surgical resection of NSCLC with no difference in extent of surgical procedures performed, operative morbidity, and mortality.[6,33,34] Immune checkpoint inhibition has the potential to provide benefits in early-stage disease. Among these benefits are the opportunity to evaluate EFS in a moderate-sized population of early-stage NSCLC patients and the potential to demonstrate long-term, disease-free status in these patients.[7,35,36,37]

5.4.3 Rationale for Immuno-oncology Treatment in Neoadjuvant NSCLC

In contrast to the adjuvant setting in which only micrometastatic disease is present, one may hypothesize that the higher tumor burden present at the time of induction treatment may be necessary for abundant antigen release and presentation to the immune system, and consequently, development of a robust immune response to immune checkpoint inhibitors.

In an ongoing feasibility trial with nivolumab monotherapy, a major pathologic response (MPR) rate was observed in 45% (9/20) of stage IB-IIIA NSCLC participants who were evaluable post-surgery after 2 cycles of nivolumab. Two patients (10%, 2/20) achieved a pathologic complete response. Major pathologic response was defined as ≤ 10% residual viable tumor at resection.[38] Responses were observed across histology and regardless of PD-L1 expression. High tumor mutation burden and neoantigen density was associated with pathological response following neoadjuvant nivolumab treatment. With a median post-op follow-up of 12 months (range of 0.8 to 19.7 months), 1 patient with MPR had a mediastinal lymph-node recurrence that was treated with concurrent chemoradiation and was free from further progression at more than 12 months of follow-up. Two patients without MPR have recurrence of lung cancer (1 solitary brain metastasis which was treated with radiotherapy and has no evidence of further recurrence at more than 16 months of follow-up; 1 systemic recurrence 1 year after surgery and died from recurrent disease 4 months later). Two additional deaths were reported including 1 disease progression in an unresected patient and 1 death not related to drug or disease).

Approved v 9.0 [redacted]

Nivolumab was well tolerated with a safety profile comparable to that observed in the phase 3 program. Administration of nivolumab in the neoadjuvant setting was deemed to be feasible with no surgical delays or post-surgical complications (within 30 days post-surgery). This trial was recently expanded to include an additional 30 participants, who will be treated with nivolumab monotherapy (n=15) and nivolumab plus ipilimumab (n=15). In addition, nivolumab monotherapy will be extended to 3 cycles. It is hypothesized that the addition of a single dose of ipilimumab to 3 doses of nivolumab and the addition of chemotherapy to nivolumab will result in the achievement of higher rate and deeper pathologic responses while maintaining an acceptable safety profile.

The 45% MPR rate from nivolumab in neoadjuvant NSCLC compares with a rate of approximately 20% from platinum doublet chemotherapy in this context. The pCR of 10% also compares favorably to the historically observed pCR rate of approximately 4% with platinum doublet chemotherapy.

In another ongoing phase 2 trial, patients with resectable stage IIIA N2 NSCLC receive 3 cycles of neoadjuvant therapy consisting of nivolumab 360mg IV plus paclitaxel/carboplatin IV Q3W then proceed to definitive surgery after which patients receive 1 year adjuvant nivolumab.[39] In the first 22 patients in whom surgical resection was performed following neoadjuvant therapy, pCR was recorded in 13 patients (60%), MPR was noted in a further 4 patients (18%). Neoadjuvant nivolumab plus paclitaxel/carboplatin was well tolerated and surgery was not delayed.

These findings, coupled with encouraging survival data from nivolumab plus ipilimumab and anti-PD1 plus chemotherapy in first-line advanced NSCLC suggest that nivolumab plus ipilimumab, especially nivolumab plus platinum doublet chemotherapy, may improve both EFS and pathologic response relative to platinum doublet chemotherapy, while providing an acceptable safety profile in the neoadjuvant treatment of NSCLC.[27]

5.4.3.1 Rationale for Combination of Nivolumab and Ipilimumab (Arm A):

Combining immunotherapeutic agents with different mechanisms of action offers the possibility of synergistic response. PD-1 and CTLA-4 are both co-inhibitory molecules, but evidence suggests that they use distinct mechanisms, to limit T-cell activation. Preliminary indirect data from peripheral T-cell assessments suggests that a given T-cell checkpoint inhibitor may modulate host immune cell phenotype rendering them more susceptible to alternate checkpoint inhibitors and thereby enhancing anti-tumor activity.

Preclinical data indicate that the combination of PD-1 and CTLA-4 receptor blockade may improve antitumor activity. In vitro combinations of nivolumab plus ipilimumab increase IFN-γ production 2- to 7-fold over either agent alone in a mixed lymphocyte reaction. Increased antitumor activity of the combination was also observed in 3 of 5 syngeneic murine cancer models. In a murine melanoma vaccine model, blockade with either CTLA-4 or PD-1 antibodies increased the proportion of CTLA-4 and PD-1-expressing CD4/CD8 tumor infiltrating T effector cells, and dual blockade increased tumor infiltration of T effector cells and decreased intratumoral T regulatory cells, as compared to either agent alone.[40]

Approved v1.0 [redacted]

The combination of nivolumab and ipilimumab was evaluated in CA209004 (MDX1106-04), a phase 1b, multiple ascending dose study in participants with treatment-naive and previously-treated advanced melanoma. Results showed promising activity with higher but tolerable toxicity than ipilimumab alone. Based on these data, CA209069, a phase 2 study, compared the combination to ipilimumab alone in treatment-naïve participants with advanced melanoma: nivolumab 1 mg/kg and ipilimumab 3 mg/kg every 3 weeks for 4 doses followed by nivolumab 3 mg/kg every 2 weeks versus ipilimumab 3 mg/kg every 3 weeks for 4 doses.[41] In participants with BRAF wild type tumors, the ORR was 61% (44/72), including 22% (16/72) CRs in the group treated with the combination, compared to 11% (4/37) with 0 CRs in those treated with ipilimumab alone. The median PFS was not reached in the combination versus 4.4 months for ipilimumab alone (HR = 0.4). Recently, a phase 3 study (CA209067, n = 945) reported significantly improved PFS and ORR with the combination of nivolumab and ipilimumab versus ipilimumab alone in previously untreated melanoma. The median PFS was 6.9 months (95% confidence interval [CI], 4.3 to 9.5) in the nivolumab group, 11.5 months (95% CI, 8.9 to 16.7) in the nivolumab plus ipilimumab group, and 2.9 months (95% CI, 2.8 to 3.4) in the ipilimumab group. Significantly longer PFS was observed in the nivolumab plus ipilimumab group than in the ipilimumab group (hazard ratio for death or disease progression, 0.42; 99.5% CI, 0.31 to 0.57; $P < 0.001$) and in the nivolumab group than in the ipilimumab group (hazard ratio, 0.57; 99.5% CI, 0.43 to 0.76; $P < 0.001$). The hazard ratio for the comparison between the nivolumab plus ipilimumab group and the nivolumab group was 0.74 (95% CI, 0.60 to 0.92).[42]

In addition, deep and durable responses were observed in previously treated, extensive stage small cell lung cancer (SCLC), with a response rate of 31.1% with the combination of nivolumab and ipilimumab.[43]

Based on the initial data in melanoma and the activity observed with nivolumab and ipilimumab in lung cancer, the nivolumab plus ipilimumab combination has been also evaluated as first-line therapy in participants with advanced NSCLC. In CA209012, early combination cohorts evaluated 2 dosing schedules that were studied in the CA209004 study in melanoma.[44]

- Nivolumab 1 mg/kg + ipilimumab 3 mg/kg, every 3 weeks for 4 doses, followed by nivolumab 3 mg/kg q 2 weeks (Arms G and H, n=24);
- Nivolumab 3 mg/kg + ipilimumab 1 mg/kg, every 3 weeks for 4 doses, followed by nivolumab 3 mg/kg q 2 weeks (Arms I and J, n=25)

These regimens resulted in significant toxicity, with 39% of participants discontinuing treatment due to a treatment-related adverse event. Thus, additional combination cohorts were initiated (Arms N, O, P, Q), using lower doses of both nivolumab and ipilimumab, or the approved dose of nivolumab with less frequent dosing of ipilimumab. These new regimens were better tolerated, and the safety data are not dissimilar to what has been observed in the nivolumab monotherapy cohort (Arm F in CA209012) (Table 5.4.3.1-1).

Approved v9.0 [redacted]

Table 5.4.3.1-1: Treatment-related Adverse Events from Selected Cohorts in CA209012

Arm[a]	No. Participants/ arm	Follow-up time (median, wks)	No. Participants still on treatment	No. Participants with drug-related AEs	No. Participants with grade 3-4 drug-related AEs	No. participants d/c due to drug-related AEs (all grades)
N[b]	31	72	6 (19%)	24 (77%)	9 (29%)	4 (13%)
O[b]	40	27	14 (35%)	29 (73%)	14 (35%)	3 (8%)
P[b]	38	37	20 (53%)	28 (74%)	11 (29%)	2 (5%)
Q[b]	39	34	15 (39%)	27 (69%)	11 (28%)	4 (10%)
F[c]	52	62	5 (10%)	37 (71%)	10 (19%)	5 (10%)

[a] N:nivolumab 1 mg/kg plus ipilimumab 1 mg/kg every 3 weeks x 4, followed by nivolumab 3 mg/kg every 2 weeks; O: nivolumab 1 mg/kg every 2 weeks plus ipilimumab 1 mg/kg every 6 weeks; P: nivolumab 3 mg/kg every 2 weeks plus ipilimumab every 12 weeks; Q: nivolumab 3 mg/kg every 2 weeks plus ipilimumab 1 mg/kg every 6 weeks; F: nivolumab 3 mg/kg every 2 weeks

[b] based on August 2015 database lock

[c] based on March 2015 database lock

Recently, the pooled results from the CA209012 (Checkmate 012) nivolumab 3 mg/kg Q2W and ipilimumab 1 mg/kg Q12W and Q6W cohorts showed continued clinical benefit (OS and PFS) with nivolumab plus ipilimumab in all patients and those with ≥ 1% and ≥ 50% tumor PD-L1 expression.[45] ORR was 43% in all treated patients and 57% and 92% in patients with ≥ 1% and ≥ 50% tumor PD-L1 expression, respectively, consistent with the previous report. Additionally, 6 investigator-assessed complete responses ([CRs] 8%; including 3 pathologic responses) were achieved in the Q12W/Q6W cohorts.[46] Median duration of response had not yet been reached in the overall population or in subgroups with <1%, ≥ 1%, or ≥ 50% PD-L1 expression; duration of response ranged from 1.4+ to 27.9+ months. All treated patients (n = 77) showed a 1 year OS of 76% and 2 year OS of 49%. When analyzed by PD-L1 expression, patients with ≥ 1% PD-L1 (n = 47) had a 1 years OS of 87% and 2 year OS of 58%, which increased to 100% and 62%, respectively for ≥ 50% PD-L1 patients. Nivolumab plus ipilimumab remained tolerable, as most treatment-related AEs were manageable and no new safety concerns were identified in follow-up.

As described in Section 3.2.1, ongoing phase 3 CheckMate 227 study randomized patients with stage IV or recurrent NSCLC that was not previously treated in various study treatment arms by tumor PD-L1 status. Recently, in the pre-specified analysis of patients with high tumor mutational burden at a prospective cutoff of ≥ 10 mutations/MB, progression-free survival was significantly longer in the group treated by nivolumab plus ipilimumab compared to chemotherapy, HR of PFS being 0.58 (97.5% CI 0.41, 0.81). The treatment effect was consistent and independent of PD-L1

Approved v[illegible].0 [redacted]

expression or histology. The ORR was also significantly greater for the nivolumab plus ipilimumab group (45.3% vs 26.9%) and the responses appeared more durable with median DoR not reached (95% CI 12.2, NR) compared to 5.4 months (95% CI 4.2, 6.9) for chemotherapy. In patients with TMB < 10 mut/Mb, similar efficacy was seen in terms of PFS and ORR between nivolumab plus ipilimumab and chemotherapy. The safety profile was overall consistent with previously reported data with no new safety signals. In another readout focusing on patients with tumor of <1% PD-L1 expression, nivolumab plus ipilimumab showed similar efficacy to chemotherapy in terms of PFS and ORR but with much longer DoR, the safety profile was consistent with previously reported data.

5.4.3.2 Rationale for PD-1 Inhibitor with Chemotherapy (Arm C)

The interaction of a tumor with the immune system is complex. Tumors and the tumor microenvironment are known to express a variety of factors that impede a robust immune response from eliminating the tumor. Soluble and membrane-bound factors have been shown to inhibit the cytolytic activity of tumor infiltrating T-cells (eg, PD-L1 expression; TGF-beta). In addition, some tumor-derived factors are able to enhance the immune system counter-regulatory systems (eg, increased T-regulatory cells). Finally, suboptimal tumor antigen delivery and presentation has been postulated as another mechanism by which tumors can successfully evade immune system recognition.

Cancer therapeutics such as chemotherapy may modulate tumor/immune-system interactions in favor of the immune system. Chemotherapy can result in tumor cell death with a resultant increase in tumor antigen delivery to antigen-presenting cells. Tumor cell death may also lead to a reduction in soluble and membrane-bound factors inhibiting tumor-infiltrating T-cells. Chemotherapy may also disrupt immune system regulatory networks by decreasing numbers of T-regulatory cells.

Nivolumab added to chemotherapy has been evaluated in several cohorts of chemotherapy-naive subjects with advanced NSCLC in study CA209012. Nivolumab 10 mg/kg was combined with gemcitabine and cisplatin and pemetrexed + cisplatin. Nivolumab 10 mg/kg and 5 mg/kg was combined with paclitaxel and carboplatin.

The safety profile of nivolumab plus platinum-doublet chemotherapy reflects additive toxicities of the individual agents, which were manageable using established safety guidelines. No dose-limiting toxicities were observed during first 6 weeks of treatment. The frequency of most immune-related select AEs was higher for the combination than what has been observed for nivolumab monotherapy. However, these treatment-related AEs, including pneumonitis, were effectively managed and did not lead to any deaths. Pneumonitis of any grade was reported in 7 subjects (13%): Grade 3-4 in 4 subjects (7%). Twelve (21%) subjects discontinued due to treatment-related AEs (Table 5.4.3.2-1).

The overall response rate across all the nivolumab and chemotherapy cohorts ranged from 33-47% and median duration of response was 27.3 weeks. In the 15 participants that received nivolumab 10 mg/kg plus pemetrexed and cisplatin, 47% achieved a PR or CR. In the 12 participants that received nivolumab 10 mg/kg plus gemcitabine and cisplatin, 33% achieved a CR or PR. The 1-year survival rate was 87% (Table 5.4.3.2-2).

Approved v 9.0 [redacted]

Activity was evaluated by PD-L1 expression and was observed in subjects with both PD-L1 expressing and non-expressing tumors. Overall, 79% (44/56) of subjects had evaluable tumor samples. At the ≥ 1% expression level, the response rate was 48% and 43% for expressers and non-expressers, respectively. The 1-year OS was 70% and 76% for expressers and non-expressers, respectively.

Table 5.4.3.2-1: Safety Evaluation in CA209012

	Total (N=56)		
	**All Grades**	**Grade 3**	**Grade 4**
Subjects with any treatment-related AE, % (n)	95 (53)	41 (23)	4 (2)[a]
Treatment-related AE in >15% of Patients, % (n)			
Fatigue	71(40)	5 (3)	0
Nausea	46 (26)	2 (1)	0
Decrease Appetite	36 (20)	2 (1)	0
Alopecia	30 (17)	0	0
Anemia	27 (15)	4 (2)	0
Rash	27 (15)	2 (1)	0
Arthralgia	21 (12)	0	0
Diarrhea	21 (12)	2 (1)	0
Constipation	20 (11)	0	0
Peripheral Neuropathy	20 (11)	0	0

[a] Grade 4 events: neutrophil count decreased (n = 1), pneumonitis and neutropenia (n = 1 each; occurred in the same patient).

Approved v 9.0 [redacted]

Table 5.4.3.2-2: Efficacy of First-Line Treatment of Nivolumab/Chemotherapy Combination in CA209012

Efficacy of First-Line Treatment of Nivolumab/Chemotherapy Combination in CA209012				
	Nivolumab 10 mg/kg			Nivolumab 5 mg/kg
	Gem/Cis (n=12)	Pem/Cis (n=15)	Pac/Carb (n=15)	Pac/Carb (n=14)
ORR, %	33	47	47	43
SD, %	58	47	27	43
Median Duration of Response, Weeks	45	24.4	27.3	27.3
12-mo OS rate, %	50	87	72	86
18-mo OS Rate, %	33	60	40	62
Median OS, Weeks	51	83	65	Not Reached

As described in Section 3.2.1, in the ongoing CheckMate 227 trial, in the setting of first line NSCLC with <1% tumor PD-L1 expression, PFS was improved with nivolumab plus chemotherapy vs chemotherapy (mPFS: 5.6m vs 4.7m; HR=0.74 [95% CI: 0.58 to 0.94]), mPFS of nivolumab plus ipilimumab was 4.4 months (95%CI: 3.1 to 6.0). ORR was 36.7% in nivolumab plus chemotherapy arm, 23.1% in chemotherapy arm, and 25.1% in nivolumab plus ipilimumab arm. mDOR was 7.2 months in nivolumab plus chemotherapy arm, 4.7 months in chemotherapy arm, and 18.0 months in nivolumab plus ipilimumab arm. The rate of grade 3 or 4 treatment-related adverse events was 52% with nivolumab plus chemotherapy, 35% with chemotherapy, and 25% with nivolumab plus ipilimumab, overall, for nivolumab plus chemotherapy, the safety profile and efficacy are consistent with previously reported data as well as data from other PD-(L)1 blockades in combination with chemotherapy.

5.4.4 Rationale for Pathologic Response

Pathologic complete response (pCR) has been long known to correlate with survival after tumor resection. Recently, the FDA approved pertuzumab based on pathologic response rate for the treatment of locally advanced breast cancer. In the ongoing feasibility trial with nivolumab monotherapy, 2 out of 20 (10%) patients achieved pCR. In another ongoing phase II trial (NADIM) to evaluate neoadjuvant nivolumab plus chemotherapy, 13 out of 22 (60%) patients achieved pCR. These compare favorably to the approximate 4% pCR in NSCLC observed historically with neoadjuvant chemotherapy. It is predicted that patients treated with the combination of nivolumab plus ipilimumab, especially nivolumab plus platinum doublet chemotherapy, will achieve a higher pCR rate than in patients treated with nivolumab monotherapy. Therefore, pCR will be one of primary objectives.

In a recent publication by Hellmann and colleagues, major pathologic response (MPR), defined as ≤ 10% viable tumor, was proposed as a better surrogate for survival and is observed in

Approved v9.0 [redacted]

approximately 22% of NSCLC patients treated with neoadjuvant chemotherapy.[47] This proposed surrogate for survival was supported by the data from a prospective study and retrospective study.[48, 49] Pataer and colleagues used a score system that quantifies the percentage of viable tumor cells and demonstrated that there is a statistically significant correlation between higher percentage of viable cells and shorter disease-free and OS in patients who received induction treatment. This correlation was not evident in patients treated with upfront surgery alone. The 5-year recurrence-free survival for patients with and without a pathologic response were 78% and 35%, respectively (P < 0.001). The 5-year overall survival for patients with and without a pathologic response were 85% and 40%, respectively (P < 0.0001). In the on-going feasibility CA209159 trial with nivolumab monotherapy, a MPR rate of 45% (9/20) was observed in stage IB-IIIA NSCLC participants who were evaluable post-surgery after 2 cycles of nivolumab. In NADIM, further to 60% pCR rate, MPR was also noted in 4 additional patients (18%). These results compare favorably to the MPR rate of approximately 22% observed with historical neoadjuvant chemotherapy. These results further support the exploration of MPR as a secondary objective for the CA209816 trial.

5.4.5 Rationale for Patient Population

The OS benefit of several adjuvant therapies in this population is presented in Table 3.1-1. The choice of stage II and resectable IIIA (N2) NSCLC was made because these participants have a high risk of tumor relapse and death with current standard therapy, including surgery with preoperative or postoperative chemotherapy. There is an urgent need for improved, novel therapies for this group of participants. Participants with stage IB NSCLC with primary tumors of ≥ 4 cm diameter have been included because these participants are also at a high risk of tumor relapse and may be considered candidates for standard adjuvant chemotherapy.[2,50] It is anticipated that participants enrolled on this study may require adjuvant chemotherapy with or without radiation, and the administration of adjuvant chemotherapy will commence if considered clinically indicated in the postoperative period.

5.4.6 Rationale for Choices of Platinum-based Chemotherapy Doublet

The regimens selected for use in this study are commonly used adjuvant and neoadjuvant therapies in the clinical setting. A significant difference in outcome was not seen between participants using the regimens proposed in this trial.[51] Cisplatin-based therapy has been shown to significantly improve survival.[52] Several clinical trials have found a significant improvement in OS when using platinum-based chemotherapy regimens.[53,54,55,56,57] In revised protocol 03, paclitaxel/carboplatin is added among available chemotherapy options based on the promising data of using this doublet as backbone to combine with nivolumab in neoadjuvant therapy. Besides, meta-analysis of individual participant data from neoadjuvant trials did not identify evidence of a difference in effect of chemotherapy by whether regimens were cisplatin or carboplatin-based (interaction p=0.48).[58]

Approved v9.0 [redacted]

5.5 Justification for Dose

5.5.1 Rationale for Shorter Infusion Times for Nivolumab

Nivolumab has been administered safely over 60 minutes at doses ranging up to 10 mg/kg safely over long treatment duration. In Study CA209010 (a phase 2, randomized, double-blinded, dose-ranging study of nivolumab in participants with advanced/metastatic clear cell RCC), a dose association was observed for infusion site reactions and hypersensitivity reactions (1.7% at 0.3 mg/kg, 3.7% at 2 mg/kg and 18.5% at 10 mg/kg). All the events were Grade 1-2 and were manageable. An infusion duration of 30 minutes for 3 mg/kg of nivolumab and for a flat dose of 360 mg (30% of the dose provided at 10 mg/kg and 45% of dose provided at 10 mg/kg assuming an average body weight of 80 kg, respectively) is not expected to present any safety concerns compared to the prior experience at 10 mg/kg nivolumab dose infused over a 60-minute duration.

Of note, CA209153, a phase 3b/4 safety study of nivolumab in participants with metastatic NSCLC who have progressed during or after at least 1 prior systemic regimen, used a 30-minute infusion in a cohort of participants with no safety issues.

Overall, infusion reactions including high-grade hypersensitivity reactions have been uncommon across nivolumab. Overall, a change in safety profile is not anticipated with 30-minute infusion of nivolumab.

5.5.2 Rationale for Nivolumab 360 mg Flat Dose

Nivolumab monotherapy has been extensively studied in NSCLC patient population in studies CA209003, CA209063, CA209017, and CA209057 with body weight normalized dosing (mg/kg). Nivolumab pharmacokinetics (PK) and exposures of subjects in these studies have been characterized by population pharmacokinetic (PPK) analysis of data collected from these studies, together with PK data from several Phase 1, 2, and 3 clinical studies of nivolumab monotherapy in solid tumors. Nivolumab PK was determined to be linear, with dose proportional exposures over a dose range of 0.1 to 10 mg/kg. Nivolumab clearance and volume of distribution was found to increase with increasing body weight, but the increase was less than proportional, indicating that a mg/kg dose represents an over-adjustment for the effect of body weight on nivolumab PK.

Flat dosing offers several advantages over body weight normalized dosing, including reduced potential for dosing errors and shortened dosage preparation time. A flat dose of 360 mg every 3 weeks is expected to produce the equivalent average exposure to 3 mg/kg every 2 weeks at the median body weight of ~80 kg in nivolumab-treated subjects.

A PPK model predicted overall nivolumab exposures across subjects with a wide range of body weight (35-160 kg) for a 360 mg every 3 weeks flat dose to be similar to that from 3 mg/kg every 2 weeks. Although the flat dose is expected to lead to higher exposure in lighter patients, relative to the exposure in heavier patients given the relationship between nivolumab PK and body weight, the predicted median and 95th percentile of exposures from these regimens are maintained well below those in 10 mg/kg every 2 weeks, which was established as a safe and well-tolerable dose.

In addition, data from the Japanese Phase 1 study ONO-4538-01 did not demonstrate dose-limiting toxicity at nivolumab up to 20 mg/kg every 2 weeks in Japanese patients and showed similarity in

Approved v 9.0 [redacted]

PK properties between Global and Japanese population. Therefore, the proposed 360 mg flat dose is expected to be safe and tolerable in an Asian population.

Nivolumab 5 or 10 mg every 3 weeks plus platinum-based chemotherapy was evaluated in CA209012 and deemed to be tolerable. In addition, nivolumab 360 mg every 3 weeks plus platinum based chemotherapy is further being evaluated on several global randomized phase 3 trials including CA209227 and CA209722.

6 STUDY POPULATION

For entry into the study, the following criteria MUST be met.

6.1 Inclusion Criteria

1) **Signed Written Informed Consent**
   a) Participants must have signed and dated an IRB/IEC approved written informed consent form in accordance with regulatory and institutional guidelines. This must be obtained before the performance of any protocol related procedures that are not part of normal participant care.
   b) Participants must be willing and able to comply with scheduled visits, treatment schedule, and laboratory testing.
2) **Type of Participant and Target Disease Characteristics**
   a) Eastern Cooperative Group (ECOG) Performance Status 0-1 (Appendix 3)
   b) Participants with histologically confirmed Stage IB (≥ 4 cm), II, IIIA (N2) NSCLC (per the 7th International Association for the Study of Lung Cancer) with disease that is considered resectable.[59]
   c) Measurable disease according to RECIST version 1.1
   d) Participants must have a tumor tissue sample available for PD-L1 IHC testing performed by a third-party analyzing lab during the screening period:
      i) Either a formalin-fixed, paraffin-embedded (FFPE) tissue block or unstained tumor tissue sections, with an associated pathology report, must be submitted for biomarker evaluation prior to randomization. The tumor tissue sample may be fresh or archival if obtained within 3 months prior to enrollment.
      ii) Tissue must be a core needle biopsy, excisional or incisional biopsy. Fine needle biopsies obtained by EBUS is not considered adequate for biomarker review and randomization. Core needle biopsies obtained by EBUS are acceptable for randomization.
   e) Absence of major associated pathologies that increase the surgery risk to an unacceptable level
   f) All suspicious mediastinal lymph nodes including those that are pathologically enlarged or FDG avid on PET/CT require further sampling for pathological confirmation if accessible by mediastinoscopy, thoracoscopy, or EBUS.
   g) Pulmonary function capacity (eg, FVC, FEV1, TLC, FRC, and DLco) capable of tolerating the proposed lung resection according to the surgeon.

Approved v9.0 [redacted]

3) Age and Reproductive Status

a) Males and Females, ages ≥18 or age of majority

b) Women of childbearing potential (WOCBP) must have a negative serum or urine pregnancy test (minimum sensitivity 25 IU/L or equivalent units of HCG) within 24 hours prior to the start of study treatment.

c) Women must not be breastfeeding

d) Women of childbearing potential (WOCBP) must agree to follow instructions for method(s) of contraception (Appendix 6) for the duration of treatment with nivolumab and 5 months after the last dose of study treatment (ie, 30 days [duration of ovulatory cycle]) plus the time required for the investigational drug to undergo approximately 5 half-lives (for participants treated in Arm A and Arm C).

e) WOCBP must also agree to follow instructions for method(s) of contraception from the time of enrollment for the duration of treatment with chemotherapy plus 5 half-lives of chemotherapy plus 30 days (duration of ovulatory cycle) for a total of 30 days post-treatment completion or a duration specified by the local labels of the chemotherapy drugs received, whichever is longer (for participants treated in Arm B and Arm C).

f) Males who are sexually active with WOCBP must agree to follow instructions for method(s) of contraception (Appendix 6) for the duration of treatment with nivolumab and 7 months after the last dose of study treatment (ie, 90 days [duration of sperm turnover] plus the time required for the investigational drug to undergo approximately 5 half-lives) (for participants treated in Arm A and Arm C). In addition, male participants must be willing to refrain from sperm donation during this time.

g) Males who are sexually active with WOCBP must agree to follow instructions for method(s) of contraception for the duration of treatment with study treatment(s) with chemotherapy plus 5 half-lives of the study treatment plus 90 days (duration of sperm turnover) for a total of 90 days post-treatment completion or a duration specified by the local labels of the chemotherapy drugs received, whichever is longer (participants in Arm B and Arm C). In addition, male participants must be willing to refrain from sperm donation during this time.

h) Azoospermic males are exempt from contraceptive requirements. WOCBP who are continuously not heterosexually active are also exempt from contraceptive requirements, and still must undergo pregnancy testing as described in this section.

Investigators shall counsel WOCBP, and male participants who are sexually active with WOCBP, on the importance of pregnancy prevention and the implications of an unexpected pregnancy. Investigators shall advise on the use of highly effective methods of contraception (Appendix 6), which have a failure rate of < 1% when used consistently and correctly.

Approved v9.0 [redacted]

6.2 Exclusion Criteria

1) Medical Conditions

a) Presence of locally advanced unresectable (regardless of stage) or metastatic disease (stage IV).

b) Participants with known EGFR mutations or ALK translocation. If testing is done, an FDA-approved assay should be used, and testing will be performed locally.

c) Participants with brain metastases are excluded from this study, and all participants with stage II or higher disease and those with suspicion of brain metastases should have MRI or CT of the brain with pre- and post-contrast within 28 days prior to randomization.

d) Participants with ≥ Grade 2 peripheral neuropathy

e) Participants with an active, known or suspected autoimmune disease. Participants with type I diabetes mellitus, hypothyroidism only requiring hormone replacement, skin disorders (such as vitiligo, psoriasis, or alopecia) not requiring systemic treatment, or conditions not expected to recur in the absence of an external trigger are permitted to enroll.

f) Participants with a condition requiring systemic treatment with either corticosteroids (> 10 mg daily prednisone equivalent) or other immunosuppressive medications within 14 days of randomization. Inhaled or topical steroids, and adrenal replacement steroid doses > 10 mg daily prednisone equivalent, are permitted in the absence of active autoimmune disease

g) Known history of positive test for human immunodeficiency virus (HIV) or known acquired immunodeficiency syndrome (AIDS). NOTE: Testing for HIV must be performed at sites where mandated locally.

h) Participants with serious or uncontrolled medical disorders

i) Participants with large-cell neuroendocrine carcinoma tumor histology

2) Prior/Concomitant Therapy

a) Prior administration of chemotherapy or any other cancer therapy for early stage NSCLC.

b) Prior therapy with an anti-PD-1, anti-PD-L1, anti-PDL-2, or anti-CTLA-4 antibody or any other antibody targeting T cell co-regulatory pathways.

3) Physical and Laboratory Test Findings

a) Screening laboratory values must meet the following criteria (using CTCAE v4):

i) WBC < 2000/μL

ii) Neutrophils < 1500/μL

iii) Platelets < $100x10^3$/μL

iv) Hemoglobin < 9.0 g/dL

v) Serum creatinine > 1.5 x ULN or calculated creatinine clearance (CrCl) < 50 mL/min (using the Cockcroft-Gault formula)

$$\text{Female CrCl} = \frac{\text{(140- age in years) x weight in kg x 0.85}}{\text{72 x serum creatinine in mg/ dL}}$$

$$\text{Male CrCl} = \frac{\text{(140- age in years) x weight in kg x 1.00}}{\text{72 x serum creatinine in mg/ dL}}$$

vi) AST > 3.0 x ULN

Approved v 9.0 [redacted]

vii) ALT > 3.0 x ULN

viii) Total Bilirubin > 1.5 x ULN (except participants with Gilbert Syndrome who must have a total bilirubin level of < 3.0 x ULN).

b) Participants with active hepatitis B (positive hepatitis B surface antigen [HBsAg] or hepatitis C virus (HCV) (positive HCV RNA)

i) Participants with past HBV infection or resolved HBV infection (defined as the presence of hepatitis B core antibody [HBcAb] and the absence of HBsAg) are eligible. HBV DNA must be obtained in these patients prior to randomization. HBV carriers or those participants requiring antiviral therapy are not eligible to participate.

ii) Participants positive for HCV antibody are eligible only if PCR is negative for HCV RNA.

c) Prior malignancy active within the previous 3 years except for locally curable cancers that have been apparently cured, such as basal or squamous cell skin cancer, superficial bladder cancer, or carcinoma in situ of the prostate, cervix, or breast.

d) Participants with serious or uncontrolled medical disorders

4) **Allergies and Adverse Drug Reaction**

a) History of allergy or hypersensitivity to study drug components

5) **Other Exclusion Criteria**

a) Prisoners or participants who are involuntarily incarcerated. (Note: under certain specific circumstances a person who has been imprisoned may be included or permitted to continue as a participant. Strict conditions apply and Bristol-Myers Squibb Company approval is required.

b) Participants who are compulsorily detained for treatment of either a psychiatric or physical (eg, infectious disease) illness

Eligibility criteria for this study have been carefully considered to ensure the safety of the study participants and that the results of the study can be used. It is imperative that participants fully meet all eligibility criteria.

6.3 Lifestyle Restrictions

Not applicable. No restrictions are required.

6.4 Screen Failures

Screen failures are defined as participants who consent to participate in the clinical study but are not subsequently randomized. A minimal set of screen failure information is required to ensure transparent reporting of screen failure participants, to meet the Consolidated Standards of Reporting Trials (CONSORT) publishing requirements, and to respond to queries from regulatory authorities. Minimal information includes date of consent, demography, screen failure details, eligibility criteria, and any serious AEs.

Approved v1.0 [redacted]

6.4.1 Retesting During Screening or Lead-In Period

Participant Re-enrollment: This study permits the re-enrollment of a participant who has discontinued the study as a pre-treatment failure (ie, participant has not been randomized / has not been treated). If re-enrolled, the participant must be re-consented. Participants are allowed to re-enroll twice. Eligibility will need to be confirmed if a participant is re-enrolled.

Retesting of laboratory parameters and/or other assessments within any single Screening or Lead-in period will be permitted up to 3 times (in addition to any parameters that require a confirmatory value).

The most current result prior to randomization is the value by which study inclusion will be assessed, as it represents the participant's most current, clinical state.

7 TREATMENT

Study treatment is defined as any investigational treatment(s), marketed product(s), placebo or medical device intended to be administered to a study participant according to the study randomization or treatment allocation

Study treatment includes both Investigational [Medicinal] Product (IP/IMP) and Non-investigational [Medicinal] Product (Non-IP/Non-IMP) and can consist of the following:

All products, active or placebo, being tested or used as a comparator in a clinical trial.

An investigational product, also known as investigational medicinal product in some regions, is defined a pharmaceutical form of an active substance or placebo being tested or used as a reference in a clinical study, including products already with a marketing authorization but used or assembled (formulated or packaged) differently than the authorized form, or used for an unauthorized indication, or when used to gain further information about the authorized form.

Other medications used as support or escape medication for preventative, diagnostic, or therapeutic reasons, as components of the standard of care for a given diagnosis, may be considered as non-investigational products.

Approved v 9.0 [redacted]

Table 7-1: Study treatments for CA209816

Product Description / Class and Dosage Form	Potency	IP/ Non-IMP	Blinded or Open Label	Packaging / Appearance	Storage Conditions (per label)
BMS-936558-01 Solution for Injection/Nivolumab[a]	10 mg/mL	IP	Open-label	5 or 10 (100 mg) vials per carton; Clear to opalescent, colorless to pale yellow liquid. Few particulates may be present.	2 to 8° C. Protect from light and freezing
Ipilimumab Solution for Injection	5 mg/mL	IP	Open-label	4 vials/carton. Clear to opalescent, colorless to pale yellow liquid. May contain particles	2 to 8° C. Protect from light and freezing
Vinorelbine NC Concentrate for Solution for Infusion[b]	10 mg/mL	IP	Open-label	1 vial/carton. Clear, colorless to pale yellow solution.	Product should be stored as per market product conditions.
Gemcitabine Concentrate for Solution for Infusion[b]	1000 mg/vial (38 mg/mL)	IP	Open-label	1 vial/carton. Clear, colorless or light straw-colored solution	Product should be stored as per market product conditions.
Gemcitabine Powder for Solution for Infusion[b]	1000 mg/vial	IP	Open label	1 vial per carton. White to off-white plug or powder	Product should be stored as per market product conditions.
Docetaxel Concentrate for Solution for Infusion[b]	10 mg/mL	IP	Open-label	1 vial/carton. Pale yellow to brownish yellow solution	Product should be stored as per market product conditions.
Pemetrexed Powder for Concentrate for Solution for Infusion[b]	500 mg/vial	IP	Open-label	1 vial/carton. White to either light yellow or green-yellow lyophilised powder	Product should be stored as per market product conditions.
Cisplatin Concentrate for Solution for Infusion[b]	100 mg/vial (1 mg/mL)	IP	Open-label	4 vials per carton. Clear, colorless solution	Product should be stored as per market product conditions.

Approved v9.0 [redacted]

Table 7-1: Study treatments for CA209816

Product Description / Class and Dosage Form	Potency	IP/ Non-IMP	Blinded or Open Label	Packaging / Appearance	Storage Conditions (per label)
Carboplatin Solution for Injection[b]	450 mg/vial (10 mg/mL)	IP	Open-label	4 vials/carton. Clear, colorless or slightly yellow solution.	Product should be stored as per market product conditions.
Paclitaxel Solution for Injection[b]	100mg/vial (6mg/mL)	IP	Open-label	4 vials/carton. Clear, colorless or slightly yellow viscous solution.	Product should be stored as per market product conditions.

[a] May be labeled as either "BMS-936558-01" or "nivolumab"

[b] These products may be obtained as local commercial product in certain countries if allowed by local regulations. In these cases, products may be a different pack size/potency than listed in the table. These products should be prepared, stored, stored and administered with the Package Insert or Summary of Product Characteristics.

Note: As of revised protocol 03, enrollment in nivolumab + ipilimumab arm has stopped enrollment.

Approved v1.0 [redacted]

7.1 Treatments Administered

The selection and timing of dose for each participant is presented in Table 7.1-1.

Table 7.1-1: Selection and Timing of Dose

Study Treatment	Unit dose strength(s)/ Dosage level(s)	Dosage formulation Frequency of Administration	Route of Administration
BMS-936558-01 Nivolumab	10 mg/mL	3 mg/kg every 2 weeks for up to 3 cycles (Arm A)<br>360 mg every 3 weeks for up to 3 cycles (Arm C)	IV
Ipilimumab	5 mg/mL	1 mg/kg at Cycle 1 only	IV
Vinorelbine[60]	10 mg/mL	25 mg/m^2 or 30 mg/m^2 on Days 1 and 8 of a 3-week cycle for up to 3 cycles[a]	IV
Gemcitabine[61]	38 mg/mL	1000 mg/m^2 or 1250 mg/m^2 on Days 1 and 8 of a 3-week cycle for up to 3 cycles[a]	IV
Docetaxel[62]	10 mg/mL	60 mg/m^2 or 75 mg/m^2 on Day 1 of a 3-week cycle for up to 3 cycles[a]	IV
Pemetrexed[63]	500 mg/vial	500 mg/m^2 on Day 1 of a 3-week cycle for up to 3 cycles[a]	IV
Cisplatin[64]	1 mg/mL	75 mg/m^2 on Day 1 of a 3-week cycle for up to 3 cycles[a]	IV
Carboplatin[65]	10 mg/mL	AUC 5 or 6 on Day 1 of a 3-week cycle for up to 3 cycles[a,b]	IV.
Paclitaxel (Taxol PI TBC)[66,67]	6 mg/mL	175 or 200 mg/m^2 on Day 1 of a 3-week cycle for up to 3 cycles[a]	IV

a Following definitive surgery, participants may receive up to 4 cycles of adjuvant chemotherapy with or without radiation at the discretion of the investigator.

b Carboplatin is initiated at a dose of AUC 5 or 6 when combined with paclitaxel.

Note: As of revised protocol 03, enrollment in nivolumab + ipilimumab arm has stopped enrollment.

Nivolumab and Ipilimumab (Arm A)

NOTE: As of revised protocol 03, enrollment has stopped in Arm A.

Participants are to receive nivolumab at a dose of 3 mg/kg as a 30-minute infusion on Day 1 of each treatment cycle every 2 weeks for a maximum of 3 doses. In Cycle 1, participants will also receive a single dose of ipilimumab 1 mg/kg as a 30-minute infusion on Day 1. Participants are to begin study treatment within 7 calendar days of randomization.

Dosing calculations should be based on the body weight assessed at baseline. It is not necessary to re-calculate subsequent doses if the participant weight is within 10% of the weight used to calculate the previous dose. All doses should be rounded up or to the nearest milligram or per institutional standard.

When study drugs (nivolumab and ipilimumab) are to be administered on the same day, nivolumab is to be administered first. Nivolumab infusion must be promptly followed by a saline flush to clear the line of nivolumab before starting the ipilimumab infusion. The second infusion will always be the ipilimumab study drug and will start after the infusion line has been flushed, filters changed and participant has been observed to ensure no infusion reaction has occurred. The time in between infusions is expected to be approximately 30 minutes but may be more or less depending on the situation.

There will be no dose escalations or reductions of nivolumab or ipilimumab allowed. Participants may be dosed no less than 12 days from the previous dose. Premedications are not recommended for the first dose of nivolumab and ipilimumab.

Participants should be carefully monitored for infusion reactions during nivolumab and ipilimumab administration. If an acute infusion reaction is noted, participants should be managed according to Section 7.4.3.

Doses of nivolumab and ipilimumab may be interrupted, delayed, or discontinued depending on how well the participants tolerates the treatment. If a participant requires a dose delay of > 7 days, the dose should be skipped.

Dose delay criteria can be found in Section 7.4.2.1, and discontinuation criteria can be found in Section 8.1.1.1. Criteria to resume treatment can be found in Section 8.1.2.1.

Please see the nivolumab IB, ipilimumab IB, and pharmacy manual for specific infusion preparation recommendations.

Nivolumab plus Platinum Doublet Chemotherapy (Arm C)

Participants are to begin study treatment within 7 calendar days of randomization. Participants are to receive nivolumab 360 mg IV plus histology dependent platinum doublet chemotherapy in 3-week cycles up to a maximum of 3 cycles:

- Non-squamous NSCLC: nivolumab at a flat dose of 360 mg as 30-minute IV infusion on Day 1, followed by pemetrexed at a dose of 500 mg/m^2 IV over 10 minutes or per institutional standard with cisplatin at a dose of 75 mg/m^2 IV over 120 minutes or per institutional standard, of a 3-week treatment cycle, for up to 3 cycles.
- Squamous NSCLC: nivolumab at a flat dose of 360 mg as 30 minute IV infusion on day 1, followed by gemcitabine at a dose of 1000 mg/m^2 or 1250 mg/m^2 (per local prescribing information) IV over 30 minutes or per institutional standard with cisplatin at a dose of 75 mg/m^2 IV over 120 minutes or per institutional standard, of a 3-week treatment cycle for up to 3 cycles. Gemcitabine will also be administered at a dose of 1000 mg/m^2 or 1250 mg/m^2 for a 30 minute IV infusion or per institutional standard on day 8 of each 3-week treatment cycle.

Approved v 9.0 [redacted]

- Any histology: nivolumab at a flat dose of 360 mg as 30-minute IV infusion on Day 1, followed by paclitaxel 175 or 200 mg/m^2 IV over 180 minutes or per institutional standard and carboplatin AUC 5 or 6 IV over 30 minutes or per institutional standard of a 3-week treatment cycle, for up to 3 cycles.
- Dosing calculations for chemotherapy should be based on the body surface area calculation assessed as per standard of care. The dose should remain the same if the participant's weight is within 10% of the baseline weight or prior dose weight All doses should be rounded up or to the nearest milligram or per institutional standard.

When study drugs (nivolumab and chemotherapy) are to be administered on the same day, nivolumab is to be administered first. Nivolumab infusion must be promptly followed by a saline flush to clear the line of nivolumab before starting the chemotherapy infusion. The time in between infusion of nivolumab and chemotherapy is expected to be approximately 30 minutes but may be more or less depending on the situation

There will be no dose escalations or reductions of nivolumab allowed. Participants may be dosed no less than 18 days from the previous dose. Premedications are not recommended for the first dose of nivolumab.

Participants should be carefully monitored for infusion reactions during nivolumab administration. If an acute infusion reaction is noted, participants should be managed according to Section 7.4.3.

Dose of nivolumab may be interrupted, delayed, or discontinued depending on how well the participants tolerates the treatment. If a participant requires a dose delay of > 7 days, the dose should be skipped.

Dose delay criteria can be found in Section 7.4.2.1, and discontinuation criteria can be found in Section 8.1.1.1. Criteria to resume treatment can be found in Section 8.1.2.1

Please see the nivolumab IB, ipilimumab IB, and pharmacy manual for specific infusion preparation recommendations.

Refer to instructions below for instructions for pemetrexed/cisplatin, gemcitabine/cisplatin, and paclitaxel/carboplatin administration.

Doses of gemcitabine and/or cisplatin may be modified, delayed, or discontinued depending on how well the participant tolerates the treatment. Dose modifications for toxicity will be performed according to Section 7.4.1. Dose delay criteria can be found in Section 7.4.2.2, and discontinuation criteria can be found in Section 8.1.1.2. Criteria to resume treatment can be found in Section 8.1.2.2.

Doses of pemetrexed and/or cisplatin may be interrupted, delayed, or discontinued depending on how well the participant tolerates the treatment. Dose modifications for toxicity will be performed according to Section 7.4.1. Dose delay criteria can be found in Section 7.4.2.2, and discontinuation criteria can be found in Section 8.1.1.2. Caution should be used when administering NSAIDs concurrently with pemetrexed to patients with mild to moderate renal insufficiency (creatinine

clearance from 45 to 79 mL/min). Pemetrexed should not be administered if the calculated CrCl is < 45 mL/min.

Doses of paclitaxel and/or carboplatin may be modified, delayed, or discontinued depending on how well the participant tolerates the treatment. Dose modifications for toxicity will be performed according to Section 7.4.1. Dose delay criteria can be found in Section 7.4.2.2, and discontinuation criteria can be found in Section 8.1.1.2. Criteria to resume treatment can be found in Section 8.1.2.2.

Dosing of all drugs should be delayed if any criteria in Section 7.4.2.1 (nivolumab), Section 7.4.2.2, or Section 7.4.2.3 (platinum doublet chemotherapy) are met. That is, nivolumab should be delayed if criteria for delay of platinum doublet chemotherapy are met, and platinum doublet chemotherapy should be delayed if criteria for delay of nivolumab are met.

Participants may resume dosing only when criteria for BOTH resumption of nivolumab (Section 8.1.2.1) AND platinum doublet chemotherapy (Section 8.1.2.2 and Section 8.1.2.3) are met. That is, nivolumab and platinum doublet chemotherapy must be administered together until treatment discontinuation criteria (Sections 8.1.1.1, 8.1.1.2, and 8.1.1.3) or up to 3 cycles of study treatment have been completed.

If a participant who is receiving nivolumab and platinum doublet chemotherapy experiences an adverse event and the investigator can attribute it to either nivolumab or chemotherapy, then either nivolumab or the chemotherapy agents can be discontinued, and the other agent(s) can be continued.

Platinum Doublet Chemotherapy (Arm B)

Vinorelbine/Cisplatin

Vinorelbine will be administered at a dose of 25 mg/m^2 or 30 mg/m^2 (per local prescribing information) over 10 minutes or per institutional standard as an IV infusion on Days 1 and 8 followed by cisplatin at a dose of 75 mg/m^2 as a 120-minute or per institutional standard IV infusion on Day 1 only, of a 3-week treatment cycle for up to 3 cycles.

Dosing calculations for vinorelbine should be based on the body surface area calculation assessed as per standard of care. The dose should remain the same if the participant's weight is within 10% of the baseline weight or prior dose weight. Doses of vinorelbine and/or cisplatin may be interrupted, delayed, reduced, or discontinued depending on how well the participant tolerates the treatment. Dose modifications for toxicity will be performed according to Section 7.4.1. Dose delay criteria can be found in Section 7.4.2.2, and discontinuation criteria can be found in Section 8.1.1.2. Criteria to resume treatment can be found in Section 8.1.2.2.

See below for the details regarding administration of cisplatin.

Docetaxel/Cisplatin

Docetaxel will be administered at a dose of 60 mg/m^2 or 75 mg/m^2 (per local prescribing information) as a 60 minute or per institutional standard IV infusion on Day 1 followed by cisplatin

Approved v 9.0 [redacted]

at a dose of 75 mg/m^2 as a 120 minute or per institutional standard IV infusion on Day 1 of a 3-week treatment cycle for up to 3 cycles.

Dosing calculations for docetaxel should be based on the body surface area calculation assessed as per standard of care. The dose should remain the same if the participant's weight is within 10% of the baseline weight or prior dose weight. Dose modifications for toxicity will be performed according to Section 7.4.1. Dose delay criteria can be found in Section 7.4.2.3, and dose discontinuation criteria can be found in Section 8.1.1.3. Criteria to resume treatment can be found in Section 8.1.2.3.

See below for the details regarding administration of cisplatin.

Premedications for use with docetaxel: Premedication with corticosteroids will be given to participants receiving docetaxel. The recommended premedication per the USPI and SmPC is dexamethasone 8 mg PO twice daily given one day before, on the day of, and one day after administration of chemotherapy. For institutions that have established an equivalent premedication regimen consistent with local docetaxel labeling, such premedication regimens will be permitted.

Gemcitabine/Cisplatin (Squamous histology only)

Participants will receive gemcitabine at a dose of 1000 mg/m^2 or 1250 mg/m^2 (per local prescribing information) as a 30 minute or per local institutional standard IV infusion on Days 1 and 8 followed by cisplatin at a dose of 75 mg/m^2 as a 120-minute or per institutional standard IV infusion on Day 1 of a 3-week treatment cycle for up to 3 cycles. Prolonged gemcitabine infusions > 60 minutes and/or administration more frequent than weekly has been associated with increased toxicity.

Dosing calculations for gemcitabine should be based on the body surface area calculation and may be capped per local standards. The dose may remain the same if the participant's weight is within 10% of the baseline weight or prior dose weight.

Doses of gemcitabine and/or cisplatin may be modified, delayed, or discontinued depending on how well the participant tolerates the treatment. Dose modifications for toxicity will be performed according to Section 7.4.1. Dose delay criteria can be found in Section 7.4.2.2, and discontinuation criteria can be found in Section 8.1.1.2. Criteria to resume treatment can be found in Section 8.1.2.2.

See below for the details regarding administration of cisplatin.

Pemetrexed/Cisplatin (non-Squamous histology only)

Pemetrexed will be administered at a dose of 500 mg/m^2 as a 10 minute or per institutional standard IV infusion and cisplatin will be administered at a dose of 75 mg/m^2 as a 120-minute or per institutional standard IV infusion on Day 1 of a 3-week treatment cycle for up to 3 cycles.

Dosing calculation for pemetrexed should be based on the body surface area calculation and may be capped per local standards. The dose may remain the same if the participant's weight is within 10% of the baseline weight or prior dose weight. All doses should be rounded up or to the nearest milligram per institutional standard.

Approved v 9.0 [redacted]

Doses of pemetrexed and/or cisplatin may be interrupted, delayed, or discontinued depending on how well the participant tolerates the treatment. Dose modifications for toxicity will be performed according to Section 7.4.1. Dose delay criteria can be found in Section 7.4.2.2, and discontinuation criteria can be found in Section 8.1.1.2. Caution should be used when administering NSAIDs concurrently with pemetrexed to patients with mild to moderate renal insufficiency (creatinine clearance from 45 to 79 mL/min). Pemetrexed should not be administered if the calculated CrCl is < 45 mL/min.

Premedications for use with pemetrexed: Oral corticosteroid should be given according to local standards at a dose equivalent to dexamethasone 4 mg twice daily on the day prior to, the day of, and the day after the administration of pemetrexed. Oral folic acid 350 to 1,000 mcg daily should be given starting approximately 1 week prior to the first dose of pemetrexed, with at least 5 doses of folic acid administered in the 7 days prior to the first dose. Oral folic acid should be continued daily throughout the treatment with pemetrexed and for 21 days after the last dose of pemetrexed. Intramuscular (IM) injection of vitamin B12 1000 mcg should be given approximately 1 week prior to the first dose of pemetrexed.

See below for the details regarding administration of cisplatin.

Paclitaxel/Carboplatin

Paclitaxel will be administered at a dose of 175 or 200 mg/m^2 as a 180 minute or per institutional standard IV infusion and carboplatin will be administered at a dose of AUC 5 or 6 as a 30-minute or per institutional standard IV infusion on Day 1 of a 3-week treatment cycle for up to 3 cycles.

Dosing calculations for paclitaxel should be based on the body surface area calculation and may be capped per local standards. The dose may remain the same if the participant's weight is within 10% of the baseline weight or prior dose weight.

Doses of paclitaxel and/or carboplatin may be modified, delayed, or discontinued depending on how well the participant tolerates the treatment. Dose modifications for toxicity will be performed according to Section 7.4.1. Dose delay criteria can be found in Section 7.4.2.2, and discontinuation criteria can be found in Section 8.1.1.2. Criteria to resume treatment can be found in Section 8.1.2.2.

Premedications for use with paclitaxel: Oral or IV corticosteroid should be given prior to paclitaxel according to local standard. Such premedication may consist of oral dexamethasone 20 mg 12 hours and 6 hours prior to paclitaxel administration. Oral or IV diphenydramine 50 mg (or its equivalent) and an H2-blocker (per local standard of care) should be administered 30 to 60 minutes prior to paclitaxel infusion. Antiemetic premedication will be administered according to local standards. Recommended antiemetic treatments are dexamethasone (dosing according to local standards; an equivalent dose of another corticosteroid may be substituted) and a 5-HT3 receptor antagonist (type per investigator discretion and local standards of care). Additional use of antiemetic premedications may be employed at the discretion of the investigator per local standards of care.

Approved v 9.0 [redacted]

All participants should be carefully monitored for infusion reactions during the paclitaxel administration. Participants should be treated in a facility with the necessary medical-resuscitation equipment and medications on hand to manage serious acute infusion reactions.

See below for the details regarding administration of carboplatin.

Cisplatin

Cisplatin 75 mg/m^2 as a 120 minute or per institutional standard IV infusion will be administered to participants as the second infusion. Dosing calculations for cisplatin should be based on the body surface area calculation and may be capped per local standards. The dose may remain the same if the participant's weight is within 10% of the baseline weight or prior dose weight.

Pretreatment hydration for cisplatin can follow local standard of care or use 1 to 2 liters of fluid infused IV for 8 to 12 hours prior to cisplatin infusion is recommended. Adequate hydration and urinary output must be maintained for at least 24 hours following cisplatin administration. Administration and monitoring should be performed according to local standards. Use of mannitol following the cisplatin infusion should also follow local standards of care.

All participants who will be receiving cisplatin should have audiometric testing performed prior to initiation of therapy and prior to subsequent doses of cisplatin, as per local or institutional standards of care. Participants considered to be at higher risk for ototoxicity with cisplatin have the option to receive carboplatin-based chemotherapy regimen after discussion with the Medical Monitor.

For participants who are unable to tolerate cisplatin, the reasons for intolerability should be documented. If the investigator would like to use a carboplatin-based regimen, the investigator should discuss this with and obtain approval from the Medical Monitor prior to utilization, except for opting for carboplatin plus paclitaxel.

Carboplatin

Carboplatin will be administered at a dose of AUC 5 or 6 as a 30-minute or as per institutional standard IV infusion, on Day 1 of each 3-week cycle.

Carboplatin should be given following gemcitabine, docetaxel, vinorelbine or pemetrexed on Day 1 of each cycle, and the carboplatin dose will be calculated using the Calvert formula as follows:

- Carboplatin dose (mg) will be calculated using the Calvert formula as follows = Target AUC x [(CrCl (mL/min) + 25]
- Creatinine clearance (CrCl) calculation is based on the Cockroft-Gault formula and should include the most recent serum creatinine and most recent weight. NOTE: If calculation of the CrCl by the Cockroft-Gault formula yields a result of > 125 mL/min, then a CrCl should be calculated by an alternative formula per institutional standards or capped at 125 mL/min.
- The dose of carboplatin may be capped per local standards.

Approved v 9.0 [redacted]

For All Arms Containing Chemotherapy:

Participants should begin study treatment within 7 calendar days of randomization. Doses of chemotherapy may be interrupted, delayed, or discontinued depending on how well the participants tolerates the treatment. If a dose is delayed for any reason, participants should be dosed no later than 7 days following a planned dose on any arm.

If more than 7 days delay is needed for any reason, the intended dose should be skipped. If a participant receiving chemotherapy on a Day 1 and Day 8 schedule (ie, cisplatin/gemcitabine) is unable to receive Day 1 of chemotherapy but recovers in time to receive the Day 8 dose, the Day 8 dose of chemotherapy may be administered.

Premedications: Antiemetic premedication will be administered according to local standards. Recommended antiemetic treatments are dexamethasone (dosing according to local standards; an equivalent dose of another corticosteroid may be substituted) and a 5-HT3 receptor antagonist (type per investigator discretion and local standards-of-care). Additional use of antiemetic premedications may be employed at the discretion of the Investigator.

7.2 Method of Treatment Assignment

CA209816 is an open-label, randomized trial. Participants with Stage IB (≥ 4 cm), II and IIIA (N2) considered resectable will be eligible to participate. After the participant's initial eligibility is established and informed consent has been obtained, the participant must be enrolled into the study by calling the IRT to obtain a participant number. Every participant that signs the informed consent form must be assigned a participant number in IRT. Specific instructions for using IRT will be provided to the investigational site in a separate document. The investigator or designee will register the participant for enrollment by following the enrollment procedures established by BMS.

The following information is required for enrollment:

- Date that informed consent was obtained
- Date of birth, where applicable by local regulations
- Gender at birth

Once enrolled in IRT, enrolled participants who have met all eligibility criteria will be ready to be randomized through IRT. PD-L1 expression data will be transferred directly from analyzing lab to IRT. The following information is required for participant randomization:

- Participant number
- Date of birth, where applicable per local regulations
- Stage of disease (IB, IIA, IIB or IIIA)
- PD-L1 status
- Gender

Approved v9.0 [redacted]

Participants meeting all eligibility criteria will be stratified according to PD-L1 status into 2 categories (≥1% and < 1% or not evaluable/indeterminate). Enrollment of participants with not evaluable or indeterminate PD-L1 status will be capped at 10%. Participants will also be stratified based disease stage (IB/II vs IIIA) and gender.

The exact procedures for using the IRT will be detailed in the IRT manual.

7.3 Blinding

This is an open-label study; blinding procedures between participants and investigators are not applicable. The BIPR and BICR will be blinded.

7.4 Dosage Modification

7.4.1 Dose Reductions for Platinum Doublet Chemotherapy

Dose reductions of platinum doublet chemotherapy may be required and will be performed according to Table 7.4.1-1. Chemotherapy dose reductions are permanent; once the dose of any chemotherapy agent is reduced, it may not be re-escalated in subsequent cycles. The dose reductions for each agent in the platinum doublet chemotherapy regimen are not linked and may be adjusted independently as summarized below.

Approved v1.0 [redacted]

Table 7.4.1-1: Dose Modifications of Chemotherapeutic Agents[a]

Dose Level	Vinorelbine	Docetaxel	Gemcitabine	Pemetrexed	Cisplatin	Carboplatin	Paclitaxel
Starting dose	25mg/m^2 or 30 mg/m^2	60 mg/mg^2 or 75 mg/m^2	1000 mg/m^2 or 1250 mg/m^2	500 mg/m^2	75 mg/m^2	AUC 5 or 6	175 or 200 mg/m^2
First dose reduction	75% of starting dose	75% of starting dose	75% of starting dose	75% of starting dose	75% of starting dose	AUC 4 or 5	150 mg/m^2
Second dose reduction	50% of the starting dose	50% of starting dose	50% of the starting dose	50% of the starting dose	50% of the starting dose	AUC 3 or 4	100 mg/m^2
Third dose reduction	Discontinue	Discontinue	Discontinue	Discontinue	Discontinue	Discontinue	Discontinue

[a] Follow local regulations if they are different than what appears in this table.

Any participants with 2 prior dose reductions for 1 agent who experiences a toxicity that would cause a third dose reduction must be discontinued from that agent.

7.4.1.1 Platinum Doublet Chemotherapy - Dose Reductions for Hematologic Toxicity

Dose modifications for hematologic toxicities (according to CTCAE version 4) are summarized in Table 7.4.1.1-1. Dose adjustments are based on nadir blood counts (assessed as per local standards) since the preceding drug administration. Dose level adjustments for platinum doublet chemotherapy are relative to that of the preceding administration. Generally, both chemotherapy agents in the platinum doublet chemotherapy regimen should be dose reduced together for hematologic toxicity. After the first cycle, growth factors may be used to assist hematologic recovery. Use local standards of care in the use of these supportive measures. Additionally, prophylactic antibiotics may be used according to local standards of care. Please report any antibiotic or growth factor use on the eCRF.

Approved v 1.0 [redacted]

Table 7.4.1.1-1: Dose Modifications for Hematologic Toxicity (based on Nadir Counts)[a]

Toxicity	Vinorelbine	Gemcitabine	Pemetrexed	Cisplatin	Carboplatin	Paclixatel
**Neutrophil Count Decreased**						
Grade 4 (< 500/mm^3 or < 0.5 x 10^9/L)	Reduce one dose level and consider prophylactic G-CSF in subsequent cycles	Reduce one dose level and consider prophylactic G-CSF in subsequent cycles	Reduce one dose level and consider prophylactic G-CSF in subsequent cycles	Reduce one dose level and consider prophylactic G-CSF in subsequent cycles	Reduce one dose level and consider prophylactic G-CSF in subsequent cycles	Reduce one dose level and consider prophylactic G-CSF in subsequent cycles
**Platelet Count Decreased**						
Grade 3 (25,000 to < 50,000/mm^3; 25.0 to < 50.0 x 10^9/L)	Reduce one dose level	Reduce one dose level	Reduce one dose level	Reduce one dose level	Reduce one dose level	Reduce one dose level
Grade 4 (< 25,000/mm^3; < 25.0 x 10^9/L)	Reduce one dose level	Reduce one dose level	Reduce one dose level	Reduce one dose level	Reduce one dose level	Reduce one dose level
**Hemoglobin**						
Grade 2 (< 10.0 to 8.0 g/dL; < 6.2 to 4.9 mmol/L; < 100 - 80 g/L)	Reduce one dose level	Reduce one dose level	Reduce one dose level	Reduce one dose level	Reduce one dose level	Reduce one dose level
Grade 3 (< 8.0 g/dL; < 4.9 mmol/L, < 80 g/L)	Reduce one dose level	Reduce one dose level	Reduce one dose level	Reduce one dose level	Reduce one dose level	Reduce one dose level
Grade 4 (Life threatening consequences)	Hold drug	Hold drug	Hold drug	Hold Drug	Hold Drug	Hold Drug

[a] If local standard for dose adjustments differ from those outlined, please discuss with the Medical Monitor.

Dose Modifications for Hematologic Toxicity for Docetaxel: Doses of docetaxel will be modified for participants who experience docetaxel-related events of febrile neutropenia, neutrophils < 500 cell/mm^3 for > 7 days during docetaxel treatment. Participants should have treatment delayed according to Section 7.4.2.3 and then resumed at 1 dose level reduction (75% of starting dose). Should these AEs occur after the first dose reduction, then a second dose reduction to (50% of starting dose) is permitted. If a third dose reduction is required, then the participants should discontinue docetaxel treatment.

7.4.1.2 Platinum Doublet Chemotherapy - Dose Reductions for Non-Hematologic Toxicities

Dose adjustments for platinum doublet chemotherapy for non-hematologic toxicities during treatment are described in Table 7.4.1.2-1. All dose reductions should be made based on the worst grade toxicity. Participants experiencing any of the toxicities detailed in Table 7.4.1.2-1 during the previous cycle should have their chemotherapy delayed until retreatment criteria are met (per Section 8.1.2.2) and then reduced for all subsequent cycles by 1 dose level or discontinued as appropriate. Dose levels for the 2 drugs in the platinum-doublet chemotherapy regimen are not linked and may be reduced independently, as summarized in Table 7.4.1.2-1.

Table 7.4.1.2-1: Dose Modifications for Non-hematologic Toxicity[a,b]

Toxicity	Vinorelbine	Gemcitabine	Pemetrexed	Cisplatin	Carboplatin	Paclitaxel
**Febrile Neutropenia** Grade ≥ 3	Reduce one dose level	Reduce one dose level	Reduce one dose level	Reduce one dose level	Reduce one dose level	Reduce one dose level
**Diarrhea** Grade ≥ 3	Reduce one dose level	Reduce one dose level	Reduce one dose level	No change	No change	Reduce one dose level
**Allergic reaction**[c] Grade ≥ 3	Reduce one dose level	Discontinue	Discontinue	Discontinue	Discontinue	Discontinue
**Neuropathy** Grade 2	No Change	No change	No change	Reduce one dose level[d]	No change	Reduce one dose level
**Neuropathy** Grade ≥ 3	Discontinue	Discontinue	Discontinue	Discontinue	Discontinue	Discontinue
**Calculated creatinine clearance < 50 mL/min**	No change	No change	Discontinue if creatinine clearance < 45 mL/min	Discontinue	Discontinue if creatinine clearance < 20 mL/min	No change
**Other Grade ≥ 3** toxicity (except for fatigue and transient arthralgia and myalgia)	Adjust as medically indicated	Adjust as medically indicated	Adjust as medically indicated	Adjust as medically indicated	Adjust as medically indicated	Adjust as medically indicated

[a] If local standard for dose adjustments differ from those outlined, please discuss with the Medical Monitor.

[b] Please see local drug label for additional adjustments.

[c] Only the drug(s) causing the hypersensitivity reaction or acute infusion reaction (≥ Grade 3) require(s) discontinuation. All other drugs may be continued.

[d] When given with pemetrexed, cisplatin should be reduced 2 dose levels (ie, by 50% for Grade 2 neuropathy.

Participants receiving docetaxel who develop Grade ≥ 3 peripheral neuropathy, or who otherwise meet criteria specified in Section 8.1.1.3, should discontinue docetaxel treatment.

Dose Modifications for Non-hematologic Toxicity for Docetaxel: Doses of docetaxel will be modified for participants who experience docetaxel-related events of severe or cumulative cutaneous reactions or other Grade 3/4 non-hematological toxicities during docetaxel treatment. Participants should have treatment delayed according to Section 7.4.2.3 and then resumed at 1 dose level reduction (75% of starting dose). Should these AEs occur after the first dose reduction, then a second dose reduction to (50% of starting dose) is permitted. If a third dose reduction is required, then the participants should discontinue docetaxel treatment.

7.4.2 Dose Delay

If any agent is delayed > 7 days, the dose should be skipped, and the participant should resume treatment at the next scheduled dose if criteria to resume treatment (Section 8.1.2) for the appropriate arm are met. If the last dose of study drug is delayed >7 but < 11 days, while it is recommended that this dose be skipped, if the investigator feels that it is appropriate to administer the last dose, discuss with the Medical Monitor before administering the last dose of study drug.

7.4.2.1 Nivolumab Dose Delay Criteria

Nivolumab administration should be delayed for the following:

- Grade 2 non-skin, drug-related adverse event, with the exception of fatigue
- Grade 2 drug-related creatinine, AST, ALT and/or Total Bilirubin abnormalities
- Grade 3 skin, drug-related adverse event
- Grade 3 drug-related laboratory abnormality, with the following exceptions:
  - Grade 3 lymphopenia or asymptomatic amylase or lipase does not require dose delay
  - Grade ≥ 3 AST, ALT, Total Bilirubin will require dose discontinuation (see Section 8.1.1.1)
- Any adverse event, laboratory abnormality, or intercurrent illness which, in the judgment of the investigator, warrants delaying the dose of study medication.

Participants who require delay of nivolumab should be re-evaluated weekly or more frequently if clinically indicated and resume nivolumab dosing when re-treatment criteria are met.

7.4.2.2 Dose Delay Criteria for Platinum Doublet Chemotherapy

Dosing of both drugs in the platinum doublet chemotherapy regimen selected should be delayed for any of the following on the Day 1 of each cycle:

- Absolute neutrophil count (ANC) ≤ 1500/µL
- Platelets < 100,000/mm^3
- Any Grade ≥ 2 non-skin, non-hematologic, drug-related adverse event (excluding Grade 2 alopecia, Grade 2 fatigue, and Grade 2 laboratory abnormalities)
- Any Grade ≥ 3 skin, drug-related adverse event

Approved v 9.0 [redacted]

- Any Grade ≥ 3 drug-related laboratory abnormality, with the following exceptions for lymphopenia, AST, ALT, or total bilirubin:
  - Grade 3 lymphopenia does not require dose delay.
  - If a participant has a baseline AST, ALT or total bilirubin that is within normal limits, delay dosing for drug-related Grade ≥ 2 toxicity.
  - If a participant has baseline AST, ALT, or total bilirubin within the Grade 1 toxicity range, delay dosing for drug-related Grade ≥ 3 toxicity.

Any adverse event, laboratory abnormality, or intercurrent illness which, in the judgment of the investigator, warrants delaying the dose of study medication. Investigators should consult local labeling for the chemotherapy drugs being administered to any given participant for additional guidance on dose delays. Participants receiving gemcitabine with cisplatin or carboplatin should omit the Day 8 gemcitabine dose for any of the following on Day 8 of any cycle:

- ANC < 1,000/mm^3
- Platelets < 75,000/mm^3

If any non-hematologic adverse event meeting the dose delay criteria above is felt to be related to only 1 particular agent in the platinum doublet chemotherapy regimen, then that agent alone may be omitted for that cycle while the other agent is given. In order to maintain synchronized dosing of the regimen, the omitted agent should be resumed with the next scheduled cycle once the AE has improved and retreatment criteria are met. Please refer to Section 7.4.1 to determine if dose reduction of the resumed agent is required.

If both drugs in the platinum doublet chemotherapy regimen are delayed, then the participant should be re-evaluated weekly or more frequently if clinically indicated until re-treatment criteria are met (as per Section 8.1.2.2 or Section 8.1.2.3).

7.4.2.3 Docetaxel Dose Delay Criteria

Docetaxel administration should be delayed for the following:

- Any Grade ≥ 2 non-skin, drug-related adverse event, with the following exceptions:
  - Grade 2 drug-related fatigue or laboratory abnormalities do not require a treatment delay
- Any Grade 3 skin, drug-related adverse event
- Any Grade 3 drug-related laboratory abnormality, with the following exceptions for lymphopenia, neutrophil count, AST, ALT, or total bilirubin:
  - Grade 3 lymphopenia does not require dose delay
  - Should not be given if neutrophil counts are < 1500 cells/mm^3
  - Should not be given if total bilirubin > upper limit of normal (ULN), or if AST and/or ALT > 1.5 x ULN concomitant with alkaline phosphatase > 2.5 x ULN

Approved v9.0 [redacted]

- Any AE, laboratory abnormality or inter-current illness which, in the judgment of the investigator, warrants delaying the dose of study medication

Subsequent dose reductions may be required as per Section 7.4.1.

Participants receiving docetaxel may receive growth factors (including G-CSF and erythropoietin) at the discretion of the investigator.

7.4.3 Treatment of Nivolumab- and Ipilimumab-related Infusion Reactions

Since nivolumab and ipilimumab contain only human immunoglobulin protein sequences, they are unlikely to be immunogenic and induce infusion or hypersensitivity reactions. However, if such a reaction were to occur, it might manifest with fever, chills, rigors, headache, rash, pruritus, arthralgias, hypotension, hypertension, bronchospasm, or other allergic-like reactions. All Grade 3 or 4 infusion reactions should be reported within 24 hours to the study Medical Monitor and reported as an SAE if it meets the criteria. Infusion reactions should be graded according to NCI CTCAE (Version 4) guidelines.

Treatment recommendations are provided below and may be modified based on local treatment standards and guidelines, as appropriate:

For Grade 1 symptoms: (mild reaction; infusion interruption not indicated; intervention not indicated):

- Remain at bedside and monitor participant until recovery from symptoms. The following prophylactic premedications are recommended for future infusions: diphenhydramine 50 mg (or equivalent) and/or acetaminophen/paracetamol 325 to 1000 mg at least 30 minutes before additional nivolumab administrations.

For Grade 2 symptoms: (moderate reaction required therapy or infusion interruption but responds promptly to symptomatic treatment (eg, antihistamines, non-steroidal anti-inflammatory drugs, narcotics, corticosteroids, bronchodilators, IV fluids); prophylactic medications indicated for ≤ 24 hours):

- Stop the nivolumab/ipilimumab infusion, begin an IV infusion of normal saline, and treat the participant with diphenhydramine 50 mg IV (or equivalent) and/or acetaminophen/paracetamol 325 to 1000 mg; remain at bedside and monitor participant until resolution of symptoms. Corticosteroid and/or bronchodilator therapy may also be administered as appropriate. If the infusion is interrupted, then restart the infusion at 50% of the original infusion rate when symptoms resolve; if no further complications ensue after 30 minutes, the rate may be increased to 100% of the original infusion rate. Monitor participant closely. If symptoms recur, then no further nivolumab will be administered at that visit.
- For future infusions, the following prophylactic premedications are recommended: diphenhydramine 50 mg (or equivalent) and/or acetaminophen/paracetamol 325 to 1000 mg should be administered at least 30 minutes before nivolumab infusions. If necessary, corticosteroids (up to 25 mg of hydrocortisone or equivalent) may be used.

Approved v9.0 [redacted]

For Grade 3 or 4 symptoms: (severe reaction, Grade 3: prolonged [ie, not rapidly responsive to symptomatic medication and/or brief interruption of infusion]; recurrence of symptoms following initial improvement; hospitalization indicated for other clinical sequelae (eg, renal impairment, pulmonary infiltrates). Grade 4: Life-threatening; pressor or ventilatory support indicated):

- Immediately discontinue infusion of nivolumab/ipilimumab. Begin an IV infusion of normal saline and treat the participant as follows: Recommend bronchodilators, epinephrine 0.2 to 1 mg of a 1:1000 solution for subcutaneous administration or 0.1 to 0.25 mg of a 1:10,000 solution injected slowly for IV administration, and/or diphenhydramine 50 mg IV with methylprednisolone 100 mg IV (or equivalent), as needed. Participant should be monitored until the Investigator is comfortable that the symptoms will not recur. Nivolumab will be permanently discontinued. Investigators should follow their institutional guidelines for the treatment of anaphylaxis. Remain at bedside and monitor participant until recovery of the symptoms.

In case of late-occurring hypersensitivity symptoms (eg, appearance of a localized or generalized pruritus within 1 week after treatment), symptomatic treatment may be given (eg, oral antihistamine or corticosteroids).

7.5 Preparation/Handling/Storage/Accountability

The investigational product should be stored in a secure area according to local regulations. It is the responsibility of the investigator to ensure that investigational product is only dispensed to study participants. The investigational product must be dispensed only from official study sites by authorized personnel according to local regulations.

The product storage manager should ensure that the study treatment is stored in accordance with the environmental conditions (temperature, light, and humidity) as determined by BMS. If concerns regarding the quality or appearance of the study treatment arise, the study treatment should not be dispensed and contact BMS immediately.

Study treatment not supplied by BMS will be stored in accordance with the package insert.

Investigational product documentation (whether supplied by BMS or not) must be maintained that includes all processes required to ensure drug is accurately administered. This includes documentation of drug storage, administration and, as applicable, storage temperatures, reconstitution, and use of required processes (eg, required diluents, administration sets).

Handling of chemotherapy should be according to local prescribing information.

Please refer to the current version of the Investigator Brochures (IBs) and/or the Pharmacy Manual for complete storage, handling, and preparation information. Further guidance and information for final disposition of unused study treatment are provided in Appendix 7 and the Pharmacy Manual.

7.5.1 Retained Samples for Bioavailability / Bioequivalence

At the time of receipt of the investigational product by the investigator or designee's, BMS will specify the appropriate number of containers or units to select for retention, the conditions of sample storage, required duration of sample retention, and provisions for returning or disposing of

Approved v9.0 [redacted]

the investigational product. When samples are selected, containers or units should be placed in packaging with a tamper evident seal provided by BMS. Package labeling should clearly identify the contents as bioavailability/bioequivalence (BA/BE) samples and state that the investigational product should be stored in the restricted area with limited access.

7.6 Treatment Compliance

Treatment compliance will be monitored by drug accountability as well as the participant's medical record and eCRF.

7.7 Concomitant Therapy

7.7.1 Prohibited and/or Restricted Treatments

The following medications are prohibited during the study (unless utilized to treat a drug related adverse event):

- Immunosuppressive agents
- Immunosuppressive doses of systemic corticosteroids (except as stated in Section 7.7.3)
- Any concurrent anti-neoplastic therapy (ie, chemotherapy, hormonal therapy, immunotherapy, extensive, non-palliative radiation therapy, or standard or investigational agents for treatment of NSCLC)
- Investigators should refer to the local product labeling for the chemotherapy drugs selected for use in Arm B and Arm C and for additional prohibited and restricted concomitant medications.
- Caution should be used regarding the use of herbal medication, as there may be as yet unknown interactions with nivolumab and ipilimumab. Discontinuation of the use of herbal medications prior to the study enrollment is encouraged.
- Live vaccines (eg, yellow fever, MMR, nasal flu vaccine, chicken pox [varicella]) should be avoided while receiving neoadjuvant or adjuvant study drug
- Avoid using concomitant strong CYP3A4 inhibitors or inducers with docetaxel, vinorelbine, or paclitaxel. Avoid using concomitant strong CYP2C8 inhibitors or inducers with paclitaxel. Consult the local prescribing information for further guidance.
- Avoid the concomitant administration of substances that are also tubularly secreted (eg, probenecid) which could potentially result in delayed clearance of pemetrexed.

7.7.2 Other Restrictions and Precautions

Participants with a condition requiring systemic treatment with either corticosteroids (> 10 mg daily prednisone equivalent) or other immunosuppressive medications within 14 days of randomization are excluded. Inhaled or topical steroids, and adrenal replacement steroid doses > 10 mg daily prednisone equivalent, are permitted in the absence of active autoimmune disease.

It is the local imaging facility's responsibility to determine, based on participants attributes (eg, allergy history, diabetic history and renal status), the appropriate imaging modality and contrast regimen for each participant. Imaging contraindications and contrast risks should be considered in this assessment. Participants with renal insufficiency should be assessed as to whether or not they should receive contrast and if so, what type and dose of contrast is appropriate.

Approved v9.0 [redacted]

Specific to MRI, participants with severe renal insufficiency (ie, estimated glomerular filtration rate [eGFR] < 30 mL/min/1.73 m^2) are at increased risk of nephrogenic systemic fibrosis. MRI contrast should not be given to this population. In addition, participants are excluded from MRI if they have tattoos, metallic implants, pacemakers, etc. The ultimate decision to perform MRI in an individual participant in this study rests with the site radiologist, the investigator, and the standard set by the local Ethics Committee.

7.7.3 Permitted Therapy

Participants are permitted the use of topical, ocular, intra-articular, intranasal, and inhalational corticosteroids (with minimal systemic absorption). Adrenal replacement steroid doses > 10 mg daily prednisone are permitted. A brief (less than 3 weeks) course of corticosteroids for prophylaxis (eg, contrast dye allergy) or for treatment of non-autoimmune conditions (eg, delayed-type hypersensitivity reaction caused by a contact allergen) is permitted.

7.8 Treatment After the End of the Study

At the end of the study period, BMS will not continue to provide BMS supplied study treatment to participants/investigators unless BMS chooses to extend the study. The investigator should ensure that the participant receives appropriate standard of care lung cancer treatment.

8 DISCONTINUATION CRITERIA

8.1 Discontinuation from Study Treatment

Participants MUST discontinue investigational product (and non-investigational product at the discretion of the investigator) for any of the following reasons:

- Participant's request to stop study treatment. Participants who request to discontinue study treatment will remain in the study and must continue to be followed for protocol specified follow-up procedures. The only exception to this is when a participant specifically withdraws consent for any further contact with him/her or persons previously authorized by participant to provide this information. Participant should notify the investigator of the decision to withdraw from future follow-up in writing, whenever possible, and this should be documented in the medical records.
- Any clinical adverse event (AE), laboratory abnormality or intercurrent illness which, in the opinion of the investigator, indicates that continued participation in the study is not in the best interest of the participant
- Termination of the study by Bristol-Myers Squibb Company (BMS)
- Loss of ability to freely provide consent through imprisonment or involuntarily incarceration for treatment of either a psychiatric or physical (eg, infectious disease) illness

Refer to the Schedule of Activities for data to be collected at the time of neoadjuvant treatment discontinuation and subsequent study procedures and for any further evaluations that can be completed.

In the case of pregnancy, the investigator must immediately notify the BMS Medical Monitor/designee of this event. In the event a normal healthy female participant becomes pregnant

Approved v 1.0 [redacted]

during a clinical trial, the study treatment must be discontinued immediately. In most cases, the study treatment will be permanently discontinued in an appropriate manner (eg, dose tapering if necessary for participant safety). Please call the BMS Medical Monitor within 24 hours of awareness of the pregnancy. If the investigator determines a possible favorable benefit/risk ratio that warrants continuation of study treatment, a discussion between the investigator and the BMS Medical Monitor/designee must occur.

All participants who discontinue study treatment should comply with protocol specified procedures as outlined in Section 2. The only exception to this requirement is when a participant withdraws consent for all study procedures including post-treatment study follow-up or loses the ability to consent freely (ie, is imprisoned or involuntarily incarcerated for the treatment of either a psychiatric or physical illness).

If study treatment is discontinued prior to the participant's completion of the study, the reason for the discontinuation must be documented in the participant's medical records and entered on the appropriate case report form (CRF) page.

8.1.1 Study Treatment Discontinuation Criteria

Doses that are missed or delayed by > 7 days will not be replaced, and participants should proceed to surgery within the indicated timelines. Participants who discontinue their assigned study treatment prematurely will not be allowed to receive subsequent neoadjuvant therapy, and participants should proceed to surgery within the indicated timelines.

In Arm C, nivolumab and platinum doublet chemotherapy can be discontinued independently based on their discontinuation criteria.

8.1.1.1 Nivolumab Discontinuation Criteria

Nivolumab treatment should be permanently discontinued for the following:

- Any Grade 2 drug-related uveitis, eye pain or blurred vision that does not respond to topical therapy and does not improve to Grade 1 severity within the re-treatment period OR requires systemic treatment
- Any Grade 3 non-skin, drug-related adverse event lasting > 7 days, or recurs with the following exceptions for laboratory abnormalities, diarrhea, colitis, neurologic toxicity, drug-related uveitis, pneumonitis, bronchospasm, hypersensitivity reactions, infusion reactions, and endocrinopathies:
  - Grade 3 drug-related diarrhea, colitis, neurologic toxicity, uveitis, pneumonitis, bronchospasm, hypersensitivity reaction, or infusion reaction of any duration requires discontinuation
  - Grade 3 drug-related endocrinopathies, adequately controlled with only physiologic hormone replacement do not require discontinuation. Adrenal insufficiency requires discontinuation regardless of control with hormone replacement.

Approved v9.0 [redacted]

  - Grade 3 drug-related laboratory abnormalities do not require treatment discontinuation except:
    - ♦ Grade 3 drug-related thrombocytopenia > 7 days or associated with bleeding requires discontinuation
    - ♦ Any drug-related liver function test (LFT) abnormality that meets the following criteria require discontinuation:
      - o Grade ≥ 3 drug-related AST, ALT or Total Bilirubin requires discontinuation*
- * In most cases of Grade 3 AST or ALT elevation, study drug(s) will be permanently discontinued. If the investigator determines a possible favorable benefit/risk ratio that warrants continuation of study drug(s), a discussion between the investigator and the BMS Medical Monitor/designee must occur.
      - o Concurrent AST or ALT > 3 x ULN and total bilirubin > 2x ULN
- Any Grade 4 drug-related adverse event or laboratory abnormality (including but not limited to creatinine, AST, ALT, or Total Bilirubin), except for the following events which do not require discontinuation:
  - Grade 4 neutropenia ≤ 7 days
  - Grade 4 lymphopenia or leukopenia or asymptomatic amylase or lipase
  - Isolated Grade 4 electrolyte imbalances/abnormalities that are not associated with clinical sequelae and are corrected with supplementation/appropriate management within 72 hours of their onset
  - Grade 4 drug-related endocrinopathy adverse events, such as, hyper- or hypothyroidism, or glucose intolerance, which resolve or are adequately controlled with physiologic hormone replacement (corticosteroids, thyroid hormones) or glucose-controlling agents, respectively, may not require discontinuation after discussion with and approval from the BMS Medical Monitor.
- Any event that leads to delay in dosing of any study drug for > 6 weeks from the previous dose requires discontinuation of that drug(s) with the following exception:
  - Dosing delays lasting > 6 weeks from the previous dose that occur for non-drug-related reasons may be allowed if approved by the BMS medical monitor. Prior to re-initiating treatment in a participant with a dosing delay lasting > 6 weeks, the BMS Medical Monitor must be consulted. Periodic study visits to assess safety and laboratory studies should also continue every 6 weeks or more frequently if clinically indicated during such dosing delays.
- Any adverse event, laboratory abnormality, or intercurrent illness which, in the judgment of the Investigator, presents a substantial clinical risk to the participant with continued nivolumab dosing.

8.1.1.2 Platinum Doublet Chemotherapy Dose Discontinuation

Except where specified below, both chemotherapy drugs in the platinum doublet chemotherapy regimen should be discontinued for any of the following:

- Any Grade ≥ 3 peripheral neuropathy

Approved v 9.0 [redacted]

- Grade ≥ 3 drug-related thrombocytopenia associated with clinically significant bleeding
- Any drug-related liver function test (LFT) abnormality that meets the following criteria requires discontinuation:
  - AST or ALT > 5-10x ULN for > 2 weeks
  - AST or ALT > 10x ULN
  - Total bilirubin > 5 x ULN
  - Concurrent AST or ALT > 3 x ULN and total bilirubin > 2 x ULN
- Any cisplatin-related decrease in creatinine clearance to < 50 mL/min (using the Cockroft Gault formula) requires discontinuation of cisplatin. The other chemotherapeutic agent may be continued, and the platinum agent may be switched to carboplatin, the investigator should discuss this with and obtain approval from the Medical Monitor prior to switch.
- Pemetrexed should be discontinued when creatinine clearance to < 45 mL/min (using the Cockroft Gault formula)
- Any drug-related adverse event which recurs after 2 prior dose reductions for the same drug-related adverse event (as specified in Section 7.4.1.1 and Section 7.4.1.2) requires discontinuation of the drug(s) which was/were previously dose reduced.
- Any Grade ≥ 3 drug-related hypersensitivity reaction or infusion reaction requires discontinuation of the drug(s) felt to be causing the reaction. The drug not felt to be related to the hypersensitivity reaction or infusion reaction may be continued.
- Any Grade 4 drug-related adverse event which the investigator deems is inappropriate to be managed by dose reduction(s) requires discontinuation of the drug(s) felt to be causing the event. The drug not felt to be related to the event may be continued.
- Any event that leads to delay in dosing of any study drug(s) for > 6 weeks from the previous dose requires discontinuation of that drug(s) with the following exception:
  - Dosing delays lasting > 6 weeks from the previous dose that occur for non-drug-related reasons may be allowed if approved by the BMS medical monitor. Prior to re-initiating treatment in a participant with a dosing delay lasting > 6 weeks, the BMS Medical Monitor must be consulted. Periodic study visits to assess safety and laboratory studies should also continue every 6 weeks or more frequently if clinically indicated during such dosing delays.
- Any adverse event, laboratory abnormality, or intercurrent illness which, in the judgment of the Investigator, presents a substantial clinical risk to the participant with continued platinum doublet chemotherapy dosing. Investigators should consult local labeling for the chemotherapy drugs being administered to any given participant for additional guidance on dose discontinuation.

For participants in Arms C, if the investigator is unable to determine whether an adverse event is due to nivolumab or to platinum doublet chemotherapy, then all drugs must be discontinued.

Approved v 9.0 [redacted]

8.1.1.3 Docetaxel Dose Discontinuation

Docetaxel treatment should be permanently discontinued for the following:

- Any ≥ Grade 3 peripheral neuropathy
- Any Grade 3 non-skin, drug-related adverse event lasting > 7 days, with the following exceptions for laboratory abnormalities:
  - Grade 3 drug-related laboratory abnormalities do not require treatment discontinuation except
    - ♦ Grade 3 drug-related thrombocytopenia associated with bleeding requires discontinuation.
    - ♦ Any drug-related liver function test (LFT) abnormalities that meets the following criteria require discontinuation:
      - o AST or ALT > 5 - 10x ULN for > 2 weeks
      - o AST or ALT > 10x ULN
      - o Total bilirubin > 5x ULN
      - o Concurrent AST or ALT > 3x ULN and total bilirubin > 2x ULN
- Any Grade 4 drug-related adverse event including laboratory abnormalities except for the following events which do not require discontinuation:
  - Grade 4 neutropenia >7 days despite 2 prior docetaxel reductions requires discontinuation
  - Grade 4 lymphopenia or leukopenia does not require discontinuation
  - Isolated Grade 4 electrolyte imbalances/abnormalities that are not associated with clinical sequelae and are corrected with supplementation/appropriate management within 72 hours of their onset does not require discontinuation.
- Any dosing interruption lasting > 6 weeks with the following exceptions:
  - Dosing interruptions > 6 weeks that occur for non-drug-related reasons may be allowed if approved by the BMS Medical Monitor/Study Director. Prior to re-initiating treatment in a participant with a dosing interruption lasting > 6 weeks, the BMS Medical Monitor/Study Director must be consulted. Tumor assessments should continue as per protocol even if dosing is interrupted.

8.1.2 Criteria to Resume Treatment

8.1.2.1 Criteria to Resume Nivolumab Treatment

Participants may resume treatment with study drug when the drug-related AE(s) resolve to Grade ≤ 1 or baseline value, with the following exceptions:

- Participants may resume treatment in the presence of Grade 2 fatigue
- Participants who have not experienced a Grade 3 drug-related skin AE may resume treatment in the presence of Grade 2 skin toxicity
- For participants with Grade 2 AST, ALT and/or Total Bilirubin Abnormalities, dosing may resume when laboratory values return to baseline and management with corticosteroids, if needed, is complete.

- Participants with combined Grade 2 AST/ALT AND total bilirubin values meeting discontinuation parameters (Section 8.1.1.1) should have treatment permanently discontinued.
- Drug-related pulmonary toxicity, diarrhea or colitis must have resolved to baseline before treatment is resumed. Participants with persistent Grade 1 pneumonitis after completion of a steroid taper over at least 1 month may be eligible for retreatment if discussed with and approved by BMS Medical Monitor.

Participants with drug-related endocrinopathies adequately controlled with only physiologic hormone replacement may resume treatment after consultation with the BMS Medical Monitor. Please refer to the Management Algorithms in Appendix 4.

8.1.2.2 Criteria to Resume Platinum Doublet Chemotherapy Dosing

- Participants may resume treatment with platinum doublet chemotherapy when the ANC returns to ≥ 1500/µL, the platelet count returns to ≥ 100,000/mm^3, and all other drug-related toxicities have returned to baseline or Grade ≤ 1 (or Grade ≤ 2 for alopecia and fatigue)
- If a participant fails to meet criteria for reinitiating treatment, then treatment should be delayed, and the participant should be re-evaluated weekly or more frequently as clinically indicated
- When resuming platinum doublet chemotherapy treatment, please follow the dose reduction recommendations in Section 7.4.1

8.1.2.3 Criteria to Resume Treatment with Docetaxel

Participants may resume treatment with docetaxel when the drug-related AE(s) resolve(s) to Grade ≤ 1 or baseline, with the following exceptions:

- Participants may resume treatment in the presence of Grade 2 fatigue
- Participants who have not experienced a Grade 3 drug-related skin AE may resume treatment in the presence of Grade 2 skin toxicity
- Participants with decreased neutrophil counts, or with elevations in total bilirubin, AST or ALT must meet criteria for resuming treatment according to the boxed warning contained within the docetaxel Prescribing Information
- Participants with combined Grade 2 AST/ALT AND total bilirubin values meeting discontinuation parameters (Section 8.1.1.3) should have treatment permanently discontinued

When resuming docetaxel treatment, please follow the dose reduction recommendations noted in Section 7.4.1.

8.1.3 Post Study Follow-up

In this study, EFS and pCR are multiple primary endpoints. Post study follow-up is of critical importance and is essential to preserving participant safety and the integrity of the study. Participants who discontinue study treatment must continue to be followed for collection of

Approved v 9.0 [redacted]

outcome and/or survival follow-up data as required and in line with Section 2 until death or the conclusion of the study.

BMS may request that survival data be collected on all treated/randomized participants outside of the protocol defined window (Table 2-3 and Table 2-4). At the time of this request, each participant will be contacted to determine their survival status unless the participant has withdrawn consent for all contacts or is lost to follow-up.

8.2 Discontinuation from the Study

Participants who request to discontinue study participation will remain in the study and must continue to be followed for protocol specified follow-up procedures. The only exception to this is when a participant specifically withdraws consent for any further contact with him/her or persons previously authorized by participant to provide this information.

- Participants should notify the investigator of the decision to withdraw consent from future follow-up **in writing**, whenever possible.
- The withdrawal of consent should be explained in detail in the medical records by the investigator, as to whether the withdrawal is from further treatment with study treatment only or also from study procedures and/or post treatment study follow-up, and entered on the appropriate CRF page.
- In the event that vital status (whether the participant is alive or dead) is being measured, publicly available information should be used to determine vital status only as appropriately directed in accordance with local law.
- If the participant withdraws consent for disclosure of future information, the sponsor may retain and continue to use any data collected before such a withdrawal of consent.

8.3 Lost to Follow-Up

- All reasonable efforts must be made to locate participants to determine and report their ongoing status. This includes follow-up with persons authorized by the participant.
- Lost to follow-up is defined by the inability to reach the participant after a minimum of **three** documented phone calls, faxes, or emails as well as lack of response by participant to one registered mail letter. All attempts should be documented in the participant's medical records.
- If it is determined that the participant has died, the site will use permissible local methods to obtain date and cause of death.
- If investigator's use of third-party representative to assist in the follow-up portion of the study has been included in the participant's informed consent, then the investigator may use a Sponsor retained third-party representative to assist site staff with obtaining participant's contact information or other public vital status data necessary to complete the follow-up portion of the study.
- The site staff and representative will consult publicly available sources, such as public health registries and databases, in order to obtain updated contact information.

Approved v 9.0 [redacted]

- If after all attempts, the participant remains lost to follow-up, then the last known alive date as determined by the investigator should be reported and documented in the participant's medical records.

9 STUDY ASSESSMENTS AND PROCEDURES

- Study procedures and timing are summarized in the Schedule of Activities.
- Protocol waivers or exemptions are not allowed.
- All immediate safety concerns must be discussed with the Sponsor immediately upon occurrence or awareness to determine if the participant should continue or discontinue treatment.
- Adherence to the study design requirements, including those specified in the Schedule of Activities, is essential and required for study conduct.
- All screening evaluations must be completed and reviewed to confirm that potential participants meet all eligibility criteria before randomization. The investigator will maintain a screening log to record details of all participants screened and to confirm eligibility or record reasons for screening failure, as applicable.
- Procedures conducted as part of the participant's routine clinical management (eg, blood count) and obtained before signing of informed consent may be utilized for screening or baseline purposes provided the procedure meets the protocol-defined criteria and has been performed within the timeframe defined in the Schedule of Activities.

Additional measures, including non-study required laboratory tests, should be performed as clinically indicated or to comply with local regulations. Laboratory toxicities (eg, suspected drug induced liver enzyme evaluations) will be monitored during the follow-up phase via on site/local labs until all study drug related toxicities resolve, return to baseline, or are deemed irreversible.

If a participant shows pulmonary-related signs (hypoxia, fever) or symptoms (eg, dyspnea, cough, fever) consistent with possible pulmonary adverse events, the participant should be immediately evaluated to rule out pulmonary toxicity, according to the suspected pulmonary toxicity management algorithm in the BMS-936558 (nivolumab) Investigator Brochure.

Some of the assessments referred to in this section may not be captured as data in the eCRF. They are intended to be used as safety monitoring by the treating physician. Additional testing or assessments may be performed as clinically necessary or where required by institutional or local regulations.

9.1 Efficacy Assessments

The primary efficacy assessments will be EFS and pCR, and a key secondary endpoint is MPR rate (defined as number of randomized participants with ≤ 10% residual tumor in lung and lymph nodes as evaluated by BIPR, divided by the number of randomized participants for each treatment group), which will be assessed by BIPR at the time of definitive surgery. Surgery should be

Approved v9.0 [redacted]

performed within 6 weeks after completing up to 3 cycles (last dose) of neoadjuvant treatment. The following lymph node levels should be sampled at the time of definitive surgery:

- For right-sided tumor: Levels 4, 7, 9R, 10R and 11 R
- For left-sided tumor: Levels 5 and/or 6; 7, 9L, 10L, and 11L

Tumor and lymph node collection from definitive surgical resection and sampling of fresh tumor sample in RNAlater for biomarker studies (as applicable dependent on the size of residual tumor) is mandatory on the day of surgery. Processing of the remainder of the specimens for histopathologic analysis should be performed within 72 hours of the procedure. Sections will be used for central pathology review assessing pCR and MPR. **Any tumor, tumor bed, or lymph node specimens that are reviewed locally must be submitted for central pathology review.** Gross examination on the entire specimen should be performed which includes all tumor, associated lymph node tissue, and uninvolved parenchyma. The specimen should be sectioned at 0.5 cm intervals, and blocks should be submitted for the full cross section for every other 0.5 cm interval. When estimating viable tumors, in situ carcinoma should not be included. For very large tumors with no gross evidence of response, a minimum of 1 slide/cm is required for assessment of major pathologic response. Sites are strongly encouraged to submit the diagnostic H&E stained slides assessed by the local pathologist for central pathology review. The final diagnostic pathology report must be submitted with the slides and blocks for review. Tumor sample acquisition guidelines and submission process will be outlined in the study Laboratory Manual to be provided by the central lab.

Radiographic assessments will be performed with the Schedule Activities in Table 2-2.

Disease recurrence, change in tumor measurements, and tumor response will be assessed by the BICR using the RECIST 1.1 criteria.

9.1.1 Imaging Assessment for the Study

Study evaluations will take place in accordance with the Schedule of Activities.

Screening (baseline) assessments are to be performed within 28 days prior to randomization. PET/CT with contrast should be assessed at baseline. A separate CT with contrast of the chest, abdomen, and other suspected areas (as well as the PET/CT) is required if the CT component of a PET/CT is not of sufficient diagnostic quality for RECIST 1.1 measurements. Participants with suspected brain metastases and all those with stage II disease or higher should be evaluated with MRI/CT of the brain pre- and post-contrast. Tumor assessments should be performed following RECIST 1.1 criteria.

A preoperative PET/CT with contrast should be acquired within 14 days of surgery. A separate CT with contrast of the chest, abdomen, and other suspected areas (as well as the PET/CT) is required if the CT component of a PET/CT is not sufficient diagnostic quality for RECIST 1.1 measurements.

Approved v 9.0 [redacted]

Postoperative assessments with CT with contrast of the chest including the adrenal glands and CT or MRI of other additional suspected/known sites of disease. The first tumor assessment should occur 12 weeks (± 7 days) after definitive surgery per RECIST 1.1 and then should occur every 12 weeks (± 7 days) for 2 years (104 weeks), then every 6 months (24 weeks ± 7 days) for 3 years, and then every year (52 weeks ± 7 days) for 5 years or until disease recurrence or progression confirmed by BICR. The same imaging method should be used as the screening/baseline. For participants who did not receive definitive surgery AND without tumor progression confirmed by BICR, the first tumor assessments should be done 12 weeks (± 7 days) following the tumor restaging, subsequent tumor assessments will be performed with the same frequency and methods as described for those having received definitive surgery; in case the planned initiation of subsequent anti-cancer therapy is within 12 weeks of restaging, every effort should be made to repeat tumor assessment prior to subsequent anti-cancer therapy.

Images will be submitted to the third-party radiology vendor for central review as they are performed on an ongoing basis. Sites will be trained prior to scanning the first study participant. Image acquisition guidelines and submission process will be outlined in the study Imaging Manual to be provided by the radiology vendor. Any incidental findings of potential clinical relevance that are not directly associated with the objectives of the protocol should be evaluated and handled by the Study Investigator as per standard medical/clinical judgement.

9.1.2 Patient-reported Outcomes

The evaluation of health-related quality of life (QoL) is an increasingly important aspect of a clinical efficacy in oncology trials. Such data provides an understanding of the impact of treatment from the participants' perspective and offers insights into the patient experience that may not be captured through physician reporting. Generic health-related QoL scales additionally provide data necessary in calculating utility values for health economic models.

Participants will be asked to complete the 3-level version of the EQ-5D before any clinical activities are performed during on-treatment clinic visits, at Post-neoadjuvant Visits 1 and 2, and at designated visits or during phone calls during the Survival Follow-up Phase. The questionnaire will be provided in the participants preferred language and may be administered by telephone during the Survival Follow-up Phase. A standardized script will be used to facilitate telephone administration of the EQ-5D-3L. Table 2-2, Table 2-3, and Table 2-4 provide information regarding the timing of participant-reported outcomes assessments.

The EQ-5D-3L is a standardized instrument used to measure self-reports of health status and functioning. The instrument's descriptive system consists of 5 dimensions: mobility, self-care, usual activities, pain/discomfort, and anxiety/depression. Each dimension has 3 levels, reflecting "no health problems," "moderate health problems," and "extreme health problems." A dimension for which there are no problems is said to be at level 1, while a dimension for which there are extreme problems is said to be at level 3. Thus, the vectors 11111 and 33333 represent the best health state and the worst health state, respectively, described by the EQ-5D-3L. Altogether, the instrument describes $3^5 = 243$ health states. Empirically derived weights can be applied to an individual's responses to the EQ-5D-3L descriptive system to generate an index measuring the

Approved v 9.0 [redacted]

value to society of his or her current health. Such preference-weighting systems have been developed for Japan, UK, US, Spain, Germany, and numerous other populations. In addition, the EQ-5D-3L includes a visual analog scale that allows respondents to rate their own current health on a 101-point scale ranging from “best imaginable” to “worst imaginable” health. The EQ-5D is available for use in over 150 languages.

9.2 Adverse Events

The definitions of an AE or serious adverse event (SAE) can be found in Appendix 8.

AEs will be reported by the participant (or, when appropriate, by a caregiver, surrogate, or the participant's legally authorized representative).

The investigator and any designees are responsible for detecting, documenting, and reporting events that meet the definition of an AE or SAE and remain responsible for following up AEs that are serious, considered related to the study treatment or the study, or that caused the participant to discontinue before completing the study.

Contacts for SAE reporting specified in Appendix 8.

Immune-mediated adverse events (IMAEs) are AEs consistent with an immune-mediated mechanism or immune-mediated component for which non-inflammatory etiologies (eg, infection or tumor progression) have been ruled out. IMAEs can include events with an alternate etiology which were exacerbated by the induction of autoimmunity. Information supporting the assessment will be collected on the participant’s case report form.

9.2.1 Time Period and Frequency for Collecting AE and SAE Information

The collection of nonserious AE information should begin at initiation of study treatment until 100 days after the last dose of neoadjuvant therapy or 90 days after surgery, whichever is longer, and 30 days after the last dose of adjuvant therapy, at the time points specified in the Schedule of Activities (Section 2). Nonserious AE information should also be collected from the start of a placebo lead-in period or other observational period intended to establish a baseline status for the participants.

The Investigator Brochure (IB) represents the Reference Safety Information to determine expectedness of serious adverse events for expedited reporting. Following the participant’s written consent to participate in the study, all SAEs, whether related or not related to study drug, must be collected, including those thought to be associated with protocol-specified procedures.

All SAEs must be collected that occur during the screening period and 100 days after the last dose of neoadjuvant therapy or 90 days after surgery, whichever is longer, and 30 days after the last dose of adjuvant therapy. If applicable, SAEs must be collected that relate to any later protocol-specified procedure (eg, a follow-up skin biopsy).

The investigator must report any SAE that occurs after these time periods and that is believed to be related to study drug or protocol-specified procedure.

- Medical occurrences that begin before the start of study treatment but after obtaining informed consent will be recorded on the appropriate section of the eCRF section.

- All SAEs will be recorded and reported to Sponsor or designee within 24 hours, as indicated in Appendix 8.
- The investigator will submit any updated SAE data to the sponsor within 24 hours of this being available.

Investigators are not obligated to actively seek AEs or SAEs in former study participants. However, if the investigator learns of any SAE, including a death, at any time after a participant has been discharged from the study, and he/she considers the event reasonably related to the study treatment or study participation, the investigator must promptly notify the sponsor.

The method of evaluating, and assessing causality of AEs and SAEs and the procedures for completing and reporting/transmitting SAE reports are provided in Appendix 8.

9.2.2 Method of Detecting AEs and SAEs

Adverse events can be spontaneously reported or elicited during open-ended questioning, examination, or evaluation of a participant. (In order to prevent reporting bias, participants should not be questioned regarding the specific occurrence of one or more AEs.)

9.2.3 Follow-up of AEs and SAEs

- Nonserious AEs should be followed to resolution or stabilization, or reported as SAEs if they become serious (see Appendix 8)
- Follow-up is also required for nonserious AEs that cause interruption or discontinuation of study treatment and for those present at the end of study treatment as appropriate
- All identified nonserious AEs must be recorded and described on the nonserious AE page of the CRF (paper or electronic). Completion of supplemental CRFs may be requested for AEs and/or laboratory abnormalities that are reported/identified during the course of the study

After the initial AE/SAE report, the investigator is required to proactively follow each participant at subsequent visits/contacts. All SAEs, and non-serious AEs of special interest (as defined in Section 9.2 will be followed until resolution, until the condition stabilizes, until the event is otherwise explained, or until the participant is lost to follow-up (as defined in Section 8.3).

Further information on follow-up procedures is given in Appendix 8.

9.2.4 Regulatory Reporting Requirements for SAEs

- Prompt notification by the investigator to the Sponsor of SAEs is essential so that legal obligations and ethical responsibilities towards the safety of participants and the safety of a product under clinical investigation are met.
- An investigator who receives an investigator safety report describing SAEs or other specific safety information (eg, summary or listing of SAEs) from the Sponsor will file it along with the Investigator's Brochure and will notify the IRB/IEC, if appropriate according to local requirements.

Sponsor or designee will be reporting adverse events to regulatory authorities and ethics committees according to local applicable laws including European Directive 2001/20/EC and FDA Code of Federal Regulations 21 CFR Parts 312 and 320. A SUSAR (Suspected, Unexpected Serious Adverse Reaction) is a subset of SAEs and will be reported to the appropriate regulatory authorities and investigators following local and global guidelines and requirements.

All SAEs must be collected that occur during the screening period and 100 days after the last dose of last dose of neoadjuvant therapy or 90 days after surgery, whichever is longer, and 30 days after the last dose of adjuvant therapy. For participants randomized/assigned to treatment and never treated with study drug, SAEs should be collected for 30 days from the date of randomization.

9.2.5 Pregnancy

If, following initiation of the study treatment, it is subsequently discovered that a participant is pregnant or may have been pregnant at the time of study exposure, including during at least 5 half-lives after product administration, the investigator must immediately notify the BMS Medical Monitor/designee of this event and complete and forward a Pregnancy Surveillance Form to BMS Designee within 24 hours of awareness of the event and in accordance with SAE reporting procedures described in Appendix 8.

In most cases, the study treatment will be permanently discontinued in an appropriate manner (eg, dose tapering if necessary for participant safety). Please call the BMS Medical Monitor within 24 hours of awareness of the pregnancy.

Protocol-required procedures for study discontinuation and follow-up must be performed on the participant.

Follow-up information regarding the course of the pregnancy, including perinatal and neonatal outcome and, where applicable, offspring information must be reported on the Pregnancy Surveillance Form.

Any pregnancy that occurs in a female partner of a male study participant should be reported to Sponsor or designee. In order for Sponsor or designee to collect any pregnancy surveillance information from the female partner, the female partner must sign an informed consent form for disclosure of this information. Information on this pregnancy will be collected on the Pregnancy Surveillance Form.

9.2.6 Laboratory Test Result Abnormalities

The following laboratory test result abnormalities should be captured on the nonserious AE CRF page or SAE Report Form electronic, as appropriate. Paper forms are only intended as a back-up option when the electronic system is not functioning.

- Any laboratory test result that is clinically significant or meets the definition of an SAE
- Any laboratory test result abnormality that required the participant to have study treatment discontinued or interrupted
- Any laboratory test result abnormality that required the participant to receive specific corrective therapy

Approved v 9.0 [redacted]

It is expected that wherever possible, the clinical rather than laboratory term would be used by the reporting investigator (eg, anemia versus low hemoglobin value).

9.2.7 Potential Drug Induced Liver Injury (DILI)

Wherever possible, timely confirmation of initial liver-related laboratory abnormalities should occur prior to the reporting of a potential DILI event. All occurrences of potential DILIs, meeting the defined criteria, must be reported as SAEs (see Section 9.2 and Appendix 8 for reporting details).

Potential drug induced liver injury is defined as:

1) AT (ALT or AST) elevation > 3 times upper limit of normal (ULN)

   AND

2) Total bilirubin > 2 times ULN, without initial findings of cholestasis (elevated serum alkaline phosphatase),

   AND

3) No other immediately apparent possible causes of AT elevation and hyperbilirubinemia, including, but not limited to, viral hepatitis, pre-existing chronic or acute liver disease, or the administration of other drug(s) known to be hepatotoxic.

9.2.8 Other Safety Considerations

Any significant worsening noted during interim or final physical examinations, electrocardiogram, x-ray filming, any other potential safety assessment required or not required by protocol should also be recorded as a nonserious or serious AE, as appropriate, and reported accordingly.

Definition of immune-mediated adverse events (IMAEs)

Immune-mediated AEs are specific events (that include pneumonitis, diarrhea/colitis, hepatitis, nephritis/renal dysfunction, rash, and endocrine [adrenal insufficiency, hypothyroidism/thyroiditis, hyperthyroidism, diabetes mellitus, and hypophysitis]) for which participants received immunosuppressive medication for treatment of the event, with the exception of endocrine events (hypothyroidism/thyroiditis, hyperthyroidism, hypophysitis, diabetes mellitus, adrenal insufficiency), which are included regardless of treatment since these events are often managed without immunosuppression.

Immune-mediated AEs are specific events (or groups of PTs describing specific events) that include pneumonitis, diarrhea/colitis, hepatitis, nephritis/renal dysfunction, rash, endocrine (adrenal insufficiency, hypothyroidism/thyroiditis, hyperthyroidism, diabetes mellitus, and hypophysitis), and other specific events, considered as potential immune-mediated events by investigator, that meet the definition summarized below:

- those occurring within 100 days of the last dose
- regardless of causality

Approved v 9.0 [redacted]

- with no clear alternate etiology based on investigator assessment, or with an immune-mediated component
- treated with immune-modulating medication (Of note, adrenal insufficiency, hypothyroidism/thyroiditis, hyperthyroidism, diabetes mellitus, and hypophysitis are considered IMAEs regardless of immune-modulating medication use, since endocrine drug reactions are often managed without immune-modulating medication).

- Table 9.2.8-1 below provides a summary of the IMAEs category and their respective preferred terms.

Table 9.2.8-1: Preferred Terms Included in Analysis of IMAEs to Support Warnings and Precautions

IMAE Category	Preferred terms included under IMAE Category
Pneumonitis	Pneumonitis, interstitial lung disease
Diarrhea/Colitis	Diarrhea, colitis, enterocolitis
Hepatitis	Hepatotoxicity, hepatitis, hepatitis acute, autoimmune hepatitis, AST increased, ALT increased, bilirubin increased, ALP increased
Adrenal insufficiency	Adrenal insufficiency
Hypothyroidism/Thyroiditis	Hypothyroidism, thyroiditis<br>Thyroiditis acute (collapsed with thyroiditis for frequency), Autoimmune thyroiditis (collapsed with thyroiditis for frequency)
Hyperthyroidism	Hyperthyroidism
Hypophysitis	Hypophysitis
Diabetes mellitus	Diabetes mellitus, diabetic ketoacidosis
Nephritis and renal dysfunction	Nephritis, nephritis allergic, tubulointerstitial nephritis, acute renal failure, renal failure, increased creatinine
Rash	Rash, rash maculopapular

9.2.9 Management Algorithms

Immuno-oncology (I-O) agents are associated with AEs that can differ in severity and duration than AEs caused by other therapeutic classes. Nivolumab and ipilimumab are considered an immuno-oncology agent in this protocol. Early recognition and management of AEs associated with immuno-oncology agents may mitigate severe toxicity. Management Algorithms have been developed to assist investigators in assessing and managing the following groups of AEs:

- Gastrointestinal
- Renal
- Pulmonary
- Hepatic
- Endocrinopathy

Approved v9.0 [redacted]

- Skin
- Neurological
- Myocardial

The above algorithms are found in the Nivolumab Investigator Brochure and Appendix 4.

9.3 Overdose

All occurrences of overdose must be reported as SAEs (see Section 9.2).

An overdose is defined as the accidental or intentional administration of any dose of a product that is considered both excessive and medically important. All occurrences of overdose must be reported as an SAE (see Section 9.2).

9.4 Safety

Planned time points for all safety assessments are listed in the Schedule of Activities.

At screening, a medical history will be obtained to capture relevant underlying conditions. The screening examinations should include weight, height, ECOG Performance Status, blood pressure (BP), heart rate (HR), temperature, and oxygen saturation by pulse oximetry (at rest). Screening assessments and screening laboratory assessments should be performed in accordance with Table 2-1.

Pregnancy tests for WOCBP must be performed within 24 hours prior to the initial administration of study drug.

Participants will be evaluated for safety if they have received any study drug. Toxicity assessments will be continuous during the treatment phase as well as during the first 2 safety (neo-adjuvant) follow-up visits, for 90 days after surgery, and for 30 days after adjuvant therapy. Once participants reach the Survival Follow-up Phase, either in-person visits or documented telephone calls/email correspondence to assess the participant's status are acceptable.

Adverse events and laboratory values will be graded according to the NCI-CTCAE version 4.

The start and stop time of the study therapy infusions and any interruptions or infusion rate reductions should be documented.

Physical examinations are to be performed as clinically indicated. If there are any new or worsening clinically significant changes since the last exam, report changes on the appropriate non-serious or serious adverse event page.

On treatment local laboratory assessments are to be completed within 3 calendar days prior to dosing in accordance with Table 2-2.

On treatment pregnancy tests should be performed as per the schedule in the Schedule of Activity Table.

Additional measures, including non-study required laboratory tests, should be performed as clinically indicated or to comply with local regulations. Laboratory toxicities (eg, suspected drug

Approved v 9.0 [redacted]

induced liver enzyme evaluations) will be monitored during the Neoadjuvant and Adjuvant Follow-up Phases via on site/local labs until all study drug-related toxicities resolve, return to baseline, or are deemed irreversible.

If a participant shows pulmonary-related signs (hypoxia, fever) or symptoms (eg, dyspnea, cough, fever) consistent with possible pulmonary adverse events, the participant should be immediately evaluated to rule out pulmonary toxicity, according to the suspected pulmonary toxicity management algorithm in the nivolumab Investigator Brochure.

Some of the assessments referred to in this section may not be captured as data in the eCRF. They are intended to be used as safety monitoring by the treating physician. Additional testing or assessments may be performed as clinically necessary or where required by institutional or local regulations.

9.4.1 Clinical Safety Laboratory Assessments

Investigators must document their review of each laboratory safety report.

Please refer to the Schedule of Activities in Section 2 for details related to the required laboratory assessment.

9.4.2 Imaging Safety Assessment

Any incidental findings of potential clinical relevance that are not directly associated with the objectives of the protocol should be evaluated and handled by the Study Investigator as per standard medical/clinical judgment.

9.5 Pharmacokinetics

Samples for ipilimumab PK assessment will be collected from participants in Arm A only. Samples for nivolumab PK assessments will be collected from participants in Arm A and Arm C described in Table 9.5-1. Treatment assignments will be released to the bioanalytical laboratory in order to minimize unnecessary analysis of samples. All time points are relative to the start of study drug administration. All on-treatment time points are intended to align with days on which study drug is administered, if dosing occurs on a different day, the PK sampling should be adjusted accordingly. Further details of sample collection, processing, and shipment will be provided in the laboratory procedures manual. PK samples will be analyzed for nivolumab and ipilimumab by a validated ligand binding assay.

Table 9.5-1: Pharmacokinetic (PK) Sample Collections for Nivolumab and Ipilimumab - Arm A and Arm C

Study Day 1 Cycle = 2 weeks for Arm A and 1 Cycle = 3 weeks for Arm C	Event (Relative to Dosing) Hour	Time (Relative to Dosing) Hour: Min	Pharmacokinetic Blood Sample for Nivolumab	Pharmacokinetic Blood Sample for Ipilimumab[a]
C1D1	0.5 (EOI) [b]	00:30	X	X

Approved v9.0 [redacted]

Table 9.5-1: Pharmacokinetic (PK) Sample Collections for Nivolumab and Ipilimumab - Arm A and Arm C

Study Day 1 Cycle = 2 weeks for Arm A and 1 Cycle = 3 weeks for Arm C	Event (Relative to Dosing) Hour	Time (Relative to Dosing) Hour: Min	Pharmacokinetic Blood Sample for Nivolumab	Pharmacokinetic Blood Sample for Ipilimumab[a]
C2D1	Predose[c]	00:00	X	X
C3D1	Predose[c]	00:00	X	X

[a] PK blood samples for ipilimumab were be collected from Arm A participants only.

[b] EOI: End of Infusion. This sample should be taken immediately (ie, within 5 minutes) prior to stopping ipilimumab infusion in Arm A or nivolumab infusion in Arm C. If the end of infusion is delayed to beyond the nominal infusion duration, the collection of this sample should also be delayed accordingly. EOI samples may not be collected from the same IV access as drug was administered.

[c] Predose sample should be collected just before the administration of nivolumab (preferably within 30 minutes). If it is known that a dose is going to be delayed, then the predose sample should be collected just prior to the delayed dose. However, if a predose sample is collected, but the dose is subsequently delayed, an additional predose sample should not be collected.

9.6 Pharmacodynamics

Pharmacodynamic parameters are not evaluated in this study.

9.7 Pharmacogenomics

Not applicable.

9.8 Biomarkers

9.8.1 Additional Research Collection

Additional research retention are mandatory for all study participants, except where prohibited by local laws or regulations, ethics committees, institutional requirements, or where a waiver is provided by BMS or their designee. Where one or more of these exceptions occurs, participation in the additional research should be encouraged, but will not be a condition of overall study participation. Study participants may opt out of the additional research.

This protocol will include residual sample storage for additional research (AR).

This retention for additional research is intended to expand the translational R&D capability at Bristol-Myers Squibb Company and will support as yet undefined research aims that will advance our understanding of disease and options for treatment. It may also be used to support health authority requests for analysis, and advancement of pharmacodiagnostic development to better target drugs to the right participants. This may also include genetic/genomic exploration aimed at exploring disease pathways, progression and response to treatment etc.

All requests for access to samples or data for additional research will be vetted through a diverse committee of the study sponsor's senior leaders in Research and Development to ensure the research supports appropriate and well-defined scientific research activities.

[redacted]

Samples kept for future research will be stored at the BMS Biorepository [redacted] or an independent, BMS-approved storage vendor. The manager of these samples will ensure they are properly used throughout their usable life and will destroy the samples at the end of the scheduled storage period, no longer than fifteen (15) years after the end of the study or the maximum allowed by applicable law. Transfers of samples by research sponsor to third parties will be subject to the recipient's agreement to establish similar storage procedures.

Samples will be stored in a coded fashion; no researcher will have access to the key, which is securely held by the Investigator at the clinical site, so that there is no direct ability for a researcher to connect a sample to a specific individual.

Further details of sample collection and processing will be provided to the site in the procedure manual.

[redacted]

[redacted]

9.8.1.1 Tumor Tissue Specimens

Archival (slides [blocks/slides] ≤ 3 months) FFPE tumor tissue collected at initial diagnosis is required for study entry. Samples will be sent to a third party to stain and score for PD-L1 expression using the PD-L1 IHC 28-8 pharmDx kit (Dako). Stained tissue samples will be assessed by a pathologist at a central lab identified by the Sponsor and scored as PD-L1 expressing if membrane staining is observed in ≥ 1% tumor cells among a minimum of 100 evaluable tumor cells. If an archived specimen is not available, a fresh tumor biopsy will be collected. Additional tumor will be collected during surgical resection, which will be after 3 cycles of treatment in each arm. A third (optional) biopsy will be collected at the time of disease progression. The third biopsy will not be collected in China.

Collected tumors may be further analyzed by additional modalities [redacted]

Approved v 9.0 [redacted]

[redacted]

[redacted]

9.8.2 Safety Analyses

Endpoint	Statistical Analysis Methods
Exploratory	Proportion of delayed or canceled surgery, duration of surgery, length of hospital stay, surgical approach, incidence of AE/SAE associated with surgery, including pneumonitis, ARDS, re-admission to the Intensive Care Unity, atrial fibrillation or other supraventricular tachycardia up to 90 days after surgery will be summarized by treatment group using descriptive statistics.<br>The safety and tolerability objective will be measured by the incidence of adverse events, serious adverse events, deaths, and laboratory abnormalities.<br>The safety analysis will be performed in all treated participants. The rate of treatment-related selected AEs and SAEs in each treatment arm will be summarized. Descriptive statistics of safety will be presented using National Cancer Institute (NCI) Common Terminology Criteria for Adverse Events (CTCAE) version 4.0 by treatment arm. All treatment-emergent AEs, drug-related AEs, SAEs and drug-related SAEs will be tabulated using worst grade per NCI CTCAE v 4.0 criteria by system organ class and preferred term. On-study lab parameters including hematology, chemistry, liver function, thyroid function and renal function will be summarized using worst grade per NCI CTCAE v 4.0 criteria.

Approved v 9.0 [redacted]

9.9 Health Economics OR Medical Resource Utilization and Health Economics

Health Economics/Medical Resource Utilization and Health Economics parameters will not be evaluated in this study.

9.10 Other Assessments

9.10.1 Diagnostic/Pre-surgical Evaluation and Surgical Guidelines:

A participant is considered functionally operable if the following criteria are met:

- ECOG Performance Status ≤ 1
- Absence of major associated pathologies that increase the surgery risk to an unacceptable level
- Pulmonary function capacity (eg, FVC, FEV1, TLC, FRC, and DLco) capable of tolerating the proposed lung resection according to the surgeon
- Absence of locally advanced, unresectable, or metastatic disease (stage IV) in the pre-treatment assessment

All participants enrolled on this study must be surgical candidates with clinical stage IB (≥ 4 cm), II or resectable IIIA (N2) NSCLC. Participants will have undergone radiographic evaluation (PET/CT) indicating no evidence of distant disease and no evidence of unresectable loco-regional tumor extension (EBUS, mediastinoscopy, or thoracoscopy) prior to randomization. Any further preoperative testing that is recommended by the surgeon or anesthesiologist will be performed as part of standard of care. Surgery for participants enrolled on this protocol will be according to generally accepted standards of care. Operative approach (VATS vs open) will be determined by the surgeon.

Accepted types of resection (with negative margins) will consist of lobectomy, sleeve lobectomy, bi-lobectomy, or pneumonectomy. Resections by segmentectomy or wedge resection will not be accepted. If mediastinoscopy was not performed preoperatively, it is expected that, at a minimum, mediastinal lymph node systematic sampling and evaluation will have occurred, though complete mediastinal lymph node dissection (MLND) is preferred. It is recommended that participants have at least 3 mediastinal and hilar lymph node stations sampled and evaluated during surgery. Systematic sampling is defined as removal of at least 1 representative lymph node at specified levels. MLND entails resection of all lymph nodes at those same levels.

The following sampling or MLND locations are required:

- For right-sided tumor: Levels 4, 7, 9R, 10R and 11 R
- For left-sided tumor: Levels 5 and/or 6; 7, 9L, 10L, and 11L

If there is clear documentation in the operative report or in a separately submitted addendum by the surgeon of exploration of the required lymph node areas, the participant will be considered eligible if no lymph nodes are found in those areas.

Approved v 9.0 [redacted]

Participants should have a PET/CT scan with contrast of the base of the skull through the upper thigh performed within the 14 days prior to planned surgery to assess for clinical response. A separate CT with contrast of the chest, abdomen, and other suspected areas (as well as the PET/CT) is required if the CT component of a PET/CT is not of sufficient diagnostic quality of RECIST 1.1 measurements. Surgery should be performed within 6 weeks of completing up to 3 cycles (last dose) of neoadjuvant treatment.

9.10.2 Post-surgical Evaluation:

The first tumor assessment should occur 12 weeks (±7 days) after definitive surgery per RECIST 1.1. Afterwards, radiographic assessment will be performed every 12 weeks (± 7 days) for 2 years) following surgery, every 6 months (± 7 days) for 3 years, and then every year (±7 days) for 5 years or until recurrence or progression of disease is documented.

10 STATISTICAL CONSIDERATIONS

10.1 Sample Size Determination

The original study design (before Revised protocol 01) had two arms, with participants randomized in a 1:1 ratio to either neoadjuvant nivolumab plus ipilimumab or platinum doublet chemotherapy arm. Revised protocol 02 added a new, neoadjuvant nivolumab plus platinum doublet chemotherapy arm. When the third arm opens and as each site receives IRB/EC approval of revised protocol 02, the IRT will switch to a 1:1:1 randomization at the respective site. Starting from that point on, the site will only enroll under revised protocol 02.

Revised protocol 03 withholds randomization into the arm of neoadjuvant nivolumab plus ipilimumab but continues randomizing eligible participants into either neoadjuvant nivolumab plus platinum doublet chemotherapy arm or platinum doublet chemotherapy arm in a 1:1 ratio. Approximately 350 participants (175 participants per arm) will be randomized between 2 arms neoadjuvant nivolumab plus platinum doublet chemotherapy or platinum doublet chemotherapy from 1:1:1 randomization in revised protocol 02 and 1:1 randomization in revised protocol 03. Participants already randomized in the original 2-arm part (neoadjuvant nivolumab plus ipilimumab vs neoadjuvant chemotherapy) and in the arm of neoadjuvant nivolumab plus ipilimumab in 3-arm part defined by revised protocol 02 will remain in trial and continue scheduled trial procedures. It is expected to have around 70 participants randomized in the original 2-arm part and approximately another 75 participants randomized in the arm of neoadjuvant nivolumab plus ipilimumab in the 3-arm part. It is estimated that there will be a total of approximately 500 participants on the study.

Starting from 1:1:1 randomization, approximately 350 participants will be randomized to the 2 arms neoadjuvant nivolumab plus platinum doublet chemotherapy or platinum doublet chemotherapy in a 1:1 ratio. The sample size of the study is calculated based on the primary endpoint of EFS and accounts for the multiple primary endpoints comparisons: pCR (per BIPR) and EFS (per BICR) with an initial alpha allocation of 0.01 and 0.04 respectively. Formal analyses of pCR and EFS may be conducted at different timepoints. The fallback method will be used, ie, if the pCR comparison between Arm C and Arm B is statistically significant, then 0.01 alpha allocated to pCR will be passed to the EFS comparison for Arm C vs Arm B and the EFS

Approved v 9.0 [redacted]

comparison will be conducted at the alpha = 0.05 level. If the pCR comparison between Arm C and Arm B is not statistically significant, then the EFS comparison for Arm C vs Arm B will be conducted at the alpha = 0.04 level. In addition, if EFS is significant, OS will be tested hierarchically at the same level as EFS.

10.1.1 Pathologic Complete Response (pCR)

The primary analysis of pCR will be performed after 350 randomized participants in neoadjuvant nivolumab plus platinum doublet chemotherapy and platinum doublet chemotherapy (from start of 1:1:1 randomization) have an opportunity for surgery.

Assuming an accrual rate of 10 participants (all comers) a month between Arms B and C during 1:1:1 randomization (about 10 months), and 15 participants per month during 1:1 randomization, it is anticipated that the 350 participants will be randomized in approximately 27 months. The pCR endpoint is expected to be analyzed after about 30 months from start of 1:1:1 randomization.

The sample size of 350 participants is based on the EFS endpoint. With this sample size, assuming pCR rate of 10% on Arm B chemotherapy and 30% on Arm C nivolumab plus chemotherapy, respectively, the 350 participants will provide more than 90% power to detect an odds ratio of 3.857 with a 2-sided type I error of 1%.

It is estimated that there will be about 110 subjects randomized to Arm A neoadjuvant nivolumab plus ipilimumab before revised protocol 03 is implemented. Assuming true pCR rate is 15% on this arm, there is 95% probability that the lower bound of 95% exact confidence interval of pCR is above 5%.

10.1.2 Event Free Survival (EFS)

For the formal comparison of EFS as assessed by BICR for nivolumab plus platinum doublet chemotherapy (Arm C) vs platinum doublet chemotherapy (Arm B), only participants randomized from 1:1:1 randomization in revised protocol 02 and 1:1 randomization in revised protocol 03 will be counted.

The power details are provided below using alpha = 0.05 (0.01 alpha from the pCR endpoint fallback to the EFS comparison), considering this protocol amendment (protamend07) occurs after the statistically significant pCR final analysis but before any analysis of EFS or OS. In addition, the accrual reflects 358 participants who were actually concurrently randomized in Arms B and C.

A total of 185 events ensure that an overall 2-sided 5% significance level sequential test procedure with 2 interim analyses occurring after 148 events (80% of events required for final analysis) and 167 events (90% of events required for final analysis) in 358 randomized participants will have 82% power assuming an HR of 0.65 between the 2 arms. Considering a piecewise exponential distribution with control hazard rates of 0.028 before 20 months, 0.017 between 20 months and 40 months, 0.014 between 40 and 60 months, and 0.008 after 60 months, and a dropout rate of approximately 20%, it is anticipated the EFS analyses will take place at approximately 48, 58, and 73 months from the start of 1:1:1 randomization. The trigger of the first interim analysis is event driven. The second interim analysis will take place when 167 events are observed [redacted]. The final analysis will take place

Approved v 1.0 [redacted]

when approximately 185 events are observed [redacted]. The stopping boundaries at the interim and final EFS analyses will be derived based on the exact number of events using Lan-DeMets alpha spending function with O'Brien-Fleming boundaries. If the interim analyses of EFS are performed at exactly 148 and 167 events, the nominal significance level for EFS superiority will be 0.024 and 0.030, respectively. The nominal significance level for the final look of EFS after 185 events would then be 0.038.

[redacted]

Table 10.1.3-1 summarizes the key parameters of the sample size justification in the randomized participants.

10.1.3 Power Consideration for Overall Survival (OS)

The secondary endpoint OS will be tested hierarchically after EFS with the same overall alpha as for the EFS comparison (two-sided 4% if the pCR comparison is not significant or 5% if the pCR comparison is significant).

For the formal comparison of OS for nivolumab plus platinum doublet chemotherapy (Arm C) vs platinum doublet chemotherapy (Arm B), only participants concurrently randomized from 1:1:1 randomization in revised protocol 02 and 1:1 randomization in revised protocol 03 will be included. Three interim analyses for OS are planned to occur at the time of EFS interim and final analyses, only if EFS is significant.

The power details are provided below using alpha = 0.05 (0.01 alpha from the pCR endpoint fallback to the EFS comparison, then hierarchically on the OS comparison), considering this protocol amendment (protamend07) occurs after the readout of the statistically significant pCR final analysis but before any analysis of EFS or OS. In addition, they reflect the 358 participants that were actually concurrently randomized in Arms B and C:

[redacted]

[redacted]

Table 10.1.3-1: Power Calculation for EFS and OS

	EFS<br>Arm C vs Arm B	OS<br>Arm C vs Arm B
Accrual	Actual accrual<br>25 months	Actual accrual<br>25 months
Power	82%	[redacted]
Two-sided alpha	0.05	0.05
Hypothesized median control vs exp (months)	28 vs 52*	[redacted]
Hypothesized hazard ratio	0.65	
Sample size for concurrent comparison	358	358
First interim analysis for EFS (EFS IA1) and OS (OS IA1)	148 events<br>Alpha boundary: 0.024	Triggered by EFS IA1<br>[redacted]
Second interim analysis for EFS (EFS IA2) and OS (OS IA2)	167 events [redacted]<br>Alpha boundary: 0.030	• If EFS IA1 not significant: triggered by EFS IA2.<br>[redacted]
Final EFS (EFS FA) and third OS (OS IA3) interim analysis	185 events [redacted]<br>Alpha boundary: 0.038	• If EFS IA2 not significant: triggered by EFS FA.<br>[redacted]
Final OS analysis (OS FA)	-	[redacted]

*Calculated from piecewise exponential distribution

EFS, event free survival; FA, final analysis; IA, interim analysis; OS, overall survival

Approved v 9.0 [redacted]

10.1.4 Analyses Timing Projections

The pCR analysis occurred with a database lock on 16-Sep-2020.

Considering the actual enrollment, it will take:

- Approximately 48 months when 148 events on Arms B and C (after start of 1:1:1 randomization) are observed for the first interim analysis (EFS IA1, OS IA1). This is about 54 months from first patient first visit (FPFV) of the study. This analysis will be triggered by the number of EFS events.
- Approximately 58 months when 167 EFS events on Arms B and C (after start of 1:1:1 randomization) are observed for the second interim analysis (EFS IA2, OS IA2). This is approximately 64 months from FPFV. [redacted]
- Approximately 73 months when 185 EFS events on Arms B and C (after start of 1:1:1 randomization) are observed for the final EFS analysis (EFS FA, OS IA3). This is about 79 months from FPFV of the study. [redacted]

[redacted]

10.2 Populations for Analyses

Population	Description
All enrolled participants	All participants who signed an informed consent form and were registered into the IRT
All randomized participants	All participants who were randomized to any treatment arm in the study. This is the primary dataset for analyses of study conduct, study population and efficacy.
All concurrently randomized participants in Arms B and C	All randomized participants on Arms B and C after 1:1:1 randomization. Analysis of demography, protocol deviations, baseline characteristics, and primary efficacy analyses will be performed for this population.
All treated participants	All participants who received any dose of study medication in neoadjuvant setting. This is the primary dataset for drug exposure and safety analysis.
PK participants	All participants with available serum time-concentration data from randomized participants dosed in Arm A and Arm C

Approved v 1.0 [redacted]

10.3 Statistical Analyses

10.3.1 Efficacy Analyses

Endpoint	Statistical Analysis Methods
Primary	The fallback method will be used for the hypothesis testing of the two primary endpoints pCR and EFS. Initially, the type 1 error of 0.05 will be allocated as follows: 0.01 will be allocated to the pCR comparison between Arm C and Arm B, and 0.04 will be allocated to the EFS comparison between Arm C and Arm B. If the pCR comparison between Arm C and Arm B is statistically significant, then 0.01 alpha will be passed to the EFS comparison for Arm C vs Arm B and the test comparison will be conducted at the alpha = 0.05 level.<br>pCR analysis will be based on all concurrently randomized participants in Arms B and C analysis population. pCR rate will be computed in each treatment group along with the exact 95% CI using Clopper-Pearson method. For pCR rate comparison between arms C vs B, the difference will be tested via the Cochran Mantel-Haenszel (CMH) test using a 2-sided, 1% alpha level. the odds ratios stratified by PD-L1, disease stage (IB/II vs IIIA) and gender (Mantel-Haenszel estimator) between Arms C and B will be provided along with the 99% CI. In addition, an estimate of the difference in pCR rates and corresponding 99% CI will be calculated using CMH methodology and adjusted by stratification factors (PD-L1, disease stage (IB/II vs IIIA) and gender).<br>pCR rate will also be computed for subjects randomized to Arm A neoadjuvant nivolumab plus ipilimumab along with the exact 95% CI using Clopper-Pearson method.<br>EFS is defined as time from randomization to any of the following events: any documented progression of disease precluding surgery, progression or recurrence disease (based on BICR assessment per RECIST 1.1) after surgery, or death due to any cause. Participants who don't undergo surgery for reason other than progression will be considered to have an event at RECIST 1.1 progression (based on BICR) or death. Participants who die without a reported progression/disease recurrence will be considered to have experienced an event on the date of their death. Participants who did not report progression/recurrence of disease or die will be censored on the date of their last evaluable tumor assessment. Participants who did not have any on-study tumor assessments and did not die will be censored on the date they were randomized. Participants who started any subsequent anti-cancer therapy outside of the protocol-specified adjuvant therapy without a prior reported progression/recurrence will be censored at the last evaluable tumor assessment prior to initiation of the subsequent anti-cancer therapy.<br>If the pCR analysis comparing Arms C vs B is statistically significant, a two-sided alpha of 0.05 will be used for the comparison of EFS in Arms C vs B. If the pCR analysis comparing Arms C vs B is not statistically significant, a two-sided alpha of 0.04 will be used for the comparison of EFS in Arms C vs B. EFS among the all concurrently randomized participants in Arms B and C analysis population will be tested. The distribution of EFS will be compared between Arm C vs Arm B via a 2-sided, log rank test stratified by the randomization stratification factor (ie, PD-L1, disease stage [IB/II vs IIIA] and gender).The hazard ratio and the corresponding (1-adjusted alpha) confidence interval will be estimated for Arm C vs Arm B in a stratified Cox proportional hazards model using the randomized arm as a single covariate. The EFS curves for each randomized arm will be estimated using the Kaplan-Meier (KM) product-limit method using a log-log transformation. In addition, EFS rates at 1, 2, 3, and 4 years will be estimated using KM estimates on the EFS curve for each randomized arm provided a minimum follow-up is longer than the time point to generate the rate. Associated 2-sided 95% CIs will be calculated using the Greenwood formula (using log-log transformation).<br>Sensitivity analysis on EFS will also be performed, and details will be included in statistical analysis plan.
Secondary	At the time of pCR analysis, MPR rate will be computed in each treatment group along with the exact 95% CI using Clopper-Pearson method. An estimate of the difference in MPR rates between Arm C and Arm B and corresponding 95% CI will be calculated using CMH methodology and adjusted by stratification factors (PD-L1, disease stage (IB/II vs IIIA) and gender).

Approved v9.0 [redacted]

Endpoint	Statistical Analysis Methods
	OS curves, OS medians with 95% CIs, and OS rates at 1, 2, 3, and 4 years with 95% CIs will be estimated using Kaplan-Meier methodology. HR and corresponding 2-sided 95% CI will be estimated for Arm C vs Arm B using a Cox proportional hazards model with treatment group as a single covariate, stratified by randomization stratification factors. If tested, the distribution of OS will be compared between Arm C vs Arm B in the all concurrently randomized participants in Arms B and C population via a 2-sided, log rank test stratified by the randomization stratification factors.<br><br>Time to death or distant metastases (TTDM) is defined as the time between the date of randomization and the first date of distant metastasis or the date of death in the absence of distant metastasis. Distant metastasis is defined as any new lesion that is outside of the thorax using BICR according to RECIST 1.1. Participants who die without a reported distant metastasis will be considered to have experienced an event on the date of their death. Patients who have not developed distant metastasis or died at the time of analysis will be censored on the date of their last evaluable tumor assessment. Participants who did not have baseline scan, or participants who did not have any on-study tumor assessments and did not die, will be censored on the date they were randomized.<br><br>TTDM curves, TTDM medians with 95% CIs, and TTDM rates at 1, 2, 3, and 4 years with 95% CIs will be estimated using Kaplan-Meier methodology. HR and corresponding 2-sided 95% CI will be estimated for Arm C vs Arm B using a Cox proportional hazards model with treatment group as a single covariate, stratified by randomization stratification factors. There will be no formal comparison of TTDM.
Exploratory	Will be described in the statistical analysis plan finalized before database lock

10.3.2 Other Analyses

Biomarker exploratory analyses will be described in the statistical analysis plan finalized before database lock. The population pharmacokinetics analysis will be presented separately from the main clinical study report.

The EQ-5D-3L questionnaire completion rate, defined as the proportion of questionnaires actually received out of the expected number, will be calculated and summarized at each assessment point. Summary statistics (ie, N, mean with SD and 95% CI, median, first and third quartiles, minimum, maximum) for the EQ-5D-3L utility index and VAS scores, as well as their corresponding changes from baseline, will be tabulated by treatment and planned time point. In the base case, index scores will be derived using the UK weighting algorithm. In addition, the proportion of participants reporting no, moderate, or severe problems in each of the 5 EQ-5D-3L dimensions will be summarized by treatment group at each time point. Proportions will be based on the number of participants assessed at assessment time point. A by-participant listing of the level of problems in each dimension, corresponding EQ-5D-3L health state (ie, 5-digit vector), EQ-5D-3L index score, and EQ-5D VAS score will be provided.

10.3.3 Interim Analyses

There is no interim analysis for the pCR endpoint.

Approved v9.0 [redacted]

Two formal interim analyses for EFS are planned after approximately 148 and 167 events have been observed on Arms B and C, after the start of 1:1:1 randomization, which is projected to occur approximately 48 and 58 months after start of 1:1:1 randomization. [redacted] The formal comparisons of EFS will allow for early stopping for superiority. An independent statistician external to BMS will perform the analysis. If the study continues beyond the second interim analysis, the final analysis will be conducted after 185 EFS events have been observed on Arms B and C from the start of 1:1:1 randomization. The stopping boundaries at the interim and final analyses will be based on the actual number of EFS events at the time of the analysis using Lan-DeMets alpha spending function with O'Brien-Fleming boundaries.

[redacted]

11 REFERENCES

1 Mountain, CA. Regional lymph node classification for lung cancer staging. Chest. 1997: 1718-23.

2 Goldstraw P, Crowley J, Chansky K, et al for the International Association for the Study of Lung Cancer International Staging Committee. The IASLC Lung Cancer Staging Project: proposals for the revision of the TNM stage groupings in the forthcoming (seventh) edition of the TNM Classification of malignant tumours. J Thorac Onco. 2007; 2(8):706-14.

3 Detterbeck FC, Boffa DJ, Tanoue LT. The new lung cancer staging system. Chest. 2009; 136: 260-71.

4 NSCLC Meta-analyses Collaborative Group. Adjuvant chemotherapy, with or without postoperative radiotherapy, in operable non-small-cell lung cancer: two meta-analyses of individual patient data. Lancet. 2010 Apr 10;375(9722):1267-77.

5 Siegel RL, Miller KD, Jemal A. Cancer statistics, 2015. A Cancer J Clin. 2015; 65(1):5-29.

6 Arriagada R, Bergman B, Dunant A, et al. Cisplatin-based adjuvant chemotherapy in patients with completely resected non-small-cell lung cancer. N Engl J Med, 2004, 351-60.

7 Douillard JY, R. R. Adjuvant vinorelbine plus cisplatin versus observation in patients with completely resected stage IB-IIIA non-small-cell lung cancer (Adjuvant Navelbine International Trialist Association [ANITA]): a randomised controlled trial. Lancet Oncology. 2006;7: 719-27.

8 Strauss GM, Herndon JE 2nd, Maddaus MA, et al. Adjuvant paclitaxel plus carboplatin compared with observation in stage IB non-small cell lung cancer: CALGB 9633 with the Cancer and Leukemia Group B, Radiation Therapy Oncology Group and Northern Central Cancer Treatment Study Groups. Journal of Clinical Oncology. 2008;26: 5043-51.

9 Winton T, L. R. Vinorelbine plus cisplatin vs. observation in resected non-small-cell lung cancer. N Engl J Med. 2005;352: 2589-97.

10 Burdett S, Stewart LA, Rydzewska L. A systematic review and meta-analysis of the literature: chemotherapy and surgery versus surgery alone in non-small cell lung cancer. J Thorac Oncol. 2006 Sep;1(7):611-21.

11 Brahmer J, Reckamp KL, Baas P, et al. Nivolumab versus Docetaxel in Advanced Squamous Cell Non-small Cell Lung Cancer. N Engl J Med 2015;373:123-35.

12 Borghaei H, Paz-Ares L, Horn L, et al. Nivolumab versus Docetaxel in Advanced Nonsquamous Non-small Cell Lung Cancer. N Engl J Med 2015;373:1627-39.

13 Hellmann, MD, Gettinger, SN, Goldman, JW, et al. J Clin Oncol 34, 2016 (suppl; abstr 3001).

Approved v 1.0 [redacted]

14 Goldman JW, Antonia SJ, Gettinger SN, et al. Nivolumab Plus Ipilimumab as First-Line Treatment for Advanced NSCLC: 2-Year Overall Survival and Long-Term Outcomes From CheckMate 012. ASCO, 53rd Annual Meeting, June 2–6, 2017, Chicago, IL

15 Hellmann MD, Ciuleanu TE, Pluzanski A, et al. N Engl J Med. 2018;378:2093-2104.

16 Antonia SJ, Brahmer JR, Gettinger S et al. Nivolumab (Anti-PD-1; BMS-936558, ONO-4538) in Combination With Platinum-Based Doublet Chemotherapy (PT-DC) in Advanced Non-Small Cell Lung Cancer (NSCLC). Presented at CMSTO 2014 Annual Meeting in Chicago, IL

17 Borghaei H, Hellmann MD, Paz-Ares L et al. Nivolumab + ipilimumab, nivolumab + chemotherapy, and chemotherapy in chemo-naive patients with advanced non-small-cell lung cancer and <1% tumor PD-L1 expression: results from CheckMate 227. ASCO 2018. Abstr 9001

18 Sorenson S, Glimelius B, Nygren P. A Systematic Overview of Chemotherapy effects in non-small cell lung cancer. Act Oncologica. 2001; 40 (2-3): 327-339.

19 Pardoll D. Does the immune system see tumors as foreign or self? Annu Rev Immunol. 2003;21:807-39.

20 Zitvogel L, Tesniere A, Kroemer G. Cancer despite immunosurveillance: immunoselection and immunosubversion. Nat Rev Immunol. 2006;6:715-27.

21 Dunn GP, Bruce AT, Ikeda H, et al. Cancer immunoediting: from immunosurveillance to tumor escape. Nat Immunol. 2002;3:991-8.

22 Greenwald RJ, Freeman GH, Sharpe AH. The B7 family revisited. Annu Rev Immunol. 2004;23:515-48.

23 Freeman GJ, Long AJ, Iwai Y, et al. Engagement of the PD-1 immunoinhibitory receptor by a novel B7 family member leads to negative regulation of lymphocyte activation. J Exp Med 2000; 192(7): 1027-34.

24 Sharpe AH, Wherry EJ, Ahmed R, et al. The function of programmed cell death 1 and its ligands in regulating autoimmunity and infection. Nature Immunol. 2007;8:237-45.

25 Wolchok JD, Hoos A, O'Day S, et al. Guidelines for the evaluation of immune therapy activity in solid tumors: immune-related response criteria. Clin Cancer Res. 2009;15:7412-20.

26 Postow MA, Harding J, Wolchok JD. Targeting immune checkpoints: Releasing the restraints on anti-tumor immunity for patients with melanoma. Cancer J 2012; 18:153-159.

27 van der Most RG, Currie AJ, Mahendran S, et al. Tumor eradication after cyclophosphamide depends on concurrent depletion of regulatory T cells: A role for cycling TNFR2-expressing effector-suppressor T cells in limiting effective chemotherapy. Cancer Immunol Immunother. 2008;58:1219–28.

28 Zitvogel L, Galluzzi L, Smyth MJ, Kroemer G. Mechanism of action of conventional and targeted anticancer therapies: reinstating immunosurveillance. Immunity. 2013;39:74–88.

Approved v 1.0 [redacted]

29 Harris SJ, Brown J, Lopez J, and Yap TA. Immuno-oncology combinations: raising the tail of the survival curve Cancer Biol Med. 2016 Jun; 13(2): 171–193.

30 First-Line Treatment for Advanced NSCLC: 2-Year Overall Survival and Long-Term Outcomes From CheckMate 012. ASCO, 53rd Annual Meeting, June 2–6, 2017, Chicago, IL

31 Xu XL, D. L. Neoadjuvant chemoradiotherapy or chemotherapy followed by surgery is superior to that followed by definitive chemoradiation or radiotherapy in stage IIIA (N2) nonsmall-cell lung cancer: a meta-analysis and system review. Onco Targets Ther.2016:9: 845-53.

32 Gilligan D, Nicolson M, Smith I, et al. Preoperative chemotherapy in patients with resectable non-small cell lung cancer: results of the results of the MRC 22 ? NVALT 2 / EORTC 08012 multicentre randomised trial and update of systematic review. Lancet. 2007;369:1929.

33 Depierre A, Milleron B, Moro-Sibilot D, et al. Preoperative chemotherapy followed by surgery compared with primary surgery in resectable stage I (except T1N0), II, and IIIa non-small-cell lung cancer. J Clin Oncol. 2002;20:247-253.

34 Scagliotti GV, Pastorino U, Vansteenkiste JF, et al. Randomized phase III study of surgery alone or surgery plus preoperative cisplatin and gemcitabine in stages IB to IIIA non-small-cell lung cancer. J Clin Oncol. 2012:30(2),172-8.

35 Pisters K. S9900: A phase III trial of surgery alone or surgery plus preoperative (preop) paclitaxel/carboplatin (PC) chemotherapy in early stage non-small cell lung cancer (NSCLC): Preliminary results. Proc ASCO 2005;(Abstract 7012), 23.

36 Scagliotti G. et al. A phase III randomized study of surgery alone or surgery plus preoperative gemcitabine-cisplatin in early-stage non-small cell lung cancer (NSCLC): Follow-up data of C.h.E.S.T. Proc ASCO 2009, abstract 7508.

37 Felip E, Massuti B, Alonso G, et al. Surgery alone, pre-operative paclitaxel/carboplatin chemotherapy followed by surgery, or surgery followed by adjuvant PC chemotherapy in early stage non-small cell lung cancer. Results of the NATCH multicenter randomized phase III trial. Proc ASCO 2009 (Abstract 7500).

38 Forde PM, Chaft JE, Smith KN, et al. Neoadjuvant PD-1 blockade in resectable lung cancer. N Engl J Med. 2018; 378:1976-86.

39 Provencio M, Nadal E, Cobo-Dols M et al. Neo-adjuvant chemo/immunotherapy for the treatment of stage IIIA resectable non-small-cell lung cancer (NSCLC): a phase II multicenter exploratory study-NADIM study-SLCG. 2018 ASCO Abstr.8521

40 Curran MA, Montalvo W, Yagita H, et al. PD-1 and CTLA-4 combination blockade expands infiltrating T cells and reduces regulatory T and myeloid cells within B16 melanoma tumors. PNAS 2010; 107: 4275-80.

41 Postow MA, Chesney J, Pavlick AC, et al. Nivolumab and ipilimumab versus ipilimumab in untreated melanoma. N Engl J Med 2015; 372:2006-2017.

Approved v 9.0 [redacted]

42 Larkin J, Chiarion-Sileni V, Gonzalez R, et al. Combined Nivolumab and Ipilimumab or Monotherapy in Untreated Melanoma. New Eng J Med. 2015;373(1):23-34.

43 Calvo, et al., presented at the 18th ECCO—40th ESMO European Cancer Congress; September 25-29, 2015; Vienna, Austria.

44 Rizvi NA, Gettinger S, Goldman J et al. Safety and Efficacy of First-line Nivolumab (Anti-programmed Death-1 [PD-1]) and Ipilimumab in Non-small Cell Lung Cancer (NSCLC). Presented at WCLC 2015 Annual Meeting in Denver, CO

45 Goldman JW, Antonia SJ, Gettinger SN, et al. Nivolumab Plus Ipilimumab as First-Line Treatment for Advanced NSCLC: 2-Year Overall Survival and Long-Term Outcomes From CheckMate 012. ASCO, 53rd Annual Meeting, June 2–6, 2017, Chicago, IL

46 Gettinger S, et al. Oral presentation at the IASLC 17th World Conference on Lung Cancer; December 4–7, 2016; Vienna, Austria. Abstract OA03.01.

47 Hellmann MD, Chaft JE, William Jr WN, for The University of Texas MD Anderson Lung Cancer Collaborative Group. Pathological response after neoadjuvant chemotherapy in resectable non-small-cell lung cancers: proposal for the use of major pathological response as a surrogate endpoint. Lancet Oncol 2014; 15: e42–50.

48 Betticher DC, Hsu Schmitz S-F, Totsch M. Prognostic factors affecting long-term outcomes in patients with resected stage IIIA pN2 non-small-cell lung cancer: 5-year follow-up of a phase II study. British Journal of Cancer. 2006;94:1099 – 1106.

49 Pataer A, Kalhor N, Correa AM, et al for the University of Texas M. D. Anderson Lung Cancer Collaborative Research Group. Histopathologic response criteria predict survival of patients with resected lung cancer after neoadjuvant chemotherapy. J Thorac Oncol. 2012; 7(5):825-32.

50 NSCLC Meta-analyses Collaborative Group. Adjuvant chemotherapy, with or without postoperative radiotherapy, in operable non-small-cell lung cancer: two meta-analyses of individual patient data. Lancet. 2010;375:267-77.

51 Wakelee H, Chhatwani L. Adjuvant Chemotherapy for Resected Non-Small Cell Lung Cancer. Seminars in Thoracic and Cardiovascular Surgery. 2008 20:198-203.

52 Felip E, Rosell R, Maestre JA, et al. Preoperative chemotherapy plus surgery versus surgery plus adjuvant chemotherapy versus surgery alone in early-stage non-small-cell lung cancer. J Clin Oncol 2010; 28: 3138–45.

53 Arriagada R, Bergman B, Dunant A, et al: Cisplatin-based adjuvant chemotherapy in patients with completely resected non-small-cell lung cancer: The International Adjuvant Lung Cancer Trial Collaborative Group. N Engl J Med. 2004; 350:351-360.

54 Arriagada R, Dunant A, Pignon JP, et al: Long-term results of the International Adjuvant Lung Cancer Trial evaluating adjuvant cisplatin-based chemotherapy in resected lung cancer. J Clin Oncol 2010; 28:35-42.

55 Winton T, Livingston R, Johnson D, et al: Vinorelbine plus cisplatin vs. observation in resected non-small-cell lung cancer. N Engl J Med. 2005; 352:2589-2597.

56 Butts CA, Ding K, Seymour L, et al: Randomized, phase III trial of vinorelbine plus cisplatin compared with observation in completely resected stage IB and II non-small-cell lung cancer: Updated survival analysis of JBR-10. J Clin Oncol. 2010; 28:29-34.

57 Douillard JY, Rosell R, De Lena M, et al: Adjuvant vinorelbine plus cisplatin versus observation in patients with completely resected stage IB-IIIA non-small-cell lung cancer (Adjuvant Navelbine International Trialist Association [ANITA]): A randomised controlled trial. Lancet Oncol. 2006; 7:719-727.

58 NSCLC Meta-analysis Collaborative Group. Preoperative chemotherapy for non-small-cell lung cancer: a systematic review and meta-analysis of individual participant data. Lancet 2014;383:1561-71.

59 Rami-Porta, R. Bolejack, V. Crowley, J. et al. The IASLC Cancer Staging Project: Proposals for the Revisions of the T Descriptors in the Forthcoming Eigth Edition of the TNM Classification for Lung Cancer. J Thorac Oncol. 2015 Jul;10(7):990-1003.

60 NAVELBINE® (vinorelbine tartrate). Prescribing Information. 2002. GlaxoSmithKline. Research Triangle Park, NC.

61 GEMZAR (gemcitabine for injection), for intravenous use. Revised June 2014. Lilly, USA, Inc. Indianapolis, IN.

62 TAXOTERE® (docetaxel) Injection Concentrate, Intravenous Infusion (IV). Prescribing Information. Revised December 2015. sanofi-aventis, US, LLC, Bridgewater, NJ.

63 ALIMTA (pemetrexed for injection), for Intravenous Use. Update September 2013. Lilly, USA, Inc. Indianapolis, IN.

64 Platinol (cisplatin for injection). Prescribing information. Revised May 2010. Bristol-Myers Squibb Company, Princeton, NJ.

65 PARAPLATIN (carboplatin aqueous solution) INJECTION. Updated July 2010. Bristol-Myers Squibb Company, Princeton, NJ.

66 Paclitaxel Aurobindo 6 mg/ml Concentrate for solution for infusion. Summary of Product Characteristics. Puren Pharma.

67 Taxol (paclitaxel) Injection. Corden Pharma Latina. January 2011

[redacted]

Approved v 1.0 [redacted]

12 APPENDICES

APPENDIX 1 TNM STAGING SYSTEM FOR LUNG CANCER (7TH EDITION)

Table -1: International Association for the Study of Lung Cancer Definitions for T, N, and M Descriptors

**T (Primary Tumor)**			
T1	Tumor ≤ 3 cm in greatest dimension, surrounded by lung or visceral pleura, without invasion more proximal than the lobar bronchus		
T1a	Tumor ≤ 2 cm in diameter		
T1b	Tumor > 2 cm but ≤ 3 cm in diameter		
T2	Tumor > 3 cm but ≤ 7 cm or tumor with any of the following features<br>Involves main bronchus, ≥ 2 cm distal to carina<br>Invades visceral pleura<br>Associated with atelectasis or obstructive pneumonitis that extends to the hilar region but does not involve the entire lung		
T2a	Tumor > 3 cm but _5 cm		
T2b	Tumor > 5 cm but ≤ 7 cm		
T3	Tumor > 7 cm or any of the following:<br>Directly invades any of the following: chest wall, diaphragm, phrenic nerve, mediastinal pleura, parietal pericardium; main bronchus < 2 cm distal to the carina (without involvement of the carina)<br>Atlelectasis or obstructive pneumonitis of the entire lung<br>Separate tumor nodule in same lobe		
T4	Tumor of any size that invades any of the following: mediastinum, heart, great vessels, trachea, recurrent laryngeal nerve, esophagus, vertebral body, carina; or without separate tumor nodules in a different ipsilateral lobe		
**N (Regional Lymph Nodes)**			
N0	No regional lymph node metastasis		
N1	Metastasis in ipsilateral peribronchial and/or ipsilateral hilar lymph nodes and intrapulmonary nodes, including involvement by direct extension		
N2	Metastasis in ipsilateral mediastinal and/or subcarinal lymph node(s)		
N3	Metastasis in contralateral mediastinal, contralateral hilar, ipsilateral or contralateral scalene, or supraclavicular lymph node(s)		
**M (Distant Metastasis)**			
M0	No distant metastasis		
M1	Distant metastasis		
M1a	Separate tumor nodule(s) in a contralateral lobe; tumor with pleural nodules or malignant pleural or pericardial effusion		
M1b	Distant metastasis (in extrathoracic organs)		
**Stage Groupings**			
Stage IA	T1a-T1b	N0	M0
Stage IB	T2a	N0	M0

Approved v9.0 [redacted]

Table -1: International Association for the Study of Lung Cancer Definitions for T, N, and M Descriptors

Stage IIA	T1a, T1b, T2a	N1	M0
	T2b	N0	M0
Stage IIB	T2b	N1	M0
	T3	N0	M0
Stage IIIA	T1a, T1b, T2a, T2b	N2	M0
	T3	N1, N2	M0
	T4	N0, N1	M0
Stage IIIB	T4	N3	M0
	Any T	N3	M0
Stage IV	Any T	Any N	M1a or M1b

Adapted from Goldstraw P, Crowley J, Chansky K, et al. The IASLC Lung Cancer Staging Project: proposals for the revision of the TNM stage groupings in the forthcoming (seventh) edition of the TNM Classification of malignant tumours. J Thorac Oncol. 2007 Aug;2(8):706-14.

APPENDIX 2 ABBREVIATIONS AND TRADEMARKS

Term	Definition
AE	adverse event
ALT	alanine aminotransferase
ANC	absolute neutrophil count
AR	Additional Research
AST	aspartate aminotransferase
BICR	Blinded independent central review
BID, bid	bis in die, twice daily
BIPR	Blinded independent pathological review
BMI	body mass index
BMS	Bristol-Myers Squibb Company
BP	blood pressure
BUN	blood urea nitrogen
C	Celsius
CBC	complete blood count
CFR	Code of Federal Regulations
CI	confidence interval
$C1^{-}$	chloride
cm	centimeter
CONSORT	Consolidated Standards of Reporting Trials
CRF	Case Report Form, paper or electronic
cRR	clinical response rate
[redacted]	
dL	deciliter
DMC	Data Monitoring Committee
EBUS	endobronchial ultrasound
ECG	electrocardiogram
ECOG	Eastern Cooperative Oncology Group
eCRF	Electronic Case Report Form
EFS	event-free survival

Term	Definition
eg	exempli gratia (for example)
FA	final analysis
FDA	Food and Drug Administration
FFPE	formalin-fixed paraffin-embedded
FSH	follicle stimulating hormone
g	gram
GCP	Good Clinical Practice
GFR	glomerular filtration rate
[redacted]	
HBsAg	hepatitis B surface antigen
HBV	hepatitis B virus
HCV	hepatitis C virus
HIV	Human Immunodeficiency Virus
HR	heart rate
HRT	hormone replacement therapy
IA	Interim analysis
IB	Investigator’s brochure
ICH	International Conference on Harmonisation
ie	id est (that is)
IEC	Independent Ethics Committee
IHC	Immunohistochemical
IMP	investigational medicinal products
IRB	Institutional Review Board
IRT	Interactive Response Technology
IU	International Unit
IV	intravenous
kg	kilogram
L	liter
LC	liquid chromatography
LDH	lactate dehydrogenase

Term	Definition
mg	milligram
min	minute
mL	milliliter
mmHg	millimeters of mercury
MPR	major pathological response
MLND	mediastinal lymph node dissection
μg	microgram
N	number of subjects or observations
Na^+	sodium
N/A	not applicable
NCI CTCAE	National Cancer Institute Common Terminology Criteria for Adverse Events
NSCLC	non-small cell lung carcinoma
NIMP	non-investigational medicinal products
OS	overall survival
PBMC	peripheral blood mononuclear cells
pCR	Pathologic complete response
PFS	progression-free survival
PK	pharmacokinetics
PPK	Population pharmacokinetic
PO	per os (by mouth route of administration)
R&D	Research and Development
RCC	renal cell carcinoma
RECIST	Response Evaluation Criteria in Solid Tumors
SAE	serious adverse event
SCCHN	squamous cell carcinoma of the head and neck
SD	standard deviation
SNP	single nucleotide polymorphisms
SOC	Standard of care
SOP	Standard Operating Procedures

Approved v9.0 [redacted]

Term	Definition
t	temperature
[redacted]	
WHO	World Health Organization
WOCBP	women of childbearing potential

Approved v 9.0 [redacted]

APPENDIX 3 ECOG PERFORMANCE STATUS

ECOG PERFORMANCE STATUS [a]	
0	Fully active, able to carry on all pre-disease performance without restriction
1	Restricted in physically strenuous activity but ambulatory and able to carry out work of a light or sedentary nature, eg, light house work, office work
2	Ambulatory and capable of all selfcare but unable to carry out any work activities. Up and about more than 50% of waking hours
3	Capable of only limited selfcare, confined to bed or chair more than 50% of waking hours
4	Completely disabled. Cannot carry on any selfcare. Totally confined to bed or chair
5	Dead

[a] Oken MM, Creech RH, Tormey DC, Horton J, Davis TE, McFadden ET, and Carbone PP. Toxicity and Response Criteria of the Eastern Cooperative Oncology Group. Am J Clin Oncol 1982; 5: 649-655.

Approved v9.0 [redacted]

APPENDIX 4 NIVOLUMAB MANAGEMENT ALGORITHMS

These general guidelines constitute guidance to the Investigator and may be supplemented by discussions with the Medical Monitor representing the Sponsor. The guidance applies to all immuno-oncology agents and regimens.

A general principle is that differential diagnoses should be diligently evaluated according to standard medical practice. Non-inflammatory etiologies should be considered and appropriately treated.

Corticosteroids are a primary therapy for immuno-oncology drug-related adverse events. The oral equivalent of the recommended IV doses may be considered for ambulatory patients with low-grade toxicity. The lower bioavailability of oral corticosteroids should be taken into account when switching to the equivalent dose of oral corticosteroids.

Consultation with a medical or surgical specialist, especially prior to an invasive diagnostic or therapeutic procedure, is recommended.

The frequency and severity of the related adverse events covered by these algorithms will depend on the immuno-oncology agent or regimen being used.

Approved v 1.0 [redacted]

GI Adverse Event Management Algorithm

Rule out non-inflammatory causes. If non-inflammatory cause is identified, treat accordingly and continue I-O therapy. Opiates/narcotics may mask symptoms of perforation. Infliximab should not be used in cases of perforation or sepsis.

Grade of Diarrhea/ Colitis (NCI CTCAE v4)	Management	Follow-up
**Grade 1**<br>Diarrhea:< 4 stools/day over baseline;<br>Colitis: asymptomatic	• Continue I-O therapy per protocol<br>• Symptomatic treatment	• Close monitoring for worsening symptoms.<br>• Educate patient to report worsening immediately<br>If worsens:<br>• Treat as Grade 2 or 3/4
**Grade 2**<br>Diarrhea: 4-6 stools per day over baseline; IV fluids indicated <24 hrs; not interfering with ADL<br>Colitis: abdominal pain; blood in stool	• Delay I-O therapy per protocol<br>• Symptomatic treatment	If improves to grade 1:<br>• Resume I-O therapy per protocol<br>If persists > 5-7 days or recur:<br>• 0.5-1.0 mg/kg/day methylprednisolone or oral equivalent<br>• When symptoms improve to grade 1, taper steroids over at least 1 month, consider prophylactic antibiotics for opportunistic infections, and resume I-O therapy per protocol.<br>If worsens or persists > 3-5 days with oral steroids:<br>• Treat as grade 3/4
**Grade 3-4**<br>Diarrhea (G3): ≥7 stools per day over baseline; incontinence; IV fluids ≥24 hrs; interfering with ADL<br>Colitis (G3): severe abdominal pain, medical intervention indicated, peritoneal signs<br>G4: life-threatening, perforation	• Discontinue I-O therapy per protocol<br>• 1.0 to 2.0 mg/kg/day methylprednisolone IV or IV equivalent<br>• Add prophylactic antibiotics for opportunistic infections<br>• Consider lower endoscopy	If improves:<br>• Continue steroids until grade 1, then taper over at least 1 month<br>If persists > 3-5 days, or recurs after improvement:<br>• Add infliximab 5 mg/kg (if no contraindication). Note: Infliximab should not be used in cases of perforation or sepsis

Patients on IV steroids may be switched to an equivalent dose of oral corticosteroids (e.g. prednisone) at start of tapering or earlier, once sustained clinical improvement is observed. Lower bioavailability of oral corticosteroids should be taken into account when switching to the equivalent dose of oral corticosteroids.

27-Jun-2019

Renal Adverse Event Management Algorithm

Rule out non-inflammatory causes. If non-inflammatory cause, treat accordingly and continue I-O therapy.

Grade of Creatinine Elevation (NCI CTCAE v4)	Management	Follow-up
**Grade 1**<br>Creatinine > ULN and > than baseline but ≤ 1.5x baseline	• Continue I-O therapy per protocol<br>• Monitor creatinine weekly	If returns to baseline:<br>•Resume routine creatinine monitoring per protocol<br>If worsens:<br>•Treat as Grade 2 or 3/4
**Grade 2-3**<br>Creatinine > 1.5x baseline to ≤ 6x ULN	• Delay I-O therapy per protocol<br>• Monitor creatinine every 2-3 days<br>• 0.5 to 1.0 mg/kg/day methylprednisolone IV or oral equivalent<br>• Consider renal biopsy with nephrology consult	If returns to Grade 1:<br>•Taper steroids over at least 1 month, consider prophylactic antibiotics for opportunistic infections, and resume I-O therapy and routine creatinine monitoring per protocol<br><br>If elevations persist > 7 days or worsen:<br>•Treat as Grade 4
**Grade 4**<br>Creatinine > 6x ULN	• Discontinue I-O therapy per protocol<br>• Monitor creatinine daily<br>• 1.0-2.0 mg/kg/day methylprednisolone IV or IV equivalent<br>• Consult nephrologist<br>• Consider renal biopsy	If returns to Grade 1 : Taper steroids over at least 1 month and add prophylactic antibiotics for opportunistic infections

Patients on IV steroids may be switched to an equivalent dose of oral corticosteroids (e.g. prednisone) at start of tapering or earlier, once sustained clinical improvement is observed. Lower bioavailability of oral corticosteroids should be taken into account when switching to the equivalent dose of oral corticosteroids.

27-Jun-2019

Approved v1.0 [redacted]

Pulmonary Adverse Event Management Algorithm

Rule out non-inflammatory causes. If non-inflammatory cause, treat accordingly and continue I-O therapy. Evaluate with imaging and pulmonary consultation.

Grade of Pneumonitis (NCI CTCAE v4)	Management	Follow-up
**Grade 1**<br>Radiographic changes only	• Consider delay of I-O therapy<br>• Monitor for symptoms every 2-3 days<br>• Consider Pulmonary and ID consults	• Re-image at least every 3 weeks<br><u>If worsens:</u><br>• Treat as Grade 2 or 3-4
**Grade 2**<br>Mild to moderate new symptoms	• Delay I-O therapy per protocol<br>• Pulmonary and ID consults<br>• Monitor symptoms daily, consider hospitalization<br>• 1.0 mg/kg/day methyl-prednisolone IV or oral equivalent<br>• Consider bronchoscopy , lung biopsy	• Re-image every 1-3 days<br><u>If improves:</u><br>• When symptoms return to near baseline, taper steroids over at least 1 month and then resume I-O therapy per protocol and consider prophylactic antibiotics<br><u>If not improving after 2 weeks or worsening:</u><br>• Treat as Grade 3-4
**Grade 3-4**<br>Severe new symptoms;<br>New/worsening hypoxia;<br>Life-threatening	• Discontinue I-O therapy per protocol<br>• Hospitalize<br>• Pulmonary and ID consults<br>• 2-4 mg/kg/day methylprednisolone IV or IV equivalent<br>• Add prophylactic antibiotics for opportunistic infections<br>• Consider bronchoscopy, lung biopsy	<u>If improves to baseline:</u><br>• Taper steroids over at least 6 weeks<br><u>If not improving after 48 hours or worsening:</u><br>• Add additional immunosuppression

Patients on IV steroids may be switched to an equivalent dose of oral corticosteroids (e.g. prednisone) at start of tapering or earlier, once sustained clinical improvement is observed. Lower bioavailability of oral corticosteroids should be taken into account when switching to the equivalent dose of oral corticosteroids

27-Jun-2019

Hepatic Adverse Event Management Algorithm

Rule out non-inflammatory causes. If non-inflammatory cause, treat accordingly and continue I-O therapy. Consider imaging for obstruction.

Grade of Liver Test Elevation (NCI CTCAE v4)	Management	Follow-up
**Grade 1**<br>AST or ALT > ULN to 3.0 x ULN and/or T. bili > ULN to 1.5 x ULN	• Continue I-O therapy per protocol	• Continue LFT monitoring per protocol<br>If worsens:<br>• Treat as Grade 2 or 3-4
**Grade 2**<br>AST or ALT > 3.0 to ≤ 5 x ULN and/or T. bili > 1.5 to ≤ 3 x ULN	• Delay I-O therapy per protocol<br>• Increase frequency of monitoring to every 3 days	If returns to baseline:<br>•Resume routine monitoring, resume I-O therapy per protocol<br><br>If elevations persist > 5-7 days or worsen :<br>• 0.5-1 mg/kg/day methylprednisolone or oral equivalent and when LFT returns to grade 1 or baseline, taper steroids over at least 1 month, consider prophylactic antibiotics for opportunistic infections, and resume I-O therapy per protocol
**Grade 3-4**<br>AST or ALT > 5 x ULN or T.bili >3 x ULN	• Discontinue I-O therapy*<br>• Increase frequency of monitoring to every 1-2 days<br>• 1.0 to 2.0 mg/kg/day methylprednisolone IV or IV equivalent*<br>• Add prophylactic antibiotics for opportunistic infections<br>• Consult gastroenterologist	If returns to grade 2:<br>• Taper steroids over at least 1 month<br><br>If does not improve in >3-5 days, worsens or rebounds:<br>• Add mycophenolate mofetil 1 g BID<br>• If no response within an additional 3-5 days, consider other immunosuppressants per local guidelines

Patients on IV steroids may be switched to an equivalent dose of oral corticosteroids (e.g. prednisone) at start of tapering or earlier, once sustained clinical improvement is observed. Lower bioavailability of oral corticosteroids should be taken into account when switching to the equivalent dose of oral corticosteroids.

*The recommended starting dose for grade 4 hepatitis is 2 mg/kg/day methylprednisolone IV.

27-Jun-2019

Approved v1.0 [redacted]

Endocrinopathy Adverse Event Management Algorithm

Rule out non-inflammatory causes. If non-inflammatory cause, treat accordingly and continue I-O therapy. Consider visual field testing, endocrinology consultation, and imaging.

Asymptomatic TSH elevation

- Continue I-O therapy per protocol
- If TSH < 0.5 x LLN, or TSH > 2 x ULN, or consistently out of range in 2 subsequent measurements: include fT4 at subsequent cycles as clinically indicated; consider endocrinology consult

Symptomatic endocrinopathy

- Evaluate endocrine function
- Consider pituitary scan

Symptomatic with abnormal lab/pituitary scan:
- Delay I-O therapy per protocol
- 1-2 mg/kg/day methylprednisolone IV or PO equivalent
- Initiate appropriate hormone therapy

No abnormal lab/pituitary MRI scan but symptoms persist:
- Repeat labs in 1-3 weeks / MRI in 1 month

If improves (with or without hormone replacement):
- Taper steroids over at least 1 month and consider prophylactic antibiotics for opportunistic infections
- Resume I-O therapy per protocol
- Patients with adrenal insufficiency may need to continue steroids with mineralocorticoid component

Suspicion of adrenal crisis (e.g. severe dehydration, hypotension, shock out of proportion to current illness

- Delay or discontinue I-O therapy per protocol
- Rule out sepsis
- Stress dose of IV steroids with mineralocorticoid activity
- IV fluids
- Consult endocrinologist
- If adrenal crisis ruled out, then treat as above for symptomatic endocrinopathy

Patients on IV steroids may be switched to an equivalent dose of oral corticosteroids (e.g. prednisone) at start of tapering or earlier, once sustained clinical improvement is observed. Lower bioavailability of oral corticosteroids should be taken into account when switching to the equivalent dose of oral corticosteroids.

27-Jun-2019

Skin Adverse Event Management Algorithm

Rule out non-inflammatory causes. If non-inflammatory cause, treat accordingly and continue I-O therapy.

Grade of Rash (NCI CTCAE v4)	Management	Follow-up
**Grade 1-2**<br>Covering ≤ 30% BSA*	• Symptomatic therapy (e.g. antihistamines, topical steroids)<br>• Continue I-O therapy per protocol	<u>If persists > 1-2 weeks or recurs:</u><br>• Consider skin biopsy<br>• Delay I-O therapy per protocol<br>• Consider 0.5-1.0 mg/kg/day methylprednisolone IV or oral equivalent. Once improving , taper steroids over at least 1 month, consider prophylactic antibiotics for opportunistic infections, and resume I-O therapy per protocol<br><u>If worsens:</u><br>• Treat as Grade 3-4
**Grade 3-4**<br>Covering >30% BSA; Life threatening consequences*^	• Delay or discontinue I-O therapy per protocol<br>• Consider skin biopsy<br>• Dermatology consult<br>• 1.0-2.0 mg/kg/day IV methylprednisolone IV or IV equivalent	<u>If improves to Grade 1:</u><br>• Taper steroids over at least 1 month and add prophylactic antibiotics for opportunistic infections<br>• Resume I-O therapy per protocol

Patients on IV steroids may be switched to an equivalent dose of oral corticosteroids (e.g. prednisone) at start of tapering or earlier, once sustained clinical improvement is observed. Lower bioavailability of oral corticosteroids should be taken into account when switching to the equivalent dose of oral corticosteroids.

*Refer to NCI CTCAE v4 for term-specific grading criteria.

^If SJS/TEN is suspected, withhold I-O therapy and refer patient for specialized care for assessment and treatment. If SJS or TEN is diagnosed, permanently discontinue I-O therapy.

27-Jun-2019

Neurological Adverse Event Management Algorithm

Rule out non-inflammatory causes. If non-inflammatory cause, treat accordingly and continue I-O therapy.

Grade of Neurological Toxicity (NCI CTCAE v4)	Management	Follow-up
**Grade 1**<br>Asymptomatic or mild symptoms; Intervention not indicated	• Continue I-O therapy per protocol	Continue to monitor the patient.<br>If worsens:<br>• Treat as Grade 2 or 3-4
**Grade 2**<br>Moderate symptoms; Limiting instrumental ADL	• Delay I-O therapy per protocol<br>• Treat symptoms per local guidelines<br>• Consider 0.5 to 1.0 mg/kg/day methylprednisolone IV or PO equivalent	If improves to baseline:<br>•Resume I-O therapy per protocol when improved to baseline<br>If worsens:<br>• Treat as Grade 3-4
**Grade 3-4**<br>Severe symptoms; Limiting self-care ADL; Life-threatening	• Discontinue I-O therapy per protocol<br>• Obtain neurology consult<br>• Treat symptoms per local guidelines<br>• 1.0-2.0 mg/kg/day IV methylprednisolone IV or IV equivalent<br>• Add prophylactic antibiotics for opportunistic infections	If improves to Grade 2:<br>• Taper steroids over at least 1 month<br><br>If worsens or atypical presentation:<br>• Consider IVIG or other immunosuppressive therapies per local guidelines

Patients on IV steroids may be switched to an equivalent dose of oral corticosteroids (e.g. prednisone) at start of tapering or earlier, once sustained clinical improvement is observed. Lower bioavailability of oral corticosteroids should be taken into account when switching to the equivalent dose of oral corticosteroids.

27-Jun-2019

Myocarditis Adverse Event Management Algorithm

Grade of Myocarditis (NCI CTCAE v4)	Management	Follow-up
**Grade 2**<br><br>**Symptoms with mild to moderate activity or exertion**	• Delay I-O therapy; hospitalization with cardiac monitoring<br>• Urgent cardiology consultation for evaluation and management<br>o Troponin and BNP<br>o ECG ± continuous cardiac monitoring<br>o Echocardiogram<br>o Cardiac MRI<br>• Prompt initiation of 2 mg/kg/day methylprednisolone IV or equivalent	• If worsens, intensify treatment according to grade<br>• Upon recovery, taper steroids over at least 1 month with close monitoring of troponin and BNP as well as for new symptoms<br>• Repeat cardiac MRI for post treatment assessment and cardiology follow-up<br>• Retreatment may be considered after recovery and completion of steroid taper
**<u>Grade 3</u>: Severe with symptoms at rest or with minimal activity or exertion; intervention indicated**<br><br>**<u>Grade 4</u>: Life-threatening consequences; urgent intervention indicated (e.g., continuous IV therapy or mechanical hemodynamic support)**	• Permanently discontinue I-O therapy<br>• Hospitalize to intensive cardiac monitoring<br>• Cardiac evaluation to include:<br>– Troponin and BNP monitoring<br>– ECG ± continuous cardiac monitoring<br>– Echocardiogram<br>– Cardiac MRI<br>– Myocardial biopsy if feasible<br>• Immediate initiation of 2 mg/kg/day methylprednisolone IV or 1 g IV bolus<br>• Consider adding a second immunosuppressive agent<br>Additionally, for Grade 4:<br>• Hospitalize/transfer to institution with expertise in intensive cardiac monitoring<br>• Consider ATG as second agent given its immediate effect	• If no improvement, consider additional immunosuppression<br>• Upon recovery, taper steroids over at least 1 month with close monitoring of troponin and BNP as well as for new symptoms<br>• Repeat cardiac MRI for post treatment assessments and cardiology follow-up

Patients on IV steroids may be switched to an equivalent dose of oral corticosteroids (eg, prednisone) at start of tapering or earlier, once sustained clinical improvement is observed. Lower bioavailability of oral corticosteroids should be taken into account when switching to the equivalent dose of oral corticosteroids.

Prophylactic antibiotics should be considered in the setting of ongoing immunosuppression.

ATG = anti-thymocyte globulin; BNP = B-type natriuretic peptide; ECG = electrocardiogram; IV = intravenous; MRI = magnetic resonance imaging

27-Jun-2019

APPENDIX 5 RESPONSE CRITERIA (RECIST 1.1)

1 EVALUATION OF LESIONS

At baseline, tumor lesions/lymph nodes will be categorized measurable or non-measurable as follows:

1.1 Measurable

Tumor lesions: Must be accurately measured in at least one dimension (longest diameter in the plane of measurement is to be recorded) with a minimum size of:

1) 10 mm by CT scan (CT scan slice thickness no greater than 5 mm)
2) 10 mm caliper measurement by clinical exam (lesions which cannot be accurately measured with calipers should be recorded as non-measurable)
3) 20 mm by chest x-ray

Malignant lymph nodes: To be considered pathologically enlarged and measurable, a lymph node must be ≥ 15 mm in short axis when assessed by CT scan (CT scan slice thickness recommended to be no greater than 5 mm).

Lymph nodes merit special mention since they are normal anatomical structures which may be visible by imaging even if not involved by tumor. Pathological nodes which are defined as measurable and may be identified as target lesions must meet the criterion of a short axis of ≥ 15 mm by CT scan. Only the short axis of these nodes will contribute to the baseline sum. The short axis of the node is the diameter normally used by radiologists to judge if a node is involved by solid tumor. Nodal size is normally reported as two dimensions in the plane in which the image is obtained (for CT scan this is almost always the axial plane; for MRI the plane of acquisition may be axial, saggital or coronal). The smaller of these measures is the short axis. For example, an abdominal node which is reported as being 20 mm x 30 mm has a short axis of 20 mm and qualifies as a malignant, measurable node. In this example, 20 mm should be recorded as the node measurement. All other pathological nodes (those with short axis ≥ 10 mm but < 15 mm) should be considered non-target lesions. Nodes that have a short axis < 10 mm are considered non-pathological and should not be recorded or followed.

1.2 Non-Measurable

All other lesions are considered non-measurable, including small lesions (longest diameter < 10mm or pathological lymph nodes with ≥ 10 to < 15 mm short axis) as well as truly non-measurable lesions. Lesions considered truly non-measurable include: leptomeningeal disease, ascites, pleural or pericardial effusion, inflammatory breast disease, lymphangitic involvement of skin or lung, abdominal masses/abdominal organomegaly identified by physical exam that is not measurable by reproducible imaging techniques.

Approved v 9.0 [redacted]

2 BASELINE DOCUMENTATION OF 'TARGET' AND 'NON-TARGET' LESIONS

When more than one measurable lesion is present at baseline all lesions up to a maximum of five lesions total (and a maximum of two lesions per organ) representative of all involved organs should be identified as target lesions and will be recorded and measured at baseline (this means in instances where patients have only one or two organ sites involved a maximum of two and four lesions respectively will be recorded).

Target lesions should be selected on the basis of their size (lesions with the longest diameter), be representative of all involved organs, but in addition should be those that lend themselves to reproducible repeated measurements. It may be the case that, on occasion, the largest lesion does not lend itself to reproducible measurement in which circumstance the next largest lesion which can be measured reproducibly should be selected.

A sum of the diameters (longest for non-nodal lesions, short axis for nodal lesions) for all target lesions will be calculated and reported as the baseline sum diameters. If lymph nodes are to be included in the sum, then as noted above, only the short axis is added into the sum. The baseline sum diameters will be used as reference to further characterize any objective tumor regression in the measurable dimension of the disease.

All other lesions (or sites of disease) including pathological lymph nodes should be identified as non-target lesions and should also be recorded at baseline. Measurements are not required and these lesions should be followed as 'present', 'absent', or in rare cases 'unequivocal progression' (more details to follow). In addition, it is possible to record multiple nontarget lesions involving the same organ as a single item on the case record form (eg, 'multiple enlarged pelvic lymph nodes' or 'multiple liver metastases').

3 RESPONSE CRITERIA

3.1 Evaluation of Target Lesions

Complete Response (CR): Disappearance of all target lesions. Any pathological lymph nodes (whether target or non-target) must have reduction in short axis to < 10 mm.

Partial Response (PR): At least a 30% decrease in the sum of diameters of target lesions, taking as reference the baseline sum diameters.

Progressive Disease (PD): At least a 20% increase in the sum of diameters of target lesions, taking as reference the smallest sum on study (this includes the baseline sum if that is the smallest on study). In addition to the relative increase of 20%, the sum must also demonstrate an absolute increase of at least 5 mm. (Note: the appearance of one or more new lesions is also considered progression).

Stable Disease (SD): Neither sufficient shrinkage to qualify for PR nor sufficient increase to qualify for PD, taking as reference the smallest sum diameters while on study.

Approved v 1.0 [redacted]

3.1.1 Special Notes on the Assessment of Target Lesions

3.1.1.1 Lymph nodes

Lymph nodes identified as target lesions should always have the actual short axis measurement recorded (measured in the same anatomical plane as the baseline examination), even if the nodes regress to below 10 mm on study. This means that when lymph nodes are included as target lesions, the ‘sum’ of lesions may not be zero even if complete response criteria are met, since a normal lymph node is defined as having a short axis of < 10 mm. Case report forms or other data collection methods may therefore be designed to have target nodal lesions recorded in a separate section where, in order to qualify for CR, each node must achieve a short axis < 10 mm. For PR, SD and PD, the actual short axis measurement of the nodes is to be included in the sum of target lesions.

3.1.1.2 Target lesions that become ‘too small to measure’

While on study, all lesions (nodal and non-nodal) recorded at baseline should have their actual measurements recorded at each subsequent evaluation, even when very small (eg, 2 mm). However, sometimes lesions or lymph nodes which are recorded as target lesions at baseline become so faint on CT scan that the radiologist may not feel comfortable assigning an exact measure and may report them as being ‘too small to measure’. When this occurs it is important that a value be recorded on the case report form. If it is the opinion of the radiologist that the lesion has likely disappeared, the measurement should be recorded as 0 mm. If the lesion is believed to be present and is faintly seen but too small to measure, a default value of 5 mm should be assigned (Note: It is less likely that this rule will be used for lymph nodes since they usually have a definable size when normal and are frequently surrounded by fat such as in the retroperitoneum; however, if a lymph node is believed to be present and is faintly seen but too small to measure, a default value of 5 mm should be assigned in this circumstance as well). This default value is derived from the 5 mm CT slice thickness (but should not be changed with varying CT slice thickness). The measurement of these lesions is potentially non-reproducible, therefore providing this default value will prevent false responses or progressions based upon measurement error. To reiterate, however, if the radiologist is able to provide an actual measure, that should be recorded, even if it is below 5 mm.

3.1.1.3 Lesions that split or coalesce on treatment

When non-nodal lesions ‘fragment’, the longest diameters of the fragmented portions should be added together to calculate the target lesion sum. Similarly, as lesions coalesce, a plane between them may be maintained that would aid in obtaining maximal diameter measurements of each individual lesion. If the lesions have truly coalesced such that they are no longer separable, the vector of the longest diameter in this instance should be the maximal longest diameter for the ‘coalesced lesion’.

3.2 Evaluation of Non-Target Lesions

This section provides the definitions of the criteria used to determine the tumor response for the group of non-target lesions. While some non-target lesions may actually be measurable, they need not be measured and instead should be assessed only qualitatively at the time points specified in the protocol.

Approved v9.0 [redacted]

Complete Response (CR): Disappearance of all non-target lesions and normalization of tumor marker level. All lymph nodes must be non-pathological in size (< 10mm short axis).

Non-CR/Non-PD: Persistence of one or more non-target lesion(s) and/or maintenance of tumor marker level above the normal limits.

Progressive Disease (PD): Unequivocal progression (see comments below) of existing non-target lesions. (Note: the appearance of one or more new lesions is also considered progression).

3.2.1 Special Notes on Assessment of Progression of Non-Target Disease

The concept of progression of non-target disease requires additional explanation as follows:

3.2.1.1 When the patient also has measurable disease

In this setting, to achieve ‘unequivocal progression’ on the basis of the non-target disease, there must be an overall level of substantial worsening in non-target disease such that, even in presence of SD or PR in target disease, the overall tumor burden has increased sufficiently to merit discontinuation of therapy (see further details below). A modest ‘increase’ in the size of one or more non-target lesions is usually not sufficient to qualify for unequivocal progression status. The designation of overall progression solely on the basis of change in non-target disease in the face of SD or PR of target disease will therefore be extremely rare.

3.2.1.2 When the patient has only non-measurable disease

This circumstance arises in some trials when it is not a criterion of study entry to have measurable disease. The same general concepts apply here as noted above, however, in this instance there is no measurable disease assessment to factor into the interpretation of an increase in non-measurable disease burden. Because worsening in non-target disease cannot be easily quantified (by definition: if all lesions are truly non-measurable) a useful test that can be applied when assessing patients for unequivocal progression is to consider if the increase in overall disease burden based on the change in non-measurable disease is comparable in magnitude to the increase that would be required to declare PD for measurable disease: ie, an increase in tumor burden representing an additional 73% increase in ‘volume’ (which is equivalent to a 20% increase diameter in a measurable lesion). Examples include an increase in a pleural effusion from ‘trace’ to ‘large’, an increase in lymphangitic disease from localized to widespread, or may be described in protocols as ‘sufficient to require a change in therapy’. If ‘unequivocal progression’ is seen, the patient should be considered to have had overall PD at that point. While it would be ideal to have objective criteria to apply to non-measurable disease, the very nature of that disease makes it impossible to do so; therefore the increase must be substantial.

3.2.2 New Lesions

The appearance of new malignant lesions denotes disease progression; therefore, some comments on detection of new lesions are important. There are no specific criteria for the identification of new radiographic lesions; however, the finding of a new lesion should be unequivocal: ie, not attributable to differences in scanning technique, change in imaging modality or findings thought to represent something other than tumor (for example, some ‘new’ bone lesions may be simply healing or flare of pre-existing lesions). This is particularly important when the patient’s baseline

Approved v9.0 [redacted]

lesions show partial or complete response. For example, necrosis of a liver lesion may be reported on a CT scan report as a 'new' cystic lesion, which it is not.

A lesion identified on a follow-up study in an anatomical location that was not scanned at baseline is considered a new lesion and will indicate disease progression. An example of this is the patient who has visceral disease at baseline and while on study has a CT or MRI brain ordered which reveals metastases. The patient's brain metastases are considered to be evidence of PD even if he/she did not have brain imaging at baseline.

If a new lesion is equivocal, for example because of its small size, continued therapy and follow-up evaluation will clarify if it represents truly new disease. If repeat scans confirm there is definitely a new lesion, then progression should be declared using the date of the initial scan. While FDG-PET response assessments need additional study, it is sometimes reasonable to incorporate the use of FDG-PET scanning to complement CT scanning in assessment of progression (particularly possible 'new' disease). New lesions on the basis of FDG-PET imaging can be identified according to the following algorithm:

4) Negative FDG-PET at baseline, with a positive FDG-PET at follow-up is a sign of PD based on a new lesion.
5) No FDG-PET at baseline and a positive FDG-PET at follow-up: If the positive FDG-PET at follow-up corresponds to a new site of disease confirmed by CT, this is PD. If the positive FDG-PET at follow-up is not confirmed as a new site of disease on CT, additional follow-up CT scans are needed to determine if there is truly progression occurring at that site (if so, the date of PD will be the date of the initial abnormal FDG-PET scan). If the positive FDG-PET at follow-up corresponds to a pre-existing site of disease on CT that is not progressing on the basis of the anatomic images, this is not PD.

3.3 Response Assessment

3.3.1 Evaluation of Best Overall Response

The best overall response is the best response recorded from the start of the study treatment until the end of treatment taking into account any requirement for confirmation. The patient's best overall response assignment will depend on the findings of both target and non-target disease and will also take into consideration the appearance of new lesions. Furthermore, depending on the nature of the study and the protocol requirements, it may also require confirmatory measurement.

3.3.2 Time Point Response

It is assumed that at each protocol specified time point, a response assessment occurs. Table 3.3.2-1 provides a summary of the overall response status calculation at each time point for patients who have measurable disease at baseline. When patients have non-measurable (therefore non-target) disease only, Table 3.3.2-2 is to be used.

Approved v 9.0 [redacted]

Table 3.3.2-1: Time Point Response - Patients With Target (+/- Non-Target) Disease

Target Lesions	Non-Target Lesions	New Lesions	Overall Response
CR	CR	No	CR
CR	Non-CR/non-PD	No	PR
CR	Not evaluated	No	PR
PR	Non-PD or not all evaluated	No	PR
SD	Non-PD or not all evaluated	No	SD
Not all evaluated	Non-PD	No	NE
PD	Any	Yes or No	PD
Any	PD	Yes or No	PD
Any	Any	Yes	PD

CR = complete response, PR = partial response, SD = stable disease, PD = progressive disease and NE = inevaluable

Table 3.3.2-2: Time Point Response - Patients with Non-target Disease Only

Non-Target Lesions	New Lesions	Overall Response
CR	No	CR
Non-CR/non-PD	No	Non-CR/non-PD[a]
Not all evaluated	No	NE
Unequivocal PD	Yes or No	PD
Any	Yes	PD
CR = complete response, PD = progressive disease and NE = inevaluable		

[a] Non-CR/non-PD is preferred over SD for non-target disease since SD is increasingly used as endpoint for assessment of efficacy in some trials so to assign this category when no lesions can be measured is not advised.

3.3.3 Best Overall Response

Best response determination of complete or partial response requires confirmation: Complete or partial responses may be claimed only if the criteria for each are met at a subsequent time point of ≥ 4 weeks later. In this circumstance, the best overall response can be interpreted as in Table 3.3.3-1.

Special note on response assessment: When nodal disease is included in the sum of target lesions and the nodes decrease to 'normal' size (< 10 mm), they may still have a measurement reported on scans. This measurement should be recorded even though the nodes are normal in order not to overstate progression should it be based on increase in size of the nodes. As noted earlier, this means that patients with CR may not have a total sum of 'zero' on the case report form (CRF).

Approved v 1.0 [redacted]

Table 3.3.3-1: Best Overall Response (Confirmation of CR&PR Required)		
**Overall Response First Time Point**	**Overall Response Subsequent Time Point**	**BEST Overall Response**
CR	CR	CR
CR	PR	SD, PD OR PR[a]
CR	SD	SD provided minimum criteria for SD duration[b] met, otherwise, PD
CR	PD	SD provided minimum criteria for SD duration[b] met, otherwise, PD
CR	NE	SD provided minimum criteria for SD duration[b] met, otherwise, NE
PR	CR	PR
PR	PR	PR
PR	SD	SD
PR	PD	SD provided minimum criteria for SD duration[b] met, otherwise, PD
PR	NE	SD provided minimum criteria for SD duration [b]met, otherwise, NE
NE	NE	NE
CR = complete response, PR = partial response, SD = stable disease, PD = progressive disease, and NE = inevaluable		

[a] If a CR is truly met at first time point, then any disease seen at a subsequent time point, even disease meeting PR criteria relative to baseline, makes the disease PD at that point (since disease must have reappeared after CR). Best response would depend on whether minimum duration for SD was met. However, sometimes 'CR' may be claimed when subsequent scans suggest small lesions were likely still present and in fact the patient had PR, not CR at the first time point. Under these circumstances, the original CR should be changed to PR and the best response is PR.

[b] Minimum criteria for SD duration is 6 weeks.

3.3.4 Confirmation Scans

Verification of Response: To be assigned a status of CR or PR, changes in tumor measurements must be confirmed by consecutive repeat assessments that should be performed no less than 28 days after the criteria for response are first met. For this study, the next scheduled tumor assessment can meet this requirement.

Verification of Progression: Progression of disease should be verified in cases where progression is equivocal. If repeat scans confirm PD, then progression should be declared using the date of the initial scan. If repeat scans do not confirm PD, then the subject is considered to not have progressive disease.

Approved v[illegible].0 [redacted]

APPENDIX 6 WOMEN OF CHILDBEARING POTENTIAL DEFINITIONS AND METHODS OF CONTRACEPTION

DEFINITIONS

Woman of Childbearing Potential (WOCBP)

A woman is considered fertile following menarche and until becoming post-menopausal unless permanently sterile. Permanent sterilization methods include hysterectomy, bilateral salpingectomy, and bilateral oophorectomy.

Women in the following categories are not considered WOCBP

- Premenarchal
- Premenopausal female with 1 of the following:
  - Documented hysterectomy
  - Documented bilateral salpingectomy
  - Documented bilateral oophorectomy

Note: Documentation can come from the site personnel's review of the participant's medical records, medical examination, or medical history interview.

- Postmenopausal female
  - A postmenopausal state is defined as 12 months of amenorrhea in a woman over age 45 years in the absence of other biological or physiological causes. In addition, females under the age of 55 years must have a serum follicle stimulating hormone, (FSH) level > 40 mIU/mL to confirm menopause.

CONTRACEPTION GUIDANCE FOR FEMALE PARTICIPANTS OF CHILD BEARING POTENTIAL

One of the highly effective methods of contraception listed below is required during study duration and until the end of relevant systemic exposure, defined as 5 months after the end of study treatment.

Local laws and regulations may require use of alternative and/or additional contraception methods.

**Highly Effective Contraceptive Methods That Are User Dependent**<br>*Failure rate of <1% per year when used consistently and correctly.a*
• Combined (estrogen- and progestogen-containing) hormonal contraception associated with inhibition of ovulation[b]<br>– oral<br>– intravaginal<br>– transdermal<br>• Progestogen-only hormonal contraception associated with inhibition of ovulation[b]

Approved v 9.0 [redacted]

- oral
- injectable

Highly Effective Methods That Are User Independent

- Implantable progestogen-only hormonal contraception associated with inhibition of ovulation [b]
- Hormonal methods of contraception including oral contraceptive pills containing a combination of estrogen and progesterone, vaginal ring, injectables, implants and intrauterine hormone-releasing system (IUS)[c]
- Intrauterine device (IUD)[c]
- Bilateral tubal occlusion

- Vasectomized partner

A vasectomized partner is a highly effective contraception method provided that the partner is the sole male sexual partner of the WOCBP and the absence of sperm has been confirmed. If not, an additional highly effective method of contraception should be used.

- Sexual abstinence

Sexual abstinence is considered a highly effective method only if defined as refraining from heterosexual intercourse during the entire period of risk associated with the study drug. The reliability of sexual abstinence needs to be evaluated in relation to the duration of the study and the preferred and usual lifestyle of the participant.

- It is not necessary to use any other method of contraception when complete abstinence is elected.
- WOCBP participants who choose complete abstinence must continue to have pregnancy tests, as specified in Section 2.
- Acceptable alternate methods of highly effective contraception must be discussed in the event that the WOCBP participants chooses to forego complete abstinence

NOTES:

[a] Typical use failure rates may differ from those when used consistently and correctly. Use should be consistent with local regulations regarding the use of contraceptive methods for participants participating in clinical studies.

[b] Hormonal contraception may be susceptible to interaction with the study drug, which may reduce the efficacy of the contraceptive method. Hormonal contraception is permissible only when there is sufficient evidence that the IMP and other study medications will not alter hormonal exposures such that contraception would be ineffective or result in increased exposures that could be potentially hazardous. In this case, alternative methods of contraception should be utilized.

[c] Intrauterine devices and intrauterine hormone releasing systems are acceptable methods of contraception in the absence of definitive drug interaction studies when hormone exposures from intrauterine devices do not alter contraception effectiveness

Approved v9.0 [redacted]

Unacceptable Methods of Contraception
• Male or female condom with or without spermicide. Male and female condoms cannot be used simultaneously<br>• Diaphragm with spermicide<br>• Cervical cap with spermicide<br>• Vaginal Sponge with spermicide<br>• Periodic abstinence (calendar, symptothermal, post-ovulation methods)<br>• Withdrawal(coitus interruptus).<br>• Spermicide only<br>• Lactation amenorrhea method (LAM)

CONTRACEPTION GUIDANCE FOR MALE PARTICIPANTS WITH PARTNER(S) OF CHILD BEARING POTENTIAL.

Male participants with female partners of childbearing potential are eligible to participate if they agree to the following during the treatment and until the end of relevant systemic exposure.

- Inform any and all partner(s) of their participation in a clinical drug study and the need to comply with contraception instructions as directed by the investigator.
- Male participants are required to use a condom for study duration and until the end of relevant systemic exposure defined as 7 months after the end of treatment in the male participant.
- Female partners of males participating in the study to consider use of effective methods of contraception until the end of relevant systemic exposure, defined as 7 months after the end of treatment in the male participant.
- Male participants with a pregnant or breastfeeding partner must agree to remain abstinent from penile vaginal intercourse or use a male condom during each episode of penile penetration during the treatment and until 7 months after the end of treatment.
- Refrain from donating sperm for the duration of the study treatment and until 7 months after the end of treatment.

COLLECTION OF PREGNANCY INFORMATION

Guidance for collection of Pregnancy Information and outcome of pregnancy on the Pregnancy Surveillance Form is provided in Section 9.2.5 and the Appendix for Adverse Events and Serious Adverse Events Definitions and procedures for Evaluating, Follow-up and Reporting

Approved v1.0 [redacted]

APPENDIX 7 STUDY GOVERNANCE CONSIDERATIONS

The term 'Participant' is used in the protocol to refer to a person who has consented to participate in the clinical research study. The term 'Subject' used in the eCRF is intended to refer to a person (Participant) who has consented to participate in the clinical research study.

REGULATORY AND ETHICAL CONSIDERATIONS

GOOD CLINICAL PRACTICE

This study will be conducted in accordance with:

- Good Clinical Practice (GCP),
- as defined by the International Council on Harmonisation (ICH)
- in accordance with the ethical principles underlying European Union Directive 2001/20/EC
- United States Code of Federal Regulations, Title 21, Part 50 (21CFR50)
- applicable local requirements.

The study will be conducted in compliance with the protocol. The protocol and any amendments and the participant informed consent will receive approval/favorable opinion by Institutional Review Board/Independent Ethics Committee (IRB/IEC), and regulatory authorities according to applicable local regulations prior to initiation of the study.

All potential serious breaches must be reported to Sponsor or designee immediately. A serious breach is a breach of the conditions and principles of GCP in connection with the study or the protocol, which is likely to affect, to a significant degree, the safety or physical or mental integrity of the subjects of the study or the scientific value of the study.

Personnel involved in conducting this study will be qualified by education, training, and experience to perform their respective tasks.

This study will not use the services of study personnel where sanctions have been invoked or where there has been scientific misconduct or fraud (e.g., loss of medical licensure, debarment).

INSTITUTIONAL REVIEW BOARD/INDEPENDENT ETHICS COMMITTEE

Before study initiation, the investigator must have written and dated approval/favorable opinion from the IRB/IEC for the protocol, consent form, participant recruitment materials (e.g., advertisements), and any other written information to be provided to subjects. The investigator or BMS should also provide the IRB/IEC with a copy of the Investigator Brochure or product labeling information to be provided to subjects and any updates.

The investigator, Sponsor or designee should provide the IRB/IEC with reports, updates and other information (e.g., expedited safety reports, amendments, and administrative letters) according to regulatory requirements or institution procedures.

COMPLIANCE WITH THE PROTOCOL AND PROTOCOL REVISIONS

The investigator should not implement any deviation or change to the protocol without prior review and documented approval/favorable opinion of an amendment from the IRB/IEC (and if applicable, also by local health authority) except where necessary to eliminate an immediate hazard(s) to study subjects.

If a deviation or change to a protocol is implemented to eliminate an immediate hazard(s) prior to obtaining relevant approval/favorable opinion(s) the deviation or change will be submitted, as soon as possible to:

- IRB/IEC for
- Regulatory Authority(ies), if applicable by local regulations (per national requirements)

Documentation of approval/favorable opinion signed by the chairperson or designee of the IRB(s)/IEC(s) and if applicable, also by local health authority must be sent to BMS.

If an amendment substantially alters the study design or increases the potential risk to the participant: (1) the consent form must be revised and submitted to the IRB(s)/IEC(s) for review and approval/favorable opinion; (2) the revised form must be used to obtain consent from subjects currently enrolled in the study if they are affected by the amendment; and (3) the new form must be used to obtain consent from new subjects prior to enrollment.

If the revision is done via an administrative letter, investigators must inform their IRB(s)/IEC(s).

FINANCIAL DISCLOSURE

Investigators and sub-Investigators will provide the Sponsor with sufficient, accurate financial information in accordance with local regulations to allow the Sponsor to submit complete and accurate financial certification or disclosure statements to the appropriate health authorities. Investigators are responsible for providing information on financial interests during the course of the study and for 1 year after completion of the study.

INFORMED CONSENT PROCESS

Investigators must ensure that subjects are clearly and fully informed about the purpose, potential risks, and other critical issues regarding clinical studies in which they volunteer to participate.

In situations where consent cannot be given to subjects, their legally acceptable representatives (as per country guidelines) are clearly and fully informed about the purpose, potential risks, and other critical issues regarding clinical studies in which the participant volunteers to participate.

Sponsor or designee will provide the investigator with an appropriate (i.e., Global or Local) sample informed consent form which will include all elements required by ICH, GCP and applicable regulatory requirements. The sample informed consent form will adhere to the ethical principles that have their origin in the Declaration of Helsinki.

Approved v 9.0 [redacted]

Investigators must:

- Provide a copy of the consent form and written information about the study in the language in which the participant is most proficient prior to clinical study participation. The language must be non-technical and easily understood.
- Allow time necessary for participant or participant's legally acceptable representative to inquire about the details of the study.
- Obtain an informed consent signed and personally dated by the participant or the participant's legally acceptable representative and by the person who conducted the informed consent discussion.
- Obtain the IRB/IEC's written approval/favorable opinion of the written informed consent form and any other information to be provided to the subjects, prior to the beginning of the study, and after any revisions are completed for new information.

If informed consent is initially given by a participant's legally acceptable representative or legal guardian, and the participant subsequently becomes capable of making and communicating his or her informed consent during the study, consent must additionally be obtained from the participant.

Revise the informed consent whenever important new information becomes available that is relevant to the participant's consent. The investigator, or a person designated by the investigator, should fully inform the participant or the participant's legally acceptable representative or legal guardian, of all pertinent aspects of the study and of any new information relevant to the participant's willingness to continue participation in the study. This communication should be documented.

The confidentiality of records that could identify subjects must be protected, respecting the privacy and confidentiality rules applicable to regulatory requirements, the subjects' signed ICF and, in the US, the subjects' signed HIPAA Authorization.

The consent form must also include a statement that BMS and regulatory authorities have direct access to participant records.

Subjects unable to give their written consent (e.g., stroke or subjects with or severe dementia) may only be enrolled in the study with the consent of a legally acceptable representative. The participant must also be informed about the nature of the study to the extent compatible with his or her understanding, and should this participant become capable, he or she should personally sign and date the consent form as soon as possible. The explicit wish of a participant who is unable to give his or her written consent, but who is capable of forming an opinion and assessing information to refuse participation in, or to be withdrawn from, the clinical study at any time should be considered by the investigator.

The rights, safety, and well-being of the study subjects are the most important considerations and should prevail over interests of science and society.

Approved v9.0 [redacted]

SOURCE DOCUMENTS

The Investigator is responsible for ensuring that the source data are accurate, legible, contemporaneous, original and attributable, whether the data are hand-written on paper or entered electronically. If source data are created (first entered), modified, maintained, archived, retrieved, or transmitted electronically via computerized systems (and/or any other kind of electronic devices) as part of regulated clinical trial activities, such systems must be compliant with all applicable laws and regulations governing use of electronic records and/or electronic signatures. Such systems may include, but are not limited to, electronic medical/health records (EMRs/EHRs), adverse event tracking/reporting, protocol required assessments, and/or drug accountability records).

When paper records from such systems are used in place of electronic format to perform regulated activities, such paper records should be certified copies. A certified copy consists of a copy of original information that has been verified, as indicated by a dated signature, as an exact copy having all of the same attributes and information as the original.

STUDY TREATMENT RECORDS

Records for study treatments (whether supplied by BMS, its vendors, or the site) must substantiate study treatment integrity and traceability from receipt, preparation, administration, and through destruction or return. Records must be made available for review at the request of BMS/designee or a Health Authority.

If	Then
Supplied by BMS (or its vendors):	Records or logs must comply with applicable regulations and guidelines and should include:<br>• amount received and placed in storage area<br>• amount currently in storage area<br>• label identification number or batch number<br>• amount dispensed to and returned by each participant, including unique participant identifiers<br>• amount transferred to another area/site for dispensing or storage<br>• nonstudy disposition (e.g., lost, wasted)<br>• amount destroyed at study site, if applicable<br>• amount returned to BMS<br>• retain samples for bioavailability/bioequivalence, if applicable<br>• dates and initials of person responsible for Investigational Product dispensing/accountability, as per the Delegation of Authority Form.
Sourced by site, and not supplied by BMS or its vendors (examples include IP sourced from the sites stock or commercial supply, or a specialty pharmacy)	The investigator or designee accepts responsibility for documenting traceability and study drug integrity in accordance with requirements applicable under law and the SOPs/standards of the sourcing pharmacy.<br>These records should include:

Approved v9.0 [redacted]

If	Then
	• label identification number or batch number<br>• amount dispensed to and returned by each participant, including unique participant identifiers<br>• dates and initials of person responsible for Investigational Product dispensing/accountability, as per the Delegation of Authority Form.

BMS or designee will provide forms to facilitate inventory control if the investigational site does not have an established system that meets these requirements.

CASE REPORT FORMS

An investigator is required to prepare and maintain adequate and accurate case histories designed to record all observations and other data pertinent to the investigation on each individual treated or entered as a control in the investigation. Data that are derived from source documents and reported on the CRF must be consistent with the source documents or the discrepancies must be explained. Additional clinical information may be collected and analyzed in an effort to enhance understanding of product safety. CRFs may be requested for AEs and/or laboratory abnormalities that are reported or identified during the course of the study.

For sites using the Sponsor or designee electronic data capture tool, electronic CRFs will be prepared for all data collection fields except for fields specific to SAEs and pregnancy, which will be reported on the electronic SAE form and Pregnancy Surveillance form, respectively. If electronic SAE form is not available, a paper SAE form can be used. Spaces may be left blank only in those circumstances permitted by study-specific CRF completion guidelines provided by Sponsor or designee.

The confidentiality of records that could identify subjects must be protected, respecting the privacy and confidentiality rules in accordance with the applicable regulatory requirement(s).

The investigator will maintain a signature sheet to document signatures and initials of all persons authorized to make entries and/or corrections on CRFs.

The completed CRF, SAE/pregnancy CRFs, must be promptly reviewed, signed, and dated by the investigator or qualified physician who is a subinvestigator and who is delegated this task on the Delegation of Authority Form. Subinvestigators in Japan may not be delegated the CRF approval task For electronic CRFs, review and approval/signature is completed electronically through the BMS electronic data capture tool. The investigator must retain a copy of the CRFs including records of the changes and corrections.

Each individual electronically signing electronic CRFs must meet Sponsor or designee training requirements and must only access the BMS electronic data capture tool using the unique user account provided by Sponsor or designee. User accounts are not to be shared or reassigned to other individuals

MONITORING

Sponsor or designee representatives will review data centrally to identify potential issues to determine a schedule of on-site visits for targeted review of study records.

Approved v9.0 [redacted]

Representatives of BMS must be allowed to visit all study site locations periodically to assess the data quality and study integrity. On site they will review study records and directly compare them with source documents, discuss the conduct of the study with the investigator, and verify that the facilities remain acceptable.

In addition, the study may be evaluated by Sponsor or designee internal auditors and government inspectors who must be allowed access to CRFs, source documents, other study files, and study facilities. BMS audit reports will be kept confidential.

The investigator must notify BMS promptly of any inspections scheduled by regulatory authorities, and promptly forward copies of inspection reports to Sponsor or designee.

RECORDS RETENTION

The investigator (or head of the study site in Japan) must retain all study records and source documents for the maximum period required by applicable regulations and guidelines, or institution procedures, or for the period specified by BMS or designee, whichever is longer. The investigator (or head of the study site in Japan) must contact BMS prior to destroying any records associated with the study.

BMS or designee will notify the investigator (or head of the study site in Japan) when the study records are no longer needed.

If the investigator withdraws from the study (e.g., relocation, retirement), the records shall be transferred to a mutually agreed upon designee (e.g., another investigator, study site, IRB). Notice of such transfer will be given in writing to BMS or designee.

RETURN OF STUDY TREATMENT

For this study, study treatments (those supplied by BMS, a vendor or sourced by the investigator) such as partially used study treatment containers, vials and syringes may be destroyed on site.

If..	Then
Study treatments supplied by BMS (including its vendors	Any unused study treatments supplied by BMS can only be destroyed after being inspected and reconciled by the responsible Study Monitor unless study treatments containers must be immediately destroyed as required for safety, or to meet local regulations (e.g., cytotoxics or biologics).<br>If study treatments will be returned, the return will be arranged by the responsible Study Monitor.
Study treatments sourced by site, not supplied by BMS (or its vendors) (examples include study treatments sourced from the sites stock or commercial supply, or a specialty pharmacy)	It is the investigator’s or designee’s responsibility to dispose of all containers according to the institutional guidelines and procedures.

It is the investigator’s or designee’s responsibility to arrange for disposal, provided that procedures for proper disposal have been established according to applicable federal, state, local, and

Approved v9.0 [redacted]

institutional guidelines and procedures, and provided that appropriate records of disposal are kept. The following minimal standards must be met:

- On-site disposal practices must not expose humans to risks from the drug.
- On-site disposal practices and procedures are in agreement with applicable laws and regulations, including any special requirements for controlled or hazardous substances.
- Written procedures for on-site disposal are available and followed. The procedures must be filed with the site's SOPs and a copy provided to BMS upon request.
- Records are maintained that allow for traceability of each container, including the date disposed of, quantity disposed, and identification of the person disposing the containers. The method of disposal, i.e., incinerator, licensed sanitary landfill, or licensed waste disposal vendor must be documented.
- Accountability and disposal records are complete, up-to-date, and available for the Monitor to review throughout the clinical trial period.

It is the investigator's or designee's responsibility to arrange for disposal of all empty containers.

If conditions for destruction cannot be met the responsible Study Monitor will make arrangements for return of study treatments provided by BMS (or its vendors). Destruction of non- study treatments sourced by the site, not supplied by BMS, is solely the responsibility of the investigator or designee.

CLINICAL STUDY REPORT AND PUBLICATIONS

A Signatory Investigator must be selected to sign the clinical study report.

For this protocol, the Signatory Investigator will be selected as appropriate based on the following criteria:

- External Principal Investigator designated at protocol development
- National Coordinating Investigator
- Study Steering Committee chair or their designee
- Participant recruitment (e.g., among the top quartile of enrollers)
- Involvement in trial design
- Regional representation (e.g., among top quartile of enrollers from a specified region or country)
- Other criteria (as determined by the study team)

The data collected during this study are confidential and proprietary to Sponsor or designee. Any publications or abstracts arising from this study must adhere to the publication requirements set forth in the clinical trial agreement (CTA) governing [Study site or Investigator] participation in the study. These requirements include, but are not limited to, submitting proposed publications to Sponsor or designee at the earliest practicable time prior to submission or presentation and otherwise within the time period set forth in the CTA.

Approved v 9.0 [redacted]

APPENDIX 8 ADVERSE EVENTS AND SERIOUS ADVERSE EVENTS: DEFINITIONS AND PROCEDURES FOR RECORDING, EVALUATING, FOLLOW UP AND REPORTING ADVERSE EVENTS

**Adverse Event Definition:**
An Adverse Event (AE) is defined as any new untoward medical occurrence or worsening of a preexisting medical condition in a clinical investigation participant administered study treatment and that does not necessarily have a causal relationship with this treatment.
An AE can therefore be any unfavorable and unintended sign (such as an abnormal laboratory finding), symptom, or disease temporally associated with the use of study treatment, whether or not considered related to the study treatment.
**Events Meeting the AE Definition**
• Any abnormal laboratory test results (hematology, clinical chemistry, or urinalysis) or results from other safety assessments (eg, ECG, radiological scans, vital signs measurements), including those that worsen from baseline, considered clinically significant in the medical and scientific judgment of the investigator. Note that abnormal lab tests or other safety assessments should only be reported as AEs if the final diagnosis is not available. Once the final diagnosis is known, the reported term should be updated to be the diagnosis.<br>• Exacerbation of a chronic or intermittent pre-existing condition including either an increase in frequency and/or intensity of the condition.<br>• New conditions detected or diagnosed after study intervention administration even though it may have been present before the start of the study.<br>• Signs, symptoms, or the clinical sequelae of a suspected drug-drug interaction.<br>• Signs, symptoms, or the clinical sequelae of a suspected overdose of either study intervention or a concomitant medication. Overdose, as a verbatim term (as reported by the investigator), should not be reported as an AE/SAE unless it is an intentional overdose taken with possible suicidal/self-harming intent. Such overdoses should be reported regardless of sequelae and should specify "intentional overdose" as the verbatim term
**Events NOT Meeting the AE Definition**
• Medical or surgical procedure (eg, endoscopy, appendectomy): the condition that leads to the procedure is the AE.<br>• Situations in which an untoward medical occurrence did not occur (social and/or convenience admission to a hospital).

DEFINITION OF SAE

If an event is not an AE per definition above, then it cannot be an SAE even if serious conditions are met.

SERIOUS ADVERSE EVENTS

**Serious Adverse Event (SAE) is defined as any untoward medical occurrence that, at any dose:**
Results in death
Is life-threatening (defined as an event in which the participant was at risk of death at the time of the event; it does not refer to an event which hypothetically might have caused death if it were more severe)
Requires inpatient hospitalization or causes prolongation of existing hospitalization (see NOTE below)<br>NOTE:<br>The following hospitalizations are not considered SAEs in BMS clinical studies:<br>• a visit to the emergency room or other hospital department < 24 hours, that does not result in admission (unless considered an important medical or life-threatening event)<br>• elective surgery, planned prior to signing consent<br>• admissions as per protocol for a planned medical/surgical procedure<br>• routine health assessment requiring admission for baseline/trending of health status (e.g., routine colonoscopy)<br>• medical/surgical admission other than to remedy ill health and planned prior to entry into the study. Appropriate documentation is required in these cases<br>• admission encountered for another life circumstance that carries no bearing on health status and requires no medical/surgical intervention (e.g., lack of housing, economic inadequacy, caregiver respite, family circumstances, administrative reason)<br>• admission for administration of anticancer therapy in the absence of any other SAEs (applies to oncology protocols)
Results in persistent or significant disability/incapacity
Is a congenital anomaly/birth defect
Is an important medical event (defined as a medical event(s) that may not be immediately life-threatening or result in death or hospitalization but, based upon appropriate medical and scientific judgment, may jeopardize the participant or may require intervention [e.g., medical, surgical] to prevent one of the other serious outcomes listed in the definition above.) Examples of such events include, but are not limited to, intensive treatment in an emergency room or at home for allergic bronchospasm; blood dyscrasias or convulsions that do not result in hospitalization.) Potential drug induced liver injury (DILI) is also considered an important medical event. (See Section 9.2.7 for the definition of potential DILI.)

Pregnancy and potential drug induced liver injury (DILI) must follow the same transmission timing and processes to BMS as used for SAEs (see section 9.2.5 for reporting pregnancies).

Approved v 9.0 [redacted]

Any component of a study endpoint that is considered related to study therapy should be reported as SAE (e.g., death is an endpoint, if death occurred due to anaphylaxis, anaphylaxis must be reported).

EVALUATING AES AND SAES

**Assessment of Causality**
• The investigator is obligated to assess the relationship between study intervention and each occurrence of each AE/SAE.<br>• A "reasonable possibility" of a relationship conveys that there are facts, evidence, and/or arguments to suggest a causal relationship, rather than a relationship cannot be ruled out.<br>• The investigator will use clinical judgment to determine the relationship.<br>• Alternative causes, such as underlying disease(s), concomitant therapy, and other risk factors, as well as the temporal relationship of the event to study intervention administration will be considered and investigated.<br>• The investigator will also consult the Investigator's Brochure (IB) and/or Product Information, for marketed products, in his/her assessment.<br>• For each AE/SAE, the investigator must document in the medical notes that he/she has reviewed the AE/SAE and has provided an assessment of causality.<br>• There may be situations in which an SAE has occurred and the investigator has minimal information to include in the initial report to Sponsor. However, it is very important that the investigator always make an assessment of causality for every event before the initial transmission of the SAE data to Sponsor.<br>• The investigator may change his/her opinion of causality in light of follow-up information and send a SAE follow-up report with the updated causality assessment.<br>• The causality assessment is one of the criteria used when determining regulatory reporting requirements.

**Follow-up of AEs and SAEs**
If only limited information is initially available, follow-up reports are required. (Note: Follow-up SAE reports must include the same investigator term(s) initially reported.)<br>If an ongoing SAE changes in its intensity or relationship to study treatment or if new information becomes available, the SAE report must be updated and submitted within 24 hours to BMS (or designee) using the same procedure used for transmitting the initial SAE report.<br>All SAEs must be followed to resolution or stabilization.

Approved v9.0 [redacted]

REPORTING OF SAES TO SPONSOR OR DESIGNEE

- SAEs, whether related or not related to study treatment, and pregnancies must be reported to BMS (or designee) immediately within 24 hours of awareness of the event.
- SAEs must be recorded on the SAE Report Form.
  - The required method for SAE data reporting is through the eCRF.
  - The paper SAE Report Form is only intended as a back-up option when the electronic data capture (EDC) system is unavailable/not functioning for transmission of the eCRF to BMS (or designee).
    - In this case, the paper form is transmitted via email or confirmed facsimile (fax) transmission
    - When paper forms are used, the original paper forms are to remain on site
- Pregnancies must be recorded on a paper Pregnancy Surveillance Form and transmitted via email or confirmed facsimile (fax) transmission

SAE Email Address: Refer to Contact Information list.

SAE Facsimile Number: Refer to Contact Information list.

SAE Telephone Contact (required for SAE and pregnancy reporting): Refer to Contact Information list

Approved v 9.0 [redacted]

APPENDIX 9 REVISED PROTOCOL SUMMARY OF CHANGE HISTORY

OVERALL RATIONALE FOR REVISED PROTOCOL 06, 14-Jul-2020

During recent interactions with health authorities (2020), they indicated that the early event-free survival (EFS) interim analysis (IA) may lack robustness due to immature data. Accordingly, the EFS IA plan was changed in protocol revision 06 to remove the first planned IA.

SUMMARY OF KEY CHANGES OF REVISED PROTOCOL 06		
**Section Number and Name**	**Description of Change**	**Brief Rationale**
Synopsis, Objectives and Endpoints<br>Table 4-1 Objectives and Endpoints<br>Table 10.3.1 Efficacy Analyses	Clarified that any progression precluding surgery is an EFS event and that RECIST 1.1 progression/recurrence per blinded independent central review (BICR) applies post surgery or for participants without surgery	Clarified EFS definition
Synopsis, Number of Participants<br>5.2 Number of Participants	Corrected the number of participants	To align with 10.1 Sample Size Determination
5.3 End of Study Definition<br>10.1.2 Event Free Survival<br>10.1.3 Power Consideration for Overall Survival (OS)<br>Table 10.1.3-1 Power Calculation for EFS and OS<br>10.1.4 Analyses Timing Projections<br>Table 10.1.4-1 Scheduled Analyses, Criteria, and Projected Timelines<br>10.3.3 Interim Analyses	Removed the first of 2 IA of EFS and updated alpha spending on the remaining single interim and final analyses of EFS	Health authorities suggested that the early EFS IA may lack robustness at that level of data maturity
[redacted]		
10.1.2 Event Free Survival<br>10.1.4 Analyses Timing Projections<br>Table 10.1.4-1 Scheduled Analyses, Criteria, and Projected Timelines	Clarified that actual timing of analyses may differ from projected timing	Clarified potential variation in analyses timing
10.1.2 Event Free Survival	Removed text about descriptive EFS analysis	Descriptive analyses for the DMC will be described in the DMC Charter
All	Corrected typographical errors	Minor, so not specified

Approved v 1.0 [redacted]

OVERALL RATIONALE FOR REVISED PROTOCOL 05, 18-Sep-2019

The revised protocol updates analyses projections for pCR and EFS and clarifications of surgery exploratory endpoints, instruction for biomarker collection and pathology review, and rules for PD-L1 stratification. Additionally, management algorithms were updated for myocarditis.

SUMMARY OF KEY CHANGES OF REVISED PROTOCOL 05		
**Section Number and Name**	**Description of Change**	**Brief Rationale**
Synopsis<br>Section 4 Objectives and Endpoints	Updated exploratory endpoint to include completeness of resection in surgical approach	Update provides clarity on collection of surgical outcomes
Synopsis<br>Section 5.1 Overall Design	Updated stratification of participants with PD-L1 not evaluable/ indeterminate status to PD-L1 negative participants	Update provides clarity for stratification of participants with PD-L1 not evaluable/ indeterminate status
Synopsis	Language updated to identify the CA209816 Laboratory Manual as a source for pathology specimen collection and processing. Additionally, blinded pathology review process will assess for confirmation of endpoints.	Updates provide instruction for guidelines for collection and processing of pathology specimens and confirmation of endpoints
Section 5.3 End of Study Definitions	Updated the timing of pCR rate	Provides updated projection of analysis for pCR rate.
[redacted]<br>Section 9.8.1.1 Tumor Tissue Specimens	Clarified tumor biopsy collection will not be collected in China	Sites are unable to collect optional tumor biopsy in China.
Section 10.1 Sample Size Determination<br>Section 10.1.1 Pathologic Complete Response (pCR)<br>Sections 10.1.4 Analyses Timing Projections<br>Section 10.2 Populations for Analyses<br>Section 10.3.1 Efficacy Analyses<br>Section 10.3.3 Interim Analyses	Updates anticipated sample size and projected timing of analyses for pCR and EFS	Provides updated information for analysis for pCR and EFS..
Section 10.3.1 Efficacy Analyses	Updated statistical analysis methods for TTDM	Statistical analysis methods for TTDM were updated.
Appendix 4 Management Algorithms	Updated management algorithms to include myocarditis	Aligns management algorithms with updated Investigator Brochure

Approved v 1.0 [redacted]

OVERALL RATIONALE FOR REVISED PROTOCOL 04, 25-Jun-2019

The CA209816 revised protocol 04 adds EFS on next line of therapy as an exploratory objective. Statistical analyses, power calculations, projected timelines [redacted]. Additional changes were added for clarity.

SUMMARY OF KEY CHANGES OF REVISED PROTOCOL 04		
**Section Number and Name**	**Description of Change**	**Brief Rationale**
Synopsis<br>Table 4-1: Objectives and Endpoints	Added exploratory endpoint of Event Free Survival on next line of therapy	Added EFS on next line of therapy, as provides additional follow-up information for efficacy and survival
Section 7.7.1 Prohibited and/or Restricted Treatments	Added the concomitant administration of substances that are also tubularly secreted (eg, probenecid) could potentially result in delayed clearance of pemetrexed.	Added to comply with HA request
Section 10.2.1 Populations for Analysis at Pathologic Complete Response Analysis Timepoint	Clarified the pCR analysis population	Updated section for clarity
[redacted]		
Synopsis<br>Section 5.1 Overall Design<br>Section 5.1.1 Data Monitoring Committee and Other External Committees	• BICR confirmation of progression should not be requested if investigator judges the progression will not preclude surgery.<br>• Tumor assessments (if clinically feasible) and BICR should continue even after the initiation of subsequent anti-cancer therapies.	Updated parameters for BICR confirmation for clarity
Section 10.1.3 Power Consideration for Overall Survival (OS)<br>Table 10.1.3-1: Power Calculation for EFS and OS<br>Section 10.3.3 Interim Analyses	Added power consideration for OS testing	In case of significant EFS, OS will be tested.
Section 10.1.4 Analyses Timing Projections<br>Table 10.1.3-1 Scheduled Analyses, Criteria, and Projected Timelines	Updated the analysis timing projections	Updated the analysis timing projections
Appendix 8 Adverse Events and Serious Adverse Events: Definitions and Procedures for Recording, Evaluating, Follow Up and Reporting Adverse Events	Updated AE template language	Updated appendix to align with program standards

Approved v 1.0 [redacted]

OVERALL RATIONALE FOR REVISED PROTOCOL 03, 21-Sep-2018

The CA209816 revised protocol closes enrollment in Arm A (nivolumab plus ipilimumab) and expands the sample size in Arm B (chemotherapy) and Arm C (nivolumab plus chemotherapy). Subsequently, the study rationale and statistical section was updated to support the new analyses. Additional changes were made to provide clarity in study conduct.

SUMMARY OF KEY CHANGES OF REVISED PROTOCOL 03		
**Section Number and Name**	**Description of Change**	**Brief Rationale**
Synopsis, Objectives, Overall Design, Schema, Number of Participants<br>Section 4 Objectives and Endpoints<br>Section 5.1 Overall Design<br>Section 5.2 Number of Participants	Enrollment in Arm A (nivolumab plus ipilimumab) was stopped and randomization of participants (1:1 ratio) into Arm B and C will continue enrolling for a total of 350 participants	Study design was changed to support nivolumab plus chemotherapy as neoadjuvant therapy in NSCLC based on emerging data.
Synopsis, Objectives<br>Section 4 Objectives and Endpoints	• Updated objectives for assessments of Arm B to Arm C<br>• Added time to death or distant metastases (TTDM) as secondary objective<br>• Updated tertiary objectives for Arm A (nivolumab and ipilimumab)	Objectives were updated to align with change in study design
Synopsis Overall Design	Participants with large-cell neuroendocrine carcinoma tumor histology are excluded	Eligibility criteria was updated as protocol's chemotherapy regimens are not optimal for treating this specific subtype of NSCLC
Synopsis Treatment Arms and Duration, Study Treatment<br>Section 5.1 Overall Design<br>Section 5.4.6 Rationale for Choices of Platinum-based Chemotherapy Doublet<br>Table 7-1 Study Treatments for CA209816<br>Section 7.1 Treatments Administered<br>Table 7.1-1 Selection and Timing of Doses<br>Section 7.4 Dose Modifications	Additional platinum doublet chemotherapy regimen (paclitaxel/carboplatin) was added	Additional platinum doublet chemotherapy regimens (paclitaxel/carboplatin) added to reflect knowledge gained from emerging data.
Section 7.4.1.1 Platinum Doublet Chemotherapy - Dose Reductions for Hematologic Toxicity<br>Section 7.4.1.2 Platinum Doublet Chemotherapy - Dose Reductions for Non-Hematologic Toxicities	Updated and moved dose modification for docetaxel into hematologic toxicity and non-hematologic toxicity sections.	Updated dose modification for docetaxel into correct sections of protocol.
Section 7.4.2.2 Dose Delay Criteria for Platinum Doublet Chemotherapy	Provided updated dose delay criteria for chemotherapy	Provided updated dose delay criteria for safety

Approved v 9.0 [redacted]

SUMMARY OF KEY CHANGES OF REVISED PROTOCOL 03		
**Section Number and Name**	**Description of Change**	**Brief Rationale**
Table 2-3 Post Neoadjuvant Period for Those Participants Not Receiving Adjuvant Therapy<br>Section 9.1.1 Imaging Assessment for the Study	• Updated time relationship between adjuvant radiotherapy and tumor imaging assessments<br>• Clarified imaging for tumor assessments for participants who do not proceed to definitive surgery	Updated imaging assessment requirements for clarity
Section 5.3 End of Study Definition<br>Section 10.1 Sample Size Determination<br>Section 10.1.1 Pathologic Complete Response (pCR)<br>Section 10.1.2 Event Free Survival (EFS)<br>Section 10.1.3 Analyses Timing Projections<br>Section 10.3.1 Efficacy Analyses<br>Section 10.3.3 Interim Analyses	Statistical analysis plan was updated	Statistical analyses were updated to align with change in study design.
Section 3.2.1 Indication Background<br>Section 3.2.4 Nivolumab Combined with Ipilimumab Clinical Activity<br>Section 3.2.5 Immuno-oncology Treatment Combined with Chemotherapy Clinical Activity<br>Section 3.3 Benefit/Risk Assessment<br>Section 5.4.3 Rationale for Immuno-oncology Treatment in Neoadjuvant NSCLC<br>Section 5.4.3.1 Rationale for Combination of Nivolumab and Ipilimumab (Arm A)<br>Section 5.4.3.2 Rationale for PD-1 Inhibitor with Chemotherapy (Arm C)<br>Section 5.4.4 Rationale for Pathologic Response	Rationale, background information, and trial schematic were updated	Rationale, background information, and trial schematic were updated to align with changes in study design and current data.
Table 2-1 Screening Procedural Outline<br>Table 2-2 Neoadjuvant Period Procedural Outline	Updated to include FVC, FEV1, TLC, FRC, and DLco.	Parameters of pulmonary function tests to be collected were clarified.
Section 9.5 Pharmacokinetics	Time window of Cycle 1 Day 1 end-of-infusion PK sampling for Arm A and C was clarified	Time window for certain PK sample collection was provided.
Appendix 4 Hepatic Adverse Event Management Algorithm	Footnote stating I-O therapy may be delayed rather than discontinued if AST/ALT ≤ 8 x ULN or T.bili ≤ 5 x ULN was removed.	Language was modified to align protocol with current Nivolumab Investigator Brochure and nivolumab program safety management principals.

Approved v 9.0 [redacted]

OVERALL RATIONALE FOR REVISED PROTOCOL 02, 06-JUL-2017

The CA209816 study has been revised in response to a health authority request to add the primary objective of event-free survival for the study population. In addition, a new treatment arm was added to the study as a new comparison for the current treatment landscape.

This revised protocol applies to all current and future participants.

SUMMARY OF KEY CHANGES OF REVISED PROTOCOL 02

Section Number and Name	Description of Change	Brief Rationale
Title; Section 1 Synopsis, Objective and Endpoints, Study Design, Number of Participants, Treatment Arms and Duration; Section 2 Schedule of Activities; Section 3.1 Study Rationale, Research Hypothesis; Section 4 Objectives and Endpoints; Section 5 Study Design; Section 5.2 Number of Participants; Section 5.3 End of Study Definition; Section 6 Study Population; Section 7 Treatment; Section 9.5 Pharmacokinetics; Section 10 Statistical Considerations (Sections 10.1.1, 10.1.2, 10.1.3, 10.2, 10.3, 10.3.3)	A new treatment arm of nivolumab plus platinum doublet chemotherapy (Arm C) was added to the study. The sample size was increased to 642 with the addition of the new treatment arm.	New treatment arm was added for current treatment landscape.
Title, Section 1 Synopsis, Objective and Endpoints, Study Design, Number of Participants, Treatment Arms and Duration; Section 2 Schedule of Activities' Section 3.1 Study Rationale; Section 3.2; Section 4 Objectives and Endpoints; Section 5 Study Design (Section 5.1, Schema, Section 5.2, Number of participants, Section 5.3 End of Study Definition; Section 9.1 Efficacy Assessments; Section 10 Statistical Considerations (Sections 10.1.1, 10.1.2, 10.1.3, 10.2 Populations for Analyses, 10.3 Statistical Analyses, 10.3.3)	Primary objectives were modified to event free survival and pathological response.	New multiple primary objective was added in response to health authority request.
Synopsis, Background Section 3.2 (Sections 3.2.4, 3.2.5); Section 3.3 Benefit/Risk Assessment, Background, Section 5.4 Scientific Rationale for Study Design (Sections 5.4.3, 5.4.4, 5.5.2)	With the addition of new treatment arm and new primary endpoints, the background and rationale were updated with current safety and efficacy data.	Study sections were updated to reflect current study design and available data on safety and efficacy of study treatments.

STATISTICAL ANALYSIS PLAN
FOR CLINICAL STUDY REPORT

RANDOMIZED, OPEN-LABEL, PHASE 3 TRIAL OF NIVOLUMAB PLUS IPILIMUMAB OR NIVOLUMAB PLUS PLATINUM-DOUBLET CHEMOTHERAPY VERSUS PLATINUM-DOUBLET CHEMOTHERAPY IN EARLY STAGE NSCLC

PROTOCOL(S) CA209816

VERSION # 1.0

DATE: 17-APR-2020

TABLE OF CONTENTS

STATISTICAL ANALYSIS PLAN FOR CLINICAL STUDY REPORT 1
TABLE OF CONTENTS 2
LIST OF TABLES 6
LIST OF FIGURES 7
1 BACKGROUND AND RATIONALE....8
2 STUDY DESCRIPTION 9
2.1 Study Design 9
2.2 Treatment Assignment....12
2.3 Blinding and Unblinding....13
2.4 Protocol Amendments....13
2.5 Data Monitoring and Other External Committees 16
3 OBJECTIVES 16
3.1 Primary....16
3.2 Secondary....16
3.3 Exploratory Objectives 16
4 ENDPOINTS....17
4.1 Primary Endpoint(s) 17
4.1.1 Event-Free Survival 18
4.1.1.1 Primary Definition of Event-Free Survival....18
4.1.1.2 Secondary Definition of Event-Free Survival 19
4.1.2 Pathologic Complete Response Rate....21
4.2 Secondary Endpoint(s)....21
4.2.1 Overall Survival 21
4.2.2 Major Pathological Response Rate....21
4.2.3 Time to Death or Distant Metastases 22
4.3 Exploratory Endpoint(s)....22
4.3.1 Clinical Response Rate by BICR....22
4.3.2 Event Free Survival on Next Line of Therapy (EFS2)....22
4.3.3 Surgery Related Endpoints....23
4.3.4 Safety and Tolerability....23
4.3.5 Pharmacokinetics 23
4.3.6 Biomarkers 23
4.3.6.1 Tumor Mutational Burden 23
4.3.6.2 PD-L1 Protein Expression....24
4.3.7 Outcomes Research 24
4.3.7.1 EQ-5D-3L....24
5 SAMPLE SIZE AND POWER....26
5.1 Pathologic Complete Response (pCR) 27
5.2 Event Free Survival (EFS) 28
5.3 Power Considerations for Overall Survival 28
5.4 Analyses Timing Projections 30
6 STUDY PERIODS, TREATMENT REGIMENS AND POPULATIONS FOR ANALYSES 33
6.1 Study Periods....33
6.2 Treatment Regimens....34

Approved v1.0 [redacted]

6.3 Populations for Analyses 35
7 STATISTICAL ANALYSES....36
7.1 General Methods 36
7.1.1 Adverse Events, Serious Adverse Events, Multiple Events, Select Adverse Events, Other Events of Special Interest and Immune-Mediated Adverse Events....37
7.1.1.1 Select Adverse Events 38
7.1.1.2 Other Events of Special Interest....38
7.1.1.3 Immune-Mediated Adverse Events 38
7.1.2 Laboratory Tests....39
7.1.3 Immunogenicity Data....39
7.2 Study Conduct 39
7.2.1 Accrual....39
7.2.2 Relevant Protocol Deviations....39
7.3 Study Population 40
7.3.1 Subject Disposition....40
7.3.2 Demographics and Other Baseline Characteristics....40
7.3.3 Medical History....41
7.3.4 Prior Therapy....42
7.3.5 Physical Examinations....42
7.3.6 Baseline Physical Measurements 42
7.3.7 Consistency of Demographics and Baseline Characteristics by Randomization Period 42
7.4 Extent of Exposure 42
7.4.1 Administration of Study Therapy....42
7.4.2 Modifications of Study Therapy 46
7.4.2.1 Dose Delay/Omission 46
7.4.2.2 Infusion Interruptions and Rate Changes....47
7.4.2.3 Dose Reductions....47
7.4.3 Concomitant Medications....48
7.4.3.1 Immune Modulating Medication 49
7.4.3.2 Subsequent Cancer Therapy 49
7.5 Definitive Surgery 50
7.6 Efficacy....50
7.6.1 Type I Error Control....51
7.6.2 Analysis of Pathological Complete Response....51
7.6.2.1 Primary pCR Analysis 51
7.6.2.2 Supportive Analyses of pCR....52
7.6.2.3 Subset Analyses of pCR....52
7.6.2.4 Major Pathological Response Rate....53
7.6.2.5 Additional Pathological Related Analyses....53
7.6.2.6 Clinical Response Rate....53
7.6.3 Analysis of Event Free Survival 54
7.6.3.1 Primary Event Free Survival 54
7.6.3.2 Supportive Analyses of Event-Free Survival....55
7.6.3.3 Subset Analyses of EFS....57

Approved v1.0 [redacted]

7.6.3.4 EFS Landmark Analyses by pCR and MPR Status 57
7.6.3.5 Current Status of EFS 57
7.6.4 Analysis of Overall Survival 57
7.6.4.1 OS Analyses 57
7.6.4.2 Supportive Analyses for OS 58
7.6.4.3 Subset Analyses of OS 59
7.6.4.4 OS Landmark Analyses by pCR and MPR Status 59
7.6.4.5 Subject Follow-Up 59
7.6.5 Interim Analyses of EFS and OS 59
7.6.6 Analysis of TTDM 60
7.6.7 Event Free Survival on Next Line of Therapy 60
7.6.8 Consistency of Efficacy by Randomization Period 60
7.7 Safety 61
7.7.1 Definitive Surgery Related Safety 61
7.7.2 Deaths 61
7.7.3 Serious Adverse Events 61
7.7.4 Adverse Events Leading to Discontinuation of Study Therapy 62
7.7.5 Adverse Events Leading to Dose Modification 62
7.7.6 Adverse Events 62
7.7.7 Select Adverse Events 63
7.7.7.1 Incidence of Select AE 63
7.7.7.2 Time-to Onset of Select AE 63
7.7.7.3 Time-to Resolution of Select AE 64
7.7.8 Immune-Mediated Adverse Events 64
7.7.9 Other Events of Special Interest 65
7.7.10 Multiple Events 65
7.7.11 New Primary Cancers 65
7.7.12 Laboratory Parameters 65
7.7.12.1 Hematology 66
7.7.12.2 Serum Chemistry 66
7.7.12.3 Electrolytes 66
7.7.12.4 Additional Analyses 66
7.7.13 Vital Signs and Pulse Oximetry 67
7.7.14 Physical Measurements 67
7.7.15 Non-Protocol Medical Procedures 67
7.7.16 Immunogenicity Analysis 67
7.7.17 Pregnancy 68
7.7.18 Adverse Events By Subgroup 68
7.7.19 Consistency of Safety by Randomization Period 69
7.8 Pharmacokinetics 69
7.9 Biomarkers 69
7.9.1 PD-L1 Expression 70
7.9.2 Tumor Mutational Burden (TMB) 70
7.10 Outcomes Research Analyses 71
8 CONVENTIONS 72
9 CONTENT OF REPORTS 73

10 DOCUMENT HISTORY 74
11 PREVIOUS ANALYSES 74
APPENDIX 1 TIME-TO ONSET AND TIME-TO RESOLUTION DEFINITION AND CONVENTIONS FOR SELECT ADVERSE EVENTS, IMMUNE-MEDIATED ADVERSE EVENTS AND EVENTS OF SPECIAL INTEREST 75
APPENDIX 2 MISSING AND PARTIAL RADIOTHERAPY AND SURGERY DATES IMPUTATION ALGORITHMS 77
APPENDIX 3 IMMUNOGENICITY ANALYSIS: BACKGROUND AND RATIONALE 79
12 REFERENCES 81

Approved v1.0 [redacted]

LIST OF TABLES

Table 2.4-1: Protocol Amendments 13

Table 4.3.7.1-1: Time Windows for EQ-5D-3L Assessments 25

Table 5.3-1: Power Calculation for EFS and OS 29

Table 5.4-1: Scheduled Analyses, Criteria, and Projected Timelines for Formal Analyses of pCR and EFS 31

Table 7.4.1-1: Study Therapy Parameter Definitions- Nivolumab and Ipilimumab 43

Table 7.4.1-2: Study Therapy Parameter Definitions - Regimen 1: Vinorelbine/Cisplatin 43

Table 7.4.1-3: Study Therapy Parameter Definitions - Regimen 2: Docetaxel/Cisplatin 44

Table 7.4.1-4: Study Therapy Parameter Definitions - Regimen 3: Gemcitabine/Cisplatin 45

Table 7.4.1-5: Study Therapy Parameter Definitions - Regimen 4: Pemetrexed/Cisplatin 45

Table 7.4.1-6: Study Therapy Parameter Definitions - Regimen 5: Paclitaxel/Carboplatin 46

Table 7.4.2.3-1: Dose Modifications of Chemotherapeutic Agents (Arms B and C) 47

Table 7.4.2.3-2: Calculated Dose Ranges and Related Dose Levels 48

Table 10-1: Document History 74

Table 11-1: Derivation of Clustered AE 76

Approved v1.0 [redacted]

LIST OF FIGURES

Figure 2.1-1: Study Design Schematic 12
Figure 4.1.1.1-1: EFS Primary Definition 19
Figure 4.1.1.2-1: EFS Secondary Definition 21

[redacted]

1 BACKGROUND AND RATIONALE

Approximately 80% of lung cancer cases are non-small cell lung cancer (NSCLC), with most patients presenting with late-stage disease. At initial diagnosis, 20% of patients present with stage I or II disease, whereas 30% present with stage III disease and 50% with stage IV disease. With enhanced lung cancer screening techniques, the percentage of patients diagnosed during the early stages may increase over the duration of the trial. A standard TNM staging system is used to determine the staging for NSCLC. Patients with pathologic stage I NSCLC have a 5-year survival of approximately 60%. Stage II to III NSCLC patients have a 5-year survival of approximately 25% to 40%.[1] Surgical resection remains the mainstay of treatment for stage I and II patients; however, despite potentially curative surgery, approximately 50% of stage IB and 60-75% of stage II NSCLC patients will relapse and eventually die of their disease.[2,3] A rational approach to improve survival in these patients is to eradicate micrometastatic disease and to minimize the risk of relapse after adjuvant or neoadjuvant chemotherapy.

The phase 3 study, CA209816, will evaluate the clinical efficacy and will establish the safety of nivolumab plus platinum doublet chemotherapy and nivolumab plus ipilimumab, in resectable lung cancer. Specifically, this study will compare EFS and pCR rate among participants treated with neoadjuvant nivolumab plus platinum doublet chemotherapy vs participants treated with platinum doublet chemotherapy, and will describe pCR rate and EFS for those treated with neoadjuvant nivolumab plus ipilimumab in Stage Ib-IIIa NSCLC.

This document contains description of the statistical analyses that will be conducted for the clinical study report (CSR) of study CA209816. This document also refers to Core Safety statistical analysis plan[4] that contains program level safety analyses description.

Research Hypothesis:

In participants with stage IB (≥ 4 cm), II or IIIA (N2) NSCLC considered resectable by the local multidisciplinary team, administration of neoadjuvant nivolumab plus platinum doublet chemotherapy (up to 3 cycles) has superior efficacy to neoadjuvant platinum doublet chemotherapy (up to 3 cycles).

Schedule of Analyses:

Formal analysis of Pathological Complete Response (pCR) will occur after the 350 randomized participants on Arms B and C from start of 1:1:1 randomization have an opportunity for surgery and is projected to occur approximately 30 months after 1:1:1 randomization.

Two formal interim analyses for Event Free Survival (EFS) are planned after 111 and 148 events have been observed on Arms B and C after start of 1:1:1 randomization, respectively. These are projected to occur approximately 42 and 54 months after start of 1:1:1 randomization. The formal comparisons of EFS will allow for early stopping for superiority. If the study continues beyond these interim analyses, the final analysis will be conducted after 185 EFS events have been observed on Arms B and C from start of 1:1:1 randomization.

[redacted]

2 STUDY DESCRIPTION

2.1 Study Design

This is an open-label, randomized clinical trial of up to 3 cycles of neoadjuvant nivolumab (3 mg/kg every 2 weeks) and a single dose of 1 mg/kg dose of ipilimumab, nivolumab 360mg flat dose plus platinum doublet chemotherapy (up to 3 cycles), or platinum doublet chemotherapy (up to 3 cycles) as neoadjuvant treatment in participants with early stage (Stage IB [≥ 4 cm], II, and resectable IIIA [N2]) NSCLC.

The original study design (before revised protocol 02) had two arms. After signing the informed consent form and upon confirmation of the participant's eligibility, participants were randomized in an open-label fashion (1:1 ratio) to either neoadjuvant nivolumab plus ipilimumab or platinum doublet chemotherapy.

Revised protocol 02 added a new, neoadjuvant nivolumab plus platinum doublet chemotherapy arm. When the third arm had opened and as each site had received IRB/EC approval of revised protocol 02, the interactive response technology IRT switched to a 1:1:1 randomization at the respective site. Starting from that point on, the sites were only enrolling under revised protocol 02.

Revised protocol 03 withholds randomization into the arm of neoadjuvant nivolumab plus ipilimumab but continues randomizing eligible participants into either neoadjuvant nivolumab plus platinum doublet chemotherapy arm or platinum doublet chemotherapy arm. Participants already randomized in the original 2-arm part (neoadjuvant nivolumab plus ipilimumab vs neoadjuvant chemotherapy) and in the arm of neoadjuvant nivolumab plus ipilimumab in 3-arm part defined by revised protocol 02 will remain in trial and continue scheduled trial procedures. The primary population for comparisons of the primary endpoints is the subjects concurrently randomized in arms B and C (as of revised protocol 02).

As of Revised protocol 03, participants will be randomized between 2 arms in a 1:1 ratio to neoadjuvant nivolumab plus platinum doublet chemotherapy or platinum doublet chemotherapy. Eligible participants will be stratified by:

- PD-L1 expression (≥1% or <1%/not evaluable/indeterminate)
- Disease stage (IB/II vs IIIA)
- Gender

The treatment arms are as follows:

Arm A treatment: Participants randomized into Arm A received nivolumab 3 mg/kg IV over 30 minutes every 2 weeks for up to 3 doses (ie, 6 weeks of treatment; each cycle is 14 days). With Cycle 1 only, nivolumab was followed by a single dose ipilimumab 1 mg/kg IV over 30 minutes.

Arm B treatment: Participants randomized into Arm B will receive investigator-choice platinum doublet chemotherapy in 3-week cycles up to a maximum of 3 cycles (ie, 9 weeks of treatment; each cycle is 21 days):

- Regimen 1:
  - Vinorelbine 25 mg/m^2 or 30 mg/m^2 IV (per local prescribing information) push over 10 minutes or per institutional standard on Days 1 and 8
  - Cisplatin 75 mg/m^2 IV over 120 minutes or per institutional standard on Day 1, immediately following vinorelbine
- Regimen 2:
  - Docetaxel 60 mg/m^2 or 75 mg/m^2 IV (per local prescribing information) over 60 minutes or per institutional standard on Day 1
  - Cisplatin 75 mg/m^2 IV over 120 minutes or per institutional standard on Day 1, immediately following docetaxel
- Regimen 3 (squamous histology):
  - Gemcitabine 1000 mg/m^2 or 1250 mg/m^2 (per local prescribing information) IV over 30 minutes or per institutional standard on Days 1 and 8
  - Cisplatin 75 mg/m^2 IV over 120 minutes or per institutional standard on Day 1, immediately following gemcitabine
- Regimen 4 (non-squamous histology only):
  - Pemetrexed 500 mg/m^2 IV over 10 minutes or per institutional standard on Day 1
  - Cisplatin 75 mg/m^2 IV over 120 minutes or per institutional standard on Day 1, immediately following pemetrexed
- Regimen 5:
  - Paclitaxel 175 or 200 mg/m^2 IV over 180 minutes or per institutional standard on Day 1
  - Carboplatin AUC 5 or 6 IV over 30 minutes or per institutional standard on Day 1, immediately following paclitaxel

Arm C treatment: Participants randomized into Arm C will receive nivolumab 360 mg IV plus platinum doublet chemotherapy in 3-week cycles up to a maximum of 3 cycles of chemotherapy (ie, 9 weeks of treatment; each cycle is 21 days)

- Non-squamous NSCLC: nivolumab at a flat dose of 360 mg as 30-minute IV infusion on Day 1, followed by pemetrexed at a dose of 500 mg/m^2 IV over 10 minutes or per institutional standard and cisplatin at a dose of 75 mg/m^2 IV over 120 minutes or per institutional standard of a 3-week treatment cycle, for up to 3 cycles.

- Squamous NSCLC: nivolumab at a dose of flat dose of 360 mg as 30 minute IV infusion on Day 1, followed by gemcitabine at a dose of 1000 mg/m^2 or 1250 mg/m^2 (per local prescribing information) for a 30 minute IV infusion or per institutional standard and cisplatin at a dose of 75 mg/m^2 as a 120-minute IV infusion or per institutional standard, of a 3-week treatment cycle

Approved v1.0 [redacted]

for up to 3 cycles. Gemcitabine will also be administered at a dose of 1000 mg/m^2 or 1250 mg/m^2 as a 30 minute IV infusion or per institutional standard on day 8 of each 3-week treatment cycle.

- Any histology: nivolumab at a flat dose of 360 mg as 30-minute IV infusion on Day 1, followed by paclitaxel 175 or 200 mg/m^2 IV over 180 minutes or per institutional standard and carboplatin AUC 5 or 6 IV over 30 minutes or per institutional standard of a 3-week treatment cycle, for up to 3 cycles.

Following the completion of neoadjuvant treatment, all participants who remain operative candidates will undergo definitive surgery for their NSCLC within 6 weeks after completing neoadjuvant treatment.

Following definitive surgery, participants in each arm may receive up to 4 cycles of adjuvant chemotherapy with or without radiation per institutional standard at the discretion of the investigator. Investigators may choose from the following post-operative regimens:

- Regimen 1:
  - Vinorelbine 25 mg/m^2 or 30 mg/m^2 IV (per local prescribing information) push over 10 minutes or per institutional standard on Days 1 and 8
  - Cisplatin 75 mg/m^2 IV over 120 minutes or per institutional standard on Day 1, immediately following vinorelbine
- Regimen 2:
  - Docetaxel 60 mg/m^2 or 75 mg/m^2 IV (per local prescribing information) over 60 minutes or per institutional standard on Day 1
  - Cisplatin 75 mg/m^2 IV over 120 minutes or per institutional standard on Day 1, immediately following docetaxel
- Regimen 3 (squamous histology):
  - Gemcitabine 1000 mg/m^2 or 1250 mg/m^2 IV (per local prescribing information) over 30 minutes or per institutional standard on Days 1 and 8
  - Cisplatin 75 mg/m^2 IV over 120 minutes or per institutional standard on Day 1, immediately following gemcitabine
- Regimen 4 (non-squamous histology only):
  - Pemetrexed 500 mg/m^2 IV over 10 minutes or per institutional standard on Day 1
  - Cisplatin 75 mg/m^2 IV over 120 minutes or per institutional standard on Day 1, immediately following pemetrexed
- Regimen 5:
  - Paclitaxel 175 or 200 mg/m^2 IV over 180 minutes or per institutional standard on Day 1
  - Carboplatin AUC5 or 6 IV over 30 minutes or per institutional standard on Day 1, immediately following paclitaxel

The study design schematic is presented in Figure 2.1-1.

Figure 2.1-1: Study Design Schematic

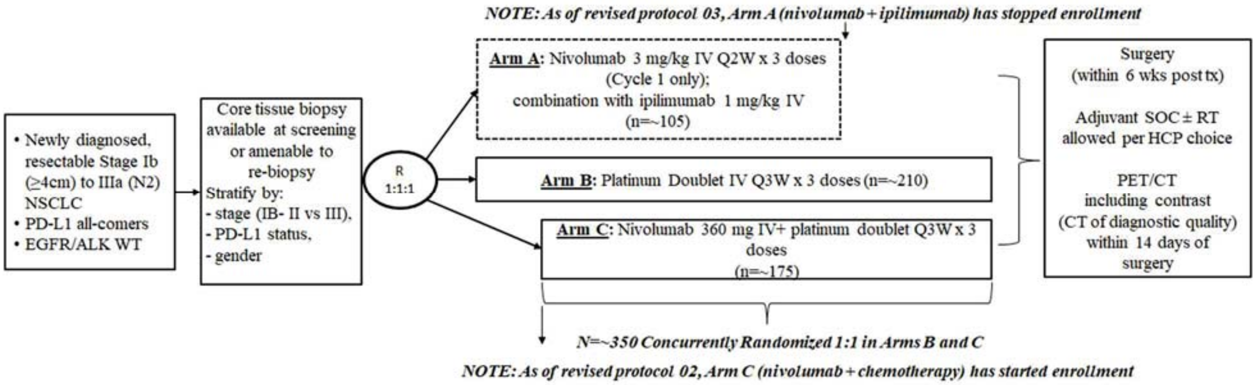

A Data Monitoring Committee (DMC) will be established to provide oversight of safety and efficacy and overall risk/benefit monitoring of the study.

Note that in this document the words “participant” and “subject” are used intercheangeably.

2.2 Treatment Assignment

CA209816 is an open-label, randomized trial. Participants with Stage IB (≥ 4 cm), II and IIIA (N2) considered resectable will be eligible to participate. After the participant’s initial eligibility is established and informed consent has been obtained, the participant must be enrolled into the study by calling the IRT to obtain a participant number. Every participant that signs the informed consent form must be assigned a participant number in IRT.

Once enrolled in IRT, enrolled participants who have met all eligibility criteria will be ready to be randomized through IRT to treatment Arm A, Arm B or Arm C.

In the original study design (before revised protocol 02) subjects were randomized to in a 1:1 ratio to arms A or B.

Following revised protocol 02, subjects were randomized to in a 1:1:1 ratio to arms A, B or C.

As of revised protocol 03, subjects were randomized to in a 1:1 ratio to arms B or C.

- Arm A: nivolumab 3 mg/kg plus ipilimumab 1 mg/kg.
- Arm B: platinum doublet chemotherapy.
- Arm C: nivolumab 360mg flat dose plus platinum doublet chemotherapy

The randomization uses permuted blocks stratified by the following factors:

- PD-L1 expression (≥1% or <1%/not evaluable/indeterminate)
- Disease stage (IB/II vs IIIA)
- Gender

2.3 Blinding and Unblinding

This is an open-label study; blinding procedures between participants and investigators are not applicable. The specific treatment to be taken by a participant will be assigned using an IRT. No aggregate summary data by treatment group are disclosed to the study team at any time of the study conduct until achievement of primary endpoint significance (pCR or EFS at interim analysis) or the final EFS analysis. Treatment assignments will be released to the bioanalytical laboratory in order to minimize unnecessary analysis of samples.

The blinded independent pathology review (BIPR) and blinded independent central review (BICR) will be blinded to treatment arms.

2.4 Protocol Amendments

Table 2.4-1: Protocol Amendments

Document/Date of Issue	Summary of Change
Revised Protocol 05<br>18-Sep-2019	• Modified pCR analysis population and projected timelines<br>• Updated surgical approach endpoint<br>• Updated the censoring rule of TTDM<br>• No optional biopsy at disease progression collected in China.<br>• Updated Management Algorithms to include myocarditis
Revised Protocol 04<br>25-Jun-2019	[redacted]<br>• Added the concomitant administration of substances that are also tubularly secreted (eg, probenecid) could potentially result in delayed clearance of pemetrexed.<br>• Added hypothesis testing for overall survival<br>• Clarified the pCR analysis population<br>• Added exploratory endpoint of Event Free Survival on next line of therapy<br>• Added instructions for BICR<br>• Updated Appendix 8 Adverse Events and Serious Adverse Events: Definitions and Procedures for Recording, Evaluating, Follow Up and Reporting Adverse Events
Revised Protocol 03<br>21-Sep-2018	• Enrollment in Arm A (nivolumab plus ipilimumab) was stopped. Based on external data from a neo-adjuvant phase II trial (NADIM5) which suggested anti-PD-1 + chemotherapy to be more promising, clinical development of nivolumab + chemotherapy was prioritized.<br>• Randomization of participants (1:1 ratio) into Arm C and B will continue to for a total of 350 participants

Table 2.4-1: Protocol Amendments

Document/Date of Issue	Summary of Change
	• Definition of event free survival is clarified.<br>• Participants with large-cell neuroendocrine carcinoma tumor histology are excluded<br>• Additional platinum doublet chemotherapy regimen (paclitaxel/carboplatin) was added<br>• Dose modification for docetaxel was updated<br>• Time to death or distant metastases (TTDM) was added to secondary endpoints.<br>• Tumor assessments for participants who do not proceed to definitive surgery was clarified<br>• Endpoints and statistical analyses adapted consequently to Arm A discontinuation.<br>• Rationale, background information, and trial schematic were updated<br>• Pulmonary function parameters were clarified<br>• Time relationship between adjuvant radiotherapy and tumor imaging assessments was clarified<br>• Time window of Cycle 1 Day 1 end-of-infusion PK sampling for Arm A and C was clarified
Revised Protocol 02<br>06-Jul-2017	• Nivolumab plus platinum-doublet chemotherapy arm (Arm C) was added.<br>• The sample size was increased to 642 participants consequently to the addition of Arm C and change of primary endpoint.<br>• The primary objective was changed to multiple primary objectives of event free survival and pathological complete response; major pathological response was changed to the secondary objective.<br>• Additional rationale and background information was provided.<br>• Pre-screening tissue requirement was increased from minimum of 10 slides to 15 slides.<br>• Contrast requirements for brain MRI scans were updated.<br>• Time window for pulmonary function test window was expanded from within 28 days of randomization to within 6 weeks of randomization.<br>• Synopsis was updated with exploratory objectives, endpoints and schema.<br>• Language in treatment administered was deleted and reference to Investigator Brochure and Pharmacy Manual was included.<br>[redacted]
Revised Protocol 01	• Incorporates Amendment 02 and Administrative Letters 01 and 02

Approved v1.0 [redacted]

Table 2.4-1: Protocol Amendments

Document/Date of Issue	Summary of Change
03-Mar-2017	
Amendment 02<br>03-Mar-2017	• To adjust the dosing details of the chemotherapy regimens to include the dose approved by the local prescribing information and the standard of care infusion time for each country included in this study.<br>• To expand and to split the broad biomarker objective into 3 more detailed objectives.<br>• Clarify lymph node samples at screening and at definitive surgery.<br>• Clarify requirements for PET/CT scans and broadening the window of scans prior to surgery.<br>• Clarify tissue sample process for calculation of the primary endpoint.<br>• Adjust Hepatitis B Virus criteria.<br>• Added live vaccines and strong CYP3A4 inhibitors to the Prohibited Treatments.<br>• added caution for concomitant administration of NSAIDs with pemetrexed<br>• added unacceptable methods of contraception to Appendix 6.
Administrative Letter 02<br>30-Nov-2017	• Clarify the correct version of the TNM Staging System.<br>• Clarify that a minimum of 228 PD-L1+ participants will be randomized.<br>• Clarify that physical exams, vital signs, and physical measurements should be collected prior to each dose of neoadjuvant and adjuvant therapy.<br>• Clarify that the first post-operative tumor assessment should be performed 12 weeks (± 7 days) after definitive surgery.<br>• Remove the phrase “non-protocol regimen” in regards to a noncisplatin<br>• Regimen as the protocol includes a non-cisplatin regimen option.<br>• Clarify that weight-based dosing should be rounded up to the nearest milligram or per institutional standards.<br>• Clarify that the EQ-5D-3L should be collected prior to Day 1 only in cycles that have multiple dosing days in each cycle.
Administrative Letter 01<br>31-Oct-2017	• To correct the IND number
Original Protocol	• Not Applicable

Approved v1.0 [redacted]

Table 2.4-1: Protocol Amendments

Document/Date of Issue	Summary of Change
30-Sep-2016	

2.5 Data Monitoring and Other External Committees

A Data Monitoring Committee (DMC) is established to provide oversight of safety and efficacy considerations in protocol CA209816. Additionally, the DMC will provide advice to the sponsor regarding actions the committee deems necessary for the continuing protection of participants enrolled in the study. The DMC will be charged with assessing such actions in light of an acceptable benefit/risk profile for nivolumab in combination with ipilimumab or chemotherapy. The DMC will act in an advisory capacity to BMS and will monitor participant safety and evaluate the available efficacy data for the study. The oncology therapeutic area of BMS has primary responsibility for design and conduct of the study.

Independent pathology (BIPR) and radiology review (BICR) will be established for central review and confirmation of efficacy endpoints.

3 OBJECTIVES

3.1 Primary

- To compare the event-free survival (EFS) by BICR in participants receiving nivolumab plus platinum doublet chemotherapy vs participants receiving platinum doublet chemotherapy in operable stage IB (≥ 4 cm), II, or resectable IIIA (N2) NSCLC
- To compare the pathologic complete response (pCR) rate in participants receiving nivolumab plus platinum doublet chemotherapy vs participants receiving platinum doublet chemotherapy in operable stage IB (≥ 4 cm), II, or resectable IIIA (N2) NSCLC

3.2 Secondary

- To assess the major pathologic response (MPR) rate by BIPR of participants receiving nivolumab plus platinum doublet chemotherapy vs participants receiving platinum doublet chemotherapy in operable stage IB (≥ 4 cm), II, or resectable IIIA (N2) NSCLC
- To assess the OS of participants receiving nivolumab plus platinum doublet chemotherapy vs participants receiving platinum doublet chemotherapy in operable stage IB (≥ 4 cm), II, or resectable IIIA (N2) NSCLC
- To assess the time to death or distant metastases (TTDM) of participants receiving nivolumab plus platinum doublet chemotherapy vs participants receiving platinum doublet chemotherapy in operable stage IB (≥ 4 cm), II, or resectable IIIA (N2) NSCLC

3.3 Exploratory Objectives

- To assess clinical response rate (cRR) by BICR of participants receiving nivolumab plus platinum doublet chemotherapy vs participants receiving platinum doublet chemotherapy in operable stage IB (≥ 4 cm), II, or resectable IIIA (N2) NSCLC

Approved v1.0 [redacted]

- To assess the pCR rate, MPR rate , cRR, EFS, TTDM and OS in early-stage NSCLC participants treated with nivolumab plus platinum doublet chemotherapy compared to those treated with platinum doublet chemotherapy by PDL1 status (PD-L1 ≥ 1% , PD L1< 1% /not evaluable/ indeterminate)
- To assess the feasibility of surgery and rate of peri- and post-operative complications (within 90 days of surgery) in participants receiving nivolumab plus platinum doublet chemotherapy compared to participants receiving platinum doublet
- To assess the safety and tolerability of nivolumab plus platinum doublet chemotherapy compared to platinum doublet chemotherapy in early stage NSCLC
- To describe the pCR rate, MPR rate , cRR, EFS, OS, TTDM, feasibility of surgery, rate of peri- and post-operative complications (within 90 days of surgery), safety and tolerability in early-stage NSCLC participants treated with nivolumab plus ipilimumab and by PDL1 status (PD-L1 ≥ 1% , PD L1< 1%/not evaluable/indeterminate)
- To assess pharmacokinetics of the nivolumab plus ipilimumab or nivolumab plus platinum doublet chemotherapy in participants with early stage NSCLC
- To assess the participant's overall health status and health utility using the 3-level version of the EQ-5D-3L visual analog scale (VAS) and utility index, respectively
- To evaluate tumor mutational burden as a potential predictive biomarker of efficacy (such as EFS and OS) of nivolumab plus platinum doublet chemotherapy and of platinum-doublet chemotherapy, using data generated from tumor and blood (germ-line control) specimens.

[redacted]

- To explore potential predictive biomarkers of nivolumab plus platinum doublet chemotherapy efficacy (such as EFS and OS) in peripheral blood and tumor specimens [redacted]

[redacted]

4 ENDPOINTS

4.1 Primary Endpoint(s)

The primary objectives in the study will be evaluated by the multiple primary endpoints of EFS and pCR.

4.1.1 Event-Free Survival

Two definitions are used for analysis of EFS. The primary definition accounts for subsequent therapy by censoring at the last evaluable tumor assessment on or prior to the date of subsequent therapy (outside of the protocol specified adjuvant therapy). The secondary definition does not incorporate censoring due to subsequent therapy.

EFS rate at time *T* is defined as the probability that a subject has not progressed/recurred and is alive at time *T* following randomization. EFS rates at fixed time points (e.g. 12 months, depending on the minimum follow-up) are defined as the probability that a subject has not progressed and is alive at time *T* following randomization.

4.1.1.1 Primary Definition of Event-Free Survival

Event free survival is defined as the length of time from randomization to any of the following events: progression of disease precluding surgery, progression or recurrence disease after surgery, or death due to any cause.

- Progression/recurrence will be based on BICR assessment per RECIST 1.1.
- A pre-surgical progression (even if reaching the RECIST 1.1 criteria) which does not preclude surgery is not considered as an event.
- A progression not reaching the RECIST 1.1 criteria (e.g. clinical progression) but which still precludes surgery (i.e. reason for no surgery is disease progression) is considered as an event (event at the investigator reported earliest clinical or radiographic progression date, or at the date of randomization if no progression date reported).
- For participants with surgery, any new lesions on the first post-surgical baseline imaging compared with the pre-surgical scans will be identified as new lesion and will be counted as an event. For those without new lesion on the first post-surgical scan, the first tumor assessment post surgery will be used as re-baseline and recurrence/progression per RECIST 1.1 will be evaluated based on that re-baseline.
- Participants who do not undergo surgery for reason other than progression will be considered to have an event at RECIST 1.1 progression or death.
- Participants who died without a reported progression/disease recurrence will be considered to have experienced an event on the date of their death.

The following censoring rules will be applied for the primary definition of EFS:

- Participants who did not report progression/recurrence of disease or die will be censored on the date of their last evaluable tumor assessment.
- Participants who did not have any on study tumor assessments and did not die will be censored on the date they were randomized.
- Subjects who receive subsequent anti-cancer therapy, outside of the protocol-specified adjuvant therapy, prior to documented progression/recurrence/death will be censored at the date of the last evaluable tumor assessment conducted on or prior to the date of initiation of the subsequent anti-cancer therapy.
- Subjects who did not have a documented progression/recurrence/death and received subsequent anti-cancer therapy outside of the protocol-specified adjuvant therapy will be

censored at the date of the last evaluable tumor assessment conducted on or prior to the initiation of the subsequent anti-cancer therapy.

- Participants without baseline scan and without surgery will be censored on the date of randomization (regardless of death).
- Censoring rules for the primary definition of EFS (EFS truncated at subsequent therapy) are presented as follows and depicted in Figure 4.1.1.1-1
- It is to be noted that in case of new primary cancer, if such lesions are present on tumor assessment at the BICR, they will be considered as new lesions, since the BICR does not have access to biopsy results.

Figure 4.1.1.1-1: EFS Primary Definition

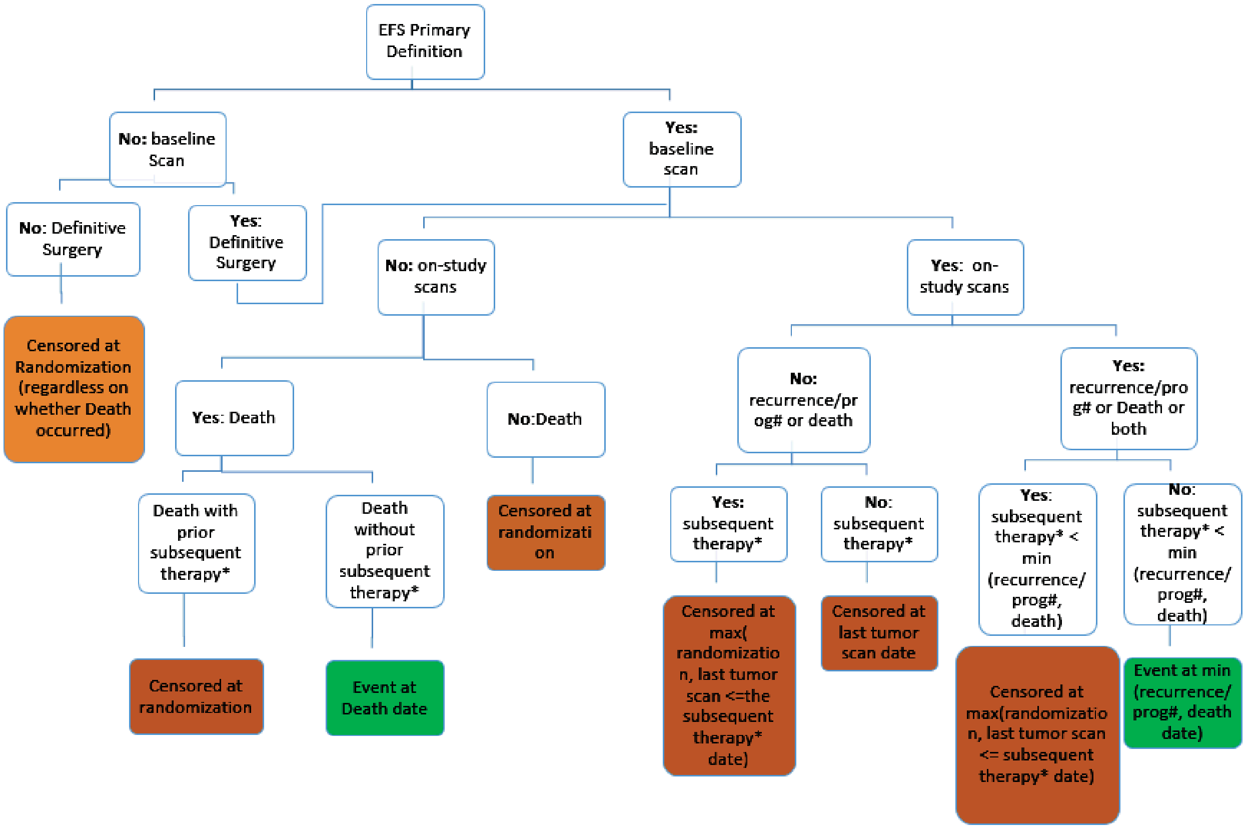

*Subsequent Therapy excluding per protocol adjuvant therapy

Progression precluding surgery, RECIST 1.1 recurrence or progression post surgery (for participants with surgery), RECIST 1.1 progression (for participants without surgery)

4.1.1.2 Secondary Definition of Event-Free Survival

The secondary definition of EFS (ITT definition) is defined as the length of time from randomization to any of the following events: progression of disease precluding surgery, progression or recurrence disease after surgery, or death due to any cause.

- Progression/recurrence will be based on BICR assessment per RECIST 1.1.

- A progression (even if reaching the RECIST 1.1 criteria) which does not preclude surgery is not considered as an event.
- A progression not reaching the RECIST 1.1 criteria but which still precludes surgery (i.e. reason for no surgery is disease progression) is considered as an event (event at the investigator reported earliest clinical or radiographic progression date, or at the date of randomization if no progression date reported).
- For participants with surgery, the first tumor assessment post surgery will be used as re-baseline and recurrence/progression per RECIST 1.1 will be evaluated based on that re-baseline. Any new lesions on the post-surgical baseline imaging compared with the pre-surgical scans will be identified as new lesion and will be counted as an event.
- Participants who do not undergo surgery for reason other than progression will be considered to have an event at RECIST 1.1 progression or death.
- Participants who died without a reported progression/disease recurrence will be considered to have experienced an event on the date of their death.

The following censoring rules will be applied for the secondary definition of EFS:

- Participants who did not report progression/recurrence of disease or die will be censored on the date of their last evaluable tumor assessment.
- Participants who did not have any on study tumor assessments and did not die will be censored on the date they were randomized.
- Participants without baseline scan and without surgery will be censored on the date of randomization (regardless of death).
- Censoring rules for the secondary definition of EFS (ITT definition) are presented as follows and depicted in Figure 4.1.1.2-1

Approved v1.0 [redacted]

Figure 4.1.1.2-1: EFS Secondary Definition

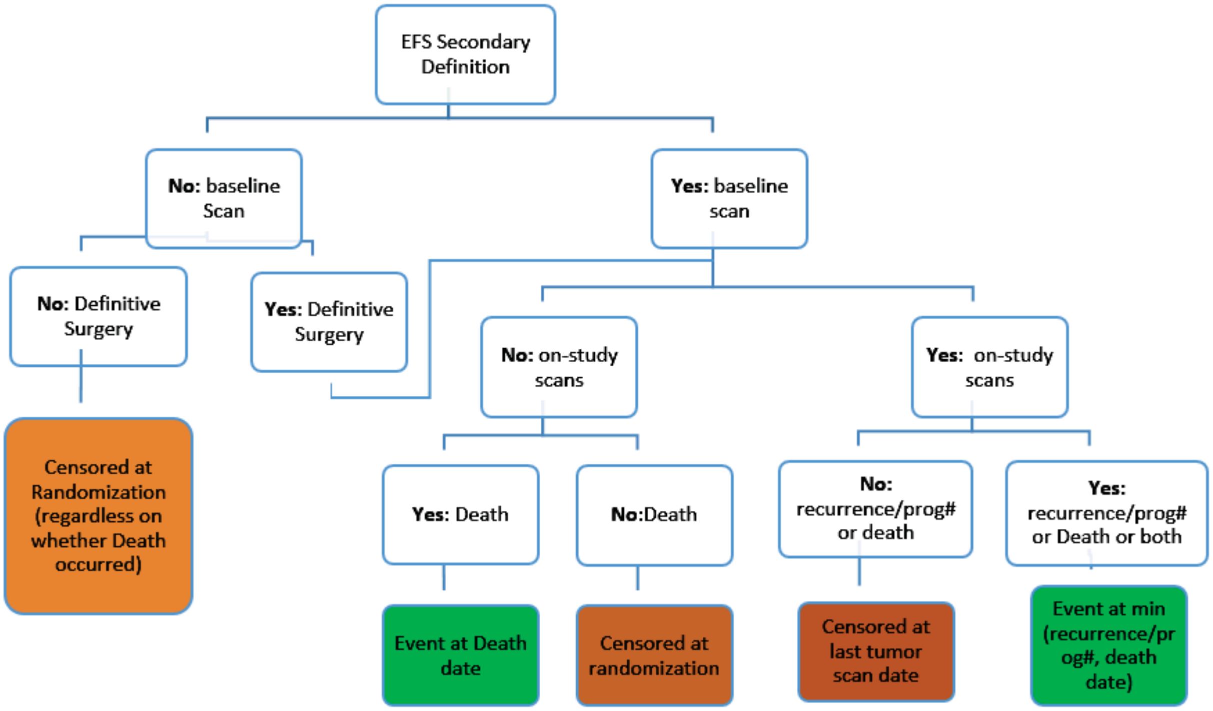

Progression precluding surgery, RECIST 1.1 recurrence or progression post surgery (for participants with surgery), RECIST 1.1 progression (for participants without surgery)

4.1.2 Pathologic Complete Response Rate

Pathological complete response (pCR) rate is defined as number of randomized participants with absence of residual tumor in lung and lymph nodes at surgery as evaluated by blinded independent pathological review (BIPR), divided by the number of randomized participants for each treatment group. Randomized subjects who are no longer eligible for surgery, or who are on alternative anti-cancer therapy before surgery, or who discontinue the study (e.g. withdraw consent) before surgery are all counted as non-responders.

4.2 Secondary Endpoint(s)

4.2.1 Overall Survival

Overall survival (OS) is defined as the time between the date of randomization and the date of death due to any cause. For a subject without documentation of death, OS will be censored on the last date the subject was known to be alive.

4.2.2 Major Pathological Response Rate

Major pathological response (MPR) rate, defined as number of randomized participants with ≤ 10% residual tumor in lung and lymph nodes at surgery as evaluated by BIPR, divided by the number of randomized participants for each treatment group. Viable tumors in situ carcinoma should not be included in MPR calculation. Randomized subjects who are no longer eligible for

surgery, or who are on alternative anti-cancer therapy, or who discontinue the study (e.g. withdraw consent) before surgery are all counted as non-responders.

4.2.3 Time to Death or Distant Metastases

Time to Death or Distant Metastates (TTDM) is defined as the time between the date of randomization and the first date of distant metastasis or the date of death in the absence of distant metastasis. Distant metastasis is defined as any new lesion that is outside of the thorax using BICR according to RECIST 1.1. It will be derived based on the location of lesions outside the thorax. Participants who died without reported distant metastasis will be considered to have experienced an event on the date of their death.

The following censoring rules will be applied TTDM:

- Participants who have not developed distant metastasis nor died will be censored on the date of their last evaluable tumor assessment.
- Participants who did not have any on study tumor assessments and did not die will be censored on the date they were randomized.

4.3 Exploratory Endpoint(s)

4.3.1 Clinical Response Rate by BICR

Clinical response rate (cRR) is defined as proportion of randomized participants whose overall radiological response prior to definitive surgery (or best overall radiological response (BOR) at the first protocol planned tumor assessment if a subject has no surgery) is either a complete response (CR) or partial response (PR) per RECIST 1.1 criteria by BICR. Participants who received alternative anti-cancer therapy before the pre-surgery tumor assessment will be counted as non-responders.

4.3.2 Event Free Survival on Next Line of Therapy (EFS2)

EFS on next line therapy (EFS2) is defined as the time from randomization to objectively documented progression, per investigator assessment, after the next line of therapy or to death from any cause, whichever occurs first. Subjects who were alive and without progression after the next line of therapy will be censored at last known alive date.

The following censoring rules will be applied for EFS2:

- Subjects who did not receive subsequent next line systemic anti-cancer therapy:
  - Subjects who died, the death date is the event date;
  - Else the subject’s EFS2 is censored at the last known alive date.
- Subjects who received subsequent next line anti-cancer therapy:
  - Subjects who had a disease recurrence/progression after the start of subsequent anti-cancer therapy, this disease progression date is the event date;
  - Else if a subject died or start of second next line therapy, the date of min (death, start date of second next line therapy) is the event date;
  - Else the subject’s EFS2 is censored at the last known alive date.

Subsequent next line of therapy will include subsequent systemic regimen given in one of the following settings Unresectable, Locally Advanced or lines of therapy in metastatic setting.

4.3.3 Surgery Related Endpoints

The endpoints related to surgery include proportion of subjects with delayed or canceled surgery, duration of surgery, length of hospital stay, surgical approach, including completeness of surgery (R0/R1/R2 resection), incidence of AE/SAE associated with surgery up to 90 days after surgery.

4.3.4 Safety and Tolerability

The assessment of safety will be based on the incidence of adverse events (AEs), serious adverse events (SAEs), adverse events leading to discontinuation, adverse events leading to dose modification, select adverse events (select AEs) for EU/ROW Submissions, immune-mediated AEs (IMAEs) for US Submission, other events of special interest (OEOSI), and deaths. The use of immune modulating concomitant medication will be also summarized. In addition clinical laboratory tests, and immunogenicity (i.e. development of anti-drug antibody) will be analyzed.

4.3.5 Pharmacokinetics

Pharmacokinetics will be measured by the serum concentration of nivolumab and ipilimumab. Samples will be collected to characterize pharmacokinetics of nivolumab and ipilimumab and to explore exposure-safety and exposure-efficacy relationships. The population pharmacokinetics analysis will be presented separately from the main clinical study report.

4.3.6 Biomarkers

Biomarkers potentially associated with clinical endpoints will be measured by analyzing tumor and blood samples.

Biomarker endpoints include, but not limited to, tumor mutational burden (TMB) using data generated from tumor specimens. [redacted]

Results for biomarkers analyses (other than PD-L1 and TMB) will be summarized outside of CSR.

4.3.6.1 Tumor Mutational Burden

TMB is measured in CA209816 using the [redacted] assay. [redacted] is a next-generation sequencing (NGS) assay targeting the full coding regions of 523 genes implicated in the pathogenesis of solid tumors. Using enrichment-based library preparation techniques for use with formalin-fixed, paraffin-embedded (FFPE) samples, [redacted] can analyze DNA and RNA from the same sample, detecting single nucleotide variants (SNVs), insertions and deletions (indels), amplifications, splice variants, and fusions, in a single sequencing run. TMB, is derived by summing the total of all synonymous and non-synonymous detected small DNA variants (SNVs and indels) across the entire coding region (~1.3Mb are in coding regions) with sophisticated variant calling and germline filtering algorithms for enhanced accuracy. The

resulting number is communicated as mutations per Mb unit (mut/Mb). The cutoff used for analysis will be ≥ 12.3 mut/Mb, <12.3 mut/Mb[6].

4.3.6.2 PD-L1 Protein Expression

PD-L1 expression is defined as the percent of tumor cells membrane staining in a minimum of 100 evaluable tumor cells per validated Dako PD-L1 immunohistochemistry (IHC) assay. This is referred to as quantifiable PD-L1 expression. If the PD-L1 staining could not be quantified, it is further classified as:

1) Indeterminate: Tumor cell membrane staining hampered for reasons attributed to the biology of the tumor tissue sample and not because of improper sample preparation or handling.
2) Not evaluable: Tumor tissue sample was not optimally collected or prepared and PD-L1 expression is neither quantifiable nor indeterminate. Not evaluable can be determined from H&E process before the tumor biopsy specimen is sent for PD-L1 evaluation or from the H&E process during PD-L1 evaluation.

Subjects with missing PD-L1 expression are subjects with no tumor tissue sample available for evaluation.

PD-L1 expression will be collected in the IRT as well as in the clinical database. Statistical analysis using PD-L1 expression will be solely based on PD-L1 expression data from clinical database. Stratified analyses will use stratification from IRT, unless otherwise specified.

4.3.7 Outcomes Research

4.3.7.1 EQ-5D-3L

Subjects' reports of general health status will be assessed using the EuroQoL Group's EQ-5D-3L. EQ-5D-3L essentially has 2 components: the descriptive system and the visual analogue scale (VAS).

The instrument's descriptive system consists of 5 dimensions: mobility, self-care, usual activities, pain/discomfort, and anxiety/depression. Each dimension has 3 levels, reflecting "no health problems," "moderate health problems," and "extreme health problems." A dimension for which there are no problems is said to be at level 1, while a dimension for which there are extreme problems is said to be at level 3. Thus, the vectors 11111 and 33333 represent the best health state and the worst health state, respectively, described by the EQ-5D-3L. Altogether, the instrument describes $3^5 = 243$ health states. Empirically derived weights can be applied to an individual's responses to the EQ-5D-3L descriptive system to generate an index measuring the value to society of his or her current health. Such preference-weighting systems have been developed for the UK, US, Spain, Germany, and numerous other populations. For this study, EQ-5D-3L utility index values will be computed using a scoring algorithm based on the United Kingdom Time-Trade-Off (UK TTO) value set[7]

In addition, the EQ-5D-3L includes a VAS, which allows respondents to rate their own current health on a 101-point scale ranging from 0="worst imaginable" health to 100="best imaginable" health state [8].

All questionnaires completed at baseline and on-study will be assigned to a time-point according to the windowing criteria in Table 4.3.7.1-1 and included in the analysis. In case a subject has two on-study assessments within the same window, the assessment closest to the time-point will be used and, in the case of two assessments at a similar distance to the time-point, the latest one will be chosen. In the event where the subject has no assessment at all in a specific window, the observation will be treated as missing for that time-point.

Table 4.3.7.1-1: Time Windows for EQ-5D-3L Assessments

Time Point	Nominal Day	Time Window
Baseline	D1	Prior to first dose on Day 1
Week 3 (Arm A)	D15	Day 2 thru day 22 inclusive
Week 4 (Arms B and C)	D22	Day 2 thru day 32 inclusive
Week 5 (Arm A)	D29	Day 23 thru day 36 inclusive
Week 7 (Arms B and C)	D43	Day 33 thru day 53 inclusive
Post-neoadjuvant visit 1	Last neodajuvant dose + 30 days	Assessment post last neoadjuvant dose and within 65 days of last neo dose
Post-neoadjuvant visit 2	Last neodajuvant dose + 100 days	Assessment post 65 days of last neoadjuvant dose and within 145 days of last neo dose
**Subjects without adjuvant:**		
Survival Follow-up 1	Last neodajuvant dose + 190 days	Assessment post 145 days of last neoadjuvant dose and within 235 days of last neo dose
Survival Follow-up 2	Last neodajuvant dose + 280 days	Assessment post 235 days of last neoadjuvant dose and within 325 days of last neo dose
Survival Follow-up 3	Last neodajuvant dose + 370 days	Assessment post 325 days of last neoadjuvant dose and within 415 days of last neo dose
Survival Follow-up 4	Last neodajuvant dose + 460 days	Assessment post 415 days of last neoadjuvant dose and within 550 days of last neo dose
Survival Follow-up 5	Last neodajuvant dose + 640 days	Assessment post 550 days of last neoadjuvant dose and within 730 days of last neo dose
Survival Follow-up 6	Last neodajuvant dose + 820 days	Assessment post 730 days of last neoadjuvant dose and within 910 days of last neo dose
Then Survival Follow-up i	Last neodajuvant dose + 460 + (i-4)*180 days	Assessment post (nominal day - 90) days of last neoadjuvant dose and within (nominal day + 90) days of last neo dose
**Subjects with adjuvant:**		
Adjuvant Cycle 1	-	Assessment reported in the F01 (adjuvant cycle 1) visit
Adjuvant Cycle 2	-	Assessment reported in the F02 (adjuvant cycle 2) visit
Adjuvant Cycle 3	-	Assessment reported in the F03 (adjuvant cycle 3) visit

Table 4.3.7.1-1: Time Windows for EQ-5D-3L Assessments

Time Point	Nominal Day	Time Window
Adjuvant Cycle 4	-	Assessment reported in the F04 (adjuvant cycle 4) visit
Survival Follow-up 1	Max (Last adjuvant dose or Post-neoadjuvant visit) + 90 days	Assessment post 45 days of Max (Last adjuvant dose or Post-neoadjuvant visit) and within 135 days of Max (Last adjuvant dose or Post-neoadjuvant visit)
Survival Follow-up 2	Max (Last adjuvant dose or Post-neoadjuvant visit) + 180 days	Assessment post 135 days of Max (Last adjuvant dose or Post-neoadjuvant visit) and within 225 days of Max (Last adjuvant dose or Post-neoadjuvant visit)
Survival Follow-up 3	Max (Last adjuvant dose or Post-neoadjuvant visit) + 270 days	Assessment post 225 days of Max (Last adjuvant dose or Post-neoadjuvant visit) and within 315 days of Max (Last adjuvant dose or Post-neoadjuvant visit)
Survival Follow-up 4	Max (Last adjuvant dose or Post-neoadjuvant visit) + 360 days	Assessment post 315 days of Max (Last adjuvant dose or Post-neoadjuvant visit) and within 450 days of Max (Last adjuvant dose or Post-neoadjuvant visit)
Survival Follow-up 5	Max (Last adjuvant dose or Post-neoadjuvant visit) + 540 days	Assessment post 450 days of Max (Last adjuvant dose or Post-neoadjuvant visit) and within 630 days of Max (Last adjuvant dose or Post-neoadjuvant visit)
Survival Follow-up 6	Max (Last adjuvant dose or Post-neoadjuvant visit) + 720 days	Assessment post 630 days of Max (Last adjuvant dose or Post-neoadjuvant visit) and within 810 days of Max (Last adjuvant dose or Post-neoadjuvant visit)
Then Survival Follow-up i	Max (Last adjuvant dose or Post-neoadjuvant visit) + 360 + (i-4)*180 days	Assessment post (nominal day - 90) days of last neo dose and within (nominal day + 90) days of last neo dose

5 SAMPLE SIZE AND POWER

The original study design (before Revised protocol 02) had two arms, with participants randomized in a 1:1 ratio to either neoadjuvant nivolumab plus ipilimumab or platinum doublet chemotherapy arm. Revised protocol 02 added a new, neoadjuvant nivolumab plus platinum doublet chemotherapy arm. When the third arm opens and as each site receives IRB/EC approval of revised protocol 02, the IRT will switch to a 1:1:1 randomization at the respective site. Starting from that point on, the site will only enroll under revised protocol 02.

Revised protocol 03 withholds randomization into the arm of neoadjuvant nivolumab plus ipilimumab but continues randomizing eligible participants into either neoadjuvant nivolumab plus

platinum doublet chemotherapy arm or platinum doublet chemotherapy arm in a 1:1 ratio. Approximately 350 participants (175 participants per arm) will be randomized between 2 arms neoadjuvant nivolumab plus platinum doublet chemotherapy or platinum doublet chemotherapy from 1:1:1 randomization in revised protocol 02 and 1:1 randomization in revised protocol 03. Participants already randomized in the original 2-arm part (neoadjuvant nivolumab plus ipilimumab vs neoadjuvant chemotherapy) and in the arm of neoadjuvant nivolumab plus ipilimumab in 3-arm part defined by revised protocol 02 will remain in trial and continue scheduled trial procedures. It is expected to have around 70 participants randomized in the original 2-arm part and approximately other 75 participants randomized in the arm of neoadjuvant nivolumab plus ipilimumab in the 3-arm part. It is estimated that there will be a total of approximately 500 participants on the study.

Starting from 1:1:1 randomization, approximately 350 participants will be randomized to the 2 arms neoadjuvant nivolumab plus platinum doublet chemotherapy or platinum doublet chemotherapy in a 1:1 ratio (concurrently randomized).

The sample size of the study is calculated based on the primary endpoint of EFS and accounts for the multiple primary endpoints comparisons: pCR (per BIPR) and EFS (per BICR) with an initial alpha allocation of 0.01 and 0.04 respectively. Formal analyses of pCR and EFS may be conducted at different timepoints. The fallback method will be used, ie, if the pCR comparison between Arm C and Arm B is statistically significant, then 0.01 alpha allocated to pCR will be passed to the EFS comparison for Arm C vs Arm B and the EFS comparison will be conducted at the alpha = 0.05 level. If the pCR comparison between Arm C and Arm B is not statistically significant, then the EFS comparison for Arm C vs Arm B will be conducted at the alpha = 0.04 level. [redacted]

5.1 Pathologic Complete Response (pCR)

The primary analysis of pCR will be performed after the 350 randomized participants in neoadjuvant nivolumab plus platinum doublet chemotherapy and platinum doublet chemotherapy (from start of 1:1:1 randomization) have an opportunity for surgery.

Assuming an accrual rate of 10 participants (all comers) a month between Arms B and C during 1:1:1 randomization (about 10 months), and 15 participants per month during 1:1 randomization, it is anticipated that the 350 participants will be randomized in approximately 27 months. The pCR endpoint is expected to be analyzed after about 30 months from start of 1:1:1 randomization.

Assuming pCR rate of 10% on Arm B chemotherapy and 30% on Arm C nivolumab plus chemotherapy, respectively, the 350 participants will provide more than 90% power to detect an odds ratio of 3.857 with a 2-sided type I error of 1%.

It is estimated that there will be about 110 subjects randomized to Arm A neoadjuvant nivolumab plus ipilimumab before revised protocol 03 is implemented. Assuming true pCR rate is 15% on this arm, there is 95% probability that the lower bound of 95% exact confidence interval of pCR is above 5%.

5.2 Event Free Survival (EFS)

For the formal comparison of EFS as assessed by BICR for nivolumab plus platinum doublet chemotherapy (Arm C) vs platinum doublet chemotherapy (Arm B), only participants randomized from 1:1:1 randomization in revised protocol 02 and 1:1 randomization in revised protocol 03 will be counted (participants concurrently randomized in arms B and C). If the comparison is based on alpha=0.04, a total of 185 events ensure that an overall 2-sided 4% significance level sequential test procedure with 2 interim analyses after 111 and 148 events (60% and 80% of events required for final analysis) in 350 randomized participants will have 80% power assuming an exponential distribution with the median EFS time in the control (Arm B) is 40 months and of 61.5 months in the nivolumab and platinum doublet chemotherapy (Arm C) (corresponding to an hazard ratio of 0.65). It is anticipated the EFS analyses will take place at about 42, 54, and 69 months from start of 1:1:1 randomization. The stopping boundaries at the interim and final EFS analyses will be derived based on the exact number of events using Lan-DeMets alpha spending function with O'Brien-Fleming boundaries. If the interim analyses of EFS is performed at exactly 111 and 148 events, respectively, the nominal significance level for EFS superiority are 0.005 and 0.017, respectively. The nominal significance level for the final look of EFS after 185 events would then be 0.034. If pCR comparison is significant, then the EFS comparison will be based on alpha=0.05, the same number of events will have 82.5% power. The nominal significance level for the 2 interim and final EFS analyses will be 0.008, 0.022, 0.042.

Table 5.3-1 summarizes the key parameters of the sample size justification in the concurrently randomized participants from Arms B and C.

5.3 Power Considerations for Overall Survival

The secondary endpoint Overall survival will be tested hierarchically after EFS with the same overall alpha as for the EFS comparison (two-sided 4% if the pCR comparison is not significant or 5% if the pCR comparison is significant).

For the formal comparison of OS for nivolumab plus platinum doublet chemotherapy (Arm C) vs platinum doublet chemotherapy (Arm B), only participants concurrently randomized from 1:1:1 randomization in revised protocol 02 and 1:1 randomization in revised protocol 03 will be included. [redacted]

[redacted] The stopping boundaries at the interim and final OS analyses will be derived based on the exact number of events using Lan-DeMets alpha spending function with O'Brien-Fleming boundaries. This spending function is specific to OS and accounts for potential interim OS analyses even if they did not actually take place because of EFS non-significance[9].

[redacted]

Table 5.3-1 summarizes the key parameters of the power calculation for EFS and OS in the concurrently randomized participants from Arms B and C.

Table 5.3-1: Power Calculation for EFS and OS

	EFS<br>Arm C vs Arm B	OS<br>Arm C vs Arm B
Accrual	10/month during 1:1:1 randomization, then 15/month during 1:1 randomization<br>27 months	
Power	80%	[redacted]
Two-sided alpha	0.04	0.04
Hypothesized Median Control vs exp (months)	40 vs 61.5	[redacted]
Hypothesized Hazard ratio	0.65	[redacted]
Sample size for concurrent comparison	350	350
First EFS interim analysis (EFS IA1)	42 month after 1:1:1 randomization<br>111 events<br>Alpha boundary: 0.005	-
Second EFS (EFS IA2) and first OS (OS IA1) interim analysis	54 month after 1:1:1 randomization<br>148 events<br>Alpha boundary: 0.017	54 month after 1:1:1 randomization<br>[redacted]

Table 5.3-1: Power Calculation for EFS and OS

	EFS<br>Arm C vs Arm B	OS<br>Arm C vs Arm B
Final EFS (EFS FA) and second OS (OS IA2) interim analysis	69 month after 1:1:1 randomization<br>185 events<br>Alpha boundary: 0.034	69 month after 1:1:1 randomization<br>[redacted]
Final OS analysis (OS FA)	-	[redacted]

OS among all randomized participants on Arms B and C concurrently randomized after 1:1:1 randomization will be tested. The distribution of OS will be compared between Arm C vs Arm B via a 2-sided, log rank test stratified by the randomization stratification factor (ie, PD-L1, disease stage [IB/II vs IIIA] and gender). The hazard ratio and the corresponding (1-adjusted alpha) confidence interval will be estimated for Arm C vs Arm B in a stratified Cox proportional hazards model using the randomized arm as a single covariate. The OS curves for each randomized arm will be estimated using the Kaplan-Meier (KM) product-limit method using a log-log transformation. In addition, OS yearly rates will be estimated using KM estimates on the OS curve for each randomized arm provided a minimum follow-up is longer than the time point to generate the rate. Associated 2-sided 95% CIs will be calculated using the Greenwood formula (using log-log transformation).

5.4 Analyses Timing Projections

Assuming an accrual rate of 10 participants (all comers) a month between Arms B and C during 1:1:1 randomization (about 10 months), and 15 participants per month during 1:1 randomization afterwards, it will take

- Approximately 30 months (27 months to complete the accrual plus 3 months for them to have opportunity for surgery) from start of 1:1:1 randomization to conduct the pCR analysis. This is about 36 months from FPFV of the study.
- Approximately 42 months when 111 events on Arms B and C (after start of 1:1:1 randomization) are observed for the first interim EFS analysis. This is about 48 months from FPFV of the study.
- Approximately 54 months when 148 events on Arms B and C (after start of 1:1:1 randomization) are observed for the second interim EFS analysis. This is about 60 months from FPFV of the study. In case of significant EFS, OS will also be tested at that time (OS IA1).
- Approximately 69 months when 185 events on Arms B and C (after start of 1:1:1 randomization) are observed for the final EFS analysis. This is about 75 months from FPFV of the study. In case of significant EFS, OS will also be tested at that time (OS IA2).

[redacted]

Table 5.4-1 [redacted] summarize the scheduled analysis, criteria, and projected timeline.

Table 5.4-1: Scheduled Analyses, Criteria, and Projected Timelines for Formal Analyses of pCR and EFS

Scheduled Analysis	Criteria and Population	Projected timeline	Formal Analysis
pCR analysis	When participants on Arms B and C have an opportunity for surgery	30 months after 1:1:1 randomization (36 months from FPFV)	**pCR comparison Arms C vs B**, alpha=0.01.
First interim EFS analysis	111 EFS events on Arms B and C after start of 1:1:1 randomization	42 months after 1:1:1 randomization (48 months from FPFV)	**EFS comparison Arms C vs B**<br>The stopping boundaries is based on Lan-DeMets alpha spending function with O'Brien-Fleming boundaries: nominal p-value cutoff: 0.005 if EFS comparison alpha is 0.04, or 0.008 if EFS comparison alpha is 0.05.

Table 5.4-1: Scheduled Analyses, Criteria, and Projected Timelines for Formal Analyses of pCR and EFS

Scheduled Analysis	Criteria and Population	Projected timeline	Formal Analysis
Second interim EFS analysis<br>First interim OS analysis (a)	148 EFS events on Arms B and C after start of 1:1:1 randomization (b)	54 months after 1:1:1 randomization (60 months from FPFV)	The stopping boundaries is based on Lan-DeMets alpha spending function with O'Brien-Fleming boundaries:<br>**EFS comparison Arms C vs B**<br>nominal p-value cutoff is 0.017 if EFS comparison alpha is 0.04, or 0.022 if EFS comparison alpha is 0.05.<br>**OS comparison Arms C vs B** (if EFS significant)<br>[redacted]
Final EFS analysis<br>Second interim OS analysis (a)	185 EFS events on Arms B and C after start of 1:1:1 randomization (b)	69 months after 1:1:1 randomization (75 months from FPFV)	The stopping boundaries is based on Lan-DeMets alpha spending function with O'Brien-Fleming boundaries:<br>**EFS comparison Arms C vs B**<br>nominal p-value cutoff is 0.034 if EFS comparison alpha is 0.04, or 0.042 if EFS comparison alpha is 0.05.<br>**OS comparison Arms C vs B** (if EFS significant)<br>[redacted]
Final OS analysis	[redacted]		**OS comparison Arms C vs B** (if EFS significant)<br>The stopping boundaries is based on Lan-DeMets alpha spending function with O'Brien-Fleming boundaries: [redacted]

Approved v1.0 [redacted]

Table 5.4-1: Scheduled Analyses, Criteria, and Projected Timelines for Formal Analyses of pCR and EFS

Scheduled Analysis	Criteria and Population	Projected timeline	Formal Analysis
			[redacted]

(a) If EFS reaches significance at earlier Interim EFS, only Interim OS will be conducted, EFS will not be formally re-tested.

(b) If EFS reaches significance at earlier Interim EFS, Interim OS analyses will be triggered by the number of OS events.

[redacted]

6 STUDY PERIODS, TREATMENT REGIMENS AND POPULATIONS FOR ANALYSES

6.1 Study Periods

- Baseline period:
  - Baseline evaluations or events will be defined as evaluations or events that occur before the date and time of the first dose of study treatment. Evaluations (laboratory tests, pulse oximetry and vital signs) on the same date and time of the first dose of study treatment will be considered as baseline evaluations. Events (AEs) on the same date and time of the first dose of study treatment will not be considered as pre-treatment events.
  - In cases where the time (onset time of event or evaluation time and dosing time) is missing or not collected, the following definitions will apply:
    - ♦ Pre-treatment AEs will be defined as AEs with an onset date prior to but not including the day of the first dose of study treatment;

- ♦ Baseline evaluations (laboratory tests, pulse oximetry and vital signs) will be defined as evaluations with a date on or prior to the day of first dose of study treatment.
- – If there are multiple valid observations in the baseline period, then the latest non missing observation will be used as the baseline in the analyses. If multiple observations exist on the latest collection date (and time if collected), the record with the latest data entry date and time will be used. If multiple observations exist on the latest collection date (and time if collected) and data entry date and time, then the first observation is used as baseline, unless otherwise specified.
  - ♦ For PD-L1, non-missing is identified as those with quantifiable test result. After applying the rule above, if there are no records with a quantifiable test result, then select those with indeterminate result (“INDETERMINATE”). If there are no records with indeterminate test result, then select those with unavailable result (“NOT EVALUABLE”). If there are no records with unavailable test result, then select those which are not reported or not available result (all other records).
  - ♦ For Anti-Drug Antibody (ADA), the baseline record of nivolumab and ipilimumab immunoglobulin (IMG) evaluation must be less than the date and time of the first nivolumab and ipilimumab dose date and time.

- Post baseline period:
  - – Neoadjuvant on-treatment AEs will be defined as AEs with an onset date and time on or after the date and time of the first dose of neoadjuvant study treatment (or with an onset date on or after the day of first dose of neoadjuvant study treatment if time is not collected or is missing). For participants who are off neoadjuvant study treatment, AEs will be included if event occurred within a safety window of 30 days (or 100 days depending on the analysis) after the last dose of neoadjuvant study treatment. No “subtracting rule” will be applied when an AE occurs both pre-treatment and post-treatment with the same preferred term and grade.
  - – Neoadjuvant on-treatment evaluations (laboratory tests, pulse oximetry and vital signs) will be defined as evaluations taken after the day (and time, if collected and not missing) of first dose of neoadjuvant study treatment. For participants who are off neoadjuvant study treatment, evaluations should be either within a safety window of 30 days (or 100 days depending on the analysis) after the last dose of neoadjuvant study treatment.
  - – Adjuvant on-treatment AEs will be defined as AEs with an onset date and time on or after the date and time of the first dose of adjuvant systemic study treatment (or with an onset date on or after the day of first dose of adjuvant systemic study treatment if time is not collected or is missing). For participants who are off adjuvant study treatment, AEs will be included if event occurred within a safety window of 30 days after the last dose of adjuvant study treatment.

6.2 Treatment Regimens

The treatment group “as randomized” corresponds to the treatment group assigned by the Interactive Response Technology (IRT) system.

The treatment group “as treated” will be same as the treatment group “as randomized” by IRT unless a subject received the incorrect study treatment for the entire period of treatment, in which case the subject’s treatment group “as treated” will be defined as the incorrect study treatment.

Unless otherwise specified, the safety analysis will be based on the treatment group "as treated".

Unless otherwise specified, the efficacy analysis will be based on the treatment group "as randomized".

The treatment arms are as follows:

- Arm A: nivolumab 3 mg/kg plus ipilimumab 1 mg/kg.
- Arm B: platinum doublet chemotherapy
- Arm C: nivolumab 360 mg plus platinum doublet chemotherapy

6.3 Populations for Analyses

- All Enrolled Participants: All participants who signed an informed consent form and were registered into the IRT.
- All Randomized Participants: All participants who were randomized to any treatment group in the study.
- All Treated Participants: All participants who received at least one dose of any study medication in neoadjuvant setting. This is the primary dataset for drug exposure and safety analysis for arm A.
- All Concurrently Randomized Participants in Arms B and C: All participants concurrently randomized on Arms B and C as of the 1:1:1 randomization. This will be the **primary analysis population** for efficacy.
- All Concurrently Randomized Participants in Arms A and B: All participants concurrently randomized on Arms A and B.
- All Treated Participants from the Concurrently Randomized Arms B and C: All participants concurrently randomized on Arms B and C as of the 1:1:1 randomization who received at least one dose of any study medication in the neoadjuvant setting. This will be the primary analysis population for drug exposure and safety for arms B and C.
- Immunogenicity (ADA evaluable) Subjects: All treated subjects with baseline and at least 1 post-baseline immunogenicity assessment (Treatment arms A and C only) for nivolumab and ipilimumab respectively.
- Tumor Tissue TMB evaluable subjects: All randomized subjects from the global study population with baseline evaluable tumor tissue TMB (non-missing numeric).
- Concurrently randomized subjects from arms B and C (as of the 1:1:1 randomization) is considered at the site level basis, when the site switched to the revised protocol. In practice, this includes subjects randomized on the randomization lists from the 1:1:1 randomization (revised protocol 02) and the subsequent 1:1 randomization between B and C only (revised protocol 03).

Unless otherwise specified, all analyses will be performed using the treatment arm as randomized (intent to treat), with the exception of dosing and safety, for which the treatment arm as received will be used.

7 STATISTICAL ANALYSES

7.1 General Methods

Unless otherwise specified, analyses will be performed by treatment group (as randomized or as treated, depending on the analysis) for all concurrently randomized participants from Arms B and C. Descriptive analyses will also be produced for Arm A. Participants in Arm B randomized in the initial protocol will only be reported in listings and in the consistency by randomization period analyses (Sections 7.3.7, 7.6.8 and 7.7.19).

Unless otherwise noted, discrete variables will be tabulated by the frequency and proportion of subjects falling into each category, grouped by treatment. Percentages given in these tables will be rounded to the first decimal and, therefore, may not always sum to 100%. Percentages less than 0.1 will be indicated as '< 0.1'. If a missing category is not being presented in the data display, only those subjects with non-missing values for the parameter being assessed are included in the percentage calculation. Confidence intervals for binomial proportions will be derived using the Clopper-Pearson method[10].

Continuous variables will be summarized by treatment group using the mean, standard deviation, median, minimum, and maximum values and quartiles.

Time-to-event variables (e.g. time-to resolution, EFS) will be analyzed using the Kaplan-Meier technique. When specified, the median will be reported along with 95% CI using Brookmeyer and Crowley method[11] (using log-log transformation for constructing the confidence intervals[12]). Rates at fixed timepoints (e.g., OS at 12 months) will be derived from the Kaplan Meier estimate along with their corresponding log-log transformed confidence intervals[13].

Unless otherwise specified, the stratified hazard ratio between 2 treatment groups along with CI will be obtained by fitting a stratified Cox model with the treatment group variable as unique covariate. Stratification factors per IRT (PD-L1 expression (≥1% or <1%/not evaluable/indeterminate), disease stage (IB/II vs IIIA) and gender).

Unless otherwise specified, the stratified log-rank test will be performed to test the comparison between time to event distributions (OS and EFS). Stratification factors will be as described above.

The p-values from sensitivity analyses for efficacy endpoints, if presented, are for descriptive purpose only and not adjusted for multiplicity.

The conventions to be used for imputing missing and partial dates for analyses requiring dates are described in Section 8.

Note that in this document the terms "participant" and "subject" are used interchangeably. Terminology used in CSR will follow the BMS standard at the time of CSR.

Additional analyses by country or region may be conducted separately for country specific submissions.

Approved v1.0 [redacted]

7.1.1 *Adverse Events, Serious Adverse Events, Multiple Events, Select Adverse Events, Other Events of Special Interest and Immune-Mediated Adverse Events*

Drug-related AEs are those events with relationship to study drug "Related", as recorded on the CRF. If the relationship to study drug is missing, the AE will be considered as drug-related.

Serious adverse events consist of AEs deemed serious by the Investigator and flagged accordingly in the CRF and clinical database.

Adverse events leading to study drug discontinuation are AEs with action taken regarding study drug(s) = "Drug was discontinued". This option is selected when at least one agent from the regimen is discontinued.

Adverse events leading to dose delay are AEs with action taken regarding study drug(s) = "Drug was delayed".

Adverse events leading to dose reduction are AEs with action taken regarding study drug(s) = "Dose was reduced".

Adverse events will be coded using the Medical Dictionary for Regulatory Activities (MedDRA), and the most recent version of the dictionary at the time of the database lock will be used. Adverse events results will be graded for severity using NCI Common Terminology Criteria for Adverse Events (CTCAE) and the version of the criteria specified in the protocol will be used (version 4).

In the AE summary tables, unless otherwise specified, subjects will be counted only once at the Preferred Term (PT), only once at the System Organ Class (SOC), and only once at subject level for the counting of total number of subjects with an AE. The AE tables will be sorted by the SOCs and then PTs. SOC will be ordered by descending frequency overall and then alphabetically. PTs will be ordered within SOC by descending frequency overall and then alphabetically. The sorting will be done based on the 'Any Grade' column of the experimental arm when arms are presented side-by-side.

Unless otherwise specified, the AE summary tables will be restricted to on-treatment events regardless of the causality.

Analyses that take into account the multiple occurrences of a given adverse event will be conducted (see Section 7.7.10). To prepare these analyses, the CRF data will be processed according to standard BMS algorithms[14] in order to collapse adverse event records into unique records based on the preferred term. These data will be presented as the rate per 100 person-years of exposure. These analyses will take into account all on-treatment events (allowing more than 1 event per subject) and the total exposure time. The person-year exposure will be computed as the sum over the subjects' exposure expressed in years where the exposure time is defined as

- (Date of last dose of study treatment - date of first dose of study treatment + 31 days (or 101 days, depending on the analysis))/365.25, for subject who are off study treatment and were followed for at least 30 days (or 100 days, depending on the analysis) after last dose of study treatment.

Approved v1.0 [redacted]

- (Last known alive date - date of first dose of study treatment +1)/365.25, for subjects who are still on-treatment or who are off study treatment and were followed less than 30 days (or 100 days depending on the analysis) after last dose of study treatment.

7.1.1.1 Select Adverse Events

The select Adverse Events (select AEs) consist of a list of preferred terms grouped by specific category (e.g. pulmonary events, gastrointestinal events categories, etc.). AEs that may differ from or be more severe than AEs caused by non-immunotherapies and AEs whose early recognition and management may mitigate severe toxicity are included as select AEs. Categories of select AEs may include subcategories (e.g. adrenal disorders, diabetes, pituitary disorders, and thyroid disorders are subcategories of the endocrine event category).

The list of MedDRA preferred terms used to identify select adverse events is revisited quarterly and updated accordingly. The preferred terms used for the selection at the time of the database lock will be provided by categories/subcategories.

In addition to the frequency and worst severity of select AEs, time-to onset, time-to resolution, and time-to resolution where immune modulating medication was initiated will be analyzed for each specific category/subcategory of drug-related select AEs when applicable.

Further details on the definitions time-to onset and time-to resolution are described in APPENDIX 1.

7.1.1.2 Other Events of Special Interest

Other events of special interest (OEOSI) consist of a list of preferred terms grouped by specific category (e.g. Myositis Event, Myocarditis Event, Demyelination Event, Guillain-Barre Syndrome, Pancreatitis Event, Uveitis Event, Encephalitis Event, Myasthenic Syndrome, Rhabdomyolysis Event, Graft Versus Host Disease). The list of MedDRA preferred terms used to identify OEOSI is revisited quarterly and updated accordingly. The preferred terms used for the selection at the time of the database lock by categories will be provided.

7.1.1.3 Immune-Mediated Adverse Events

In order to further characterize AEs of special clinical interest, analysis of immune-mediated AEs (IMAE) will be conducted. IMAEs are specific events (or groups of PTs describing specific events) that include pneumonitis, diarrhea/colitis, hepatitis, nephritis/renal dysfunction, rash, endocrine (adrenal insufficiency, hypothyroidism/thyroiditis, hypothyroidism, thyroiditis, hyperthyroidism, diabetes mellitus, and hypophysitis), and other specific events, considered as potential immune-mediated events by investigator that meet the definition summarized below:

- those occurring within 100 days of the last dose,
- regardless of causality,
- treated with immune-modulating medication (of note, endocrine AEs such as adrenal insufficiency, hypothyroidism/thyroiditis, hypothyroidism, thyroiditis, hyperthyroidism, diabetes mellitus, and hypophysitis are considered IMAEs regardless of immune-modulating medication use, since endocrine drug reactions are often managed without immune-modulating medication).

- with no clear alternate etiology based on investigator assessment, or with an immune-mediated component

The list of MedDRA preferred terms used to identify IMAEs is revisited quarterly and updated accordingly. The preferred terms used for the selection at the time of the database lock by categories will be provided.

7.1.2 Laboratory Tests

Clinical laboratory parameters (hematology, serum chemistry and electrolytes) will be evaluated.

Laboratory tests will be graded using the NCI Common Terminology Criteria, and the most recent version of the criteria at the time of the database lock will be used.

Clinical laboratory data will be analyzed using International System of Units (SI). Analyses will be repeated using US conventional units.

In the laboratory summary tables, unless otherwise specified, subjects will be counted only once for each lab parameter according to their worst on treatment CTC grade (worst being the highest CTC grade). The laboratory tables and listings will be sorted by laboratory category, laboratory subcategory and laboratory test code sequence number.

7.1.3 Immunogenicity Data

Blood samples for immunogenicity analysis will be collected from subjects assigned to the experimental treatment group(s) according to the protocol schedule. Samples will be evaluated for development of Anti-Drug Antibody (ADA) by a validated electrochemiluminescent (ECL) immunoassay.

7.2 Study Conduct

Unless otherwise specified, analyses will be performed by treatment group as randomized for all concurrently randomized participants from Arms B and C. Descriptive analyses will also be produced for Arm A. Participants in Arm B randomized in the initial protocol will only be reported in listings.

For analysis based on enrolled subjects, the analysis population will consist of the all enrolled population.

7.2.1 Accrual

Enrollment and randomization by country and site, and enrollment and randomization by month will be summarized and listed for all enrolled and randomized subjects.

7.2.2 Relevant Protocol Deviations

Unless otherwise specified, analyses will be performed by treatment group as randomized for all concurrently randomized participants from Arms B and C. Descriptive analyses will also be produced for Arm A. Participants in Arm B randomized in the initial protocol will only be reported in listings.

Eligibility:

- Inadequate disease stage: presence of locally advanced unresectable (regardless of stage), stage IIIB or metastatic disease (stage IV) or stage IA disease.
- Subjects without measurable disease at baseline as per investigator.
- Subject with baseline ECOG performance status > 1.

On-study:

- Subjects receiving any concurrent anti-cancer therapy (chemotherapy, hormonal therapy, immunotherapy, radiation therapy, cancer related surgery (except definitive surgery), standard or investigational agents for treatment of cancer) outside of the protocol-specified neoadjuvant and adjuvant therapy (systemic and randiotherapy) while on study therapy (i.e. neoadjuvant or protocol adjuvant treatment).
- Subjects whose “as treated” arm different than their as randomized arm (subjects who received the wrong treatment for the entire neoadjuvant treatment period, excluding the never treated)

7.3 Study Population

Unless otherwise specified, analyses will be performed by treatment group as randomized for all concurrently randomized participants from Arms B and C. Descriptive analyses will also be produced for Arm A. Participants in Arm B randomized in the initial protocol will only be reported in listings.

7.3.1 Subject Disposition

The total number of subjects enrolled (randomized or not randomized) will be presented along with the reason for not being randomized. This analysis will be performed on the all enrolled subjects population.

Number of subjects randomized but not treated along with the reason will be tabulated by treatment group as randomized.

Number of subjects who discontinued study treatment along with corresponding reason will be tabulated by treatment group as treated. Reason for discontinuation will be derived from subject status CRF page. This analysis will be restricted to the all treated subjects population.

A by-subject listing for all treated subjects will be provided showing the subject’s off treatment date along with the reason for going off treatment period. A by-subject listing for all enrolled subjects will also be provided, showing whether the subject was randomized/treated along with the reason for not being randomized/treated.

7.3.2 Demographics and Other Baseline Characteristics

The following demographic and baseline characteristics will be summarized and listed by treatment group as randomized:Age (descriptive statistics)

- Age (continuous)
- Age categorization (< 65, ≥ 65 and < 75, ≥ 75 and < 85, ≥ 85, ≥ 75, ≥ 65)
- Sex (male vs. female, CRF)
- Sex (male vs. female, IRT)

- Race (white, black , asian [asian Indian, Chinese, Japanese, asian other], American Indian or Alaska Native, Native Hawaiian or Other Pacific Islander, Other)
- Ethnicity (Hispanic/Latino, Not Hispanic/Latino, Unknown) - Required for US subjects only, but any available data will be presented
- Country by geographic region (North America, Europe, Asia, Rest of World)

The following baseline disease characteristics will be summarized by treatment group as randomized.

- Baseline ECOG performance status
- Baseline weight
- Tobacco use (Never Smoker, Current/Former, Unknown).
- Electronic Cigarette Use (Never Smoker, Current/Former, Unknown).
- Disease stage at study entry (CRF)
- Disease stage at study entry (IRT)
- Cell type (histology) at study entry (squamous cell carcinoma, non-squamous: adenocarcinoma, large cell carcinoma, broncho-alveolar carcinoma, other)
- Time from current NSCLC diagnosis to randomization
- Sites of diseases (all lesions) per BICR,
- Number of disease sites per subject (all lesions) per BICR
- Number of target lesions, non-target lesions and disease sites at baseline as per BICR
- Sum of the diameters of target lesions as baseline per BICR
- PD-L1 expression subgroups (CRF, (<1%, >=1%, 1-49%, >=50%, inderterminate, not evaluable))
- PD-L1 expression subgroups (IRT, (<1%, >=1%/indeterminate/not evaluable)
- Tumor Tissue TMB subgroups (≥12.3 mut/MB, < 12.3 mut/MB, not evaluable)

Summary table (cross-tabulation) by treatment group for stratification factor will be provided to show any discrepancies between what was reported through IRT vs. CRF/Clinical database at baseline. This summary will be performed based on all randomized subjects.

- PD-L1 status (IRT vs. clinical database)
- Disease stage (IRT vs CRF data)
- Gender (IRT vs CRF data)

A listing of randomization scheme presenting randomized treatment group and as treated treatment group will be provided for all randomized subjects.

7.3.3 Medical History

A by-subject listing of general medical history for all randomized subjects will be provided.

7.3.4 Prior Therapy

- Prior Cancer therapies:
- Prior systemic cancer therapy will be summarized by treatment group and overall and listed by subject.
- Prior radiotherapy and prior surgery related to cancer will be summarized by treatment group and overall and listed by subject.
- Other Prior therapy:
- Prior/current non-study medication classified by anatomic and therapeutic classes.

Agents and medication will be reported using the generic name. A listing by subject will also be provided.

7.3.5 Physical Examinations

Subjects with abnormal baseline physical examination will be listed by subject.

7.3.6 Baseline Physical Measurements

Baseline physical measurements will be listed by subject.

7.3.7 Consistency of Demographics and Baseline Characteristics by Randomization Period

Consistency of population amongs the different randomization periods will be examined by summarizing the characteristics listed in Section 7.3.2 by treatment arm as randomized in the the different randomization period: before revised protocol 02 (1:1 A vs B), under revised protocol 02 (1:1:1 A vs B vs C) and after revised protocol 03 (1:1 B vs C).

Concurrent randomization is considered at the site level basis, when the site switched to the revised protcol. In practice, this includes subjects randomized on the randomization lists from the 1:1:1 randomization (revised protocol 02) and the subsequent 1:1 randomization between B and C only (revised protocol 03).

7.4 Extent of Exposure

Listings will include all available exposure data. Analyses will be performed by treatment group "as treated" in all treated subjects and for all treated participants from concurrently randomized Arms B and C, unless otherwise specified. Descriptive analyses will also be produced for Arm A. Participants in Arm B randomized in the initial protocol will only be reported in listings.

7.4.1 Administration of Study Therapy

The following parameters will be summarized (descriptive statistics) by treatment group:

- Number of neoadjuvant doses received by drug
- Cumulative dose by drug in neoadjuvant
- Relative dose intensity (%) by drug in neoadjuvant using the following categories: < 50%; 50 - < 70%; 70 - < 90%; 90 - < 110%; ≥ 110%

- Number of subjects who received protocol specified adjuvant systemic therapy number of adjuvant doses received by drug for subjects who received adjuvant therapy.
- The frequency of subjects receiving carboplatin instead of cisplatin in the regimens other than carboplatin-paclitaxel will be reported and the reason for not using cisplatin for regimens other that carboplatin-paclitaxel will be summarized.

A by-subject listing of dosing of study medication (record of study medication, infusion details, and dose changes) and a listing of batch numbers will be also provided.

- Number of subjects who received adjuvant radiotherapy, including radiotherapy type, number of doses and total cumulative dose.

Table 7.4.1-1 to Table 7.4.1-6 summarize the key parameters used to calculate dosing data.

Table 7.4.1-1: Study Therapy Parameter Definitions- Nivolumab and Ipilimumab

	Nivolumab	Nivolumab	Ipilimumab
Dosing schedule per protocol	3 mg/kg every 2 weeks	360 mg every 3 weeks	1 mg/kg on first cycle
Dose	*Dose (mg/kg)* is defined as Total Dose administered (mg)/Most recent weight (kg). Dose administered in mg at each dosing date and weight are collected on the CRF.	*Dose (mg)* is defined as Total Dose administered (mg) at each dosing date as collected on the CRF.	*Dose (mg/kg)* is defined as Total Dose administered (mg)/Most recent weight (kg). Dose administered in mg at each dosing date and weight are collected on the CRF.
Cumulative Dose	*Cum dose (mg/kg) is* sum of the doses (mg/kg) administered to a subject.	*Cum dose (mg)* is the sum of the doses (mg) administered to a subject.	*Cum dose (mg/kg) is* sum of the doses (mg/kg) administered to a subject.
Relative dose intensity (%)	Cum dose (mg/kg)/[(Last Nivolumab dose date - Nivolumab start dose date + 14) x 3/14] x 100	Cum dose (mg)/[(Last Nivolumab dose date - Nivolumab start dose date + 21) x 360/21] x 100	Cum dose (mg/kg) x 100

Table 7.4.1-2: Study Therapy Parameter Definitions - Regimen 1: Vinorelbine/Cisplatin

	Vinorelbine	Cisplatin
Dosing schedule per protocol	25 mg/m^2 or 30 mg/m^2 on Day1 and Day8 of a 3 week cycle.	75mg/ m^2 on Day 1 of a 3 week cycle
Dose	*Dose (mg/m^2)* is defined as Total Dose administered (mg)/Most recent BSA. Dose administered in mg at each dosing date is collected on the CRF and BSA is derived from most recent weight and	*Dose (mg/m^2)* is defined as Total Dose administered (mg)/Most recent BSA. Dose administered in mg at each dosing date is collected on the CRF and BSA is derived from most recent weight and

Table 7.4.1-2: Study Therapy Parameter Definitions - Regimen 1: Vinorelbine/Cisplatin

	Vinorelbine	Cisplatin
	baseline height also collected on the CRF.	baseline height also collected on the CRF.
Cumulative Dose	*Cum dose (mg/ m²) is* sum of the doses (mg/ m^2) administered to a subject.	*Cum dose (mg/m²) is* sum of the doses (mg/m²) administered to a subject.
Relative dose intensity (%)	Cum dose (mg/m²)/[(First Vinorelbine dose date in the last cycle - Vinorelbine Start dose date + 21) x 50/21] x 100 or Cum dose (mg/m²)/[(First Vinorelbine dose date in the last cycle - Vinorelbine Start dose date + 21) x 60/21] x 100	Cum dose (mg/m²)/[(Last Cisplatin dose date - Start Cisplatin dose date + 21) x 75/21] x 100

Table 7.4.1-3: Study Therapy Parameter Definitions - Regimen 2: Docetaxel/Cisplatin

	Docetaxel	Cisplatin
Dosing schedule per protocol	60 mg/m^2 or 75 mg/m^2 on Day1 of a 3 week cycle.	75mg/ m² on Day 1 of a 3 week cycle
Dose	*Dose (mg/m²)* is defined as Total Dose administered (mg)/Most recent BSA. Dose administered in mg at each dosing date is collected on the CRF and BSA is derived from most recent weight and baseline height also collected on the CRF.	*Dose (mg/m²)* is defined as Total Dose administered (mg)/Most recent BSA. Dose administered in mg at each dosing date is collected on the CRF and BSA is derived from most recent weight and baseline height also collected on the CRF.
Cumulative Dose	*Cum dose (mg/ m²) is* sum of the doses (mg/ m^2) administered to a subject.	*Cum dose (mg/m²) is* sum of the doses (mg/m²) administered to a subject.
Relative dose intensity (%)	Cum dose (mg/m²)/[(First Docetaxel dose date in the last cycle - Docetaxel Start dose date + 21) x 60/21] x 100 or Cum dose (mg/m²)/[(First Docetaxel dose date in the last cycle - Docetaxel Start dose date + 21) x 75/21] x 100	Cum dose (mg/m²)/[(Last Cisplatin dose date - Start Cisplatin dose date + 21) x 75/21] x 100

Approved v1.0 [redacted]

Table 7.4.1-4: Study Therapy Parameter Definitions - Regimen 3: Gemcitabine/Cisplatin

	Gemcitabine	Cisplatin
Dosing schedule per protocol	1250 mg/m^2 or 1000 mg/m^2 on Day1 and Day8 of a 3 week cycle.	75mg/ m² on Day 1 of a 3 week cycle
Dose	*Dose (mg/m²)* is defined as Total Dose administered (mg)/Most recent BSA. Dose administered in mg at each dosing date is collected on the CRF and BSA is derived from most recent weight and baseline height also collected on the CRF.	*Dose (mg/m²)* is defined as Total Dose administered (mg)/Most recent BSA. Dose administered in mg at each dosing date is collected on the CRF and BSA is derived from most recent weight and baseline height also collected on the CRF.
Cumulative Dose	*Cum dose (mg/ m²) is* sum of the doses (mg/ m^2) administered to a subject.	*Cum dose (mg/m²) is* sum of the doses (mg/m²) administered to a subject.
Relative dose intensity (%)	Cum dose (mg/m²)/[(First Gemcitabine dose date in the last cycle - Gemcitabine Start dose date + 21) x 2500/21] x 100 or Cum dose (mg/m²)/[(First Gemcitabine dose date in the last cycle - Gemcitabine Start dose date + 21) x 2000/21] x 100	Cum dose (mg/m²)/[(Last Cisplatin dose date - Start Cisplatin dose date + 21) x 75/21] x 100

Table 7.4.1-5: Study Therapy Parameter Definitions - Regimen 4: Pemetrexed/Cisplatin

	Pemetrexed	Cisplatin
Dosing schedule per protocol	500 mg/ m² every 3 weeks	75mg/ m² every 3 weeks
Dose	*Dose (mg/m²)* is defined as Total Dose administered (mg)/Most recent BSA. Dose administered in mg at each dosing date is collected on the CRF and BSA is derived from most recent weight and baseline height also collected on the CRF.	*Dose (mg/m²)* is defined as Total Dose administered (mg)/Most recent BSA. Dose administered in mg at each dosing date is collected on the CRF and BSA is derived from most recent weight and baseline height also collected on the CRF.
Cumulative Dose	*Cum dose (mg/m²) is* sum of the doses (mg/m²) administered to a subject.	*Cum dose (mg/m²) is* sum of the doses (mg/m²) administered to a subject.
Relative dose intensity (%)	Cum dose (mg/m²)/[(Last Pemetrexed dose date - Pemetrexed Start dose date + 21) x 500/21] x 100	Cum dose (mg/m²)/[(Last Cisplatin dose date - Cisplatin Start dose date + 21) x 75/21] x 100

Approved v1.0 [redacted]

Table 7.4.1-6: Study Therapy Parameter Definitions - Regimen 5: Paclitaxel/Carboplatin

	Paclitaxel	Carboplatin
Dosing schedule per protocol	200 mg/ m^2 or 175 mg/ m^2 every 3 weeks	AUC 5 or 6 every 3 weeks
Dose	*Dose (mg/m²)* is defined as Total Dose administered in mg at each dosing date is collected on the CRF and BSA is derived from most recent weight and baseline height also collected on the CRF.	*Dose (AUC)* is defined as Total Dose administered (mg)/(creatinine clearance +25). Dose administered in mg at each dosing date is collected on the CRF and creatinine clearance derived from the CRF data and capped at 125 mL/min
Cumulative Dose	*Cum dose (mg/m²) is* sum of the doses (mg/m²) administered to a subject.	*Cum dose (AUC) is* sum of the doses (AUC) administered to a subject.
Relative dose intensity (%)	Cum dose (mg/m²)/[(Last paclitaxel dose date - paclitaxel Start dose date + 21) x 200/21] x 100 or Cum dose (mg/m²)/[(Last paclitaxel dose date - paclitaxel Start dose date + 21) x 175/21] x 100	Cum dose (AUC)/[(Last dose date of Carbo - Start dose date of Carbo + 21) x 6/21] x 100 or Cum dose (AUC)/[(Last dose date of Carbo - Start dose date of Carbo + 21) x 5/21] x 100

Where the creatinine clearance will be calculated using Cockroft-Gault formula, defined as:

$$CrCL(ml/min) = \frac{(140 - age(in\ years)) * weight(in\ kg)}{72 * serum creatinine(in\ mg/dL)}$$

for males and

$$CrCL(ml/min) = \frac{(140 - age(in\ years)) * weight(in\ kg)}{72 * serum creatinine(in\ mg/dL)} * 0.85$$

for females. The most recent weight will be used. If the computed creatinine clearance is more than 125 ml/min, then the creatinine clearance value should be capped at 125ml/min for dose exposure computations.

7.4.2 Modifications of Study Therapy

7.4.2.1 Dose Delay/Omission

Each study medication infusion may be delayed. A dose will be considered as actually delayed if the delay is exceeding 3 days (i.e., greater than or equal to 4 days from scheduled dosing date) for study medication. Since this is a calculated delay, it will also include omitted doses. Reason for dose delay/omission will be retrieved from CRF dosing pages.

The following parameters will be summarized by treatment group and by drug.

Approved v1.0 [redacted]

- Number of subjects with at least one dose delayed, the number of dose delays per subject, the reason for dose delay and the length of dose delay.
- Number of subjects with last dose omitted (without discontinuation of the other drugs of the regimen), reason for omission.

7.4.2.2 Infusion Interruptions and Rate Changes

Each study drug infusion can be interrupted and/or the IV infusion rate can be reduced. This information will be retrieved from CRF dosing pages

The following parameters will be summarized by treatment group and study drug:

- Number of subjects with at least one dose infusion interruption, the reason for interruption, and the number of infusion interruptions per subject.
- Number of subjects with at least one IV infusion rate reduction, the reason for reduction and the number of infusion with IV rate reduction per subject.

7.4.2.3 Dose Reductions

There will be no dose reductions of nivolumab and ipilimumab allowed. Dose of platinum doublet chemotherapy (Arms B and C) may be modified for toxicity. Dose levels of platinum doublet chemotherapy (Arms B and C) are defined in the protocol as follows:

Table 7.4.2.3-1: Dose Modifications of Chemotherapeutic Agents (Arms B and C)

Dose Level	Vinorelbine	Docetaxel	Gemcitabine	Pemetrexed	Cisplatin	Carboplatin	Paclitaxel
Starting dose	25 mg/m² or 30 mg/m²	60 mg/m² or 75 mg/m²	1000 mg/m² or 1250 mg/m²	500 mg/m²	75 mg/m²	AUC 5 or 6	175 or 200 mg/m²
First dose reduction	75% of strating dose	75% of starting dose	75% of starting dose	75% of starting dose	75% of starting dose	AUC 4 or 5	150 mg/m²
Second dose reduction	50% of starting dose	50% of starting dose	50% of starting dose	50% of starting dose	50% of starting dose	AUC 3 or 4	100 mg/m²
Third dose reduction	Discontinue	Discontinue	Discontinue	Discontinue	Discontinue	Discontinue	Discontinue

For any cycle, it will be defined as a dose reduction if the observed dose level (based on calculated administered dose) is below protocol specified dose level. Dose ranges for dose levels of platinum doublet chemotherapy are defined in Table 7.4.2.3-2.

Approved v1.0 [redacted]

Table 7.4.2.3-2: Calculated Dose Ranges and Related Dose Levels

Dose Level	Dose Range						
	Vinorelbine (mg/m²)	Docetaxel (mg/m²)	Gemcitabine (mg/m²)	Pemetrexed (mg/m²)	Cisplatin (mg/m²)	Carboplatin (AUC)	Paclitaxel (mg/m²)
Level 0	≥21.875 or ≥26.25	≥ 52.5 or ≥65.625	≥875 or ≥1093.75	≥437.5	≥65.625	≥4.5 or ≥5.5	≥153.125 or ≥175
Level -1	<21,875 and ≥ 15.625 or <26.25 and ≥ 18.75	<52.5 and ≥37.5 or < 65.625 and ≥46.875	<875 and ≥625 or <1093.75 and ≥781.25	<437.5 and ≥312.5	<65.625 and ≥46.875	<5.5 and ≥4.5 or <4.5 and ≥3.5	<175 and ≥125
Level -2	<15.625 or <18.75	<37.5 or < 46.875	<625 or < 781.25	<312.5	<46.875	<4.5 or <3.5	<125

The reason for dose reduction as reported by the investigator will be tabulated for all instances of dose reduction based on the Dose Change CRF page. A category 'Unknown' will be defined for all reductions with no reason reported by the investigator.

Chemotherapy dose reductions are permanent; once the dose of any chemotherapy agent is reduced, it may not be re-escalated in subsequent cycles.

The following will be summarized for chemotherapeutic agent arm only:

Number and percentage of subjects with at least one dose reduction and reason of the dose reduction, number and percentage of subjects with a dose reduction to dose level -1, number and percentage of subjects with a dose reduction to dose level -2.

7.4.3 Concomitant Medications

Concomitant medications, defined as medications other than study medications which are taken at any time on-treatment (i.e. on or after the first day of study therapy and within 100 days following the last dose of neoadjuvant study therapy or within 30 days following the last dose of adjuvant therapy, whichever is longest), will be coded using the WHO Drug Dictionary.

The following summary tables by treatment group will be provided:

- Concomitant medications (subjects with any concomitant medication, subjects by medication class and generic term).

Prior medications, defined as non-study medications with a start date before consent date, and current medications, defined as non-study medications with a start date before the first date of study medication and stop date after consent date, will be coded using the WHO Drug Dictionary.

The following summary table will be provided:

- Prior/current medications (subjects with any prior/current medication, subjects by medication class and generic term)

Approved v1.0 [redacted]

By-subject listings will accompany the tables.

7.4.3.1 Immune Modulating Medication

Immune modulating concomitant medications are medications entered on an immune modulating medication form or available from the most current pre-defined list of immune modulating medications. The list of anatomic class, therapeutic class and generic name used for the selection at the time of the database lock will be provided.

The percentage of subjects who received immune modulating concomitant medication for

- management of adverse event
- premedication
- other use
- any use
- management of drug-related select adverse event (any grade, grade 3-5) by select AE category/ subcategory
- management of IMAEs (any grade, grade 3-5) by IMAE category

will be reported separately for each treatment group (percentages of treated subjects by medication class and generic term).

For each category/subcategory of drug-related select AEs (any grade, grade 3-5) and IMAEs (any grade, grade 3-5), the following will be reported for each treatment group:

- The total immune modulating medication treatment duration (excluding overlaps), duration of high dose of corticosteroid, initial dose of corticosteroid, and tapering duration (summary statistics)

Duration represents the total duration the subject received the concomitant medication of interest. If the subject took the medication periodically, then DURATION in the summation of all use. Initial dose represents the dose of the concomitant medication of interest received at the start of the event. In the case multiple medications started on the same date, the highest equivalent dose is chosen and converted to mg/kg by dividing by the subject's recent weight.

These analyses, except the ones related to IMAEs will be conducted using the 30-day safety window. The analyses related to IMAEs will be conducted using the 100-day safety window.

7.4.3.2 Subsequent Cancer Therapy

Subsequent therapies are defined as Cancer therapies started on or after the first study drug dose or date of randomization if the subject is no treated, outside of the protocol defined adjuvant therapy (systemic and radiotherapy).

The following information pertaining to subsequent therapies will be summarized by treatment arm, as randomized:

- Number and percentage of subjects receiving subsequent therapies including:
- Subsequent systemic therapy by drug name
- Subsequent disease related surgery

- Subsequent radiotherapy for treatment of tumors

A by-subject listing of subsequent cancer therapy will also be produced for randomized subjects.

7.5 Definitive Surgery

Unless otherwise specified, analyses will be performed by treatment group as randomized for all concurrently randomized participants from Arms B and C. Descriptive analyses will also be produced for Arm A. Participants in Arm B randomized in the initial protocol will only be reported in listings.

The following parameters will be summarized:

- Disease Stage Prior to Surgery
- Subjects with clinical downstaging (lower stage prior to surgery vs baseline)
- Subjects with surgery
-

Subjects without surgery:

- Reason for cancelled surgery
-

Subjects with surgery:

- Delayed surgery (>6 weeks post last neoadjuvant dose, as reported in CRF), Reason for delay
- Duration of surgery
- Length of hospitalization for definitive surgery
- Method of surgery (Minimally invasive-thoracoscopic/robotic, Thoracotomy, Minimally innvasive to thoracotomy)
- Type of Surgery (Pneumonectomy, Lobectomy, Sleeve Lobectomy, Bilobectomy, Other)
- Surgery Outcome (R0, R1, R2, unknown)

Safety related to surgery analyses are described in section 7.7.1

7.6 Efficacy

Unless otherwise specified, analyses will be performed by treatment group as randomized for all concurrently randomized participants from Arms B and C. Descriptive analyses will also be produced for Arm A. Participants in Arm B randomized in the initial protocol will only be reported in listings and in analyses based on the All Concurrently Randomized Participants in Arms A and B population.

Unless stated otherwise, whenever a stratified analysis is specified, the following stratifications factors (recorded at randomization as per IRT) will be used:

- PD-L1 expression (≥1% or <1%/not evaluable/indeterminate)
- Disease stage (IB/II vs IIIA)
- Gender

Alpha (α) for the confidence intervals (CIs) for hazard ratios, odds ratios or difference of rates will be the same as nominal significance level for hypothesis testing. CIs for endpoints not tested will be at the two-sided 95% level. All p-values reported will be two-sided. P-values will be rounded to the fourth decimal place. Point estimates and confidence bounds for efficacy variables will be rounded to the second decimal place.

7.6.1 Type I Error Control

The overall alpha will be controlled using the following procedure. The overall alpha is primarily allocated to the two primary endpoints: 1% for pCR and 4% for EFS.

- The primary endpoint pCR will be tested at 1% alpha.
- If pCR is not significant, the primary endpoint EFS will be tested at 4%
- If pCR is significant, the 1% alpha will be re-allocated to the EFS primary endpoint which will be tested at 5% alpha level
- If EFS is significant, OS will be tested at the same level as EFS

EFS and OS (if EFS is significant) will be tested at planned interim and final analyses. Stopping boundaries will be calculated for each endpoint according to the observed number of events by Lan-DeMets alpha spending function with O'Brien-Fleming boundaries corresponding to an overall alpha of 4% or 5%. Given EFS and OS endpoints are tested using group sequential approach, overall hierarchical testing approach will be used where each endpoint will have its own specific Lan-DeMets alpha spending function with O'Brien-Fleming boundaries[9]. Also refer to Sections 5.2 and 5.3.

If the p-value crosses the boundary at the interim analysis (EFS or OS), the p-value from the interim stratified log-rank test will be considered the final analysis result for the study.

The secondary endpoints of Major Pathologic Response and Time to Death or Distant Metastases will be analyzed descriptively without hypothesis testing.

7.6.2 Analysis of Pathological Complete Response

7.6.2.1 Primary pCR Analysis

Formal analysis of pCR will occur after the 350 randomized participants in arms B and C from start of 1:1:1 randomization have an opportunity for surgery.

At pCR analysis, the primary analysis population is the concurrently randomization participants in arms B and C. PCR rate will be computed in each treatment group along with the exact 95% CI using Clopper-Pearson method.

The numerator is based on randomized participants achieving pCR in both tumor and lymph nodes, as assessed by independent pathological review (BIPR). The denominator is based on All Concurrently Randomized Participants in Arms B and C. Subjects who are no longer eligible for surgery, or who are on alternative anti-cancer therapy before surgery, or who discontinue before surgery or for whom pCR results are not available are all counted as non-responders.

pCR will be compared between concurrent arms B and C by the stratified Cochran Mantel-Haenszel (CMH) test using a 2-sided, 1% alpha level.

An estimate of the difference in pCR rates between the treatment groups along with the corresponding two sided 99% CI will also be computed using the following Cochran-Mantel-Haenszel (CMH) method of weighting, adjusting for stratification factors[15]. A two sided 99% CI for odds ratio of pCR between the treatment groups will also be computed.

Estimate of the difference in pCR between arms A and B (in the All Concurrently Randomized Participants in Arms A and B population) and odds ratio will also be provided together with corresponding 95% CI using the same methodology.

The analysis will be conducted by an independant statistician external to BMS and reviewed by the DMC. At the time of pCR analysis, EFS descriptive analyses (by investigator and by BICR) will be produced in the DMC closed report and might be shared with regulatory authorities. The communication of results will be tightly controlled and pre-specified in the DMC charter to maintain trial integrity.

7.6.2.2 Supportive Analyses of pCR

pCR sensitivity analyses will be performed with the following consideration:

- pCR analysis will be repeated for response evaluable subjects, where response evaluable subjects are subjects who had definitive surgery, and didn't start alternative anti-cancer therapy before surgery and pathologic samples results at surgery are evaluable. No p-value will be generated.

7.6.2.3 Subset Analyses of pCR

The influence of baseline and demographic characteristics on the treatment effect will be explored via exploratory subset analysis. BIPR assessment of pCR will be summarized for the following subgroups:

- Age category
    a) <65,
    b) >=65 and <75
    c) >=75 and <85
    d) >=85
    e) >=75
    f) >=65
- Sex (male, female), per IRT and per CRF
- Race (white, black, Asian, other)
- Region (North America, Europe, Asia, Rest of World)
- Baseline ECOG Performance Status (0, 1, >1)
- Tobacco use (current/former, never smoked, unknown)
- Disease stage (IB/II vs IIIA) per IRT and per CRF
- Baseline histology (squamous, non-squamous)
- PD-L1 subgroups (<1%, ≥1%, 1-49%, ≥50%, indeterminate, not evaluable)

Approved v1.0 [redacted]

- Tumor Tissue TMB Evaluable (≥10 mut/MB, < 10 mut/MB, Overall)
- Tumor Tissue TMB Not Evaluable
- Type of platinum therapy (cisplatin, carboplatin, subjects switching from cisplatin to carboplatin).
- Type of chemotherapy regimen in arm B (available in arm C (Gemcitabine-Cisplatin, Pemetrexed-Cisplatin, Paclitaxel-Carboplatin, not available in arm C (Vinorelbine-Cisplatin, Docetaxel-Cisplatin)) , based on first neoadjuvant cycle.

A forest plot of treatment effect on pCR per BIPR in the above subgroups will be produced. The un-weighted differences in pCR between concurrent arms B and C and corresponding 95% two-sided CI using the method of Newcombe, will be provided.

The analysis comparing treatment (i.e., pCR difference) will be conducted if the number of subjects in the subgroup category is more than 10.

7.6.2.4 Major Pathological Response Rate

MPR rate in concurrently randomized participants in arms B and C will be computed in each treatment group along with the exact 95% CI using Clopper-Pearson method. An estimate of the difference and odds ratio in MPR rates between concurrent arms B and C and corresponding 95% CI will be calculated using CMH methodology and adjusted by stratification factors.

In addition, an MPR analysis will be conducted on all Concurrently Randomized Participants from Arms B and C (will be produced at a later lock when all subjects had opportunity for surgery; e.g. at EFS analysis lock).

Estimate of the difference in MPR between arms A and B (in the All Concurrently Randomized Participants in Arms A and B population) and odds ratio will also be provided together with corresponding 95% CI.

Subset analyses by PDL1 status will be performed (PD-L1<1% PD-L1 ≥ 1%, PD-L1 1-49%, PD-L1 ≥ 50%, not evaluable/indeterminate) and by Tumor TMB (≥12.3 mut/MB, < 12.3 mut/MB, Overall, not evaluable).

7.6.2.5 Additional Pathological Related Analyses

Descriptive analyses of pCR and MPR, % tumor area with viable tumor cells in the tumor region and in lymph nodes separately will be provided.

7.6.2.6 Clinical Response Rate

Clinical response rate (cRR) by BICR will be summarized by treatment arm. cRR is defined as proportion of randomized participants whose radiologic response at the last scan prior to definitive surgery is either a complete response or partial response per RECIST 1.1 criteria by BICR. The response does not require confirmation. Response rates and their corresponding 95% exact CI will be calculated by Clopper-Pearson method presented for each randomized arm.

Clinical response rate by investigator will be reported similarly.

Approved v1.0 [redacted]

Subset analyses of cRR by BICR by PDL1 status will be performed (PD-L1<1% PD-L1 ≥ 1%, PD-L1 1-49%, PD-L1 ≥ 50%, not evaluable/indeterminate) and by Tumor TMB (≥12.3 mut/MB, < 12.3 mut/MB, Overall, not evaluable).

7.6.3 Analysis of Event Free Survival

7.6.3.1 Primary Event Free Survival

One of the primary objectives of the study is to compare the event-free survival (based on BICR assessments) between treatment groups in all concurrently randomized participants in Arms B and C.

The primary definition of EFS, censoring for subsequent anticancer therapy, will be used in this analysis.

EFS will be compared between the treatment groups (concurrent B and C) at the interim and final analyses, using stratified log-rank test, with stratification factors as per IRT, two-sided p-value will also be reported. A Lan DeMets α-spending function with O'Brien and Fleming type of boundary will be employed to determine the nominal significance levels for the interim and final analyses. The stratified hazard ratio between the treatment groups will be presented along with 100*(1- α)% CI (adjusted for interim).

EFS will be estimated using the Kaplan Meier techniques and will be displayed graphically. A two-sided 95% CI for median EFS in each treatment group will be computed via the log-log transformation method. EFS rates at fixed time points (e.g. 6, 12 months, depending on the minimum follow-up) will be presented along with their associated 95% CIs. These estimates will be derived from the Kaplan Meier estimate and corresponding CIs will be derived based on Greenwood[16] formula for variance derivation and on log-log transformation applied on the survivor function[17.]

These EFS analyses will also be conducted for Arm A, including Kaplan Meier curve, median and EFS rates with 95% CI and stratified HR between Arm A and Arm B (in the All Concurrently Randomized Participants in Arms A and B population) with 95% HR.

Analyses of EFS will also be conducted based on the secondary definition of EFS (not censoring for subsequent therapies). These analyses will be the same as those specified above.

The source of EFS event (progression precluding surgery, progression, recurrence (locoregional, distant or both) or death) will be summarized by treatment group. The status of subjects who are censored (as per primary definition of EFS) in the EFS KM analysis will be tabulated for each treatment group including the following categories:

- On-study (on-neoadjuvant treatment, on-adjuvant treatment, in follow-up)
- Off-study (lost to follow-up, withdraw consent, never treated)
- No baseline tumor assessment
- No on-study tumor assessment and no death
- Received subsequent anticancer therapy

- A by-subject listing will be presented including treatment group, EFS duration under the primary definition, EFS duration on the secondary definition, whether the subject was censored under the primary definition, and if censored, the reason, and whether the subject was censored under the secondary definition, and if censored, the reason.

A by-subject listing of lesion evaluations per BICR will be presented.

7.6.3.2 Supportive Analyses of Event-Free Survival

The following sensitivity analyses will be conducted in the concurrently randomized subjects in arms B and C for the primary definition. The p-values from sensitivity analyses for efficacy endpoints, are for descriptive purpose only and not adjusted for multiplicity.

- Delayed effect of immunotherapy interventions may cause a late separation in the OS KM curves and non-proportional hazards.
  - EFS will be compared between treatment groups via a 2-sided max-combo test. The max-combo test statistic is the maximum of 4 diffrerent Fleming-Harrington family weighted log-rank test statistics. Zm = max (FH (0, 0), FH (0,1), FH (1,0), F(1,1)), where FH(ρ,γ) are the test statistics from the Fleming-Harrington family of test statistics. FH (0, 0) corresponds to the log-rank test, while FH (0, 1) is more sensitive to late-difference alternatives, FH(1,0) is more sensitive to early difference with decreasing treatment effect and FH(1,1) uses weigths at the median.
  - To examine the assumption of proportional hazards in the Cox regression model, in addition to treatment, a time-dependent variable defined by treatment by time interaction will be added into the model. A two-sided Wald Chi-square p-value of less than 0.1 may indicate a potential non constant treatment effect. In such case, the following analysis will be conducted:
    - ♦ The estimates of the EFS hazard ratios will be estimated in 2 periods. The periods will be defined by a cut off point. The cut off point will be calculated using a stratified time-dependent Cox model with effects for treatment and period-by-treatment interaction. The cut off point will be estimated using a grid of possible cut off points and obtained by maximizing the partial log likelihood. Ties will be handled using the exact method. A two-sided 95% CI for the hazard ratio's will also be presented. Visual interpretation of the curves may lead to additional analyses with several cut off points.
- A multivariate Cox regression model will be used in order to estimate the treatment effect after adjustment for possible imbalances in known or potential prognostic factors. The factors used in the randomization, will be included in the model as stratification factors. However, all additional factors will be incorporated as covariates. The additional factors, which are all measured at baseline, will include:
  - Histology (Squamous, Non-squamous)
  - Age categorization (< 65, ≥ 65)
  - ECOG (0, ≥ 1)
  - Race (White, Black, Asian, Other)
- The level of the covariate normally associated with the worst prognosis will be coded as the reference level. The hazard ratio associated with treatment and with each of the baseline covariates will be presented along with associated 95% CIs and p-value.

Approved v1.0 [redacted]

- The primary EFS based on BICR assessments analysis will be repeated using secondary EFS definition which accounts for the tumor scans post subsequent therapies for the primary efficacy population. Stratified log-rank test p-value will be generated.
- EFS based on BICR assessments, using stratification factors as obtained from the baseline CRF pages or database (instead of IRT). This analysis will be performed only if the stratification variable/factor at randomization (as per IRT) and baseline are discordant for at least 10% of randomized subjects. Stratified log-rank test p-value will be generated.
- EFS based on BICR assessments accounting for missing tumor assessment prior to EFS event (progression/recurrence or death). This analysis will be performed only if at least 10% of events have missing prior tumor assessment within the primary efficacy population. It will apply the following restriction to the primary definition: If the elapsed time between the EFS event and the last assessment immediately prior to the event is two or more missed visits, the subject's EFS will be censored at his/her last tumor assessment prior to the EFS event. Stratified log-rank test p-value will be generated.
- EFS based on BICR assessments accounting for site reported pathology results. In case of site reported pathology recurrence on the CRF at an earlier date than the BICR event date, an event will be assigned at the pathology site reported date. No p-value will be generated.
- EFS based on investigator assessments. The hazard ratio associated with treatment and median EFS will be presented along with the associated two-sided 95% CIs. Kaplan-Meier plot will be produced. It is to be noted that per CRF instruction, the investigator will not consider a second primary cancer as a recurrence/progression. While if such lesions are present on tumor assessment at the BICR, they will be considered as new lesions, since the BICR does not have access to biopsy results. EFS by investigator is defined the same way as for EFS by BICR, except that both pathology and imaging recurrences reported in the CRF are taken into account as event and censoring will occur at the time of second primary cancer. No p-value will be generated.
- EFS based on BICR assessments using an un-stratified Cox model. Un-stratified log-rank test p-value will be generated
- EFS in treated subjects from concurrently randomized arms B and C using treatment group "as treated" if more than 10% randomized subjects in any treatment group were never treated or treated differently than randomized among corresponding analysis population. Stratified log-rank test p-value will be generated.
- EFS analysis for participants with no relevant deviation. This analysis will be conducted only if there are more than 10% participants with relevant protocol deviations. Stratified log-rank test p-value will be generated.
- In order to assess the potential variability introduced by the optional adjuvant chemotherapy:
  - Baseline demographics and disease characterictics, characteristics at the time of surgery will be tabulated by adjuvant therapy status.
  - A Cox regression model with treatment and an additional time-dependant covariate as indicator of the start of adjuvant therapy will used.
  - Additional sensitivity analyses may be performed

7.6.3.3 Subset Analyses of EFS

The influence of baseline and demographic characteristics on the treatment effect will be explored via subset analyses for the factors specified in section 7.6.2.3.

A forest plot of the EFS based on BICR assessments, unstratified hazard ratios (HR) along with two-sided 95% CIs will be produced for each level of the subgroups listed in section 7.6.2.3. If subset category has less than 10 subjects per treatment group, HR will not be computed/displayed. Median and 95% CI will be provided.

In addition, for gender, baseline disease stage, histology, PD-L1 and tumor TMB subsets, Kaplan Meier Curves will be generated.

7.6.3.4 EFS Landmark Analyses by pCR and MPR Status

EFS (based on BICR assessments, primary definition) Kaplan-Meier curves will be generated by pCR and by MPR status. These analyses will be landmarked at the time of surgery and will be limited to subjects with pCR or MPR status available. Median and 95% CI will be provided. HR and 95% CI for concurrently randomized subjects in arms B and C will be provided by pCR and by MPR status.

7.6.3.5 Current Status of EFS

Time from last censoring point to cutoff date in months will be summarized by treatment group and overall for randomized subjects. Subjects who have a EFS event will be considered as current for this analysis. The secondary definition of EFS (by BICR) will be used for this summary.

7.6.4 Analysis of Overall Survival

7.6.4.1 OS Analyses

OS will be hierarchically tested if EFS is significant. Details are provided in section 5.3.

OS will be compared between the treatment groups (concurrent B and C) at the interim and final analyses, using stratified log-rank test, with stratification factors as per IRT, two-sided p-value will also be reported. A Lan DeMets α-spending function with O'Brien and Fleming type of boundary will be employed to determine the nominal significance levels for the interim and final analyses. The stratified hazard ratio between the treatment groups will be presented along with $100*(1- \alpha)\%$ CI (adjusted for interim).

OS will be estimated using the Kaplan Meier techniques and will be displayed graphically. A two-sided 95% CI for median EFS in each treatment group will be computed via the log-log transformation method. EFS rates at fixed time points (e.g. 6, 12 months, depending on the minimum follow-up) will be presented along with their associated 95% CIs. These estimates will be derived from the Kaplan Meier estimate and corresponding CIs will be derived based on Greenwood formula for variance derivation and on log-log transformation applied on the survivor function.

These analyses OS analyses will also be conducted for Arm A, including Kaplan Meier curve, median and OS rates with 95% CI and stratified HR between Arm A and Arm B (in the All Concurrently Randomized Participants in Arms A and B population) with 95% HR.

Approved v1.0 [redacted]

The number of participants who are censored in the OS KM analysis and their status will be tabulated using following categories:

- Still on treatment (No recurrence/progression, recurrence/progression)
- In follow-up
- Off study
  - lost to follow-up
  - participant withdrew consent
  - other

7.6.4.2 Supportive Analyses for OS

The following sensitivity analyses will be conducted in the concurrently randomized subjects in arms B and C. The p-values from sensitivity analyses for efficacy endpoints, if presented, are for descriptive purpose only and not adjusted for multiplicity.

- A multivariate Cox regression model will be used in order to estimate the treatment effect after adjustment for possible imbalances in known or potential prognostic factors. The factors used in the randomization, will be included in the model as stratification factors. However, all additional factors will be incorporated as covariates. The additional factors, which are all measured at baseline, will include:
  - Histology (Squamous, Non-squamous)
  - Age categorization (< 65, ≥ 65)
  - ECOG (0, ≥ 1)
  - Race (White, Black, Asian, Other)

The level of the covariate normally associated with the worst prognosis will be coded as the reference level. The hazard ratio associated with treatment and with each of the baseline covariates will be presented along with associated 95% CIs and p-value.

- OS analysis using stratification factors as obtained from the baseline CRF pages or database (instead of IRT). This analysis will be performed only if the stratification variable/factor at randomization (as per IRT) and baseline are discordant for at least 10% of randomized subjects. Stratified log-rank test p-value will be generated.
- OS analysis using an un-stratified Cox model. Unstratified log-rank test p-value will be generated.
- OS analysis for participants with no relevant deviation. This analysis will be conducted only if there are more than 10% participants with relevant protocol deviations. Stratified log-rank test p-value will be generated.
- OS in treated subjects from concurrently randomized arms B and C using treatment group "as treated" if more than 10% randomized subjects in any treatment group were never treated or treated differently than randomized among corresponding analysis population. Stratified log-rank test p-value will be generated.

- In order to assess the potential variability introduced by the optional adjuvant chemotherapy:
  - Baseline demographics and disease characteristics, characteristics at the time of surgery will be tabulated by adjuvant therapy status.
  - A Cox regression model with treatment and an additional time-dependant covariate as indicator of the start of adjuvant therapy will used.
  - Additional sensitivity analyses may be performed

7.6.4.3 Subset Analyses of OS

The influence of baseline and demographic characteristics on the treatment effect will be explored via subset analyses for the factors specified in section 7.6.2.3.

A forest plot of the OS unstratified hazard ratios (HR) along with two-sided 95% CIs will be produced for each level of the subgroups listed in section 7.6.2.3. If subset category has less than 10 subjects per treatment group, HR will not be computed/displayed. Median and 95% CI will be provided.

In addition, for gender, baseline disease stage, histology, PD-L1 and tumor TMB subsets, Kaplan Meier Curves will be generated.

7.6.4.4 OS Landmark Analyses by pCR and MPR Status

OS Kaplan-Meier curves will be generated by pCR and by MPR status. These analyses will be landmarked at the time of surgery and will be limited to subjects with pCR or MPR status available. Median and 95% CI will be provided. HR and 95% CI for concurrently randomized subjects in arms B and C will be provided by pCR and by MPR status.

7.6.4.5 Subject Follow-Up

The extent of follow-up defined as the time between randomization date and last known date alive (for subjects who are alive) or death date (for subjects who died). It will be summarized descriptively (median, min, max, etc) in months.

The currentness of follow-up for survival, defined as the time between last OS contact (i.e., last known date alive or death date) and cut-off date (defined by last patient last visit date), will be summarized in months by treatment group. Subjects who died and subjects with a Last Known Date Alive on or after data cut-off date will have a zero value for currentness of follow-up.

7.6.5 Interim Analyses of EFS and OS

An independent statistician external to BMS will perform the interim analyses. In addition to the formal planned interim analyses for EFS and OS, the Data Monitoring Committee (DMC) will have access to periodic un-blinded interim reports of efficacy and safety to allow a risk/benefit assessment. Details are included in the DMC charter.

Details of interim analyses timing and significance boundaries are provided in sections 5.2 and 5.3.

The DMC will review the safety and available efficacy data as planned in the DMC charter and will determine if the study should continue with or without changes or if accrual should be stopped. Subject enrollment will continue while waiting for the DMC's decisions.

The chair of the DMC and the sponsor can call an unscheduled review of the safety data.

At the time of the interim analyses for of EFS and OS, the DMC may recommend continuing or stopping the trial for superiority. If the trial continues beyond the formal interim analyses, the nominal critical point for the final analysis will be determined using the recalculated information fraction at the time of the interim analysis, as described above. The final hazard ratio and corresponding confidence interval will be reported whereby the confidence interval will be adjusted accordingly (i.e. using the recalculated nominal α level at the final analysis).

If the EFS is significant but not OS, the trial is successful but will continue for further OS evalution.

If the p-value crosses the boundary at the interim analysis (EFS or OS), the p-value from the interim stratified log-rank test will be considered the final analysis result.

7.6.6 Analysis of TTDM

Time to death or distant matastases is a secondary endpoint.

TTDM, based on BICR assessments, will be estimated using the Kaplan Meier techniques and will be displayed graphically. A two-sided 95% CI for median TTDM in each treatment group will be computed via the log-log transformation method. TTDM rates at fixed time points (e.g. 6, 12 months, depending on the minimum follow-up) will be presented along with their associated 95% CIs. These estimates will be derived from the Kaplan Meier estimate and corresponding CIs will be derived based on Greenwood formula for variance derivation and on log-log transformation applied on the survivor function.

7.6.7 Event Free Survival on Next Line of Therapy

Event Free survival on next line of therapy is an exploratory endpoint.

Event free survival will be estimated using the Kaplan Meier techniques and will be displayed graphically. A two-sided 95% CI for median in each treatment group will be computed via the log-log transformation method. Events rates at fixed time points (e.g. 6, 12 months, depending on the minimum follow-up) will be presented along with their associated 95% CIs. These estimates will be derived from the Kaplan Meier estimate and corresponding CIs will be derived based on Greenwood formula for variance derivation and on log-log transformation applied on the survivor function.

7.6.8 Consistency of Efficacy by Randomization Period

Consistency of population amongs the different randomization periods will be examined by summarizing the primary endpoints by treatment arm as randomized in the the different randomization period: before revised protocol 02 (1:1 A vs B), under revised protocol 02 (1:1:1 A vs B vs C) and after revised protocol 03 (1:1 B vs C). Descriptive statistics will be provided including pCR rates, EFS medians, 95% CI and Kaplan-Meier plots. HR and 95% CI will be

provided for arms C vs B during the 1:1:1 randomization (under revised protocol 02) and during the 1:1 randomization (under revised protocol 03).

Concurrent randomization is considered at the site level basis, when the site switched to the revised protcol. In practice, this includes subjects randomized on the randomization lists from the 1:1:1 randomization (revised protocol 02) and the subsequent 1:1 randomization between B and C only (revised protocol 03).

7.7 Safety

Analyses in this section will be tabulated for all treated subjects by treatment group as treated, unless otherwise specified.

Analyses will be performed on the treated subjects from arm A and from the concurrently randomized arms B and C. Participants in Arm B randomized in the initial protocol will only be reported in listings.

7.7.1 Definitive Surgery Related Safety

Incidence of AE/SAE indicated as surgical complication in the CRF, up to 90 days after surgery will be summarized by worst CTC grade, by treatment group.

Adverse events leading to cancellation of surgery and leading to surgery delay will be summarized by worst CTC grade, by treatment group.

7.7.2 Deaths

Deaths will be summarized by treatment group:

- All deaths, reasons for death.
- Deaths within 30 days of last neoadjuvant dose received (30-day safety window), reasons for death.
- Deaths within 100 days of last neoadjuvant dose received (100-day safety window), reasons for death.
- For subjects with adjuvant therapy: deaths from start of adjuvant to last adjuvant dose received + 30 days

A by-subject listing of deaths will be provided for the all enrolled subjects population.

7.7.3 Serious Adverse Events

Serious adverse events will be summarized by treatment group:

- Overall summary of SAEs by worst CTC grade (any grade, grade 3-4, grade 5) presented by SOC/PT.
- Overall summary of drug-related SAEs by worst CTC grade (any grade, grade 3-4, grade 5) presented by SOC/PT.
- For subjects with adjuvant treatment: Overall summary of SAEs by worst CTC grade (any grade, grade 3-4, grade 5) presented by SOC/PT from start of adjuvant to last adjuvant dose received + 30 days

- All analyses will be conducted using the 30-day safety window.

A by-subject SAE listing will be provided for the "enrolled subjects" population.

7.7.4 Adverse Events Leading to Discontinuation of Study Therapy

AEs are indicated as leading to discontinuation, when they lead to discontinuation of at least one agent of the regimen. Reporting is done based on AE CRF form.

AEs leading to discontinuation (of neoadjuvant treatment) will be summarized by treatment group:

- Overall summary of AEs leading to discontinuation by worst CTC grade (any grade, grade 3-4, grade 5) presented by SOC/PT.
- Overall summary of drug-related AEs leading to discontinuation by worst CTC grade (any grade, grade 3-4, grade 5) presented by SOC/PT.
- For subjects with adjuvant treatment: Overall summary of AEs leading to discontinuation by worst CTC grade (any grade, grade 3-4, grade 5) presented by SOC/PT from start of adjuvant to last adjuvant dose received + 30 days

The analyses will be conducted using the 30-day safety window.

A by-subject AEs leading to discontinuation listing will be provided.

7.7.5 Adverse Events Leading to Dose Modification

AEs leading to dose delay/reduction will be summarized by treatment group:

- Overall summary of AEs leading to dose delay/reduction by worst CTC grade (any grade, grade 3-4, grade 5) presented by SOC/PT.
- Overall summary of related AEs leading to dose delay/reduction by worst CTC grade (any grade, grade 3-4, grade 5) presented by SOC/PT.

The analysis will be conducted using the 30-day safety window.

A by-subject AEs leading to dose delay/reduction listing will be provided.

7.7.6 Adverse Events

Adverse events will be summarized by treatment group.

The following analyses will be conducted using the 30 days safety window only:

- Overall summary of any AEs by worst CTC grade (1, 2, 3, 4, 5, not reported, total) presented by SOC/PT.
- Overall summary of any AEs presented by worst CTC grade (any grade, grade 3-4, grade 5) by SOC/PT. This table will be restricted to events with an incidence greater or equal to 5% in any treatment group.
- for subjects with adjuvant: Overall summary of any AEs presented by worst CTC grade (any grade, grade 3-4, grade 5) by SOC/PT, from start of adjuvant to last adjuvant dose received + 30 days
- Overall summary of any non-serious AEs presented by SOC/PT. This table will be restricted to events with an incidence greater or equal to 5% in any treatment group.

- Overall summary of any AEs that required immune modulating medication by worst CTC grade (any grade, grade 3-4, grade 5) presented by SOC/PT.
- Overall summary of drug-related AEs by worst CTC grade (1, 2, 3, 4, 5, not reported, total) presented by SOC/PT.
- For subjects with adjuvant treatment: Overall summary of Drug-Related AEs by worst CTC grade (any grade, grade 3-4, grade 5) presented by SOC/PT from start of adjuvant to last adjuvant dose received + 30 days

The following analyses will be conducted using the 30 days safety window and repeated using the 100 days safety window:

- Overall summary of drug-related AEs by worst CTC grade (any grade, grade 3-4, grade 5) presented by SOC/PT

A by-subject AE listing will be provided. A by-subject listing of any AE requiring immune modulating medications will also be provided.

7.7.7 Select Adverse Events

Unless otherwise specified, analyses will be performed by select AE category. Analyses will also be repeated by subcategory of endocrine events.

7.7.7.1 Incidence of Select AE

Select AEs will be summarized by treatment group for each category/subcategory.

The following analyses will be conducted using the 30-day safety window only:

- Overall summaries of any select AEs by worst CTC grade (any grade, grade 3-4, grade 5) presented by Category or Subcategory/PT.
- Overall summaries of any drug-related select AEs by worst CTC grade (any grade, grade 3-4, grade 5) presented by Category or Subcategory/PT.
- Overall summaries of any serious select AEs by worst CTC grade (any grade, grade 3-4, grade 5) presented by Category or Subcategory /PT.
- Overall summaries of drug-related serious select AEs by worst CTC grade (any grade, grade 3-4, grade 5) presented by Category or Subcategory /PT.
- Overall summaries of any select AEs leading to discontinuation by worst CTC grade (any grade, grade 3-4, grade 5) presented by Category or Subcategory /PT.
- Overall summaries of drug-related select AEs leading to discontinuation by worst CTC grade (any grade, grade 3-4, grade 5) presented by Category or Subcategory /PT.
- Summary of frequency of unique select AEs by Category.

A by-subject select AE listing will be provided.

7.7.7.2 Time-to Onset of Select AE

Time-to onset of drug-related select AEs (any grade, grade 3-5) will be summarized for each category/subcategory by treatment group.

Time-to onset analyses are restricted to treated subjects who experienced at least one drug-related select AE in the category/subcategory. The analyses will be conducted using the 30-day safety window.

Additional details regarding the time-to onset definition are described in time-to onset definition subsection of APPENDIX 1.

7.7.7.3 Time-to Resolution of Select AE

Time-to resolution of the following specific events will be summarized separately for each category/subcategory.

- Time-to resolution of drug-related select AE (any grade, grade 3-5) by treatment group
- Time-to resolution of drug-related select AE (any grade, grade 3-5) where immune modulating medication was initiated, by treatment group

Time-to resolution analyses are restricted to treated subjects who experienced the specific events. Time-to resolution where immune modulating medication was initiated analyses are restricted to treated subjects who experienced the specific events and who received immune modulating medication during the longest select AE.

The analyses will be conducted using the 30-day safety window.

The following summary statistics will be reported: percentage of subjects with resolution of the longest select AE, median time-to resolution along with 95% CI (derived from Kaplan-Meier estimation) and ranges.

See time-to resolution definition subsection of APPENDIX 1 for additional details.

7.7.8 Immune-Mediated Adverse Events

IMAEs will be summarized by treatment group for each immune-mediated category / PT using the 100-day safety window:

- Overall summary of non-endocrine IMAEs by worst CTC grade (any grade, grade 3-4, grade 5) where immune modulating medication was initiated presented by Category / PT.
- Overall summary of endocrine IMAEs by worst CTC grade (any grade, grade 3-4, grade 5) presented by Category / PT.
- Overall summary of non-endocrine IMAEs leading to discontinuation by worst CTC grade (any grade, grade 3-4, grade 5) where immune modulating medication was initiated presented by Category / PT.
- Overall summary of endocrine IMAEs leading to discontinuation by worst CTC grade (any grade, grade 3-4, grade 5) presented by Category / PT.
- Overall summary of non-endocrine IMAEs leading to dose delay or reduction by worst CTC grade (any grade, grade 3-4, grade 5) where immune modulating medication was initiated presented by Category / PT
- Overall summary of endocrine IMAEs leading to dose delay or reduction by worst CTC grade (any grade, grade 3-4, grade 5) presented by Category / PT.

- Summaries of time-to onset and time-to resolution of non-endocrine IMAEs where immune modulating medication was initiated presented by Category.
- Summaries of time-to onset and time-to resolution of endocrine IMAEs presented by Category.

A by-subject listing of IMAEs will be provided. By-subject listings of time-to resolution for longest IMAEs cluster (any grade and grade 3-5 in separate summaries) will also be provided. For new studies which collect investigator assessment of potential IMAE data, a by-subject listing of AEs considered as immune-mediated events per investigator but not qualified for IMAEs definition will also be provided.

7.7.9 Other Events of Special Interest

OEOSI will be summarized by treatment group for each category.

The following analyses will be conducted using the 100-day safety window:

- Overall summary of OEOSI by worst CTC grade (any grade, grade 3-4, grade 5) presented by Category / PT
- Overall summary of drug-related OEOSI by worst CTC grade (any grade, grade 3-4, grade 5) presented by Category / PT

A by-subject listing of OEOSI will be provided.

7.7.10 Multiple Events

The following summary tables will be provided:

- A table showing the total number and rate (exposure adjusted) of occurrences for all AEs.
- A table showing the total number and rate (exposure adjusted) of occurrences for AEs occurring in at least 5% of subjects in any treatment group.

Exposure adjustement will be based on the exposure in neoadjuvant treatment only.s

A listing displaying the unique instances of all AEs, i.e., after duplicates have been eliminated and overlapping and contiguous occurrences of the same event (i.e. same PT) have been collapsed will be provided. No formal comparisons will be made between treatment groups.

7.7.11 New Primary Cancers

Occurence of new primary cancer reported on the new primary cancer CRF form will be listed based on the all treated population.

7.7.12 Laboratory Parameters

The analysis population for each laboratory test is restricted to treated subjects who underwent that laboratory test.

A by-subject listing of differences in categorization of SI and US laboratory test results will be provided.

7.7.12.1 Hematology

The following will be summarized by treatment group as worst CTC grade on-treatment per subject and as shift table of worst on-treatment CTC grade compared to baseline CTC grade per subject: hemoglobin (HB), platelets, white blood counts (WBC), absolute neutrophils count (ANC) and lymphocyte count (LYMPH).

The analyses will be conducted using the 30-day safety window.

A by-subject listing of these laboratory parameters will be provided.

7.7.12.2 Serum Chemistry

The following will be summarized by treatment group as worst CTC grade on-treatment per subject and as shift table of worst on-treatment CTC grade compared to baseline CTC grade per subject: ALT, AST, alkaline phosphatase (ALP), total bilirubin, creatinine, amylase, lipase.

The analyses will be conducted using the 30-day safety window.

A by-subject listing of these laboratory parameters will be provided.

7.7.12.3 Electrolytes

The following will be summarized by treatment group as worst CTC grade on-treatment per subject and as shift table of worst on-treatment CTC grade compared to baseline CTC grade per subject: sodium (high and low), potassium (high and low), calcium (high and low), magnesium (high and low), and Glucose Serum (fasting hyperglycemia and hypoglycemia regardless of fasting status).

The analyses will be conducted using the 30-day safety window.

A by-subject listing of these laboratory parameters will be provided.

7.7.12.4 Additional Analyses

In addition, further analyses on specific laboratory parameters will be performed by treatment group:

Abnormal Hepatic Function Test

The number of subjects with the following laboratory abnormalities from on-treatment evaluations will be summarized by treatment group:

- ALT or AST > 3 x ULN, > 5 x ULN, > 10 x ULN and > 20 x ULN
- Total bilirubin > 2 x ULN
- ALP > 1.5 x ULN
- Concurrent (within 1 day) ALT or AST > 3 x ULN and total bilirubin > 1.5 x ULN
- Concurrent (within 30 days) ALT or AST > 3 x ULN and total bilirubin > 1.5 x ULN
- Concurrent (within 1 day) ALT or AST > 3 x ULN and total bilirubin > 2 x ULN
- Concurrent (within 30 days) ALT or AST > 3 x ULN and total bilirubin > 2 x ULN

The analyses will be conducted using the 30-day safety window.

A by-subject listing of these specific abnormalities will be provided.

Abnormal Thyroid Function Test

The number of subjects with the following laboratory abnormalities from on-treatment evaluations will be summarized by treatment group:

- TSH value > ULN and
  - with baseline TSH value ≤ ULN
  - with at least one FT3/FT4 test value < LLN within 2-week window after the abnormal TSH test
  - with all FT3/FT4 test values ≥ LLN within 2-week window after the abnormal TSH test
  - with FT3/FT4 missing within 2-week window after the abnormal TSH test.

- TSH < LLN and
  - with baseline TSH value ≥ LLN
  - with at least one FT3/FT4 test value > ULN within 2-week window after the abnormal TSH test
  - with all FT3/FT4 test values ≤ ULN within 2-week window after the abnormal TSH test
  - with FT3/FT4 missing within 2-week window after the abnormal TSH test

The analyses will be conducted using the 30-day safety window.

A by-subject listing of these specific abnormalities will be provided.

7.7.13 Vital Signs and Pulse Oximetry

Vital signs and pulse oximetry (i.e. % oxygen saturation) collected on the CRF will be provided in separate listings.

7.7.14 Physical Measurements

Physical measurements will be listed by subject.

7.7.15 Non-Protocol Medical Procedures

Non-protocol medical procedures will be listed by subject.

7.7.16 Immunogenicity Analysis

Further details on immunogenicity background and rationale, definitions, population for analyses and endpoints are described in APPENDIX 3.

Incidence of ADA

For nivolumab and ipilimumab: Number (%) of subjects will be reported for the following parameters based on Evaluable Subjects.

- Baseline ADA-positive
- ADA-positive

- – Last Sample Positive
- – Other positive
- – Neutralizing Positive

- ADA-negative

Note that persisitent positive is not applicable for this study given the only 3 sampling timepoints during neoadjuvant treatment.

A listing of all ADA assessments will be provided.

A spider plot of nivolumab and ipilimumab ADA test result (titers) over time may be provided for nivolumab and ipilimumab ADA positive subjects.

ADA Titer Kinetics

All ADA-positive subjects will be included in the analysis.

Summary statistics of subject-level ADA titers using the maximum titer value within an ADA-positive subject will be presented for baseline ADA-negative subjects and baseline ADA-positive subjects, respectively. The median, interquartile range, and range of the maximum titers will be reported. For ADA-positive subjects with baseline ADA-positive sample, the median and interquartile range of the fold increase from baseline in titer (ratio of maximum post-baseline titer to baseline titer) will also be reported. Graphical presentation of the summary may be provided using boxplots, as appropriate.

Clinical implications

Clinical implications of nivolumab and ipilimumab immunogenicity will be primarily focused on subjects with persistent ADA-positive relative to ADA-negative. Subjects with any ADA-positive samples after initiation of treatment (relative to baseline) may be used to explore clinical implications. Effect of immunogenicity on clearance of nivolumab and ipilimumab will be explored by comparison of clearance estimates (determined by PPK analysis).

Effect of ADA response on safety will be explored by examining the frequency and type of AEs of interest such as select AEs of hypersensitivity/infusion reaction. For each category of AEs of interest, summary tables for incidence will be provided for each of the preferred terms and overall within a category by ADA status, if the number of ADA-positive subjects is of sufficient size (e.g., at least ≈10 subjects). Otherwise, individual subject's safety profile will be examined and described based on a listing.

Pathological Complete response rate and Major pathological complete response will be presented by ADA status.

7.7.17 Pregnancy

A by-subject listing of pregnancy tests results will be provided for randomized female subjects.

7.7.18 Adverse Events By Subgroup

Overall summary of any AEs and drug-related AEs by worst CTC grade (any grade, grade 3-4, grade 5) presented by SOC/PT and for each treatment group for the following subgroups:

- Sex (Male vs. Female)
- Race
- Age (< 65 vs. 65 - < 75 vs. 75 - < 85 vs. ≥ 85 vs. ≥ 75 vs. ≥ 65)
- Region (North America, Europe, Asia, Rest of World)
- Type of platinum therapy (cisplatin, carboplatin, subjects switching from cisplatin to carboplatin).
- Type of chemotherapy regimen in arm B (available in arm C (Gemcitabine-Cisplatin, Pemetrexed-Cisplatin, Paclitaxel-Carboplatin, not available in arm C (Vinorelbine-Cisplatin, Docetaxel-Cisplatin)) , based on first neoadjuvant cycle.

These analyses will be conducted using the 30-day safety window only.

7.7.19 Consistency of Safety by Randomization Period

Consistency of population amongs the different randomization periods will be examined by summarizing key safety by treatment arm as randomized in the the different randomization period: before revised protocol 02 (1:1 A vs B), under revised protocol 02 (1:1:1 A vs B vs C) and after revised protocol 03 (1:1 B vs C).

- Overall summary of any AEs presented by worst CTC grade (any grade, grade 3-4, grade 5) by SOC/PT with 30-day safety window.
- Overall summary of AEs leading to discontinuation (of neoadjuvant treatment) by worst CTC grade(any grade, grade 3-4, grade 5) presented by SOC/PT with 30-day safety window.
- Overall summary of non-endocrine IMAEs by worst CTC grade (any grade, grade 3-4, grade 5) where immune modulating medication was initiated presented by Category / PT with 100-day safety window.
- Overall summary of endocrine IMAEs by worst CTC grade (any grade, grade 3-4, grade 5) presented by Category / PT with 100-day safety window.
- Concurrent randomization is considered at the site level basis, when the site switched to the revised protcol. In practice, this includes subjects randomized on the randomization lists from the 1:1:1 randomization (revised protocol 02) and the subsequent 1:1 randomization between B and C only (revised protocol 03).

7.8 Pharmacokinetics

The nivolumab and ipilimumab concentration data obtained in this study will be combined with data from other studies in the clinical development program to develop a population PK model. This model will be used to evaluate the effects of intrinsic and extrinsic covariates on the PK of nivolumab and ipilimumab. In addition, exposure-response analyses with selected efficacy and safety endpoints will be conducted. Results of population PK and exposure response-analyses will be reported separately.

7.9 Biomarkers

Analyses for PD-L1 and TMB (tumor tissue) are described below.

Methodology for biomarkers other than PD-L1 and Tumor TMB will be reported separately.

7.9.1 PD-L1 Expression

Descriptive statistics of PD-L1 expression:

- Listing of all PD-L1 IHC data, all randomized subjects.
- Summary of tumor specimen acquisition and characteristics, all randomized subjects.
- Summary statistics of PD-L1 expression in all randomized subjects with quantifiable PD-L1 expression.
- Cumulative distribution plot of baseline PD-L1 expression versus population percentile in all randomized subjects with quantifiable PD-L1 expression.
- Box plots of PD-L1 expression versus pCR in all randomized subjects with quantifiable PD-L1 expression.
- Waterfall plot of Individual PD-L1 expression in all randomized subjects with quantifiable PD-L1 expression.
- Subgroups analyses of efficacy (pCR, MPR, EFS, OS) by PD-L1 status (PD-L1<1% PD-L1 ≥ 1%, PD-L1 1-49%, PD-L1 ≥ 50%, not evaluable/indeterminate) are described in Section 7.6.
- In addition the following analyses will be conducted in the concurrently randomized subjects in arms B and C.
- An exploratory Cox proportional hazards model, in order to assess the association of EFS (based on BICR assessments) with PD-L1, will be fitted for EFS with PD-L1, treatment arm and PD-L1* treatment arm interaction, among all PD-L1 evaluable subjects. An appropriate transformation of PD-L1 expression may be considered depending on an assessment of fit of the model. The following summaries will be presented by treatment arm
  - A plot of estimated $\log_e$(hazard ratio) with 95% confidence band vs PD-L1 expression(X-axis)
- An exploratory Cox proportional hazards model, in order to assess the association of OS with PD-L1, will be fitted for OS with PD-L1, treatment arm and PD-L1* treatment arm interaction, among all PD-L1 evaluable subjects. An appropriate transformation of PD-L1 expression may be considered depending on an assessment of fit of the model. The following summaries will be presented by treatment arm
  - A plot of estimated $\log_e$(hazard ratio) with 95% confidence band vs PD-L1 expression (X-axis).
- A logistic regression model will be fitted for pCR with PD-L1, treatment arm and PD-L1* treatment arm interaction, among all PD-L1 evaluable subjects. The following summaries will be reported:
  - A plot of estimated odds ratio with 95% confidence band vs PD-L1 expression (X-axis)

7.9.2 Tumor Mutational Burden (TMB)

The analyses are based on tumor tissue TMB evaluable subjects, defined as subjects with tissue TMB data available. It is known that not all subjects will provide tumor tissue TMB data, due to factors such as available remaining tissue and inherent failure rates of the TMB process.

The descriptive analyses of tumor tissue TMB at baseline will be conducted:

- Listing of all tumor tissue TMB data.
- Summary of tumor specimen characteristics
- Cumulative distribution plot of TMB at baseline versus population percentile in all subjects with evaluable tumor tissue TMB.

In addition, the joint distribution of PD-L1 and tumor tissue TMB among both PD-L1 and tumor tissue TMB-evaluable subjects will be examined.

Subgroups analyses of efficacy (pCR, MPR, EFS, OS) by TMB status (≥12.3 mut/MB, < 12.3 mut/MB, TMB evaluable, TMB not evaluable) are described in Section 7.6.

- In addition the following analyses will be conducted in the concurrently randomized subjects in arms B and C.
- An exploratory Cox proportional hazards model, in order to assess the association of EFS (based on BICR assessments) with TMB, will be fitted for EFS with TMB, treatment arm and TMB* treatment arm interaction, among all TMB evaluable subjects. An appropriate transformation of TMB may be considered depending on an assessment of fit of the model. The following summaries will be presented by treatment arm
  - A plot of estimated $\log_e$(hazard ratio) with 95% confidence band vs PD-L1 expression(X-axis)
- An exploratory Cox proportional hazards model, in order to assess the association of OS with TMB, will be fitted for OS with TMB, treatment arm and TMB* treatment arm interaction, among all TMB evaluable subjects. An appropriate transformation of TMB may be considered depending on an assessment of fit of the model. The following summaries will be presented by treatment arm
  - A plot of estimated $\log_e$(hazard ratio) with 95% confidence band vs TMB (X-axis).
- A logistic regression model will be fitted for pCR with TMB, treatment arm and TMB* treatment arm interaction, among all TMB evaluable subjects. The following summaries will be reported:
  - A plot of estimated odds ratio with 95% confidence band vs TMB (X-axis)

7.10 Outcomes Research Analyses

The analysis of EQ-5D-3L will be restricted to randomized subjects who have an assessment at baseline and at least one post-baseline assessment.

The following descriptive analyses will be conducted:

- EQ-5D-3L questionnaire completion rate, defined as the proportion of questionnaires actually received out of the expected number (i.e. number of subjects on treatment or in follow up), will be calculated and summarized for each assessment time point by treatment group.
- A by-subject listing of the level of problems in each dimension, corresponding to EQ-5D-3L health state (i.e., 5-digit vector), EQ-5D-3L utility index score, and EQ-5D-3L VAS score will be provided.

- Proportion of subjects reporting problems for the 5 EQ-5D-3L dimensions at each assessment time point will be summarized by level of problem and by treatment group. Percentages will be based on number of subjects assessed at assessment time point.
- For the EQ-5D-3L utility index and VAS scores, separately:
  - Mean score and mean change from baseline at each assessment time point will be summarized by treatment group using descriptive statistics (N, mean with SD and 95% CI, median, first and third quartiles, minimum, maximum).
  - A line graph summarizing the mean changes from baseline will be produced.

8 CONVENTIONS

The following conventions may be used for imputing partial dates for analyses requiring dates:

- For missing and partial adverse event onset dates, imputation will be performed using the Adverse Event Domain Requirements Specification[18]
- For missing and partial adverse event resolution dates, imputation will be performed as follows:
  - If only the day of the month is missing, the last day of the month will be used to replace the missing day. If the imputed date is after the death date or the last known alive date, then the latest known alive date or death date is considered as the resolution date.
  - If the day and month are missing or a date is completely missing, it will be considered as missing.
- Missing and partial non-study medication domain dates will be imputed using the derivation algorithm described in 4.1.3 of BMS Non-Study Medication Domain Requirements Specification[19].
- Missing and partial radiotherapy and surgery dates will be imputed using algorithm described in APPENDIX 2.
- Missing of partial definitive surgery date
  - If only the day of the month is missing, the 1st of the month will be used to replace the missing day. In case of the date of death is present and complete, the imputed definitive surgery date will be compared to the date of death. The minimum of the imputed definitive surgery date and date of death will be considered as the date of definitive surgery.
  - If the day and month are missing or a date is completely missing, it will be considered as missing.
- For death dates, the following conventions will be used for imputing partial dates:
  - If only the day of the month is missing, the 1st of the month will be used to replace the missing day. The imputed date will be compared to the last known alive date and the maximum will be considered as the death date.
  - If the month or the year is missing, the death date will be imputed as the last known alive date.

Approved v1.0 [redacted]

- If the date is completely missing but the reason for death is present, the death date will be imputed as the last known date alive.

- For date of progression/recurrence after start of study therapy, the following conventions will be used for imputing partial dates:
  - If only the day of the month is missing, the 1st of the month will be used to replace the missing day. In case of the date of death is present and complete, the imputed progression/recurrence date will be compared to the date of death. The minimum of the imputed progression/recurrence date and date of death will be considered as the date of progression/recurrence.
  - If the day and month are missing or a date is completely missing, it will be considered as missing.
- For date of progression to prior therapies, the following conventions will be used for imputing partial dates:
  - If only the day of the month is missing, the 1st of the month will be used to replace the missing day.
  - If the day and month are missing or a date is completely missing, it will be considered as missing.
- For other partial/missing dates, the following conventions were used:
  - If only the day of the month is missing, the 15th of the month will be used to replace the missing day.
  - If both the day and the month are missing, "July 1" will be used to replace the missing information.
  - If a date is completely missing, it will be considered as missing.

The following conversion factors will be used to convert days to months or years:

1 month = 30.4375 days and 1 year = 365.25 days.

Duration (e.g. time-to onset, time-to resolution) will be calculated as follows:

Duration = (Last date - first date + 1)

Last known alive date will be defined based on all appropriate dates collected on the CRF.

All statistical analyses will be carried out using SAS (Statistical Analysis System software, SAS Institute, North Carolina, USA) unless otherwise noted.

9 CONTENT OF REPORTS

All analyses described in this SAP will be included in the Clinical Study Report(s) except where otherwise noted. Additional exploratory analyses may be performed. Refer to the Data Presentation Plan for mock-ups of all tables and listings.

10 DOCUMENT HISTORY

Table 10-1: Document History

Version Number	Author(s)	Description
1.0	[redacted]	Initial version dated 17-Apr-2020

11 PREVIOUS ANALYSES

The following DMC meetings occured before approval of this SAP. Analyses were generated for these meetings according to the DMC charter.

- 1st interim safety review for Arms A and B after approximately 15 subjects in each arm completed surgery (14-Mar-2018)
- 2nd interim safety review after approximately 15 subjects in arm C completed surgery (03-Oct-2018)
- Additional safety reviews approximately every 6 months until the primary endpoint of pathological complete response is analyzed (24-May-2019, 04-Dec-2019)

No by treatment data have been share with BMS.

No other analyses have been performed prior to approval of this SAP.

APPENDIX 1 TIME-TO ONSET AND TIME-TO RESOLUTION DEFINITION AND CONVENTIONS FOR SELECT ADVERSE EVENTS, IMMUNE-MEDIATED ADVERSE EVENTS AND EVENTS OF SPECIAL INTEREST

Time-to onset definition

Time-to onset of AE (any grade) for a specific category is defined as the time between the day of the first dose of study treatment and the onset date of the earliest AE (of any grade) in this category.

The time-to onset of AE (grade 3-5) for a specific category is defined similarly with an onset date corresponding to a grade 3-5 AE.

Time-to onset of drug-related AE (any grade or grade 3-5) for a specific category is defined similarly but restricted to drug-related AE.

Time-to onset for a specific subcategory is defined similarly but restricted to event of this subcategory.

Time-to resolution definition

In order to derive the time-to resolution, overlapping or contiguous AEs within a specific category or subcategory will be collapsed into what will be termed "clustered" AEs. For example, if a subject (without pre-treatment AE) experienced an AE from 1st to 5th January, another AE (with different PT but within same category) from 6th to 11th January and same AE from 10th to 12th January, these will be collapsed into one clustered AE from 1st to 12th January. Table 11-1 is summarizing key derivation steps for each type of clustered AEs.

Time-to resolution of AE (any grade) for a specific category is defined as the longest time from onset to complete resolution or improvement to the grade at baseline among all clustered AEs experienced by the subject in this category per adverse event criteria category. Events which worsened into grade 5 events (death) or have a resolution date equal to the date of death are considered unresolved. If a clustered AE is considered as unresolved, the resolution date will be censored to the last known alive date. Improvement to the grade at baseline implies that all different events in the clustered adverse event should at least have improved to the corresponding (i.e. with same preferred term) baseline grade. This measure is defined only for subjects who experienced at least one AE in the specific category.

The time-to resolution of AE (grade 3-5) for a specific category is defined similarly with an onset date corresponding to a grade 3-5 AE.

Time-to resolution of drug-related AE (any grade or grade 3-5) for a specific category is defined similarly but restricted to drug-related AE.

The time-to resolution of AE (any grade or grade 3-5, drug-related or all) where immune modulating medication was initiated is defined similarly. For data presentation not restricted to IMAE, the additional condition that the subject started an immune modulating medication during the longest AE resolution period will be applied.

Time-to resolution for a specific subcategory is defined similarly but restricted to event of this subcategory.

Table 11-1: Derivation of Clustered AE

Type of clustered AE	Derivation
Any grade	Collapse any on-treatment AE from the same category
Drug-related of any grade	Collapse any on-treatment drug-related AE from the same category
Grade 3-5	Collapse any on-treatment AE from the same category. Resolution will be based on the onset date of the earliest grade 3-5 records (if no grade 3-5 record, clustered AE is excluded)
Drug-related of Grade 3-5	Collapse any on-treatment drug-related AE from the same category Resolution will be based on the onset date of the earliest grade 3-5 record (if no Grade 3-5 record, clustered AE is excluded)

The algorithm for collapsing adverse event records is using the following conventions:

For each subject and specified category, the corresponding adverse event records will be collapsed when:

3) Multiple adverse event records have the same onset date.
4) The onset date of an event record is either the same day or 1 day later than the resolution date of a preceding event record (contiguous events).
5) The onset date of an event record is after the onset date and prior to or on the resolution date of a preceding event record (overlapping events).

APPENDIX 2 MISSING AND PARTIAL RADIOTHERAPY AND SURGERY DATES IMPUTATION ALGORITHMS

Procedures – Imputation Rules.

If reported procedure start date is a full valid date then set start date equal to the date part of procedure start date.

In case of partial date use imputation rules described below:

- If only day is missing then
  - If month and year of procedure match month and year of first dose date then impute as date of first dose;
  - If month and year of procedure don't match month and year of first dose date then impute as first day of that month and year.
- If both day and month are missing, then impute as maximum between 01JAN of the year and date of the first dose;
- If date is completely missing or invalid then leave missing.

Note: Imputation is not applicable to data where start date is not collected (for example "PRIOR RADIOTHERAPY" CRF). Set start date to missing in this case.

If reported end date is a full valid date then set end date equal to the date part of the reported end date.

In case of partial date use imputation rules described below:

- If reported end date is partial then set end date equal to the last possible reported end date based on the partial entered reported end date.
- If reported end date is missing, continuing, unknown or invalid then set end date equal to the most recent database extraction date.

If end date was imputed then compare end date to the death date or last known alive date if subject is not dead. If posterior then end date should be imputed to death date (or last known alive date if subject not dead).

Note: Imputation of partial dates only applies to data entered on "RADIOTHERAPY” CRF page. For other CRF pages in case of partial dates set end date to missing.

Surgeries – Imputation Rules.

If reported surgery date is a full valid date then set start date equal to the date part of surgery date.

In case of partial date, use one of the two imputation rules described below:

A. For data collected on "PRIOR SURGERY RELATED TO CANCER" CRF page:

- If only day is missing then impute as the first day of the month;
- If both day and month are missing then then impute as 01JAN of the year;
- If date is completely missing or invalid then leave missing.

B. For data collected on "SUBSEQUENT SURGERY" CRF page:

- If only day is missing then
  - If month and year of surgery match month and year of first dose date then impute the missing date as the date of first dose;
  - If month and year of surgery don't match month and year of first dose date then impute as first day of that month and year;
- If both day and month are missing then impute as maximum between 01JAN of the year and date of the first dose;
- If date is completely missing or invalid then leave missing.

C. For DEFINITIVE SURGERY CRF page :

- If only day is missing then
  - if month and year of surgery match month and year of first dose date then impute the missing date as the date of first dose;
  - if month and year of surgery match month and year of last neoadjuvant dose date then impute the missing date as the date of last Neoadjuvant dose
  - if month and year of surgery don't match month and year of first dose or last Neoadjuvant dose date then impute as first day of that month and year;
- If both day and month are missing then impute as maximum between 01JAN of the year and date of last Neoadjuvant dose date;
- If date is completely missing or invalid then leave missing.

- For incomplete definitive surgery end date: set to definitive surgery start date

APPENDIX 3 IMMUNOGENICITY ANALYSIS: BACKGROUND AND RATIONALE

The following summary is from the FDA Guidance for Industry Immunogenicity Assessment for Therapeutic Protein Products and White Paper on Assessment and Reporting of the Clinical Immunogenicity of Therapeutic Proteins and Peptides – Harmonized Terminology and Tactical Recommendations by Shankar et al. The program-level definitions of sample- and subject-level ADA status are based on recommendation from the BMS Immunogenicity Council.

Immune responses to therapeutic protein products may pose problems for both patient safety and product efficacy. Immunologically based adverse events, such as anaphylaxis and infusion reactions, have caused termination of the development of therapeutic protein products or limited the use of otherwise effective therapies. Unwanted immune responses to therapeutic proteins may also neutralize the biological activity of therapeutic proteins and may result in adverse events not only by inhibiting the efficacy of the therapeutic protein product, but by cross-reacting to an endogenous protein counterpart, if present. Because most of the adverse effects resulting from elicitation of an immune response to a therapeutic protein product appear to be mediated by humoral mechanisms, circulating antibody has been the chief criterion for defining an immune response to this class of products.

ADA is defined as biologic drug-reactive antibody, including pre-existing host antibodies that are cross-reactive with the administered biologic drug (baseline ADA). Titer is a quasiquantitative expression of the level of ADA in a sample. By employing a serial dilution-based test method, titer is defined as the reciprocal of the highest dilution of the sample (e.g., dilution of 1/100 = titer of 100). The ADA is also tested, via a cell-based biologic assay or a non cell-based competitive ligand-binding assay for a subpopulation of ADA known as neutralizing antibodies (NAb), which inhibits or reduces the pharmacological activity of the biologic drug molecule regardless of its in vivo clinical relevance. Non-neutralizing ADA (non-NAb) is ADA that binds to the biologic drug molecule but does not inhibits its pharmacological activity.

ADA should be tested using sensitive and valid methods and employing an appropriate strategy for elucidating immunogenicity. Detection of ADA is typically performed in three tiers (screening, confirmatory, and titer) using statistically determined cutpoints and samples testing positive in the ADA assay are analyzed for neutralizing activity, especially in late-stage clinical studies. "Detection" of ADA implies that drug-specific ADA was confirmed. The 'drug tolerance" of an assay (highest drug concentration that does not interfere in the ADA detection method) is not an absolute value and differs between individuals due to the varying avidities of ADA immune responses. An ADA sampling strategy of collecting samples at times when the least drug concentration is anticipated (trough concentrations) can increase the likelihood of accurate ADA detection.

It is useful to present ADA results from clinical studies as (a) characteristics of the ADA immune response, (b) relationship of ADA with pharmacokinetics (PK) and, when relevant, pharmacodynamics (PD) biomarkers, and (c) relationship of ADA with clinical safety and efficacy.

Clinical consequences of ADA can range from no apparent clinical effect to lack of efficacy (primary treatment failure), loss of efficacy (secondary treatment failure) or heightened effect due to altered exposure to the biologic drug, adverse drug reactions (administration-related systemic or site reactions), and severe adverse drug reactions (anaphylaxis and unique clinical problems associated with cross-reactivity and neutralization of endogenous molecules). Thus it becomes important to examine any associations between ADA or any of its attributes with the various clinical sequelae. The presence of ADA may or may not preclude the administration of drug to ADA-positive subjects because the outcome is dependent upon the magnitude of the impact of ADA on PK and PD. Hence, the relationship of ADA with PK/PD is an important additional consideration, but does not necessarily result in a clinically impactful consequence per se.

Immunogenicity Endpoints

A fundamental metric that informs clinical immunogenicity interpretation is the incidence of ADA in a study or across comparable studies. ADA incidence is defined as the proportion of the study population found to have seroconverted or boosted their pre-existing ADA during the study period.

Terms and Definitions

Validated ADA test methods enable characterization of samples into ADA-positive vs. ADA-negative. To classify the ADA status of a subject using data from an in vitro test method, each sample from the subject is categorized based on the following definitions:

Sample ADA Status**:**

- Baseline ADA-positive sample: ADA is detected in the last sample before initiation of treatment
- Baseline ADA-negative sample: ADA is not detected in the last sample before initiation of treatment
- ADA-positive sample: After initiation of treatment, (1) an ADA detected (positive seroconversion) sample in a subject for whom ADA is not detected at baseline, or (2) an ADA detected sample with ADA titer to be at least 4-fold or greater (≥) than baseline positive titer
- ADA-negative sample: After initiation of treatment, ADA not positive sample relative to baseline

Next, using the sample ADA status, subject ADA status is defined as follows:

Subject ADA Status:

- Baseline ADA-positive subject: A subject with baseline ADA-positive sample
- **ADA-positive subject**: A subject with at least one ADA positive-sample relative to baseline at any time after initiation of treatment

6) *Persistent Positive (PP):* ADA-positive sample at 2 or more consecutive time points, where the first and last ADA-positive samples are at least 16 weeks apart
7) Note that this will not be applicable for CA209816 since the sampling is limited to 3 cycles of 3 weeks.

8) *Last Sample Positive:* Not persistent positive with ADA-positive sample at the last sampling time point
9) *Other Positive:* Not persistent positive but some ADA-positive samples with the last sample being negative
10) *Neutralizing Positive:* At least one ADA-positive sample with neutralizing antibodies detected

- **ADA-negative subject:** A subject with no ADA-positive sample after the initiation of treatment.

(Note: 16 weeks was chosen based on a long half-life of IgG4.)

Population for Analyses

Analysis of immunogenicity data will be based on ADA evaluable subjects defined as all treated subjects with baseline and at least 1 post-baseline immunogenicity assessment. Analysis dataset and data listing will include all available ADA samples. However, subject-level ADA status will be defined based on only adequate samples (e.g., excluding 1-hour post-infusion samples when clearly indicated).

12 REFERENCES

[1] Mountain, CA. Regional Lymph node classification for lung cancer staging. Chest. 1997: 1718-23.

[2] Goldstraw P, Crowley J, Chansky K, et al for the International Association for the Study of Lung Cancer International Staging Committee. The IASLC Lung Cancer Staging Project: proposals for the revision of the TNM stage grouping in the forthcoming (seventh) edition of the TNM Classification of malignant tumours. J Thorac Onco. 2007; 2(8): 706-14

[3] Detterbeck FC, Boffa DJ, Tanoue LT. The new lung cancer staging system. Chest. 2009; 136: 260-71.

[4] Core Safety Statistical Analysis Plan for CA209, Bristol-Myers Squibb

[5] Provencio et al. Abs 8521 ASCO 2018

[6] Baden et al, Comparison of Platforms for Determining Tumor Mutational Burden in Patients With Non-Small Cell Lung Cancer, ESMO 2019

[7] Dolan P. Modeling valuations for EuroQol health states. Medical Care 1997;35: 1095-1108

[8] The EuroQol Group: EuroQol: A new facility for the measurement of health-related quality of life—The EuroQol Group. Health Policy 16:199-208, 1990.

[9] Glimm E., Maurer W., Bretz F. Hierarchical testing of multiple endpoints in group-sequential trials. Stat. Med. 2010;29:219–228.

10 Clopper, CJ and Pearson, ES. The use of confidence or fiducial limits illustrated in the case of the binomial. Biometrika 26: 404-423, 1934.

11 Brookmeyer R. and Crowley J. A confidence interval for the median survival time. Biometrics 38:29-41, 1982

12 Klein, J. P. and Moeschberger, M. L. (1997), Survival Analysis: Techniques for Censored and Truncated Data, New York: Springer-Verlag

13 Kalbfleisch RL. The Statistical Analysis of Failure Time Data. Wiley-Interscience, 2002.

14 Global Biometric Sciences, SAS Analysis Dataset Specification, Unique Adverse Events. Version 2.0, April 22, 2015

15 Statistical Methodology in the Pharmaceutical Sciences/ edited by Berry DA, Chapter 13, Categorical Data Analysis, p 415 and 417 ff., Marcel Dekker, 1990.

16 Greenwood, M. The errors of sampling of the survivorship tables, Reports on Public Health and Statistical Subjects, 33, Appendix 1, HMSO, London, 1926

17 Kalbfleisch, J. D. and Prentice, R. L. (1980), The Statistical Analysis of Failure Time Data, New York: John Wiley & Sons.

18 Adverse Event Domain Requirements Specification Bristol Myers Squibb Co. PRI. Version 2.3.0 April 23, 2018

19 Non-Study Medication Domain Requirements Specification Bristol Myers Squibb Co. PRI. Version 2.10.0 April 23, 2018

STATISTICAL ANALYSIS PLAN
FOR CLINICAL STUDY REPORT

RANDOMIZED, OPEN-LABEL, PHASE 3 TRIAL OF NIVOLUMAB PLUS IPILIMUMAB OR NIVOLUMAB PLUS PLATINUM-DOUBLET CHEMOTHERAPY VERSUS PLATINUM-DOUBLET CHEMOTHERAPY IN EARLY STAGE NSCLC

PROTOCOL(S) CA209816

VERSION # 3.0

DATE: 11-AUG-2021

TABLE OF CONTENTS

STATISTICAL ANALYSIS PLAN FOR CLINICAL STUDY REPORT 1
TABLE OF CONTENTS 2
LIST OF TABLES 6
LIST OF FIGURES 7
1 BACKGROUND AND RATIONALE 8
2 STUDY DESCRIPTION 9
2.1 Study Design 9
2.2 Treatment Assignment 12
2.3 Blinding and Unblinding 13
2.4 Protocol Amendments 13
2.5 Data Monitoring and Other External Committees 17
3 OBJECTIVES 17
3.1 Primary 17
3.2 Secondary 17
3.3 Exploratory Objectives 17
4 ENDPOINTS 18
4.1 Primary Endpoint(s) 18
4.1.1 Event-Free Survival 19
4.1.1.1 Primary Definition of Event-Free Survival 19
4.1.1.2 Secondary Definition of Event-Free Survival 20
4.1.2 Pathologic Complete Response Rate 22
4.2 Secondary Endpoint(s) 22
4.2.1 Overall Survival 22
4.2.2 Major Pathological Response Rate 22
4.2.3 Time to Death or Distant Metastases 23
4.3 Exploratory Endpoint(s) 23
4.3.1 Clinical Response Rate by BICR 23
4.3.2 Event Free Survival on Next Line of Therapy (EFS2) 23
4.3.3 Surgery Related Endpoints 24
4.3.4 Safety and Tolerability 24
4.3.5 Pharmacokinetics 24
4.3.6 Biomarkers 24
4.3.6.1 Tumor Mutational Burden 24
4.3.6.2 PD-L1 Protein Expression 25
4.3.7 Outcomes Research 25
4.3.7.1 EQ-5D-3L 25
5 SAMPLE SIZE AND POWER 27
5.1 Pathologic Complete Response (pCR) 28
5.2 Event Free Survival (EFS) 29
5.3 Power Considerations for Overall Survival 30
5.4 Analyses Timing Projections 31
6 STUDY PERIODS, TREATMENT REGIMENS AND POPULATIONS FOR ANALYSES 33
6.1 Study Periods 33
6.2 Treatment Regimens 34

6.3 Populations for Analyses 34
7 STATISTICAL ANALYSES 35
7.1 General Methods 35
7.1.1 Adverse Events, Serious Adverse Events, Multiple Events, Select Adverse Events, Other Events of Special Interest and Immune-Mediated Adverse Events 36
7.1.1.1 Select Adverse Events 37
7.1.1.2 Other Events of Special Interest 38
7.1.1.3 Immune-Mediated Adverse Events 38
7.1.2 Laboratory Tests 38
7.2 Study Conduct 39
7.2.1 Accrual 39
7.2.2 Relevant Protocol Deviations 39
7.3 Study Population 39
7.3.1 Subject Disposition 39
7.3.2 Demographics and Other Baseline Characteristics 40
7.3.3 Medical History 41
7.3.4 Prior Therapy 41
7.3.5 Physical Examinations 41
7.3.6 Baseline Physical Measurements 41
7.3.7 Consistency of Demographics and Baseline Characteristics by Randomization Period 41
7.4 Extent of Exposure 42
7.4.1 Administration of Study Therapy 42
7.4.2 Modifications of Study Therapy 46
7.4.2.1 Dose Delay/Omission 46
7.4.2.2 Infusion Interruptions and Rate Changes 46
7.4.2.3 Dose Reductions 46
7.4.3 Concomitant Medications 48
7.4.3.1 Immune Modulating Medication 48
7.4.3.2 Subsequent Cancer Therapy 49
7.5 Definitive Surgery 49
7.6 Efficacy 50
7.6.1 Type I Error Control 50
7.6.2 Analysis of Pathological Complete Response 51
7.6.2.1 Primary pCR Analysis 51
7.6.2.2 Supportive Analyses of pCR 51
7.6.2.3 Subset Analyses of pCR 52
7.6.2.4 Major Pathological Response Rate 53
7.6.2.5 Additional Pathological Related Analyses 53
7.6.2.6 Clinical Response Rate 53
7.6.3 Analysis of Event Free Survival 53
7.6.3.1 Primary Event Free Survival 53
7.6.3.2 Supportive Analyses of Event-Free Survival 54
7.6.3.3 Subset Analyses of EFS 57
7.6.3.4 EFS Analyses by pCR and MPR Status 57

Approved v3.0 [redacted]

7.6.3.5 Current Status of EFS ... 57
7.6.4 Analysis of Overall Survival ... 57
7.6.4.1 OS Analyses ... 57
7.6.4.2 Supportive Analyses for OS ... 58
7.6.4.3 Subset Analyses of OS ... 59
7.6.4.4 OS Landmark Analyses by pCR and MPR Status ... 60
7.6.4.5 Subject Follow-Up ... 60
7.6.5 Interim Analyses of EFS and OS ... 60
7.6.6 Analysis of TTDM ... 61
7.6.7 Event Free Survival on Next Line of Therapy ... 61
7.6.8 Consistency of Efficacy by Randomization Period ... 61
7.7 Safety ... 61
7.7.1 Definitive Surgery Related Safety ... 62
7.7.2 Deaths ... 62
7.7.3 Serious Adverse Events ... 62
7.7.4 Adverse Events Leading to Discontinuation of Study Therapy ... 63
7.7.5 Adverse Events Leading to Dose Modification ... 63
7.7.6 Adverse Events ... 63
7.7.7 Select Adverse Events ... 64
7.7.7.1 Incidence of Select AE ... 64
7.7.7.2 Time-to Onset of Select AE ... 64
7.7.7.3 Time-to Resolution of Select AE ... 65
7.7.8 Immune-Mediated Adverse Events ... 65
7.7.9 Other Events of Special Interest ... 66
7.7.10 Multiple Events ... 66
7.7.11 New Primary Cancers ... 66
7.7.12 Laboratory Parameters ... 66
7.7.12.1 Hematology ... 66
7.7.12.2 Serum Chemistry ... 67
7.7.12.3 Electrolytes ... 67
7.7.12.4 Additional Analyses ... 67
7.7.13 Vital Signs and Pulse Oximetry ... 68
7.7.14 Physical Measurements ... 68
7.7.15 Non-Protocol Medical Procedures ... 68
7.7.16 Immunogenicity Analysis ... 68
7.7.17 Pregnancy ... 68
7.7.18 Adverse Events By Subgroup ... 68
7.7.19 Consistency of Safety by Randomization Period ... 69
7.7.20 Covid-19 ... 69
7.8 Pharmacokinetics ... 69
7.9 Biomarkers ... 69
7.9.1 PD-L1 Expression ... 69
7.9.2 Tumor Mutational Burden (TMB) ... 71

[redacted]

[redacted]

7.10 Outcomes Research Analyses 74
7.11 Country Specific Analyses 74
8 CONVENTIONS 75
9 CONTENT OF REPORTS 76
10 DOCUMENT HISTORY 76
11 PREVIOUS ANALYSES 79
APPENDIX 1 TIME-TO ONSET AND TIME-TO RESOLUTION DEFINITION AND CONVENTIONS FOR SELECT ADVERSE EVENTS, IMMUNE-MEDIATED ADVERSE EVENTS AND EVENTS OF SPECIAL INTEREST 80
APPENDIX 2 MISSING AND PARTIAL RADIOTHERAPY AND SURGERY DATES IMPUTATION ALGORITHMS 82
12 REFERENCES 84

LIST OF TABLES

Table 2.4-1: Protocol Amendments 13
Table 4.3.7.1-1: Time Windows for EQ-5D-3L Assessments 26
Table 5.3-1: Power Calculation for EFS and OS 31
Table 7.4.1-1: Study Therapy Parameter Definitions- Nivolumab and Ipilimumab 42
Table 7.4.1-2: Study Therapy Parameter Definitions - Regimen 1: Vinorelbine/Cisplatin 43
Table 7.4.1-3: Study Therapy Parameter Definitions - Regimen 2: Docetaxel/Cisplatin 43
Table 7.4.1-4: Study Therapy Parameter Definitions - Regimen 3: Gemcitabine/Cisplatin 44
Table 7.4.1-5: Study Therapy Parameter Definitions - Regimen 4: Pemetrexed/Cisplatin 44
Table 7.4.1-6: Study Therapy Parameter Definitions - Regimen 5: Paclitaxel/Carboplatin 45
Table 7.4.2.3-1: Dose Modifications of Chemotherapeutic Agents (Arms B and C) 47
Table 7.4.2.3-2: Calculated Dose Ranges and Related Dose Levels 47
Table 10-1: Document History 76

LIST OF FIGURES

Figure 2.1-1: Study Design Schematic 12
Figure 4.1.1.1-1: EFS Primary Definition 20
Figure 4.1.1.2-1: EFS Secondary Definition 22
[redacted]

1 BACKGROUND AND RATIONALE

Approximately 80% of lung cancer cases are non-small cell lung cancer (NSCLC), with most patients presenting with late-stage disease. At initial diagnosis, 20% of patients present with stage I or II disease, whereas 30% present with stage III disease and 50% with stage IV disease. With enhanced lung cancer screening techniques, the percentage of patients diagnosed during the early stages may increase over the duration of the trial. A standard TNM staging system is used to determine the staging for NSCLC. Patients with pathologic stage I NSCLC have a 5-year survival of approximately 60%. Stage II to III NSCLC patients have a 5-year survival of approximately 25% to 40%.[1] Surgical resection remains the mainstay of treatment for stage I and II patients; however, despite potentially curative surgery, approximately 50% of stage IB and 60-75% of stage II NSCLC patients will relapse and eventually die of their disease.[2,3] A rational approach to improve survival in these patients is to eradicate micrometastatic disease and to minimize the risk of relapse after adjuvant or neoadjuvant chemotherapy.

The phase 3 study, CA209816, will evaluate the clinical efficacy and will establish the safety of nivolumab plus platinum doublet chemotherapy and nivolumab plus ipilimumab, in resectable lung cancer. Specifically, this study will compare EFS and pCR rate among participants treated with neoadjuvant nivolumab plus platinum doublet chemotherapy vs participants treated with platinum doublet chemotherapy, and will describe pCR rate and EFS for those treated with neoadjuvant nivolumab plus ipilimumab in Stage Ib-IIIa NSCLC.

This document contains description of the statistical analyses that will be conducted for the clinical study report (CSR) of study CA209816.

Research Hypothesis:

In participants with stage IB (≥ 4 cm), II or IIIA (N2) NSCLC considered resectable by the local multidisciplinary team, administration of neoadjuvant nivolumab plus platinum doublet chemotherapy (up to 3 cycles) has superior efficacy to neoadjuvant platinum doublet chemotherapy (up to 3 cycles).

Schedule of Analyses:

Formal analysis of Pathological Complete Response (pCR) will occur after the 350 randomized participants on Arms B and C from start of 1:1:1 randomization have an opportunity for surgery and is projected to occur approximately 30 months after 1:1:1 randomization.

Two formal interim analyses for Event Free Survival (EFS) are planned after 148 and 167 events have been observed on Arms B and C after start of 1:1:1 randomization, respectively. The second interim analysis may take place one year after the first interim analysis in case the required number of events is not yet reached at that time. This is projected to occur approximately 48 and 58 (or max 60) months after start of 1:1:1 randomization. The formal interim comparisons of EFS will allow for determination of superiority. If the study continues beyond these interim analyses, the final analysis will be conducted after approximately 185 EFS events have been observed on Arms B and C from start of 1:1:1 randomization, or at a maximum 4 years after the last subject's randomization (December 2023).

[redacted]

2 STUDY DESCRIPTION

2.1 Study Design

This is an open-label, randomized clinical trial of up to 3 cycles of neoadjuvant nivolumab (3 mg/kg every 2 weeks) and a single dose of 1 mg/kg dose of ipilimumab, nivolumab 360mg flat dose plus platinum doublet chemotherapy (up to 3 cycles), or platinum doublet chemotherapy (up to 3 cycles) as neoadjuvant treatment in participants with early stage (Stage IB [≥ 4 cm], II, and resectable IIIA [N2]) NSCLC.

The original study design (before revised protocol 02) had two arms. After signing the informed consent form and upon confirmation of the participant's eligibility, participants were randomized in an open-label fashion (1:1 ratio) to either neoadjuvant nivolumab plus ipilimumab or platinum doublet chemotherapy.

Revised protocol 02 added a new, neoadjuvant nivolumab plus platinum doublet chemotherapy arm. When the third arm had opened and as each site had received IRB/EC approval of revised protocol 02, the interactive response technology IRT switched to a 1:1:1 randomization at the respective site. Starting from that point on, the sites were only enrolling under revised protocol 02.

Revised protocol 03 withholds randomization into the arm of neoadjuvant nivolumab plus ipilimumab but continues randomizing eligible participants into either neoadjuvant nivolumab plus platinum doublet chemotherapy arm or platinum doublet chemotherapy arm. Participants already randomized in the original 2-arm part (neoadjuvant nivolumab plus ipilimumab vs neoadjuvant chemotherapy) and in the arm of neoadjuvant nivolumab plus ipilimumab in 3-arm part defined by revised protocol 02 will remain in trial and continue scheduled trial procedures. The primary population for comparisons of the primary endpoints is the subjects concurrently randomized in arms B and C (as of revised protocol 02).

As of Revised protocol 03, participants will be randomized between 2 arms in a 1:1 ratio to neoadjuvant nivolumab plus platinum doublet chemotherapy or platinum doublet chemotherapy. Eligible participants will be stratified by:

- PD-L1 expression (≥1% or <1%/not evaluable/indeterminate)
- Disease stage (IB/II vs IIIA)
- Gender

The treatment arms are as follows:

Arm A treatment: Participants randomized into Arm A received nivolumab 3 mg/kg IV over 30 minutes every 2 weeks for up to 3 doses (ie, 6 weeks of treatment; each cycle is 14 days). With Cycle 1 only, nivolumab was followed by a single dose ipilimumab 1 mg/kg IV over 30 minutes.

Arm B treatment: Participants randomized into Arm B will receive investigator-choice platinum doublet chemotherapy in 3-week cycles up to a maximum of 3 cycles (ie, 9 weeks of treatment; each cycle is 21 days):

- Regimen 1:
  - Vinorelbine 25 mg/m^2 or 30 mg/m^2 IV (per local prescribing information) push over 10 minutes or per institutional standard on Days 1 and 8
  - Cisplatin 75 mg/m^2 IV over 120 minutes or per institutional standard on Day 1, immediately following vinorelbine
- Regimen 2:
  - Docetaxel 60 mg/m^2 or 75 mg/m^2 IV (per local prescribing information) over 60 minutes or per institutional standard on Day 1
  - Cisplatin 75 mg/m^2 IV over 120 minutes or per institutional standard on Day 1, immediately following docetaxel
- Regimen 3 (squamous histology):
  - Gemcitabine 1000 mg/m^2 or 1250 mg/m^2 (per local prescribing information) IV over 30 minutes or per institutional standard on Days 1 and 8
  - Cisplatin 75 mg/m^2 IV over 120 minutes or per institutional standard on Day 1, immediately following gemcitabine
- Regimen 4 (non-squamous histology only):
  - Pemetrexed 500 mg/m^2 IV over 10 minutes or per institutional standard on Day 1
  - Cisplatin 75 mg/m^2 IV over 120 minutes or per institutional standard on Day 1, immediately following pemetrexed
- Regimen 5:
  - Paclitaxel 175 or 200 mg/m^2 IV over 180 minutes or per institutional standard on Day 1
  - Carboplatin AUC 5 or 6 IV over 30 minutes or per institutional standard on Day 1, immediately following paclitaxel

Arm C treatment: Participants randomized into Arm C will receive nivolumab 360 mg IV plus platinum doublet chemotherapy in 3-week cycles up to a maximum of 3 cycles of chemotherapy (ie, 9 weeks of treatment; each cycle is 21 days)

- Non-squamous NSCLC: nivolumab at a flat dose of 360 mg as 30-minute IV infusion on Day 1, followed by pemetrexed at a dose of 500 mg/m^2 IV over 10 minutes or per institutional standard and cisplatin at a dose of 75 mg/m^2 IV over 120 minutes or per institutional standard of a 3-week treatment cycle, for up to 3 cycles.

- Squamous NSCLC: nivolumab at a dose of flat dose of 360 mg as 30 minute IV infusion on Day 1, followed by gemcitabine at a dose of 1000 mg/m^2 or 1250 mg/m^2 (per local prescribing information) for a 30 minute IV infusion or per institutional standard and cisplatin at a dose of 75 mg/m^2 as a 120-minute IV infusion or per institutional standard, of a 3-week treatment cycle for up to 3 cycles. Gemcitabine will also be administered at a dose of 1000 mg/m^2 or 1250 mg/m^2 as a 30 minute IV infusion or per institutional standard on day 8 of each 3-week treatment cycle.

- Any histology: nivolumab at a flat dose of 360 mg as 30-minute IV infusion on Day 1, followed by paclitaxel 175 or 200 mg/m^2 IV over 180 minutes or per institutional standard and carboplatin AUC 5 or 6 IV over 30 minutes or per institutional standard of a 3-week treatment cycle, for up to 3 cycles.

Following the completion of neoadjuvant treatment, all participants who remain operative candidates will undergo definitive surgery for their NSCLC within 6 weeks after completing neoadjuvant treatment.

Following definitive surgery, participants in each arm may receive up to 4 cycles of adjuvant chemotherapy with or without radiation per institutional standard at the discretion of the investigator. Investigators may choose from the following post-operative regimens:

- Regimen 1:
  - Vinorelbine 25 mg/m^2 or 30 mg/m^2 IV (per local prescribing information) push over 10 minutes or per institutional standard on Days 1 and 8
  - Cisplatin 75 mg/m^2 IV over 120 minutes or per institutional standard on Day 1, immediately following vinorelbine
- Regimen 2:
  - Docetaxel 60 mg/m^2 or 75 mg/m^2 IV (per local prescribing information) over 60 minutes or per institutional standard on Day 1
  - Cisplatin 75 mg/m^2 IV over 120 minutes or per institutional standard on Day 1, immediately following docetaxel
- Regimen 3 (squamous histology):
  - Gemcitabine 1000 mg/m^2 or 1250 mg/m^2 IV (per local prescribing information) over 30 minutes or per institutional standard on Days 1 and 8
  - Cisplatin 75 mg/m^2 IV over 120 minutes or per institutional standard on Day 1, immediately following gemcitabine
- Regimen 4 (non-squamous histology only):
  - Pemetrexed 500 mg/m^2 IV over 10 minutes or per institutional standard on Day 1
  - Cisplatin 75 mg/m^2 IV over 120 minutes or per institutional standard on Day 1, immediately following pemetrexed
- Regimen 5:
  - Paclitaxel 175 or 200 mg/m^2 IV over 180 minutes or per institutional standard on Day 1
  - Carboplatin AUC5 or 6 IV over 30 minutes or per institutional standard on Day 1, immediately following paclitaxel

The study design schematic is presented in Figure 2.1-1.

Figure 2.1-1: Study Design Schematic

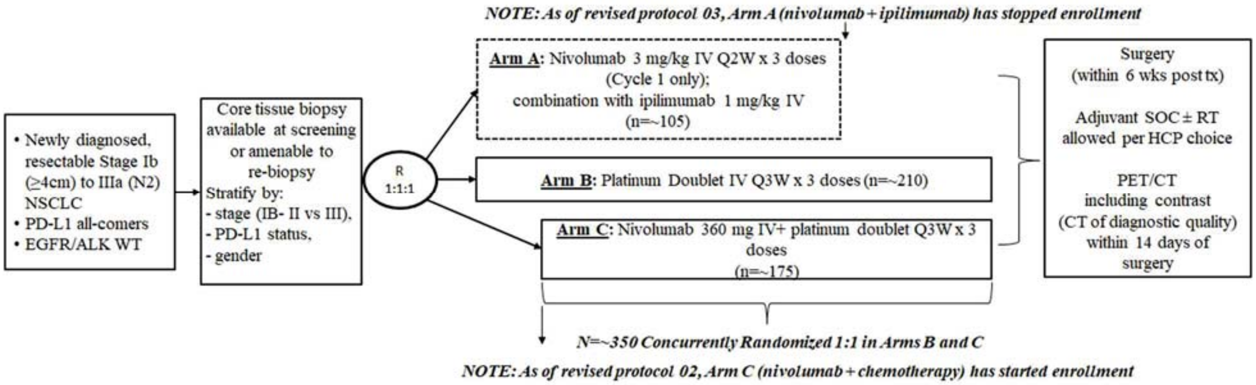

A Data Monitoring Committee (DMC) will be established to provide oversight of safety and efficacy and overall risk/benefit monitoring of the study.

Note that in this document the words “participant” and “subject” are used intercheangeably.

2.2 Treatment Assignment

CA209816 is an open-label, randomized trial. Participants with Stage IB (≥ 4 cm), II and IIIA (N2) considered resectable will be eligible to participate. After the participant’s initial eligibility is established and informed consent has been obtained, the participant must be enrolled into the study by calling the IRT to obtain a participant number. Every participant that signs the informed consent form must be assigned a participant number in IRT.

Once enrolled in IRT, enrolled participants who have met all eligibility criteria will be ready to be randomized through IRT to treatment Arm A, Arm B or Arm C.

In the original study design (before revised protocol 02) subjects were randomized to in a 1:1 ratio to arms A or B.

Following revised protocol 02, subjects were randomized to in a 1:1:1 ratio to arms A, B or C.

As of revised protocol 03, subjects were randomized to in a 1:1 ratio to arms B or C.

- Arm A: nivolumab 3 mg/kg plus ipilimumab 1 mg/kg.
- Arm B: platinum doublet chemotherapy.
- Arm C: nivolumab 360mg flat dose plus platinum doublet chemotherapy

The randomization uses permuted blocks stratified by the following factors:

- PD-L1 expression (≥1% or <1%/not evaluable/indeterminate)
- Disease stage (IB/II vs IIIA)
- Gender

2.3 Blinding and Unblinding

This is an open-label study; blinding procedures between participants and investigators are not applicable. The specific treatment to be taken by a participant will be assigned using an IRT. No aggregate summary data by treatment group are disclosed to the study team at any time of the study conduct until achievement of primary endpoint significance (pCR).

As described in the DMC charter, at the time of the first EFS interim analysis, in case EFS is not significant, the closed DMC report (unblinded) will still be shared with a BMS executive restricted team (3 persons named in DMC charter) to possibly engage in conversation with health authorities based on a trend in EFS (even if not significant), in the context of a statistically significant result in the pCR primary endpoint.

After discussion with health authorities, the BMS Executive restricted team may decide to share the DMC closed report with limited additional BMS members in order to prepare a broader health authority interaction. These additional BMS members will be outside of the CA209816 study team in addition to a small number of oncology leaders who would remain firewalled to the study in case the decision following health authorities interaction would be not to proceed to application. The members of this health authority interaction preparation team will be documented in a BMS internal document prior to the DMC meeting.

After formal discussion with FDA, should BMS proceed with an application for registration, the BMS study team will be unblinded to the study results and data.

Treatment assignments will be released to the bioanalytical laboratory in order to minimize unnecessary analysis of samples.

The blinded independent pathology review (BIPR) and blinded independent central review (BICR) will be blinded to treatment arms.

2.4 Protocol Amendments

Table 2.4-1: Protocol Amendments

Document/Date of Issue	Summary of Change
Revised Protocol 07<br>Under finalization	To account for potential slowdown in event-free survival (EFS) events accrual in long-term follow-up the revised protocol was updated to:<br>• Revise EFS modeling assumptions to a piecewise exponential with lower events rate in the longer term.<br>• Include one additional EFS interim analysis at 90% information fraction [redacted]
Revised Protocol 06	• Clarified EFS definition

Table 2.4-1: Protocol Amendments

Document/Date of Issue	Summary of Change
14-Jul-2020	• Removed the 1st interim analysis of EFS and updated alpha spending of interim and final analyses of EFS<br>• Clarified that actual timing of analyses may differ from projected timin<br>• Removed text about descriptive EFS analysis<br>[redacted]
Revised Protocol 05<br>18-Sep-2019	• Modified pCR analysis population and projected timelines.<br>– Rationale: based on FDA feedback indicating that the subset of 260 patients might not yield adequate numbers of patients with a pCR upon which to base reasonable assumptions of clinical meaningfulness.<br>• Updated surgical approach endpoint<br>• Updated the censoring rule of TTDM<br>• No optional biopsy at disease progression collected in China.<br>• Updated Management Algorithms to include myocarditis
Revised Protocol 04<br>25-Jun-2019	[redacted]<br>• Added the concomitant administration of substances that are also tubularly secreted (eg, probenecid) could potentially result in delayed clearance of pemetrexed.<br>• Added hypothesis testing for overall survival<br>• Clarified the pCR analysis population<br>• Added exploratory endpoint of Event Free Survival on next line of therapy<br>• Added instructions for BICR<br>• Updated Appendix 8 Adverse Events and Serious Adverse Events: Definitions and Procedures for Recording, Evaluating, Follow Up and Reporting Adverse Events
Revised Protocol 03<br>21-Sep-2018	• Enrollment in Arm A (nivolumab plus ipilimumab) was stopped. Based on external data from a neo-adjuvant phase II trial (NADIM[4]) which suggested anti-PD-1 + chemotherapy to be more promising, clinical development of nivolumab + chemotherapy was prioritized.<br>• Randomization of participants (1:1 ratio) into Arm C and B will continue to for a total of 350 participants

Table 2.4-1: Protocol Amendments

Document/Date of Issue	Summary of Change
	• Definition of event free survival is clarified.<br>• Participants with large-cell neuroendocrine carcinoma tumor histology are excluded<br>• Additional platinum doublet chemotherapy regimen (paclitaxel/carboplatin) was added<br>• Dose modification for docetaxel was updated<br>• Time to death or distant metastases (TTDM) was added to secondary endpoints.<br>• Tumor assessments for participants who do not proceed to definitive surgery was clarified<br>• Endpoints and statistical analyses adapted consequently to Arm A discontinuation.<br>• Rationale, background information, and trial schematic were updated<br>• Pulmonary function parameters were clarified<br>• Time relationship between adjuvant radiotherapy and tumor imaging assessments was clarified<br>• Time window of Cycle 1 Day 1 end-of-infusion PK sampling for Arm A and C was clarified
Revised Protocol 02<br>06-Jul-2017	• Nivolumab plus platinum-doublet chemotherapy arm (Arm C) was added.<br>• The sample size was increased to 642 participants consequently to the addition of Arm C and change of primary endpoint.<br>• The primary objective was changed to multiple primary objectives of event free survival and pathological complete response; major pathological response was changed to the secondary objective.<br>• Additional rationale and background information was provided.<br>• Pre-screening tissue requirement was increased from minimum of 10 slides to 15 slides.<br>• Contrast requirements for brain MRI scans were updated.<br>• Time window for pulmonary function test window was expanded from within 28 days of randomization to within 6 weeks of randomization.<br>• Synopsis was updated with exploratory objectives, endpoints and schema.<br>• Language in treatment administered was deleted and reference to Investigator Brochure and Pharmacy Manual was included.<br>[redacted]
Revised Protocol 01	• Incorporates Amendment 02 and Administrative Letters 01 and 02

Table 2.4-1: Protocol Amendments

Document/Date of Issue	Summary of Change
03-Mar-2017	
Amendment 02<br>03-Mar-2017	• To adjust the dosing details of the chemotherapy regimens to include the dose approved by the local prescribing information and the standard of care infusion time for each country included in this study.<br>• To expand and to split the broad biomarker objective into 3 more detailed objectives.<br>• Clarify lymph node samples at screening and at definitive surgery.<br>• Clarify requirements for PET/CT scans and broadening the window of scans prior to surgery.<br>• Clarify tissue sample process for calculation of the primary endpoint.<br>• Adjust Hepatitis B Virus criteria.<br>• Added live vaccines and strong CYP3A4 inhibitors to the Prohibited Treatments.<br>• added caution for concomitant administration of NSAIDs with pemetrexed<br>• added unacceptable methods of contraception to Appendix 6.
Administrative Letter 02<br>30-Nov-2017	• Clarify the correct version of the TNM Staging System.<br>• Clarify that a minimum of 228 PD-L1+ participants will be randomized.<br>• Clarify that physical exams, vital signs, and physical measurements should be collected prior to each dose of neoadjuvant and adjuvant therapy.<br>• Clarify that the first post-operative tumor assessment should be performed 12 weeks (± 7 days) after definitive surgery.<br>• Remove the phrase "non-protocol regimen" in regards to a noncisplatin<br>• Regimen as the protocol includes a non-cisplatin regimen option.<br>• Clarify that weight-based dosing should be rounded up to the nearest milligram or per institutional standards.<br>• Clarify that the EQ-5D-3L should be collected prior to Day 1 only in cycles that have multiple dosing days in each cycle.
Administrative Letter 01<br>31-Oct-2017	• To correct the IND number
Original Protocol	• Not Applicable

Table 2.4-1: Protocol Amendments

Document/Date of Issue	Summary of Change
30-Sep-2016	

2.5 Data Monitoring and Other External Committees

A Data Monitoring Committee (DMC) is established to provide oversight of safety and efficacy considerations in protocol CA209816. Additionally, the DMC will provide advice to the sponsor regarding actions the committee deems necessary for the continuing protection of participants enrolled in the study. The DMC will be charged with assessing such actions in light of an acceptable benefit/risk profile for nivolumab in combination with ipilimumab or chemotherapy. The DMC will act in an advisory capacity to BMS and will monitor participant safety and evaluate the available efficacy data for the study. The oncology therapeutic area of BMS has primary responsibility for design and conduct of the study.

Independent pathology (BIPR) and radiology review (BICR) will be established for central review and confirmation of efficacy endpoints.

3 OBJECTIVES

3.1 Primary

- To compare the event-free survival (EFS) by BICR in participants receiving nivolumab plus platinum doublet chemotherapy vs participants receiving platinum doublet chemotherapy in operable stage IB (≥ 4 cm), II, or resectable IIIA (N2) NSCLC
- To compare the pathologic complete response (pCR) rate in participants receiving nivolumab plus platinum doublet chemotherapy vs participants receiving platinum doublet chemotherapy in operable stage IB (≥ 4 cm), II, or resectable IIIA (N2) NSCLC

3.2 Secondary

- To assess the major pathologic response (MPR) rate by BIPR of participants receiving nivolumab plus platinum doublet chemotherapy vs participants receiving platinum doublet chemotherapy in operable stage IB (≥ 4 cm), II, or resectable IIIA (N2) NSCLC
- To assess the OS of participants receiving nivolumab plus platinum doublet chemotherapy vs participants receiving platinum doublet chemotherapy in operable stage IB (≥ 4 cm), II, or resectable IIIA (N2) NSCLC
- To assess the time to death or distant metastases (TTDM) of participants receiving nivolumab plus platinum doublet chemotherapy vs participants receiving platinum doublet chemotherapy in operable stage IB (≥ 4 cm), II, or resectable IIIA (N2) NSCLC

3.3 Exploratory Objectives

- To assess clinical response rate (cRR) by BICR of participants receiving nivolumab plus platinum doublet chemotherapy vs participants receiving platinum doublet chemotherapy in operable stage IB (≥ 4 cm), II, or resectable IIIA (N2) NSCLC

- To assess the pCR rate, MPR rate , cRR, EFS, TTDM and OS in early-stage NSCLC participants treated with nivolumab plus platinum doublet chemotherapy compared to those treated with platinum doublet chemotherapy by PDL1 status (PD-L1 ≥ 1% , PD L1< 1% /not evaluable/ indeterminate)
- To assess the feasibility of surgery and rate of peri- and post-operative complications (within 90 days of surgery) in participants receiving nivolumab plus platinum doublet chemotherapy compared to participants receiving platinum doublet
- To assess the safety and tolerability of nivolumab plus platinum doublet chemotherapy compared to platinum doublet chemotherapy in early stage NSCLC
- To describe the pCR rate, MPR rate, cRR, EFS, OS, TTDM, feasibility of surgery, rate of peri- and post-operative complications (within 90 days of surgery), safety and tolerability in early-stage NSCLC participants treated with nivolumab plus ipilimumab and by PDL1 status (PD-L1 ≥ 1% , PD L1< 1%/not evaluable/indeterminate)
- To assess pharmacokinetics of the nivolumab plus ipilimumab or nivolumab plus platinum doublet chemotherapy in participants with early stage NSCLC
- To assess the participant's overall health status and health utility using the 3-level version of the EQ-5D-3L visual analog scale (VAS) and utility index, respectively
- To evaluate tumor mutational burden as a potential predictive biomarker of efficacy (such as EFS and OS) of nivolumab plus platinum doublet chemotherapy and of platinum-doublet chemotherapy, using data generated from tumor and blood (germ-line control) specimens.

[redacted]

- To explore potential predictive biomarkers of nivolumab plus platinum doublet chemotherapy efficacy (such as EFS and OS) in peripheral blood and tumor specimens [redacted]

[redacted]

4 ENDPOINTS

4.1 Primary Endpoint(s)

The primary objectives in the study will be evaluated by the multiple primary endpoints of EFS and pCR.

4.1.1 Event-Free Survival

Two definitions are used for analysis of EFS. The primary definition accounts for subsequent therapy by censoring at the last evaluable tumor assessment on or prior to the date of subsequent therapy (outside of the protocol specified adjuvant therapy). The secondary definition does not incorporate censoring due to subsequent therapy.

EFS rate at time *T* is defined as the probability that a subject has not progressed/recurred and is alive at time *T* following randomization. EFS rates at fixed time points (e.g. 12 months, depending on the minimum follow-up) are defined as the probability that a subject has not progressed and is alive at time *T* following randomization.

4.1.1.1 Primary Definition of Event-Free Survival

Event free survival is defined as the length of time from randomization to any of the following events: any progression of disease precluding surgery, progression or recurrence disease after surgery (based on BICR assessment per RECIST 1.1), or death due to any cause.

- A pre-surgical progression (even if reaching the RECIST 1.1 criteria) which does not preclude surgery is not considered as an event.
- A progression not reaching the RECIST 1.1 criteria (e.g. clinical progression) but which still precludes surgery (i.e. reason for no surgery is disease progression) is considered as an event (event at the investigator reported earliest clinical or radiographic progression date, or at the date of randomization if no progression date reported).
- For participants with surgery, any new lesions identified by BICR on the first post-surgical baseline imaging compared with the pre-surgical scans will be identified as new lesion and will be counted as an event. For those without new lesion on the first post-surgical scan, the first tumor assessment post surgery will be used as re-baseline and recurrence/progression per RECIST 1.1 by BICR will be evaluated based on that re-baseline.
- Participants who do not undergo surgery for reason other than progression will be considered to have an event at RECIST 1.1 progression (based on BICR) or death.
- Participants who died without a reported progression/disease recurrence will be considered to have experienced an event on the date of their death.

The following censoring rules will be applied for the primary definition of EFS:

- Participants who did not report progression/recurrence of disease or die will be censored on the date of their last evaluable tumor assessment.
- Participants who did not have any on study tumor assessments and did not die will be censored on the date they were randomized.
- Subjects who receive subsequent anti-cancer therapy, outside of the protocol-specified adjuvant therapy, prior to documented progression/recurrence/death will be censored at the date of the last evaluable tumor assessment conducted on or prior to the date of initiation of the subsequent anti-cancer therapy.
- Subjects who did not have a documented progression/recurrence/death and received subsequent anti-cancer therapy outside of the protocol-specified adjuvant therapy will be

Approved v3.0 [redacted]

censored at the date of the last evaluable tumor assessment conducted on or prior to the initiation of the subsequent anti-cancer therapy.

- Participants without baseline scan and without surgery will be censored on the date of randomization (regardless of death).
- Censoring rules for the primary definition of EFS (EFS truncated at subsequent therapy) are presented as follows and depicted in Figure 4.1.1.1-1.
- It is to be noted that in case of new primary cancer, if such lesions are present on tumor assessment at the BICR, they will be considered as new lesions, since the BICR does not have access to biopsy results.

Figure 4.1.1.1-1: EFS Primary Definition

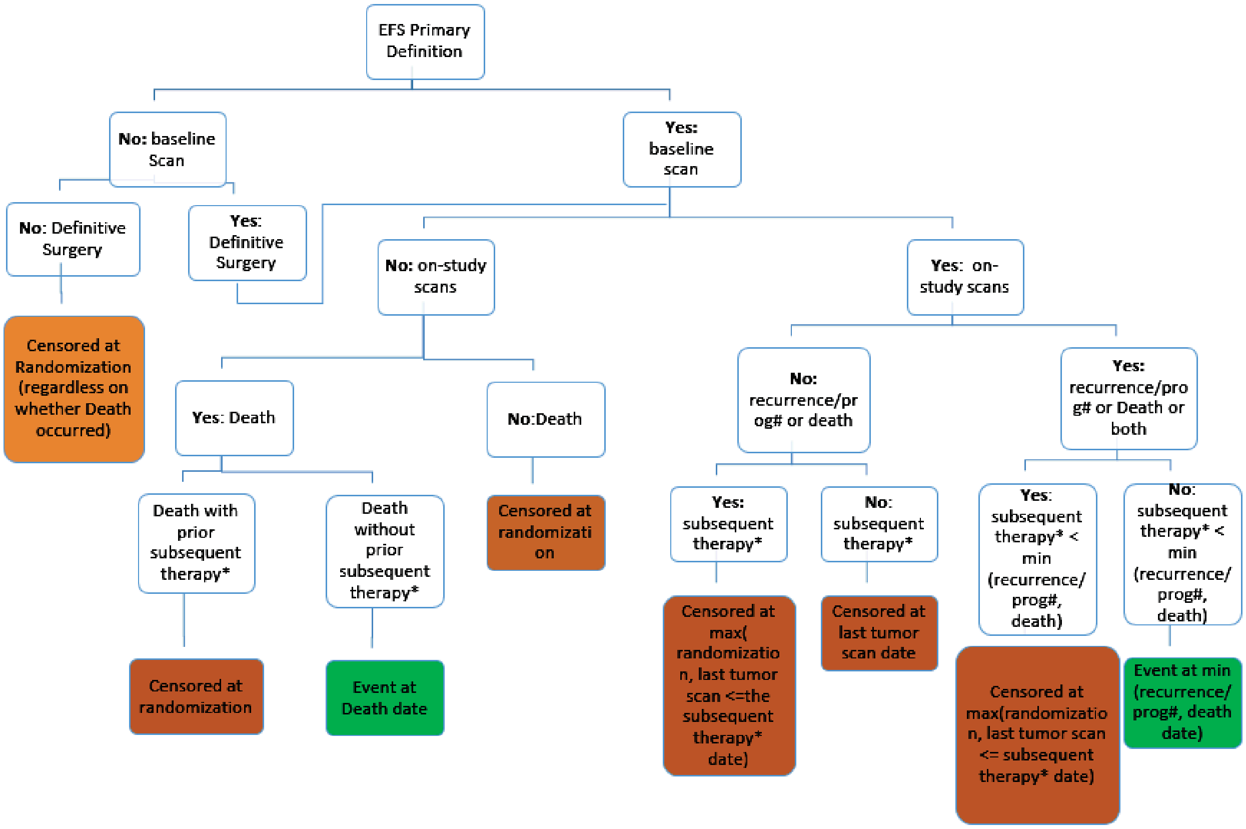

*Subsequent Therapy excluding per protocol adjuvant therapy

Progression precluding surgery, RECIST 1.1 recurrence or progression post surgery (for participants with surgery), RECIST 1.1 progression (for participants without surgery)

4.1.1.2 Secondary Definition of Event-Free Survival

The secondary definition of EFS (ITT definition) is defined as the length of time from randomization to any of the following events: any progression of disease precluding surgery, progression or recurrence disease after surgery (based on BICR assessment per RECIST 1.1), or death due to any cause.

- Progression/recurrence will be based on BICR assessment per RECIST 1.1.
- A progression (even if reaching the RECIST 1.1 criteria) which does not preclude surgery is not considered as an event.
- A progression not reaching the RECIST 1.1 criteria but which still precludes surgery (i.e. reason for no surgery is disease progression) is considered as an event (event at the investigator reported earliest clinical or radiographic progression date, or at the date of randomization if no progression date reported).
- For participants with surgery, the first tumor assessment post surgery will be used as re-baseline and recurrence/progression per RECIST 1.1 will be evaluated based on that re-baseline. Any new lesions on the post-surgical baseline imaging compared with the pre-surgical scans will be identified as new lesion and will be counted as an event.
- Participants who do not undergo surgery for reason other than progression will be considered to have an event at RECIST 1.1 progression or death.
- Participants who died without a reported progression/disease recurrence will be considered to have experienced an event on the date of their death.

The following censoring rules will be applied for the secondary definition of EFS:

- Participants who did not report progression/recurrence of disease or die will be censored on the date of their last evaluable tumor assessment.
- Participants who did not have any on study tumor assessments and did not die will be censored on the date they were randomized.
- Participants without baseline scan and without surgery will be censored on the date of randomization (regardless of death).
- Censoring rules for the secondary definition of EFS (ITT definition) are presented as follows and depicted in Figure 4.1.1.2-1

Figure 4.1.1.2-1: EFS Secondary Definition

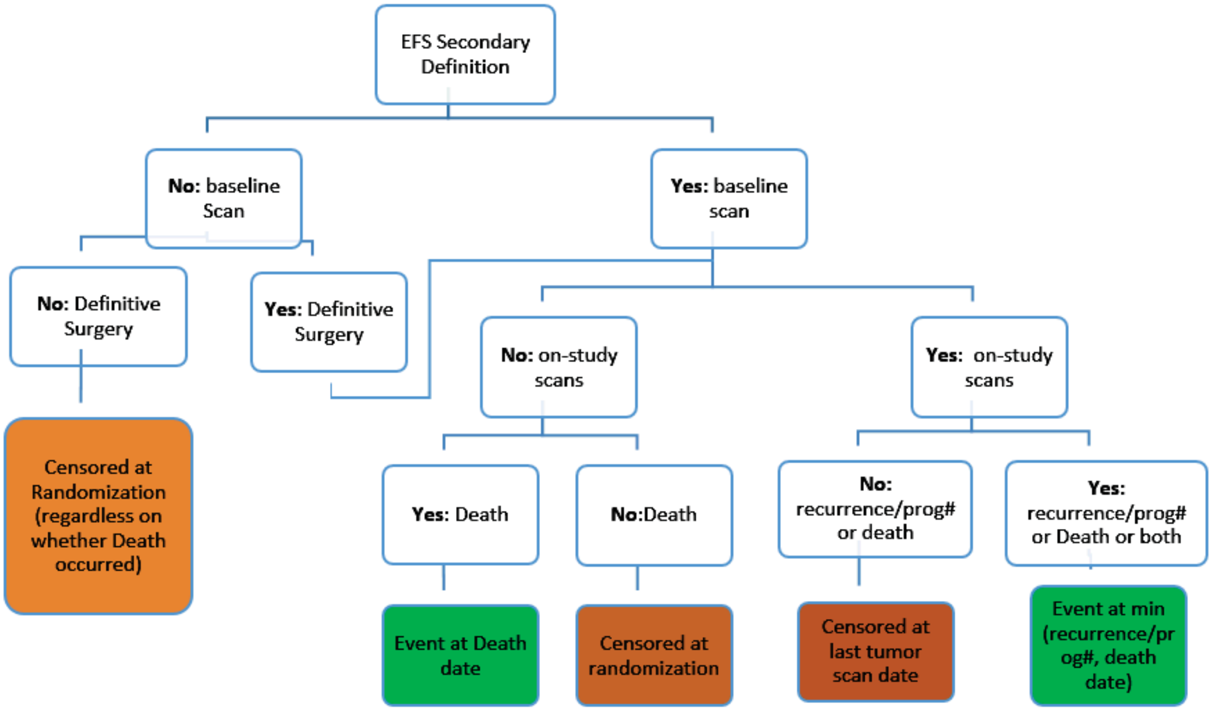

Progression precluding surgery, RECIST 1.1 recurrence or progression post surgery (for participants with surgery), RECIST 1.1 progression (for participants without surgery)

4.1.2 Pathologic Complete Response Rate

Pathological complete response (pCR) rate is defined as number of randomized participants with absence of residual tumor in lung and lymph nodes at surgery as evaluated by blinded independent pathological review (BIPR), divided by the number of randomized participants for each treatment group. Randomized subjects who are no longer eligible for surgery, or who are on alternative anti-cancer therapy before surgery, or who discontinue the study (e.g. withdraw consent) before surgery, or who otherwise do not have an evaluable BIPR result available are all counted as non-responders.

4.2 Secondary Endpoint(s)

4.2.1 Overall Survival

Overall survival (OS) is defined as the time between the date of randomization and the date of death due to any cause. For a subject without documentation of death, OS will be censored on the last date the subject was known to be alive.

4.2.2 Major Pathological Response Rate

Major pathological response (MPR) rate, defined as number of randomized participants with ≤ 10% residual tumor in lung and lymph nodes at surgery as evaluated by BIPR, divided by the number of randomized participants for each treatment group. Viable tumors in situ carcinoma should not be included in MPR calculation. Randomized subjects who are no longer eligible for

surgery, or who are on alternative anti-cancer therapy, or who discontinue the study (e.g. withdraw consent) before surgery, or who otherwise do not have an evaluable BIPR result available are all counted as non-responders.

4.2.3 Time to Death or Distant Metastases

Time to Death or Distant Metastates (TTDM) is defined as the time between the date of randomization and the first date of distant metastasis or the date of death in the absence of distant metastasis. Distant metastasis is defined as any new lesion that is outside of the thorax using BICR according to RECIST 1.1. It will be derived based on the location of lesions outside the thorax. Participants who died without reported distant metastasis will be considered to have experienced an event on the date of their death.

The following censoring rules will be applied TTDM:

- Participants who have not developed distant metastasis nor died will be censored on the date of their last evaluable tumor assessment.
- Participants who did not have any on study tumor assessments and did not die will be censored on the date they were randomized.

4.3 Exploratory Endpoint(s)

4.3.1 Clinical Response Rate by BICR

Clinical response rate (cRR) is defined as proportion of randomized participants whose overall radiological response prior to definitive surgery (or best overall radiological response (BOR) at the first protocol planned tumor assessment if a subject has no surgery) is either a complete response (CR) or partial response (PR) per RECIST 1.1 criteria by BICR. Participants who received alternative anti-cancer therapy before the pre-surgery tumor assessment will be counted as non-responders.

4.3.2 Event Free Survival on Next Line of Therapy (EFS2)

EFS on next line therapy (EFS2) is defined as the time from randomization to objectively documented progression, per investigator assessment, after the next line of therapy or to death from any cause, whichever occurs first. Subjects who were alive and without progression after the next line of therapy will be censored at last known alive date.

The following censoring rules will be applied for EFS2:

- Subjects who did not receive subsequent next line systemic anti-cancer therapy:
  - Subjects who died, the death date is the event date;
  - Else the subject's EFS2 is censored at the last known alive date.
- Subjects who received subsequent next line anti-cancer therapy:
  - Subjects who had a disease recurrence/progression after the start of subsequent anti-cancer therapy, this disease progression date is the event date;
  - Else if a subject died or start of second next line therapy, the date of min (death, start date of second next line therapy) is the event date;

- Else the subject's EFS2 is censored at the last known alive date.

Subsequent next line of therapy will include subsequent systemic regimen given in one of the following settings Unresectable, Locally Advanced or lines of therapy in metastatic setting.

4.3.3 Surgery Related Endpoints

The endpoints related to surgery include proportion of subjects with delayed (including duration of delay) or canceled surgery, duration of surgery, length of hospital stay, surgical approach, including completeness of surgery (R0/R1/R2 resection), incidence of AE/SAE associated with surgery up to 90 days after surgery.

4.3.4 Safety and Tolerability

The assessment of safety will be based on the incidence of adverse events (AEs), serious adverse events (SAEs), adverse events leading to discontinuation, adverse events leading to dose modification, select adverse events (select AEs) for EU/ROW Submissions, immune-mediated AEs (IMAEs) for US Submission, other events of special interest (OEOSI), and deaths. The use of immune modulating concomitant medication will be also summarized. In addition clinical laboratory tests will be analyzed.

4.3.5 Pharmacokinetics

Pharmacokinetics will be measured by the serum concentration of nivolumab and ipilimumab. Samples will be collected to characterize pharmacokinetics of nivolumab and ipilimumab and to explore exposure-safety and exposure-efficacy relationships. The population pharmacokinetics analysis will be presented separately from the main clinical study report.

4.3.6 Biomarkers

Biomarkers potentially associated with clinical endpoints will be measured by analyzing tumor and blood samples.

Biomarker endpoints include, but not limited to, tumor mutational burden (TMB) using data generated from tumor specimens., tumor inflammatory gene expression signatures using data generated from tumor specimen [redacted] Results for biomarkers analyses (other than PD-L1, TMB [redacted]) will be summarized outside of CSR.

4.3.6.1 Tumor Mutational Burden

TMB is measured in CA209816 using the [redacted] assay. [redacted] is a next-generation sequencing (NGS) assay targeting the full coding regions of 523 genes implicated in the pathogenesis of solid tumors. Using enrichment-based library preparation techniques for use with formalin-fixed, paraffin-embedded (FFPE) samples, [redacted] can analyze DNA and RNA from the same sample, detecting single nucleotide variants (SNVs), insertions and deletions (indels), amplifications, splice variants, and fusions, in a single sequencing run. TMB, is derived by summing the total of all synonymous and non-synonymous detected small DNA

variants (SNVs and indels) across the entire coding region (~1.3Mb are in coding regions) with sophisticated variant calling and germline filtering algorithms for enhanced accuracy. The resulting number is communicated as mutations per Mb unit (mut/Mb). The cutoff used for analysis will be ≥ 12.3 mut/Mb, <12.3 mut/Mb[5].

4.3.6.2 PD-L1 Protein Expression

PD-L1 expression is defined as the percent of tumor cells membrane staining in a minimum of 100 evaluable tumor cells per validated Dako PD-L1 immunohistochemistry (IHC) assay. This is referred to as quantifiable PD-L1 expression. If the PD-L1 staining could not be quantified, it is further classified as:

1) Indeterminate: Tumor cell membrane staining hampered for reasons attributed to the biology of the tumor tissue sample and not because of improper sample preparation or handling.
2) Not evaluable: Tumor tissue sample was not optimally collected or prepared and PD-L1 expression is neither quantifiable nor indeterminate. Not evaluable can be determined from H&E process before the tumor biopsy specimen is sent for PD-L1 evaluation or from the H&E process during PD-L1 evaluation.

Subjects with missing PD-L1 expression are subjects with no tumor tissue sample available for evaluation.

PD-L1 expression will be collected in the IRT as well as in the clinical database. Statistical analysis using PD-L1 expression will be solely based on PD-L1 expression data from clinical database. Stratified analyses will use stratification from IRT, unless otherwise specified.

4.3.7 Outcomes Research

4.3.7.1 EQ-5D-3L

Subjects' reports of general health status will be assessed using the EuroQoL Group's EQ-5D-3L. EQ-5D-3L essentially has 2 components: the descriptive system and the visual analogue scale (VAS).

The instrument's descriptive system consists of 5 dimensions: mobility, self-care, usual activities, pain/discomfort, and anxiety/depression. Each dimension has 3 levels, reflecting "no health problems," "moderate health problems," and "extreme health problems." A dimension for which there are no problems is said to be at level 1, while a dimension for which there are extreme problems is said to be at level 3. Thus, the vectors 11111 and 33333 represent the best health state and the worst health state, respectively, described by the EQ-5D-3L. Altogether, the instrument describes $3^5 = 243$ health states. Empirically derived weights can be applied to an individual's responses to the EQ-5D-3L descriptive system to generate an index measuring the value to society of his or her current health. Such preference-weighting systems have been developed for the UK, US, Spain, Germany, and numerous other populations. For this study, EQ-5D-3L utility index values will be computed using a scoring algorithm based on the United Kingdom Time-Trade-Off (UK TTO) value set[6]

Approved v3.0 [redacted]

In addition, the EQ-5D-3L includes a VAS, which allows respondents to rate their own current health on a 101-point scale ranging from 0=”worst imaginable” health to 100=“best imaginable” health state [7].

All questionnaires completed at baseline and on-study will be assigned to a time-point according to the windowing criteria in Table 4.3.7.1-1 and included in the analysis. In case a subject has two on-study assessments within the same window, the assessment closest to the time-point will be used and, in the case of two assessments at a similar distance to the time-point, the latest one will be chosen. In the event where the subject has no assessment at all in a specific window, the observation will be treated as missing for that time-point.

Table 4.3.7.1-1: Time Windows for EQ-5D-3L Assessments

Time Point	Nominal Day	Time Window
Baseline	D1	Prior to first dose on Day 1
Week 3 (Arm A)	D15	Day 2 thru day 22 inclusive
Week 4 (Arms B and C)	D22	Day 2 thru day 32 inclusive
Week 5 (Arm A)	D29	Day 23 thru day 36 inclusive
Week 7 (Arms B and C)	D43	Day 33 thru day 53 inclusive
Post-neoadjuvant visit 1	Last neodajuvant dose + 30 days	Assessment post last neoadjuvant dose and within 65 days of last neo dose
Post-neoadjuvant visit 2	Last neodajuvant dose + 100 days	Assessment post 65 days of last neoadjuvant dose and within 145 days of last neo dose
**Subjects without adjuvant:**		
Survival Follow-up 1	Last neodajuvant dose + 190 days	Assessment post 145 days of last neoadjuvant dose and within 235 days of last neo dose
Survival Follow-up 2	Last neodajuvant dose + 280 days	Assessment post 235 days of last neoadjuvant dose and within 325 days of last neo dose
Survival Follow-up 3	Last neodajuvant dose + 370 days	Assessment post 325 days of last neoadjuvant dose and within 415 days of last neo dose
Survival Follow-up 4	Last neodajuvant dose + 460 days	Assessment post 415 days of last neoadjuvant dose and within 550 days of last neo dose
Survival Follow-up 5	Last neodajuvant dose + 640 days	Assessment post 550 days of last neoadjuvant dose and within 730 days of last neo dose
Survival Follow-up 6	Last neodajuvant dose + 820 days	Assessment post 730 days of last neoadjuvant dose and within 910 days of last neo dose
Then Survival Follow-up i	Last neodajuvant dose + 460 + (i-4)*180 days	Assessment post (nominal day - 90) days of last neoadjuvant dose and within (nominal day + 90) days of last neo dose
**Subjects with systemic adjuvant:**		
Adjuvant Cycle 1	-	Assessment reported in the F01 (adjuvant cycle 1) visit

Table 4.3.7.1-1: Time Windows for EQ-5D-3L Assessments

Time Point	Nominal Day	Time Window
Adjuvant Cycle 2	-	Assessment reported in the F02 (adjuvant cycle 2) visit
Adjuvant Cycle 3	-	Assessment reported in the F03 (adjuvant cycle 3) visit
Adjuvant Cycle 4	-	Assessment reported in the F04 (adjuvant cycle 4) visit
Survival Follow-up 1	Max (Last adjuvant systemic dose or Post-neoadjuvant visit) + 90 days	Assessment post 45 days of Max (Last adjuvant dose or Post-neoadjuvant visit) and within 135 days of Max (Last adjuvant dose or Post-neoadjuvant visit)
Survival Follow-up 2	Max (Last adjuvant systemic dose or Post-neoadjuvant visit) + 180 days	Assessment post 135 days of Max (Last adjuvant dose or Post-neoadjuvant visit) and within 225 days of Max (Last adjuvant dose or Post-neoadjuvant visit)
Survival Follow-up 3	Max (Last adjuvant systemic dose or Post-neoadjuvant visit) + 270 days	Assessment post 225 days of Max (Last adjuvant dose or Post-neoadjuvant visit) and within 315 days of Max (Last adjuvant dose or Post-neoadjuvant visit)
Survival Follow-up 4	Max (Last adjuvant systemic dose or Post-neoadjuvant visit) + 360 days	Assessment post 315 days of Max (Last adjuvant dose or Post-neoadjuvant visit) and within 450 days of Max (Last adjuvant dose or Post-neoadjuvant visit)
Survival Follow-up 5	Max (Last adjuvant systemic dose or Post-neoadjuvant visit) + 540 days	Assessment post 450 days of Max (Last adjuvant dose or Post-neoadjuvant visit) and within 630 days of Max (Last adjuvant dose or Post-neoadjuvant visit)
Survival Follow-up 6	Max (Last adjuvant systemic dose or Post-neoadjuvant visit) + 720 days	Assessment post 630 days of Max (Last adjuvant dose or Post-neoadjuvant visit) and within 810 days of Max (Last adjuvant dose or Post-neoadjuvant visit)
Then Survival Follow-up i	Max (Last adjuvant systemic dose or Post-neoadjuvant visit) + 360 + (i-4)*180 days	Assessment post (nominal day - 90) days of last neo dose and within (nominal day + 90) days of last neo dose

5 SAMPLE SIZE AND POWER

The original study design (before Revised protocol 02) had two arms, with participants randomized in a 1:1 ratio to either neoadjuvant nivolumab plus ipilimumab or platinum doublet chemotherapy arm. Revised protocol 02 added a new, neoadjuvant nivolumab plus platinum doublet chemotherapy arm. When the third arm opens and as each site receives IRB/EC approval of revised

protocol 02, the IRT will switch to a 1:1:1 randomization at the respective site. Starting from that point on, the site will only enroll under revised protocol 02.

Revised protocol 03 withholds randomization into the arm of neoadjuvant nivolumab plus ipilimumab but continues randomizing eligible participants into either neoadjuvant nivolumab plus platinum doublet chemotherapy arm or platinum doublet chemotherapy arm in a 1:1 ratio. Approximately 350 participants (175 participants per arm) will be randomized between 2 arms neoadjuvant nivolumab plus platinum doublet chemotherapy or platinum doublet chemotherapy from 1:1:1 randomization in revised protocol 02 and 1:1 randomization in revised protocol 03. Participants already randomized in the original 2-arm part (neoadjuvant nivolumab plus ipilimumab vs neoadjuvant chemotherapy) and in the arm of neoadjuvant nivolumab plus ipilimumab in 3-arm part defined by revised protocol 02 will remain in trial and continue scheduled trial procedures. It is expected to have around 70 participants randomized in the original 2-arm part and approximately other 75 participants randomized in the arm of neoadjuvant nivolumab plus ipilimumab in the 3-arm part. It is estimated that there will be a total of approximately 500 participants on the study.

Starting from 1:1:1 randomization, approximately 350 participants will be randomized to the 2 arms neoadjuvant nivolumab plus platinum doublet chemotherapy or platinum doublet chemotherapy in a 1:1 ratio (concurrently randomized).

The sample size of the study is calculated based on the primary endpoint of EFS and accounts for the multiple primary endpoints comparisons: pCR (per BIPR) and EFS (per BICR) with an initial alpha allocation of 0.01 and 0.04 respectively. Formal analyses of pCR and EFS may be conducted at different timepoints. The fallback method will be used, ie, if the pCR comparison between Arm C and Arm B is statistically significant, then 0.01 alpha allocated to pCR will be passed to the EFS comparison for Arm C vs Arm B and the EFS comparison will be conducted at the alpha = 0.05 level. If the pCR comparison between Arm C and Arm B is not statistically significant, then the EFS comparison for Arm C vs Arm B will be conducted at the alpha = 0.04 level. [redacted]

5.1 Pathologic Complete Response (pCR)

The primary analysis of pCR will be performed after the 350 randomized participants in neoadjuvant nivolumab plus platinum doublet chemotherapy and platinum doublet chemotherapy (from start of 1:1:1 randomization) have an opportunity for surgery.

Assuming an accrual rate of 10 participants (all comers) a month between Arms B and C during 1:1:1 randomization (about 10 months), and 15 participants per month during 1:1 randomization, it is anticipated that the 350 participants will be randomized in approximately 27 months. The pCR endpoint is expected to be analyzed after about 30 months from start of 1:1:1 randomization.

Assuming pCR rate of 10% on Arm B chemotherapy and 30% on Arm C nivolumab plus chemotherapy, respectively, the 350 participants will provide more than 90% power to detect an odds ratio of 3.857 with a 2-sided type I error of 1%.

It is estimated that there will be about 110 subjects randomized to Arm A neoadjuvant nivolumab plus ipilimumab before revised protocol 03 is implemented. Assuming true pCR rate is 15% on this arm, there is 95% probability that the lower bound of 95% exact confidence interval of pCR is above 5%.

5.2 Event Free Survival (EFS)

For the formal comparison of EFS as assessed by BICR for nivolumab plus platinum doublet chemotherapy (Arm C) vs platinum doublet chemotherapy (Arm B), only participants randomized from 1:1:1 randomization in revised protocol 02 and 1:1 randomization in revised protocol 03 will be counted (participants concurrently randomized in arms B and C).

Considering this SAP revision (v3) occurs after the readout of the pCR primary endpoint which was statistically significant, the power details are provided below using alpha=0.05 (0.01 alpha from the pCR endpoint fallback to the EFS comparison. In addition, it reflects the number of subjects that were actually concurrently randomized in Arms B and C: 358 subjects.

A total of 185 events ensure that an overall 2-sided 5% significance level sequential test procedure with two interim analyses after 148 events (80% of events required for final analysis) and 167 events (90% of events required for final analysis) in 358 randomized participants will have 82% power assuming an HR of 0.65 between the 2 Arms. Considering a piecewise exponential distribution with control hazard rates of 0.028 before 20 months, 0.017 between 20 months and 40 months, 0.014 between 40 and 60 months and 0.008 after 60 months, and a dropout rate of approximately 20%, it is anticipated that the EFS analyses will take place at about 48, 58, and 73 months from start of 1:1:1 randomization. The trigger of the first interim analysis is event driven. The second interim analysis will take place when 167 events are observed [redacted]. The final analysis will take place when approximately 185 events are observed [redacted]. The stopping boundaries at the interim and final EFS analyses will be derived based on the exact number of events using Lan-DeMets alpha spending function with O'Brien-Fleming boundaries. If the interim analyses of EFS are performed at exactly 148 and 167 events, the nominal significance level for EFS superiority will be 0.024 and 0.030, respectively. The nominal significance level for the final look of EFS after 185 events would then be 0.038.

[redacted]

Table 5.3-1 summarizes the key parameters of the sample size justification in the concurrently randomized participants from Arms B and C.

Approved v3.0 [redacted]

5.3 Power Considerations for Overall Survival

The secondary endpoint Overall survival will be tested hierarchically after EFS with the same overall alpha as for the EFS comparison (two-sided 4% if the pCR comparison is not significant or 5% if the pCR comparison is significant).

For the formal comparison of OS for nivolumab plus platinum doublet chemotherapy (Arm C) vs platinum doublet chemotherapy (Arm B), only participants concurrently randomized from 1:1:1 randomization in revised protocol 02 and 1:1 randomization in revised protocol 03 will be included. [redacted]

Considering this SAP revision (v3) occurs after the readout of the pCR primary endpoint which was statistically significant, the power details are provided below using alpha=0.05 (0.01 alpha from the pCR endpoint fallback to the EFS comparison, then hierarchically on the OS comparison). In addition, it reflects the number of subjects that were actually concurrently randomized in Arms B and C: 358 subjects.

[redacted]

[redacted] The stopping boundaries at the interim and final OS analyses will be derived based on the exact number of events using Lan-DeMets alpha spending function with O'Brien-Fleming boundaries. This spending function is specific to OS and accounts for potential interim OS analyses even if they did not actually take place because of EFS non-significance[8].

[redacted]

Table 5.3-1 summarizes the key parameters of the power calculation for EFS and OS in the concurrently randomized participants from Arms B and C.

Table 5.3-1: Power Calculation for EFS and OS

	EFS<br>Arm C vs Arm B	OS<br>Arm C vs Arm B
Accrual	Actual accural<br>25 months	Actual accural<br>25 months
Power	82%	[redacted]
Two-sided alpha	0.05	0.05
Hypothesized Median Control vs exp (months)	28 vs 52*<br>Piecewise exponential model	[redacted]
Hypothesized Hazard ratio	0.65	0.65
Sample size for concurrent comparison	358	358
First interim analysis for EFS (EFS IA1) and OS (OS IA1)	148 events<br>Alpha boundary: 0.024	Triggered by EFS IA1<br>[redacted]
Second interim analysis for EFS (EFS IA2) and OS (OS IA2)	167 events [redacted]<br>Alpha boundary: 0.030	• If EFS IA1 not significant: triggered by EFS IA2.<br>[redacted]
Final EFS (EFS FA) and third OS (OS IA3) interim analysis	185 events [redacted]<br>Alpha boundary: 0.038	• If EFS IA2 not significant: triggered by EFS FA.<br>[redacted]
Final OS analysis (OS FA)	-	[redacted]

* Estimated from the piecewise model described in Section 5.2

5.4 Analyses Timing Projections

The pCR analysis occured with a database lock on 16-Sep-2020.

Considering the actual enrollment, it will take

- Approximately 48 months when 148 events on Arms B and C (after start of 1:1:1 randomization) are observed for the first interim analysis (EFS IA1, OS IA1). This is

about 54 months from FPFV of the study. This analysis is triggered by the number of EFS events. In case of significant EFS, OS will also be tested at that time (OS IA1).

- Approximately 58 months when 167 EFS events on Arms B and C (after start of 1:1:1 randomization) are observed for the second interim analysis (EFS IA2, OS IA2). This is about 64 months from FPFV of the study. [redacted]
- Approximately 73 months when 185 EFS events on Arms B and C (after start of 1:1:1 randomization) are observed for the final EFS analysis (EFS FA, OSIA3). This is about 79 months from FPFV of the study. [redacted]

[redacted]

6 STUDY PERIODS, TREATMENT REGIMENS AND POPULATIONS FOR ANALYSES

6.1 Study Periods

- Baseline period:
  - Baseline evaluations or events will be defined as evaluations or events that occur before the date and time of the first dose of study treatment. Evaluations (laboratory tests, pulse oximetry and vital signs) on the same date and time of the first dose of study treatment will be considered as baseline evaluations. Events (AEs) on the same date and time of the first dose of study treatment will not be considered as pre-treatment events.
  - In cases where the time (onset time of event or evaluation time and dosing time) is missing or not collected, the following definitions will apply:
    - ♦ Pre-treatment AEs will be defined as AEs with an onset date prior to but not including the day of the first dose of study treatment;
    - ♦ Baseline evaluations (laboratory tests, pulse oximetry and vital signs) will be defined as evaluations with a date on or prior to the day of first dose of study treatment.
  - If there are multiple valid observations in the baseline period, then the latest non missing observation will be used as the baseline in the analyses. If multiple observations exist on the latest collection date (and time if collected), the record with the latest data entry date and time will be used. If multiple observations exist on the latest collection date (and time if collected) and data entry date and time, then the first observation is used as baseline, unless otherwise specified.
    - ♦ For PD-L1, non-missing is identified as those with quantifiable test result. After applying the rule above, if there are no records with a quantifiable test result, then select those with indeterminate result ("INDETERMINATE"). If there are no records with

indeterminate test result, then select those with unavailable result ("NOT EVALUABLE"). If there are no records with unavailable test result, then select those which are not reported or not available result (all other records).

- Post baseline period:
  - Neoadjuvant on-treatment AEs will be defined as AEs with an onset date and time on or after the date and time of the first dose of neoadjuvant study treatment (or with an onset date on or after the day of first dose of neoadjuvant study treatment if time is not collected or is missing). For participants who are off neoadjuvant study treatment, AEs will be included if event occurred within a safety window of 30 days (or 100 days depending on the analysis) after the last dose of neoadjuvant study treatment. No "subtracting rule" will be applied when an AE occurs both pre-treatment and post-treatment with the same preferred term and grade.
  - Neoadjuvant on-treatment evaluations (laboratory tests, pulse oximetry and vital signs) will be defined as evaluations taken after the day (and time, if collected and not missing) of first dose of neoadjuvant study treatment. For participants who are off neoadjuvant study treatment, evaluations should be either within a safety window of 30 days (or 100 days depending on the analysis) after the last dose of neoadjuvant study treatment.
  - Adjuvant on-treatment AEs will be defined as AEs with an onset date and time on or after the date and time of the first dose of adjuvant systemic study treatment (or with an onset date on or after the day of first dose of adjuvant systemic study treatment if time is not collected or is missing). For participants who are off adjuvant study treatment, AEs will be included if event occurred within a safety window of 30 days after the last dose of adjuvant study treatment.

6.2 Treatment Regimens

The treatment group "as randomized" corresponds to the treatment group assigned by the Interactive Response Technology (IRT) system.

The treatment group "as treated" will be same as the treatment group "as randomized" by IRT unless a subject received the incorrect study treatment for the entire period of treatment, in which case the subject's treatment group "as treated" will be defined as the incorrect study treatment.

Unless otherwise specified, the safety analysis will be based on the treatment group "as treated".

Unless otherwise specified, the efficacy analysis will be based on the treatment group "as randomized".

The treatment arms are as follows:

- Arm A: nivolumab 3 mg/kg plus ipilimumab 1 mg/kg.
- Arm B: platinum doublet chemotherapy
- Arm C: nivolumab 360 mg plus platinum doublet chemotherapy

6.3 Populations for Analyses

- All Enrolled Participants: All participants who signed an informed consent form and were registered into the IRT.

- All Randomized Participants: All participants who were randomized to any treatment group in the study.
- All Treated Participants: All participants who received at least one dose of any study medication in neoadjuvant setting. This is the primary dataset for drug exposure and safety analysis for arm A.
- All Concurrently Randomized Participants in Arms B and C: All participants concurrently randomized on Arms B and C as of the 1:1:1 randomization. This will be the **primary analysis population** for efficacy.
- All Concurrently Randomized Participants in Arms A and B: All participants concurrently randomized on Arms A and B.
- All Treated Participants from the Concurrently Randomized Arms B and C: All participants concurrently randomized on Arms B and C as of the 1:1:1 randomization who received at least one dose of any study medication in the neoadjuvant setting. This will be the primary analysis population for drug exposure and safety for arms B and C.
- Tumor Tissue TMB evaluable subjects: All randomized subjects from the global study population with baseline evaluable tumor tissue TMB (non-missing numeric).

Concurrently randomized subjects from arms B and C (as of the 1:1:1 randomization) is considered at the site level basis, when the site switched to the revised protocol. In practice, this includes subjects randomized on the randomization lists from the 1:1:1 randomization (revised protocol 02) and the subsequent 1:1 randomization between B and C only (revised protocol 03).

Unless otherwise specified, all analyses will be performed using the treatment arm as randomized (intent to treat), with the exception of dosing and safety, for which the treatment arm as received will be used.

7 STATISTICAL ANALYSES

7.1 General Methods

Unless otherwise specified, analyses will be performed by treatment group (as randomized or as treated, depending on the analysis) for all concurrently randomized participants from Arms B and C. Descriptive analyses will also be produced for Arm A. Participants in Arm B randomized in the initial protocol will only be reported in listings and in the consistency by randomization period analyses (Sections 7.3.7, 7.6.8 and 7.7.19).

Unless otherwise noted, discrete variables will be tabulated by the frequency and proportion of subjects falling into each category, grouped by treatment. Percentages given in these tables will be rounded to the first decimal and, therefore, may not always sum to 100%. Percentages less than 0.1 will be indicated as '< 0.1'. If a missing category is not being presented in the data display, only those subjects with non-missing values for the parameter being assessed are included in the percentage calculation. Confidence intervals for binomial proportions will be derived using the Clopper-Pearson method[9].

Continuous variables will be summarized by treatment group using the mean, standard deviation, median, minimum, and maximum values and quartiles.

Time-to-event variables (e.g. time-to resolution, EFS) will be analyzed using the Kaplan-Meier technique. When specified, the median will be reported along with 95% CI using Brookmeyer and Crowley method[10] (using log-log transformation for constructing the confidence intervals[11]). Rates at fixed timepoints (e.g., OS at 12 months) will be derived from the Kaplan Meier estimate along with their corresponding log-log transformed confidence intervals[12].

Unless otherwise specified, the stratified hazard ratio between 2 treatment groups along with CI will be obtained by fitting a stratified Cox model with the treatment group variable as unique covariate. Stratification factors per IRT (PD-L1 expression (≥1% or <1%/not evaluable/indeterminate), disease stage (IB/II vs IIIA) and gender).

Unless otherwise specified, the stratified log-rank test will be performed to test the comparison between time to event distributions (OS and EFS). Stratification factors will be as described above.

The p-values from sensitivity analyses for efficacy endpoints, if presented, are for descriptive purpose only and not adjusted for multiplicity.

The conventions to be used for imputing missing and partial dates for analyses requiring dates are described in Section 8.

Note that in this document the terms "participant" and "subject" are used interchangeably. Terminology used in CSR will follow the BMS standard at the time of CSR.

Additional analyses by country or region may be conducted separately for country specific submissions.

7.1.1 Adverse Events, Serious Adverse Events, Multiple Events, Select Adverse Events, Other Events of Special Interest and Immune-Mediated Adverse Events

Drug-related AEs are those events with relationship to study drug "Related", as recorded on the CRF. If the relationship to study drug is missing, the AE will be considered as drug-related.

Serious adverse events consist of AEs deemed serious by the Investigator and flagged accordingly in the CRF and clinical database.

Adverse events leading to study drug discontinuation are AEs with action taken regarding study drug(s) = "Drug was discontinued". This option is selected when at least one agent from the regimen is discontinued.

Adverse events leading to dose delay are AEs with action taken regarding study drug(s) = "Drug was delayed".

Adverse events leading to dose reduction are AEs with action taken regarding study drug(s) = "Dose was reduced".

Adverse events will be coded using the Medical Dictionary for Regulatory Activities (MedDRA), and the most recent version of the dictionary at the time of the database lock will be used. Adverse events results will be graded for severity using NCI Common Terminology Criteria for Adverse Events (CTCAE) and the version of the criteria specified in the protocol will be used (version 4).

In the AE summary tables, unless otherwise specified, subjects will be counted only once at the Preferred Term (PT), only once at the System Organ Class (SOC), and only once at subject level for the counting of total number of subjects with an AE. The AE tables will be sorted by the SOCs and then PTs. SOC will be ordered by descending frequency overall and then alphabetically. PTs will be ordered within SOC by descending frequency overall and then alphabetically. The sorting will be done based on the 'Any Grade' column of the experimental arm when arms are presented side-by-side.

Unless otherwise specified, the AE summary tables will be restricted to on-treatment events regardless of the causality.

Analyses that take into account the multiple occurrences of a given adverse event will be conducted (see Section 7.7.10). To prepare these analyses, the CRF data will be processed according to standard BMS algorithms[13] in order to collapse adverse event records into unique records based on the preferred term. These data will be presented as the rate per 100 person-years of exposure. These analyses will take into account all on-treatment events (allowing more than 1 event per subject) and the total exposure time. The person-year exposure will be computed as the sum over the subjects' neoadjuvant exposure expressed in years where the exposure time is defined as

- (Date of last dose of study treatment - date of first dose of study treatment + 31 days (or 101 days, depending on the analysis))/365.25, for subject who are off study treatment and were followed for at least 30 days (or 100 days, depending on the analysis) after last dose of study treatment.
- (Last known alive date - date of first dose of study treatment +1)/365.25, for subjects who are still on-treatment or who are off study treatment and were followed less than 30 days (or 100 days depending on the analysis) after last dose of study treatment.

7.1.1.1 Select Adverse Events

The select Adverse Events (select AEs) consist of a list of preferred terms grouped by specific category (e.g. pulmonary events, gastrointestinal events categories, etc.). AEs that may differ from or be more severe than AEs caused by non-immunotherapies and AEs whose early recognition and management may mitigate severe toxicity are included as select AEs. Categories of select AEs may include subcategories (e.g. adrenal disorders, diabetes, pituitary disorders, and thyroid disorders are subcategories of the endocrine event category).

The list of MedDRA preferred terms used to identify select adverse events is revisited quarterly and updated accordingly. The preferred terms used for the selection at the time of the database lock will be provided by categories/subcategories.

In addition to the frequency and worst severity of select AEs, time-to onset, time-to resolution, and time-to resolution where immune modulating medication was initiated will be analyzed for each specific category/subcategory of drug-related select AEs when applicable.

Further details on the definitions time-to onset and time-to resolution are described in APPENDIX 1.

7.1.1.2 Other Events of Special Interest

Other events of special interest (OEOSI) consist of a list of preferred terms grouped by specific category (e.g. Myositis Event, Myocarditis Event, Demyelination Event, Guillain-Barre Syndrome, Pancreatitis Event, Uveitis Event, Encephalitis Event, Myasthenic Syndrome, Rhabdomyolysis Event, Graft Versus Host Disease). The list of MedDRA preferred terms used to identify OEOSI is revisited quarterly and updated accordingly. The preferred terms used for the selection at the time of the database lock by categories will be provided.

7.1.1.3 Immune-Mediated Adverse Events

In order to further characterize AEs of special clinical interest, analysis of immune-mediated AEs (IMAE) will be conducted. IMAEs are specific events (or groups of PTs describing specific events) that include pneumonitis, diarrhea/colitis, hepatitis, nephritis/renal dysfunction, rash, endocrine (adrenal insufficiency, hypothyroidism/thyroiditis, hypothyroidism, thyroiditis, hyperthyroidism, diabetes mellitus, and hypophysitis), and other specific events, considered as potential immune-mediated events by investigator that meet the definition summarized below:

- those occurring within 100 days of the last dose,
- regardless of causality,
- treated with immune-modulating medication (of note, endocrine AEs such as adrenal insufficiency, hypothyroidism/thyroiditis, hypothyroidism, thyroiditis, hyperthyroidism, diabetes mellitus, and hypophysitis are considered IMAEs regardless of immune-modulating medication use, since endocrine drug reactions are often managed without immune-modulating medication).
- with no clear alternate etiology based on investigator assessment, or with an immune-mediated component

The list of MedDRA preferred terms used to identify IMAEs is revisited quarterly and updated accordingly. The preferred terms used for the selection at the time of the database lock by categories will be provided.

7.1.2 Laboratory Tests

Clinical laboratory parameters (hematology, serum chemistry and electrolytes) will be evaluated.

Laboratory tests will be graded using the NCI Common Terminology Criteria, and the most recent version of the criteria at the time of the database lock will be used.

Clinical laboratory data will be analyzed using International System of Units (SI). Analyses will be repeated using US conventional units.

In the laboratory summary tables, unless otherwise specified, subjects will be counted only once for each lab parameter according to their worst on treatment CTC grade (worst being the highest CTC grade). The laboratory tables and listings will be sorted by laboratory category, laboratory subcategory and laboratory test code sequence number.

7.2 Study Conduct

Unless otherwise specified, analyses will be performed by treatment group as randomized for all concurrently randomized participants from Arms B and C. Descriptive analyses will also be produced for Arm A. Participants in Arm B randomized in the initial protocol will only be reported in listings.

For analysis based on enrolled subjects, the analysis population will consist of the all enrolled population.

7.2.1 Accrual

Enrollment and randomization by country and site, and enrollment and randomization by month will be summarized and listed for all enrolled and randomized subjects.

7.2.2 Relevant Protocol Deviations

Unless otherwise specified, analyses will be performed by treatment group as randomized for all concurrently randomized participants from Arms B and C. Descriptive analyses will also be produced for Arm A. Participants in Arm B randomized in the initial protocol will only be reported in listings.

<u>Eligibility:</u>

- Inadequate disease stage: presence of locally advanced unresectable (regardless of stage), stage IIIB or metastatic disease (stage IV) or stage IA disease.
- Subjects without measurable disease at baseline as per investigator.
- Subject with baseline ECOG performance status > 1.

<u>On-study:</u>

- Subjects receiving any concurrent anti-cancer therapy (chemotherapy, hormonal therapy, immunotherapy, radiation therapy, cancer related surgery (except definitive surgery), standard or investigational agents for treatment of cancer) outside of the protocol-specified neoadjuvant and adjuvant therapy (systemic and randiotherapy) while on study therapy (i.e. neoadjuvant or protocol adjuvant systemic treatment).
- Subjects whose "as treated" arm different than their as randomized arm (subjects who received the wrong treatment for the entire neoadjuvant treatment period, excluding the never treated)

7.3 Study Population

Unless otherwise specified, analyses will be performed by treatment group as randomized for all concurrently randomized participants from Arms B and C. Descriptive analyses will also be produced for Arm A. Participants in Arm B randomized in the initial protocol will only be reported in listings.

7.3.1 Subject Disposition

The total number of subjects enrolled (randomized or not randomized) will be presented along with the reason for not being randomized. This analysis will be performed on the all enrolled subjects population.

Number of subjects randomized but not treated along with the reason will be tabulated by treatment group as randomized.

Number of subjects who discontinued study treatment along with corresponding reason will be tabulated by treatment group as treated. Reason for discontinuation will be derived from subject status CRF page (including covid-19 reason). This analysis will be restricted to the all treated subjects population.

A by-subject listing for all treated subjects will be provided showing the subject's off treatment date along with the reason for going off treatment period. A by-subject listing for all enrolled subjects will also be provided, showing whether the subject was randomized/treated along with the reason for not being randomized/treated.

7.3.2 Demographics and Other Baseline Characteristics

The following demographic and baseline characteristics will be summarized and listed by treatment group as randomized:Age (descriptive statistics)

- Age (continuous)
- Age categorization (< 65, ≥ 65 and < 75, ≥ 75 and < 85, ≥ 85, ≥ 75, ≥ 65)
- Sex (male vs. female, CRF)
- Sex (male vs. female, IRT)
- Race (white, black , asian [asian Indian, Chinese, Japanese, asian other], American Indian or Alaska Native, Native Hawaiian or Other Pacific Islander, Other)
- Ethnicity (Hispanic/Latino, Not Hispanic/Latino, Unknown) - Required for US subjects only, but any available data will be presented
- Country by geographic region (North America, Europe, Asia, Rest of World)

The following baseline disease characteristics will be summarized by treatment group as randomized.

- Baseline ECOG performance status
- Baseline weight
- Tobacco use (Never Smoker, Current/Former, Unknown).
- Electronic Cigarette Use (Never Smoker, Current/Former, Unknown).
- Disease stage at study entry (CRF)
- Disease stage at study entry (IRT)
- Cell type (histology) at study entry (squamous cell carcinoma, non-squamous: adenocarcinoma, large cell carcinoma, broncho-alveolar carcinoma, other)
- Time from current NSCLC diagnosis to randomization
- Sites of diseases (all lesions) per BICR,
- Number of disease sites per subject (all lesions) per BICR
- Number of target lesions, non-target lesions and disease sites at baseline as per BICR

- Sum of the diameters of target lesions as baseline per BICR
- PD-L1 expression subgroups (clinical database, (<1%, >=1%, 1-49%, >=50%, inderterminate, not evaluable))
- PD-L1 expression subgroups (IRT, (<1%, >=1%/indeterminate/not evaluable)
- Tumor Tissue TMB subgroups (≥12.3 mut/MB, < 12.3 mut/MB, not evaluable)

Summary table (cross-tabulation) by treatment group for stratification factor will be provided to show any discrepancies between what was reported through IRT vs. CRF/Clinical database at baseline. This summary will be performed based on all randomized subjects.

- PD-L1 status (IRT vs. clinical database)
- Disease stage (IRT vs CRF data)
- Gender (IRT vs CRF data)
-

A listing of randomization scheme presenting randomized treatment group and as treated treatment group will be provided for all randomized subjects.

7.3.3 Medical History

A by-subject listing of general medical history for all randomized subjects will be provided.

7.3.4 Prior Therapy

- Prior/current non-study medication classified by anatomic and therapeutic classes.

Agents and medication will be reported using the generic name. A listing by subject will also be provided.

7.3.5 Physical Examinations

Subjects with abnormal baseline physical examination will be listed by subject.

7.3.6 Baseline Physical Measurements

Baseline physical measurements will be listed by subject.

7.3.7 Consistency of Demographics and Baseline Characteristics by Randomization Period

Consistency of population amongs the different randomization periods will be examined by summarizing the characteristics listed in Section 7.3.2 by treatment arm as randomized in the the different randomization period: before revised protocol 02 (1:1 A vs B), under revised protocol 02 (1:1:1 A vs B vs C) and after revised protocol 03 (1:1 B vs C).

Concurrent randomization is considered at the site level basis, when the site switched to the revised protcol. In practice, this includes subjects randomized on the randomization lists from the 1:1:1 randomization (revised protocol 02) and the subsequent 1:1 randomization between B and C only (revised protocol 03).

7.4 Extent of Exposure

Listings will include all available exposure data. Analyses will be performed by treatment group "as treated" in all treated subjects and for all treated participants from concurrently randomized Arms B and C, unless otherwise specified. Descriptive analyses will also be produced for Arm A. Participants in Arm B randomized in the initial protocol will only be reported in listings.

7.4.1 Administration of Study Therapy

The following parameters will be summarized (descriptive statistics) by treatment group:

- Number of neoadjuvant doses received by drug
- Cumulative dose by drug in neoadjuvant
- Relative dose intensity (%) by drug in neoadjuvant using the following categories: < 50%; 50 - < 70%; 70 - < 90%; 90 - < 110%; ≥ 110%
- Number of subjects who received protocol specified adjuvant systemic therapy number of adjuvant doses received by drug for subjects who received adjuvant therapy.
- The frequency of subjects receiving carboplatin instead of cisplatin in the regimens other than carboplatin-paclitaxel will be reported and the reason for not using cisplatin for regimens other that carboplatin-paclitaxel will be summarized.

A by-subject listing of dosing of study medication (record of study medication, infusion details, and dose changes) and a listing of batch numbers will be also provided.

- Number of subjects who received adjuvant radiotherapy, including radiotherapy type, number of doses and total cumulative dose.

Table 7.4.1-1 to Table 7.4.1-6 summarize the key parameters used to calculate dosing data.

Table 7.4.1-1: Study Therapy Parameter Definitions- Nivolumab and Ipilimumab

	Nivolumab	Nivolumab	Ipilimumab
Dosing schedule per protocol	3 mg/kg every 2 weeks	360 mg every 3 weeks	1 mg/kg on first cycle
Dose	*Dose (mg/kg)* is defined as Total Dose administered (mg)/Most recent weight (kg). Dose administered in mg at each dosing date and weight are collected on the CRF.	*Dose (mg)* is defined as Total Dose administered (mg) at each dosing date as collected on the CRF.	*Dose (mg/kg)* is defined as Total Dose administered (mg)/Most recent weight (kg). Dose administered in mg at each dosing date and weight are collected on the CRF.
Cumulative Dose	*Cum dose (mg/kg) is* sum of the doses (mg/kg) administered to a subject.	*Cum dose (mg)* is the sum of the doses (mg) administered to a subject.	*Cum dose (mg/kg) is* sum of the doses (mg/kg) administered to a subject.
Relative dose intensity (%)	Cum dose (mg/kg)/[(Last Nivolumab dose date - Nivolumab start dose date + 14) x 3/14] x 100	Cum dose (mg)/[(Last Nivolumab dose date - Nivolumab start dose date + 21) x 360/21] x 100	Cum dose (mg/kg) x 100

Approved v3.0 [redacted]

Table 7.4.1-2: Study Therapy Parameter Definitions - Regimen 1: Vinorelbine/Cisplatin

	Vinorelbine	Cisplatin
Dosing schedule per protocol	25 mg/m^2 or 30 mg/m^2 on Day1 and Day8 of a 3 week cycle.	75mg/ m² on Day 1 of a 3 week cycle
Dose	*Dose (mg/m²)* is defined as Total Dose administered (mg)/Most recent BSA. Dose administered in mg at each dosing date is collected on the CRF and BSA is derived from most recent weight and baseline height also collected on the CRF.	*Dose (mg/m²)* is defined as Total Dose administered (mg)/Most recent BSA. Dose administered in mg at each dosing date is collected on the CRF and BSA is derived from most recent weight and baseline height also collected on the CRF.
Cumulative Dose	*Cum dose (mg/ m²) is* sum of the doses (mg/ m^2) administered to a subject.	*Cum dose (mg/m²) is* sum of the doses (mg/m²) administered to a subject.
Relative dose intensity (%)	Cum dose (mg/m²)/[(First Vinorelbine dose date in the last cycle - Vinorelbine Start dose date + 21) x 50/21] x 100 or Cum dose (mg/m²)/[(First Vinorelbine dose date in the last cycle - Vinorelbine Start dose date + 21) x 60/21] x 100	Cum dose (mg/m²)/[(Last Cisplatin dose date - Start Cisplatin dose date + 21) x 75/21] x 100

Table 7.4.1-3: Study Therapy Parameter Definitions - Regimen 2: Docetaxel/Cisplatin

	Docetaxel	Cisplatin
Dosing schedule per protocol	60 mg/m^2 or 75 mg/m^2 on Day1 of a 3 week cycle.	75mg/ m² on Day 1 of a 3 week cycle
Dose	*Dose (mg/m²)* is defined as Total Dose administered (mg)/Most recent BSA. Dose administered in mg at each dosing date is collected on the CRF and BSA is derived from most recent weight and baseline height also collected on the CRF.	*Dose (mg/m²)* is defined as Total Dose administered (mg)/Most recent BSA. Dose administered in mg at each dosing date is collected on the CRF and BSA is derived from most recent weight and baseline height also collected on the CRF.
Cumulative Dose	*Cum dose (mg/ m²) is* sum of the doses (mg/ m^2) administered to a subject.	*Cum dose (mg/m²) is* sum of the doses (mg/m²) administered to a subject.
Relative dose intensity (%)	Cum dose (mg/m²)/[(First Docetaxel dose date in the last cycle - Docetaxel Start dose date + 21) x 60/21] x 100 or Cum dose	Cum dose (mg/m²)/[(Last Cisplatin dose date - Start Cisplatin dose date + 21) x 75/21] x 100

Approved v3.0 [redacted]

Table 7.4.1-3: Study Therapy Parameter Definitions - Regimen 2: Docetaxel/Cisplatin

	Docetaxel	Cisplatin
	(mg/m²)/[(First Docetaxel dose date in the last cycle - Docetaxel Start dose date + 21) x 75/21] x 100	

Table 7.4.1-4: Study Therapy Parameter Definitions - Regimen 3: Gemcitabine/Cisplatin

	Gemcitabine	Cisplatin
Dosing schedule per protocol	1250 mg/m^2 or 1000 mg/m^2 on Day1 and Day8 of a 3 week cycle.	75mg/ m² on Day 1 of a 3 week cycle
Dose	*Dose (mg/m²)* is defined as Total Dose administered (mg)/Most recent BSA. Dose administered in mg at each dosing date is collected on the CRF and BSA is derived from most recent weight and baseline height also collected on the CRF.	*Dose (mg/m²)* is defined as Total Dose administered (mg)/Most recent BSA. Dose administered in mg at each dosing date is collected on the CRF and BSA is derived from most recent weight and baseline height also collected on the CRF.
Cumulative Dose	*Cum dose (mg/ m²) is* sum of the doses (mg/ m^2) administered to a subject.	*Cum dose (mg/m²) is* sum of the doses (mg/m²) administered to a subject.
Relative dose intensity (%)	Cum dose (mg/m²)/[(First Gemcitabine dose date in the last cycle - Gemcitabine Start dose date + 21) x 2500/21] x 100 or Cum dose (mg/m²)/[(First Gemcitabine dose date in the last cycle - Gemcitabine Start dose date + 21) x 2000/21] x 100	Cum dose (mg/m²)/[(Last Cisplatin dose date - Start Cisplatin dose date + 21) x 75/21] x 100

Table 7.4.1-5: Study Therapy Parameter Definitions - Regimen 4: Pemetrexed/Cisplatin

	Pemetrexed	Cisplatin
Dosing schedule per protocol	500 mg/ m² every 3 weeks	75mg/ m² every 3 weeks

Approved v3.0 [redacted]

Table 7.4.1-5: Study Therapy Parameter Definitions - Regimen 4: Pemetrexed/Cisplatin

	**Pemetrexed**	**Cisplatin**
Dose	*Dose (mg/m²)* is defined as Total Dose administered (mg)/Most recent BSA. Dose administered in mg at each dosing date is collected on the CRF and BSA is derived from most recent weight and baseline height also collected on the CRF.	*Dose (mg/m²)* is defined as Total Dose administered (mg)/Most recent BSA. Dose administered in mg at each dosing date is collected on the CRF and BSA is derived from most recent weight and baseline height also collected on the CRF.
Cumulative Dose	*Cum dose (mg/m²) is* sum of the doses (mg/m²) administered to a subject.	*Cum dose (mg/m²) is* sum of the doses (mg/m²) administered to a subject.
Relative dose intensity (%)	Cum dose (mg/m²)/[(Last Pemetrexed dose date - Pemetrexed Start dose date + 21) x 500/21] x 100	Cum dose (mg/m²)/[(Last Cisplatin dose date - Cisplatin Start dose date + 21) x 75/21] x 100

Table 7.4.1-6: Study Therapy Parameter Definitions - Regimen 5: Paclitaxel/Carboplatin

	**Paclitaxel**	**Carboplatin**
Dosing schedule per protocol	200 mg/ m^2 or 175 mg/ m^2 every 3 weeks	AUC 5 or 6 every 3 weeks
Dose	*Dose (mg/m²)* is defined as Total Dose administered in mg at each dosing date is collected on the CRF and BSA is derived from most recent weight and baseline height also collected on the CRF.	*Dose (AUC)* is defined as Total Dose administered (mg)/(creatinine clearance +25). Dose administered in mg at each dosing date is collected on the CRF and creatinine clearance derived from the CRF data and capped at 125 mL/min
Cumulative Dose	*Cum dose (mg/m²) is* sum of the doses (mg/m²) administered to a subject.	*Cum dose (AUC) is* sum of the doses (AUC) administered to a subject.
Relative dose intensity (%)	Cum dose (mg/m²)/[(Last paclitaxel dose date - paclitaxel Start dose date + 21) x 200/21] x 100 or Cum dose (mg/m²)/[(Last paclitaxel dose date - paclitaxel Start dose date + 21) x 175/21] x 100	Cum dose (AUC)/[(Last dose date of Carbo - Start dose date of Carbo + 21) x 6/21] x 100 or Cum dose (AUC)/[(Last dose date of Carbo - Start dose date of Carbo + 21) x 5/21] x 100

Where the creatinine clearance will be calculated using Cockroft-Gault formula, defined as:

$$\text{CrCL(ml/min)} = \frac{(140 - \text{age(in years)}) * \text{weight(in kg)}}{72 * \text{serum creatinine(in mg/dL)}}$$

for males and

$$CrCL(ml/min) = \frac{(140 - age(in\ years)) * weight(in\ kg)}{72 * serum\ creatinine(in\ mg/dL)} * 0.85$$

for females. The most recent weight will be used. If the computed creatinine clearance is more than 125 ml/min, then the creatinine clearance value should be capped at 125ml/min for dose exposure computations.

7.4.2 Modifications of Study Therapy

7.4.2.1 Dose Delay/Omission

Each study medication infusion may be delayed. A dose will be considered as actually delayed if the delay is exceeding 3 days (i.e., greater than or equal to 4 days from scheduled dosing date) for study medication. In case of reported omission of a dose between 2 doses, this will be taken into account in the derivation and will not count as a delay unless there is a delay in addition to the omission.Reason for dose delay/omission will be retrieved from CRF dosing pages.

The following parameters will be summarized by treatment group and by drug.

- Number of subjects with at least one dose delayed, the number of dose delays per subject, the reason for dose delay (including covid related reason) and the length of dose delay.
- Number of subjects with reported dose omission, reason for omission.
- The listing of dosing will include the dose modifications, including dose modifications for adjuvant systemic therapy. Dose modifications for adjuvant therapy will only include the reported modifications, there will be no derivation.

7.4.2.2 Infusion Interruptions and Rate Changes

Each study drug infusion can be interrupted and/or the IV infusion rate can be reduced. This information will be retrieved from CRF dosing pages

The following parameters will be summarized by treatment group and study drug:

- Number of subjects with at least one dose infusion interruption, the reason for interruption, and the number of infusion interruptions per subject.
- Number of subjects with at least one IV infusion rate reduction, the reason for reduction and the number of infusion with IV rate reduction per subject.

7.4.2.3 Dose Reductions

There will be no dose reductions of nivolumab and ipilimumab allowed. Dose of platinum doublet chemotherapy (Arms B and C) may be modified for toxicity. Dose levels of platinum doublet chemotherapy (Arms B and C) are defined in the protocol as follows:

Table 7.4.2.3-1: Dose Modifications of Chemotherapeutic Agents (Arms B and C)

Dose Level	Vinorelbine	Docetaxel	Gemcitabine	Pemetrexed	Cisplatin	Carboplatin	Paclitaxel
Starting dose	25 mg/m^2 or 30 mg/m^2	60 mg/m^2 or 75 mg/m^2	1000 mg/m^2 or 1250 mg/m^2	500 mg/m^2	75 mg/m^2	AUC 5 or 6	175 or 200 mg/m^2
First dose reduction	75% of strating dose	75% of starting dose	75% of starting dose	75% of starting dose	75% of starting dose	AUC 4 or 5	150 mg/m^2
Second dose reduction	50% of starting dose	50% of starting dose	50% of starting dose	50% of starting dose	50% of starting dose	AUC 3 or 4	100 mg/m^2
Third dose reduction	Discontinu e	Discontinu e	Discontinu e	Discontinu e	Discontinu e	Discontinu e	Discontinu e

For any cycle, it will be defined as a dose reduction if the observed dose level (based on calculated administered dose) is below protocol specified dose level. Dose ranges for dose levels of platinum doublet chemotherapy are defined in Table 7.4.2.3-2.

Table 7.4.2.3-2: Calculated Dose Ranges and Related Dose Levels

Dose Level	Dose Range						
	Vinorelbine (mg/m^2)	Docetaxel (mg/m^2)	Gemcitabine (mg/m^2)	Pemetrexed (mg/m^2)	Cisplatin (mg/m^2)	Carboplatin (AUC)	Paclitaxel (mg/m^2)
Level 0	≥21.875 or ≥26.25	≥ 52.5 or ≥65.625	≥875 or ≥1093.75	≥437.5	≥65.625	≥4.5 or ≥5.5	≥153.125 or ≥175
Level -1	<21,875 and ≥ 15.625 or <26.25 and ≥ 18.75	<52.5 and ≥37.5 or < 65.625 and ≥46.875	<875 and ≥625 or <1093.75 and ≥781.25	<437.5 and ≥312.5	<65.625 and ≥46.875	<5.5 and ≥4.5 or <4.5 and ≥3.5	<175 and ≥125
Level -2	<15.625 or <18.75	<37.5 or < 46.875	<625 or < 781.25	<312.5	<46.875	<4.5 or <3.5	<125

The reason for dose reduction as reported by the investigator will be tabulated for all instances of dose reduction based on the Dose Change CRF page. A category 'Unknown' will be defined for all reductions with no reason reported by the investigator.

Chemotherapy dose reductions are permanent; once the dose of any chemotherapy agent is reduced, it may not be re-escalated in subsequent cycles.

The following will be summarized for chemotherapeutic agent arm only:

Number and percentage of subjects with at least one dose reduction and reason of the dose reduction, number and percentage of subjects with a dose reduction to dose level -1, number and percentage of subjects with a dose reduction to dose level -2.

7.4.3 Concomitant Medications

Concomitant medications, defined as medications other than study medications which are taken at any time on-treatment (i.e. on or after the first day of study therapy and within 100 days following the last dose of neoadjuvant study therapy or within 30 days following the last dose of adjuvant therapy, whichever is longest), will be coded using the WHO Drug Dictionary.

The following summary tables by treatment group will be provided:

- Concomitant medications (subjects with any concomitant medication, subjects by medication class and generic term).

Prior medications, defined as non-study medications with a start date before consent date, and current medications, defined as non-study medications with a start date before the first date of study medication and stop date after consent date, will be coded using the WHO Drug Dictionary.

The following summary table will be provided:

- Prior/current medications (subjects with any prior/current medication, subjects by medication class and generic term)

By-subject listings will accompany the tables.

7.4.3.1 Immune Modulating Medication

Immune modulating concomitant medications are medications entered on an immune modulating medication form or available from the most current pre-defined list of immune modulating medications. The list of anatomic class, therapeutic class and generic name used for the selection at the time of the database lock will be provided.

The percentage of subjects who received immune modulating concomitant medication for

- management of adverse event
- premedication
- other use
- any use
- management of drug-related select adverse event (any grade, grade 3-5) by select AE category/ subcategory
- management of IMAEs (any grade, grade 3-5) by IMAE category

will be reported separately for each treatment group (percentages of treated subjects by medication class and generic term).

For each category/subcategory of drug-related select AEs (any grade, grade 3-5) and IMAEs (any grade, grade 3-5), the following will be reported for each treatment group:

- The total immune modulating medication treatment duration (excluding overlaps), duration of high dose of corticosteroid, initial dose of corticosteroid, and tapering duration (summary statistics)

Duration represents the total duration the subject received the concomitant medication of interest. If the subject took the medication periodically, then DURATION in the summation of all use. Initial dose represents the dose of the concomitant medication of interest received at the start of the event. In the case multiple medications started on the same date, the highest equivalent dose is chosen and converted to mg/kg by dividing by the subject's recent weight.

These analyses, except the ones related to IMAEs will be conducted using the 30-day safety window. The analyses related to IMAEs will be conducted using the 100-day safety window.

7.4.3.2 Subsequent Cancer Therapy

Subsequent therapies are defined as Cancer therapies started on or after the first study drug dose or date of randomization if the subject is no treated, outside of the protocol defined adjuvant therapy (systemic and radiotherapy).

The following information pertaining to subsequent therapies will be summarized by treatment arm, as randomized:

- Number and percentage of subjects receiving subsequent therapies including:
- Subsequent systemic therapy by drug name
- Subsequent disease related surgery
- Subsequent radiotherapy for treatment of tumors

A by-subject listing of subsequent cancer therapy will also be produced for randomized subjects.

7.5 Definitive Surgery

Unless otherwise specified, analyses will be performed by treatment group as randomized for all concurrently randomized participants from Arms B and C. Descriptive analyses will also be produced for Arm A. Participants in Arm B randomized in the initial protocol will only be reported in listings.

The following parameters will be summarized:

- Disease Stage Prior to Surgery
- Subjects with clinical downstaging (lower stage prior to surgery vs baseline)
- Subjects with surgery
-

Subjects without surgery:

- Reason for cancelled surgery

Subjects with surgery:

- Delayed surgery (>6 weeks post last neoadjuvant dose), reason for delay as reported in CRF

- Duration of delay (number of weeks between last neoadjuvant dose and surgery date exceeding 6 weeks), descriptive statistics and categories: 1-2 weeks, 3-4 weeks, 5-6 weeks, >6 weeks delay)
- Duration of surgery
- Length of hospitalization for definitive surgery
- Method of surgery (Minimally invasive-thoracoscopic/robotic, Thoracotomy, Minimally innvasive to thoracotomy)
- Type of Surgery (Pneumonectomy, Lobectomy, Sleeve Lobectomy, Bilobectomy, Other)
- Surgery Outcome (R0, R1, R2, unknown)

Safety related to surgery analyses are described in section 7.7.1

7.6 Efficacy

Unless otherwise specified, analyses will be performed by treatment group as randomized for all concurrently randomized participants from Arms B and C. Descriptive analyses will also be produced for Arm A. Participants in Arm B randomized in the initial protocol will only be reported in listings and in analyses based on the All Concurrently Randomized Participants in Arms A and B population.

Unless stated otherwise, whenever a stratified analysis is specified, the following stratifications factors (recorded at randomization as per IRT) will be used:

- PD-L1 expression (≥1% or <1%/not evaluable/indeterminate)
- Disease stage (IB/II vs IIIA)
- Gender

Alpha (α) for the confidence intervals (CIs) for hazard ratios, odds ratios or difference of rates will be the same as nominal significance level for hypothesis testing. CIs for endpoints not tested will be at the two-sided 95% level. All p-values reported will be two-sided. P-values will be rounded to the fourth decimal place. Point estimates and confidence bounds for efficacy variables will be rounded to the second decimal place.

7.6.1 Type I Error Control

The overall alpha will be controlled using the following procedure. The overall alpha is primarily allocated to the two primary endpoints: 1% for pCR and 4% for EFS.

- The primary endpoint pCR will be tested at 1% alpha.
- If pCR is not significant, the primary endpoint EFS will be tested at 4%
- If pCR is significant, the 1% alpha will be re-allocated to the EFS primary endpoint which will be tested at 5% alpha level
- If EFS is significant, OS will be tested at the same level as EFS

EFS and OS (if EFS is significant) will be tested at planned interim and final analyses. Stopping boundaries will be calculated for each endpoint according to the observed number of events by Lan-DeMets alpha spending function with O'Brien-Fleming boundaries corresponding to an overall alpha of 4% or 5%. Given EFS and OS endpoints are tested using group sequential

approach, overall hierarchical testing approach will be used where each endpoint will have its own specific Lan-DeMets alpha spending function with O'Brien-Fleming boundaries[8]. Also refer to Sections 5.2 and 5.3.

If the p-value crosses the boundary at the interim analysis (EFS or OS), the p-value from the interim stratified log-rank test will be considered the final analysis result for the study.

The secondary endpoints of Major Pathologic Response and Time to Death or Distant Metastases will be analyzed descriptively without hypothesis testing.

7.6.2 Analysis of Pathological Complete Response

7.6.2.1 Primary pCR Analysis

Formal analysis of pCR will occur after the 350 randomized participants in arms B and C from start of 1:1:1 randomization have an opportunity for surgery.

At pCR analysis, the primary analysis population is the concurrently randomization participants in arms B and C. PCR rate will be computed in each treatment group along with the exact 95% CI using Clopper-Pearson method.

The numerator is based on randomized participants achieving pCR in both tumor and lymph nodes, as assessed by independent pathological review (BIPR). The denominator is based on All Concurrently Randomized Participants in Arms B and C. Subjects who are no longer eligible for surgery, or who are on alternative anti-cancer therapy before surgery, or who discontinue before surgery or for whom pCR results are not available are all counted as non-responders.

pCR will be compared between concurrent arms B and C by the stratified Cochran Mantel-Haenszel (CMH) test using a 2-sided, 1% alpha level.

An estimate of the difference in pCR rates between the treatment groups along with the corresponding two sided 99% CI will also be computed using the following Cochran-Mantel-Haenszel (CMH) method of weighting, adjusting for stratification factors[14]. A two sided 99% CI for odds ratio of pCR between the treatment groups will also be computed.

Estimate of the difference in pCR between arms A and B (in the All Concurrently Randomized Participants in Arms A and B population) and odds ratio will also be provided together with corresponding 95% CI using the same methodology.

The analysis will be conducted by an independant statistician external to BMS and reviewed by the DMC. At the time of pCR analysis, EFS descriptive analyses (by investigator and by BICR) will be produced in the DMC closed report and might be shared with regulatory authorities. The communication of results will be tightly controlled and pre-specified in the DMC charter to maintain trial integrity.

7.6.2.2 Supportive Analyses of pCR

pCR sensitivity analyses will be performed with the following consideration:

- pCR analysis will be repeated for response evaluable subjects, where response evaluable subjects are subjects who had definitive surgery, and didn't start alternative anti-cancer therapy

before surgery and pathologic samples results at surgery are evaluable. No p-value will be generated.

- pCR using stratification factors as obtained from the baseline CRF pages or database (instead of IRT). This analysis will be performed only if the stratification variable/factor at randomization (as per IRT) and baseline are discordant for at least 10% of randomized subjects. Stratified Cochran Mantel-Haenszel (CMH) p-value will be generated.
- Considering the small number of subjects that may be impacted by the Covid-19 situation in terms of pathology assessment, no sensitivity analysis is currently planned. The potential surgeries delays or cancellation due to Covid-19 related issues will be reported based on the surgery listing.
-

7.6.2.3 Subset Analyses of pCR

The influence of baseline and demographic characteristics on the treatment effect will be explored via exploratory subset analysis. BIPR assessment of pCR will be summarized for the following subgroups:

- Age category
    a) <65,
    b) >=65 and <75
    c) >=75 and <85
    d) >=85
    e) >=75
    f) >=65
- Sex (male, female), per IRT and per CRF
- Race (white, black, Asian, other)
- Region (North America, Europe, Asia, Rest of World)
- Baseline ECOG Performance Status (0, 1, >1)
- Tobacco use (current/former, never smoked, unknown)
- Disease stage (IB/II vs IIIA) per IRT and per CRF
- Baseline histology (squamous, non-squamous)
- PD-L1 subgroups (<1%, ≥1%, 1-49%, ≥50%, indeterminate, not evaluable)
- Tumor Tissue TMB Evaluable (≥12.3 mut/MB, < 12.3 mut/MB, Overall)
- Tumor Tissue TMB Not Evaluable
- Type of platinum therapy (cisplatin, carboplatin, subjects switching from cisplatin to carboplatin).
- Type of chemotherapy regimen in arm B (available in arm C (Gemcitabine-Cisplatin, Pemetrexed-Cisplatin, Paclitaxel-Carboplatin, not available in arm C (Vinorelbine-Cisplatin, Docetaxel-Cisplatin)) , based on first neoadjuvant cycle.

A forest plot of treatment effect on pCR per BIPR in the above subgroups will be produced. The un-weighted differences in pCR between concurrent arms B and C and corresponding 95% two-sided CI using the method of Newcombe, will be provided.

The analysis comparing treatment (i.e., pCR difference) will be conducted if the number of subjects in the subgroup category is more than 10.

7.6.2.4 Major Pathological Response Rate

MPR rate in concurrently randomized participants in arms B and C will be computed in each treatment group along with the exact 95% CI using Clopper-Pearson method. An estimate of the difference and odds ratio in MPR rates between concurrent arms B and C and corresponding 95% CI will be calculated using CMH methodology and adjusted by stratification factors.

Estimate of the difference in MPR between arms A and B (in the All Concurrently Randomized Participants in Arms A and B population) and odds ratio will also be provided together with corresponding 95% CI.

Subset analyses by PDL1 status will be performed (PD-L1<1% PD-L1 ≥ 1%, PD-L1 1-49%, PD-L1 ≥ 50%, not evaluable/indeterminate) and by Tumor TMB (≥12.3 mut/MB, < 12.3 mut/MB, Overall, not evaluable).

7.6.2.5 Additional Pathological Related Analyses

Descriptive analyses of pCR and MPR, % tumor area with viable tumor cells in the tumor region and in lymph nodes separately will be provided.

7.6.2.6 Clinical Response Rate

Clinical response rate (cRR) by BICR will be summarized by treatment arm. cRR is defined as proportion of randomized participants whose radiologic response at the last scan prior to definitive surgery is either a complete response or partial response per RECIST 1.1 criteria by BICR. The response does not require confirmation. Response rates and their corresponding 95% exact CI will be calculated by Clopper-Pearson method presented for each randomized arm.

Clinical response rate by investigator will be reported similarly.

Subset analyses of cRR by BICR by PDL1 status will be performed (PD-L1<1% PD-L1 ≥ 1%, PD-L1 1-49%, PD-L1 ≥ 50%, not evaluable/indeterminate) and by Tumor TMB (≥12.3 mut/MB, < 12.3 mut/MB, Overall, not evaluable).

7.6.3 Analysis of Event Free Survival

7.6.3.1 Primary Event Free Survival

One of the primary objectives of the study is to compare the event-free survival (based on BICR assessments) between treatment groups in all concurrently randomized participants in Arms B and C.

The primary definition of EFS, censoring for subsequent anticancer therapy, will be used in this analysis.

EFS will be compared between the treatment groups (concurrent B and C) at the interim and final analyses, using stratified log-rank test, with stratification factors as per IRT, two-sided p-value will also be reported. A Lan DeMets α-spending function with O'Brien and Fleming type of boundary will be employed to determine the nominal significance levels for the interim and final analyses. The stratified hazard ratio between the treatment groups will be presented along with 100*(1- α)% CI (adjusted for interim).

EFS will be estimated using the Kaplan Meier techniques and will be displayed graphically. A two-sided 95% CI for median EFS in each treatment group will be computed via the log-log transformation method. EFS rates at fixed time points (e.g. 6, 12 months, depending on the minimum follow-up) will be presented along with their associated 95% CIs. These estimates will be derived from the Kaplan Meier estimate and corresponding CIs will be derived based on Greenwood[15] formula for variance derivation and on log-log transformation applied on the survivor function[16].

These EFS analyses will also be conducted for Arm A, including Kaplan Meier curve, median and EFS rates with 95% CI and stratified HR between Arm A and Arm B (in the All Concurrently Randomized Participants in Arms A and B population) with 95% HR.

Analyses of EFS will also be conducted based on the secondary definition of EFS (not censoring for subsequent therapies). These analyses will be the same as those specified above.

The source of EFS event (progression precluding surgery, progression, recurrence (locoregional, distant) or death) will be summarized by treatment group. The status of subjects who are censored (as per primary definition of EFS) in the EFS KM analysis will be tabulated for each treatment group including the following categories:

- On-study (on-neoadjuvant treatment, on-adjuvant treatment, in follow-up)
- Off-study (lost to follow-up, withdraw consent, never treated)
- No baseline tumor assessment
- No on-study tumor assessment and no death
- Received subsequent anticancer therapy
- A by-subject listing will be presented including treatment group, EFS duration under the primary definition, EFS duration on the secondary definition, whether the subject was censored under the primary definition, and if censored, the reason, and whether the subject was censored under the secondary definition, and if censored, the reason.

A by-subject listing of lesion evaluations per BICR will be presented.

7.6.3.2 Supportive Analyses of Event-Free Survival

The following sensitivity analyses will be conducted in the concurrently randomized subjects in arms B and C for the primary definition. The p-values from sensitivity analyses for efficacy endpoints, are for descriptive purpose only and not adjusted for multiplicity.

- Delayed effect of immunotherapy interventions may cause a late separation in the OS KM curves and non-proportional hazards.

- EFS will be compared between treatment groups via a 2-sided max-combo test. The max-combo test statistic is the maximum of 4 diffrerent Fleming-Harrington family weighted log-rank test statistics. Zm = max (FH (0, 0), FH (0,1), FH (1,0), F(1,1)), where $FH(\rho,\gamma)$ are the test statistics from the Fleming-Harrington family of test statistics. FH (0, 0) corresponds to the log-rank test, while FH (0, 1) is more sensitive to late-difference alternatives, FH(1,0) is more sensitive to early difference with decreasing treatment effect and FH(1,1) uses weigths at the median.
- To examine the assumption of proportional hazards in the Cox regression model, in addition to treatment, a time-dependent variable defined by treatment by time interaction will be added into the model. A two-sided Wald Chi-square p-value of less than 0.1 may indicate a potential non constant treatment effect. In such case, the following analysis will be conducted:
  - ♦ The estimates of the EFS hazard ratios will be estimated in 2 periods. The periods will be defined by a cut off point. The cut off point will be calculated using a stratified time-dependent Cox model with effects for treatment and period-by-treatment interaction. The cut off point will be estimated using a grid of possible cut off points and obtained by maximizing the partial log likelihood. Ties will be handled using the exact method. A two-sided 95% CI for the hazard ratio's will also be presented. Visual interpretation of the curves may lead to additional analyses with several cut off points.

- A multivariate Cox regression model will be used in order to estimate the treatment effect after adjustment for possible imbalances in known or potential prognostic factors. The factors used in the randomization, will be included in the model as stratification factors. However, all additional factors will be incorporated as covariates. The additional factors, which are all measured at baseline, will include:
  - Histology (Squamous, Non-squamous)
  - Age categorization (< 65, ≥ 65)
  - ECOG (0, ≥ 1)
  - Race (White, Black, Asian, Other)
- The level of the covariate normally associated with the worst prognosis will be coded as the reference level. The hazard ratio associated with treatment and with each of the baseline covariates will be presented along with associated 95% CIs and p-value.
- The primary EFS based on BICR assessments analysis will be repeated using secondary EFS definition which accounts for the tumor scans post subsequent therapies for the primary efficacy population. Stratified log-rank test p-value will be generated.
- EFS based on BICR assessments, using stratification factors as obtained from the baseline CRF pages or database (instead of IRT). This analysis will be performed only if the stratification variable/factor at randomization (as per IRT) and baseline are discordant for at least 10% of randomized subjects. Stratified log-rank test p-value will be generated.
- EFS based on BICR assessments accounting for missing tumor assessment prior to EFS event (progression/recurrence or death). This analysis will be performed only if at least 10% of events have missing prior tumor assessment within the primary efficacy population. It will apply the following restriction to the primary definition: If the elapsed time between the EFS event and the last assessment immediately prior to the event is two or more missed visits, the

subject's EFS will be censored at his/her last tumor assessment prior to the EFS event. Stratified log-rank test p-value will be generated. This analyses may account for potential missed assessments due to the Covid-19 situation.

- EFS based on BICR assessments accounting for site reported pathology results. In case of site reported pathology recurrence on the CRF at an earlier date than the BICR event date, an event will be assigned at the pathology site reported date. No p-value will be generated.
- EFS based on BICR assessments accounting for BICR assessed progressions occuring before surgery but not precluding surgery. This analysis will use the primary definition of EFS but will in addition count an event for BICR assessed RECIST 1.1 progression before surgery, that would not preclude surgery.
- EFS based on investigator assessments. The hazard ratio associated with treatment and median EFS will be presented along with the associated two-sided 95% CIs. Kaplan-Meier plot will be produced. It is to be noted that per CRF instruction, the investigator will not consider a second primary cancer as a recurrence/progression. While if such lesions are present on tumor assessment at the BICR, they will be considered as new lesions, since the BICR does not have access to biopsy results. EFS by investigator is defined the same way as for EFS by BICR, except that both pathology and imaging recurrences reported in the CRF are taken into account as event and censoring will occur at the time of last tumor assessment prior to (or at the date of) second primary cancer. No p-value will be generated.
- EFS based on BICR assessments using an un-stratified Cox model. Un-stratified log-rank test p-value will be generated
- EFS in treated subjects from concurrently randomized arms B and C using treatment group "as treated" if more than 10% randomized subjects in any treatment group were never treated or treated differently than randomized among corresponding analysis population. Stratified log-rank test p-value will be generated.
- EFS analysis for participants with no relevant deviation. This analysis will be conducted only if there are more than 10% participants with relevant protocol deviations. Stratified log-rank test p-value will be generated.
- In order to assess the potential impact of the change in tumor assessment scheduled on the longer term and potential missing assessments, EFS (by BICR) will also be analyzed based on interval censoring method. The SAS PROC ICPHREG will be used to fit the proportional hazards regression models using interval censoring approach, with treatment arm as the only covariate in the model. Hazard ratio with 2-sided 95% and 100-alpha confidence intervals will be produced. The time period between time1 and time2 will be the interval during which the EFS event occurred. Time1 and time2 will be setup as follows:
  - For subjects who had EFS event per EFS primary definition, time1 is the time from randomization date to the last tumor assessment date prior to EFS event date, and time2 is time to EFS event (EFS duration).
  - For subjects who are censored for EFS, time1 is time to EFS event (EFS duration), and time2 is infinite time (missing in SAS).
- In order to assess the potential variability introduced by the optional adjuvant chemotherapy:
  - Baseline demographics and disease characterictics, characteristics at the time of surgery will be tabulated by adjuvant therapy status.

- A Cox regression model with treatment and an additional time-dependant covariate as indicator of the start of adjuvant therapy will used.
- Additional sensitivity analyses may be performed

- Given the small number of subjects expected to be impacted by a death due to Covid-19 infection, no specifc sensitivity analysis is currently planned. Subjects with death (potentially) associated with Covid-19 infection will be reported based on the reason for death in the death listing.

7.6.3.3 Subset Analyses of EFS

The influence of baseline and demographic characteristics on the treatment effect will be explored via subset analyses for the factors specified in section 7.6.2.3.

A forest plot of the EFS based on BICR assessments, unstratified hazard ratios (HR) along with two-sided 95% CIs will be produced for each level of the subgroups listed in section 7.6.2.3. If subset category has less than 10 subjects per treatment group, HR will not be computed/displayed. Median and 95% CI will be provided.

In addition, for gender, baseline disease stage, histology, PD-L1 and tumor TMB subsets, Kaplan Meier Curves will be generated.

Kaplan Meier curves, medians and 95% confidence interval will be generated for EFS based on BICR assessments by PD-L1 (≥ 1%, <1%, 1%-49%, ≥ 50%) for Arm A.

7.6.3.4 EFS Analyses by pCR and MPR Status

EFS (based on BICR assessments, primary definition) Kaplan-Meier curves will be generated by pCR and by MPR status. These analyses will be landmarked at the time of surgery and will be limited to subjects with pCR or MPR status available. Median and 95% CI will be provided. HR and 95% CI for concurrently randomized subjects in arms B and C will be provided by pCR and by MPR status, as well as HR of pCR/MPR vs no pCR/MPR by treatment arm. If subset category has less than 10 subjects per treatment group, HR will not be computed/displayed.

In addition, EFS (based on BICR assessments, primary definition) Kaplan-Meier curves will be generated by pCR and by MPR status, without landmark. These analyses will include all randomized subjects concurrently ranomdized to Arms B and C. Median and 95% CI will be provided. HR and 95% CI for concurrently randomized subjects in arms B and C will be provided by pCR and by MPR status, as well as HR of pCR/MPR vs no pCR/MPR by treatment arm. If subset category has less than 10 subjects per treatment group, HR will not be computed/displayed.

7.6.3.5 Current Status of EFS

Time from last censoring point to cutoff date in months will be summarized by treatment group and overall for randomized subjects. Subjects who have a EFS event will be considered as current for this analysis. The secondary definition of EFS (by BICR) will be used for this summary.

7.6.4 Analysis of Overall Survival

7.6.4.1 OS Analyses

OS will be hierarchically tested if EFS is significant. Details are provided in section 5.3.

OS will be compared between the treatment groups (concurrent B and C) at the interim and final analyses, using stratified log-rank test, with stratification factors as per IRT, two-sided p-value will also be reported. A Lan DeMets α-spending function with O'Brien and Fleming type of boundary will be employed to determine the nominal significance levels for the interim and final analyses. The stratified hazard ratio between the treatment groups will be presented along with 100*(1- α)% CI (adjusted for interim).

OS will be estimated using the Kaplan Meier techniques and will be displayed graphically. A two-sided 95% CI for median EFS in each treatment group will be computed via the log-log transformation method. EFS rates at fixed time points (e.g. 6, 12 months, depending on the minimum follow-up) will be presented along with their associated 95% CIs. These estimates will be derived from the Kaplan Meier estimate and corresponding CIs will be derived based on Greenwood formula for variance derivation and on log-log transformation applied on the survivor function.

These analyses OS analyses will also be conducted for Arm A, including Kaplan Meier curve, median and OS rates with 95% CI and stratified HR between Arm A and Arm B (in the All Concurrently Randomized Participants in Arms A and B population) with 95% HR.

The number of participants who are censored in the OS KM analysis and their status will be tabulated using following categories:

- Still on treatment (No recurrence/progression, recurrence/progression)
- In follow-up
- Off study
  - lost to follow-up
  - participant withdrew consent
  - other

7.6.4.2 Supportive Analyses for OS

The following sensitivity analyses will be conducted in the concurrently randomized subjects in arms B and C. The p-values from sensitivity analyses for efficacy endpoints, if presented, are for descriptive purpose only and not adjusted for multiplicity.

- A multivariate Cox regression model will be used in order to estimate the treatment effect after adjustment for possible imbalances in known or potential prognostic factors. The factors used in the randomization, will be included in the model as stratification factors. However, all additional factors will be incorporated as covariates. The additional factors, which are all measured at baseline, will include:
  - Histology (Squamous, Non-squamous)
  - Age categorization (< 65, ≥ 65)
  - ECOG (0, ≥ 1)
  - Race (White, Black, Asian, Other)

The level of the covariate normally associated with the worst prognosis will be coded as the reference level. The hazard ratio associated with treatment and with each of the baseline covariates will be presented along with associated 95% CIs and p-value.

- OS analysis using stratification factors as obtained from the baseline CRF pages or database (instead of IRT). This analysis will be performed only if the stratification variable/factor at randomization (as per IRT) and baseline are discordant for at least 10% of randomized subjects. Stratified log-rank test p-value will be generated.
- OS analysis using an un-stratified Cox model. Unstratified log-rank test p-value will be generated.
- OS analysis for participants with no relevant deviation. This analysis will be conducted only if there are more than 10% participants with relevant protocol deviations. Stratified log-rank test p-value will be generated.
- OS in treated subjects from concurrently randomized arms B and C using treatment group “as treated” if more than 10% randomized subjects in any treatment group were never treated or treated differently than randomized among corresponding analysis population. Stratified log-rank test p-value will be generated.
- In order to assess the potential variability introduced by the optional adjuvant chemotherapy:
  - Baseline demographics and disease characteristics, characteristics at the time of surgery will be tabulated by adjuvant therapy status.
  - A Cox regression model with treatment and an additional time-dependant covariate as indicator of the start of adjuvant therapy will used.
  - Additional sensitivity analyses may be performed
- Given the small number of subjects expected to be impacted by a death due to Covid-19 infection, no specifc sensitivity analysis is currently planned. Subjects with death (potentially) associated with Covid-19 infection will be reported based on the reason for death in the death listing.

7.6.4.3 Subset Analyses of OS

The influence of baseline and demographic characteristics on the treatment effect will be explored via subset analyses for the factors specified in section 7.6.2.3.

A forest plot of the OS unstratified hazard ratios (HR) along with two-sided 95% CIs will be produced for each level of the subgroups listed in section 7.6.2.3. If subset category has less than 10 subjects per treatment group, HR will not be computed/displayed. Median and 95% CI will be provided.

In addition, for gender, baseline disease stage, histology, PD-L1 and tumor TMB subsets, Kaplan Meier Curves will be generated.

Kaplan Meier curves, medians and 95% confidence interval will be generated for EFS based on BICR assessments by PD-L1 (≥ 1%, <1%, 1%-49%, ≥ 50%), for Arm A.

7.6.4.4 OS Landmark Analyses by pCR and MPR Status

OS Kaplan-Meier curves will be generated by pCR and by MPR status. These analyses will be landmarked at the time of surgery and will be limited to subjects with pCR or MPR status available. Median and 95% CI will be provided. HR and 95% CI for concurrently randomized subjects in arms B and C will be provided by pCR and by MPR status, as well as HR of pCR vs no pCR by treatment arm.

In addition, OS Kaplan-Meier curves will be generated by pCR and by MPR status, without landmark. These analyses will include all randomized subjects concurrently ranomdized to Arms B and C. Median and 95% CI will be provided. HR and 95% CI for concurrently randomized subjects in arms B and C will be provided by pCR and by MPR status, as well as HR of pCR/MPR vs no pCR/MPR by treatment arm.

If subset category has less than 10 subjects per treatment group, HR will not be computed/displayed.

7.6.4.5 Subject Follow-Up

The extent of follow-up defined as the time between randomization date and last known date alive (for subjects who are alive) or death date (for subjects who died). It will be summarized descriptively (median, min, max, etc) in months.

The currentness of follow-up for survival, defined as the time between last OS contact (i.e., last known date alive or death date) and cut-off date (defined by last patient last visit date), will be summarized in months by treatment group. Subjects who died and subjects with a Last Known Date Alive on or after data cut-off date will have a zero value for currentness of follow-up.

7.6.5 Interim Analyses of EFS and OS

An independent statistician external to BMS will perform the interim analyses. In addition to the formal planned interim analyses for EFS and OS, the Data Monitoring Committee (DMC) will have access to periodic un-blinded interim reports of efficacy and safety to allow a risk/benefit assessment. Details are included in the DMC charter.

Details of interim analyses timing and significance boundaries are provided in sections 5.2 and 5.3.

The DMC will review the safety and available efficacy data as planned in the DMC charter and will determine if the study should continue with or without changes or if accrual should be stopped. Subject enrollment will continue while waiting for the DMC's decisions.

The chair of the DMC and the sponsor can call an unscheduled review of the safety data.

At the time of the interim analysis for of EFS and OS, the DMC may recommend continuing or declare superiority. If the trial continues beyond the formal interim analyses, the nominal critical point for the final analysis will be determined using the recalculated information fraction at the time of the interim analysis, as described above. The final hazard ratio and corresponding confidence interval will be reported whereby the confidence interval will be adjusted accordingly (i.e. using the recalculated nominal α level at the final analysis).

If the EFS is significant but not OS, the trial is successful but will continue for further OS evalution.

If the p-value crosses the boundary at the interim analysis (EFS or OS), the p-value from the interim stratified log-rank test will be considered the final analysis result.

7.6.6 Analysis of TTDM

Time to death or distant matastases is a secondary endpoint.

TTDM, based on BICR assessments, will be estimated using the Kaplan Meier techniques and will be displayed graphically. A two-sided 95% CI for median TTDM in each treatment group will be computed via the log-log transformation method. TTDM rates at fixed time points (e.g. 6, 12 months, depending on the minimum follow-up) will be presented along with their associated 95% CIs. These estimates will be derived from the Kaplan Meier estimate and corresponding CIs will be derived based on Greenwood formula for variance derivation and on log-log transformation applied on the survivor function.

7.6.7 Event Free Survival on Next Line of Therapy

Event Free survival on next line of therapy is an exploratory endpoint.

Event free survival will be estimated using the Kaplan Meier techniques and will be displayed graphically. A two-sided 95% CI for median in each treatment group will be computed via the log-log transformation method. Events rates at fixed time points (e.g. 6, 12 months, depending on the minimum follow-up) will be presented along with their associated 95% CIs. These estimates will be derived from the Kaplan Meier estimate and corresponding CIs will be derived based on Greenwood formula for variance derivation and on log-log transformation applied on the survivor function.

7.6.8 Consistency of Efficacy by Randomization Period

Consistency of population amongs the different randomization periods will be examined by summarizing the primary endpoints by treatment arm as randomized in the the different randomization period: before revised protocol 02 (1:1 A vs B), under revised protocol 02 (1:1:1 A vs B vs C) and after revised protocol 03 (1:1 B vs C). Descriptive statistics will be provided including pCR rates, EFS medians, 95% CI and Kaplan-Meier plots. HR and 95% CI will be provided for arms C vs B during the 1:1:1 randomization (under revised protocol 02) and during the 1:1 randomization (under revised protocol 03).

Concurrent randomization is considered at the site level basis, when the site switched to the revised protcol. In practice, this includes subjects randomized on the randomization lists from the 1:1:1 randomization (revised protocol 02) and the subsequent 1:1 randomization between B and C only (revised protocol 03).

7.7 Safety

Analyses in this section will be tabulated for all treated subjects by treatment group as treated, unless otherwise specified.

Analyses will be performed on the treated subjects from arm A and from the concurrently randomized arms B and C. Participants in Arm B randomized in the initial protocol will only be reported in listings.

7.7.1 Definitive Surgery Related Safety

Incidence of AE/SAE indicated as surgical complication in the CRF, up to 90 days after surgery will be summarized by worst CTC grade, by treatment group.

Adverse events leading to cancellation of surgery and leading to surgery delay will be summarized by worst CTC grade, by treatment group.

7.7.2 Deaths

Deaths will be summarized by treatment group:

- All deaths, reasons for death.
- Deaths within 30 days of last neoadjuvant dose received (30-day safety window), reasons for death.
- Deaths within 100 days of last neoadjuvant dose received (100-day safety window), reasons for death.
- Deaths within 30 days and 90 days from surgery
- Overall summary of AEs leading to death within 100 days of last neoadjuvant dose received
- Overall summary of drug-related AEs leading to death within 100 days of last neoadjuvant dose received
- Overall summary of SAEs leading to death within 100 days of last dose received
- For subjects with systemic adjuvant therapy: deaths from start of systemic adjuvant to last systemic adjuvant dose received + 30 days

A by-subject listing of deaths will be provided for the all enrolled subjects population.

7.7.3 Serious Adverse Events

Serious adverse events will be summarized by treatment group:

- Overall summary of SAEs by worst CTC grade (any grade, grade 3-4, grade 5) presented by SOC/PT.
- Overall summary of drug-related SAEs by worst CTC grade (any grade, grade 3-4, grade 5) presented by SOC/PT.
- For subjects with systemic adjuvant treatment: Overall summary of SAEs by worst CTC grade (any grade, grade 3-4, grade 5) presented by SOC/PT from start of systemic adjuvant to last systemic adjuvant dose received + 30 days
- All analyses will be conducted using the 30-day safety window.

A by-subject SAE listing will be provided for the “enrolled subjects” population.

7.7.4 Adverse Events Leading to Discontinuation of Study Therapy

AEs are indicated as leading to discontinuation, when they lead to discontinuation of at least one agent of the regimen. Reporting is done based on AE CRF form.

AEs leading to discontinuation (of neoadjuvant treatment) will be summarized by treatment group:

- Overall summary of AEs leading to discontinuation by worst CTC grade (any grade, grade 3-4, grade 5) presented by SOC/PT.
- Overall summary of drug-related AEs leading to discontinuation by worst CTC grade (any grade, grade 3-4, grade 5) presented by SOC/PT.
- For subjects with systemic adjuvant treatment: Overall summary of AEs leading to discontinuation by worst CTC grade (any grade, grade 3-4, grade 5) presented by SOC/PT from start of systemic adjuvant to last systemic adjuvant dose received + 30 days

The analyses will be conducted using the 30-day safety window.

A by-subject AEs leading to discontinuation listing will be provided.

7.7.5 Adverse Events Leading to Dose Modification

AEs leading to dose delay/reduction will be summarized by treatment group:

- Overall summary of AEs leading to dose delay/reduction by worst CTC grade (any grade, grade 3-4, grade 5) presented by SOC/PT.
- Overall summary of related AEs leading to dose delay/reduction by worst CTC grade (any grade, grade 3-4, grade 5) presented by SOC/PT.

The analysis will be conducted using the 30-day safety window.

A by-subject AEs leading to dose delay/reduction listing will be provided.

7.7.6 Adverse Events

Adverse events will be summarized by treatment group.

The following analyses will be conducted using the 30 days safety window only:

- Overall summary of any AEs by worst CTC grade (1, 2, 3, 4, 5, not reported, total) presented by SOC/PT.
- Overall summary of any AEs presented by worst CTC grade (any grade, grade 3-4, grade 5) by SOC/PT. This table will be restricted to events with an incidence greater or equal to 5% in any treatment group.
- for subjects with systemic adjuvant: Overall summary of any AEs presented by worst CTC grade (any grade, grade 3-4, grade 5) by SOC/PT, from start of systemic adjuvant to last systemic adjuvant dose received + 30 days
- Overall summary of any non-serious AEs presented by SOC/PT. This table will be restricted to events with an incidence greater or equal to 5% in any treatment group.
- Overall summary of any AEs that required immune modulating medication by worst CTC grade (any grade, grade 3-4, grade 5) presented by SOC/PT.

Approved v3.0 [redacted]

- Overall summary of drug-related AEs by worst CTC grade (1, 2, 3, 4, 5, not reported, total) presented by SOC/PT.
- For subjects with systemic adjuvant treatment: Overall summary of Drug-Related AEs by worst CTC grade (any grade, grade 3-4, grade 5) presented by SOC/PT from start of systemic adjuvant to last systemic adjuvant dose received + 30 days

The following analyses will be conducted using the 30 days safety window and repeated using the 100 days safety window:

- Overall summary of drug-related AEs by worst CTC grade (any grade, grade 3-4, grade 5) presented by SOC/PT

A by-subject AE listing will be provided. A by-subject listing of any AE requiring immune modulating medications will also be provided.

7.7.7 Select Adverse Events

Unless otherwise specified, analyses will be performed by select AE category. Analyses will also be repeated by subcategory of endocrine events.

7.7.7.1 Incidence of Select AE

Select AEs will be summarized by treatment group for each category/subcategory.

The following analyses will be conducted using the 30-day safety window only:

- Overall summaries of any select AEs by worst CTC grade (any grade, grade 3-4, grade 5) presented by Category or Subcategory/PT.
- Overall summaries of any drug-related select AEs by worst CTC grade (any grade, grade 3-4, grade 5) presented by Category or Subcategory/PT.
- Overall summaries of any serious select AEs by worst CTC grade (any grade, grade 3-4, grade 5) presented by Category or Subcategory /PT.
- Overall summaries of drug-related serious select AEs by worst CTC grade (any grade, grade 3-4, grade 5) presented by Category or Subcategory /PT.
- Overall summaries of any select AEs leading to discontinuation by worst CTC grade (any grade, grade 3-4, grade 5) presented by Category or Subcategory /PT.
- Overall summaries of drug-related select AEs leading to discontinuation by worst CTC grade (any grade, grade 3-4, grade 5) presented by Category or Subcategory /PT.
- Summary of frequency of unique select AEs by Category.

A by-subject select AE listing will be provided.

7.7.7.2 Time-to Onset of Select AE

Time-to onset of drug-related select AEs (any grade, grade 3-5) will be summarized for each category/subcategory by treatment group.

Time-to onset analyses are restricted to treated subjects who experienced at least one drug-related select AE in the category/subcategory. The analyses will be conducted using the 30-day safety window.

Additional details regarding the time-to onset definition are described in time-to onset definition subsection of APPENDIX 1.

7.7.7.3 Time-to Resolution of Select AE

Time-to resolution of the following specific events will be summarized separately for each category/subcategory.

- Time-to resolution of drug-related select AE (any grade, grade 3-5) by treatment group
- Time-to resolution of drug-related select AE (any grade, grade 3-5) where immune modulating medication was initiated, by treatment group

Time-to resolution analyses are restricted to treated subjects who experienced the specific events. Time-to resolution where immune modulating medication was initiated analyses are restricted to treated subjects who experienced the specific events and who received immune modulating medication during the longest select AE.

The analyses will be conducted using the 30-day safety window.

The following summary statistics will be reported: percentage of subjects with resolution of the longest select AE, median time-to resolution along with 95% CI (derived from Kaplan-Meier estimation) and ranges.

See time-to resolution definition subsection of APPENDIX 1 for additional details.

7.7.8 Immune-Mediated Adverse Events

IMAEs will be summarized by treatment group for each immune-mediated category / PT using the 100-day safety window:

- Overall summary of non-endocrine IMAEs by worst CTC grade (any grade, grade 3-4, grade 5) where immune modulating medication was initiated presented by Category / PT.
- Overall summary of endocrine IMAEs by worst CTC grade (any grade, grade 3-4, grade 5) presented by Category / PT.
- Overall summary of non-endocrine IMAEs leading to discontinuation by worst CTC grade (any grade, grade 3-4, grade 5) where immune modulating medication was initiated presented by Category / PT.
- Overall summary of endocrine IMAEs leading to discontinuation by worst CTC grade (any grade, grade 3-4, grade 5) presented by Category / PT.
- Overall summary of non-endocrine IMAEs leading to dose delay or reduction by worst CTC grade (any grade, grade 3-4, grade 5) where immune modulating medication was initiated presented by Category / PT
- Overall summary of endocrine IMAEs leading to dose delay or reduction by worst CTC grade (any grade, grade 3-4, grade 5) presented by Category / PT.
- Summaries of time-to onset and time-to resolution of non-endocrine IMAEs where immune modulating medication was initiated presented by Category.
- Summaries of time-to onset and time-to resolution of endocrine IMAEs presented by Category.

Approved v3.0 [redacted]

A by-subject listing of IMAEs will be provided. By-subject listings of time-to resolution for longest IMAEs cluster (any grade and grade 3-5 in separate summaries) will also be provided. For new studies which collect investigator assessment of potential IMAE data, a by-subject listing of AEs considered as immune-mediated events per investigator but not qualified for IMAEs definition will also be provided.

7.7.9 Other Events of Special Interest

OEOSI will be summarized by treatment group for each category.

The following analyses will be conducted using the 100-day safety window:

- Overall summary of OEOSI by worst CTC grade (any grade, grade 3-4, grade 5) presented by Category / PT
- Overall summary of drug-related OEOSI by worst CTC grade (any grade, grade 3-4, grade 5) presented by Category / PT

A by-subject listing of OEOSI will be provided.

7.7.10 Multiple Events

The following summary tables will be provided:

- A table showing the total number and rate (exposure adjusted) of occurrences for all AEs.
- A table showing the total number and rate (exposure adjusted) of occurrences for AEs occurring in at least 5% of subjects in any treatment group.

Exposure adjustement will be based on the exposure in neoadjuvant treatment only.

A listing displaying the unique instances of all AEs, i.e., after duplicates have been eliminated and overlapping and contiguous occurrences of the same event (i.e. same PT) have been collapsed will be provided. No formal comparisons will be made between treatment groups.

7.7.11 New Primary Cancers

Occurence of new primary cancer reported on the new primary cancer CRF form will be listed based on the all treated population.

7.7.12 Laboratory Parameters

The analysis population for each laboratory test is restricted to treated subjects who underwent that laboratory test.

A by-subject listing of differences in categorization of SI and US laboratory test results will be provided.

7.7.12.1 Hematology

The following will be summarized by treatment group as worst CTC grade on-treatment per subject and as shift table of worst on-treatment CTC grade compared to baseline CTC grade per subject: hemoglobin (HB), platelets, white blood counts (WBC), absolute neutrophils count (ANC) and lymphocyte count (LYMPH).

Approved v3.0 [redacted]

The analyses will be conducted using the 30-day safety window.

A by-subject listing of these laboratory parameters will be provided.

7.7.12.2 Serum Chemistry

The following will be summarized by treatment group as worst CTC grade on-treatment per subject and as shift table of worst on-treatment CTC grade compared to baseline CTC grade per subject: ALT, AST, alkaline phosphatase (ALP), total bilirubin, creatinine, amylase, lipase.

The analyses will be conducted using the 30-day safety window.

A by-subject listing of these laboratory parameters will be provided.

7.7.12.3 Electrolytes

The following will be summarized by treatment group as worst CTC grade on-treatment per subject and as shift table of worst on-treatment CTC grade compared to baseline CTC grade per subject: sodium (high and low), potassium (high and low), calcium (high and low), magnesium (high and low), and Glucose Serum (fasting hyperglycemia and hypoglycemia regardless of fasting status).

The analyses will be conducted using the 30-day safety window.

A by-subject listing of these laboratory parameters will be provided.

7.7.12.4 Additional Analyses

In addition, further analyses on specific laboratory parameters will be performed by treatment group:

Abnormal Hepatic Function Test

The number of subjects with the following laboratory abnormalities from on-treatment evaluations will be summarized by treatment group:

- ALT or AST > 3 x ULN, > 5 x ULN, > 10 x ULN and > 20 x ULN
- Total bilirubin > 2 x ULN
- ALP > 1.5 x ULN
- Concurrent (within 1 day) ALT or AST > 3 x ULN and total bilirubin > 1.5 x ULN
- Concurrent (within 30 days) ALT or AST > 3 x ULN and total bilirubin > 1.5 x ULN
- Concurrent (within 1 day) ALT or AST > 3 x ULN and total bilirubin > 2 x ULN
- Concurrent (within 30 days) ALT or AST > 3 x ULN and total bilirubin > 2 x ULN

The analyses will be conducted using the 30-day safety window.

A by-subject listing of these specific abnormalities will be provided.

Abnormal Thyroid Function Test

The number of subjects with the following laboratory abnormalities from on-treatment evaluations will be summarized by treatment group:

- TSH value > ULN and
  - with baseline TSH value ≤ ULN
  - with at least one FT3/FT4 test value < LLN within 2-week window after the abnormal TSH test
  - with all FT3/FT4 test values ≥ LLN within 2-week window after the abnormal TSH test
  - with FT3/FT4 missing within 2-week window after the abnormal TSH test.

- TSH < LLN and
  - with baseline TSH value ≥ LLN
  - with at least one FT3/FT4 test value > ULN within 2-week window after the abnormal TSH test
  - with all FT3/FT4 test values ≤ ULN within 2-week window after the abnormal TSH test
  - with FT3/FT4 missing within 2-week window after the abnormal TSH test

The analyses will be conducted using the 30-day safety window.

A by-subject listing of these specific abnormalities will be provided.

7.7.13 Vital Signs and Pulse Oximetry

Vital signs and pulse oximetry (i.e. % oxygen saturation) collected on the CRF will be provided in separate listings.

7.7.14 Physical Measurements

Physical measurements will be listed by subject.

7.7.15 Non-Protocol Medical Procedures

Non-protocol medical procedures will be listed by subject.

7.7.16 Immunogenicity Analysis

Not applicable in this protocol.

7.7.17 Pregnancy

A by-subject listing of pregnancy tests results will be provided for randomized female subjects.

7.7.18 Adverse Events By Subgroup

Overall summary of any AEs and drug-related AEs by worst CTC grade (any grade, grade 3-4, grade 5) presented by SOC/PT and for each treatment group for the following subgroups:

- Sex (Male vs. Female)
- Race
- Age (< 65 vs. 65 - < 75 vs. 75 - < 85 vs. ≥ 85 vs. ≥ 75 vs. ≥ 65)
- Region (North America, Europe, Asia, Rest of World)
- Type of platinum therapy (cisplatin, carboplatin, subjects switching from cisplatin to carboplatin).

- Type of chemotherapy regimen in arm B (available in arm C (Gemcitabine-Cisplatin, Pemetrexed-Cisplatin, Paclitaxel-Carboplatin, not available in arm C (Vinorelbine-Cisplatin, Docetaxel-Cisplatin)) , based on first neoadjuvant cycle.

These analyses will be conducted using the 30-day safety window only.

7.7.19 Consistency of Safety by Randomization Period

Consistency of population amongs the different randomization periods will be examined by summarizing key safety by treatment arm as randomized in the the different randomization period: before revised protocol 02 (1:1 A vs B), under revised protocol 02 (1:1:1 A vs B vs C) and after revised protocol 03 (1:1 B vs C).

- Overall summary of any AEs presented by worst CTC grade (any grade, grade 3-4, grade 5) by SOC/PT with 30-day safety window.
- Overall summary of AEs leading to discontinuation (of neoadjuvant treatment) by worst CTC grade(any grade, grade 3-4, grade 5) presented by SOC/PT with 30-day safety window.
- Overall summary of non-endocrine IMAEs by worst CTC grade (any grade, grade 3-4, grade 5) where immune modulating medication was initiated presented by Category / PT with 100-day safety window.
- Overall summary of endocrine IMAEs by worst CTC grade (any grade, grade 3-4, grade 5) presented by Category / PT with 100-day safety window.
- Concurrent randomization is considered at the site level basis, when the site switched to the revised protcol. In practice, this includes subjects randomized on the randomization lists from the 1:1:1 randomization (revised protocol 02) and the subsequent 1:1 randomization between B and C only (revised protocol 03).

7.7.20 Covid-19

- COVID-19 related adverse events will be summarized (100 days window) and listed.

7.8 Pharmacokinetics

The nivolumab and ipilimumab concentration data obtained in this study will be combined with data from other studies in the clinical development program to develop a population PK model. This model will be used to evaluate the effects of intrinsic and extrinsic covariates on the PK of nivolumab and ipilimumab. In addition, exposure-response analyses with selected efficacy and safety endpoints will be conducted. Results of population PK and exposure response-analyses will be reported separately.

7.9 Biomarkers

Analyses for PD-L1 and TMB (tumor tissue) are described below.

Methodology for biomarkers other than PD-L1,Tumor TMB [redacted] will be reported separately.

7.9.1 PD-L1 Expression

<u>Descriptive statistics of PD-L1 expression</u>:

- Listing of all PD-L1 IHC data, all randomized subjects.

- Summary of tumor specimen acquisition and characteristics, all randomized subjects.
- Summary statistics of PD-L1 expression in all randomized subjects with quantifiable PD-L1 expression.
- Cumulative distribution plot of baseline PD-L1 expression versus population percentile in all randomized subjects with quantifiable PD-L1 expression.
- Box plots of PD-L1 expression versus pCR in all randomized subjects with quantifiable PD-L1 expression.
- Waterfall plot of Individual PD-L1 expression in all randomized subjects with quantifiable PD-L1 expression.
- Subgroups analyses of efficacy (pCR, MPR, EFS, OS) by PD-L1 status (PD-L1<1% PD-L1 ≥ 1%, PD-L1 1-49%, PD-L1 ≥ 50%, not evaluable/indeterminate) are described in Section 7.6.
- In addition the following analyses will be conducted in the concurrently randomized subjects in arms B and C.
- An exploratory Cox proportional hazards model, in order to assess the association of EFS (based on BICR assessments) with PD-L1, will be fitted for EFS with PD-L1, treatment arm and PD-L1* treatment arm interaction, among all PD-L1 evaluable subjects. An appropriate transformation of PD-L1 expression may be considered depending on an assessment of fit of the model. The following summaries will be presented by treatment arm
  - A plot of estimated $\log_e$(hazard ratio) with 95% confidence band vs PD-L1 expression(X-axis)
- An exploratory Cox proportional hazards model, in order to assess the association of OS with PD-L1, will be fitted for OS with PD-L1, treatment arm and PD-L1* treatment arm interaction, among all PD-L1 evaluable subjects. An appropriate transformation of PD-L1 expression may be considered depending on an assessment of fit of the model. The following summaries will be presented by treatment arm
  - A plot of estimated $\log_e$(hazard ratio) with 95% confidence band vs PD-L1 expression (X-axis).
- A logistic regression model will be fitted for pCR with PD-L1, treatment arm and PD-L1* treatment arm interaction, among all PD-L1 evaluable subjects. The following summaries will be reported:
  - A plot of estimated odds ratio with 95% confidence band vs PD-L1 expression (X-axis)
- For both EFS (using primary definition, per BICR) and OS, a Cox proportional hazards regression model will be fitted with treatment, PD-L1 status (using 1%, 5%, 50% cut offs), and treatment by PD-L1 status interaction. Although the study is not designed to have appropriate power to formally test the interaction of the model, an interaction test at significance level of 0.2 will warrant further exploration and the following statistics will be reported:
  - Interaction p-value
  - HR of treatment vs. control and its associated 95% CI for each of the PD-L1 status subgroup
  - HR PD-L1 ≥ cutoff vs. < cutoff and its associated 95% CI within each treatment group

7.9.2 Tumor Mutational Burden (TMB)

The analyses are based on tumor tissue TMB evaluable subjects, defined as subjects with tissue TMB data available. It is known that not all subjects will provide tumor tissue TMB data, due to factors such as available remaining tissue and inherent failure rates of the TMB process.

The descriptive analyses of tumor tissue TMB at baseline will be conducted:

- Listing of all tumor tissue TMB data.
- Summary of tumor specimen characteristics
- Cumulative distribution plot of TMB at baseline versus population percentile in all subjects with evaluable tumor tissue TMB.

In addition, the joint distribution of PD-L1 and tumor tissue TMB among both PD-L1 and tumor tissue TMB-evaluable subjects will be examined.

Subgroups analyses of efficacy (pCR, MPR, EFS, OS) by TMB status (≥12.3 mut/MB, < 12.3 mut/MB, TMB evaluable, TMB not evaluable) are described in Section 7.6.

- In addition the following analyses will be conducted in the concurrently randomized subjects in arms B and C.
- An exploratory Cox proportional hazards model, in order to assess the association of EFS (based on BICR assessments) with TMB, will be fitted for EFS with TMB, treatment arm and TMB* treatment arm interaction, among all TMB evaluable subjects. An appropriate transformation of TMB may be considered depending on an assessment of fit of the model. The following summaries will be presented by treatment arm
  - A plot of estimated $\log_e$(hazard ratio) with 95% confidence band vs PD-L1 expression(X-axis)
- An exploratory Cox proportional hazards model, in order to assess the association of OS with TMB, will be fitted for OS with TMB, treatment arm and TMB* treatment arm interaction, among all TMB evaluable subjects. An appropriate transformation of TMB may be considered depending on an assessment of fit of the model. The following summaries will be presented by treatment arm
  - A plot of estimated $\log_e$(hazard ratio) with 95% confidence band vs TMB (X-axis).
- A logistic regression model will be fitted for pCR with TMB, treatment arm and TMB* treatment arm interaction, among all TMB evaluable subjects. The following summaries will be reported:
  - A plot of estimated odds ratio with 95% confidence band vs TMB (X-axis)

[redacted]

[redacted]

[redacted]

7.10 Outcomes Research Analyses

The analysis of EQ-5D-3L will be restricted to randomized subjects who have an assessment at baseline and at least one post-baseline assessment.

The following descriptive analyses will be conducted:

- EQ-5D-3L questionnaire completion rate, defined as the proportion of questionnaires actually received out of the expected number (i.e. number of subjects on treatment or in follow up), will be calculated and summarized for each assessment time point by treatment group.
- A by-subject listing of the level of problems in each dimension, corresponding to EQ-5D-3L health state (i.e., 5-digit vector), EQ-5D-3L utility index score, and EQ-5D-3L VAS score will be provided.
- Proportion of subjects reporting problems for the 5 EQ-5D-3L dimensions at each assessment time point will be summarized by level of problem and by treatment group. Percentages will be based on number of subjects assessed at assessment time point.
- For the EQ-5D-3L utility index and VAS scores, separately:
  - Mean score and mean change from baseline at each assessment time point will be summarized by treatment group using descriptive statistics (N, mean with SD and 95% CI, median, first and third quartiles, minimum, maximum).
  - A line graph summarizing the mean changes from baseline will be produced.

7.11 Country Specific Analyses

Country or region specific analyses may take place to support regional health authorities submissions. In general these would consist of repeating a subset of the analyses described in this SAP for a subjects from a specific country or region, which would potentially be used for evaluation of consistency between the country/region and the overall population. Unless otherwise noted, no formal hypothesis testing will be performed to evaluate consistency of the subpopulation. Instead, descriptive statistics will be provided to assess the consistency.

These analyses provided for regional submission needs would only performed when at least one of the primary endpoints is statistically significant (based on data from the global population), for regional submission needs. The analysis methods for the subpopulation will be the same as for the global population unless otherwise noted.

For China, the the following analysis sets will be used:

China subpopulation

Chinese subpopulation is defined as Great China subgroup. It should contain subjects that are Chinese by race and enrolled from China, Hong-Kong or Taiwan sites.

Asian subpopulation

Asian subpopulation is defined as Asian subgroup. It should contain subjects that are Asian by race and enrolled from Asian countries, as per this SAP geographic region derivation.

8 CONVENTIONS

The following conventions may be used for imputing partial dates for analyses requiring dates:

- For missing and partial adverse event onset dates, imputation will be performed using the Adverse Event Domain Requirements Specification17
- For missing and partial adverse event resolution dates, imputation will be performed as follows:
  - If only the day of the month is missing, the last day of the month will be used to replace the missing day. If the imputed date is after the death date or the last known alive date, then the latest known alive date or death date is considered as the resolution date.
  - If the day and month are missing or a date is completely missing, it will be considered as missing.
- Missing and partial non-study medication domain dates will be imputed using the derivation algorithm described in 4.1.3 of BMS Non-Study Medication Domain Requirements Specification[18].
- Missing and partial radiotherapy and surgery dates will be imputed using algorithm described in APPENDIX 2.
- Missing of partial definitive surgery date
  - If only the day of the month is missing, the 1st of the month will be used to replace the missing day. In case of the date of death is present and complete, the imputed definitive surgery date will be compared to the date of death. The minimum of the imputed definitive surgery date and date of death will be considered as the date of definitive surgery.
  - If the day and month are missing or a date is completely missing, it will be considered as missing.
- For death dates, the following conventions will be used for imputing partial dates:
  - If only the day of the month is missing, the 1st of the month will be used to replace the missing day. The imputed date will be compared to the last known alive date and the maximum will be considered as the death date.
  - If the month or the year is missing, the death date will be imputed as the last known alive date.
  - If the date is completely missing but the reason for death is present, the death date will be imputed as the last known date alive.
- For date of progression/recurrence after start of study therapy, the following conventions will be used for imputing partial dates:
  - If only the day of the month is missing, the 1st of the month will be used to replace the missing day. In case of the date of death is present and complete, the imputed progression/recurrence date will be compared to the date of death. The minimum of the imputed progression/recurrence date and date of death will be considered as the date of progression/recurrence.

Approved v3.0 [redacted]

- If the day and month are missing or a date is completely missing, it will be considered as missing.

- For date of progression to prior therapies, the following conventions will be used for imputing partial dates:
  - If only the day of the month is missing, the 1st of the month will be used to replace the missing day.
  - If the day and month are missing or a date is completely missing, it will be considered as missing.
- For other partial/missing dates, the following conventions were used:
  - If only the day of the month is missing, the 15th of the month will be used to replace the missing day.
  - If both the day and the month are missing, "July 1" will be used to replace the missing information.
  - If a date is completely missing, it will be considered as missing.

The following conversion factors will be used to convert days to months or years:

1 month = 30.4375 days and 1 year = 365.25 days.

Duration (e.g. time-to onset, time-to resolution) will be calculated as follows:

Duration = (Last date - first date + 1)

Last known alive date will be defined based on all appropriate dates collected on the CRF.

All statistical analyses will be carried out using SAS (Statistical Analysis System software, SAS Institute, North Carolina, USA) unless otherwise noted.

9 CONTENT OF REPORTS

All analyses described in this SAP will be included in the Clinical Study Report(s) except where otherwise noted. Additional exploratory analyses may be performed. Refer to the Data Presentation Plan for mock-ups of all tables and listings.

10 DOCUMENT HISTORY

Table 10-1: Document History

Version Number	Author(s)	Description
1.0	[redacted]	Initial version dated 17-Apr-2020
2.0		29-Jul-2020<br>Revised Protocol 06 related changes:<br>• Clarified EFS definition

Table 10-1: Document History

Version Number	Author(s)	Description
		• Removed the 1st interim analysis of EFS and updated alpha spending of interim and final analyses of EFS, based on health authorities feedback<br>• Clarified that actual timing of analyses may differ from projected timing<br><br>Other changes:<br>• Removed immunogenicity analyses as immunogenicity is not described in the protocol.<br>• Sections 4.1.2 and 4.2.2, clarified that subjects with pCR/MPR not evaluable/no avaialble are considered as non-responders<br>• Section 4.3.3: specified that the length of surgery delay will be reported<br>[redacted]<br>• Section 4.3.7: EQ-5D analyses: clarify that the windowing for adjuvant is based on systemic adjuvant only<br>• Section 5.3: removed OS analyses methodology which is repeated in section 7<br>• Section 5.4: Refer to the DMC charter for handling of EFS descriptive analysis at the time of pCR<br>• Section 7.2.2: Relevant deviations: clarify that the concurrent therapies are limited to neoadjuvant and adjuvant systemic treatment<br>• Section 7.3.2: corrected the PDL1 should be per clinical database<br>• Section 7.3.4: removed reporting of prior systemic therapies, radiotherapy and surgeries since the study is in neoadjuvant setting<br>• Section 7.4.2.1: Dose delay/omission: describe the derivation of delay, when there is an omission between two doses. and the fact that delays during the adjuvant treatment are limited to reported delays on the CRF.<br>• Section 7.5: added duration of surgery delay<br>• Section 7.6: Added Covid-19 related consideration in the efficacy supportive analysis sections<br>• Section 7.6.2.2: added pCR sensitivity analysis using CRF stratification factors<br>• Section 7.6.3.2: added sensitivity analysis considering progressions before surgery as events, even if not precluding surgery<br>• Section 7.6.3.4 and 7.6.4.4: added calculation of HR for pCR vs no pCR.<br>• Section 7.6.4.2: remove extra paragraph on analysis on concurrently randomized arms B and C.<br>• Section 7.6.5: clarified that the DMC may declare superiority but not stop the trial for superiority as the trial will continue for further endpoints<br>• Section 7.7: clarified that the window for reporting AEs during the adjuvant period is based on systemic aduvant theratpy (not radiotherapy)<br>• Added section 7.11 for country/region specific analyses

Table 10-1: Document History

Version Number	Author(s)	Description
3.0	[redacted]	• The main reason for amendment is to reflect the changes to Revised Proocol 07: add an interim analysis for EFS/OS [redacted] The rationale for change is the potential for a slow down of EFS events with a plateau towards the end of the curve.<br>• [redacted] as well as some additional sensitivity, subgroup analyses and clarifications.<br>• Detailed changes are provided below:<br>• Section 1: removed reference to the Core SAP which is outdated<br>• Section 1: updated the schedule of analyses with an additional EFS/OS interim analysis at 90% EFS information fraction [redacted]<br>• Section 2.3: updates: In case of non-significant EFS at interim analysis, the DMC unblinded closed report will be shared with BMS executive restricted team. Further details on data communication are provided in section 2.3 and DMC charter.<br>• Section 2.4: updated the Revised Protocol 07 and added rationale for revised protocol 05 (in response to internal audit finding)<br>[redacted]<br>• Section 5.2: Updated with revised EFS assumptions assuming piecewise exponential distribution and added EFS interim analysis [redacted]<br>• Section 5.3: updated to add one OS interim analysis at the time of newly added EFS interim analysis<br>• Section 5.4: updated analyses timing following changes in section 5.2 and indicated that the the DMC closed report, including OS will be shared with the BMS restricted team. removed table 5.4-1 which was repeating information from Table 5.3-1 and section 5.4<br>• Section 7.3.1: clarified that covid-19 related reason will be provided<br>• Section 7.4.2: clarified that covid-19 related reason will be provided<br>• Section 7.6.3.2: Clarified that for EFS per investigator the censoring for new primary cancer is at the time of last tumor assessment prior or at the time of new primary cancer, instead of the date of new primary cancer<br>• Section 7.6.3.2: added sensitivity analysis with interval censoring method<br>• Section 7.6.3.3: added EFS analysis by PD-L1 for Arm A<br>• Section 7.6.3.4: clarified that no HR will be generated for subsets with less than 10 subjects, added HR by MPR<br>• Section 7.6.3.4: added EFS analysis by pCR and MPR without landmark to inform surrogacy

Table 10-1: Document History

Version Number	Author(s)	Description
		• Section 7.6.4.3: added OS analysis by PD-L1 for Arm A<br>• Section 7.6.4.4: clarified that no HR will be generated for subsets with less than 10 subjects, added HR by MPR<br>• Section 7.6.4.4: added OS analysis by pCR and MPR without landmark to inform surrogacy<br>• Section 7.7.2: added Death within 30 days and 90 days from surgery and added AE/SAE/related AE leading to death<br>• Section 7.7.20: added section on Covid-19 AEs<br>[redacted]<br>• Section 7.9.1: added predictive analysis between EFS/OS and PD-L1<br>[redacted]<br>• Section 11: updated the list of prior analysis including the pCR analysis

11 PREVIOUS ANALYSES

The following DMC safety meetings occured before approval of this SAP. Analyses were generated for these meetings according to the DMC charter.

- 1st interim safety review for Arms A and B after approximately 15 subjects in each arm completed surgery (14-Mar-2018)
- 2nd interim safety review after approximately 15 subjects in arm C completed surgery (03-Oct-2018)
- Additional safety reviews approximately every 6 months until the primary endpoint of pathological complete response is analyzed (24-May-2019, 04-Dec-2019)

No by treatment data have been share with BMS for these meetings.

The analysis of the pCR primary endpoint was performed with a database lock of 16-Sep-2020. The DMC reviewed the data and BMS was unblinded to treatment arm. A Clinical Study Report was written reporting the demographics, baseline characteristics, surgery related data, pathological response results, clinical response and safety. No EFS/OS data by treatment arm was shared with BMS.

APPENDIX 1 TIME-TO ONSET AND TIME-TO RESOLUTION DEFINITION AND CONVENTIONS FOR SELECT ADVERSE EVENTS, IMMUNE-MEDIATED ADVERSE EVENTS AND EVENTS OF SPECIAL INTEREST

Time-to onset definition

Time-to onset of AE (any grade) for a specific category is defined as the time between the day of the first dose of study treatment and the onset date of the earliest AE (of any grade) in this category.

The time-to onset of AE (grade 3-5) for a specific category is defined similarly with an onset date corresponding to a grade 3-5 AE.

Time-to onset of drug-related AE (any grade or grade 3-5) for a specific category is defined similarly but restricted to drug-related AE.

Time-to onset for a specific subcategory is defined similarly but restricted to event of this subcategory.

Time-to resolution definition

In order to derive the time-to resolution, overlapping or contiguous AEs within a specific category or subcategory will be collapsed into what will be termed "clustered" AEs. For example, if a subject (without pre-treatment AE) experienced an AE from 1st to 5th January, another AE (with different PT but within same category) from 6th to 11th January and same AE from 10th to 12th January, these will be collapsed into one clustered AE from 1st to 12th January. Table 1 is summarizing key derivation steps for each type of clustered AEs.

Time-to resolution of AE (any grade) for a specific category is defined as the longest time from onset to complete resolution or improvement to the grade at baseline among all clustered AEs experienced by the subject in this category per adverse event criteria category. Events which worsened into grade 5 events (death) or have a resolution date equal to the date of death are considered unresolved. If a clustered AE is considered as unresolved, the resolution date will be censored to the last known alive date. Improvement to the grade at baseline implies that all different events in the clustered adverse event should at least have improved to the corresponding (i.e. with same preferred term) baseline grade. This measure is defined only for subjects who experienced at least one AE in the specific category.

The time-to resolution of AE (grade 3-5) for a specific category is defined similarly with an onset date corresponding to a grade 3-5 AE.

Time-to resolution of drug-related AE (any grade or grade 3-5) for a specific category is defined similarly but restricted to drug-related AE.

The time-to resolution of AE (any grade or grade 3-5, drug-related or all) where immune modulating medication was initiated is defined similarly. For data presentation not restricted to IMAE, the additional condition that the subject started an immune modulating medication during the longest AE resolution period will be applied.

Approved v3.0 [redacted]

Time-to resolution for a specific subcategory is defined similarly but restricted to event of this subcategory.

Table 1: Derivation of Clustered AE

Type of clustered AE	Derivation
Any grade	Collapse any on-treatment AE from the same category
Drug-related of any grade	Collapse any on-treatment drug-related AE from the same category
Grade 3-5	Collapse any on-treatment AE from the same category. Resolution will be based on the onset date of the earliest grade 3-5 records (if no grade 3-5 record, clustered AE is excluded)
Drug-related of Grade 3-5	Collapse any on-treatment drug-related AE from the same category Resolution will be based on the onset date of the earliest grade 3-5 record (if no Grade 3-5 record, clustered AE is excluded)

The algorithm for collapsing adverse event records is using the following conventions:

For each subject and specified category, the corresponding adverse event records will be collapsed when:

3) Multiple adverse event records have the same onset date.
4) The onset date of an event record is either the same day or 1 day later than the resolution date of a preceding event record (contiguous events).
5) The onset date of an event record is after the onset date and prior to or on the resolution date of a preceding event record (overlapping events).

APPENDIX 2 MISSING AND PARTIAL RADIOTHERAPY AND SURGERY DATES IMPUTATION ALGORITHMS

Procedures – Imputation Rules.

If reported procedure start date is a full valid date then set start date equal to the date part of procedure start date.

In case of partial date use imputation rules described below:

- If only day is missing then
  - If month and year of procedure match month and year of first dose date then impute as date of first dose;
  - If month and year of procedure don't match month and year of first dose date then impute as first day of that month and year.
- If both day and month are missing, then impute as maximum between 01JAN of the year and date of the first dose;
- If date is completely missing or invalid then leave missing.

Note: Imputation is not applicable to data where start date is not collected (for example "PRIOR RADIOTHERAPY" CRF). Set start date to missing in this case.

If reported end date is a full valid date then set end date equal to the date part of the reported end date.

In case of partial date use imputation rules described below:

- If reported end date is partial then set end date equal to the last possible reported end date based on the partial entered reported end date.
- If reported end date is missing, continuing, unknown or invalid then set end date equal to the most recent database extraction date.

If end date was imputed then compare end date to the death date or last known alive date if subject is not dead. If posterior then end date should be imputed to death date (or last known alive date if subject not dead).

Note: Imputation of partial dates only applies to data entered on "RADIOTHERAPY" CRF page. For other CRF pages in case of partial dates set end date to missing.

Surgeries – Imputation Rules.

If reported surgery date is a full valid date then set start date equal to the date part of surgery date.

In case of partial date, use one of the two imputation rules described below:

A. For data collected on "PRIOR SURGERY RELATED TO CANCER" CRF page:

- If only day is missing then impute as the first day of the month;
- If both day and month are missing then then impute as 01JAN of the year;
- If date is completely missing or invalid then leave missing.

B. For data collected on "SUBSEQUENT SURGERY" CRF page:

- If only day is missing then
  - If month and year of surgery match month and year of first dose date then impute the missing date as the date of first dose;
  - If month and year of surgery don't match month and year of first dose date then impute as first day of that month and year;
- If both day and month are missing then impute as maximum between 01JAN of the year and date of the first dose;
- If date is completely missing or invalid then leave missing.
-
- C. For DEFINITIVE SURGERY CRF page :
- If only day is missing then
  - if month and year of surgery match month and year of first dose date then impute the missing date as the date of first dose;
  - if month and year of surgery match month and year of last neoadjuvant dose date then impute the missing date as the date of last Neoadjuvant dose
  - if month and year of surgery don't match month and year of first dose or last Neoadjuvant dose date then impute as first day of that month and year;
- If both day and month are missing then impute as maximum between 01JAN of the year and date of last Neoadjuvant dose date;
- If date is completely missing or invalid then leave missing.
- For incomplete definitive surgery end date: set to definitive surgery start date

12 REFERENCES

1 Mountain, CA. Regional Lymph node classification for lung cancer staging. Chest. 1997: 1718-23.

2 Goldstraw P, Crowley J, Chansky K, et al for the International Association for the Study of Lung Cancer International Staging Committee. The IASLC Lung Cancer Staging Project: proposals for the revision of the TNM stage grouping in the forthcoming (seventh) edition of the TNM Classification of malignant tumours. J Thorac Onco. 2007; 2(8): 706-14

3 Detterbeck FC, Boffa DJ, Tanoue LT. The new lung cancer staging system. Chest. 2009; 136: 260-71.

4 Provencio et al. Abs 8521 ASCO 2018

5 Baden et al, Comparison of Platforms for Determining Tumor Mutational Burden in Patients With Non-Small Cell Lung Cancer, ESMO 2019

6 Dolan P. Modeling valuations for EuroQol health states. Medical Care 1997;35: 1095-1108

7 The EuroQol Group: EuroQol: A new facility for the measurement of health-related quality of life—The EuroQol Group. Health Policy 16:199-208, 1990.

8 Glimm E., Maurer W., Bretz F. Hierarchical testing of multiple endpoints in group-sequential trials. Stat. Med. 2010;29:219–228.

9 Clopper, CJ and Pearson, ES. The use of confidence or fiducial limits illustrated in the case of the binomial. Biometrika 26: 404-423, 1934.

10 Brookmeyer R. and Crowley J. A confidence interval for the median survival time. Biometrics 38:29-41, 1982

11 Klein, J. P. and Moeschberger, M. L. (1997), Survival Analysis: Techniques for Censored and Truncated Data, New York: Springer-Verlag

12 Kalbfleisch RL. The Statistical Analysis of Failure Time Data. Wiley-Interscience, 2002.

13 Global Biometric Sciences, SAS Analysis Dataset Specification, Unique Adverse Events. Version 2.0, April 22, 2015

14 Statistical Methodology in the Pharmaceutical Sciences/ edited by Berry DA, Chapter 13, Categorical Data Analysis, p 415 and 417 ff., Marcel Dekker, 1990.

15 Greenwood, M. The errors of sampling of the survivorship tables, Reports on Public Health and Statistical Subjects, 33, Appendix 1, HMSO, London, 1926

16 Kalbfleisch, J. D. and Prentice, R. L. (1980), The Statistical Analysis of Failure Time Data, New York: John Wiley & Sons.

17 Adverse Event Domain Requirements Specification Bristol Myers Squibb Co. PRI. Version 2.3.0 April 23, 2018

Approved v3.0 [redacted]

18 Non-Study Medication Domain Requirements Specification Bristol Myers Squibb Co. PRI. Version 2.10.0 April 23, 2018

Approved v3.0 [redacted]
