# Supplementary material for: Association between pathologic response and survival after neoadjuvant therapy in lung cancer
Source: Nat Med. 2023 Oct 30;30(1):218–28. doi: 10.1038/s41591-023-02660-6 (PMC10803255; doi:10.1038/s41591-023-02660-6)
Supplement: Supplementary file 2 — Reporting Summary [file 41591_2023_2660_MOESM2_ESM.pdf]

nature portfolio

Corresponding author(s): Janis M. Taube

Last updated by author(s): Oct 18, 2023

Reporting Summary

Nature Portfolio wishes to improve the reproducibility of the work that we publish. This form provides structure for consistency and transparency in reporting. For further information on Nature Portfolio policies, see our Editorial Policies and the Editorial Policy Checklist.

Statistics

For all statistical analyses, confirm that the following items are present in the figure legend, table legend, main text, or Methods section.

n/a	Confirmed	
☐	☒	The exact sample size (*n*) for each experimental group/condition, given as a discrete number and unit of measurement
☒	☐	A statement on whether measurements were taken from distinct samples or whether the same sample was measured repeatedly
☒	☐	The statistical test(s) used AND whether they are one- or two-sided<br>*Only common tests should be described solely by name; describe more complex techniques in the Methods section.*
☐	☒	A description of all covariates tested
☒	☐	A description of any assumptions or corrections, such as tests of normality and adjustment for multiple comparisons
☐	☒	A full description of the statistical parameters including central tendency (e.g. means) or other basic estimates (e.g. regression coefficient) AND variation (e.g. standard deviation) or associated estimates of uncertainty (e.g. confidence intervals)
☒	☐	For null hypothesis testing, the test statistic (e.g. *F*, *t*, *r*) with confidence intervals, effect sizes, degrees of freedom and *P* value noted<br>*Give P values as exact values whenever suitable.*
☒	☐	For Bayesian analysis, information on the choice of priors and Markov chain Monte Carlo settings
☒	☐	For hierarchical and complex designs, identification of the appropriate level for tests and full reporting of outcomes
☒	☐	Estimates of effect sizes (e.g. Cohen's *d*, Pearson's *r*), indicating how they were calculated

Our web collection on statistics for biologists contains articles on many of the points above.

Software and code

Policy information about availability of computer code

Data collection	Bristol Myers Squibb's Trial Access online (TAO) eWR number 8091 dated 04-Feb-2022 along with eDM/Oracle Clinical Release number 5.4.012r7 dated 10April2023 was used for data collection.
Data analysis	SAS Studio version 9.04.01M7P08062020 (AWS) was used for data analysis.

For manuscripts utilizing custom algorithms or software that are central to the research but not yet described in published literature, software must be made available to editors and reviewers. We strongly encourage code deposition in a community repository (e.g. GitHub). See the Nature Portfolio guidelines for submitting code & software for further information.

Data

Policy information about availability of data

All manuscripts must include a data availability statement. This statement should provide the following information, where applicable:

- Accession codes, unique identifiers, or web links for publicly available datasets
- A description of any restrictions on data availability
- For clinical datasets or third party data, please ensure that the statement adheres to our policy

De-identified and anonymized data will be made available within a secured portal to qualified researchers who submit an in-scope proposal approved by the Independent Review Committee. Proposals will be reviewed to ensure that there is adequate scientific rationale and methodology, a robust statistical analysis plan and a publication plan. Researchers should have relevant experience and demonstrate a plan to address any conflicts of interest, if applicable. Requests will be

reviewed and processed by an independent committee; consequently, Bristol Myers Squibb cannot provide an estimated response time. For more information and to submit a data-sharing request, please visit https://www.bms.com/researchers-and-partners/independent-research/data-sharing-request-process.html.

Research involving human participants, their data, or biological material

Policy information about studies with human participants or human data. See also policy information about sex, gender (identity/presentation), and sexual orientation and race, ethnicity and racism.

Reporting on sex and gender	Both male and female patients were eligible for enrollment. The number of male and female patients randomized in this study has been previously reported (Forde PM, Spicer J, Lu S, et al. Neoadjuvant nivolumab plus chemotherapy in resectable lung cancer. N. Engl. J. Med. 386, 1973–1985 (2022)), and the number of male and female patients in the path-evaluable population is reported in Table 1. There are no analyses based on sex and gender reported in this manuscript.
Reporting on race, ethnicity, or other socially relevant groupings	The number of patients by geographic region randomized in this study has been previously reported (Forde PM, Spicer J, Lu S, et al. Neoadjuvant nivolumab plus chemotherapy in resectable lung cancer. N. Engl. J. Med. 386, 1973–1985 (2022)), and the number of patients by geographic region in the path-evaluable population is reported in Supplementary Table 1. There are no analyses based on race or ethnicity reported in this manuscript.
Population characteristics	Baseline patient demographics and disease characteristics for patients randomized in this study have been previously reported (Forde PM, Spicer J, Lu S, et al. Neoadjuvant nivolumab plus chemotherapy in resectable lung cancer. N. Engl. J. Med. 386, 1973–1985 (2022)), and baseline demographics and disease characteristics for the path-evaluable population are reported in Supplementary Table 1.
Recruitment	From March 2017 through November 2019, a total of 773 patients were enrolled at study sites in 14 different countries.
Ethics oversight	The extended pathologic analysis of resection specimens reported herein was conducted at Johns Hopkins and was approved by the Johns Hopkins University IRB (IRB00122321). The study protocol for the parent trial and all amendments were approved by an institutional review board or independent ethics committee at each study site, and an independent data and safety monitoring committee reviewed/monitored the efficacy and safety of all evaluated treatments. A list of investigators and study sites was previously published (Forde PM, Spicer J, Lu S, et al. Neoadjuvant nivolumab plus chemotherapy in resectable lung cancer. N. Engl. J. Med. 386, 1973–1985 (2022)).

Note that full information on the approval of the study protocol must also be provided in the manuscript.

Field-specific reporting

Please select the one below that is the best fit for your research. If you are not sure, read the appropriate sections before making your selection.

☒ Life sciences ☐ Behavioural & social sciences ☐ Ecological, evolutionary & environmental sciences

For a reference copy of the document with all sections, see nature.com/documents/nr-reporting-summary-flat.pdf

Life sciences study design

All studies must disclose on these points even when the disclosure is negative.

Sample size	Approximately 350 patients were planned for concurrent randomization to nivolumab plus chemotherapy and chemotherapy. This sample size was based on the primary end points of pathologic complete response and event-free survival with 0.01 and 0.04 type I error allocation (two-sided) respectively. If the pathologic complete response comparison was significant, the 0.01 alpha was planned to be re-allocated to the event-free survival comparison which would be based on a two-sided type I error of 0.05.
Data exclusions	No data were excluded from the reported analyses.
Replication	Attempts of replication were not performed given that CheckMate 816 is a clinical trial.
Randomization	Patients were randomly assigned in a 1:1 ratio to receive nivolumab plus platinum-doublet chemotherapy or platinum-doublet chemotherapy alone before undergoing definitive surgery. A third group that received nivolumab plus ipilimumab closed enrollment early on the basis of external trial data reported during the trial.
Blinding	CheckMate 816 is an open-label study; blinding procedures between participants and investigators are not applicable. The blinded independent pathologic reviews and and blinded independent central reviews were blinded.

Reporting for specific materials, systems and methods

We require information from authors about some types of materials, experimental systems and methods used in many studies. Here, indicate whether each material, system or method listed is relevant to your study. If you are not sure if a list item applies to your research, read the appropriate section before selecting a response.

Materials & experimental systems

n/a	Involved in the study
☐	☒ Antibodies
☒	☐ Eukaryotic cell lines
☒	☐ Palaeontology and archaeology
☒	☐ Animals and other organisms
☐	☒ Clinical data
☒	☐ Dual use research of concern
☒	☐ Plants

Methods

n/a	Involved in the study
☒	☐ ChIP-seq
☒	☐ Flow cytometry
☒	☐ MRI-based neuroimaging

Antibodies

Antibodies used	Nivolumab, a fully human anti–programmed death 1 (PD-1) antibody, was administered as the experimental treatment in this study. Nivolumab administered as part of this study was provided by the study's sponsor (Bristol Myers Squibb).
Validation	Neoadjuvant nivolumab plus chemotherapy for patients with resectable non-small cell lung carcinoma was evaluated in this study as part of Bristol Myers Squibb's clinical study program.

Clinical data

Policy information about clinical studies

All manuscripts should comply with the ICMJE guidelines for publication of clinical research and a completed CONSORT checklist must be included with all submissions.

Clinical trial registration	NCT02998528
Study protocol	The study protocol is provided in the Supplementary Information.
Data collection	From March 2017 through November 2019, a total of 773 patients were enrolled at study sites in 14 different countries. Patients were randomly assigned in a 1:1 ratio to receive nivolumab plus platinum-doublet chemotherapy or platinum-doublet chemotherapy alone before undergoing definitive surgery. Pathologic complete response (pCR), major pathologic response, and % residual viable tumor cells data are from the final analysis of pCR (September 16, 2020), whereas all other efficacy and safety results are from the prespecified interim analysis 1 of event-free survival (October 20, 2021; final analysis for event-free survival). A list of investigators and study sites where data were collected was previously published (Forde PM, Spicer J, Lu S, et al. Neoadjuvant nivolumab plus chemotherapy in resectable lung cancer. N. Engl. J. Med. 386, 1973–1985 (2022)).
Outcomes	There were two primary end points, event-free survival (EFS) according to blinded independent central review and pathologic complete response (pCR; 0% residual viable tumor cells (RVT) in the primary tumor (PT) and sampled lymph nodes (LN)) according to blinded independent pathological review. Secondary end points included major pathological response (MPR; ≤10% residual viable tumor cells in the PT and sampled LNs), time to death or distant metastases, and overall survival. Adverse events were assessed in all the treated patients. Clinical and biomarker assessments were performed during the course of the trial. Each specimen was scored for pathologic response per blinded independent pathologic review using pan-tumor irPRC. pCR was defined as 0% and MPR as ≤10% RVT in both the PT and LNs (pCR-PT, MPR-PT, pCR-LN, and MPR-LN, respectively). The association of different pathologic response categories and associated histologic features with EFS were assessed in the overall path-evaluable population and subpopulations by LN involvement. We also evaluated relationships between %RVT and radiographic response, tumor PD-L1 expression, tumor mutational burden, and ctDNA clearance before definitive surgery.
